# Supplementary figures and images for: Adaptive mask-based brain extraction method for head CT images (part 2 of 14)
Source: PLoS One. 2024 Mar 11;19(3):e0295536. doi: 10.1371/journal.pone.0295536 (PMC10927156; doi:10.1371/journal.pone.0295536)

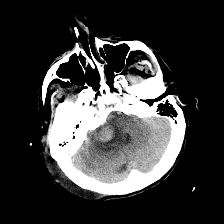

Supplement: S1 Data — (ZIP) [file pone.0295536.s002.zip › S2_Data/traindata_224/traindata/0/IM_0005-ID_2f96fefac.png]

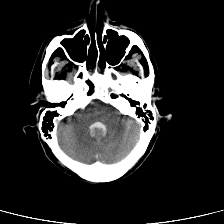

Supplement: S1 Data — (ZIP) [file pone.0295536.s002.zip › S2_Data/traindata_224/traindata/0/IM_0005-ID_39938271b.png]

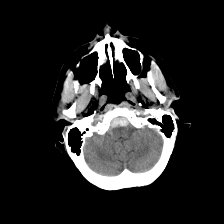

Supplement: S1 Data — (ZIP) [file pone.0295536.s002.zip › S2_Data/traindata_224/traindata/0/IM_0005-ID_3a57d214c.png]

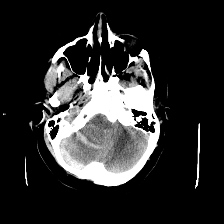

Supplement: S1 Data — (ZIP) [file pone.0295536.s002.zip › S2_Data/traindata_224/traindata/0/IM_0005-ID_3e860d214.png]

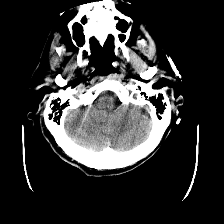

Supplement: S1 Data — (ZIP) [file pone.0295536.s002.zip › S2_Data/traindata_224/traindata/0/IM_0005-ID_41348a09c.png]

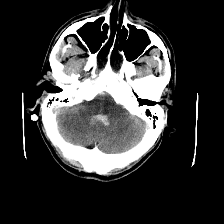

Supplement: S1 Data — (ZIP) [file pone.0295536.s002.zip › S2_Data/traindata_224/traindata/0/IM_0005-ID_4a22483bb.png]

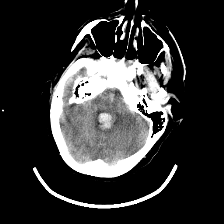

Supplement: S1 Data — (ZIP) [file pone.0295536.s002.zip › S2_Data/traindata_224/traindata/0/IM_0005-ID_4ba455613.png]

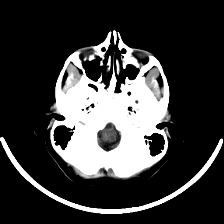

Supplement: S1 Data — (ZIP) [file pone.0295536.s002.zip › S2_Data/traindata_224/traindata/0/IM_0005-ID_5092a74dc.png]

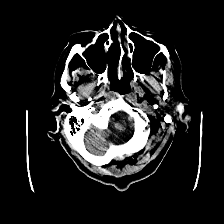

Supplement: S1 Data — (ZIP) [file pone.0295536.s002.zip › S2_Data/traindata_224/traindata/0/IM_0005-ID_52231bc36.png]

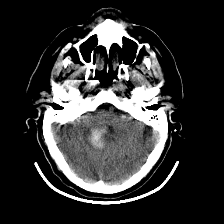

Supplement: S1 Data — (ZIP) [file pone.0295536.s002.zip › S2_Data/traindata_224/traindata/0/IM_0005-ID_556833e85.png]

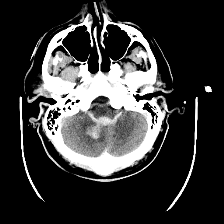

Supplement: S1 Data — (ZIP) [file pone.0295536.s002.zip › S2_Data/traindata_224/traindata/0/IM_0005-ID_55fd2b447.png]

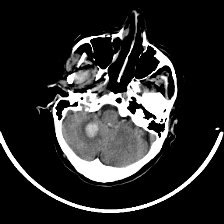

Supplement: S1 Data — (ZIP) [file pone.0295536.s002.zip › S2_Data/traindata_224/traindata/0/IM_0005-ID_5bc861901.png]

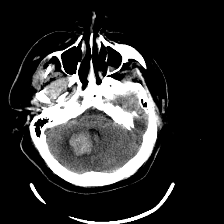

Supplement: S1 Data — (ZIP) [file pone.0295536.s002.zip › S2_Data/traindata_224/traindata/0/IM_0005-ID_61c5b974b.png]

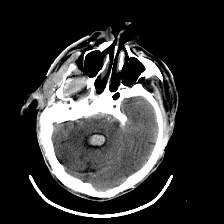

Supplement: S1 Data — (ZIP) [file pone.0295536.s002.zip › S2_Data/traindata_224/traindata/0/IM_0005-ID_63ad7bffc.png]

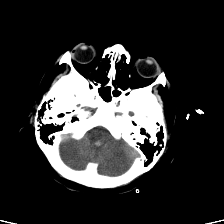

Supplement: S1 Data — (ZIP) [file pone.0295536.s002.zip › S2_Data/traindata_224/traindata/0/IM_0005-ID_65470871b.png]

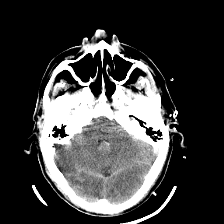

Supplement: S1 Data — (ZIP) [file pone.0295536.s002.zip › S2_Data/traindata_224/traindata/0/IM_0005-ID_65d5b4c9f.png]

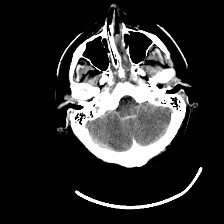

Supplement: S1 Data — (ZIP) [file pone.0295536.s002.zip › S2_Data/traindata_224/traindata/0/IM_0005-ID_6859101fc.png]

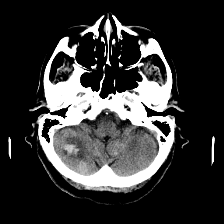

Supplement: S1 Data — (ZIP) [file pone.0295536.s002.zip › S2_Data/traindata_224/traindata/0/IM_0005-ID_6b5132d81.png]

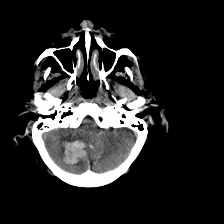

Supplement: S1 Data — (ZIP) [file pone.0295536.s002.zip › S2_Data/traindata_224/traindata/0/IM_0005-ID_6db4623dd.png]

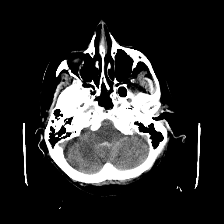

Supplement: S1 Data — (ZIP) [file pone.0295536.s002.zip › S2_Data/traindata_224/traindata/0/IM_0005-ID_73b3c1c67.png]

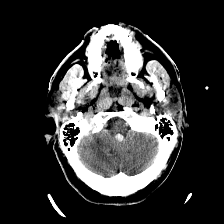

Supplement: S1 Data — (ZIP) [file pone.0295536.s002.zip › S2_Data/traindata_224/traindata/0/IM_0005-ID_743cfbcb6.png]

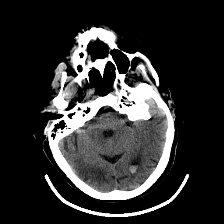

Supplement: S1 Data — (ZIP) [file pone.0295536.s002.zip › S2_Data/traindata_224/traindata/0/IM_0005-ID_74690521b.png]

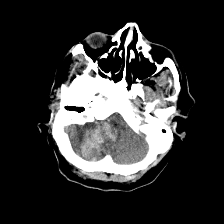

Supplement: S1 Data — (ZIP) [file pone.0295536.s002.zip › S2_Data/traindata_224/traindata/0/IM_0005-ID_7544b6fc3.png]

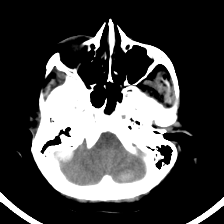

Supplement: S1 Data — (ZIP) [file pone.0295536.s002.zip › S2_Data/traindata_224/traindata/0/IM_0005-ID_76127d9a0.png]

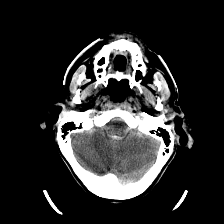

Supplement: S1 Data — (ZIP) [file pone.0295536.s002.zip › S2_Data/traindata_224/traindata/0/IM_0005-ID_77a83dadf.png]

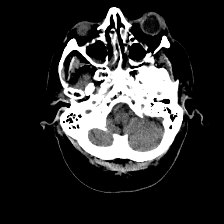

Supplement: S1 Data — (ZIP) [file pone.0295536.s002.zip › S2_Data/traindata_224/traindata/0/IM_0005-ID_8504f361d.png]

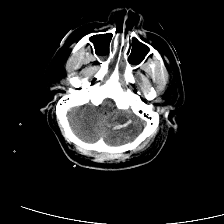

Supplement: S1 Data — (ZIP) [file pone.0295536.s002.zip › S2_Data/traindata_224/traindata/0/IM_0005-ID_8cb590ee9.png]

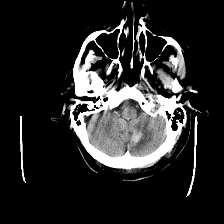

Supplement: S1 Data — (ZIP) [file pone.0295536.s002.zip › S2_Data/traindata_224/traindata/0/IM_0005-ID_94b63da36.png]

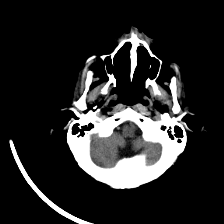

Supplement: S1 Data — (ZIP) [file pone.0295536.s002.zip › S2_Data/traindata_224/traindata/0/IM_0005-ID_968ee90c3.png]

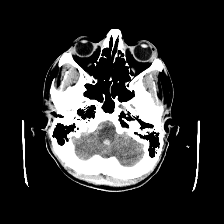

Supplement: S1 Data — (ZIP) [file pone.0295536.s002.zip › S2_Data/traindata_224/traindata/0/IM_0005-ID_99f7552d8.png]

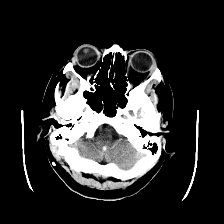

Supplement: S1 Data — (ZIP) [file pone.0295536.s002.zip › S2_Data/traindata_224/traindata/0/IM_0005-ID_9e97be164.png]

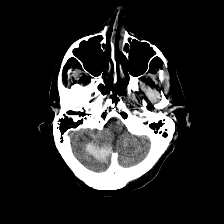

Supplement: S1 Data — (ZIP) [file pone.0295536.s002.zip › S2_Data/traindata_224/traindata/0/IM_0005-ID_a67fb2291.png]

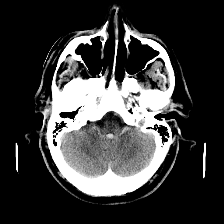

Supplement: S1 Data — (ZIP) [file pone.0295536.s002.zip › S2_Data/traindata_224/traindata/0/IM_0005-ID_aeb68590d.png]

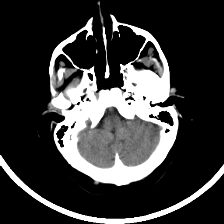

Supplement: S1 Data — (ZIP) [file pone.0295536.s002.zip › S2_Data/traindata_224/traindata/0/IM_0005-ID_b9c52fff2.png]

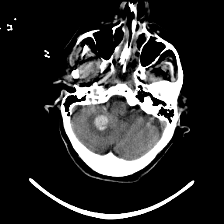

Supplement: S1 Data — (ZIP) [file pone.0295536.s002.zip › S2_Data/traindata_224/traindata/0/IM_0005-ID_bc68fe186.png]

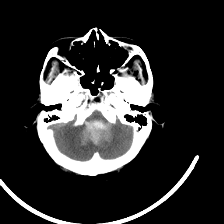

Supplement: S1 Data — (ZIP) [file pone.0295536.s002.zip › S2_Data/traindata_224/traindata/0/IM_0005-ID_bdaa860d4.png]

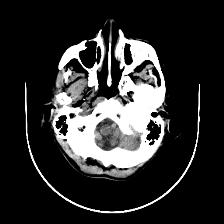

Supplement: S1 Data — (ZIP) [file pone.0295536.s002.zip › S2_Data/traindata_224/traindata/0/IM_0005-ID_c769b4372.png]

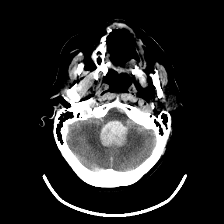

Supplement: S1 Data — (ZIP) [file pone.0295536.s002.zip › S2_Data/traindata_224/traindata/0/IM_0005-ID_d13bd523a.png]

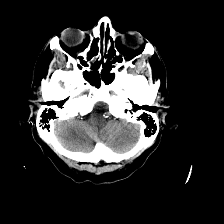

Supplement: S1 Data — (ZIP) [file pone.0295536.s002.zip › S2_Data/traindata_224/traindata/0/IM_0005-ID_d55502da3.png]

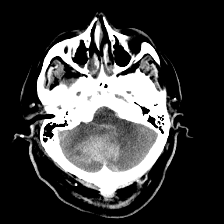

Supplement: S1 Data — (ZIP) [file pone.0295536.s002.zip › S2_Data/traindata_224/traindata/0/IM_0005-ID_e2b33235e.png]

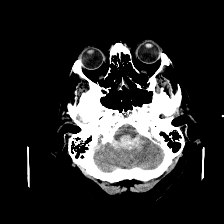

Supplement: S1 Data — (ZIP) [file pone.0295536.s002.zip › S2_Data/traindata_224/traindata/0/IM_0005-ID_e6dbf0b5f.png]

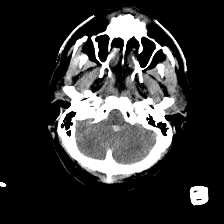

Supplement: S1 Data — (ZIP) [file pone.0295536.s002.zip › S2_Data/traindata_224/traindata/0/IM_0005-ID_ed309646d.png]

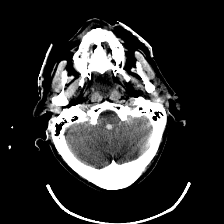

Supplement: S1 Data — (ZIP) [file pone.0295536.s002.zip › S2_Data/traindata_224/traindata/0/IM_0005-ID_f03fbb264.png]

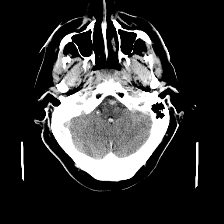

Supplement: S1 Data — (ZIP) [file pone.0295536.s002.zip › S2_Data/traindata_224/traindata/0/IM_0005-ID_f9fc621c6.png]

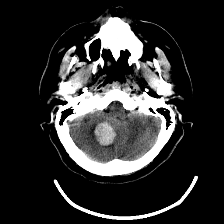

Supplement: S1 Data — (ZIP) [file pone.0295536.s002.zip › S2_Data/traindata_224/traindata/0/IM_0005-ID_fe402e6b2.png]

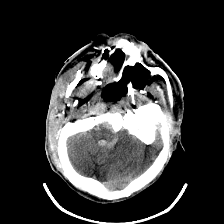

Supplement: S1 Data — (ZIP) [file pone.0295536.s002.zip › S2_Data/traindata_224/traindata/0/IM_0005-ID_fed2cbae0.png]

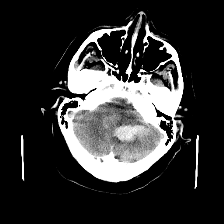

Supplement: S1 Data — (ZIP) [file pone.0295536.s002.zip › S2_Data/traindata_224/traindata/0/IM_0006-ID_002251d48.png]

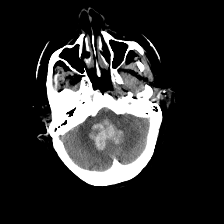

Supplement: S1 Data — (ZIP) [file pone.0295536.s002.zip › S2_Data/traindata_224/traindata/0/IM_0006-ID_036b608a9.png]

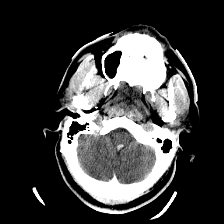

Supplement: S1 Data — (ZIP) [file pone.0295536.s002.zip › S2_Data/traindata_224/traindata/0/IM_0006-ID_05635c84d.png]

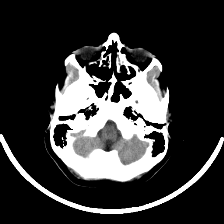

Supplement: S1 Data — (ZIP) [file pone.0295536.s002.zip › S2_Data/traindata_224/traindata/0/IM_0006-ID_09389a287.png]

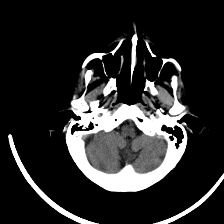

Supplement: S1 Data — (ZIP) [file pone.0295536.s002.zip › S2_Data/traindata_224/traindata/0/IM_0006-ID_0b22cad84.png]

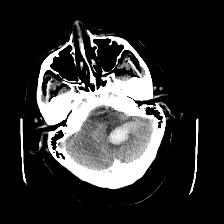

Supplement: S1 Data — (ZIP) [file pone.0295536.s002.zip › S2_Data/traindata_224/traindata/0/IM_0006-ID_0b285b9ca.png]

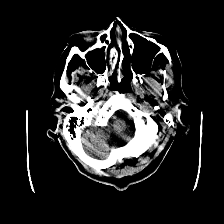

Supplement: S1 Data — (ZIP) [file pone.0295536.s002.zip › S2_Data/traindata_224/traindata/0/IM_0006-ID_0c52bc1cc.png]

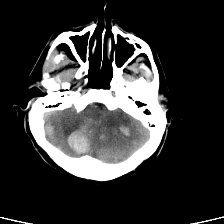

Supplement: S1 Data — (ZIP) [file pone.0295536.s002.zip › S2_Data/traindata_224/traindata/0/IM_0006-ID_0ee040c60.png]

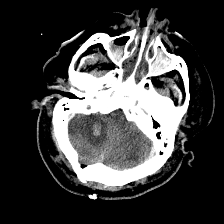

Supplement: S1 Data — (ZIP) [file pone.0295536.s002.zip › S2_Data/traindata_224/traindata/0/IM_0006-ID_1036b45a8.png]

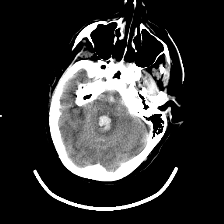

Supplement: S1 Data — (ZIP) [file pone.0295536.s002.zip › S2_Data/traindata_224/traindata/0/IM_0006-ID_10465dafc.png]

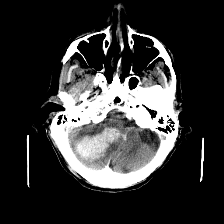

Supplement: S1 Data — (ZIP) [file pone.0295536.s002.zip › S2_Data/traindata_224/traindata/0/IM_0006-ID_10dd49a55.png]

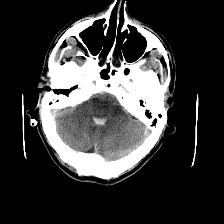

Supplement: S1 Data — (ZIP) [file pone.0295536.s002.zip › S2_Data/traindata_224/traindata/0/IM_0006-ID_11e6ad569.png]

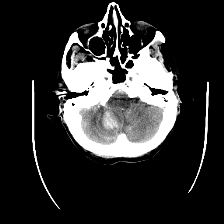

Supplement: S1 Data — (ZIP) [file pone.0295536.s002.zip › S2_Data/traindata_224/traindata/0/IM_0006-ID_1aa28e21d.png]

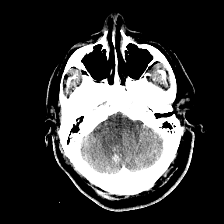

Supplement: S1 Data — (ZIP) [file pone.0295536.s002.zip › S2_Data/traindata_224/traindata/0/IM_0006-ID_223ade9af.png]

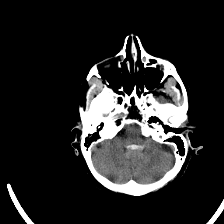

Supplement: S1 Data — (ZIP) [file pone.0295536.s002.zip › S2_Data/traindata_224/traindata/0/IM_0006-ID_27d718787.png]

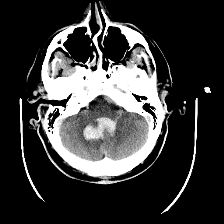

Supplement: S1 Data — (ZIP) [file pone.0295536.s002.zip › S2_Data/traindata_224/traindata/0/IM_0006-ID_2aa41ca57.png]

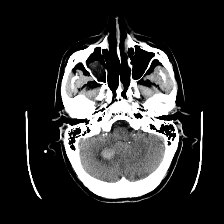

Supplement: S1 Data — (ZIP) [file pone.0295536.s002.zip › S2_Data/traindata_224/traindata/0/IM_0006-ID_2e266f901.png]

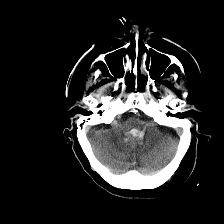

Supplement: S1 Data — (ZIP) [file pone.0295536.s002.zip › S2_Data/traindata_224/traindata/0/IM_0006-ID_3014238ac.png]

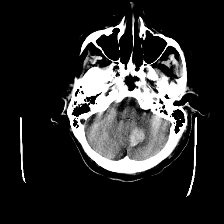

Supplement: S1 Data — (ZIP) [file pone.0295536.s002.zip › S2_Data/traindata_224/traindata/0/IM_0006-ID_316935dfd.png]

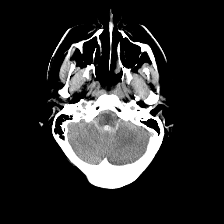

Supplement: S1 Data — (ZIP) [file pone.0295536.s002.zip › S2_Data/traindata_224/traindata/0/IM_0006-ID_36be11832.png]

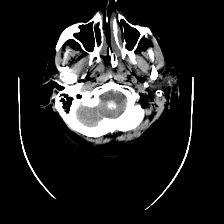

Supplement: S1 Data — (ZIP) [file pone.0295536.s002.zip › S2_Data/traindata_224/traindata/0/IM_0006-ID_3714c25ac.png]

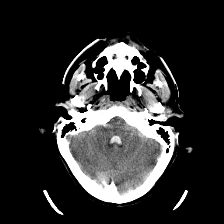

Supplement: S1 Data — (ZIP) [file pone.0295536.s002.zip › S2_Data/traindata_224/traindata/0/IM_0006-ID_37c65aa7b.png]

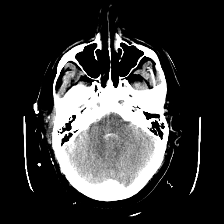

Supplement: S1 Data — (ZIP) [file pone.0295536.s002.zip › S2_Data/traindata_224/traindata/0/IM_0006-ID_3f3c0ebd0.png]

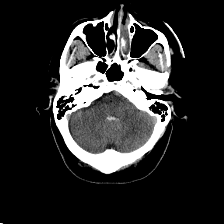

Supplement: S1 Data — (ZIP) [file pone.0295536.s002.zip › S2_Data/traindata_224/traindata/0/IM_0006-ID_5030b5552.png]

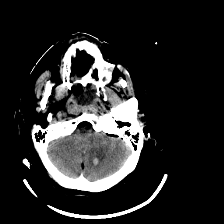

Supplement: S1 Data — (ZIP) [file pone.0295536.s002.zip › S2_Data/traindata_224/traindata/0/IM_0006-ID_5146a95c0.png]

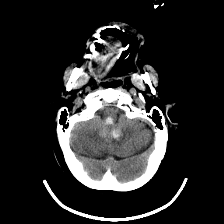

Supplement: S1 Data — (ZIP) [file pone.0295536.s002.zip › S2_Data/traindata_224/traindata/0/IM_0006-ID_51ddb206d.png]

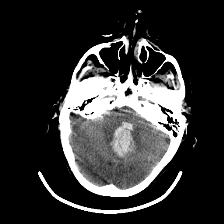

Supplement: S1 Data — (ZIP) [file pone.0295536.s002.zip › S2_Data/traindata_224/traindata/0/IM_0006-ID_5cabd9ce5.png]

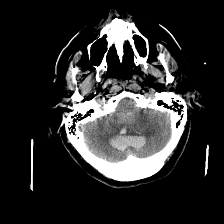

Supplement: S1 Data — (ZIP) [file pone.0295536.s002.zip › S2_Data/traindata_224/traindata/0/IM_0006-ID_5daa789db.png]

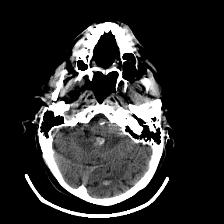

Supplement: S1 Data — (ZIP) [file pone.0295536.s002.zip › S2_Data/traindata_224/traindata/0/IM_0006-ID_5e4513613.png]

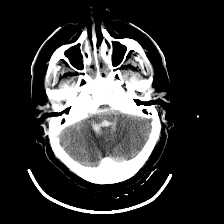

Supplement: S1 Data — (ZIP) [file pone.0295536.s002.zip › S2_Data/traindata_224/traindata/0/IM_0006-ID_5ebee68a1.png]

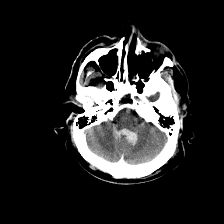

Supplement: S1 Data — (ZIP) [file pone.0295536.s002.zip › S2_Data/traindata_224/traindata/0/IM_0006-ID_63d154b5b.png]

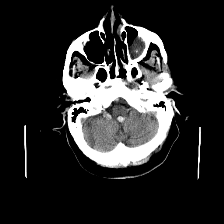

Supplement: S1 Data — (ZIP) [file pone.0295536.s002.zip › S2_Data/traindata_224/traindata/0/IM_0006-ID_64b80423e.png]

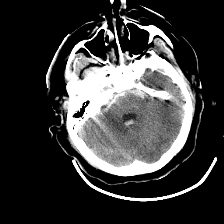

Supplement: S1 Data — (ZIP) [file pone.0295536.s002.zip › S2_Data/traindata_224/traindata/0/IM_0006-ID_6bb18579c.png]

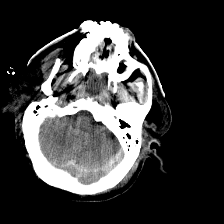

Supplement: S1 Data — (ZIP) [file pone.0295536.s002.zip › S2_Data/traindata_224/traindata/0/IM_0006-ID_6e3209e3a.png]

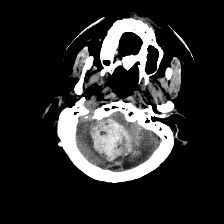

Supplement: S1 Data — (ZIP) [file pone.0295536.s002.zip › S2_Data/traindata_224/traindata/0/IM_0006-ID_6fc93dc0a.png]

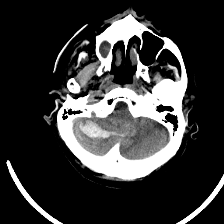

Supplement: S1 Data — (ZIP) [file pone.0295536.s002.zip › S2_Data/traindata_224/traindata/0/IM_0006-ID_72b24af72.png]

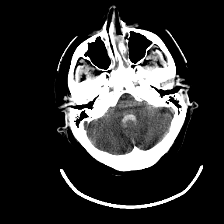

Supplement: S1 Data — (ZIP) [file pone.0295536.s002.zip › S2_Data/traindata_224/traindata/0/IM_0006-ID_786517811.png]

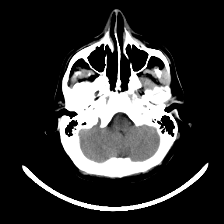

Supplement: S1 Data — (ZIP) [file pone.0295536.s002.zip › S2_Data/traindata_224/traindata/0/IM_0006-ID_7d624e9c2.png]

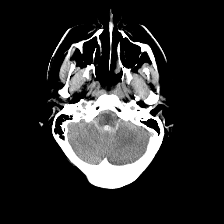

Supplement: S1 Data — (ZIP) [file pone.0295536.s002.zip › S2_Data/traindata_224/traindata/0/IM_0006-ID_7fe01a7b6.png]

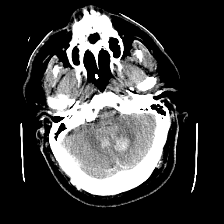

Supplement: S1 Data — (ZIP) [file pone.0295536.s002.zip › S2_Data/traindata_224/traindata/0/IM_0006-ID_8138770cc.png]

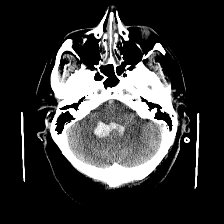

Supplement: S1 Data — (ZIP) [file pone.0295536.s002.zip › S2_Data/traindata_224/traindata/0/IM_0006-ID_82c4d8f0a.png]

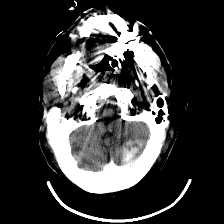

Supplement: S1 Data — (ZIP) [file pone.0295536.s002.zip › S2_Data/traindata_224/traindata/0/IM_0006-ID_8370d1270.png]

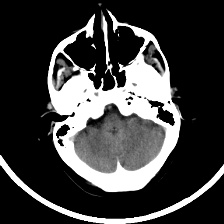

Supplement: S1 Data — (ZIP) [file pone.0295536.s002.zip › S2_Data/traindata_224/traindata/0/IM_0006-ID_83fbf00e7.png]

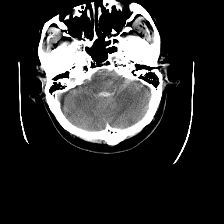

Supplement: S1 Data — (ZIP) [file pone.0295536.s002.zip › S2_Data/traindata_224/traindata/0/IM_0006-ID_845bb7e7d.png]

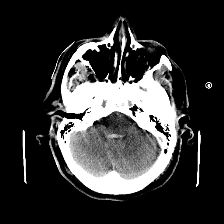

Supplement: S1 Data — (ZIP) [file pone.0295536.s002.zip › S2_Data/traindata_224/traindata/0/IM_0006-ID_86c9f4675.png]

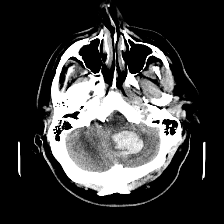

Supplement: S1 Data — (ZIP) [file pone.0295536.s002.zip › S2_Data/traindata_224/traindata/0/IM_0006-ID_88013bf52.png]

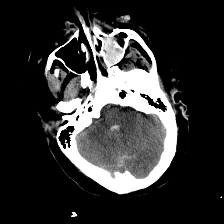

Supplement: S1 Data — (ZIP) [file pone.0295536.s002.zip › S2_Data/traindata_224/traindata/0/IM_0006-ID_8c1f8b007.png]

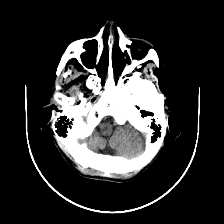

Supplement: S1 Data — (ZIP) [file pone.0295536.s002.zip › S2_Data/traindata_224/traindata/0/IM_0006-ID_8cde4bb3a.png]

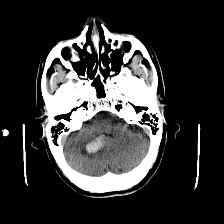

Supplement: S1 Data — (ZIP) [file pone.0295536.s002.zip › S2_Data/traindata_224/traindata/0/IM_0006-ID_8f880f968.png]

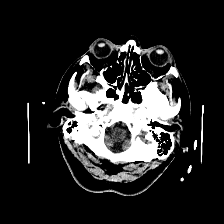

Supplement: S1 Data — (ZIP) [file pone.0295536.s002.zip › S2_Data/traindata_224/traindata/0/IM_0006-ID_91f96d54e.png]

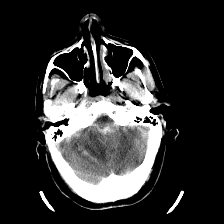

Supplement: S1 Data — (ZIP) [file pone.0295536.s002.zip › S2_Data/traindata_224/traindata/0/IM_0006-ID_9210554bb.png]

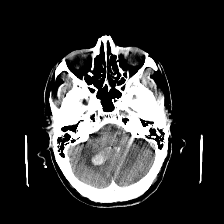

Supplement: S1 Data — (ZIP) [file pone.0295536.s002.zip › S2_Data/traindata_224/traindata/0/IM_0006-ID_94c03ca91.png]

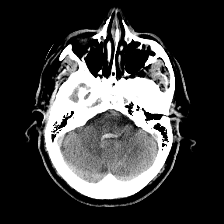

Supplement: S1 Data — (ZIP) [file pone.0295536.s002.zip › S2_Data/traindata_224/traindata/0/IM_0006-ID_9aa2f246e.png]

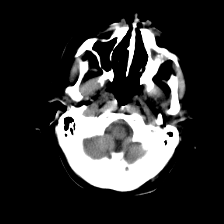

Supplement: S1 Data — (ZIP) [file pone.0295536.s002.zip › S2_Data/traindata_224/traindata/0/IM_0006-ID_9af8955d8.png]
