# Supplementary figures and images for: Adaptive mask-based brain extraction method for head CT images (part 1 of 14)
Source: PLoS One. 2024 Mar 11;19(3):e0295536. doi: 10.1371/journal.pone.0295536 (PMC10927156; doi:10.1371/journal.pone.0295536)

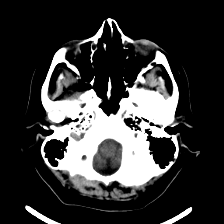

Supplement: S1 Data — (ZIP) [file pone.0295536.s002.zip › S2_Data/traindata_224/traindata/0/80IM_0004-ID_6c680710a.png]

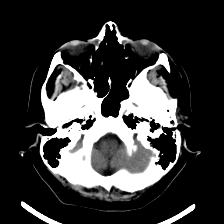

Supplement: S1 Data — (ZIP) [file pone.0295536.s002.zip › S2_Data/traindata_224/traindata/0/80IM_0005-ID_49dbe89d0.png]

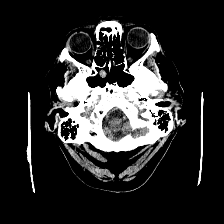

Supplement: S1 Data — (ZIP) [file pone.0295536.s002.zip › S2_Data/traindata_224/traindata/0/81IM_0002-ID_5b983c53a.png]

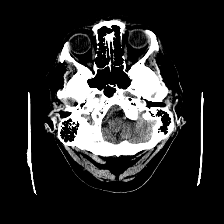

Supplement: S1 Data — (ZIP) [file pone.0295536.s002.zip › S2_Data/traindata_224/traindata/0/81IM_0003-ID_9701b509e.png]

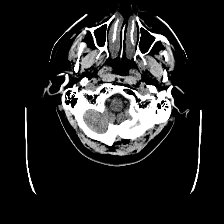

Supplement: S1 Data — (ZIP) [file pone.0295536.s002.zip › S2_Data/traindata_224/traindata/0/82IM_0006-ID_c343e78c7.png]

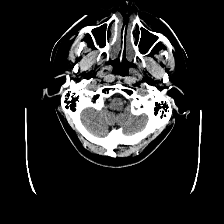

Supplement: S1 Data — (ZIP) [file pone.0295536.s002.zip › S2_Data/traindata_224/traindata/0/82IM_0007-ID_862a73233.png]

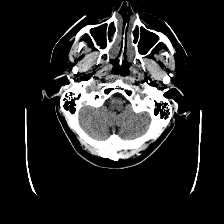

Supplement: S1 Data — (ZIP) [file pone.0295536.s002.zip › S2_Data/traindata_224/traindata/0/82IM_0008-ID_3690fcd9d.png]

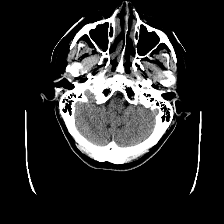

Supplement: S1 Data — (ZIP) [file pone.0295536.s002.zip › S2_Data/traindata_224/traindata/0/82IM_0010-ID_72a42a1bc.png]

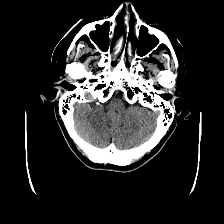

Supplement: S1 Data — (ZIP) [file pone.0295536.s002.zip › S2_Data/traindata_224/traindata/0/82IM_0011-ID_7db225051.png]

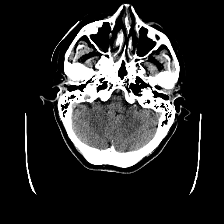

Supplement: S1 Data — (ZIP) [file pone.0295536.s002.zip › S2_Data/traindata_224/traindata/0/82IM_0012-ID_1cb650bef.png]

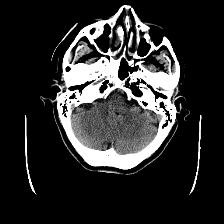

Supplement: S1 Data — (ZIP) [file pone.0295536.s002.zip › S2_Data/traindata_224/traindata/0/82IM_0013-ID_e2a827d94.png]

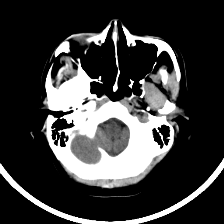

Supplement: S1 Data — (ZIP) [file pone.0295536.s002.zip › S2_Data/traindata_224/traindata/0/84IM_0003-ID_d683dddd5.png]

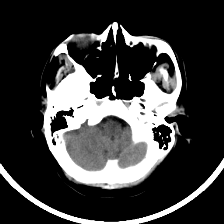

Supplement: S1 Data — (ZIP) [file pone.0295536.s002.zip › S2_Data/traindata_224/traindata/0/84IM_0004-ID_bbc14d45a.png]

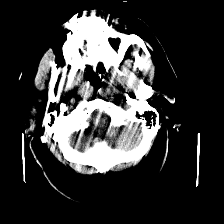

Supplement: S1 Data — (ZIP) [file pone.0295536.s002.zip › S2_Data/traindata_224/traindata/0/85IM_0003-ID_9b45fb7f4.png]

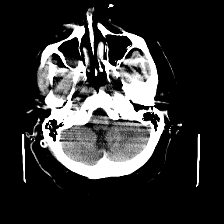

Supplement: S1 Data — (ZIP) [file pone.0295536.s002.zip › S2_Data/traindata_224/traindata/0/85IM_0005-ID_b4f61bf5d.png]

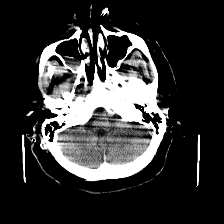

Supplement: S1 Data — (ZIP) [file pone.0295536.s002.zip › S2_Data/traindata_224/traindata/0/85IM_0006-ID_a61618439.png]

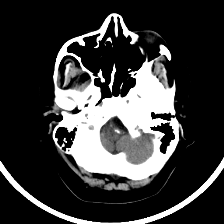

Supplement: S1 Data — (ZIP) [file pone.0295536.s002.zip › S2_Data/traindata_224/traindata/0/86IM_0005-ID_d6ac6e9f7.png]

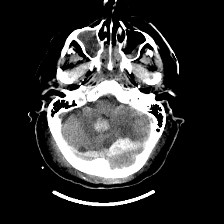

Supplement: S1 Data — (ZIP) [file pone.0295536.s002.zip › S2_Data/traindata_224/traindata/0/IM_0000-ID_176d479f0.png]

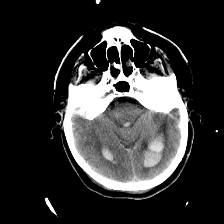

Supplement: S1 Data — (ZIP) [file pone.0295536.s002.zip › S2_Data/traindata_224/traindata/0/IM_0000-ID_7a03bfffe.png]

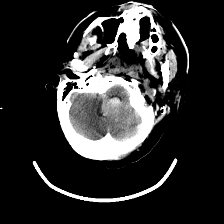

Supplement: S1 Data — (ZIP) [file pone.0295536.s002.zip › S2_Data/traindata_224/traindata/0/IM_0000-ID_d9b2ec9d8.png]

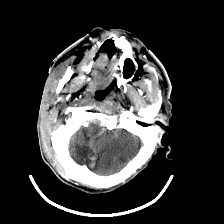

Supplement: S1 Data — (ZIP) [file pone.0295536.s002.zip › S2_Data/traindata_224/traindata/0/IM_0001-ID_1af1e5400.png]

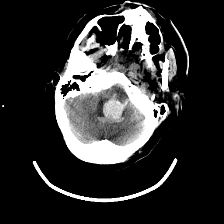

Supplement: S1 Data — (ZIP) [file pone.0295536.s002.zip › S2_Data/traindata_224/traindata/0/IM_0001-ID_a6c4d9a1f.png]

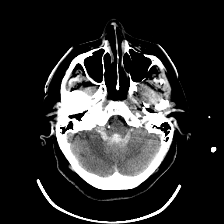

Supplement: S1 Data — (ZIP) [file pone.0295536.s002.zip › S2_Data/traindata_224/traindata/0/IM_0001-ID_f966aa3f7.png]

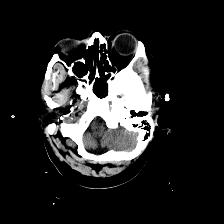

Supplement: S1 Data — (ZIP) [file pone.0295536.s002.zip › S2_Data/traindata_224/traindata/0/IM_0002-ID_051765753.png]

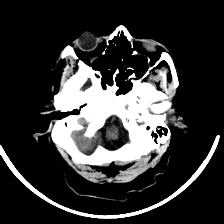

Supplement: S1 Data — (ZIP) [file pone.0295536.s002.zip › S2_Data/traindata_224/traindata/0/IM_0002-ID_294069349.png]

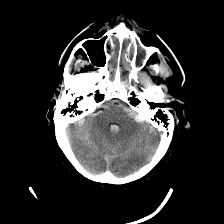

Supplement: S1 Data — (ZIP) [file pone.0295536.s002.zip › S2_Data/traindata_224/traindata/0/IM_0002-ID_3171d7dd5.png]

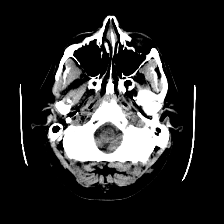

Supplement: S1 Data — (ZIP) [file pone.0295536.s002.zip › S2_Data/traindata_224/traindata/0/IM_0002-ID_326bf9943.png]

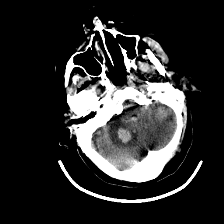

Supplement: S1 Data — (ZIP) [file pone.0295536.s002.zip › S2_Data/traindata_224/traindata/0/IM_0002-ID_57ba5653e.png]

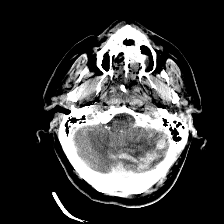

Supplement: S1 Data — (ZIP) [file pone.0295536.s002.zip › S2_Data/traindata_224/traindata/0/IM_0002-ID_650fa637e.png]

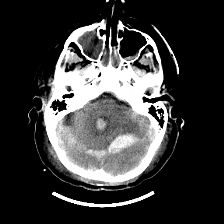

Supplement: S1 Data — (ZIP) [file pone.0295536.s002.zip › S2_Data/traindata_224/traindata/0/IM_0002-ID_b046104e3.png]

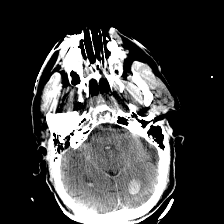

Supplement: S1 Data — (ZIP) [file pone.0295536.s002.zip › S2_Data/traindata_224/traindata/0/IM_0002-ID_b48466cfe.png]

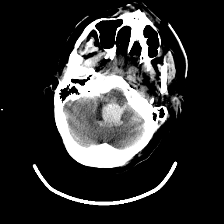

Supplement: S1 Data — (ZIP) [file pone.0295536.s002.zip › S2_Data/traindata_224/traindata/0/IM_0002-ID_cbd45c817.png]

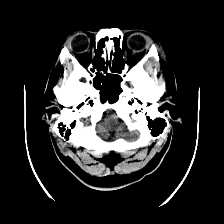

Supplement: S1 Data — (ZIP) [file pone.0295536.s002.zip › S2_Data/traindata_224/traindata/0/IM_0002-ID_f2d189b7f.png]

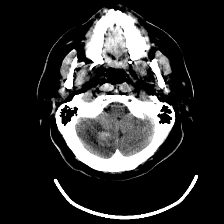

Supplement: S1 Data — (ZIP) [file pone.0295536.s002.zip › S2_Data/traindata_224/traindata/0/IM_0003-ID_093afc8d9.png]

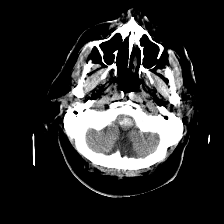

Supplement: S1 Data — (ZIP) [file pone.0295536.s002.zip › S2_Data/traindata_224/traindata/0/IM_0003-ID_0b9be23bf.png]

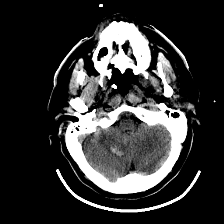

Supplement: S1 Data — (ZIP) [file pone.0295536.s002.zip › S2_Data/traindata_224/traindata/0/IM_0003-ID_1b397a5f3.png]

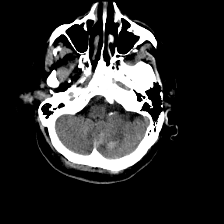

Supplement: S1 Data — (ZIP) [file pone.0295536.s002.zip › S2_Data/traindata_224/traindata/0/IM_0003-ID_1b73592a4.png]

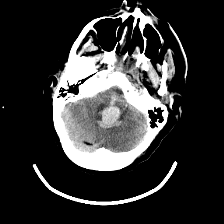

Supplement: S1 Data — (ZIP) [file pone.0295536.s002.zip › S2_Data/traindata_224/traindata/0/IM_0003-ID_31cda61a0.png]

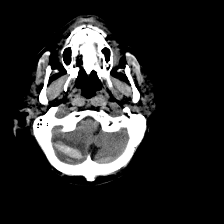

Supplement: S1 Data — (ZIP) [file pone.0295536.s002.zip › S2_Data/traindata_224/traindata/0/IM_0003-ID_6459d6004.png]

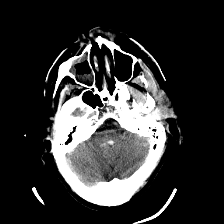

Supplement: S1 Data — (ZIP) [file pone.0295536.s002.zip › S2_Data/traindata_224/traindata/0/IM_0003-ID_68f6fac67.png]

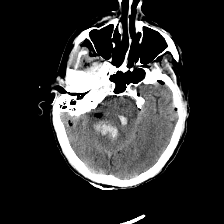

Supplement: S1 Data — (ZIP) [file pone.0295536.s002.zip › S2_Data/traindata_224/traindata/0/IM_0003-ID_72df67677.png]

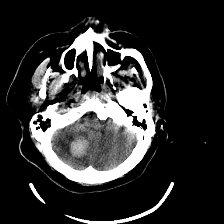

Supplement: S1 Data — (ZIP) [file pone.0295536.s002.zip › S2_Data/traindata_224/traindata/0/IM_0003-ID_7425a7f20.png]

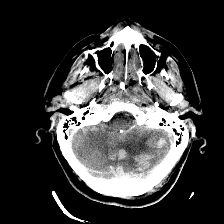

Supplement: S1 Data — (ZIP) [file pone.0295536.s002.zip › S2_Data/traindata_224/traindata/0/IM_0003-ID_84cbf705b.png]

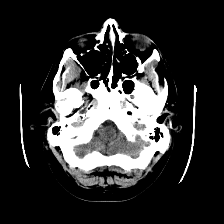

Supplement: S1 Data — (ZIP) [file pone.0295536.s002.zip › S2_Data/traindata_224/traindata/0/IM_0003-ID_9370d86f6.png]

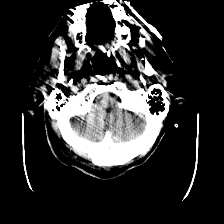

Supplement: S1 Data — (ZIP) [file pone.0295536.s002.zip › S2_Data/traindata_224/traindata/0/IM_0003-ID_9738c055d.png]

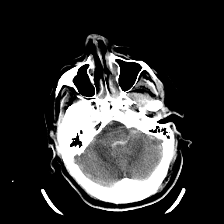

Supplement: S1 Data — (ZIP) [file pone.0295536.s002.zip › S2_Data/traindata_224/traindata/0/IM_0003-ID_a01c96213.png]

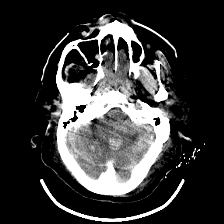

Supplement: S1 Data — (ZIP) [file pone.0295536.s002.zip › S2_Data/traindata_224/traindata/0/IM_0003-ID_a2845b9aa.png]

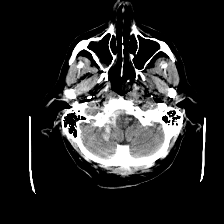

Supplement: S1 Data — (ZIP) [file pone.0295536.s002.zip › S2_Data/traindata_224/traindata/0/IM_0003-ID_b569710c4.png]

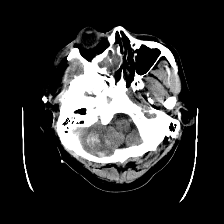

Supplement: S1 Data — (ZIP) [file pone.0295536.s002.zip › S2_Data/traindata_224/traindata/0/IM_0003-ID_d6a3dd87a.png]

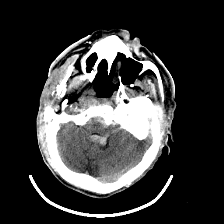

Supplement: S1 Data — (ZIP) [file pone.0295536.s002.zip › S2_Data/traindata_224/traindata/0/IM_0003-ID_db013d767.png]

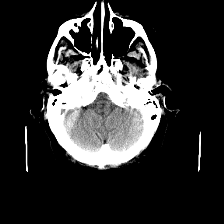

Supplement: S1 Data — (ZIP) [file pone.0295536.s002.zip › S2_Data/traindata_224/traindata/0/IM_0003-ID_e16af557c.png]

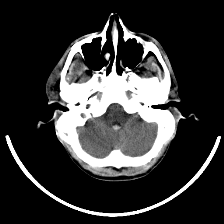

Supplement: S1 Data — (ZIP) [file pone.0295536.s002.zip › S2_Data/traindata_224/traindata/0/IM_0004-ID_039c562c4.png]

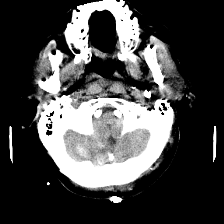

Supplement: S1 Data — (ZIP) [file pone.0295536.s002.zip › S2_Data/traindata_224/traindata/0/IM_0004-ID_08b378514.png]

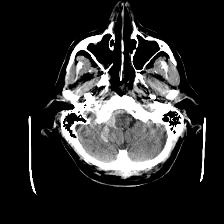

Supplement: S1 Data — (ZIP) [file pone.0295536.s002.zip › S2_Data/traindata_224/traindata/0/IM_0004-ID_0c4647359.png]

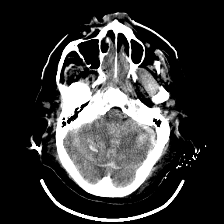

Supplement: S1 Data — (ZIP) [file pone.0295536.s002.zip › S2_Data/traindata_224/traindata/0/IM_0004-ID_2523297e5.png]

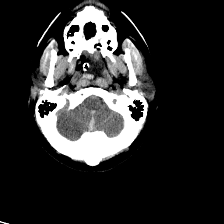

Supplement: S1 Data — (ZIP) [file pone.0295536.s002.zip › S2_Data/traindata_224/traindata/0/IM_0004-ID_28c444c9c.png]

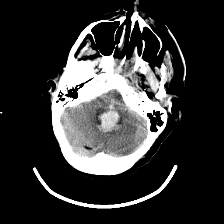

Supplement: S1 Data — (ZIP) [file pone.0295536.s002.zip › S2_Data/traindata_224/traindata/0/IM_0004-ID_337dfa8c2.png]

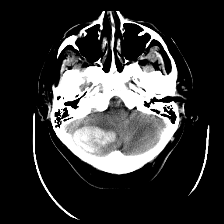

Supplement: S1 Data — (ZIP) [file pone.0295536.s002.zip › S2_Data/traindata_224/traindata/0/IM_0004-ID_3762a9d52.png]

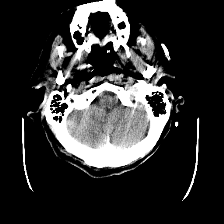

Supplement: S1 Data — (ZIP) [file pone.0295536.s002.zip › S2_Data/traindata_224/traindata/0/IM_0004-ID_3ba2759b0.png]

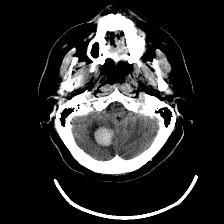

Supplement: S1 Data — (ZIP) [file pone.0295536.s002.zip › S2_Data/traindata_224/traindata/0/IM_0004-ID_3ffb00bcd.png]

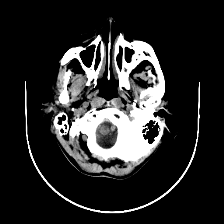

Supplement: S1 Data — (ZIP) [file pone.0295536.s002.zip › S2_Data/traindata_224/traindata/0/IM_0004-ID_411e3a4f5.png]

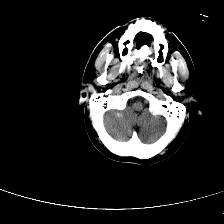

Supplement: S1 Data — (ZIP) [file pone.0295536.s002.zip › S2_Data/traindata_224/traindata/0/IM_0004-ID_460d6c7ce.png]

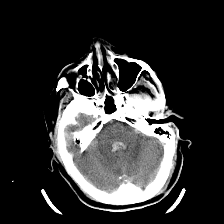

Supplement: S1 Data — (ZIP) [file pone.0295536.s002.zip › S2_Data/traindata_224/traindata/0/IM_0004-ID_4ae18ff3e.png]

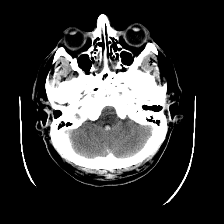

Supplement: S1 Data — (ZIP) [file pone.0295536.s002.zip › S2_Data/traindata_224/traindata/0/IM_0004-ID_4f349b39a.png]

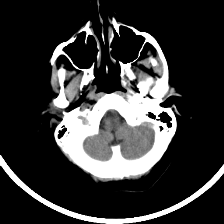

Supplement: S1 Data — (ZIP) [file pone.0295536.s002.zip › S2_Data/traindata_224/traindata/0/IM_0004-ID_518d6662e.png]

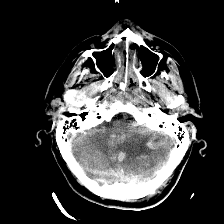

Supplement: S1 Data — (ZIP) [file pone.0295536.s002.zip › S2_Data/traindata_224/traindata/0/IM_0004-ID_52fcdbd14.png]

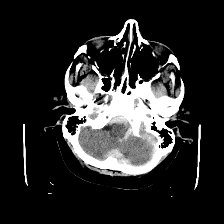

Supplement: S1 Data — (ZIP) [file pone.0295536.s002.zip › S2_Data/traindata_224/traindata/0/IM_0004-ID_566c4978e.png]

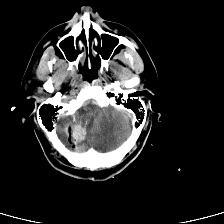

Supplement: S1 Data — (ZIP) [file pone.0295536.s002.zip › S2_Data/traindata_224/traindata/0/IM_0004-ID_6debc00bd.png]

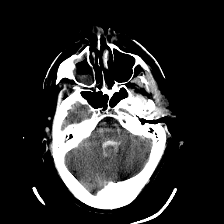

Supplement: S1 Data — (ZIP) [file pone.0295536.s002.zip › S2_Data/traindata_224/traindata/0/IM_0004-ID_70aaf1bf4.png]

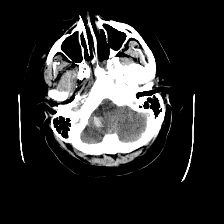

Supplement: S1 Data — (ZIP) [file pone.0295536.s002.zip › S2_Data/traindata_224/traindata/0/IM_0004-ID_72720e428.png]

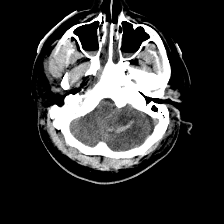

Supplement: S1 Data — (ZIP) [file pone.0295536.s002.zip › S2_Data/traindata_224/traindata/0/IM_0004-ID_72e306345.png]

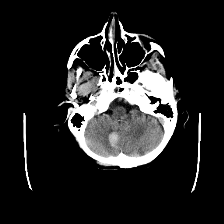

Supplement: S1 Data — (ZIP) [file pone.0295536.s002.zip › S2_Data/traindata_224/traindata/0/IM_0004-ID_77d6a8a29.png]

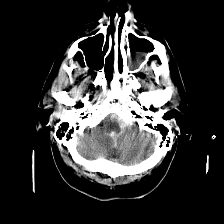

Supplement: S1 Data — (ZIP) [file pone.0295536.s002.zip › S2_Data/traindata_224/traindata/0/IM_0004-ID_7c1145ff9.png]

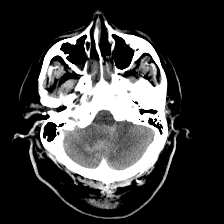

Supplement: S1 Data — (ZIP) [file pone.0295536.s002.zip › S2_Data/traindata_224/traindata/0/IM_0004-ID_7d1fd19a3.png]

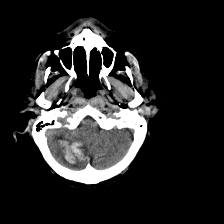

Supplement: S1 Data — (ZIP) [file pone.0295536.s002.zip › S2_Data/traindata_224/traindata/0/IM_0004-ID_7d6878ba0.png]

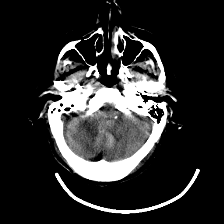

Supplement: S1 Data — (ZIP) [file pone.0295536.s002.zip › S2_Data/traindata_224/traindata/0/IM_0004-ID_854dda944.png]

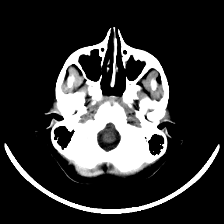

Supplement: S1 Data — (ZIP) [file pone.0295536.s002.zip › S2_Data/traindata_224/traindata/0/IM_0004-ID_9b8fd60ca.png]

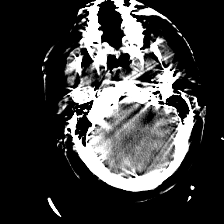

Supplement: S1 Data — (ZIP) [file pone.0295536.s002.zip › S2_Data/traindata_224/traindata/0/IM_0004-ID_9e7a9f92d.png]

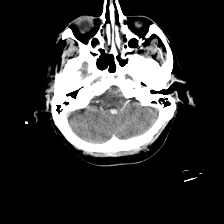

Supplement: S1 Data — (ZIP) [file pone.0295536.s002.zip › S2_Data/traindata_224/traindata/0/IM_0004-ID_a1df019d1.png]

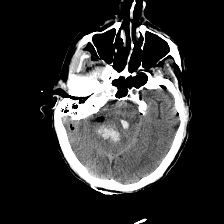

Supplement: S1 Data — (ZIP) [file pone.0295536.s002.zip › S2_Data/traindata_224/traindata/0/IM_0004-ID_a5973ef74.png]

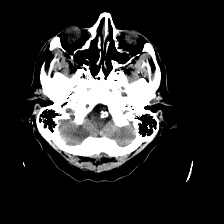

Supplement: S1 Data — (ZIP) [file pone.0295536.s002.zip › S2_Data/traindata_224/traindata/0/IM_0004-ID_aaa0701eb.png]

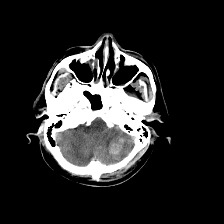

Supplement: S1 Data — (ZIP) [file pone.0295536.s002.zip › S2_Data/traindata_224/traindata/0/IM_0004-ID_af90ee0a9.png]

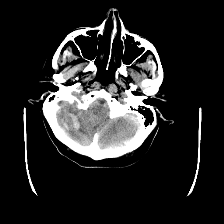

Supplement: S1 Data — (ZIP) [file pone.0295536.s002.zip › S2_Data/traindata_224/traindata/0/IM_0004-ID_b4386c146.png]

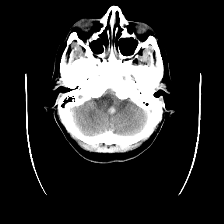

Supplement: S1 Data — (ZIP) [file pone.0295536.s002.zip › S2_Data/traindata_224/traindata/0/IM_0004-ID_b530a9807.png]

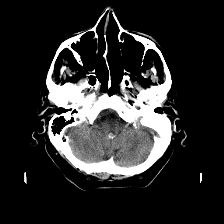

Supplement: S1 Data — (ZIP) [file pone.0295536.s002.zip › S2_Data/traindata_224/traindata/0/IM_0004-ID_b94d2ef15.png]

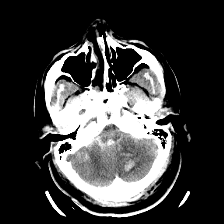

Supplement: S1 Data — (ZIP) [file pone.0295536.s002.zip › S2_Data/traindata_224/traindata/0/IM_0004-ID_d4def7e5d.png]

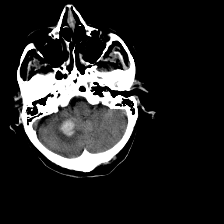

Supplement: S1 Data — (ZIP) [file pone.0295536.s002.zip › S2_Data/traindata_224/traindata/0/IM_0004-ID_d942ab733.png]

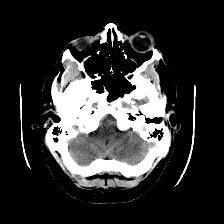

Supplement: S1 Data — (ZIP) [file pone.0295536.s002.zip › S2_Data/traindata_224/traindata/0/IM_0004-ID_e05d873ac.png]

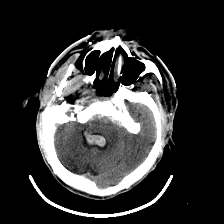

Supplement: S1 Data — (ZIP) [file pone.0295536.s002.zip › S2_Data/traindata_224/traindata/0/IM_0004-ID_ede968118.png]

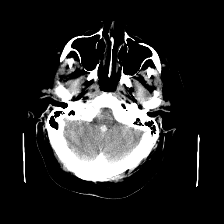

Supplement: S1 Data — (ZIP) [file pone.0295536.s002.zip › S2_Data/traindata_224/traindata/0/IM_0004-ID_f75f50ed1.png]

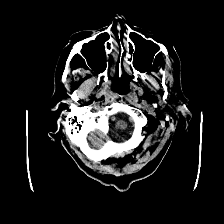

Supplement: S1 Data — (ZIP) [file pone.0295536.s002.zip › S2_Data/traindata_224/traindata/0/IM_0004-ID_fb9df8b9e.png]

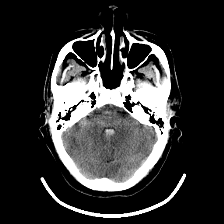

Supplement: S1 Data — (ZIP) [file pone.0295536.s002.zip › S2_Data/traindata_224/traindata/0/IM_0005-ID_00c000a81.png]

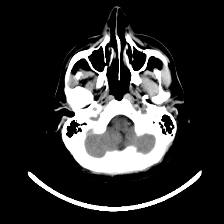

Supplement: S1 Data — (ZIP) [file pone.0295536.s002.zip › S2_Data/traindata_224/traindata/0/IM_0005-ID_097a98371.png]

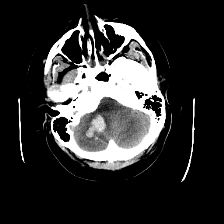

Supplement: S1 Data — (ZIP) [file pone.0295536.s002.zip › S2_Data/traindata_224/traindata/0/IM_0005-ID_0c957218a.png]

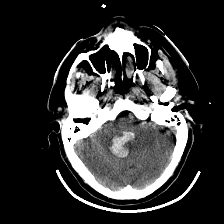

Supplement: S1 Data — (ZIP) [file pone.0295536.s002.zip › S2_Data/traindata_224/traindata/0/IM_0005-ID_15196fcfa.png]

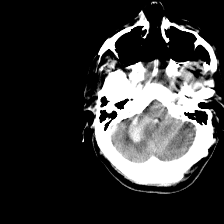

Supplement: S1 Data — (ZIP) [file pone.0295536.s002.zip › S2_Data/traindata_224/traindata/0/IM_0005-ID_1d467c5c2.png]

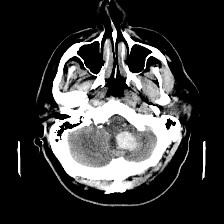

Supplement: S1 Data — (ZIP) [file pone.0295536.s002.zip › S2_Data/traindata_224/traindata/0/IM_0005-ID_1e5802dfa.png]

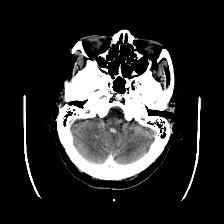

Supplement: S1 Data — (ZIP) [file pone.0295536.s002.zip › S2_Data/traindata_224/traindata/0/IM_0005-ID_246168f91.png]

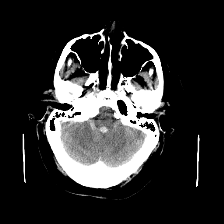

Supplement: S1 Data — (ZIP) [file pone.0295536.s002.zip › S2_Data/traindata_224/traindata/0/IM_0005-ID_2c37ed251.png]

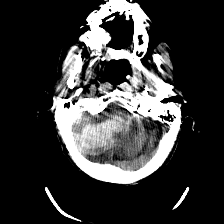

Supplement: S1 Data — (ZIP) [file pone.0295536.s002.zip › S2_Data/traindata_224/traindata/0/IM_0005-ID_2ca4d0cb1.png]
