# Supplementary figures and images for: Adaptive mask-based brain extraction method for head CT images (part 4 of 14)
Source: PLoS One. 2024 Mar 11;19(3):e0295536. doi: 10.1371/journal.pone.0295536 (PMC10927156; doi:10.1371/journal.pone.0295536)

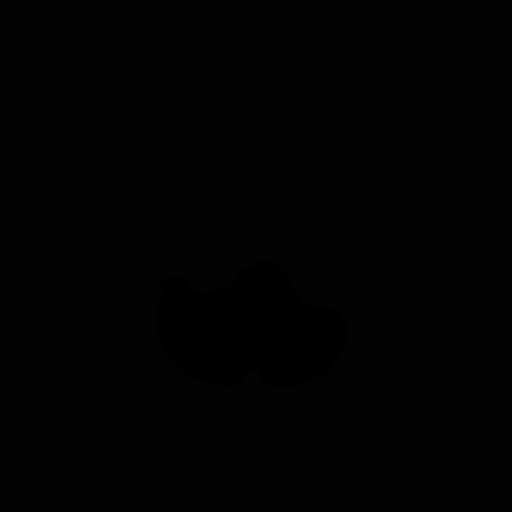

Supplement: S2 Data — (ZIP) [file pone.0295536.s003.zip › S3_Data/FCN_Training set_Label/IM_0005-ID_5a76a0838.png]

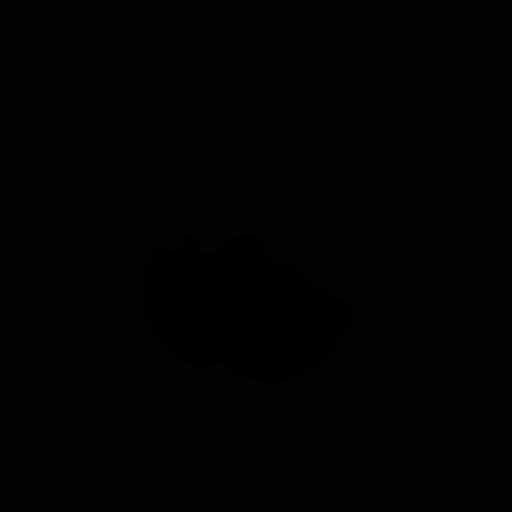

Supplement: S2 Data — (ZIP) [file pone.0295536.s003.zip › S3_Data/FCN_Training set_Label/IM_0005-ID_5bc861901.png]

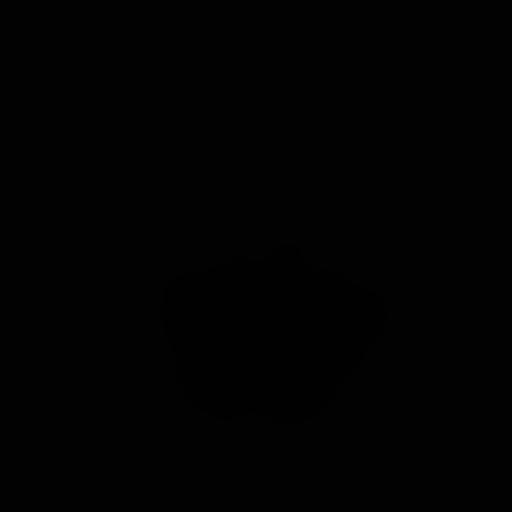

Supplement: S2 Data — (ZIP) [file pone.0295536.s003.zip › S3_Data/FCN_Training set_Label/IM_0005-ID_60072507b.png]

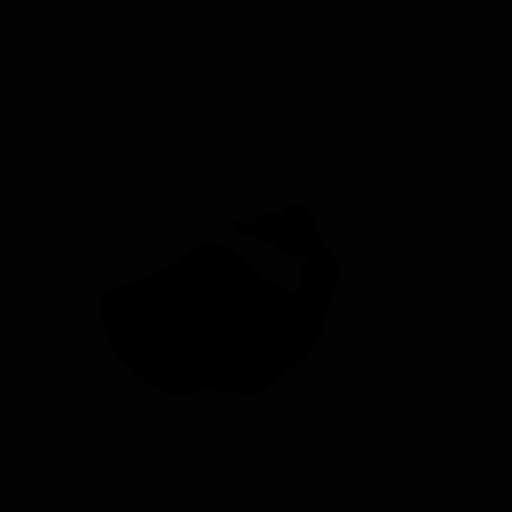

Supplement: S2 Data — (ZIP) [file pone.0295536.s003.zip › S3_Data/FCN_Training set_Label/IM_0005-ID_61c5b974b.png]

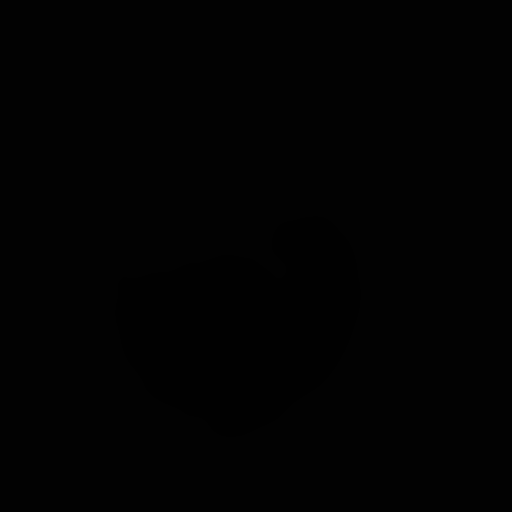

Supplement: S2 Data — (ZIP) [file pone.0295536.s003.zip › S3_Data/FCN_Training set_Label/IM_0005-ID_63ad7bffc.png]

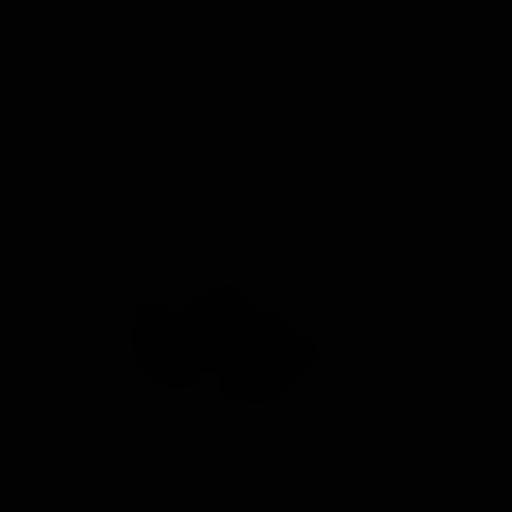

Supplement: S2 Data — (ZIP) [file pone.0295536.s003.zip › S3_Data/FCN_Training set_Label/IM_0005-ID_65470871b.png]

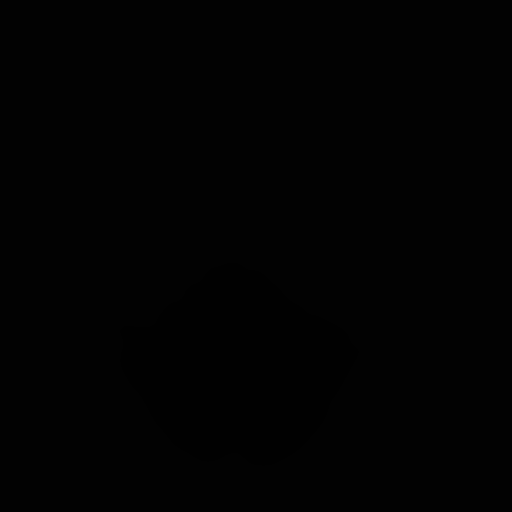

Supplement: S2 Data — (ZIP) [file pone.0295536.s003.zip › S3_Data/FCN_Training set_Label/IM_0005-ID_65d5b4c9f.png]

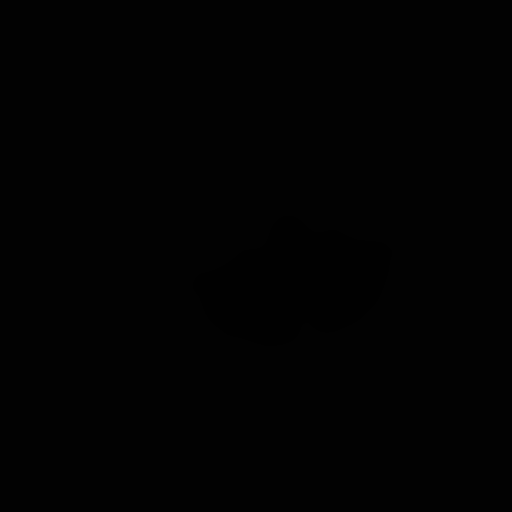

Supplement: S2 Data — (ZIP) [file pone.0295536.s003.zip › S3_Data/FCN_Training set_Label/IM_0005-ID_6859101fc.png]

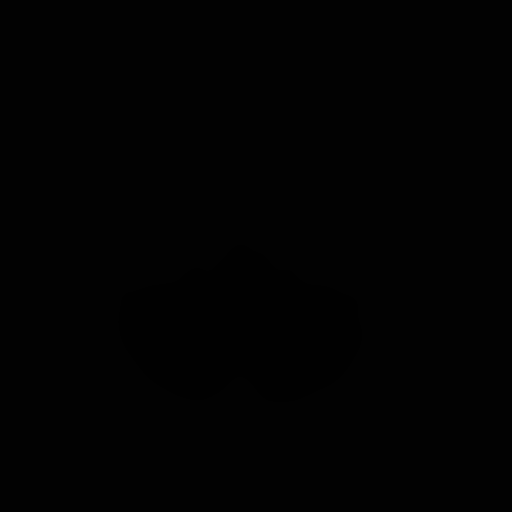

Supplement: S2 Data — (ZIP) [file pone.0295536.s003.zip › S3_Data/FCN_Training set_Label/IM_0005-ID_6b5132d81.png]

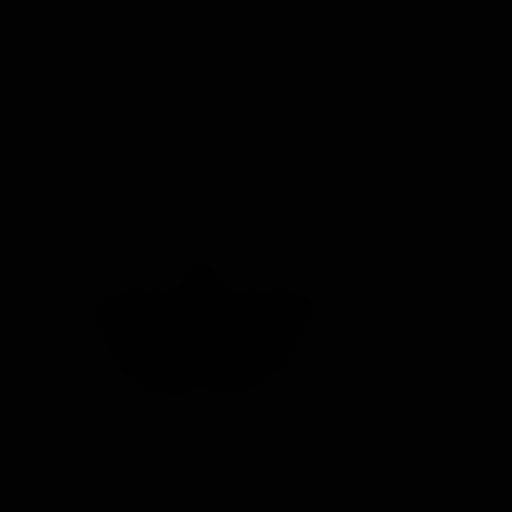

Supplement: S2 Data — (ZIP) [file pone.0295536.s003.zip › S3_Data/FCN_Training set_Label/IM_0005-ID_6db4623dd.png]

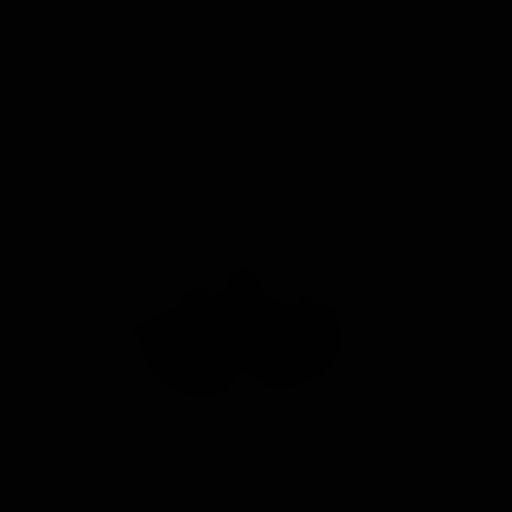

Supplement: S2 Data — (ZIP) [file pone.0295536.s003.zip › S3_Data/FCN_Training set_Label/IM_0005-ID_73b3c1c67.png]

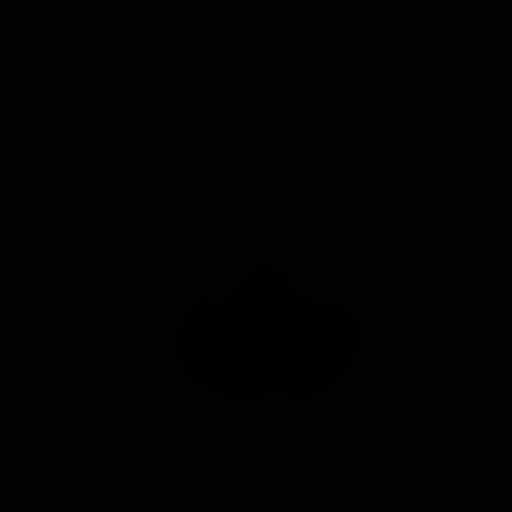

Supplement: S2 Data — (ZIP) [file pone.0295536.s003.zip › S3_Data/FCN_Training set_Label/IM_0005-ID_743cfbcb6.png]

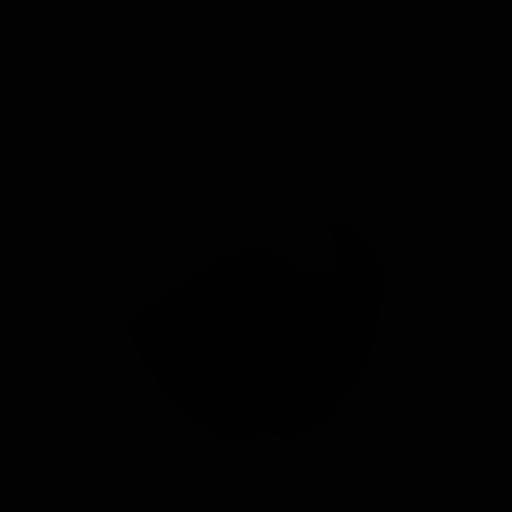

Supplement: S2 Data — (ZIP) [file pone.0295536.s003.zip › S3_Data/FCN_Training set_Label/IM_0005-ID_74690521b.png]

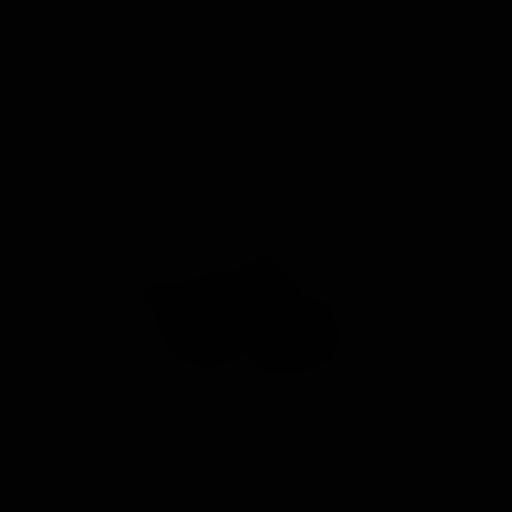

Supplement: S2 Data — (ZIP) [file pone.0295536.s003.zip › S3_Data/FCN_Training set_Label/IM_0005-ID_7544b6fc3.png]

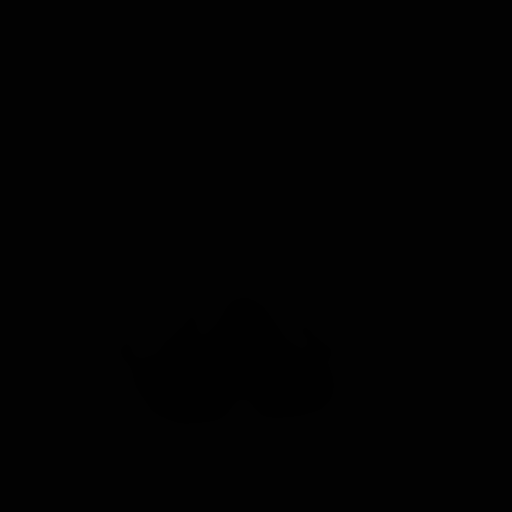

Supplement: S2 Data — (ZIP) [file pone.0295536.s003.zip › S3_Data/FCN_Training set_Label/IM_0005-ID_76127d9a0.png]

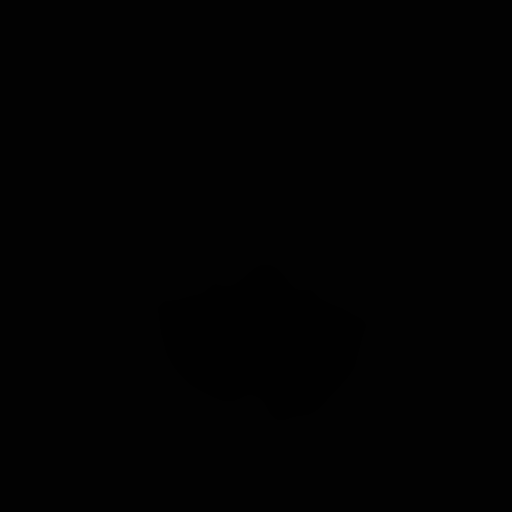

Supplement: S2 Data — (ZIP) [file pone.0295536.s003.zip › S3_Data/FCN_Training set_Label/IM_0005-ID_77a83dadf.png]

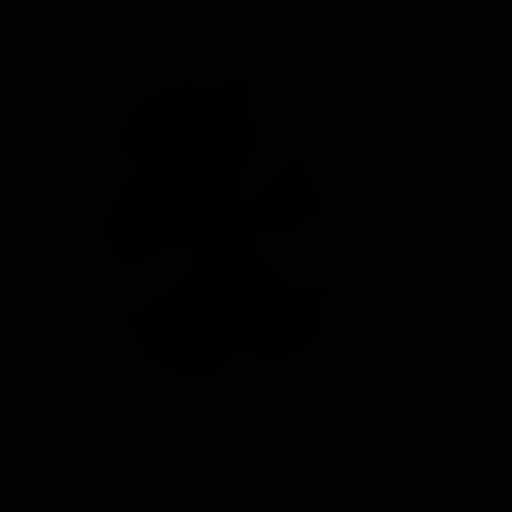

Supplement: S2 Data — (ZIP) [file pone.0295536.s003.zip › S3_Data/FCN_Training set_Label/IM_0005-ID_7a61f39f5.png]

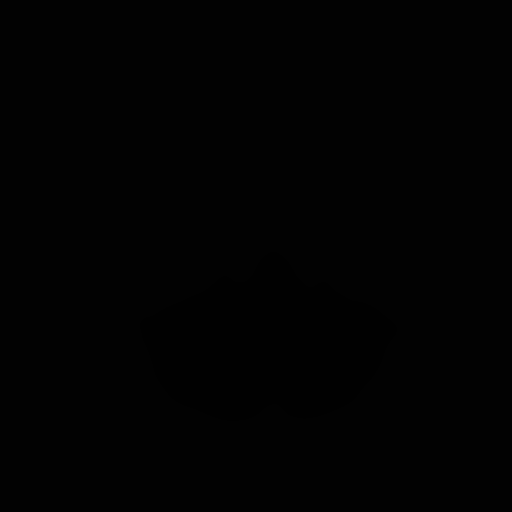

Supplement: S2 Data — (ZIP) [file pone.0295536.s003.zip › S3_Data/FCN_Training set_Label/IM_0005-ID_7c2e569a2.png]

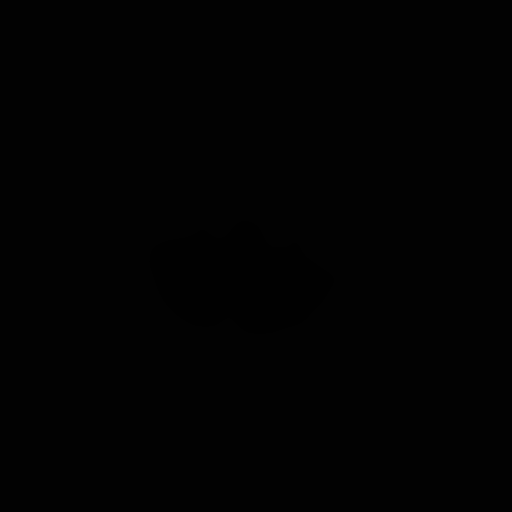

Supplement: S2 Data — (ZIP) [file pone.0295536.s003.zip › S3_Data/FCN_Training set_Label/IM_0005-ID_8cb590ee9.png]

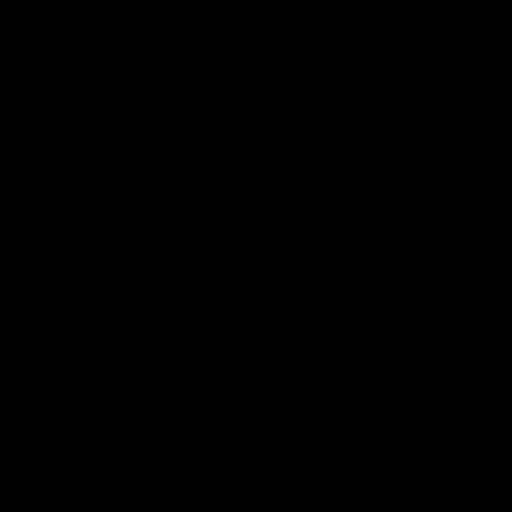

Supplement: S2 Data — (ZIP) [file pone.0295536.s003.zip › S3_Data/FCN_Training set_Label/IM_0005-ID_93a4315e4.png]

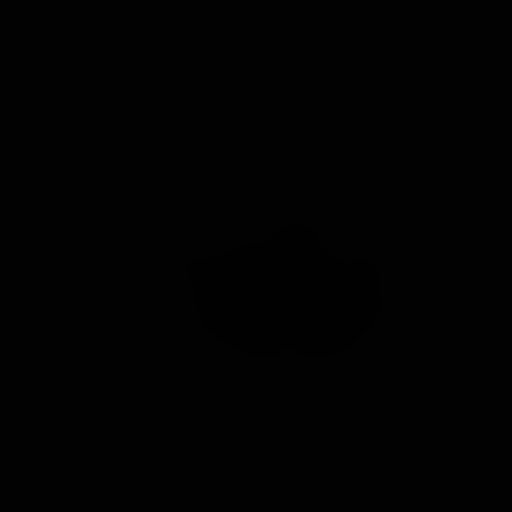

Supplement: S2 Data — (ZIP) [file pone.0295536.s003.zip › S3_Data/FCN_Training set_Label/IM_0005-ID_94b63da36.png]

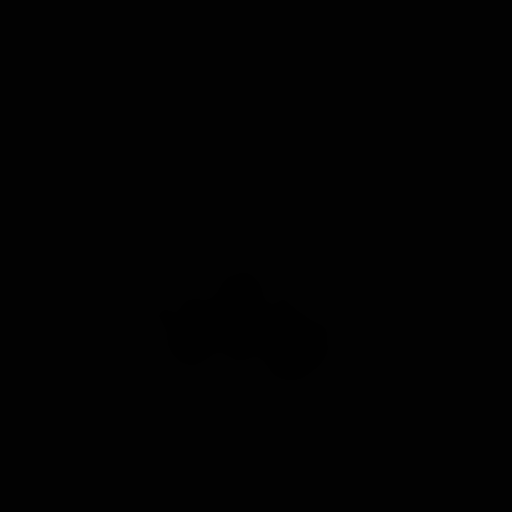

Supplement: S2 Data — (ZIP) [file pone.0295536.s003.zip › S3_Data/FCN_Training set_Label/IM_0005-ID_99f7552d8.png]

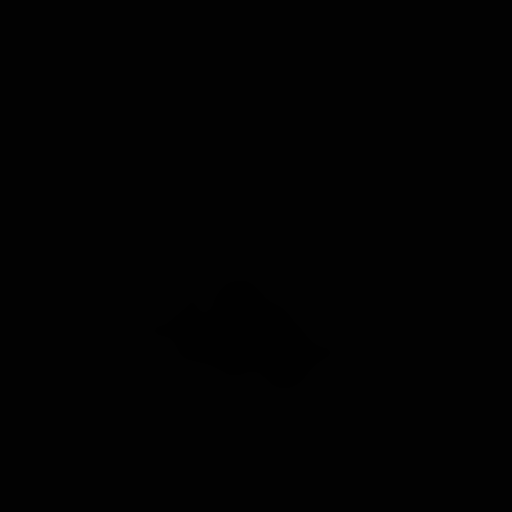

Supplement: S2 Data — (ZIP) [file pone.0295536.s003.zip › S3_Data/FCN_Training set_Label/IM_0005-ID_9e97be164.png]

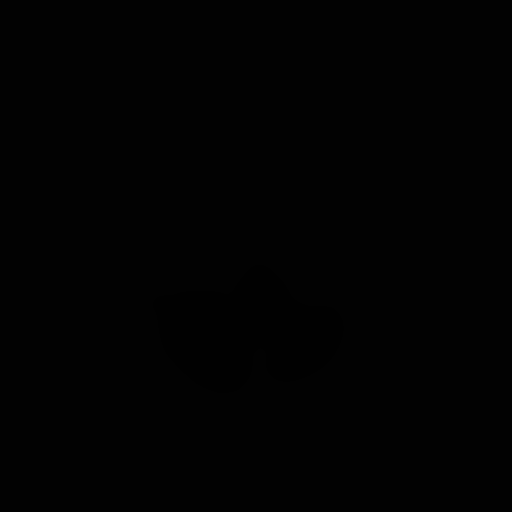

Supplement: S2 Data — (ZIP) [file pone.0295536.s003.zip › S3_Data/FCN_Training set_Label/IM_0005-ID_a67fb2291.png]

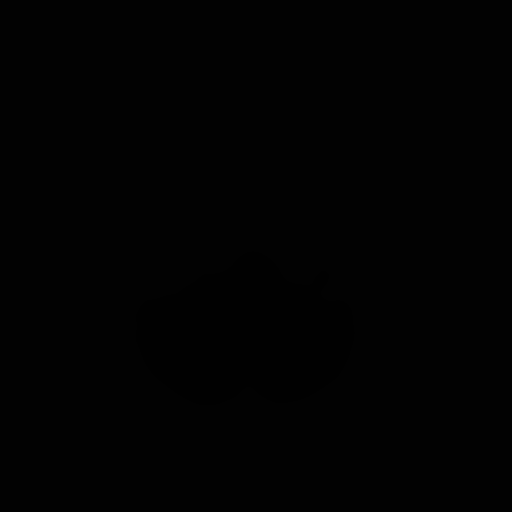

Supplement: S2 Data — (ZIP) [file pone.0295536.s003.zip › S3_Data/FCN_Training set_Label/IM_0005-ID_aeb68590d.png]

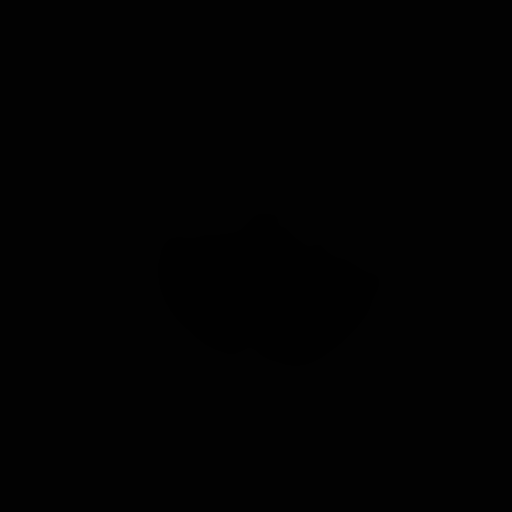

Supplement: S2 Data — (ZIP) [file pone.0295536.s003.zip › S3_Data/FCN_Training set_Label/IM_0005-ID_bc68fe186.png]

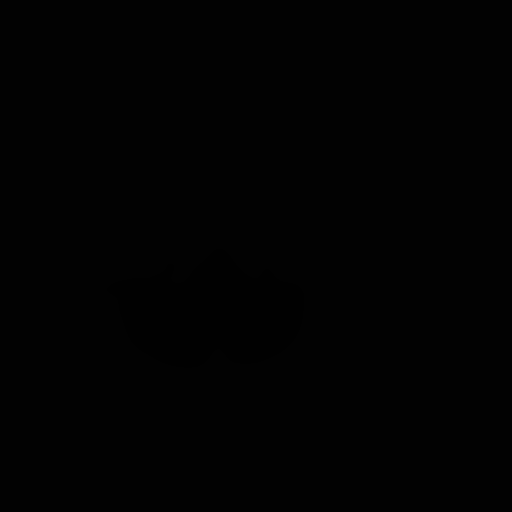

Supplement: S2 Data — (ZIP) [file pone.0295536.s003.zip › S3_Data/FCN_Training set_Label/IM_0005-ID_bdaa860d4.png]

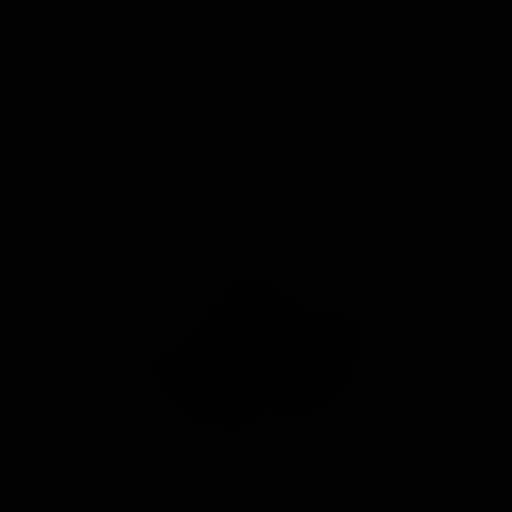

Supplement: S2 Data — (ZIP) [file pone.0295536.s003.zip › S3_Data/FCN_Training set_Label/IM_0005-ID_c5cdade23.png]

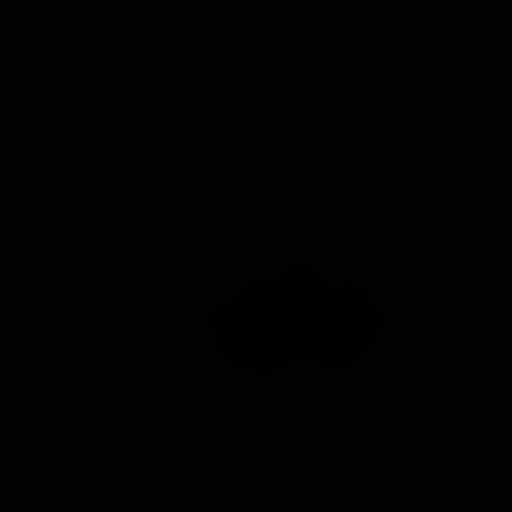

Supplement: S2 Data — (ZIP) [file pone.0295536.s003.zip › S3_Data/FCN_Training set_Label/IM_0005-ID_d05cecb80.png]

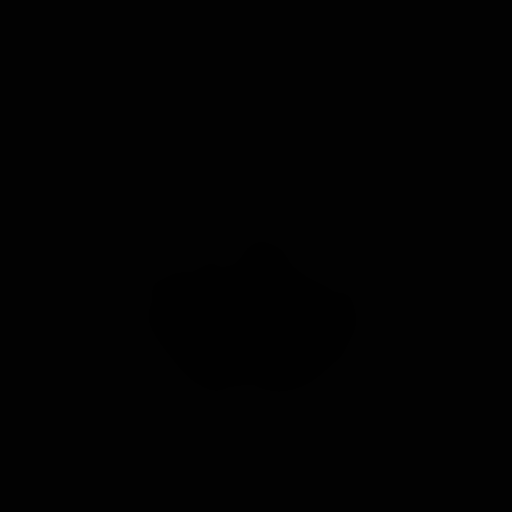

Supplement: S2 Data — (ZIP) [file pone.0295536.s003.zip › S3_Data/FCN_Training set_Label/IM_0005-ID_d13bd523a.png]

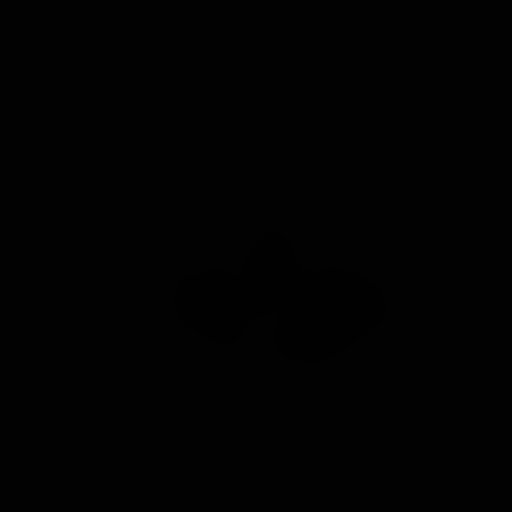

Supplement: S2 Data — (ZIP) [file pone.0295536.s003.zip › S3_Data/FCN_Training set_Label/IM_0005-ID_e1c7bfbd5.png]

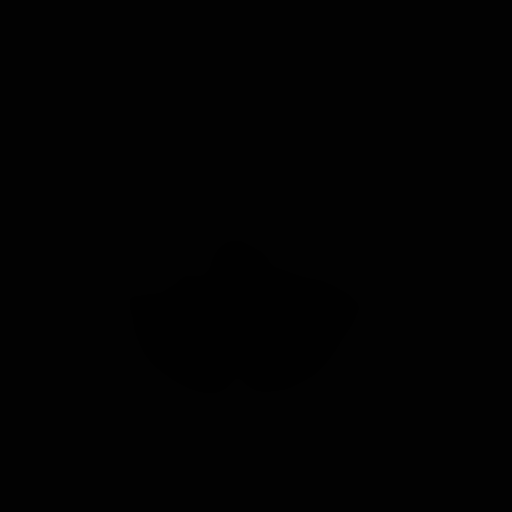

Supplement: S2 Data — (ZIP) [file pone.0295536.s003.zip › S3_Data/FCN_Training set_Label/IM_0005-ID_e2b33235e.png]

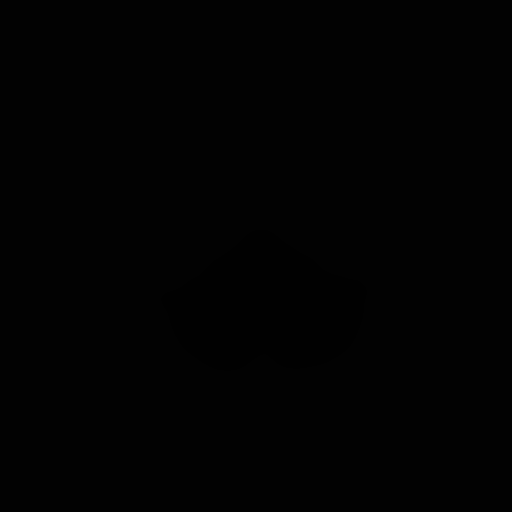

Supplement: S2 Data — (ZIP) [file pone.0295536.s003.zip › S3_Data/FCN_Training set_Label/IM_0005-ID_e40a3c4df.png]

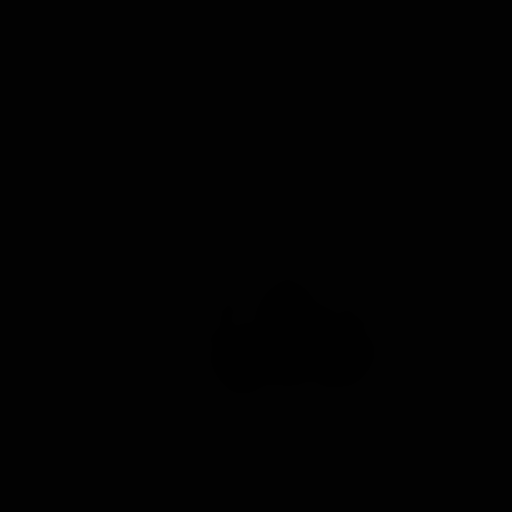

Supplement: S2 Data — (ZIP) [file pone.0295536.s003.zip › S3_Data/FCN_Training set_Label/IM_0005-ID_e6dbf0b5f.png]

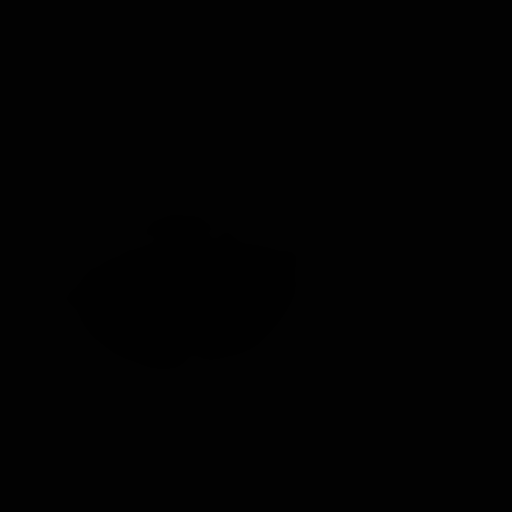

Supplement: S2 Data — (ZIP) [file pone.0295536.s003.zip › S3_Data/FCN_Training set_Label/IM_0005-ID_e77c1ba67.png]

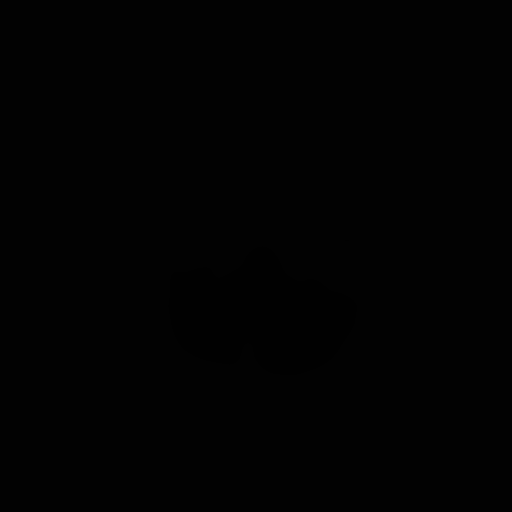

Supplement: S2 Data — (ZIP) [file pone.0295536.s003.zip › S3_Data/FCN_Training set_Label/IM_0005-ID_ed309646d.png]

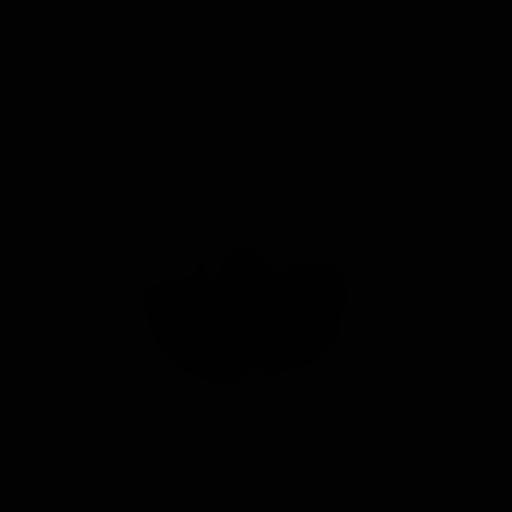

Supplement: S2 Data — (ZIP) [file pone.0295536.s003.zip › S3_Data/FCN_Training set_Label/IM_0005-ID_f03fbb264.png]

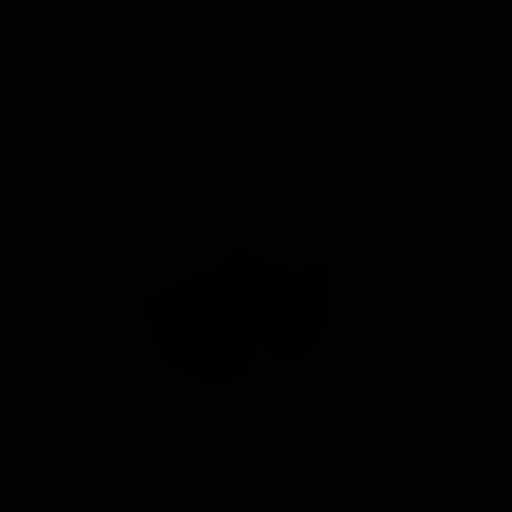

Supplement: S2 Data — (ZIP) [file pone.0295536.s003.zip › S3_Data/FCN_Training set_Label/IM_0005-ID_f592bd95b.png]

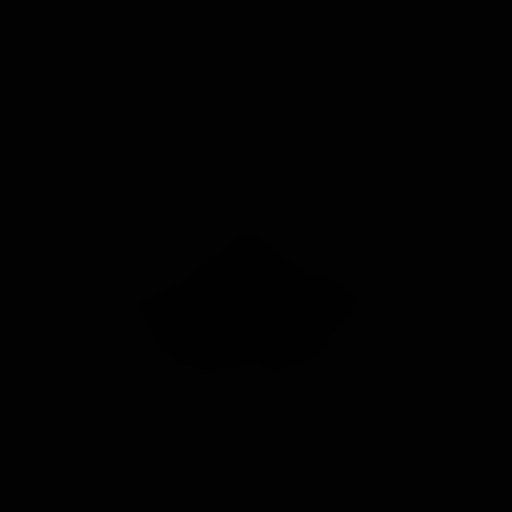

Supplement: S2 Data — (ZIP) [file pone.0295536.s003.zip › S3_Data/FCN_Training set_Label/IM_0005-ID_f92223ae1.png]

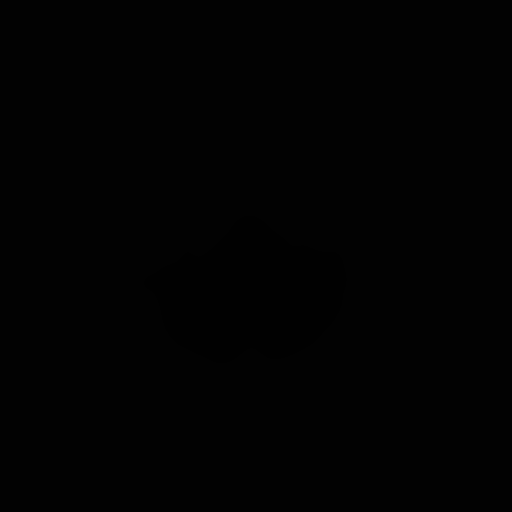

Supplement: S2 Data — (ZIP) [file pone.0295536.s003.zip › S3_Data/FCN_Training set_Label/IM_0005-ID_f9fc621c6.png]

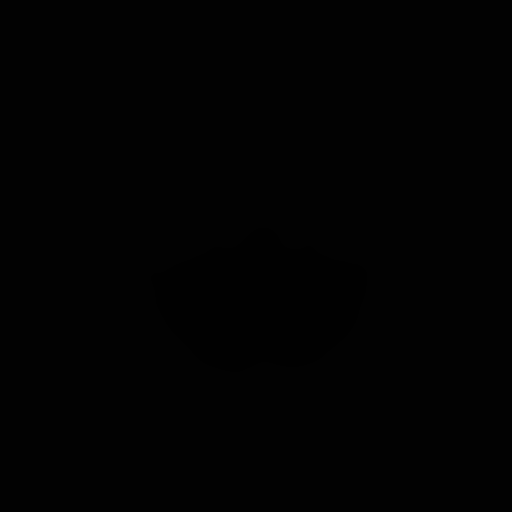

Supplement: S2 Data — (ZIP) [file pone.0295536.s003.zip › S3_Data/FCN_Training set_Label/IM_0005-ID_fe402e6b2.png]

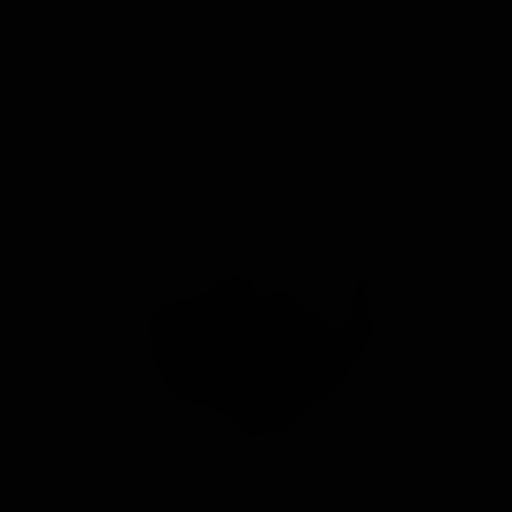

Supplement: S2 Data — (ZIP) [file pone.0295536.s003.zip › S3_Data/FCN_Training set_Label/IM_0005-ID_fed2cbae0.png]

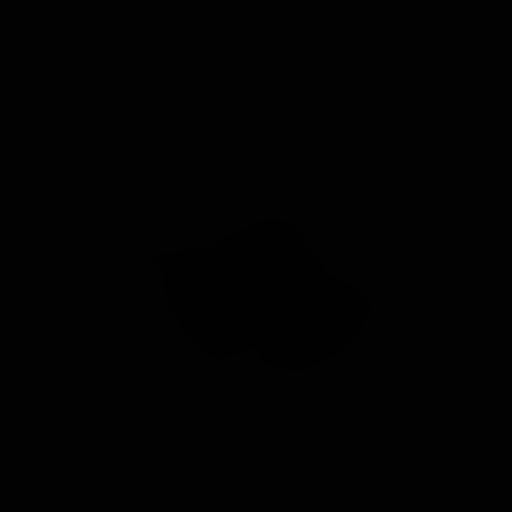

Supplement: S2 Data — (ZIP) [file pone.0295536.s003.zip › S3_Data/FCN_Training set_Label/IM_0006-ID_002251d48.png]

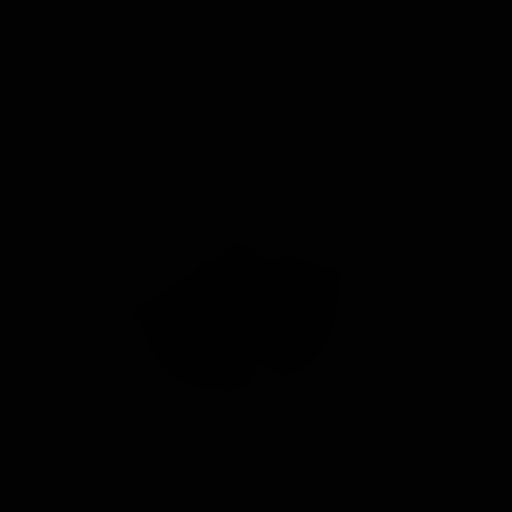

Supplement: S2 Data — (ZIP) [file pone.0295536.s003.zip › S3_Data/FCN_Training set_Label/IM_0006-ID_036b608a9.png]

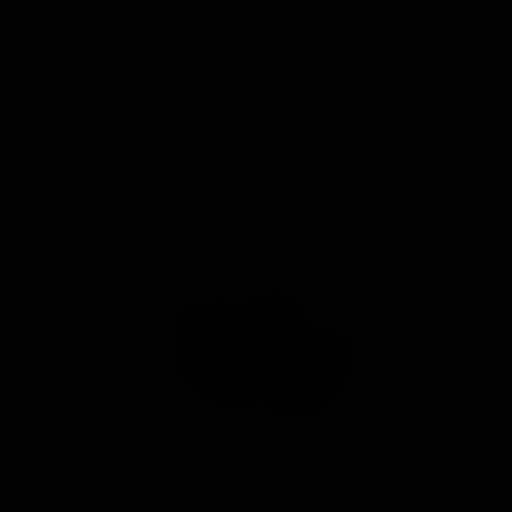

Supplement: S2 Data — (ZIP) [file pone.0295536.s003.zip › S3_Data/FCN_Training set_Label/IM_0006-ID_05635c84d.png]

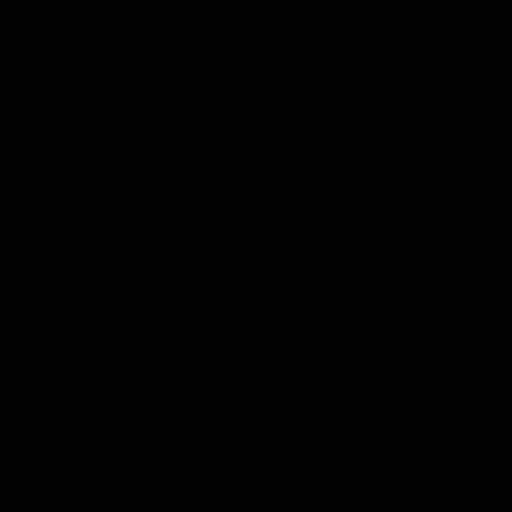

Supplement: S2 Data — (ZIP) [file pone.0295536.s003.zip › S3_Data/FCN_Training set_Label/IM_0006-ID_05e64e792.png]

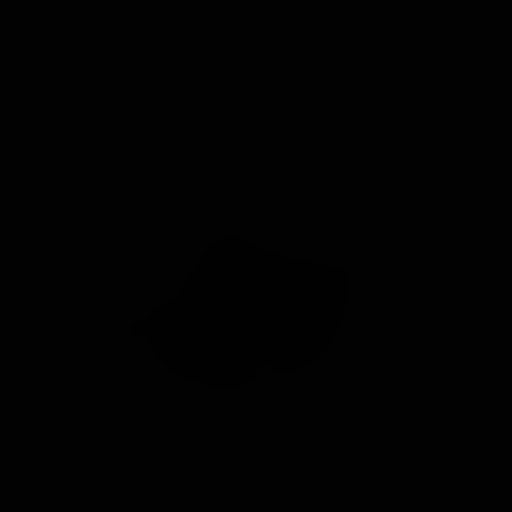

Supplement: S2 Data — (ZIP) [file pone.0295536.s003.zip › S3_Data/FCN_Training set_Label/IM_0006-ID_0b285b9ca.png]

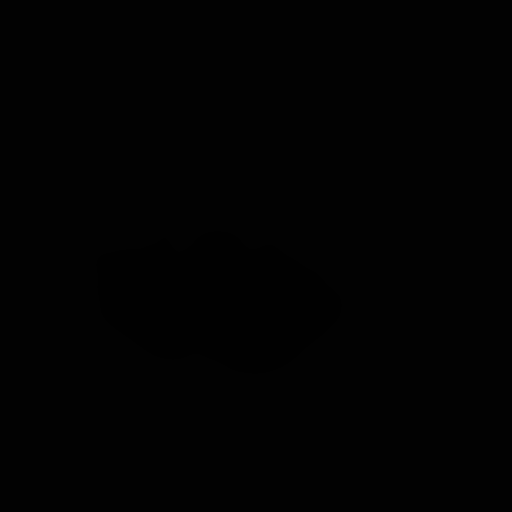

Supplement: S2 Data — (ZIP) [file pone.0295536.s003.zip › S3_Data/FCN_Training set_Label/IM_0006-ID_0ee040c60.png]

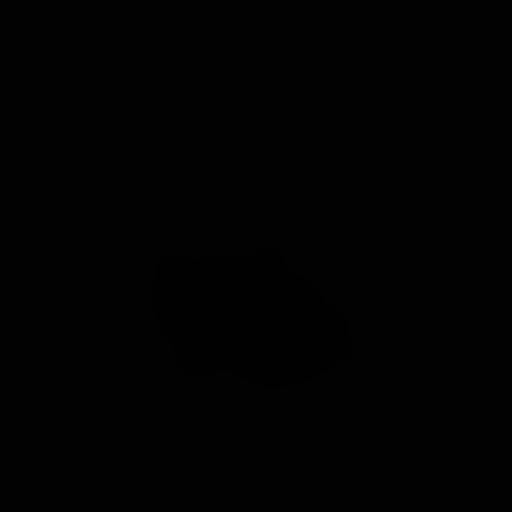

Supplement: S2 Data — (ZIP) [file pone.0295536.s003.zip › S3_Data/FCN_Training set_Label/IM_0006-ID_1036b45a8.png]

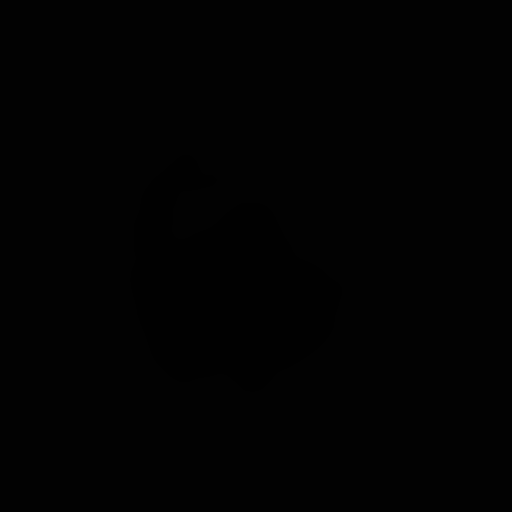

Supplement: S2 Data — (ZIP) [file pone.0295536.s003.zip › S3_Data/FCN_Training set_Label/IM_0006-ID_10465dafc.png]

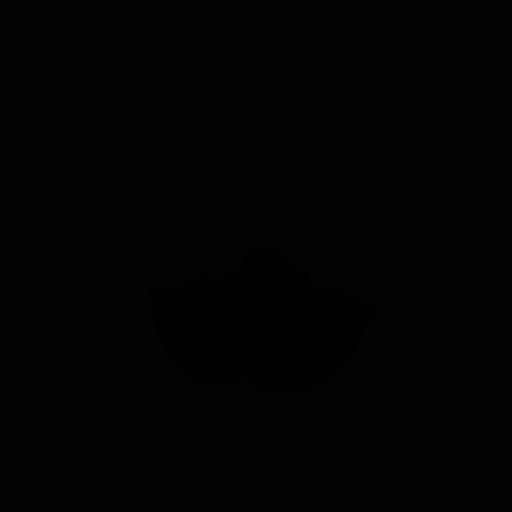

Supplement: S2 Data — (ZIP) [file pone.0295536.s003.zip › S3_Data/FCN_Training set_Label/IM_0006-ID_10dd49a55.png]

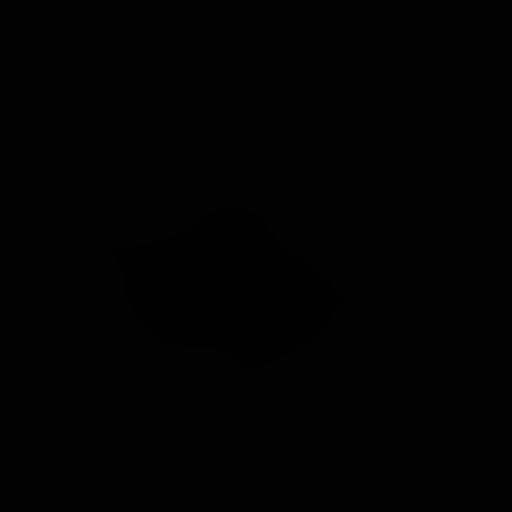

Supplement: S2 Data — (ZIP) [file pone.0295536.s003.zip › S3_Data/FCN_Training set_Label/IM_0006-ID_11e6ad569.png]

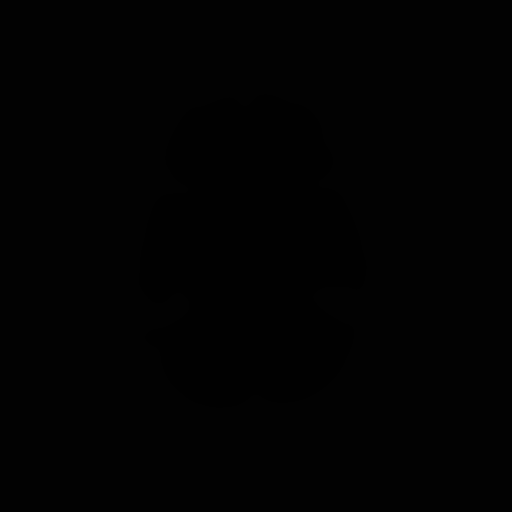

Supplement: S2 Data — (ZIP) [file pone.0295536.s003.zip › S3_Data/FCN_Training set_Label/IM_0006-ID_11f816fca.png]

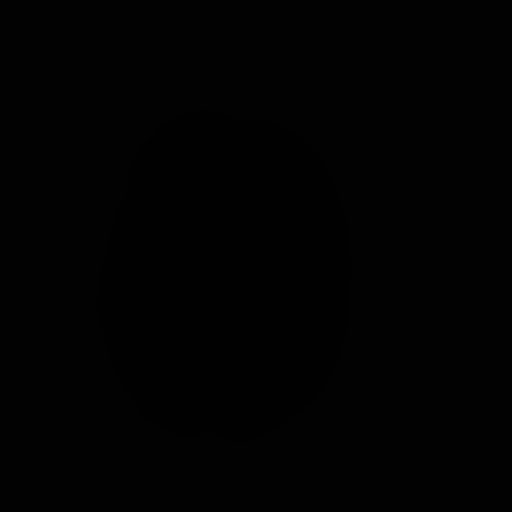

Supplement: S2 Data — (ZIP) [file pone.0295536.s003.zip › S3_Data/FCN_Training set_Label/IM_0006-ID_13df9a544.png]

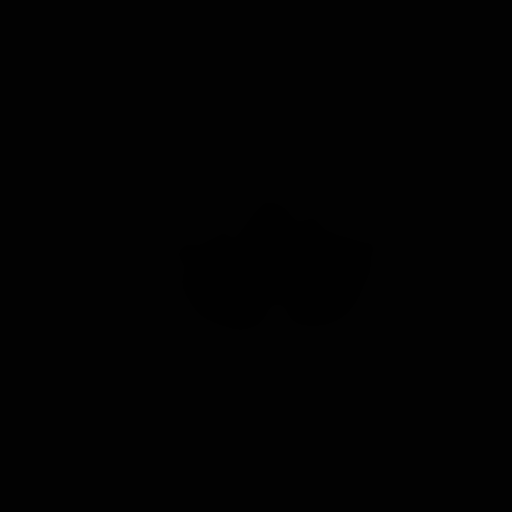

Supplement: S2 Data — (ZIP) [file pone.0295536.s003.zip › S3_Data/FCN_Training set_Label/IM_0006-ID_1aa28e21d.png]

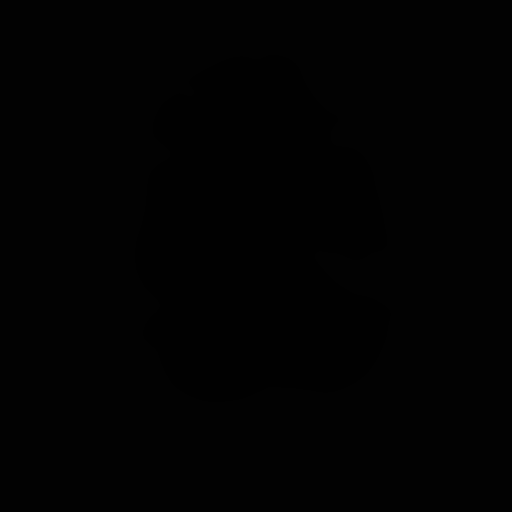

Supplement: S2 Data — (ZIP) [file pone.0295536.s003.zip › S3_Data/FCN_Training set_Label/IM_0006-ID_1aadb0431.png]

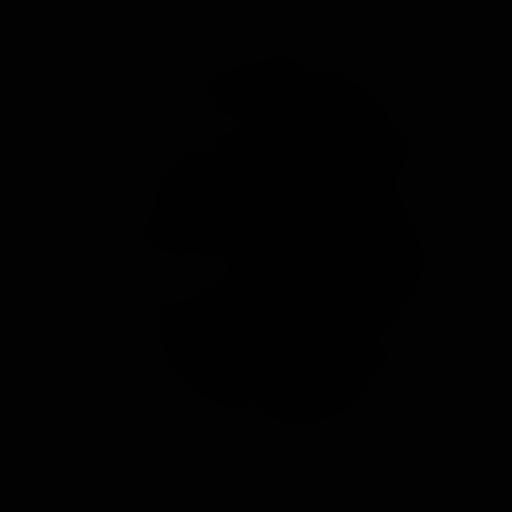

Supplement: S2 Data — (ZIP) [file pone.0295536.s003.zip › S3_Data/FCN_Training set_Label/IM_0006-ID_202da93be.png]

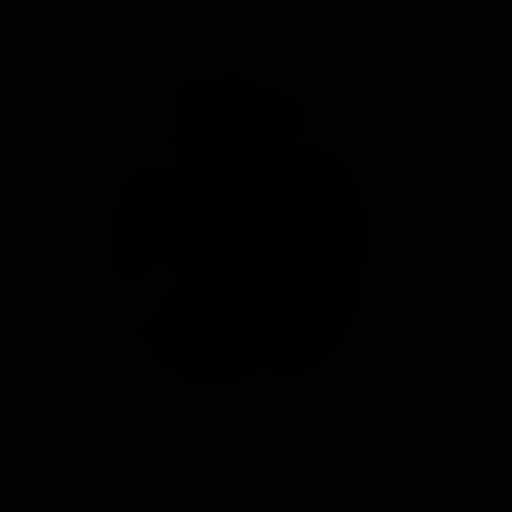

Supplement: S2 Data — (ZIP) [file pone.0295536.s003.zip › S3_Data/FCN_Training set_Label/IM_0006-ID_221a2cd9b.png]

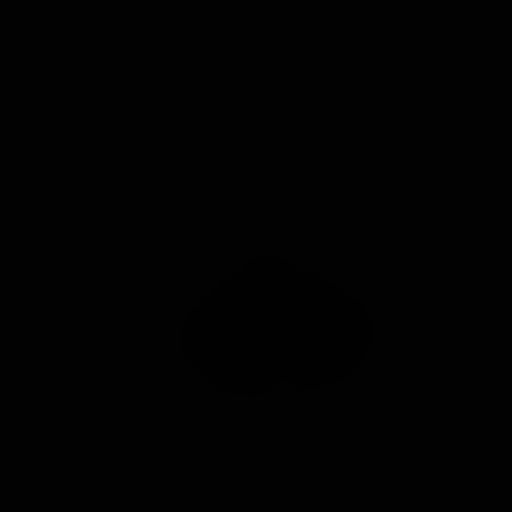

Supplement: S2 Data — (ZIP) [file pone.0295536.s003.zip › S3_Data/FCN_Training set_Label/IM_0006-ID_223ade9af.png]

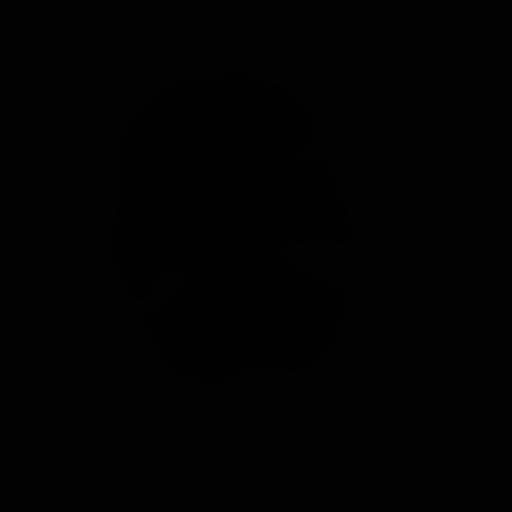

Supplement: S2 Data — (ZIP) [file pone.0295536.s003.zip › S3_Data/FCN_Training set_Label/IM_0006-ID_26ae1841e.png]

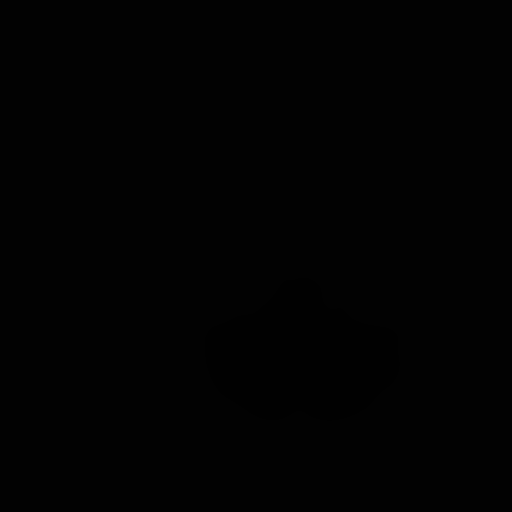

Supplement: S2 Data — (ZIP) [file pone.0295536.s003.zip › S3_Data/FCN_Training set_Label/IM_0006-ID_27d718787.png]

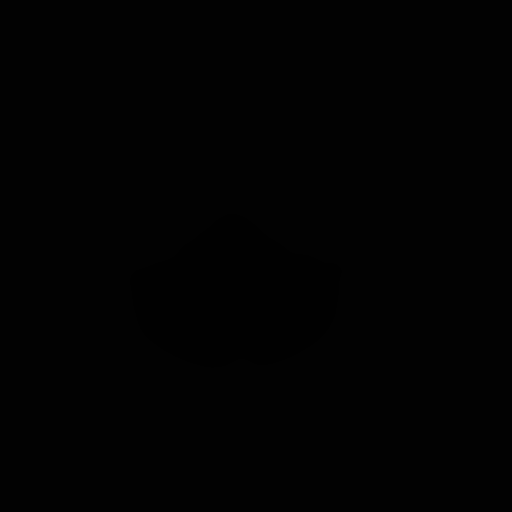

Supplement: S2 Data — (ZIP) [file pone.0295536.s003.zip › S3_Data/FCN_Training set_Label/IM_0006-ID_2aa41ca57.png]

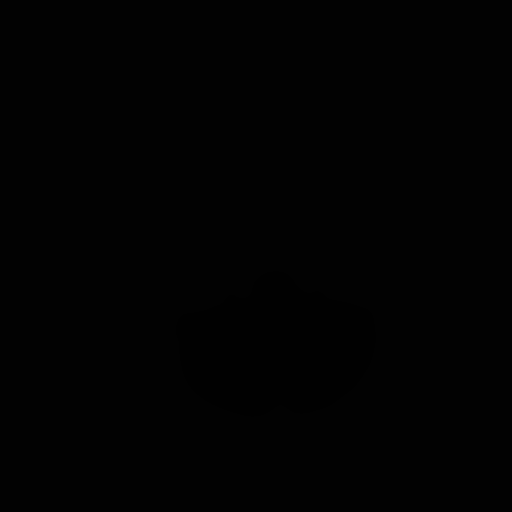

Supplement: S2 Data — (ZIP) [file pone.0295536.s003.zip › S3_Data/FCN_Training set_Label/IM_0006-ID_2e266f901.png]

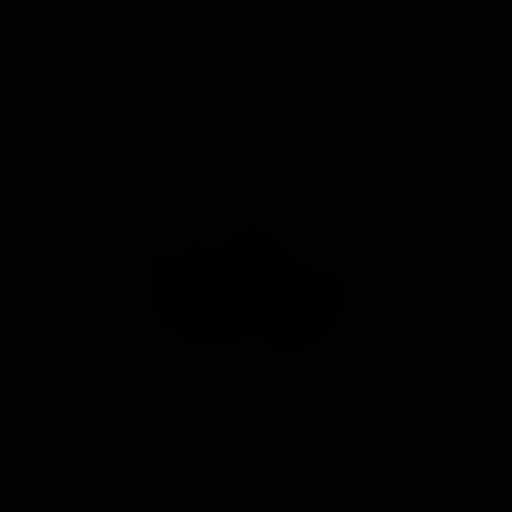

Supplement: S2 Data — (ZIP) [file pone.0295536.s003.zip › S3_Data/FCN_Training set_Label/IM_0006-ID_2f4245b48.png]

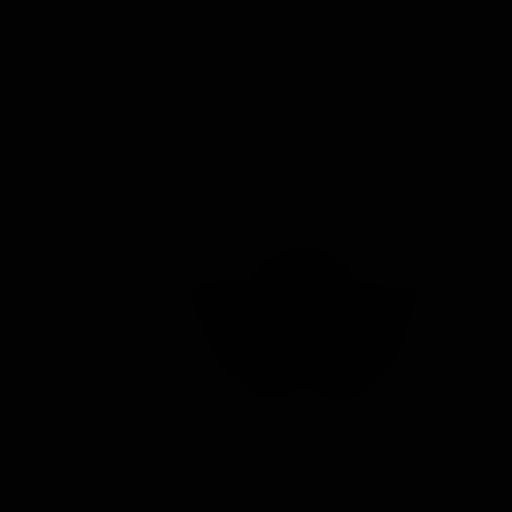

Supplement: S2 Data — (ZIP) [file pone.0295536.s003.zip › S3_Data/FCN_Training set_Label/IM_0006-ID_3014238ac.png]

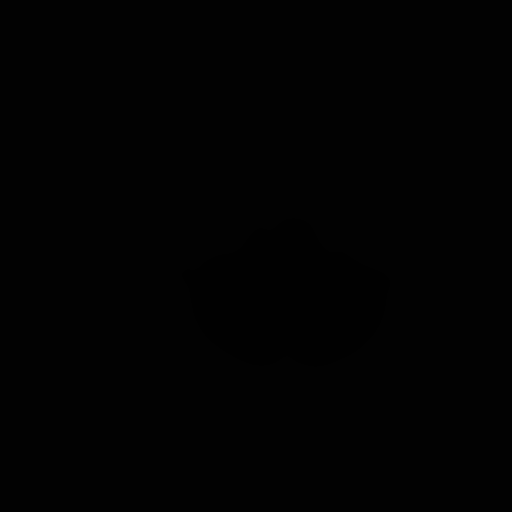

Supplement: S2 Data — (ZIP) [file pone.0295536.s003.zip › S3_Data/FCN_Training set_Label/IM_0006-ID_316935dfd.png]

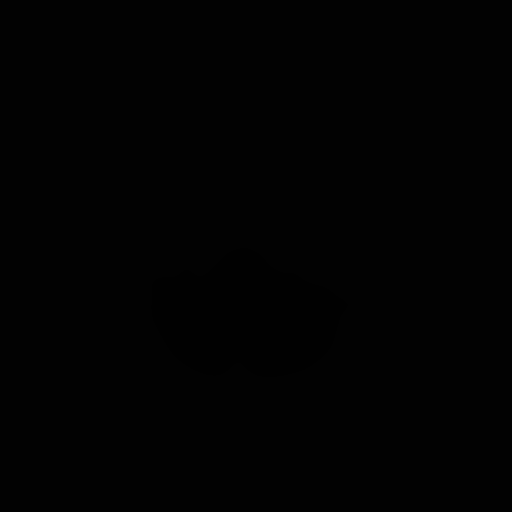

Supplement: S2 Data — (ZIP) [file pone.0295536.s003.zip › S3_Data/FCN_Training set_Label/IM_0006-ID_36be11832.png]

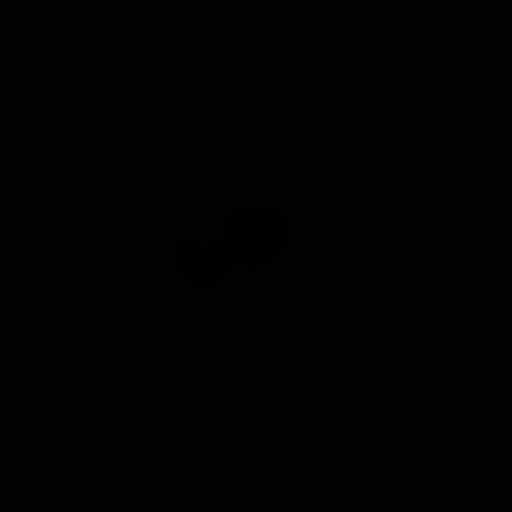

Supplement: S2 Data — (ZIP) [file pone.0295536.s003.zip › S3_Data/FCN_Training set_Label/IM_0006-ID_3714c25ac.png]

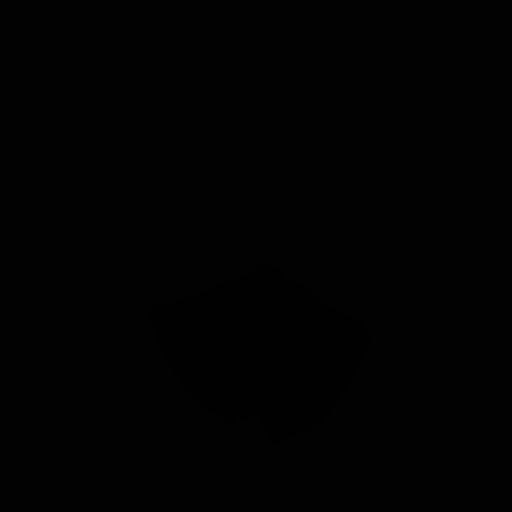

Supplement: S2 Data — (ZIP) [file pone.0295536.s003.zip › S3_Data/FCN_Training set_Label/IM_0006-ID_37c65aa7b.png]

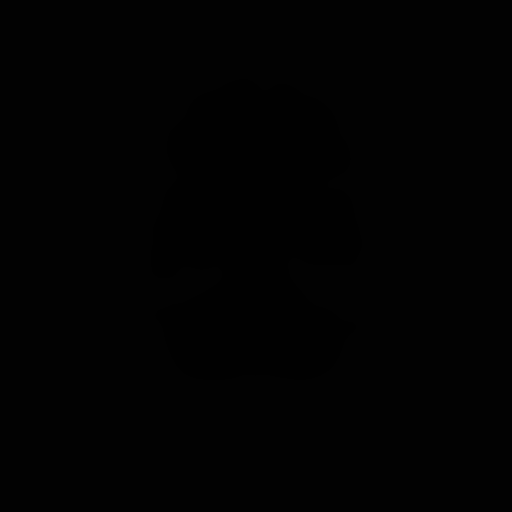

Supplement: S2 Data — (ZIP) [file pone.0295536.s003.zip › S3_Data/FCN_Training set_Label/IM_0006-ID_3f23a8b22.png]

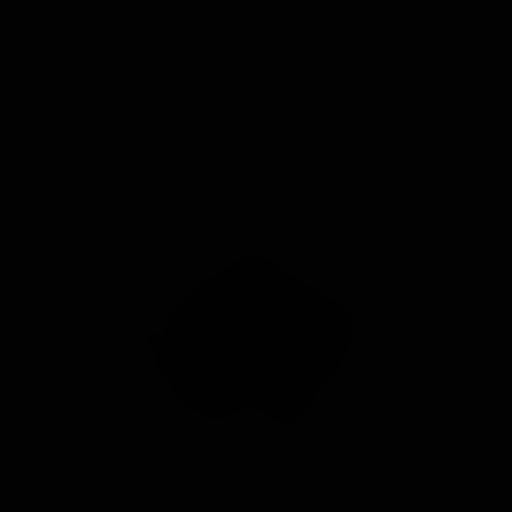

Supplement: S2 Data — (ZIP) [file pone.0295536.s003.zip › S3_Data/FCN_Training set_Label/IM_0006-ID_3f3c0ebd0.png]

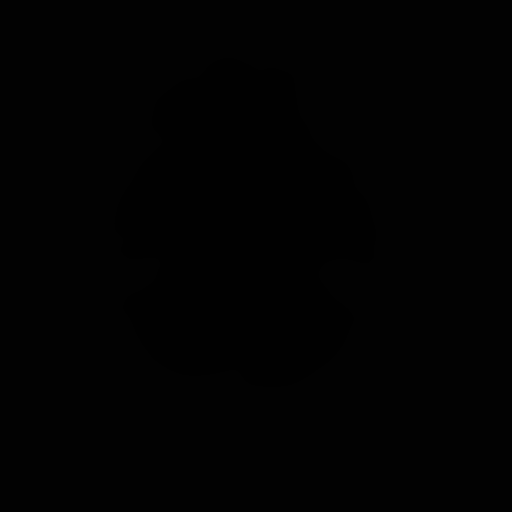

Supplement: S2 Data — (ZIP) [file pone.0295536.s003.zip › S3_Data/FCN_Training set_Label/IM_0006-ID_40627cbf6.png]

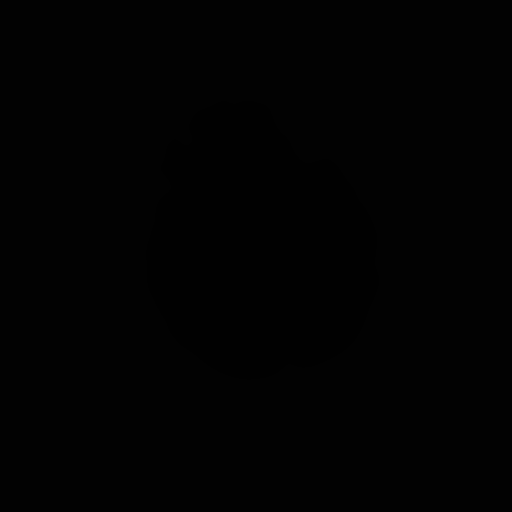

Supplement: S2 Data — (ZIP) [file pone.0295536.s003.zip › S3_Data/FCN_Training set_Label/IM_0006-ID_47daf2ff6.png]

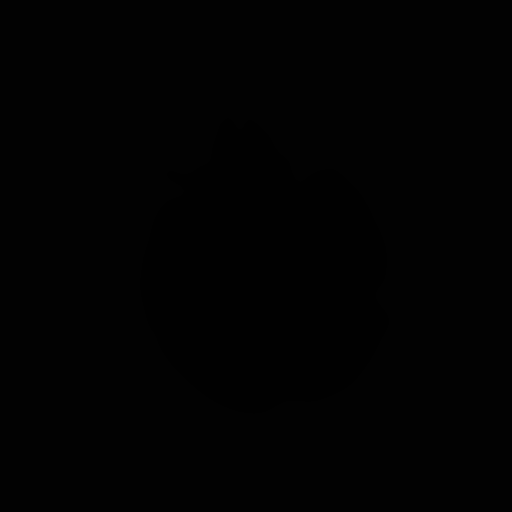

Supplement: S2 Data — (ZIP) [file pone.0295536.s003.zip › S3_Data/FCN_Training set_Label/IM_0006-ID_498ff8e23.png]

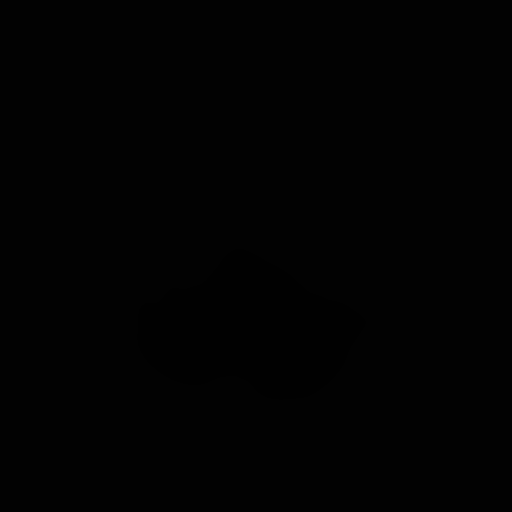

Supplement: S2 Data — (ZIP) [file pone.0295536.s003.zip › S3_Data/FCN_Training set_Label/IM_0006-ID_4caaa1f8e.png]

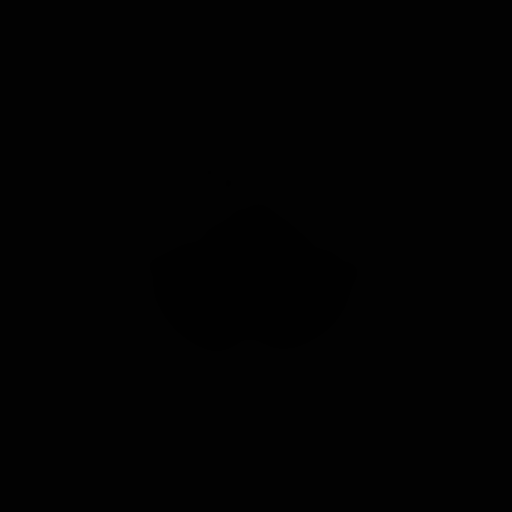

Supplement: S2 Data — (ZIP) [file pone.0295536.s003.zip › S3_Data/FCN_Training set_Label/IM_0006-ID_5030b5552.png]

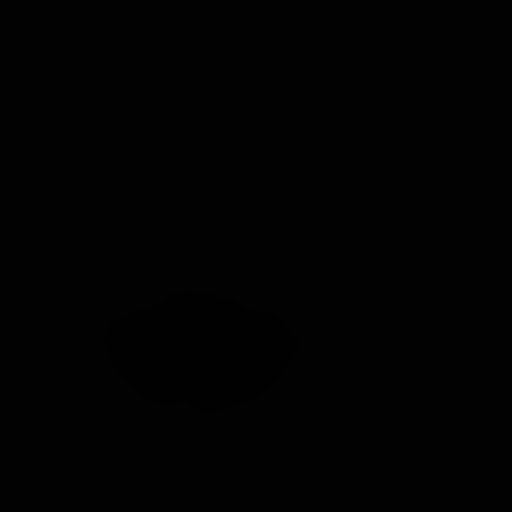

Supplement: S2 Data — (ZIP) [file pone.0295536.s003.zip › S3_Data/FCN_Training set_Label/IM_0006-ID_5146a95c0.png]

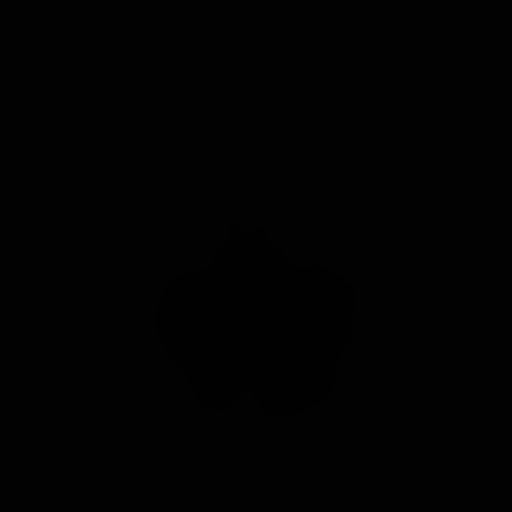

Supplement: S2 Data — (ZIP) [file pone.0295536.s003.zip › S3_Data/FCN_Training set_Label/IM_0006-ID_51ddb206d.png]

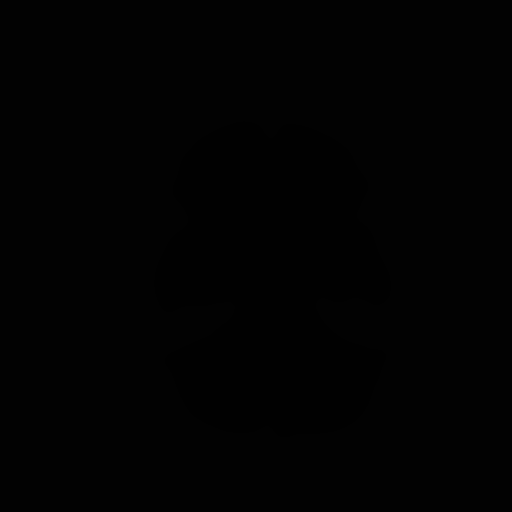

Supplement: S2 Data — (ZIP) [file pone.0295536.s003.zip › S3_Data/FCN_Training set_Label/IM_0006-ID_54264aacb.png]

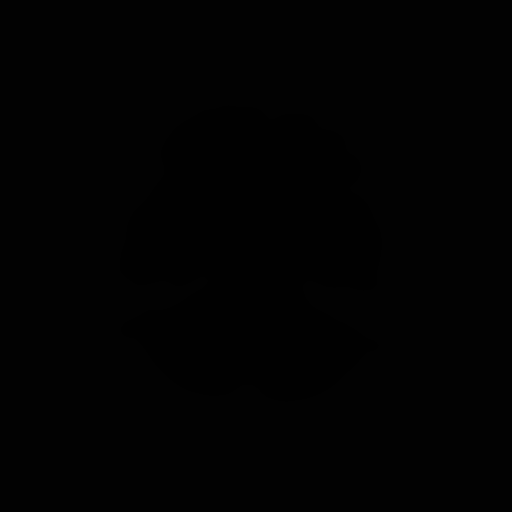

Supplement: S2 Data — (ZIP) [file pone.0295536.s003.zip › S3_Data/FCN_Training set_Label/IM_0006-ID_5527d3b1b.png]

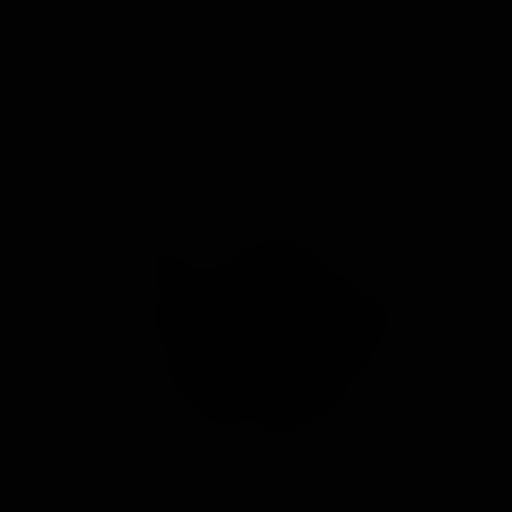

Supplement: S2 Data — (ZIP) [file pone.0295536.s003.zip › S3_Data/FCN_Training set_Label/IM_0006-ID_5cabd9ce5.png]

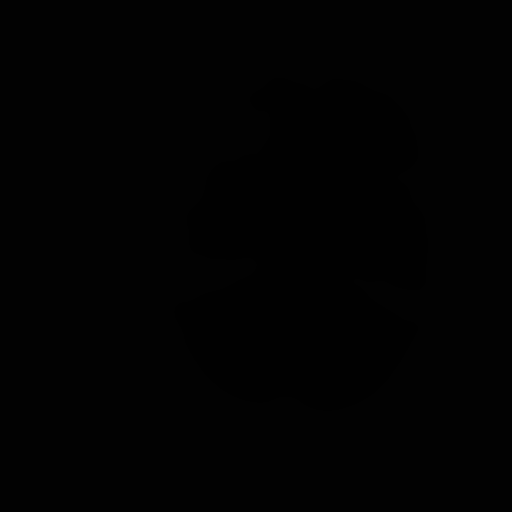

Supplement: S2 Data — (ZIP) [file pone.0295536.s003.zip › S3_Data/FCN_Training set_Label/IM_0006-ID_5d7b67621.png]

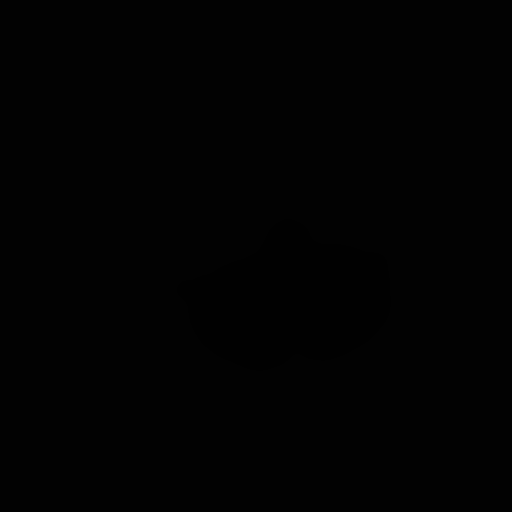

Supplement: S2 Data — (ZIP) [file pone.0295536.s003.zip › S3_Data/FCN_Training set_Label/IM_0006-ID_5daa789db.png]

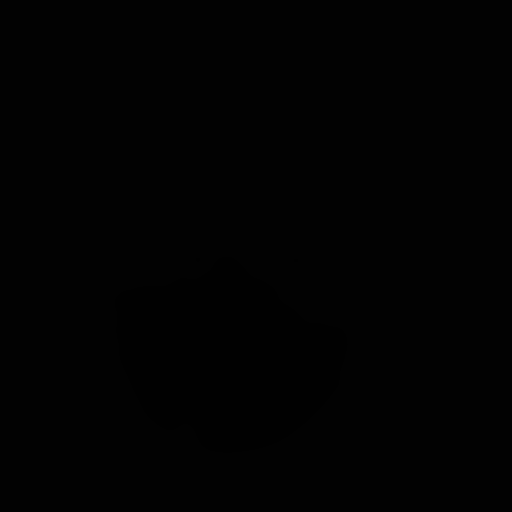

Supplement: S2 Data — (ZIP) [file pone.0295536.s003.zip › S3_Data/FCN_Training set_Label/IM_0006-ID_5e4513613.png]

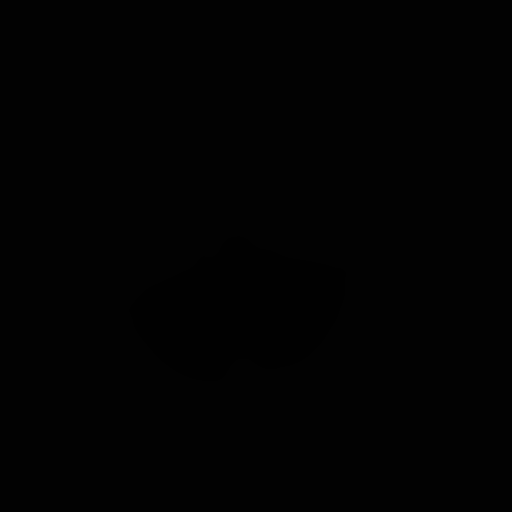

Supplement: S2 Data — (ZIP) [file pone.0295536.s003.zip › S3_Data/FCN_Training set_Label/IM_0006-ID_5ebee68a1.png]

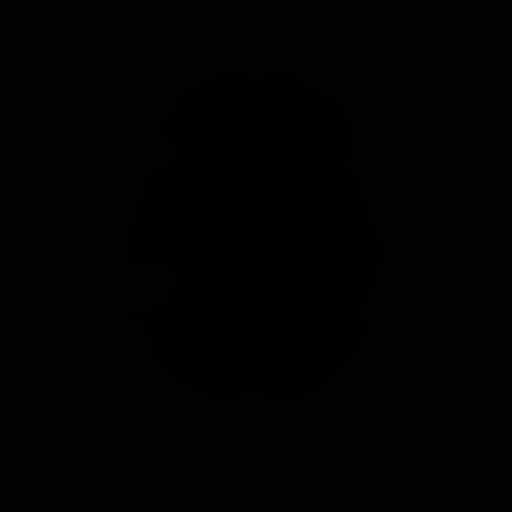

Supplement: S2 Data — (ZIP) [file pone.0295536.s003.zip › S3_Data/FCN_Training set_Label/IM_0006-ID_6081e25af.png]

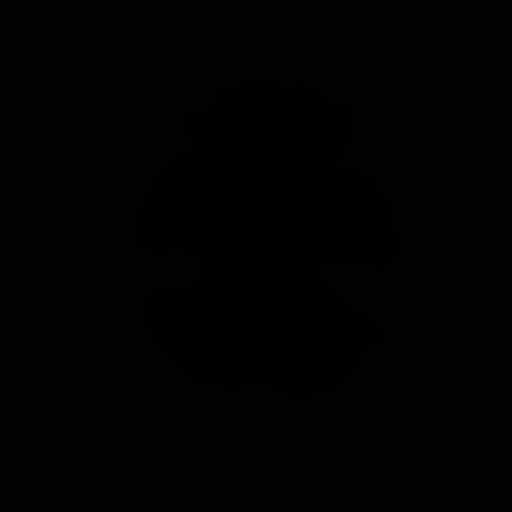

Supplement: S2 Data — (ZIP) [file pone.0295536.s003.zip › S3_Data/FCN_Training set_Label/IM_0006-ID_6354222be.png]

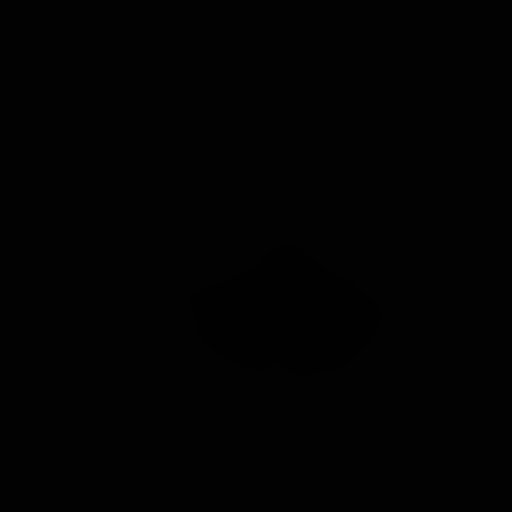

Supplement: S2 Data — (ZIP) [file pone.0295536.s003.zip › S3_Data/FCN_Training set_Label/IM_0006-ID_63d154b5b.png]

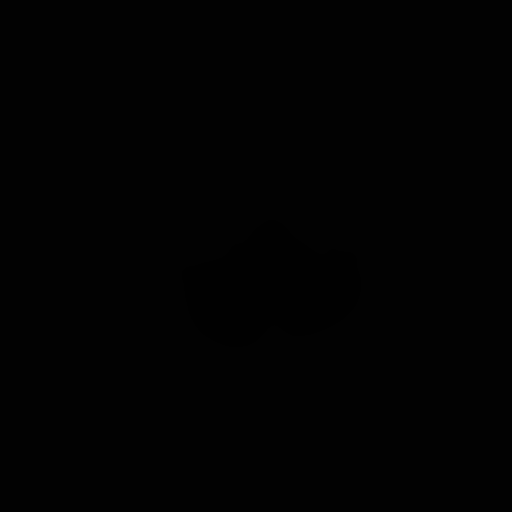

Supplement: S2 Data — (ZIP) [file pone.0295536.s003.zip › S3_Data/FCN_Training set_Label/IM_0006-ID_64b80423e.png]

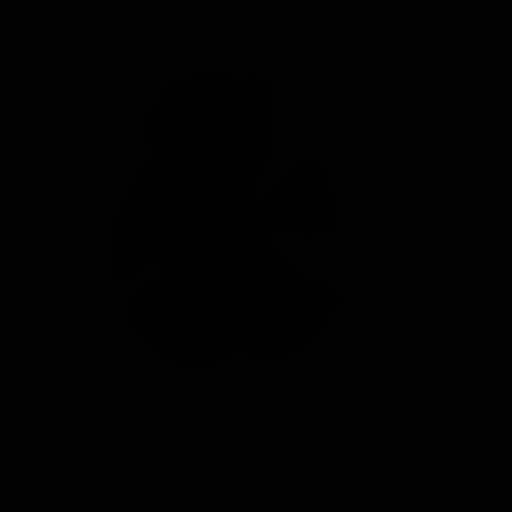

Supplement: S2 Data — (ZIP) [file pone.0295536.s003.zip › S3_Data/FCN_Training set_Label/IM_0006-ID_661b9746c.png]

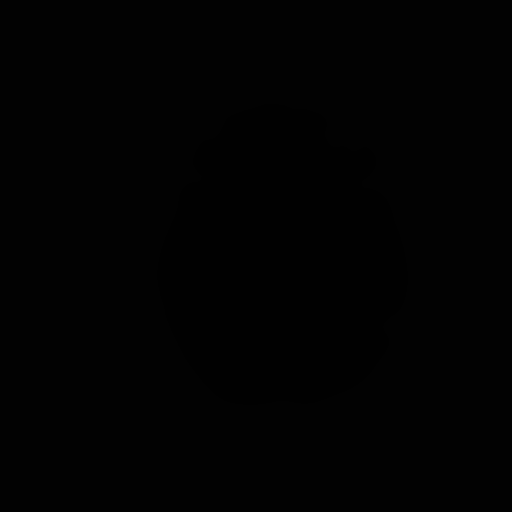

Supplement: S2 Data — (ZIP) [file pone.0295536.s003.zip › S3_Data/FCN_Training set_Label/IM_0006-ID_67c080622.png]

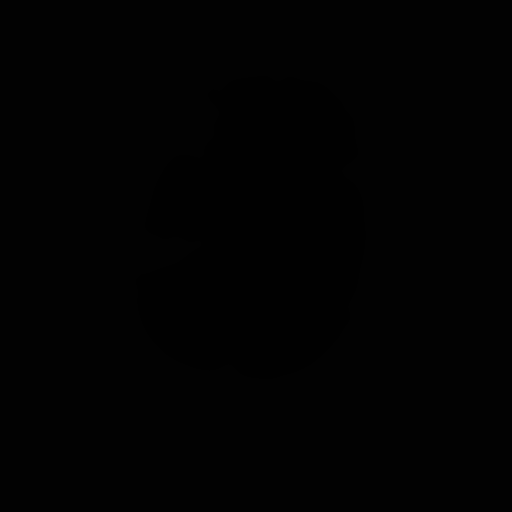

Supplement: S2 Data — (ZIP) [file pone.0295536.s003.zip › S3_Data/FCN_Training set_Label/IM_0006-ID_69045d409.png]

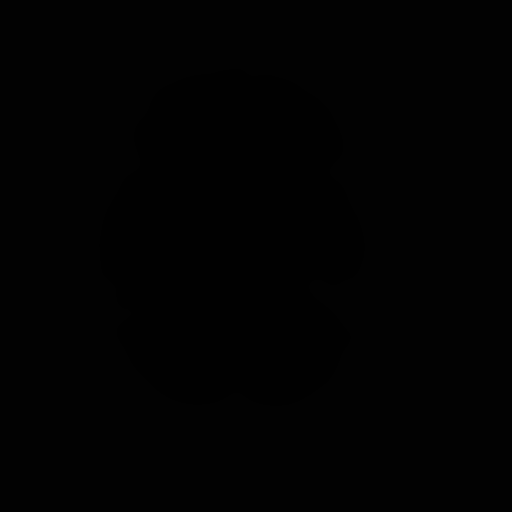

Supplement: S2 Data — (ZIP) [file pone.0295536.s003.zip › S3_Data/FCN_Training set_Label/IM_0006-ID_69dbfc6e8.png]

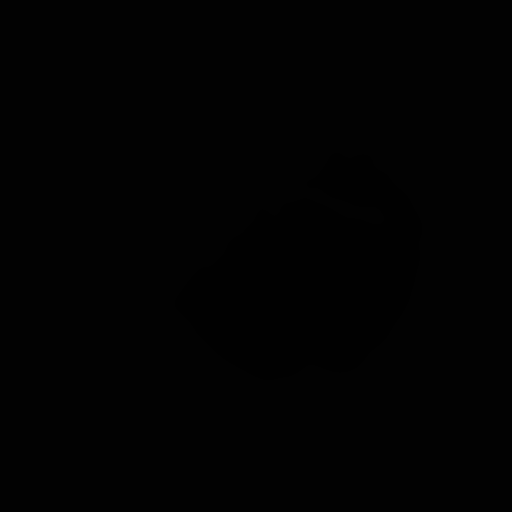

Supplement: S2 Data — (ZIP) [file pone.0295536.s003.zip › S3_Data/FCN_Training set_Label/IM_0006-ID_6bb18579c.png]

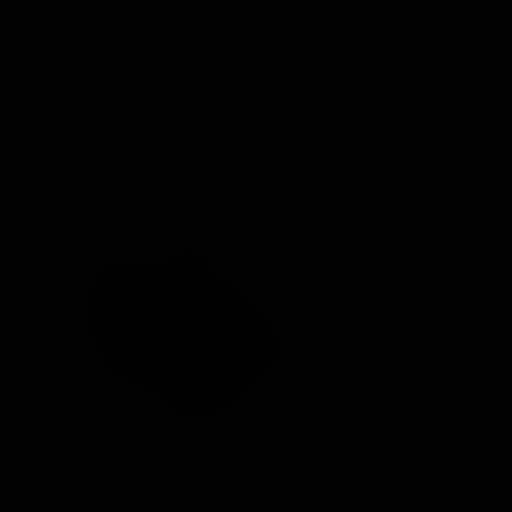

Supplement: S2 Data — (ZIP) [file pone.0295536.s003.zip › S3_Data/FCN_Training set_Label/IM_0006-ID_6e3209e3a.png]

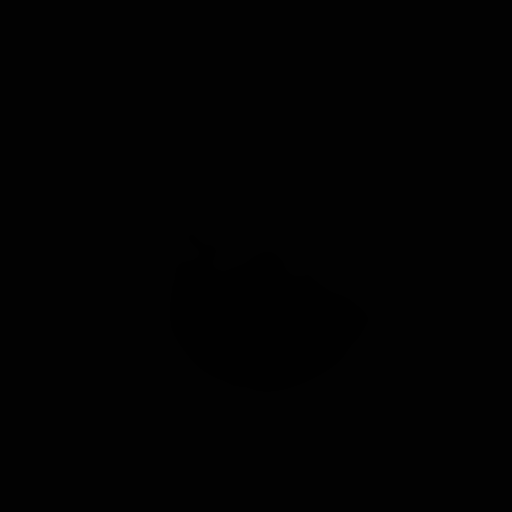

Supplement: S2 Data — (ZIP) [file pone.0295536.s003.zip › S3_Data/FCN_Training set_Label/IM_0006-ID_6fc93dc0a.png]

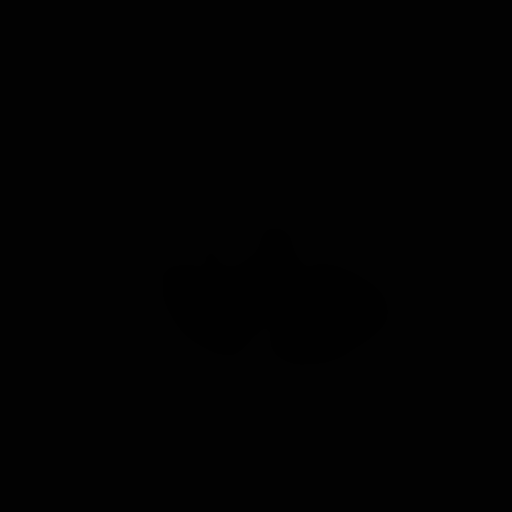

Supplement: S2 Data — (ZIP) [file pone.0295536.s003.zip › S3_Data/FCN_Training set_Label/IM_0006-ID_72b24af72.png]

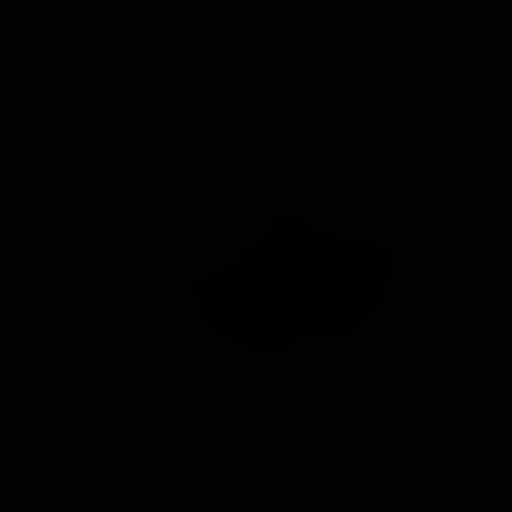

Supplement: S2 Data — (ZIP) [file pone.0295536.s003.zip › S3_Data/FCN_Training set_Label/IM_0006-ID_786517811.png]

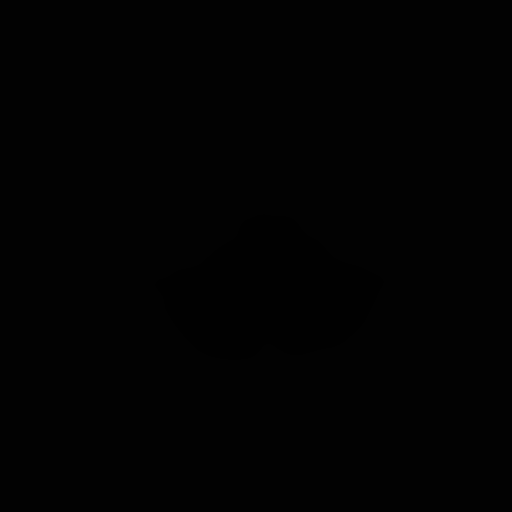

Supplement: S2 Data — (ZIP) [file pone.0295536.s003.zip › S3_Data/FCN_Training set_Label/IM_0006-ID_7a7703fef.png]

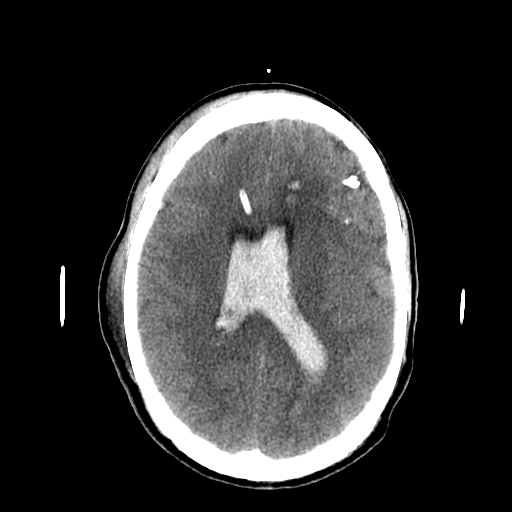

Supplement: S3 Data — (ZIP) [file pone.0295536.s004.zip › S4_Data/FCN_Training set/111111111111111111111111111111111111111111111111.png]
