# Supplementary figures and images for: Adaptive mask-based brain extraction method for head CT images (part 3 of 14)
Source: PLoS One. 2024 Mar 11;19(3):e0295536. doi: 10.1371/journal.pone.0295536 (PMC10927156; doi:10.1371/journal.pone.0295536)

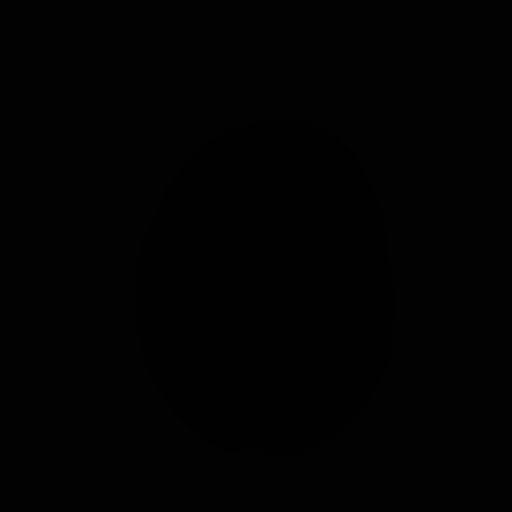

Supplement: S2 Data — (ZIP) [file pone.0295536.s003.zip › S3_Data/FCN_Training set_Label/111111111111111111111111111111111111111111111111.png]

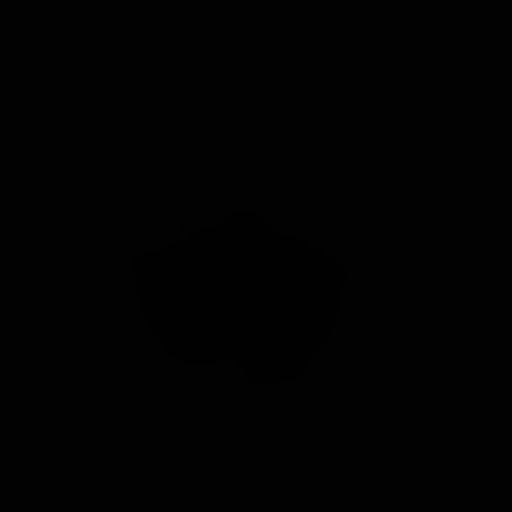

Supplement: S2 Data — (ZIP) [file pone.0295536.s003.zip › S3_Data/FCN_Training set_Label/IM_0000-ID_176d479f0.png]

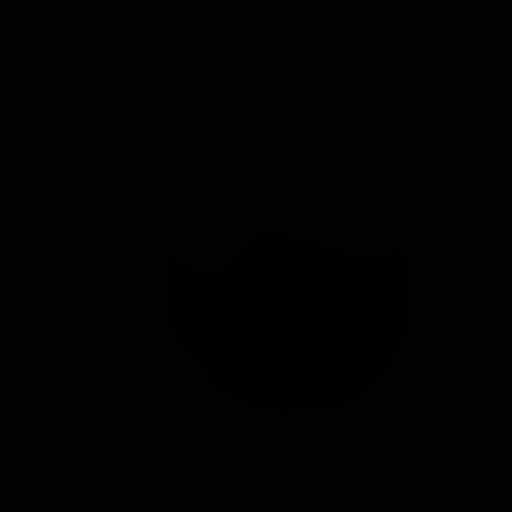

Supplement: S2 Data — (ZIP) [file pone.0295536.s003.zip › S3_Data/FCN_Training set_Label/IM_0000-ID_7a03bfffe.png]

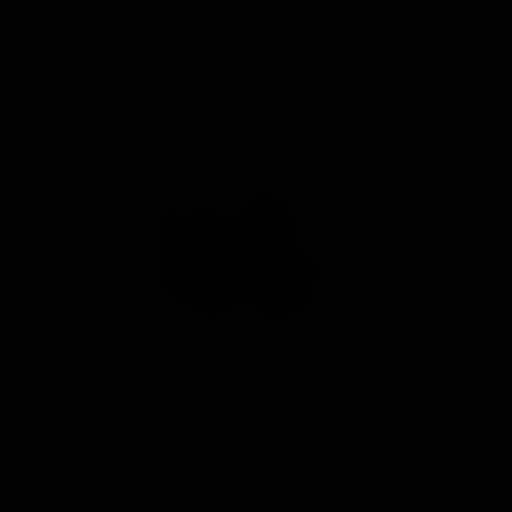

Supplement: S2 Data — (ZIP) [file pone.0295536.s003.zip › S3_Data/FCN_Training set_Label/IM_0000-ID_d9b2ec9d8.png]

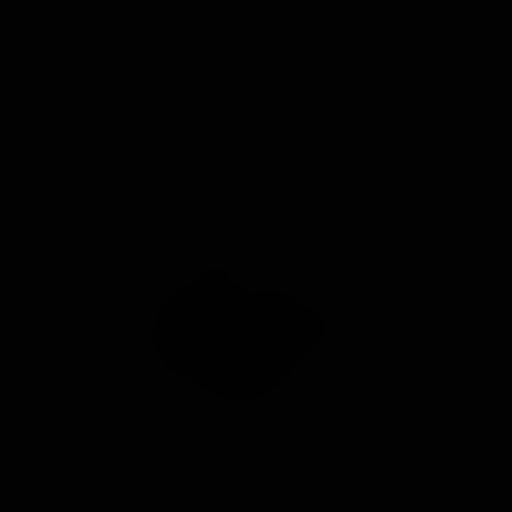

Supplement: S2 Data — (ZIP) [file pone.0295536.s003.zip › S3_Data/FCN_Training set_Label/IM_0001-ID_1af1e5400.png]

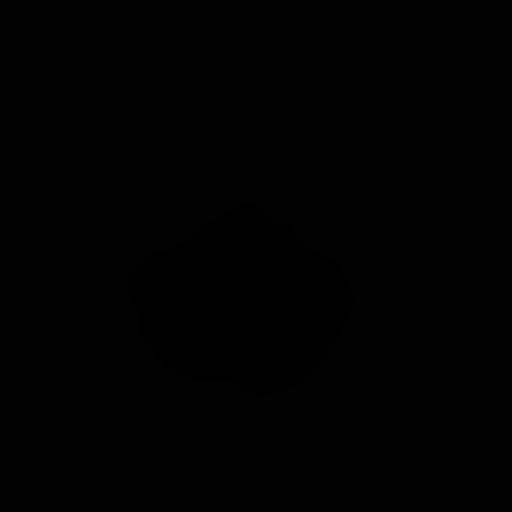

Supplement: S2 Data — (ZIP) [file pone.0295536.s003.zip › S3_Data/FCN_Training set_Label/IM_0001-ID_92a62e48b.png]

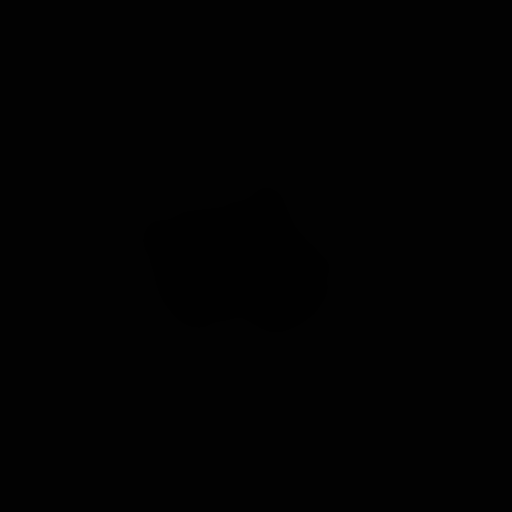

Supplement: S2 Data — (ZIP) [file pone.0295536.s003.zip › S3_Data/FCN_Training set_Label/IM_0001-ID_a6c4d9a1f.png]

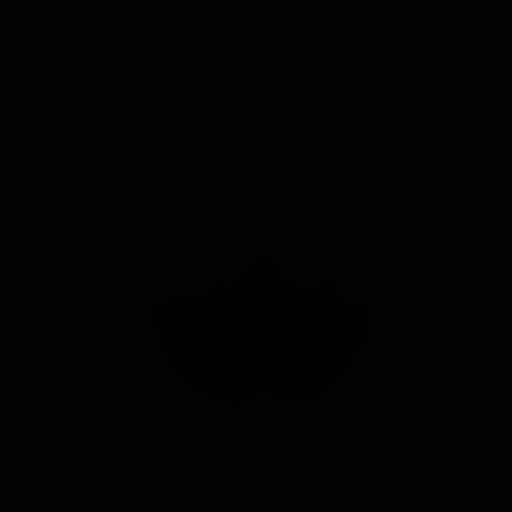

Supplement: S2 Data — (ZIP) [file pone.0295536.s003.zip › S3_Data/FCN_Training set_Label/IM_0001-ID_f966aa3f7.png]

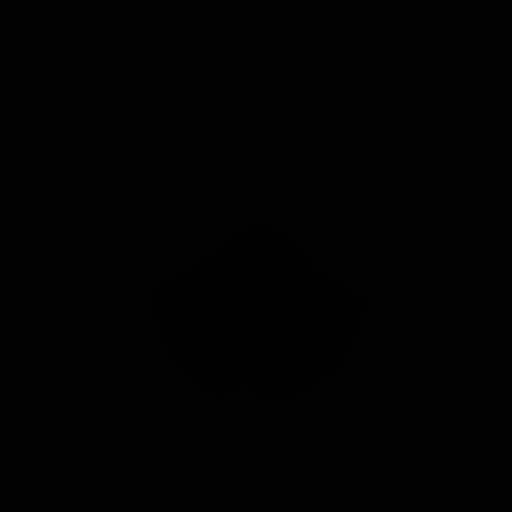

Supplement: S2 Data — (ZIP) [file pone.0295536.s003.zip › S3_Data/FCN_Training set_Label/IM_0002-ID_3171d7dd5.png]

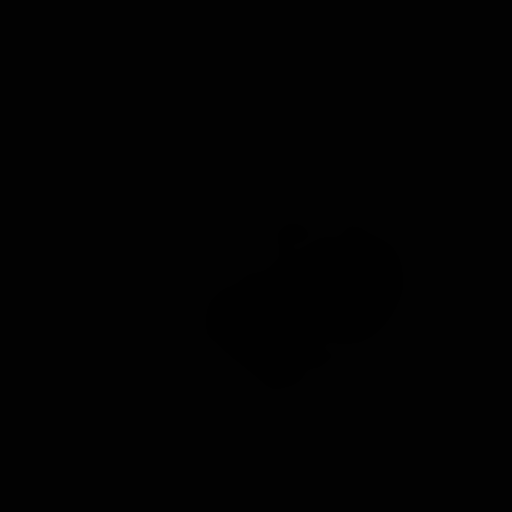

Supplement: S2 Data — (ZIP) [file pone.0295536.s003.zip › S3_Data/FCN_Training set_Label/IM_0002-ID_57ba5653e.png]

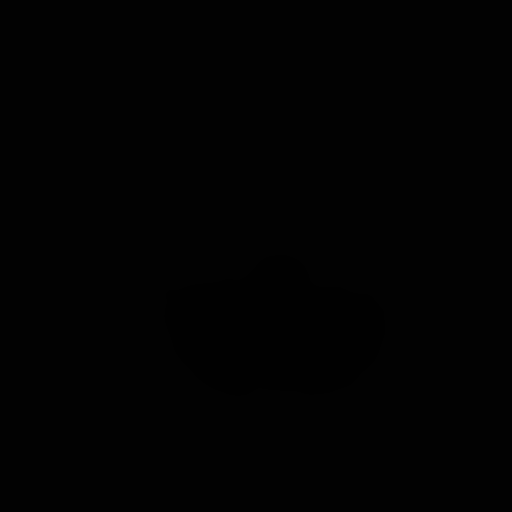

Supplement: S2 Data — (ZIP) [file pone.0295536.s003.zip › S3_Data/FCN_Training set_Label/IM_0002-ID_650fa637e.png]

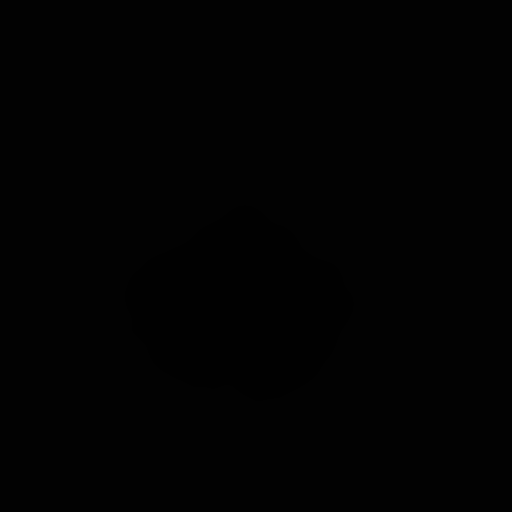

Supplement: S2 Data — (ZIP) [file pone.0295536.s003.zip › S3_Data/FCN_Training set_Label/IM_0002-ID_b046104e3.png]

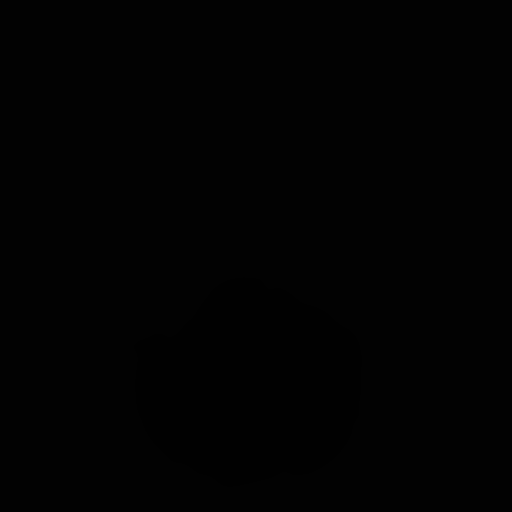

Supplement: S2 Data — (ZIP) [file pone.0295536.s003.zip › S3_Data/FCN_Training set_Label/IM_0002-ID_b48466cfe.png]

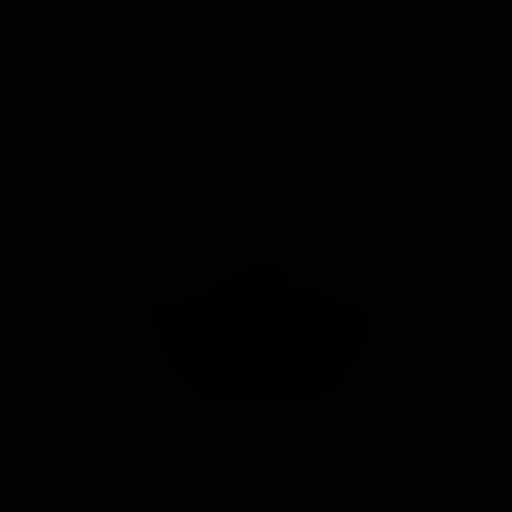

Supplement: S2 Data — (ZIP) [file pone.0295536.s003.zip › S3_Data/FCN_Training set_Label/IM_0002-ID_b5246900c.png]

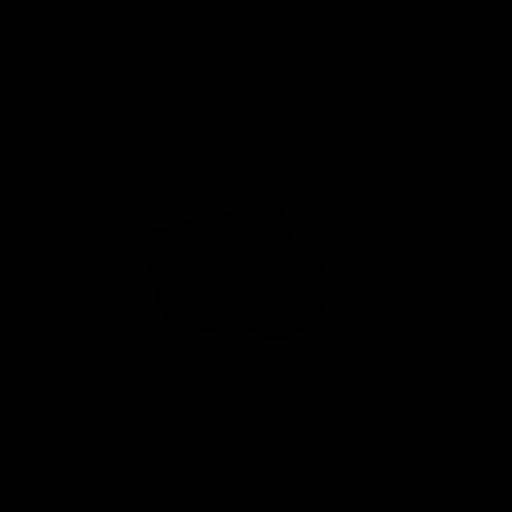

Supplement: S2 Data — (ZIP) [file pone.0295536.s003.zip › S3_Data/FCN_Training set_Label/IM_0002-ID_cbd45c817.png]

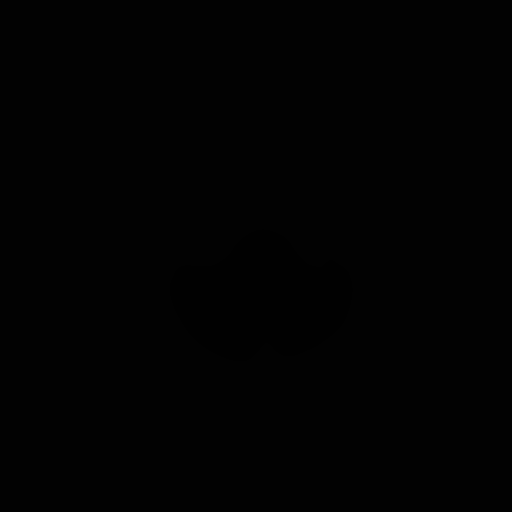

Supplement: S2 Data — (ZIP) [file pone.0295536.s003.zip › S3_Data/FCN_Training set_Label/IM_0003-ID_093afc8d9.png]

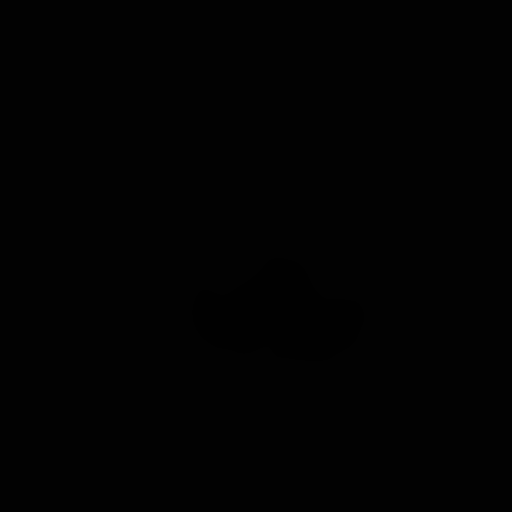

Supplement: S2 Data — (ZIP) [file pone.0295536.s003.zip › S3_Data/FCN_Training set_Label/IM_0003-ID_0b9be23bf.png]

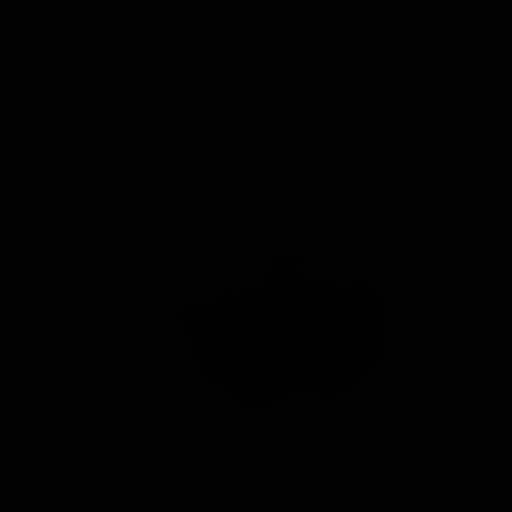

Supplement: S2 Data — (ZIP) [file pone.0295536.s003.zip › S3_Data/FCN_Training set_Label/IM_0003-ID_1b397a5f3.png]

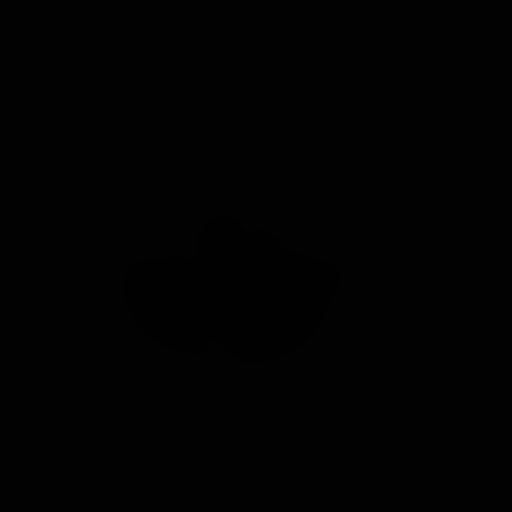

Supplement: S2 Data — (ZIP) [file pone.0295536.s003.zip › S3_Data/FCN_Training set_Label/IM_0003-ID_1b73592a4.png]

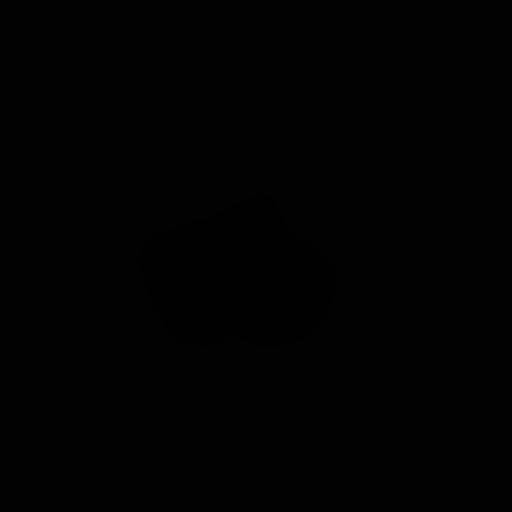

Supplement: S2 Data — (ZIP) [file pone.0295536.s003.zip › S3_Data/FCN_Training set_Label/IM_0003-ID_31cda61a0.png]

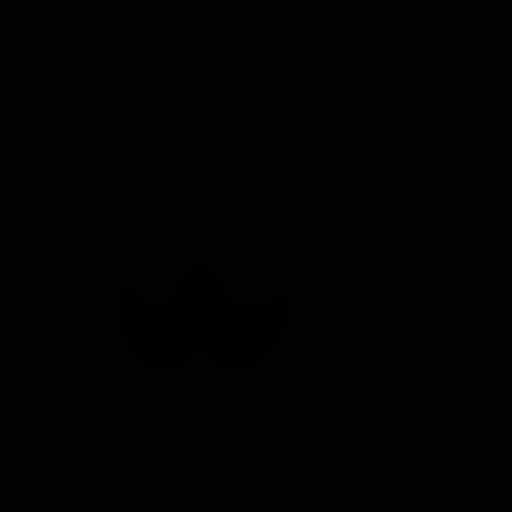

Supplement: S2 Data — (ZIP) [file pone.0295536.s003.zip › S3_Data/FCN_Training set_Label/IM_0003-ID_6459d6004.png]

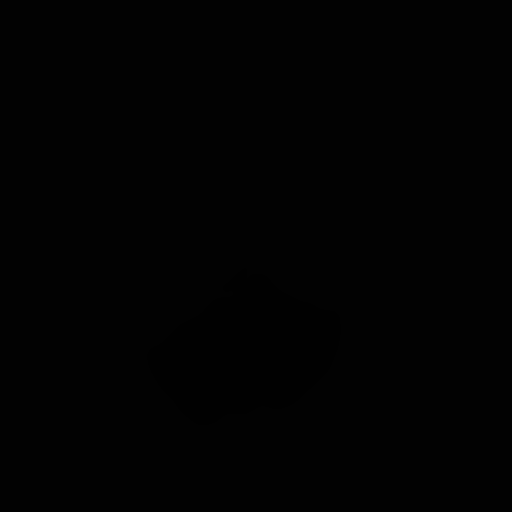

Supplement: S2 Data — (ZIP) [file pone.0295536.s003.zip › S3_Data/FCN_Training set_Label/IM_0003-ID_68f6fac67.png]

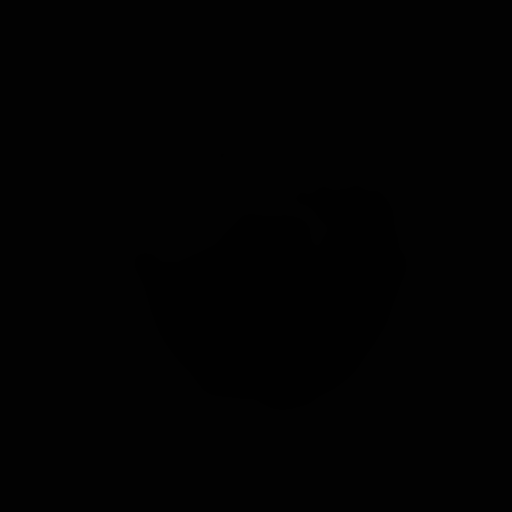

Supplement: S2 Data — (ZIP) [file pone.0295536.s003.zip › S3_Data/FCN_Training set_Label/IM_0003-ID_72df67677.png]

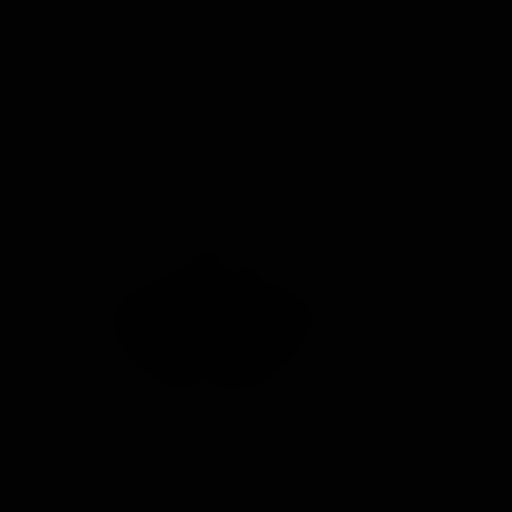

Supplement: S2 Data — (ZIP) [file pone.0295536.s003.zip › S3_Data/FCN_Training set_Label/IM_0003-ID_7425a7f20.png]

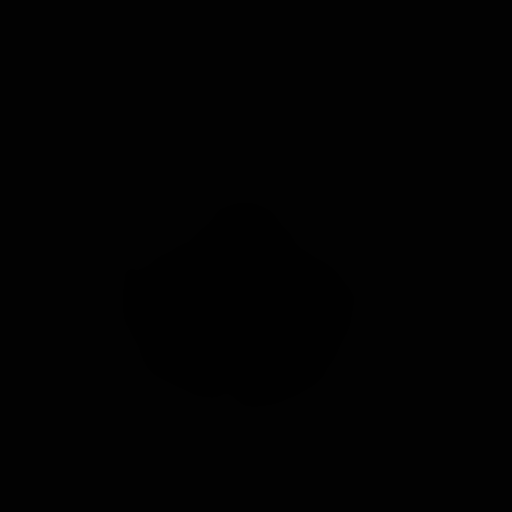

Supplement: S2 Data — (ZIP) [file pone.0295536.s003.zip › S3_Data/FCN_Training set_Label/IM_0003-ID_79b034a02.png]

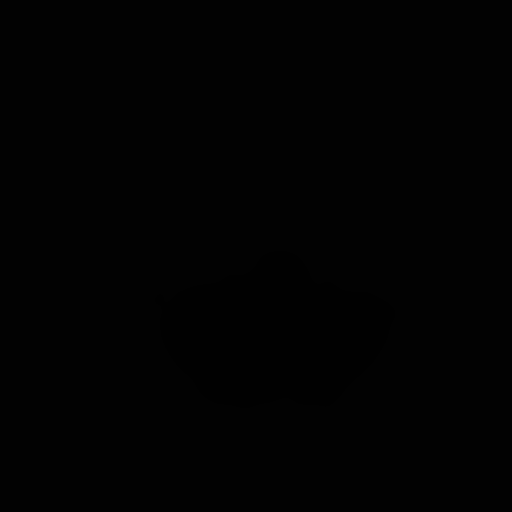

Supplement: S2 Data — (ZIP) [file pone.0295536.s003.zip › S3_Data/FCN_Training set_Label/IM_0003-ID_84cbf705b.png]

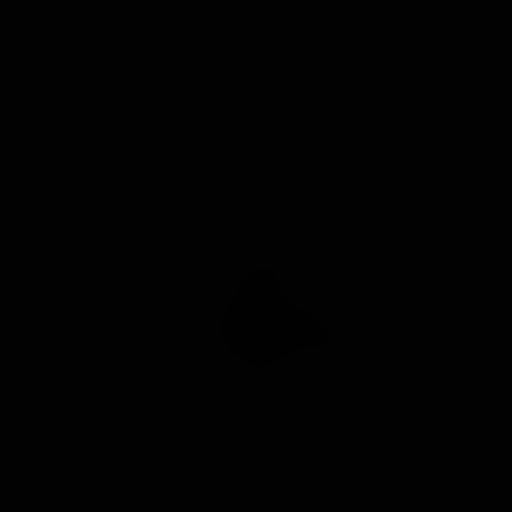

Supplement: S2 Data — (ZIP) [file pone.0295536.s003.zip › S3_Data/FCN_Training set_Label/IM_0003-ID_962a5821f.png]

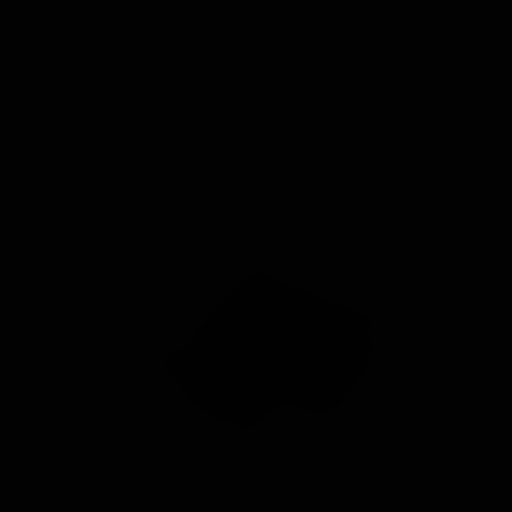

Supplement: S2 Data — (ZIP) [file pone.0295536.s003.zip › S3_Data/FCN_Training set_Label/IM_0003-ID_a01c96213.png]

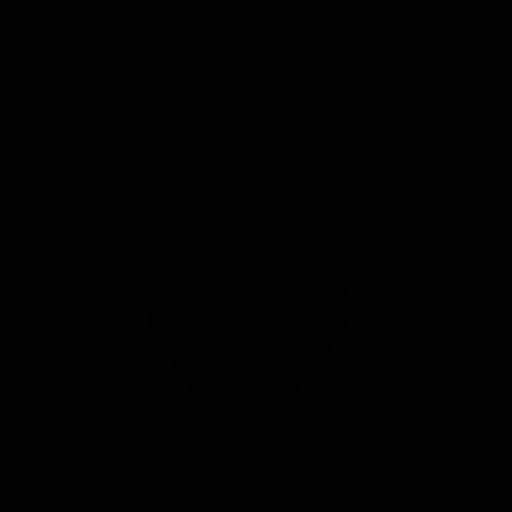

Supplement: S2 Data — (ZIP) [file pone.0295536.s003.zip › S3_Data/FCN_Training set_Label/IM_0003-ID_a2845b9aa.png]

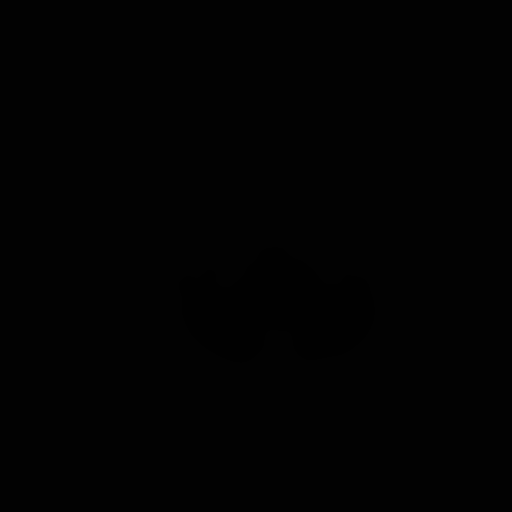

Supplement: S2 Data — (ZIP) [file pone.0295536.s003.zip › S3_Data/FCN_Training set_Label/IM_0003-ID_b569710c4.png]

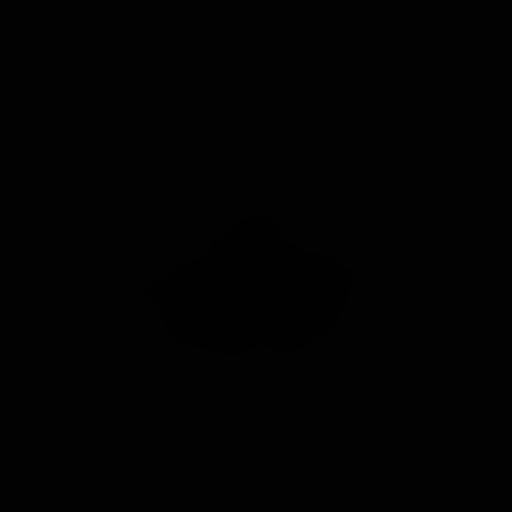

Supplement: S2 Data — (ZIP) [file pone.0295536.s003.zip › S3_Data/FCN_Training set_Label/IM_0003-ID_b5bba4428.png]

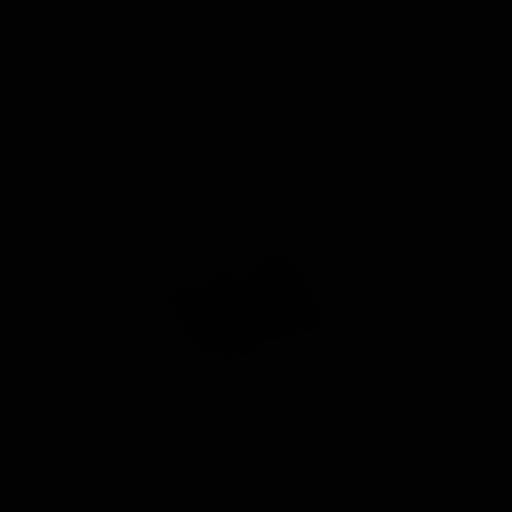

Supplement: S2 Data — (ZIP) [file pone.0295536.s003.zip › S3_Data/FCN_Training set_Label/IM_0003-ID_d6a3dd87a.png]

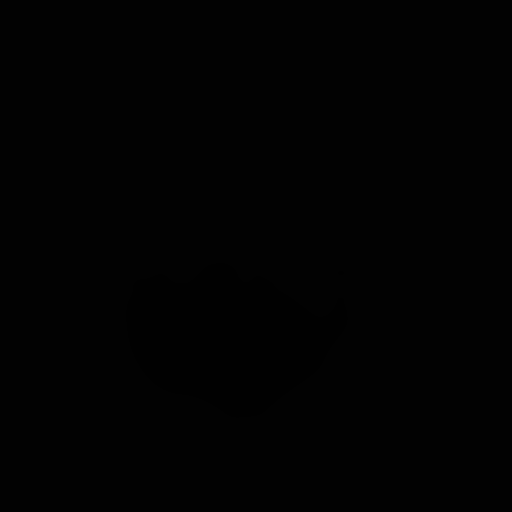

Supplement: S2 Data — (ZIP) [file pone.0295536.s003.zip › S3_Data/FCN_Training set_Label/IM_0003-ID_db013d767.png]

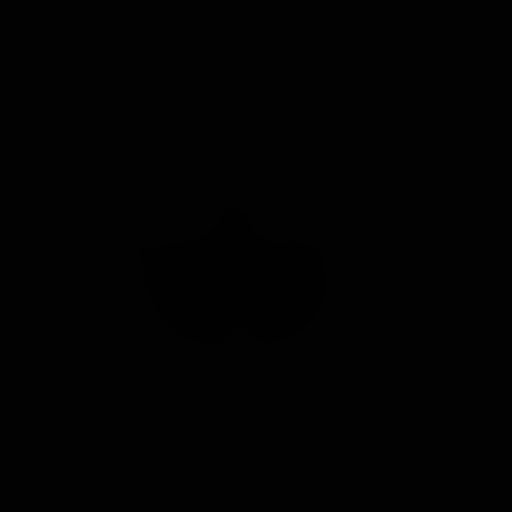

Supplement: S2 Data — (ZIP) [file pone.0295536.s003.zip › S3_Data/FCN_Training set_Label/IM_0003-ID_e16af557c.png]

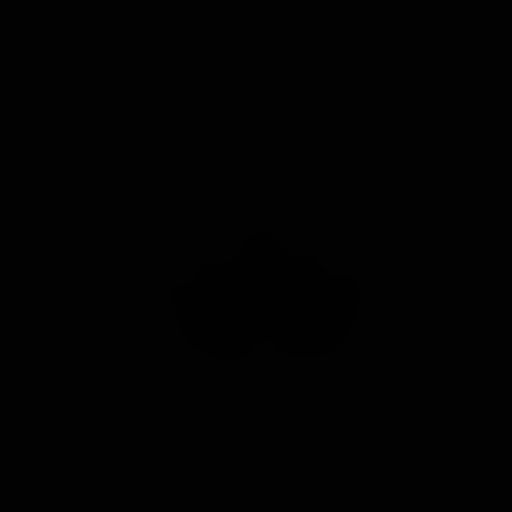

Supplement: S2 Data — (ZIP) [file pone.0295536.s003.zip › S3_Data/FCN_Training set_Label/IM_0004-ID_039c562c4.png]

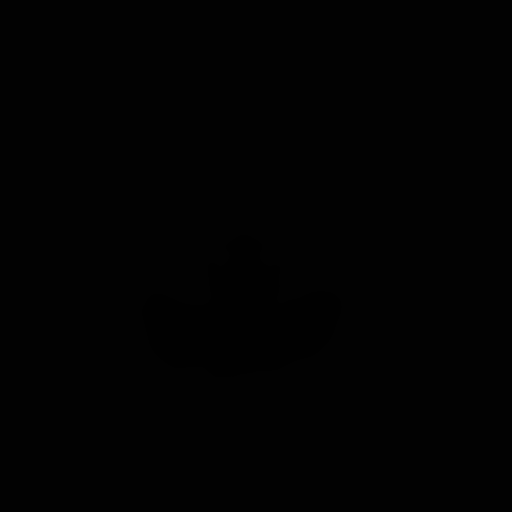

Supplement: S2 Data — (ZIP) [file pone.0295536.s003.zip › S3_Data/FCN_Training set_Label/IM_0004-ID_08b378514.png]

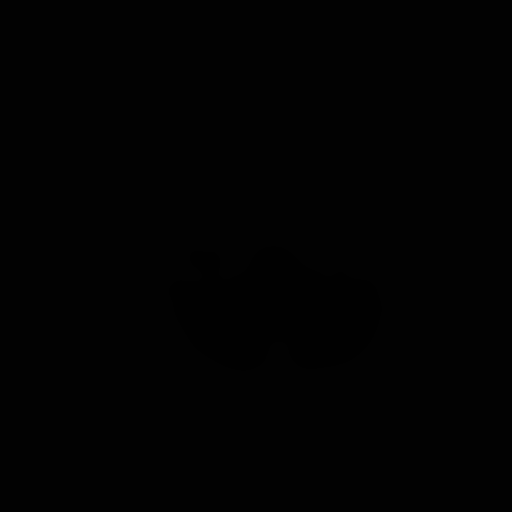

Supplement: S2 Data — (ZIP) [file pone.0295536.s003.zip › S3_Data/FCN_Training set_Label/IM_0004-ID_0c4647359.png]

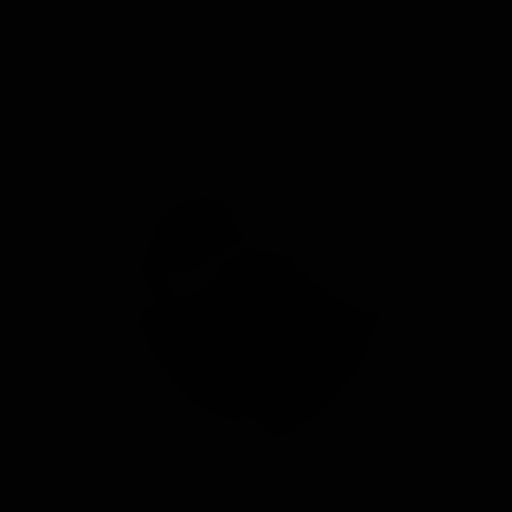

Supplement: S2 Data — (ZIP) [file pone.0295536.s003.zip › S3_Data/FCN_Training set_Label/IM_0004-ID_1327f057b.png]

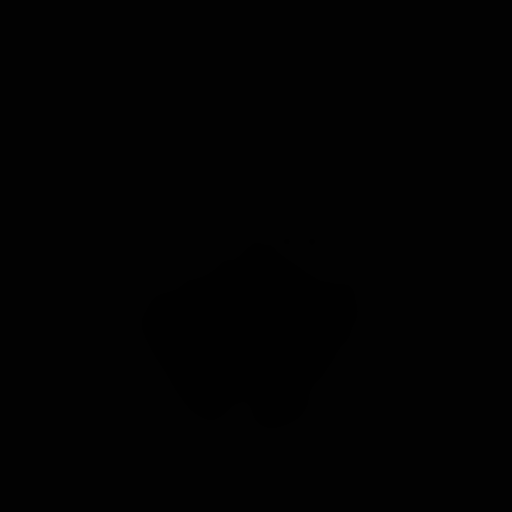

Supplement: S2 Data — (ZIP) [file pone.0295536.s003.zip › S3_Data/FCN_Training set_Label/IM_0004-ID_2523297e5.png]

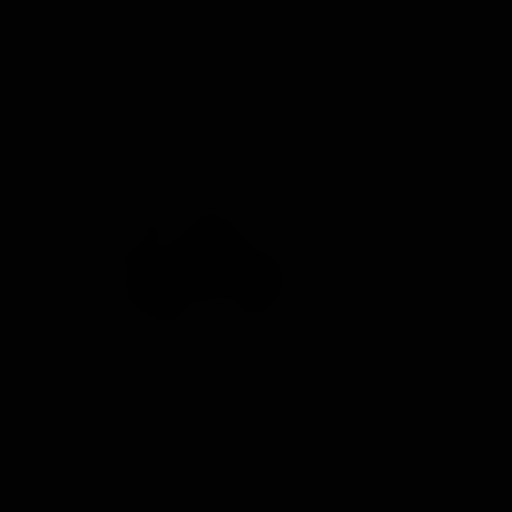

Supplement: S2 Data — (ZIP) [file pone.0295536.s003.zip › S3_Data/FCN_Training set_Label/IM_0004-ID_28c444c9c.png]

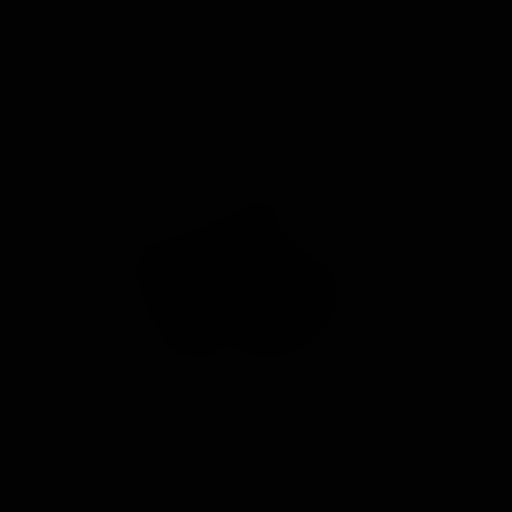

Supplement: S2 Data — (ZIP) [file pone.0295536.s003.zip › S3_Data/FCN_Training set_Label/IM_0004-ID_337dfa8c2.png]

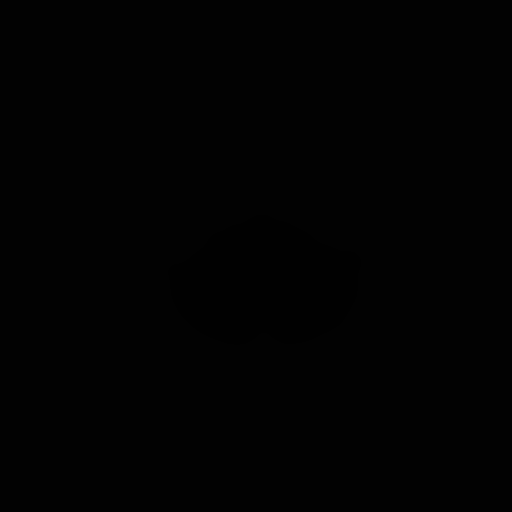

Supplement: S2 Data — (ZIP) [file pone.0295536.s003.zip › S3_Data/FCN_Training set_Label/IM_0004-ID_338afb4e7.png]

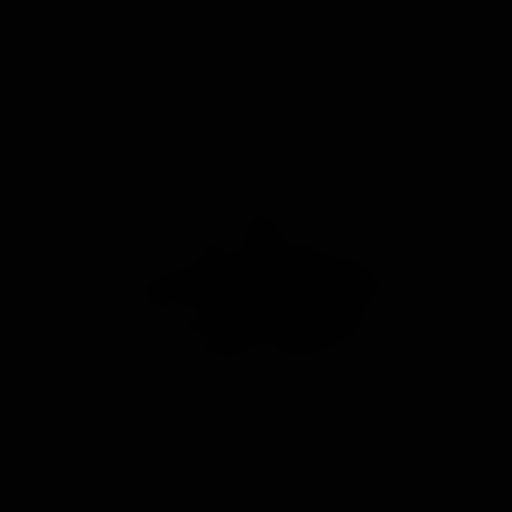

Supplement: S2 Data — (ZIP) [file pone.0295536.s003.zip › S3_Data/FCN_Training set_Label/IM_0004-ID_3762a9d52.png]

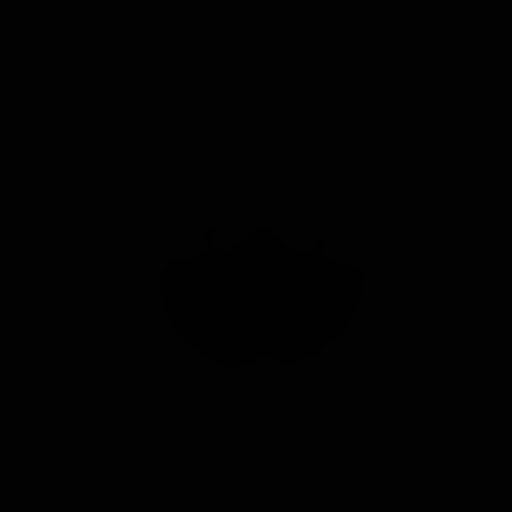

Supplement: S2 Data — (ZIP) [file pone.0295536.s003.zip › S3_Data/FCN_Training set_Label/IM_0004-ID_3ffb00bcd.png]

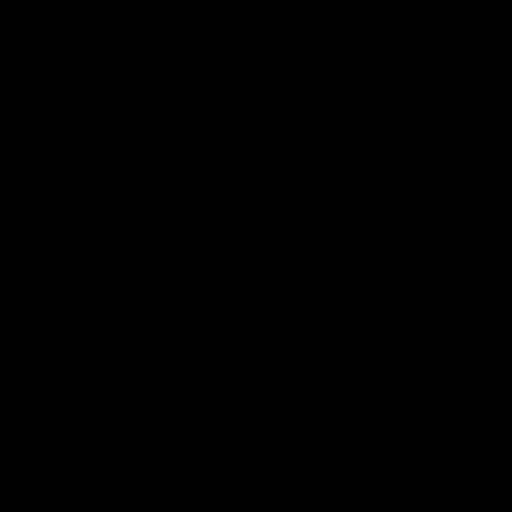

Supplement: S2 Data — (ZIP) [file pone.0295536.s003.zip › S3_Data/FCN_Training set_Label/IM_0004-ID_460d6c7ce.png]

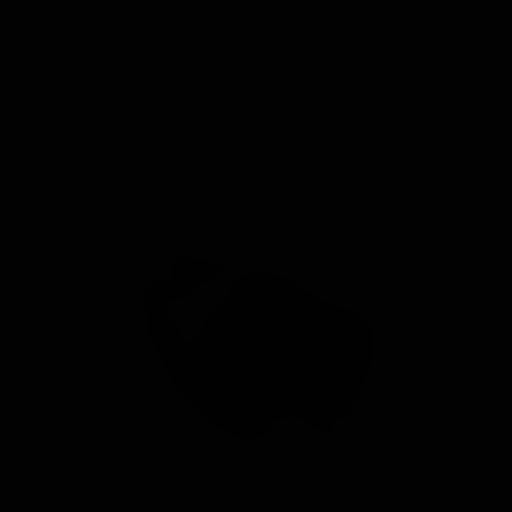

Supplement: S2 Data — (ZIP) [file pone.0295536.s003.zip › S3_Data/FCN_Training set_Label/IM_0004-ID_4ae18ff3e.png]

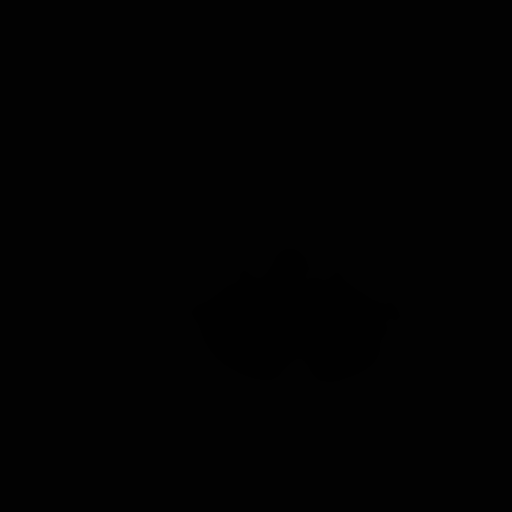

Supplement: S2 Data — (ZIP) [file pone.0295536.s003.zip › S3_Data/FCN_Training set_Label/IM_0004-ID_4bcdf8cba.png]

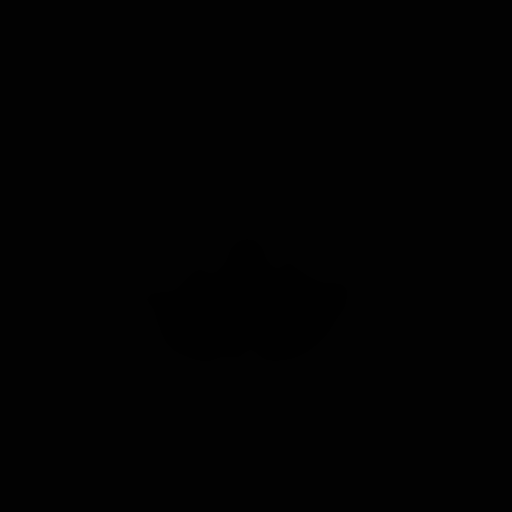

Supplement: S2 Data — (ZIP) [file pone.0295536.s003.zip › S3_Data/FCN_Training set_Label/IM_0004-ID_4f349b39a.png]

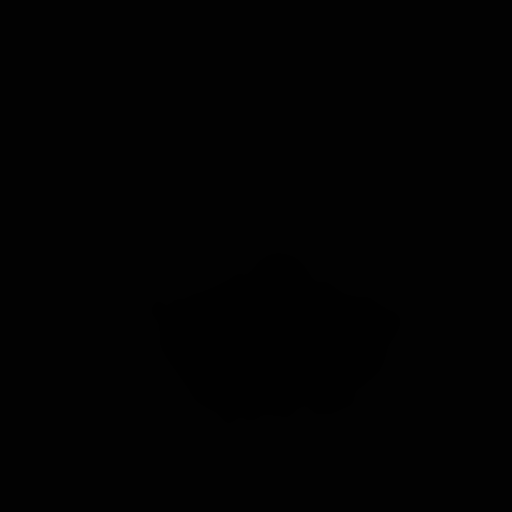

Supplement: S2 Data — (ZIP) [file pone.0295536.s003.zip › S3_Data/FCN_Training set_Label/IM_0004-ID_52fcdbd14.png]

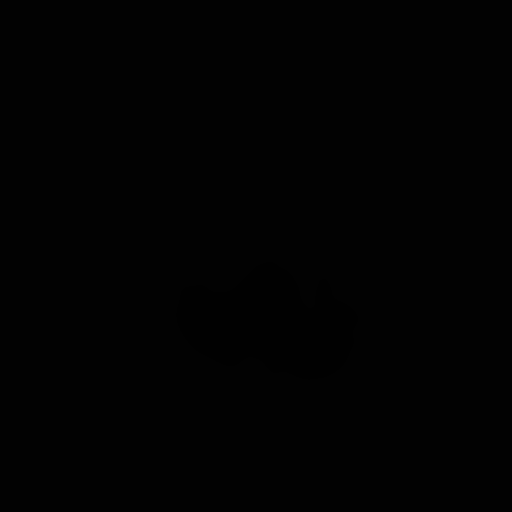

Supplement: S2 Data — (ZIP) [file pone.0295536.s003.zip › S3_Data/FCN_Training set_Label/IM_0004-ID_566c4978e.png]

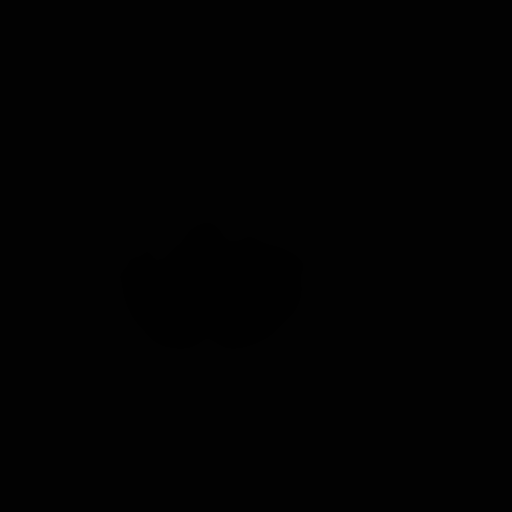

Supplement: S2 Data — (ZIP) [file pone.0295536.s003.zip › S3_Data/FCN_Training set_Label/IM_0004-ID_6debc00bd.png]

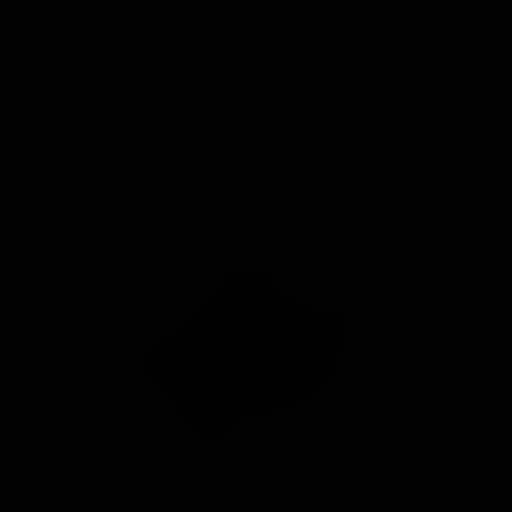

Supplement: S2 Data — (ZIP) [file pone.0295536.s003.zip › S3_Data/FCN_Training set_Label/IM_0004-ID_70aaf1bf4.png]

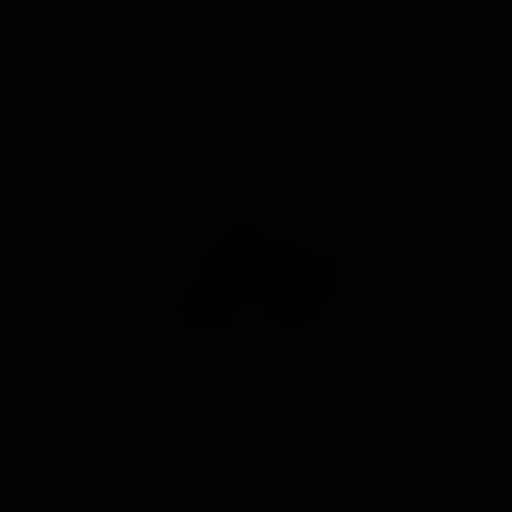

Supplement: S2 Data — (ZIP) [file pone.0295536.s003.zip › S3_Data/FCN_Training set_Label/IM_0004-ID_72720e428.png]

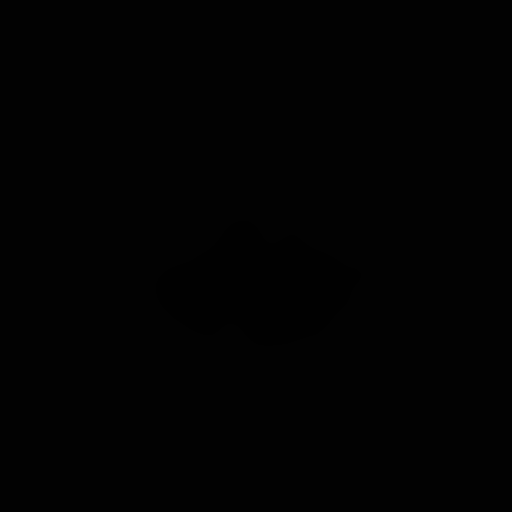

Supplement: S2 Data — (ZIP) [file pone.0295536.s003.zip › S3_Data/FCN_Training set_Label/IM_0004-ID_72e306345.png]

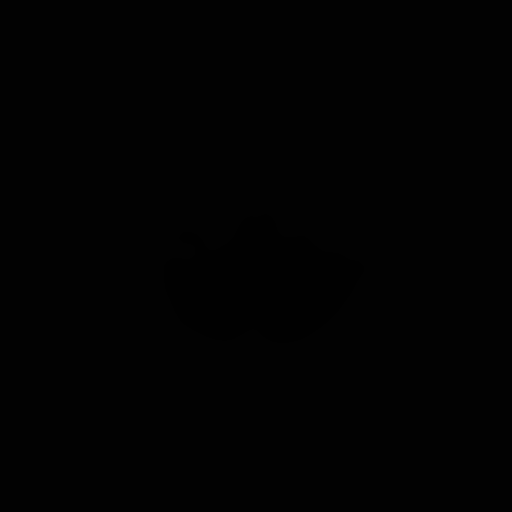

Supplement: S2 Data — (ZIP) [file pone.0295536.s003.zip › S3_Data/FCN_Training set_Label/IM_0004-ID_754129863.png]

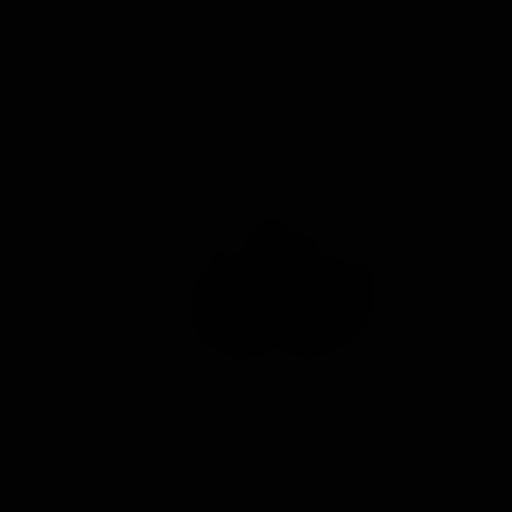

Supplement: S2 Data — (ZIP) [file pone.0295536.s003.zip › S3_Data/FCN_Training set_Label/IM_0004-ID_77d6a8a29.png]

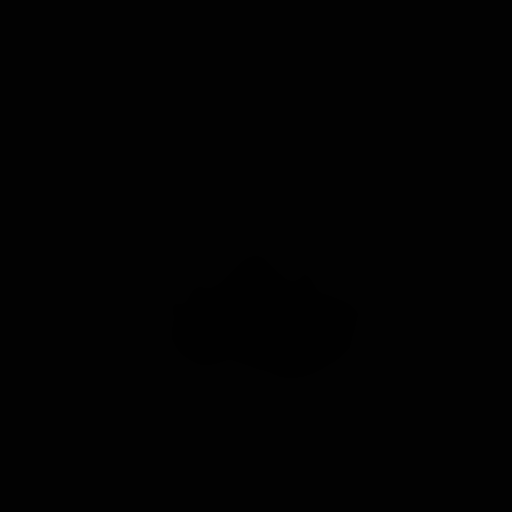

Supplement: S2 Data — (ZIP) [file pone.0295536.s003.zip › S3_Data/FCN_Training set_Label/IM_0004-ID_7c1145ff9.png]

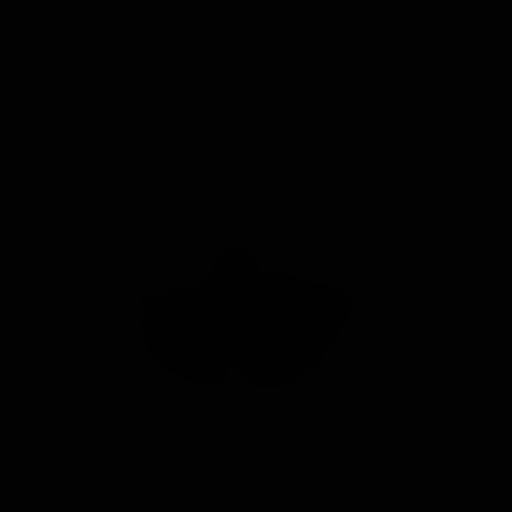

Supplement: S2 Data — (ZIP) [file pone.0295536.s003.zip › S3_Data/FCN_Training set_Label/IM_0004-ID_7d1fd19a3.png]

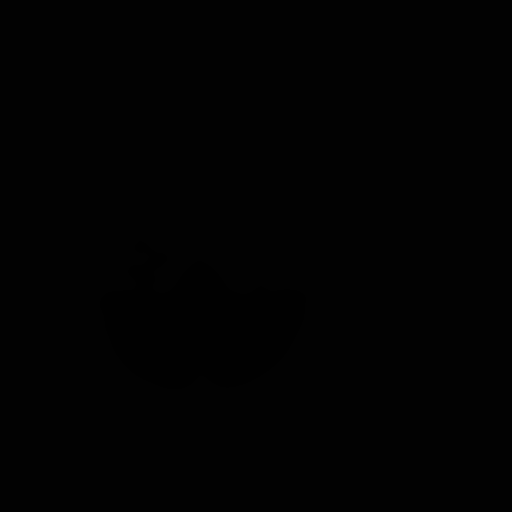

Supplement: S2 Data — (ZIP) [file pone.0295536.s003.zip › S3_Data/FCN_Training set_Label/IM_0004-ID_7d6878ba0.png]

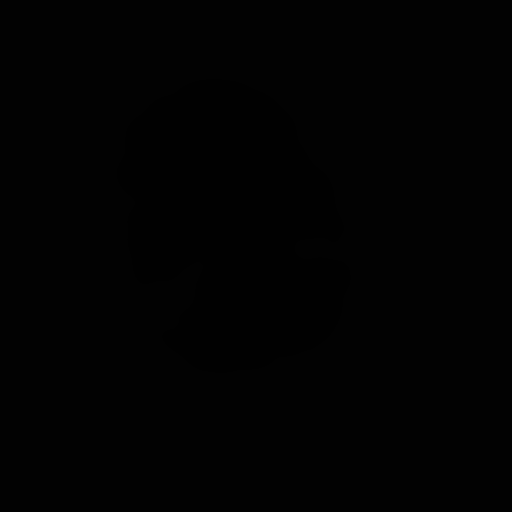

Supplement: S2 Data — (ZIP) [file pone.0295536.s003.zip › S3_Data/FCN_Training set_Label/IM_0004-ID_80eebdd30.png]

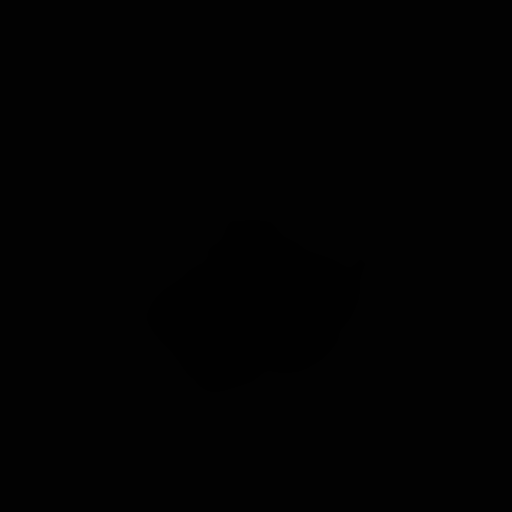

Supplement: S2 Data — (ZIP) [file pone.0295536.s003.zip › S3_Data/FCN_Training set_Label/IM_0004-ID_8280b9c5e.png]

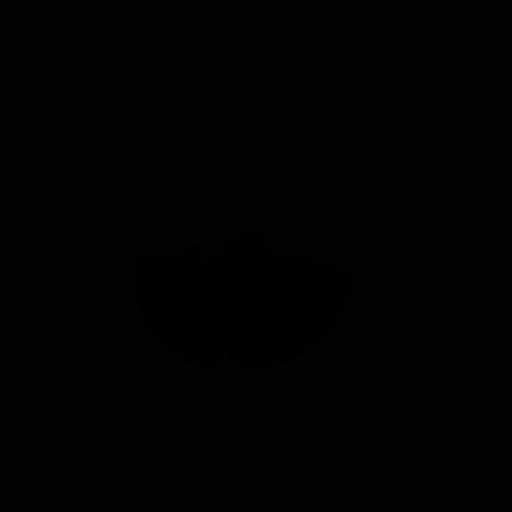

Supplement: S2 Data — (ZIP) [file pone.0295536.s003.zip › S3_Data/FCN_Training set_Label/IM_0004-ID_854dda944.png]

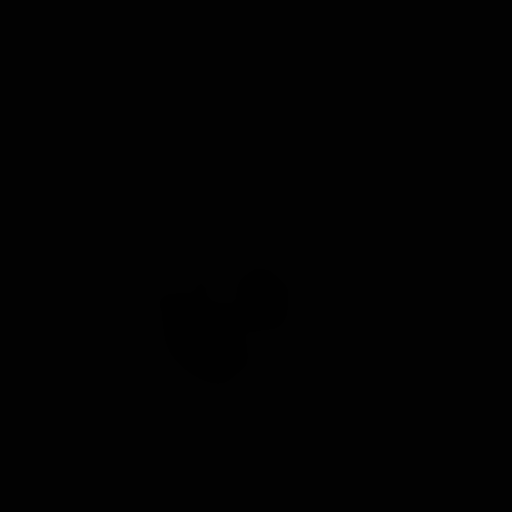

Supplement: S2 Data — (ZIP) [file pone.0295536.s003.zip › S3_Data/FCN_Training set_Label/IM_0004-ID_8b6f5a33a.png]

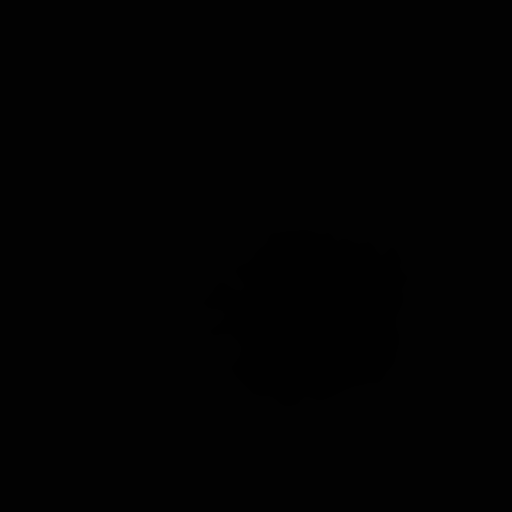

Supplement: S2 Data — (ZIP) [file pone.0295536.s003.zip › S3_Data/FCN_Training set_Label/IM_0004-ID_9e7a9f92d.png]

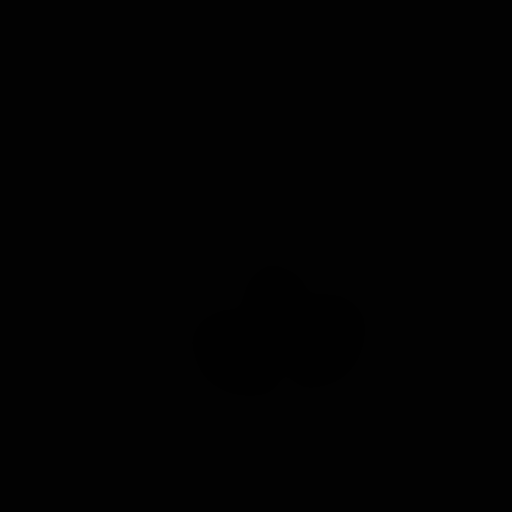

Supplement: S2 Data — (ZIP) [file pone.0295536.s003.zip › S3_Data/FCN_Training set_Label/IM_0004-ID_9fda6ef13.png]

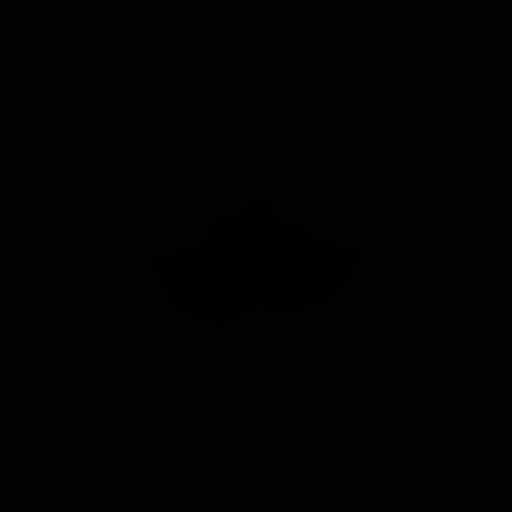

Supplement: S2 Data — (ZIP) [file pone.0295536.s003.zip › S3_Data/FCN_Training set_Label/IM_0004-ID_a1df019d1.png]

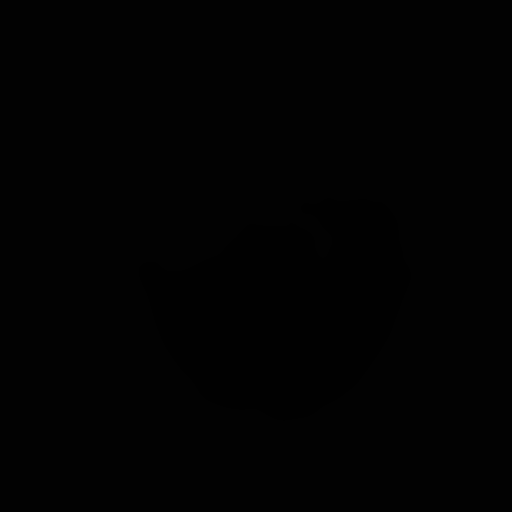

Supplement: S2 Data — (ZIP) [file pone.0295536.s003.zip › S3_Data/FCN_Training set_Label/IM_0004-ID_a5973ef74.png]

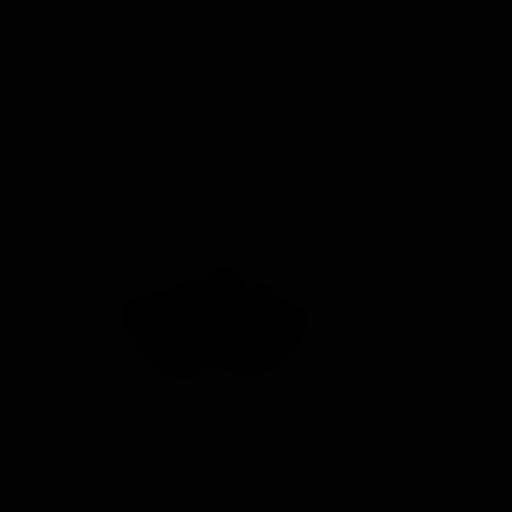

Supplement: S2 Data — (ZIP) [file pone.0295536.s003.zip › S3_Data/FCN_Training set_Label/IM_0004-ID_af90ee0a9.png]

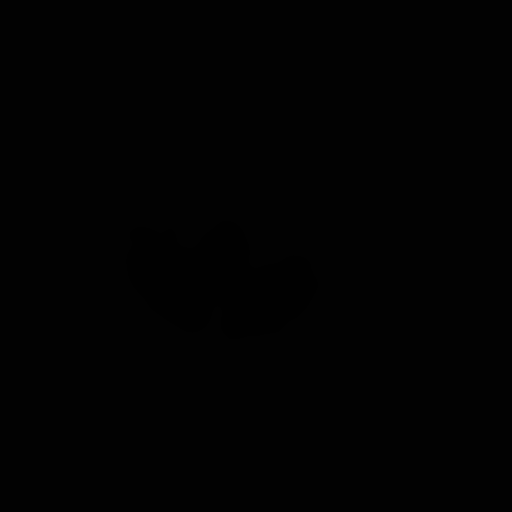

Supplement: S2 Data — (ZIP) [file pone.0295536.s003.zip › S3_Data/FCN_Training set_Label/IM_0004-ID_b4386c146.png]

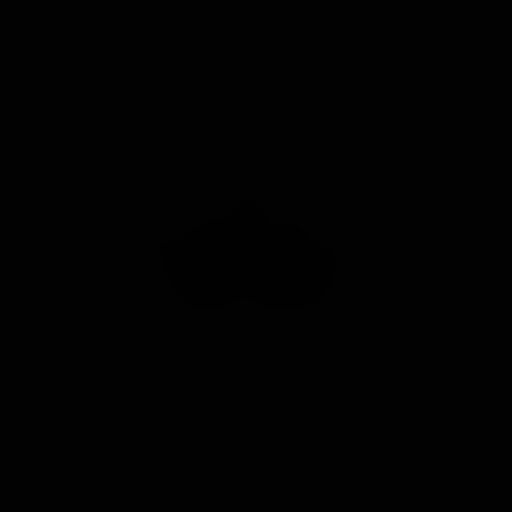

Supplement: S2 Data — (ZIP) [file pone.0295536.s003.zip › S3_Data/FCN_Training set_Label/IM_0004-ID_b530a9807.png]

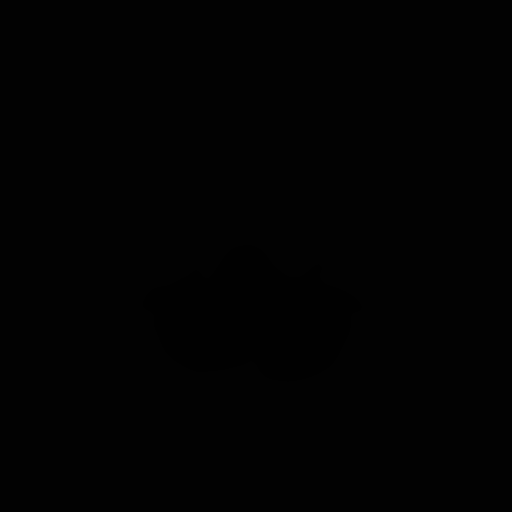

Supplement: S2 Data — (ZIP) [file pone.0295536.s003.zip › S3_Data/FCN_Training set_Label/IM_0004-ID_b94d2ef15.png]

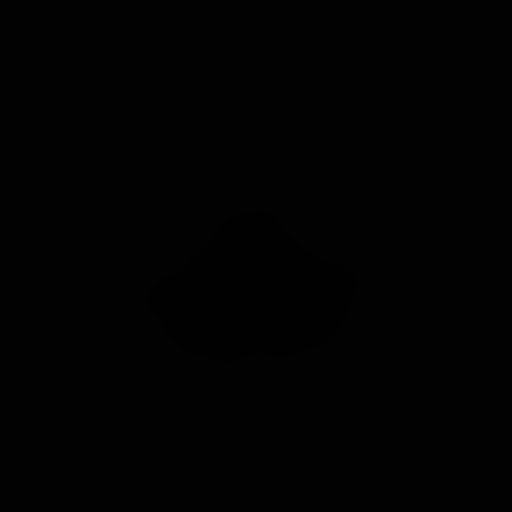

Supplement: S2 Data — (ZIP) [file pone.0295536.s003.zip › S3_Data/FCN_Training set_Label/IM_0004-ID_c05f1c333.png]

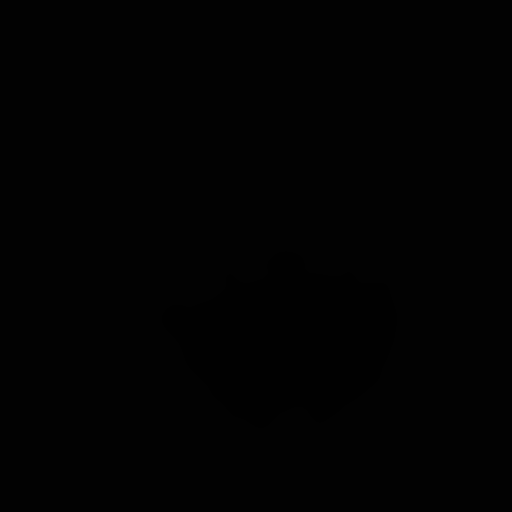

Supplement: S2 Data — (ZIP) [file pone.0295536.s003.zip › S3_Data/FCN_Training set_Label/IM_0004-ID_d12f46f3a.png]

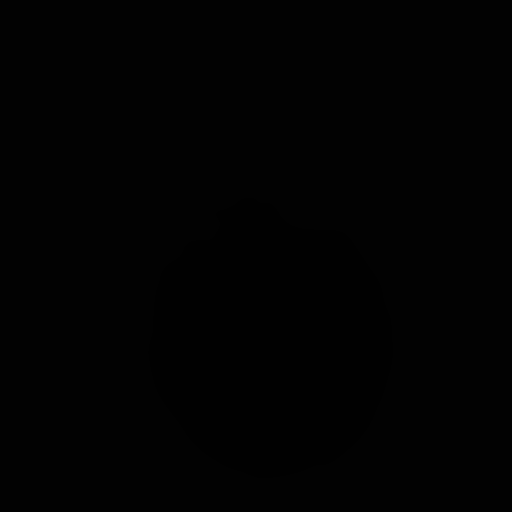

Supplement: S2 Data — (ZIP) [file pone.0295536.s003.zip › S3_Data/FCN_Training set_Label/IM_0004-ID_d1a3cb3e0.png]

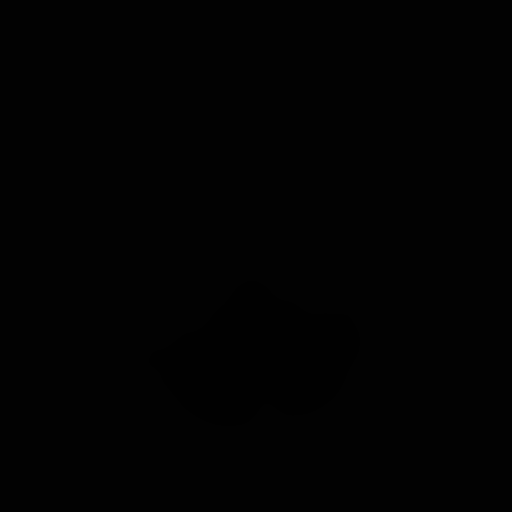

Supplement: S2 Data — (ZIP) [file pone.0295536.s003.zip › S3_Data/FCN_Training set_Label/IM_0004-ID_d4def7e5d.png]

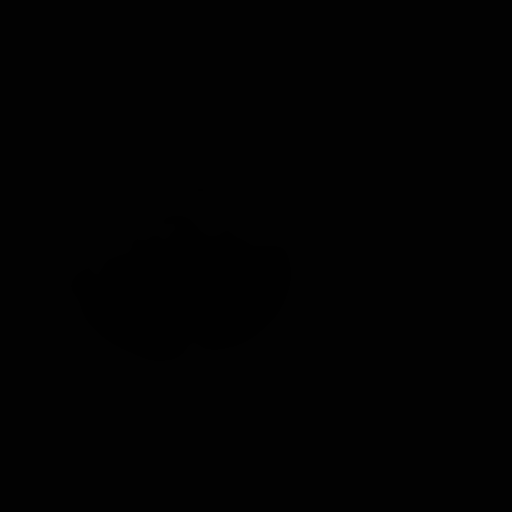

Supplement: S2 Data — (ZIP) [file pone.0295536.s003.zip › S3_Data/FCN_Training set_Label/IM_0004-ID_d942ab733.png]

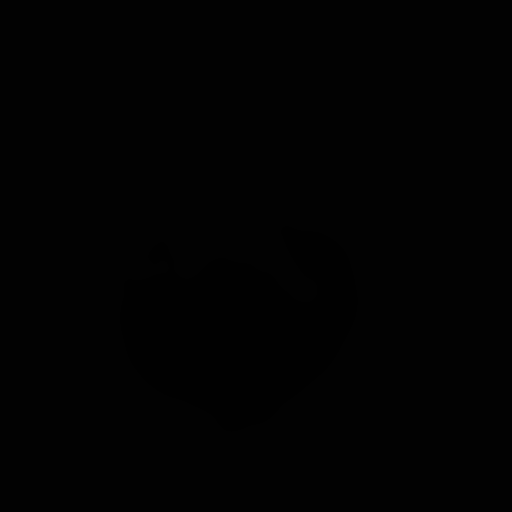

Supplement: S2 Data — (ZIP) [file pone.0295536.s003.zip › S3_Data/FCN_Training set_Label/IM_0004-ID_ede968118.png]

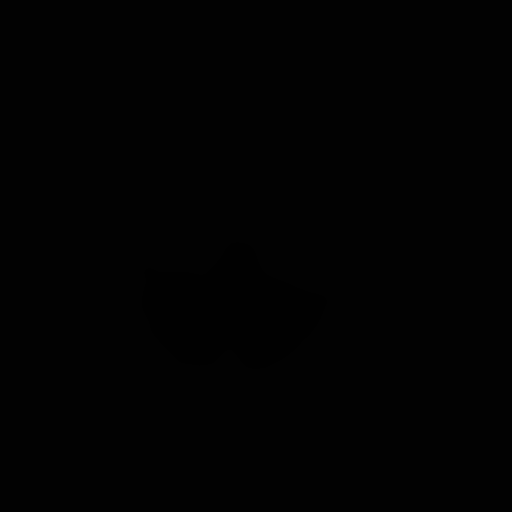

Supplement: S2 Data — (ZIP) [file pone.0295536.s003.zip › S3_Data/FCN_Training set_Label/IM_0004-ID_f75f50ed1.png]

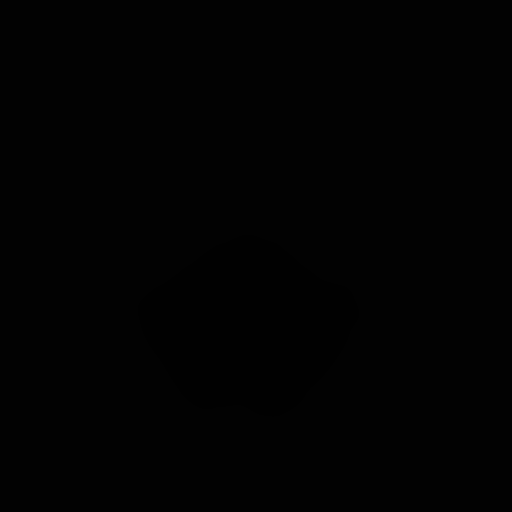

Supplement: S2 Data — (ZIP) [file pone.0295536.s003.zip › S3_Data/FCN_Training set_Label/IM_0005-ID_00c000a81.png]

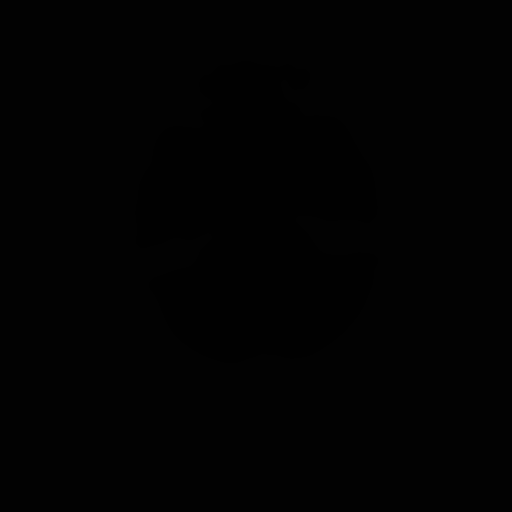

Supplement: S2 Data — (ZIP) [file pone.0295536.s003.zip › S3_Data/FCN_Training set_Label/IM_0005-ID_0a3dc59a9.png]

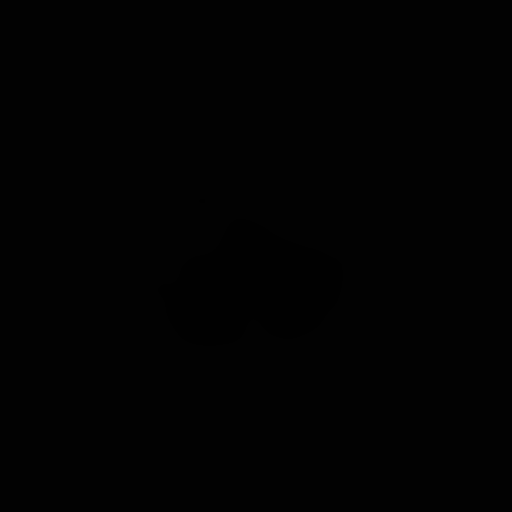

Supplement: S2 Data — (ZIP) [file pone.0295536.s003.zip › S3_Data/FCN_Training set_Label/IM_0005-ID_0c957218a.png]

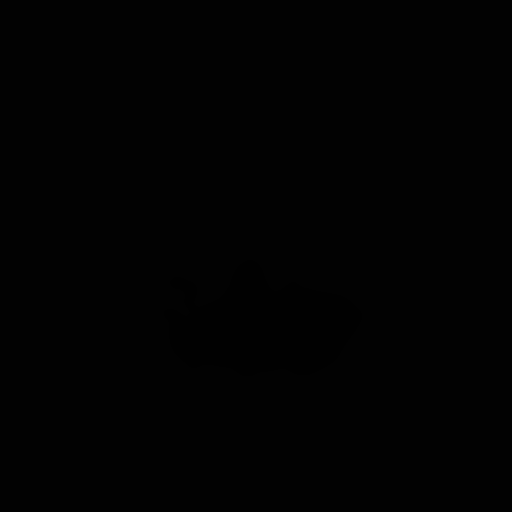

Supplement: S2 Data — (ZIP) [file pone.0295536.s003.zip › S3_Data/FCN_Training set_Label/IM_0005-ID_0dc60d342.png]

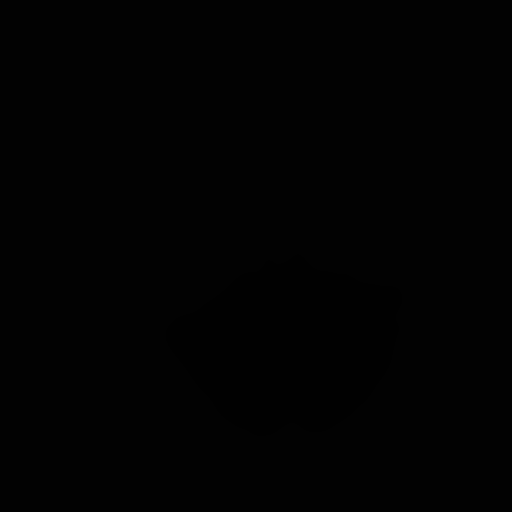

Supplement: S2 Data — (ZIP) [file pone.0295536.s003.zip › S3_Data/FCN_Training set_Label/IM_0005-ID_15196fcfa.png]

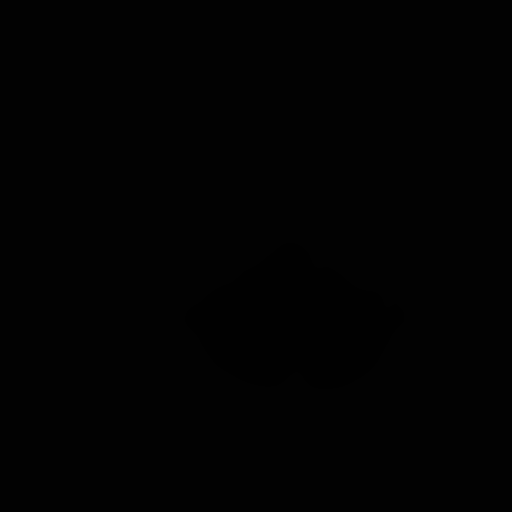

Supplement: S2 Data — (ZIP) [file pone.0295536.s003.zip › S3_Data/FCN_Training set_Label/IM_0005-ID_1847ec2b4.png]

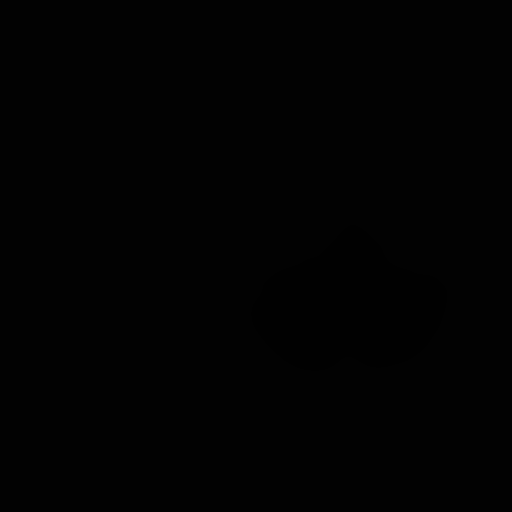

Supplement: S2 Data — (ZIP) [file pone.0295536.s003.zip › S3_Data/FCN_Training set_Label/IM_0005-ID_1d467c5c2.png]

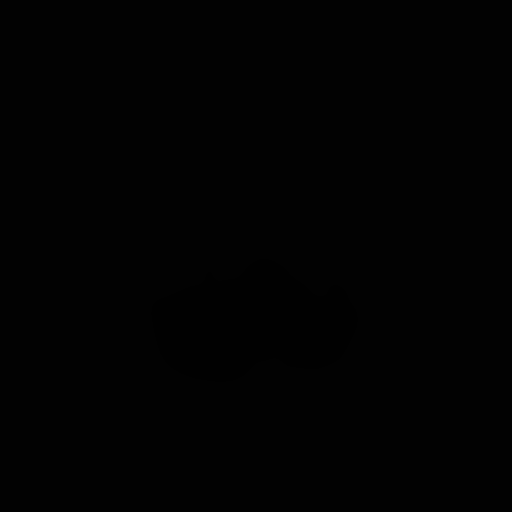

Supplement: S2 Data — (ZIP) [file pone.0295536.s003.zip › S3_Data/FCN_Training set_Label/IM_0005-ID_1e5802dfa.png]

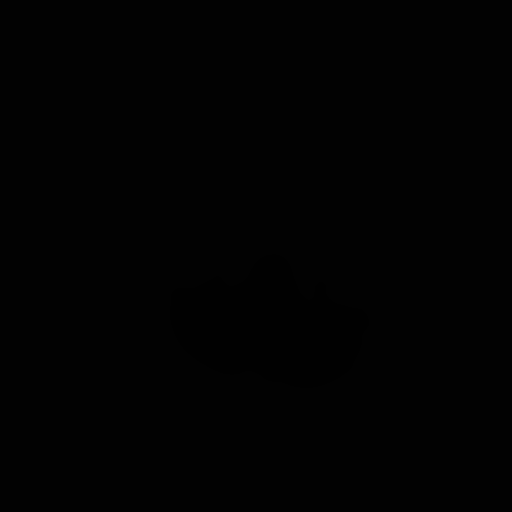

Supplement: S2 Data — (ZIP) [file pone.0295536.s003.zip › S3_Data/FCN_Training set_Label/IM_0005-ID_1f8f228e5.png]

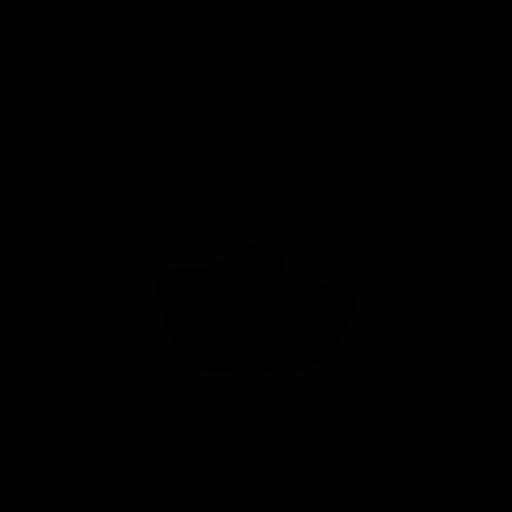

Supplement: S2 Data — (ZIP) [file pone.0295536.s003.zip › S3_Data/FCN_Training set_Label/IM_0005-ID_246168f91.png]

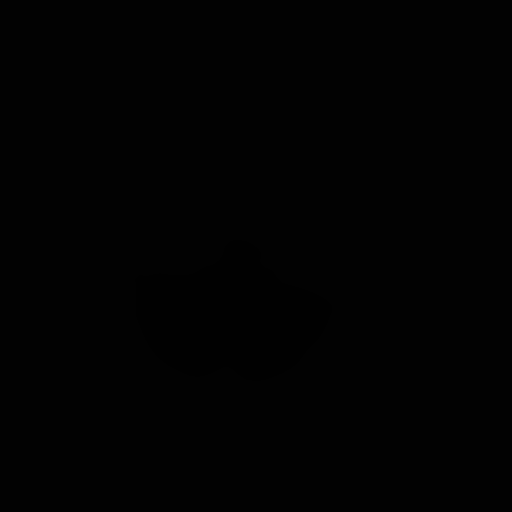

Supplement: S2 Data — (ZIP) [file pone.0295536.s003.zip › S3_Data/FCN_Training set_Label/IM_0005-ID_2c37ed251.png]

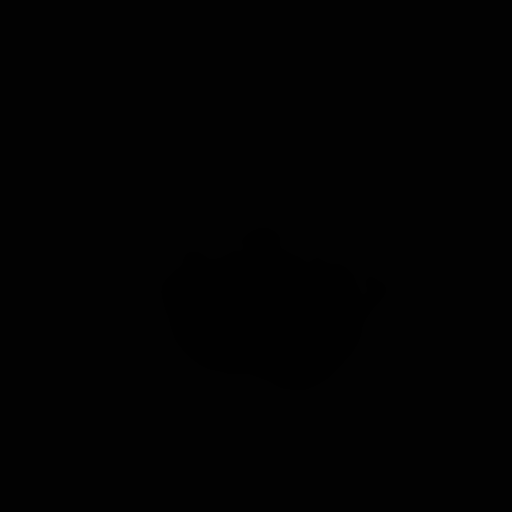

Supplement: S2 Data — (ZIP) [file pone.0295536.s003.zip › S3_Data/FCN_Training set_Label/IM_0005-ID_2ca4d0cb1.png]

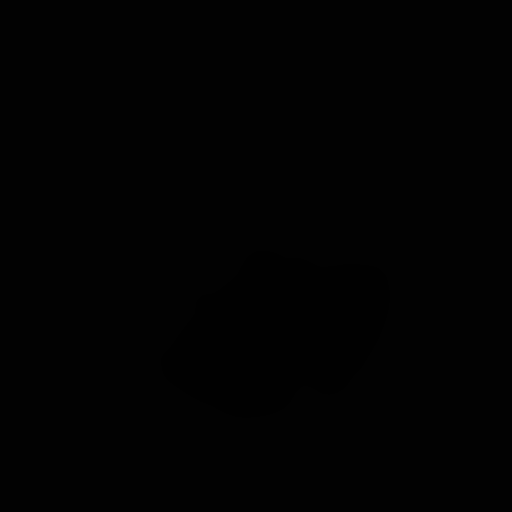

Supplement: S2 Data — (ZIP) [file pone.0295536.s003.zip › S3_Data/FCN_Training set_Label/IM_0005-ID_2f96fefac.png]

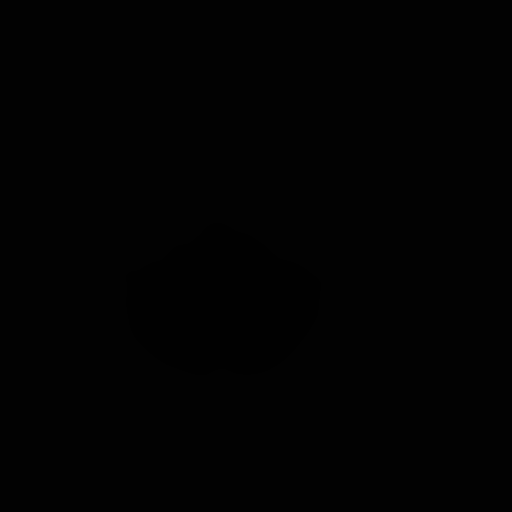

Supplement: S2 Data — (ZIP) [file pone.0295536.s003.zip › S3_Data/FCN_Training set_Label/IM_0005-ID_39938271b.png]

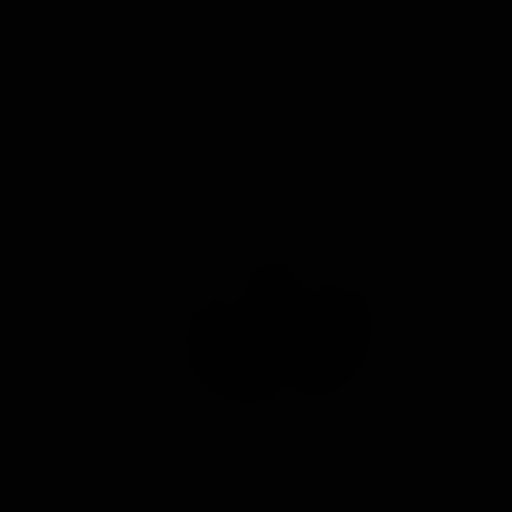

Supplement: S2 Data — (ZIP) [file pone.0295536.s003.zip › S3_Data/FCN_Training set_Label/IM_0005-ID_3a57d214c.png]

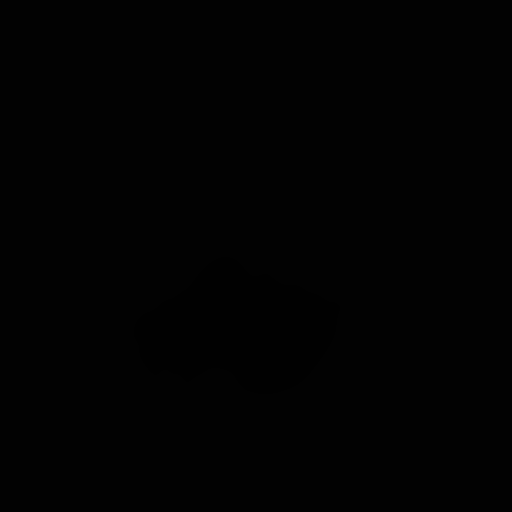

Supplement: S2 Data — (ZIP) [file pone.0295536.s003.zip › S3_Data/FCN_Training set_Label/IM_0005-ID_3e860d214.png]

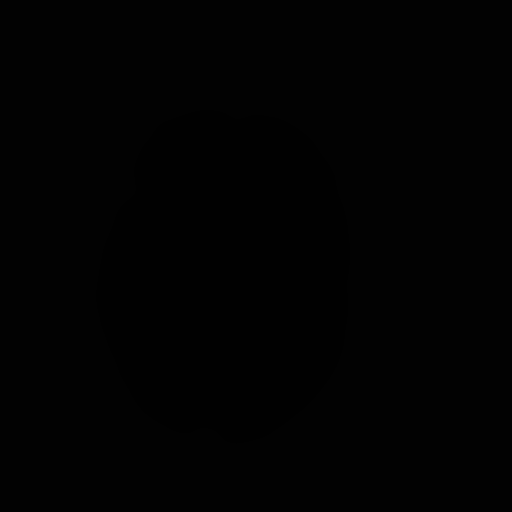

Supplement: S2 Data — (ZIP) [file pone.0295536.s003.zip › S3_Data/FCN_Training set_Label/IM_0005-ID_45b493897.png]

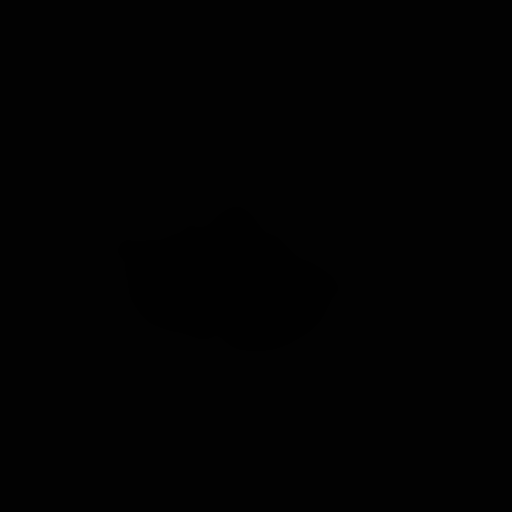

Supplement: S2 Data — (ZIP) [file pone.0295536.s003.zip › S3_Data/FCN_Training set_Label/IM_0005-ID_4a22483bb.png]

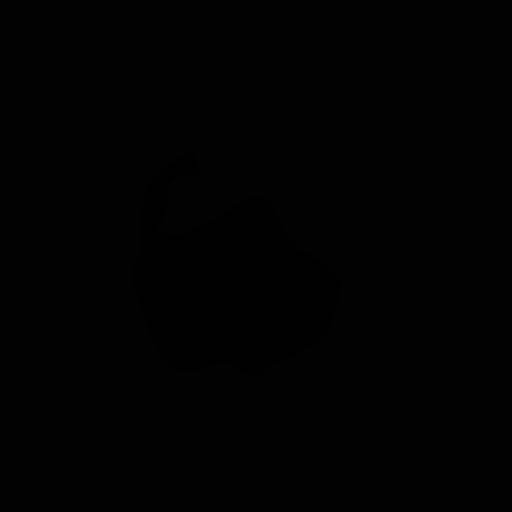

Supplement: S2 Data — (ZIP) [file pone.0295536.s003.zip › S3_Data/FCN_Training set_Label/IM_0005-ID_4ba455613.png]

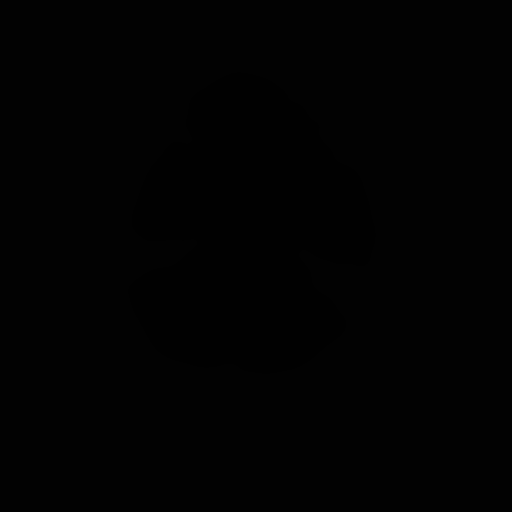

Supplement: S2 Data — (ZIP) [file pone.0295536.s003.zip › S3_Data/FCN_Training set_Label/IM_0005-ID_510f93c45.png]

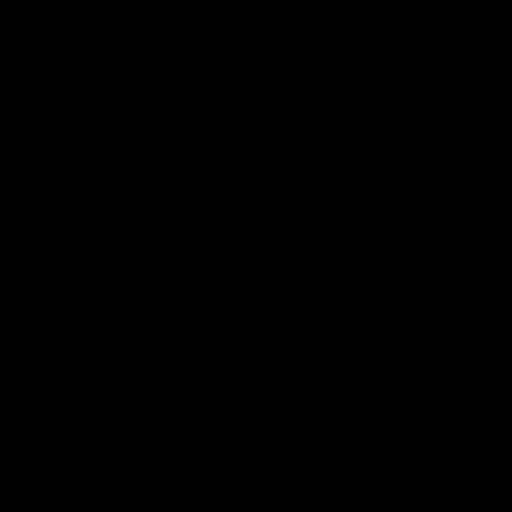

Supplement: S2 Data — (ZIP) [file pone.0295536.s003.zip › S3_Data/FCN_Training set_Label/IM_0005-ID_556833e85.png]

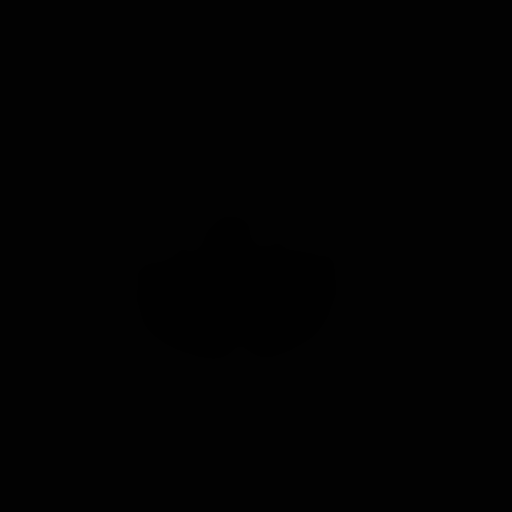

Supplement: S2 Data — (ZIP) [file pone.0295536.s003.zip › S3_Data/FCN_Training set_Label/IM_0005-ID_55fd2b447.png]
