# Supplementary figures and images for: Adaptive mask-based brain extraction method for head CT images (part 8 of 14)
Source: PLoS One. 2024 Mar 11;19(3):e0295536. doi: 10.1371/journal.pone.0295536 (PMC10927156; doi:10.1371/journal.pone.0295536)

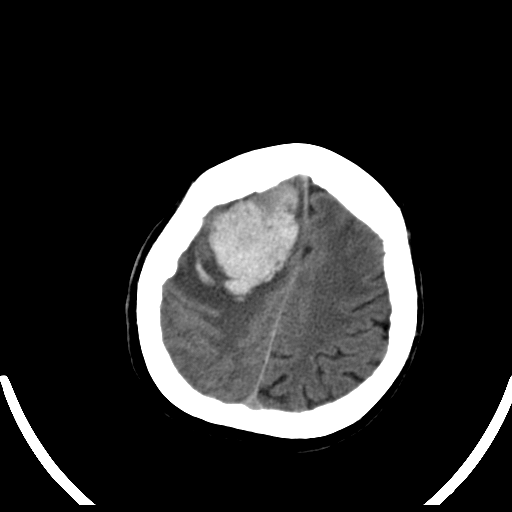

Supplement: S4 Data — (ZIP) [file pone.0295536.s005.zip › S5_Data/FCN_Training set/IM_0016-ID_86805b3f9.png]

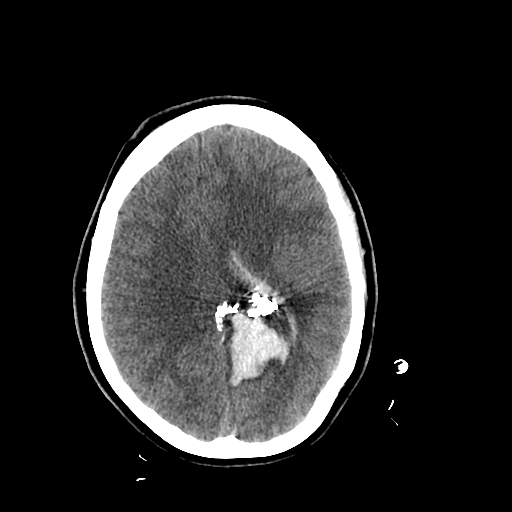

Supplement: S4 Data — (ZIP) [file pone.0295536.s005.zip › S5_Data/FCN_Training set/IM_0016-ID_a218fc52c.png]

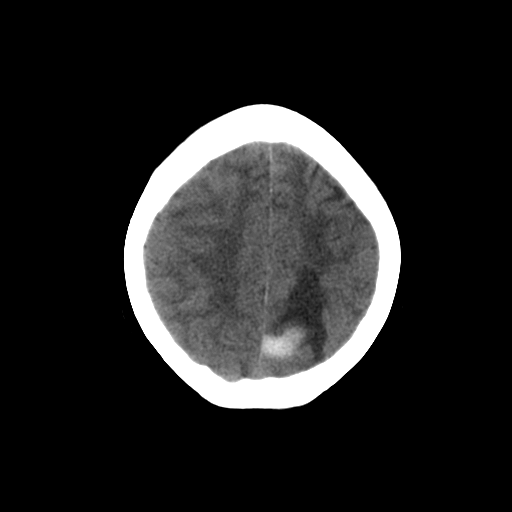

Supplement: S4 Data — (ZIP) [file pone.0295536.s005.zip › S5_Data/FCN_Training set/IM_0016-ID_ab5c1dfaf.png]

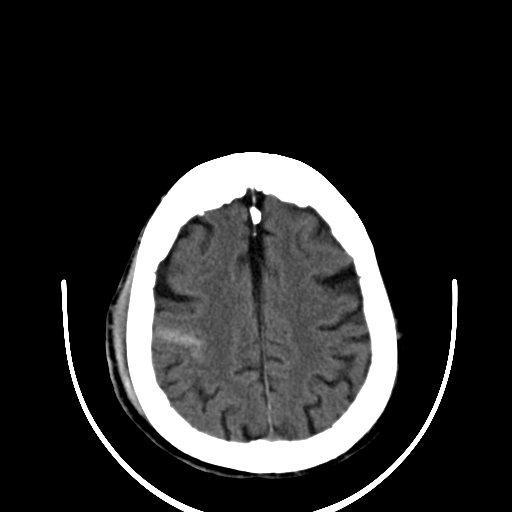

Supplement: S4 Data — (ZIP) [file pone.0295536.s005.zip › S5_Data/FCN_Training set/IM_0016-ID_b3a44dca9.png]

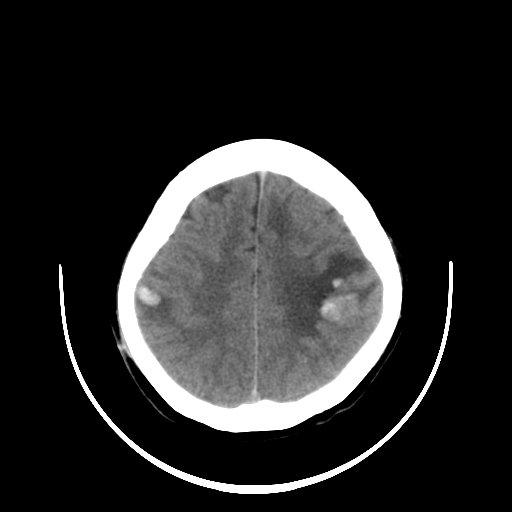

Supplement: S4 Data — (ZIP) [file pone.0295536.s005.zip › S5_Data/FCN_Training set/IM_0016-ID_b864c6107.png]

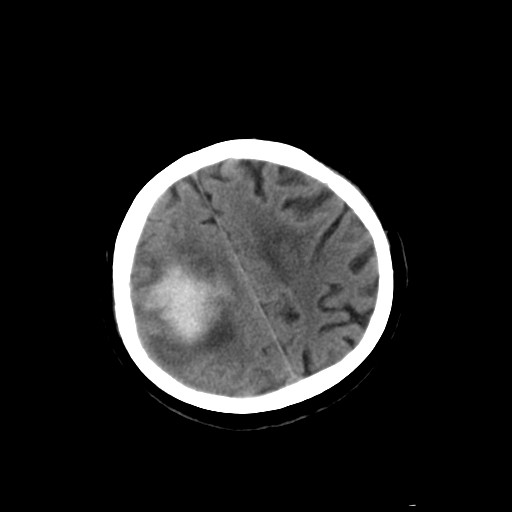

Supplement: S4 Data — (ZIP) [file pone.0295536.s005.zip › S5_Data/FCN_Training set/IM_0016-ID_bcf52d204.png]

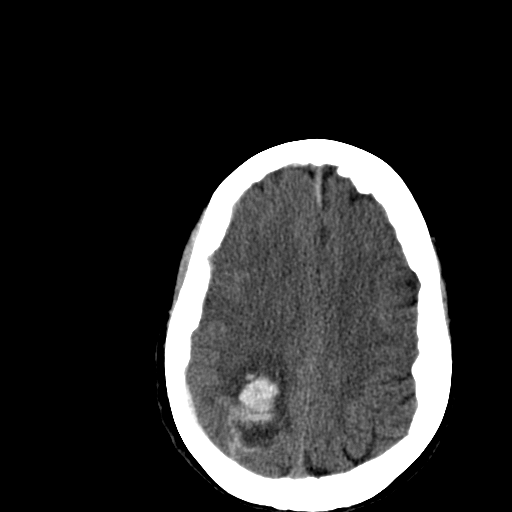

Supplement: S4 Data — (ZIP) [file pone.0295536.s005.zip › S5_Data/FCN_Training set/IM_0016-ID_f0aec0c7c.png]

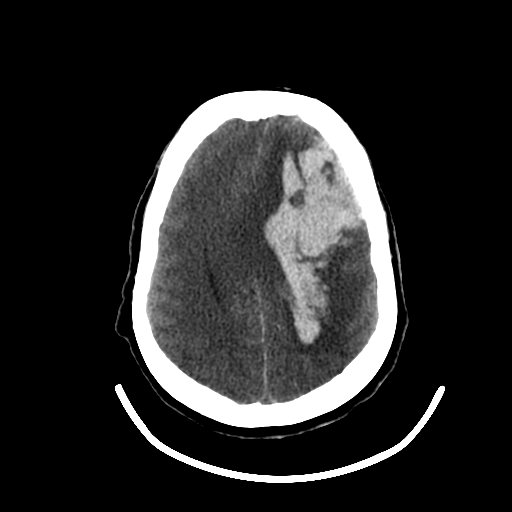

Supplement: S4 Data — (ZIP) [file pone.0295536.s005.zip › S5_Data/FCN_Training set/IM_0016-ID_f3704fffd.png]

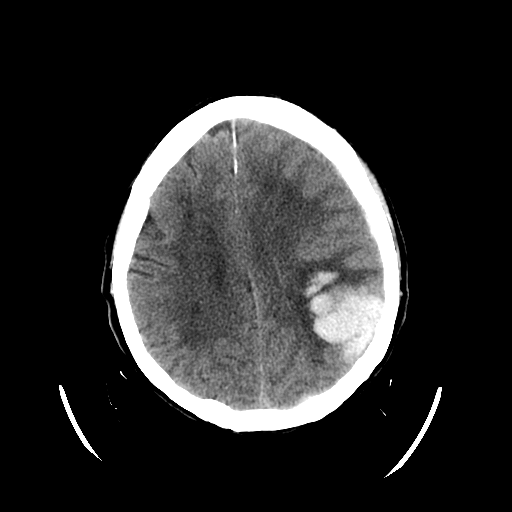

Supplement: S4 Data — (ZIP) [file pone.0295536.s005.zip › S5_Data/FCN_Training set/IM_0016-ID_ffe36e8e2.png]

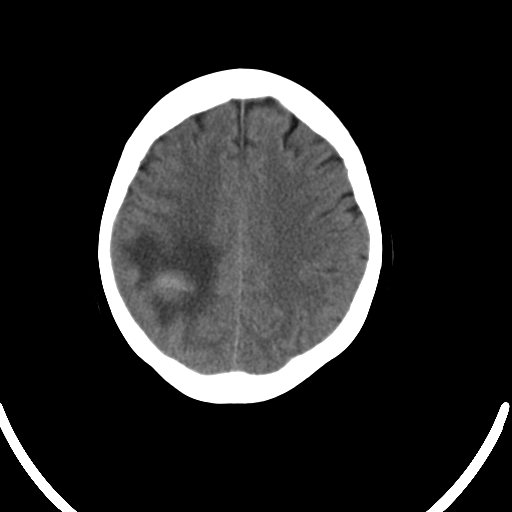

Supplement: S4 Data — (ZIP) [file pone.0295536.s005.zip › S5_Data/FCN_Training set/IM_0017-ID_068128d45.png]

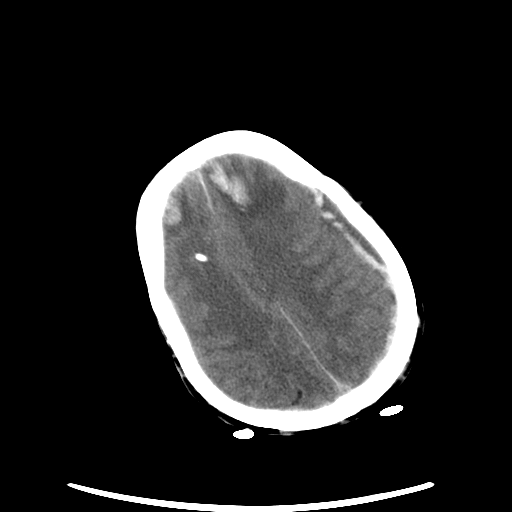

Supplement: S4 Data — (ZIP) [file pone.0295536.s005.zip › S5_Data/FCN_Training set/IM_0017-ID_098fc057d.png]

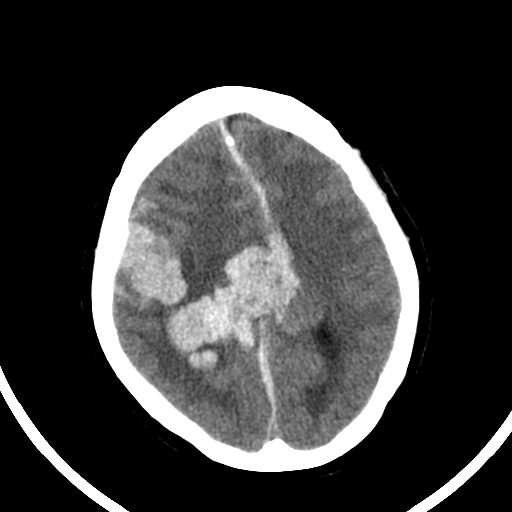

Supplement: S4 Data — (ZIP) [file pone.0295536.s005.zip › S5_Data/FCN_Training set/IM_0017-ID_0aec1f3a4.png]

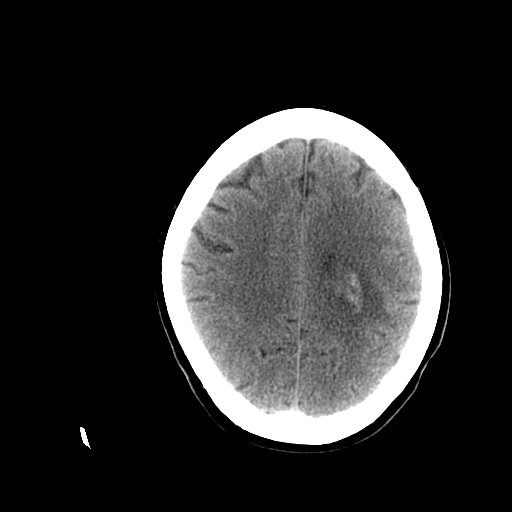

Supplement: S4 Data — (ZIP) [file pone.0295536.s005.zip › S5_Data/FCN_Training set/IM_0017-ID_0bce7830a.png]

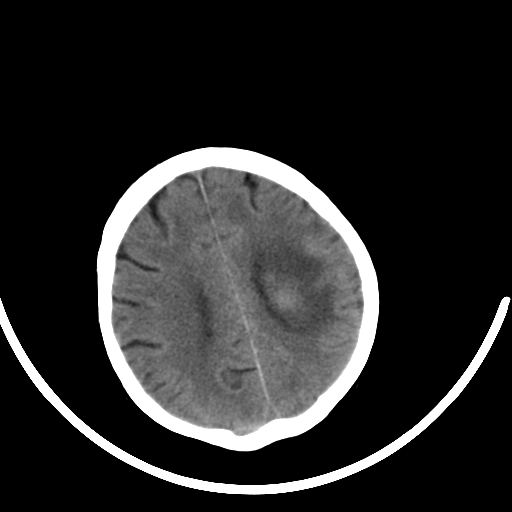

Supplement: S4 Data — (ZIP) [file pone.0295536.s005.zip › S5_Data/FCN_Training set/IM_0017-ID_132859dc8.png]

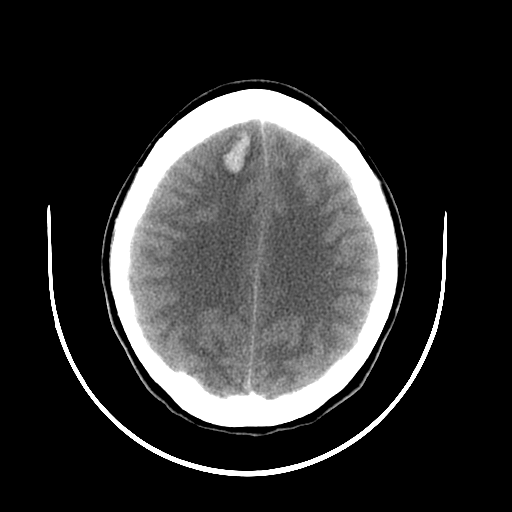

Supplement: S4 Data — (ZIP) [file pone.0295536.s005.zip › S5_Data/FCN_Training set/IM_0017-ID_155759de0.png]

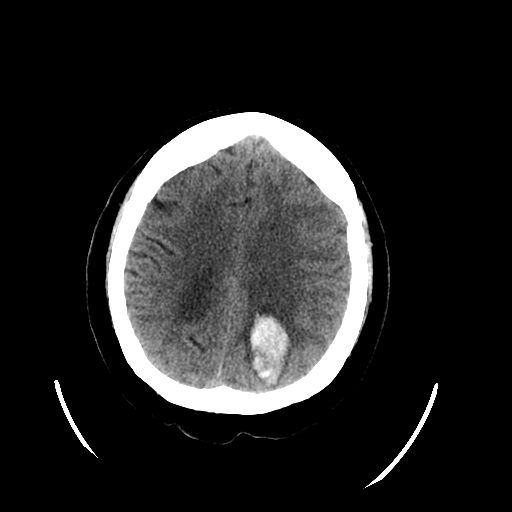

Supplement: S4 Data — (ZIP) [file pone.0295536.s005.zip › S5_Data/FCN_Training set/IM_0017-ID_1b75364ce.png]

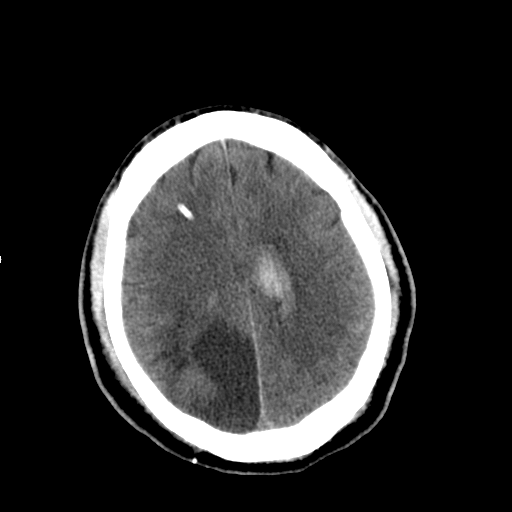

Supplement: S4 Data — (ZIP) [file pone.0295536.s005.zip › S5_Data/FCN_Training set/IM_0017-ID_1cfad2bcb.png]

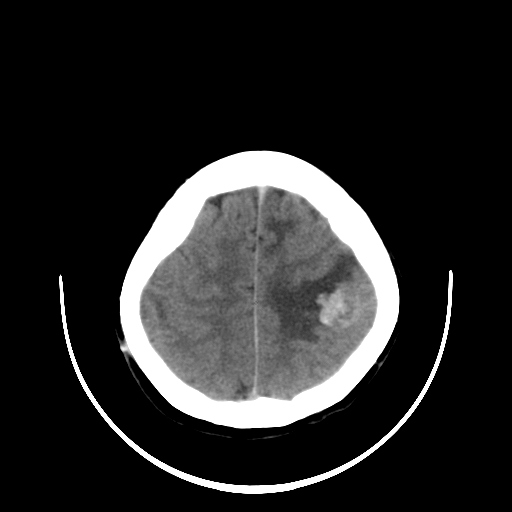

Supplement: S4 Data — (ZIP) [file pone.0295536.s005.zip › S5_Data/FCN_Training set/IM_0017-ID_1e1e0ecfa.png]

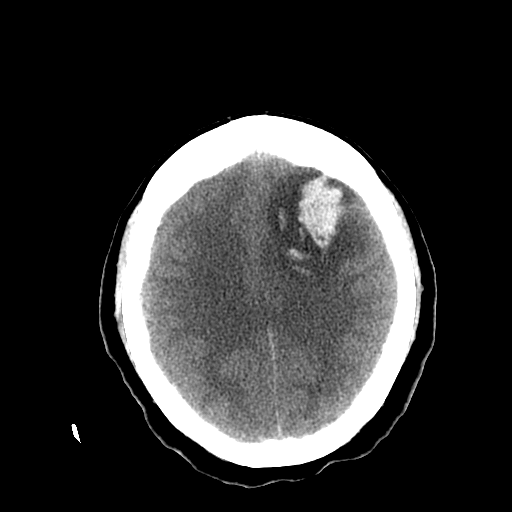

Supplement: S4 Data — (ZIP) [file pone.0295536.s005.zip › S5_Data/FCN_Training set/IM_0017-ID_209cde4eb.png]

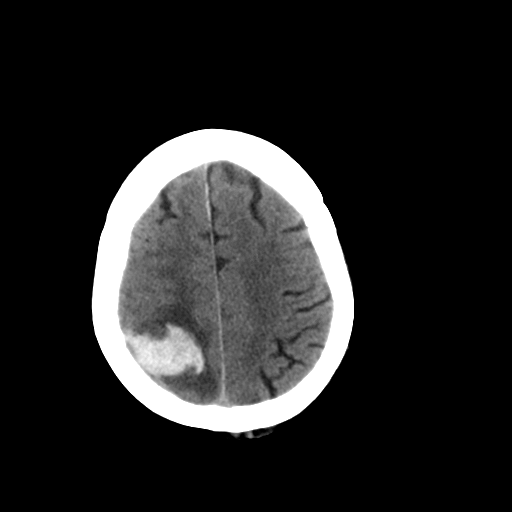

Supplement: S4 Data — (ZIP) [file pone.0295536.s005.zip › S5_Data/FCN_Training set/IM_0017-ID_210e652af.png]

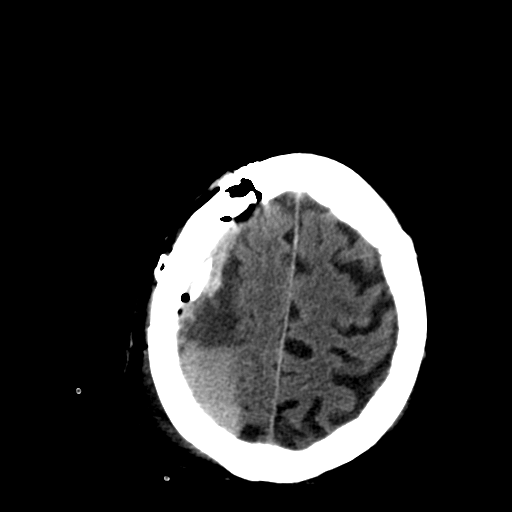

Supplement: S4 Data — (ZIP) [file pone.0295536.s005.zip › S5_Data/FCN_Training set/IM_0017-ID_23afeb89a.png]

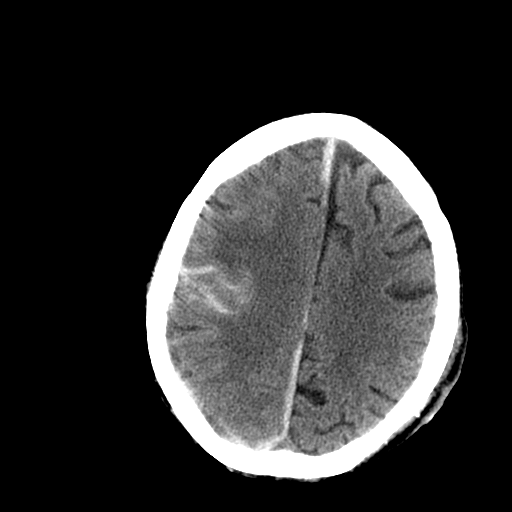

Supplement: S4 Data — (ZIP) [file pone.0295536.s005.zip › S5_Data/FCN_Training set/IM_0017-ID_252b891d3.png]

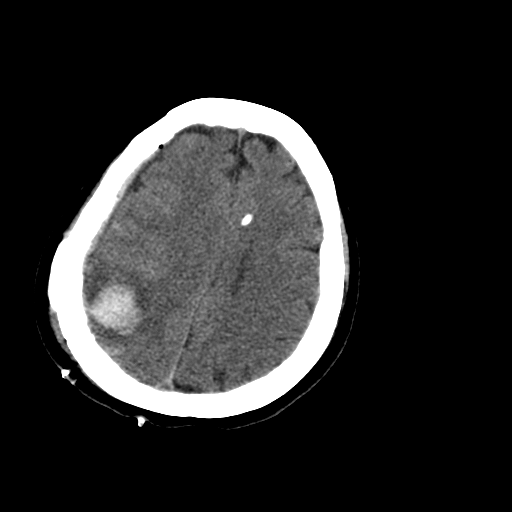

Supplement: S4 Data — (ZIP) [file pone.0295536.s005.zip › S5_Data/FCN_Training set/IM_0017-ID_2555d1736.png]

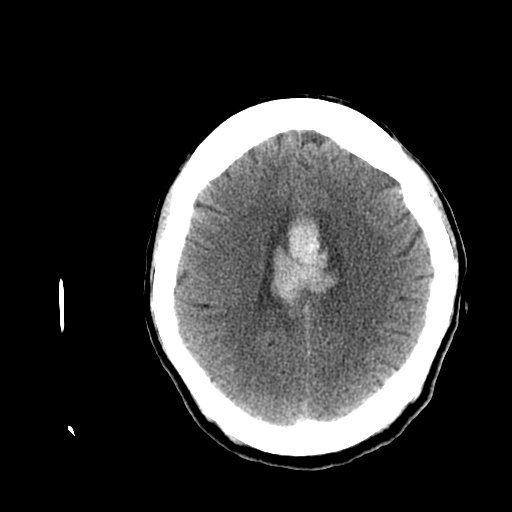

Supplement: S4 Data — (ZIP) [file pone.0295536.s005.zip › S5_Data/FCN_Training set/IM_0017-ID_2572c106b.png]

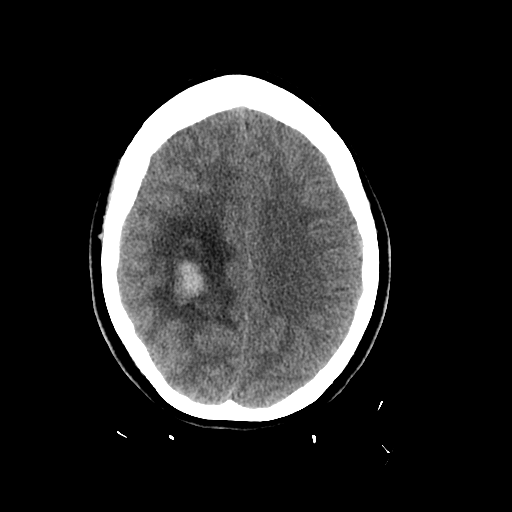

Supplement: S4 Data — (ZIP) [file pone.0295536.s005.zip › S5_Data/FCN_Training set/IM_0017-ID_29355c0c1.png]

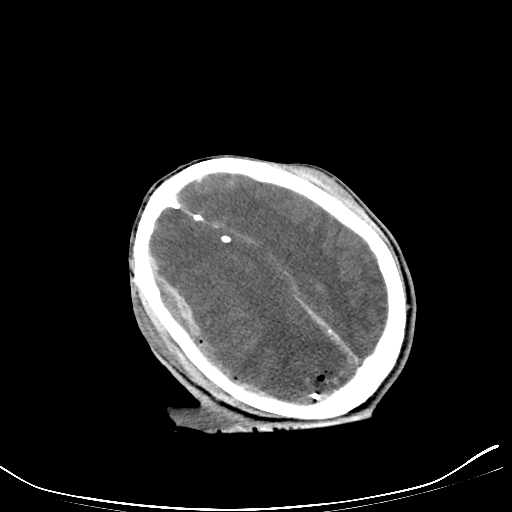

Supplement: S4 Data — (ZIP) [file pone.0295536.s005.zip › S5_Data/FCN_Training set/IM_0017-ID_299fb53e7.png]

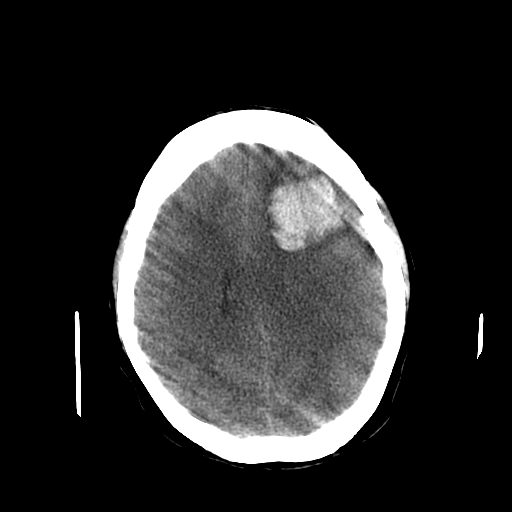

Supplement: S4 Data — (ZIP) [file pone.0295536.s005.zip › S5_Data/FCN_Training set/IM_0017-ID_2c9f7bbc7.png]

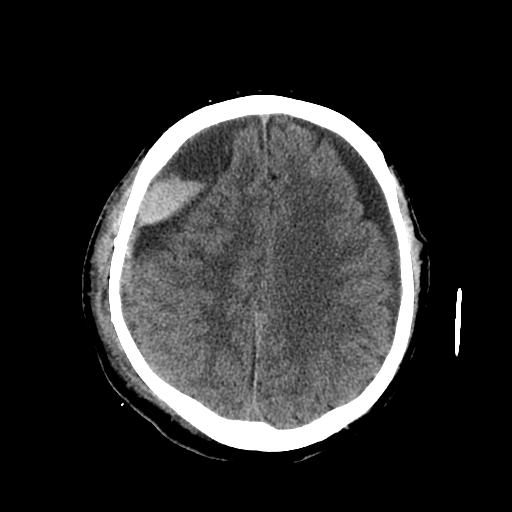

Supplement: S4 Data — (ZIP) [file pone.0295536.s005.zip › S5_Data/FCN_Training set/IM_0017-ID_2d1b3204c.png]

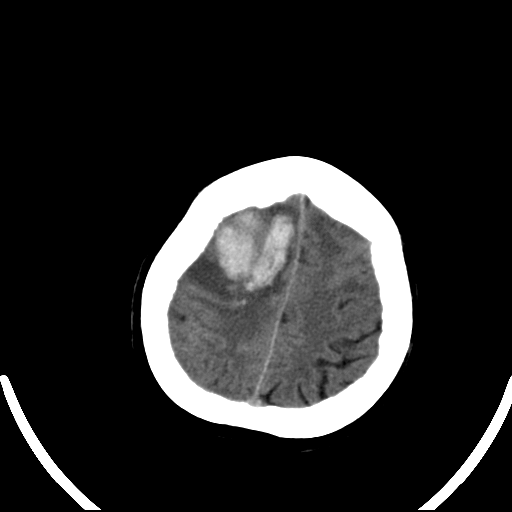

Supplement: S4 Data — (ZIP) [file pone.0295536.s005.zip › S5_Data/FCN_Training set/IM_0017-ID_2d3103029.png]

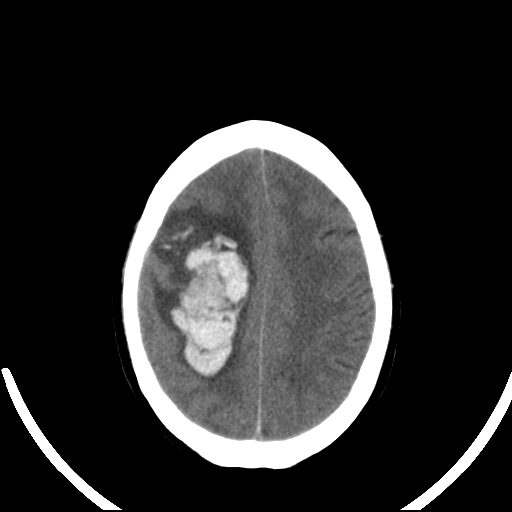

Supplement: S4 Data — (ZIP) [file pone.0295536.s005.zip › S5_Data/FCN_Training set/IM_0017-ID_2fc596c38.png]

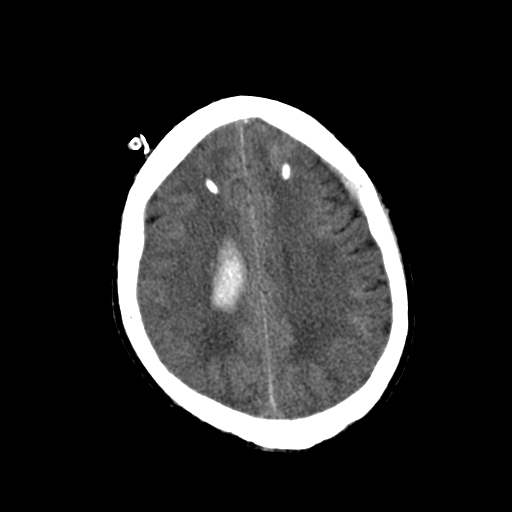

Supplement: S4 Data — (ZIP) [file pone.0295536.s005.zip › S5_Data/FCN_Training set/IM_0017-ID_3852563c6.png]

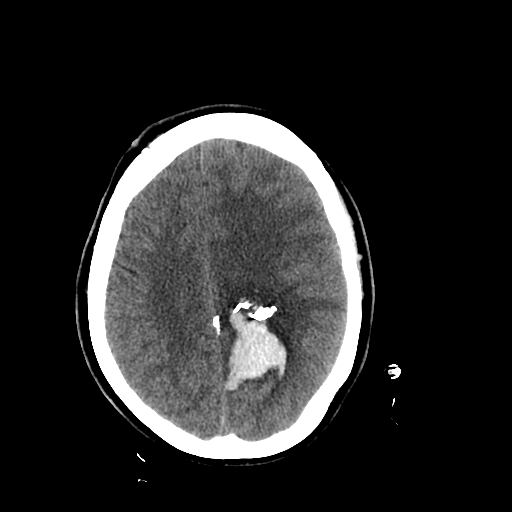

Supplement: S4 Data — (ZIP) [file pone.0295536.s005.zip › S5_Data/FCN_Training set/IM_0017-ID_3959df455.png]

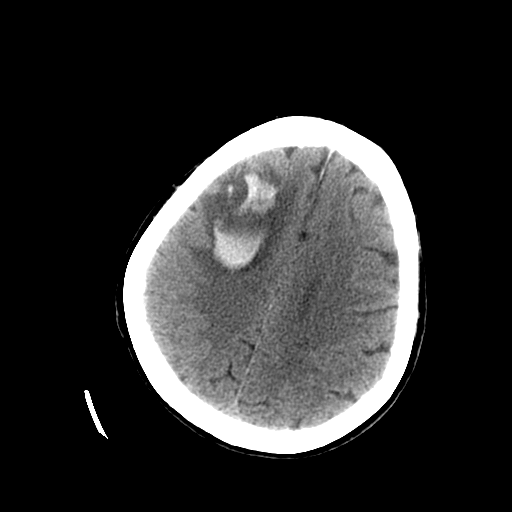

Supplement: S4 Data — (ZIP) [file pone.0295536.s005.zip › S5_Data/FCN_Training set/IM_0017-ID_40645762e.png]

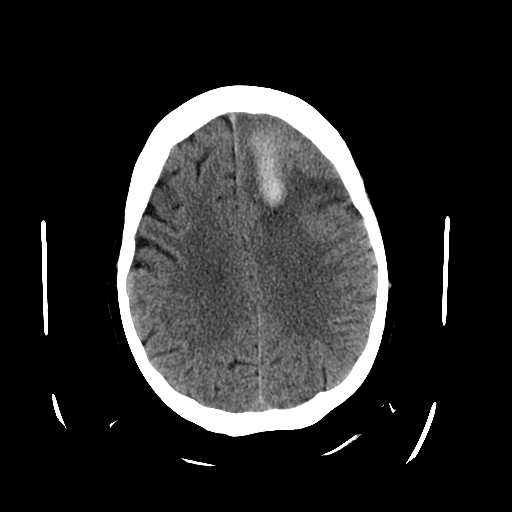

Supplement: S4 Data — (ZIP) [file pone.0295536.s005.zip › S5_Data/FCN_Training set/IM_0017-ID_4ba2aa069.png]

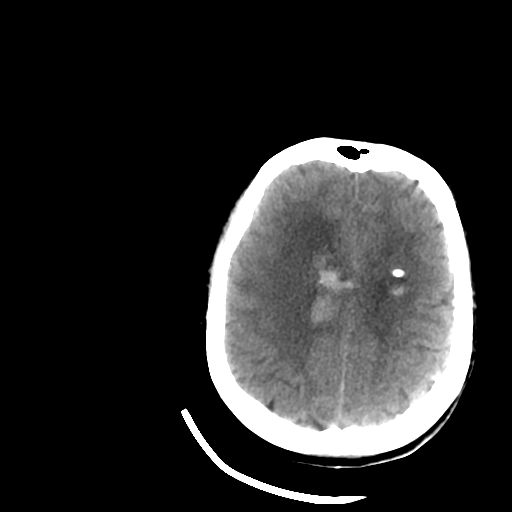

Supplement: S4 Data — (ZIP) [file pone.0295536.s005.zip › S5_Data/FCN_Training set/IM_0017-ID_50ab0d0ea.png]

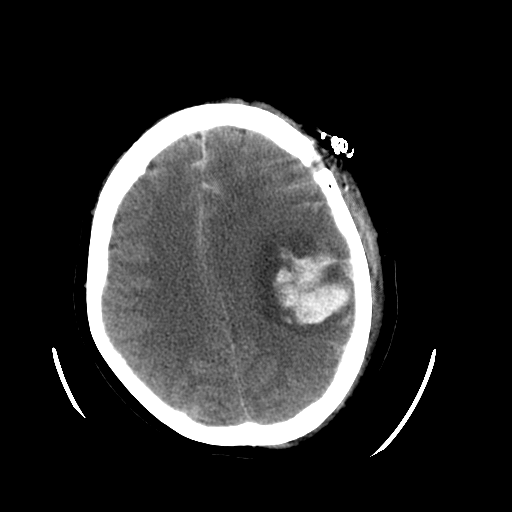

Supplement: S4 Data — (ZIP) [file pone.0295536.s005.zip › S5_Data/FCN_Training set/IM_0017-ID_57ddba268.png]

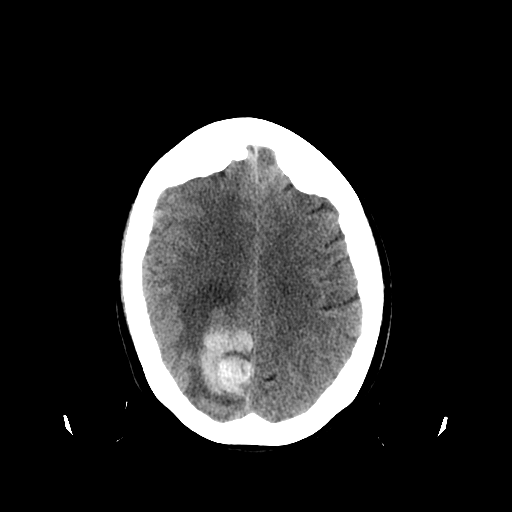

Supplement: S4 Data — (ZIP) [file pone.0295536.s005.zip › S5_Data/FCN_Training set/IM_0017-ID_599bc9d96.png]

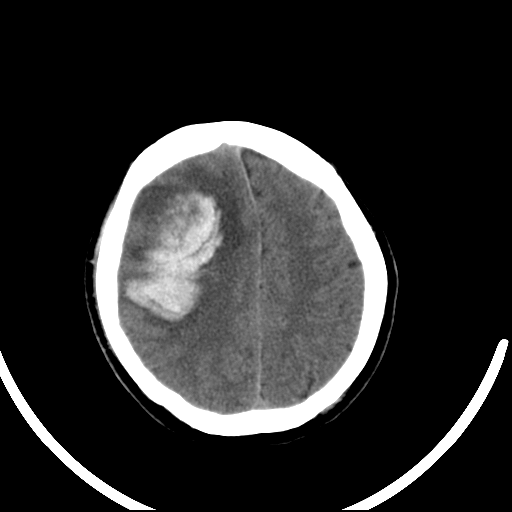

Supplement: S4 Data — (ZIP) [file pone.0295536.s005.zip › S5_Data/FCN_Training set/IM_0017-ID_5b4750542.png]

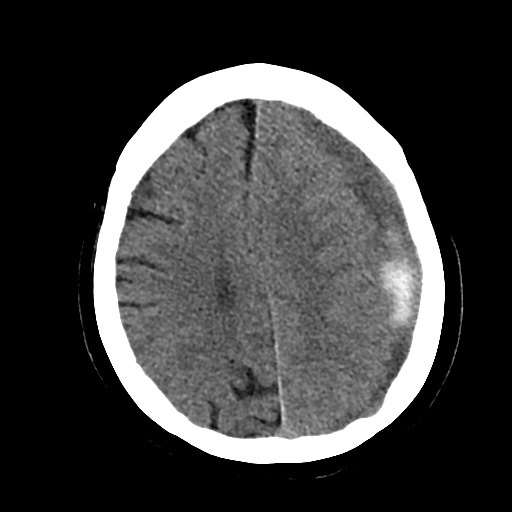

Supplement: S4 Data — (ZIP) [file pone.0295536.s005.zip › S5_Data/FCN_Training set/IM_0017-ID_5d70118db.png]

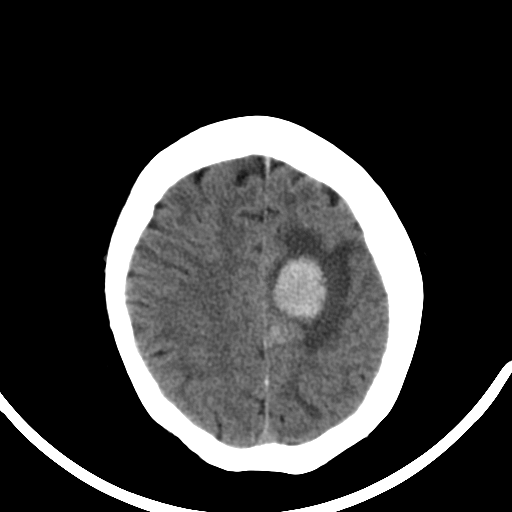

Supplement: S4 Data — (ZIP) [file pone.0295536.s005.zip › S5_Data/FCN_Training set/IM_0017-ID_5ed3e5b04.png]

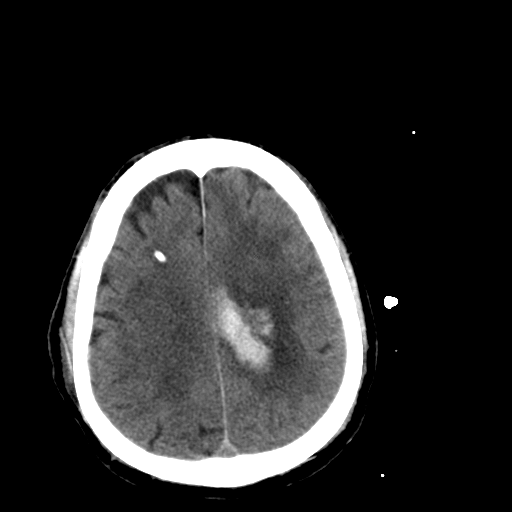

Supplement: S4 Data — (ZIP) [file pone.0295536.s005.zip › S5_Data/FCN_Training set/IM_0017-ID_5fd586573.png]

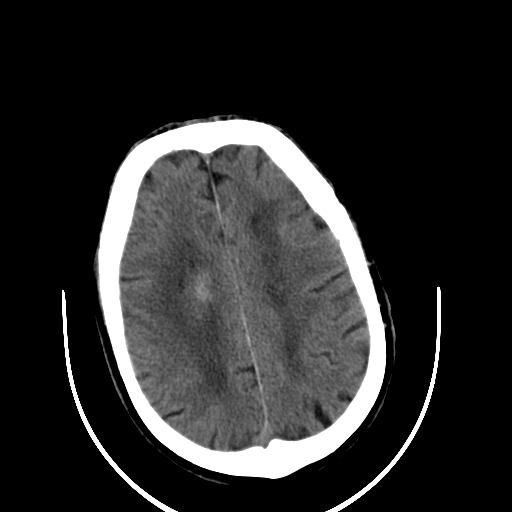

Supplement: S4 Data — (ZIP) [file pone.0295536.s005.zip › S5_Data/FCN_Training set/IM_0017-ID_6415ddc8e.png]

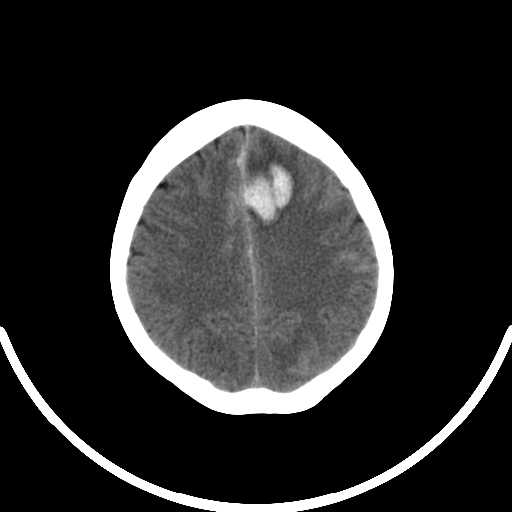

Supplement: S4 Data — (ZIP) [file pone.0295536.s005.zip › S5_Data/FCN_Training set/IM_0017-ID_6598e5b61.png]

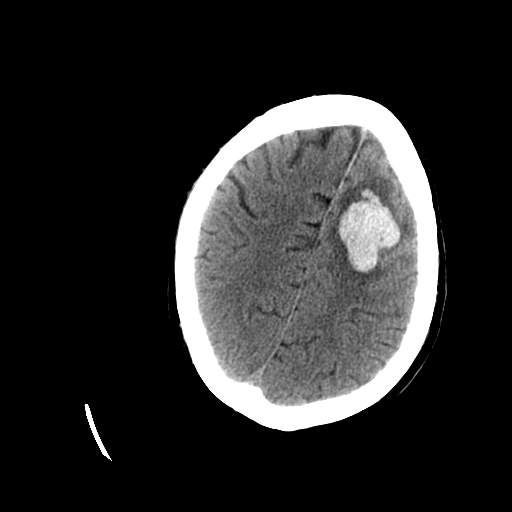

Supplement: S4 Data — (ZIP) [file pone.0295536.s005.zip › S5_Data/FCN_Training set/IM_0017-ID_65d7dda39.png]

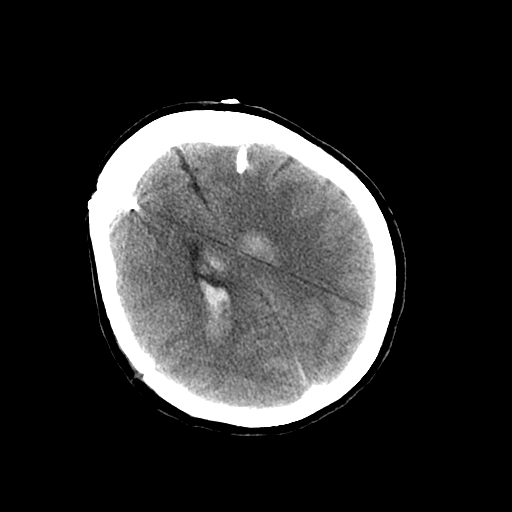

Supplement: S4 Data — (ZIP) [file pone.0295536.s005.zip › S5_Data/FCN_Training set/IM_0017-ID_702962f22.png]

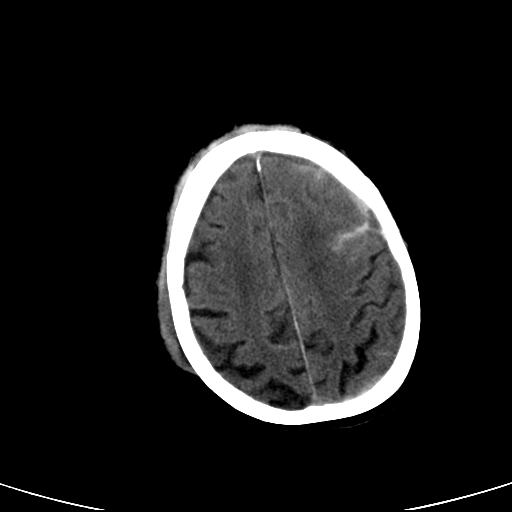

Supplement: S4 Data — (ZIP) [file pone.0295536.s005.zip › S5_Data/FCN_Training set/IM_0017-ID_7082407c0.png]

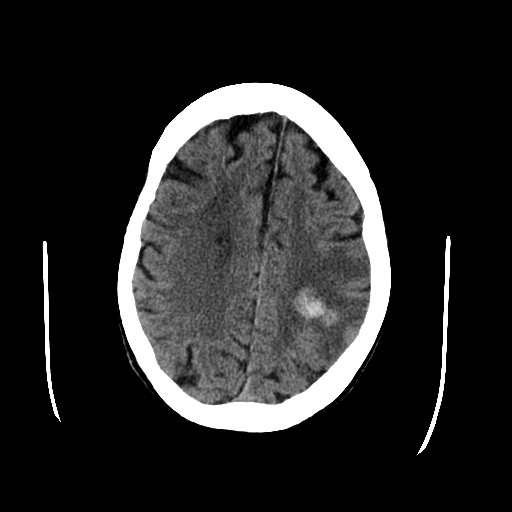

Supplement: S4 Data — (ZIP) [file pone.0295536.s005.zip › S5_Data/FCN_Training set/IM_0017-ID_7da3002f3.png]

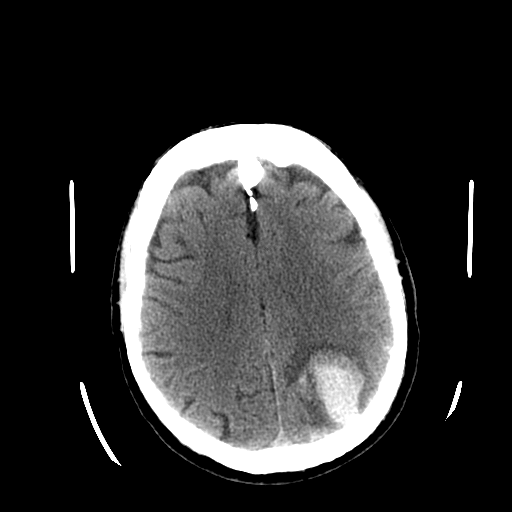

Supplement: S4 Data — (ZIP) [file pone.0295536.s005.zip › S5_Data/FCN_Training set/IM_0017-ID_80823861b.png]

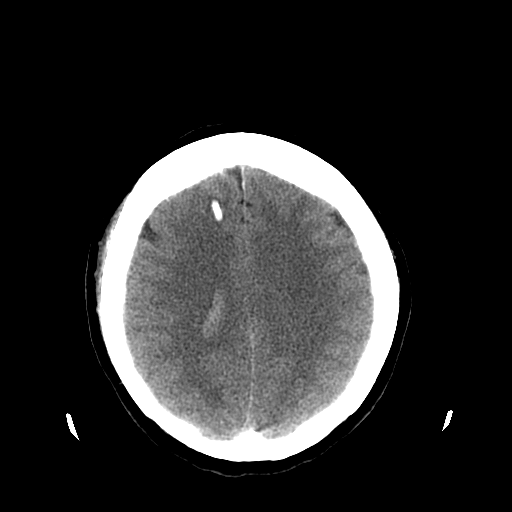

Supplement: S4 Data — (ZIP) [file pone.0295536.s005.zip › S5_Data/FCN_Training set/IM_0017-ID_84afb0859.png]

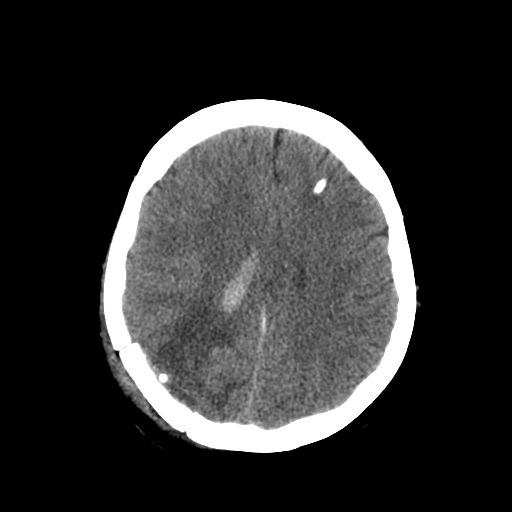

Supplement: S4 Data — (ZIP) [file pone.0295536.s005.zip › S5_Data/FCN_Training set/IM_0017-ID_86ee8c2bf.png]

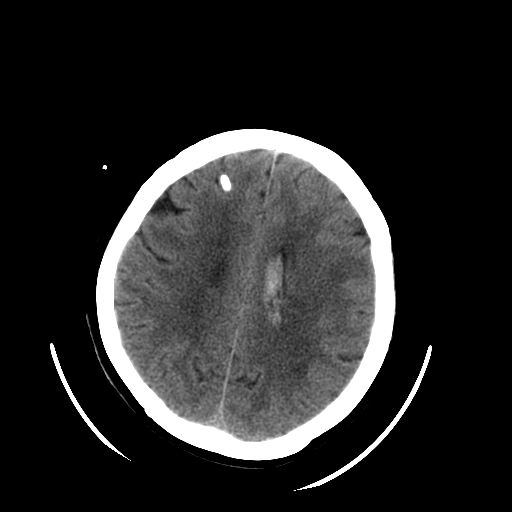

Supplement: S4 Data — (ZIP) [file pone.0295536.s005.zip › S5_Data/FCN_Training set/IM_0017-ID_885515589.png]

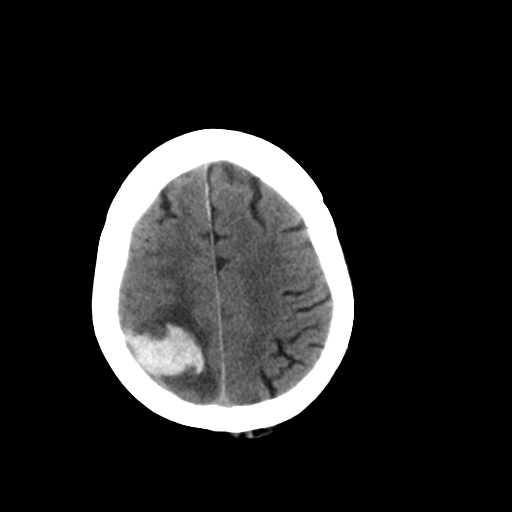

Supplement: S4 Data — (ZIP) [file pone.0295536.s005.zip › S5_Data/FCN_Training set/IM_0017-ID_8bb8c3db9.png]

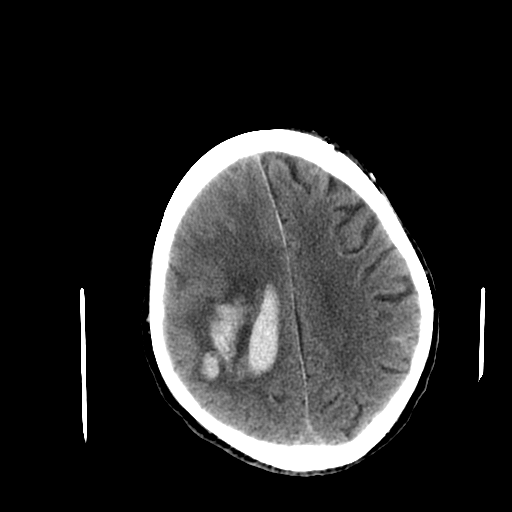

Supplement: S4 Data — (ZIP) [file pone.0295536.s005.zip › S5_Data/FCN_Training set/IM_0017-ID_8cfcfb3df.png]

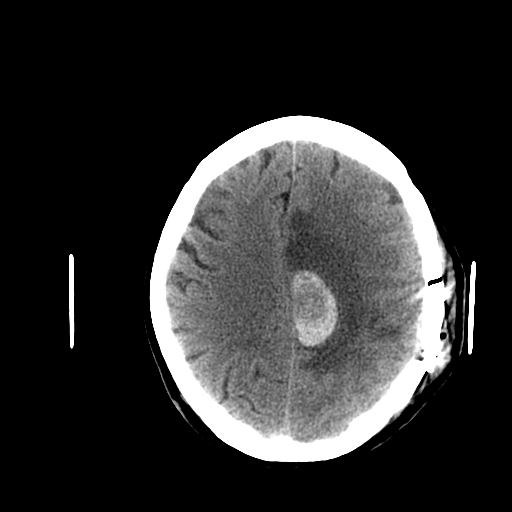

Supplement: S4 Data — (ZIP) [file pone.0295536.s005.zip › S5_Data/FCN_Training set/IM_0017-ID_8e78034f0.png]

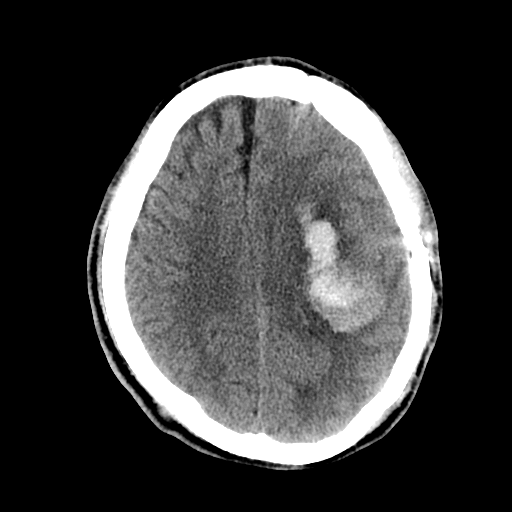

Supplement: S4 Data — (ZIP) [file pone.0295536.s005.zip › S5_Data/FCN_Training set/IM_0017-ID_8f2632a58.png]

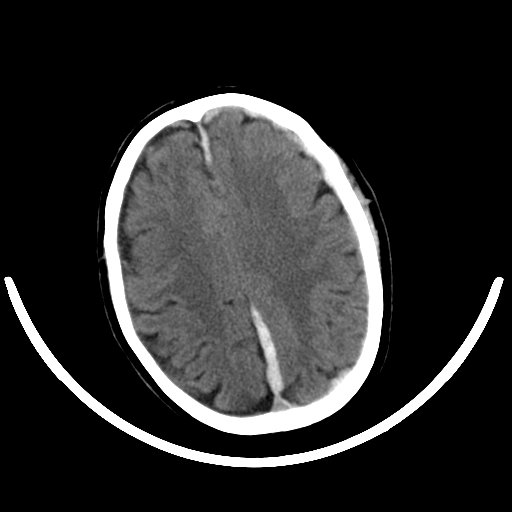

Supplement: S4 Data — (ZIP) [file pone.0295536.s005.zip › S5_Data/FCN_Training set/IM_0017-ID_91278b32e.png]

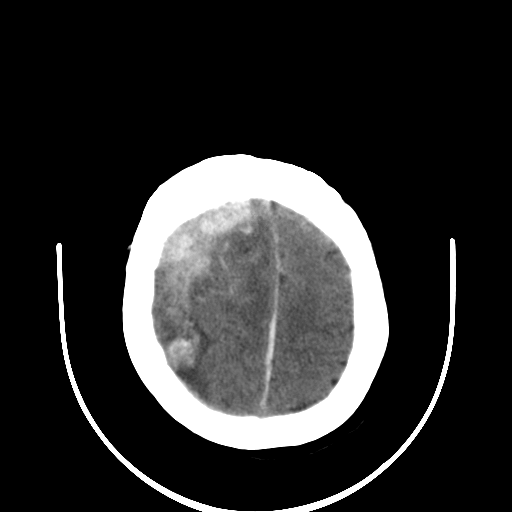

Supplement: S4 Data — (ZIP) [file pone.0295536.s005.zip › S5_Data/FCN_Training set/IM_0017-ID_925cea7d6.png]

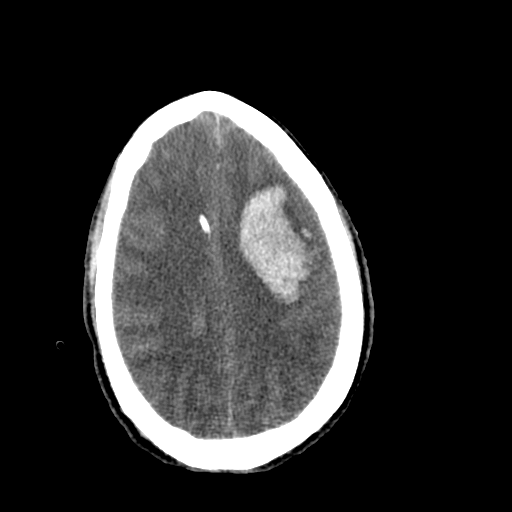

Supplement: S4 Data — (ZIP) [file pone.0295536.s005.zip › S5_Data/FCN_Training set/IM_0017-ID_982748601.png]

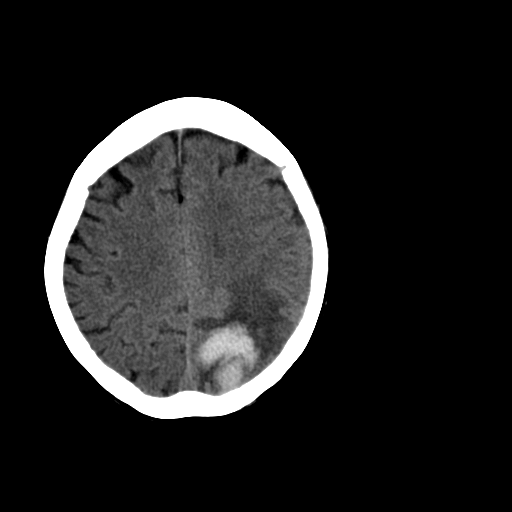

Supplement: S4 Data — (ZIP) [file pone.0295536.s005.zip › S5_Data/FCN_Training set/IM_0017-ID_9835cdac9.png]

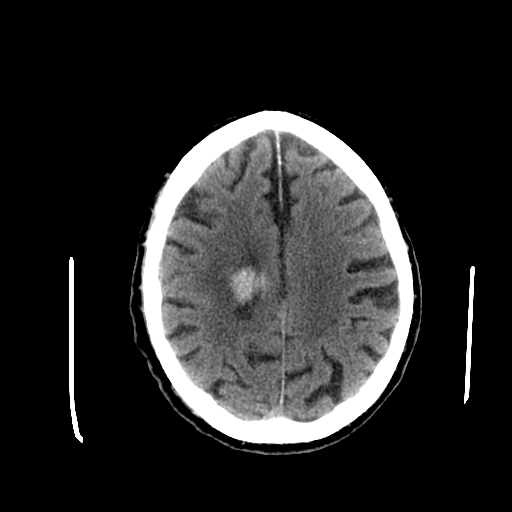

Supplement: S4 Data — (ZIP) [file pone.0295536.s005.zip › S5_Data/FCN_Training set/IM_0017-ID_9a0c69189.png]

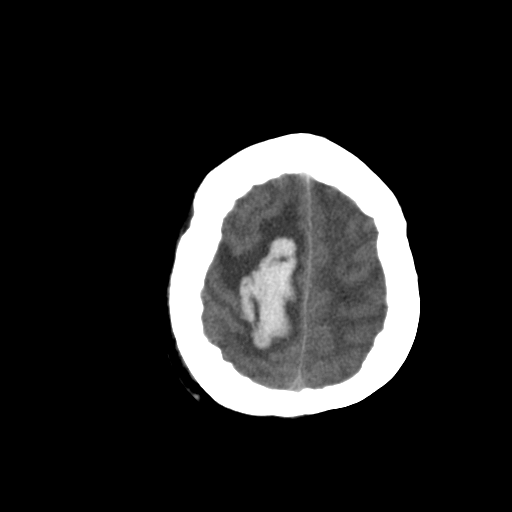

Supplement: S4 Data — (ZIP) [file pone.0295536.s005.zip › S5_Data/FCN_Training set/IM_0017-ID_9bdc5cbf2.png]

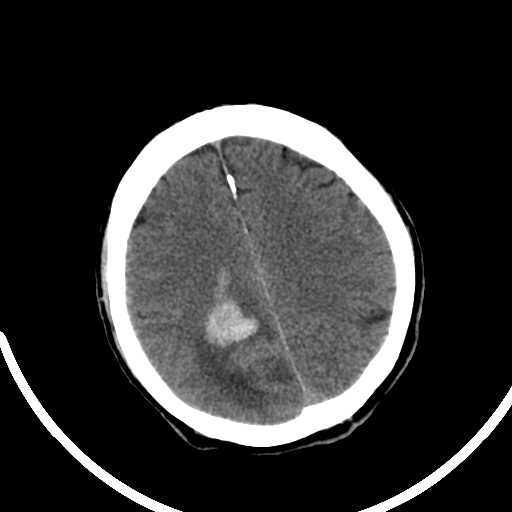

Supplement: S4 Data — (ZIP) [file pone.0295536.s005.zip › S5_Data/FCN_Training set/IM_0017-ID_a024c0f70.png]

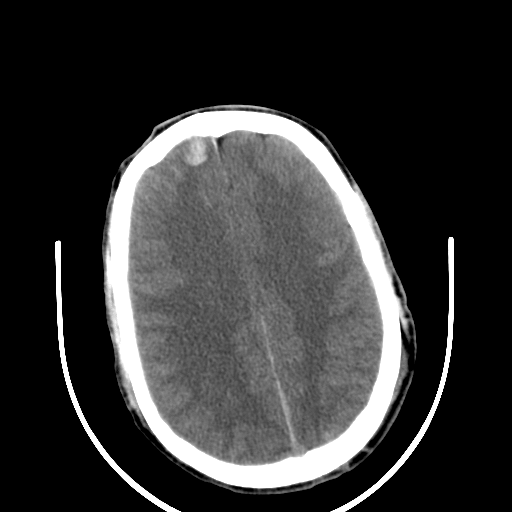

Supplement: S4 Data — (ZIP) [file pone.0295536.s005.zip › S5_Data/FCN_Training set/IM_0017-ID_a23cc408f.png]

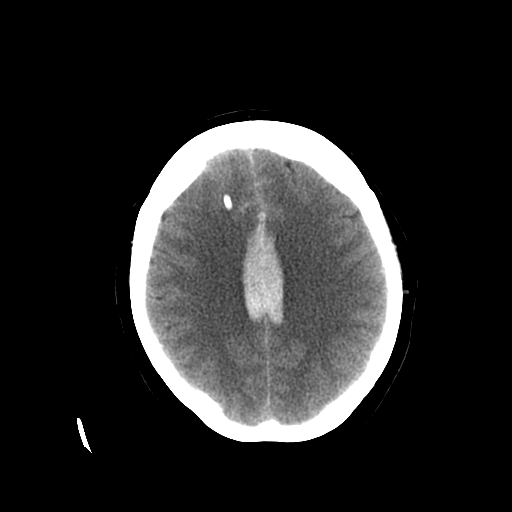

Supplement: S4 Data — (ZIP) [file pone.0295536.s005.zip › S5_Data/FCN_Training set/IM_0017-ID_a4141a849.png]

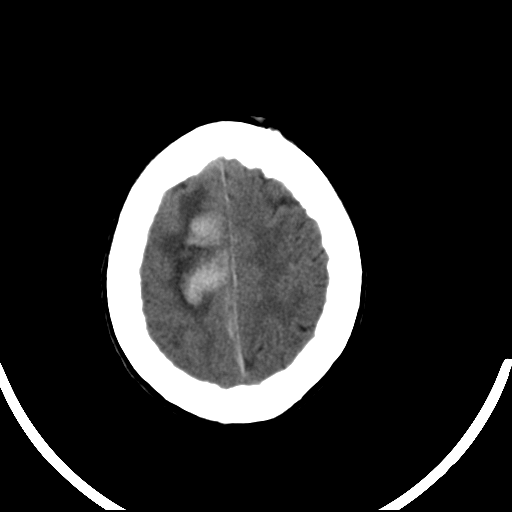

Supplement: S4 Data — (ZIP) [file pone.0295536.s005.zip › S5_Data/FCN_Training set/IM_0017-ID_a8d42e6e9.png]

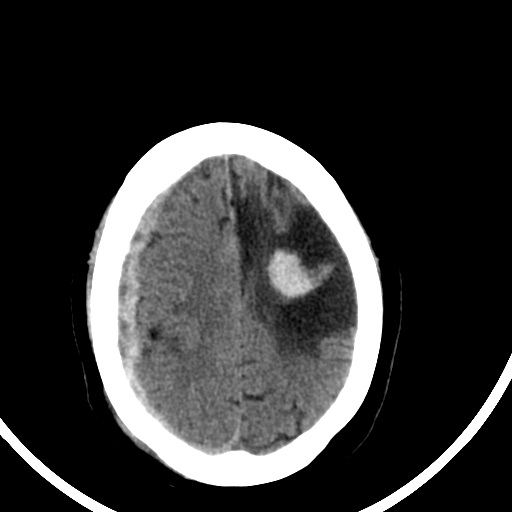

Supplement: S4 Data — (ZIP) [file pone.0295536.s005.zip › S5_Data/FCN_Training set/IM_0017-ID_aa45252b9.png]

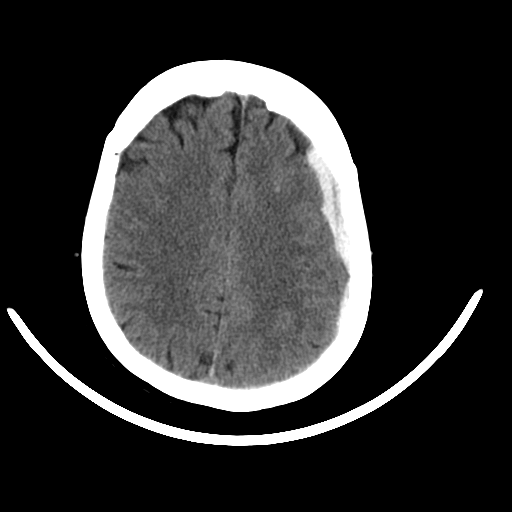

Supplement: S4 Data — (ZIP) [file pone.0295536.s005.zip › S5_Data/FCN_Training set/IM_0017-ID_aaacad122.png]

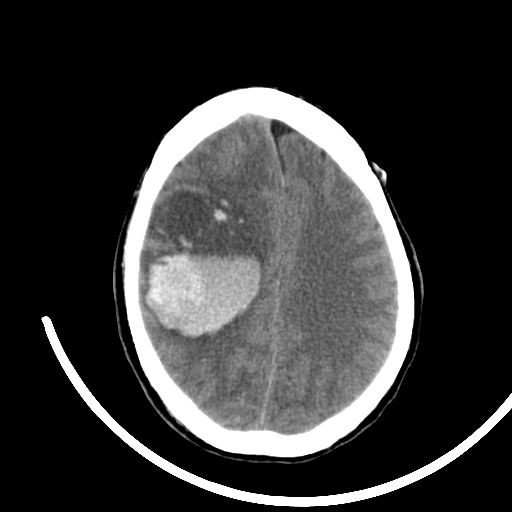

Supplement: S4 Data — (ZIP) [file pone.0295536.s005.zip › S5_Data/FCN_Training set/IM_0017-ID_ad7b5f00c.png]

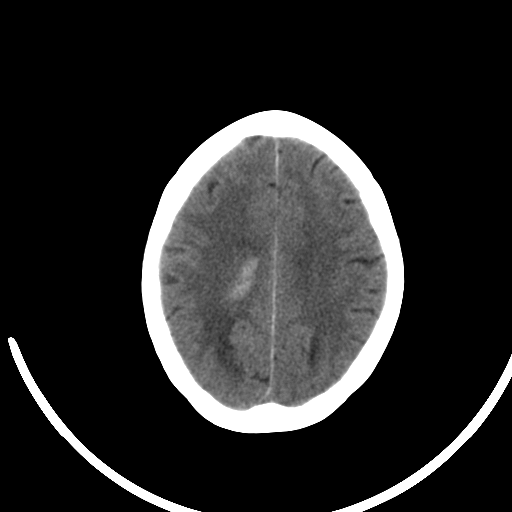

Supplement: S4 Data — (ZIP) [file pone.0295536.s005.zip › S5_Data/FCN_Training set/IM_0017-ID_aeed3d53f.png]

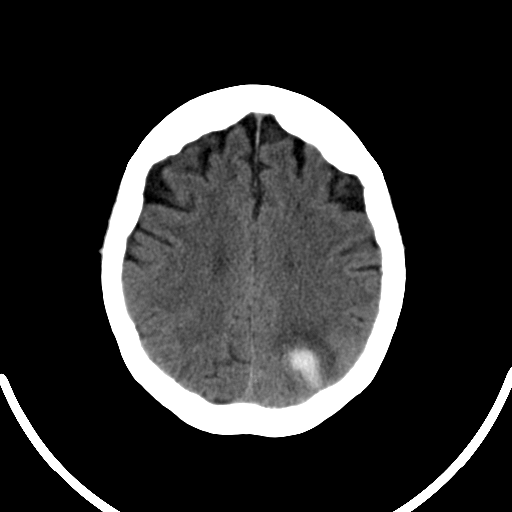

Supplement: S4 Data — (ZIP) [file pone.0295536.s005.zip › S5_Data/FCN_Training set/IM_0017-ID_b1f4a29bd.png]

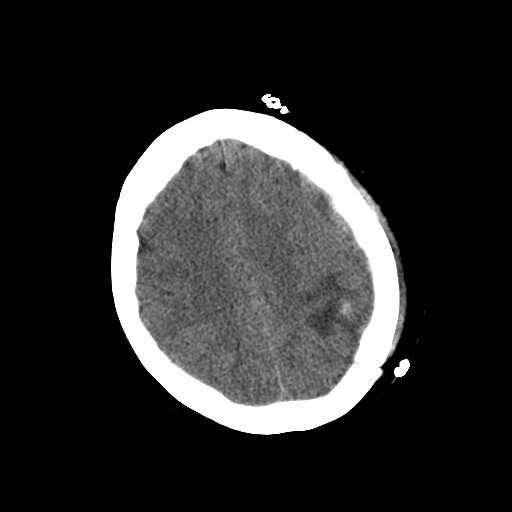

Supplement: S4 Data — (ZIP) [file pone.0295536.s005.zip › S5_Data/FCN_Training set/IM_0017-ID_b1f895b26.png]

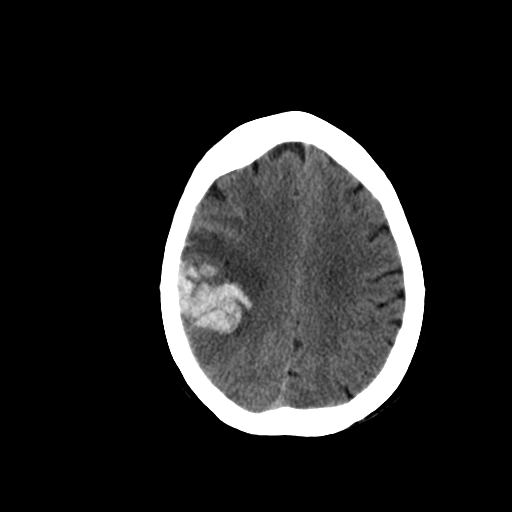

Supplement: S4 Data — (ZIP) [file pone.0295536.s005.zip › S5_Data/FCN_Training set/IM_0017-ID_b4f2dcebc.png]

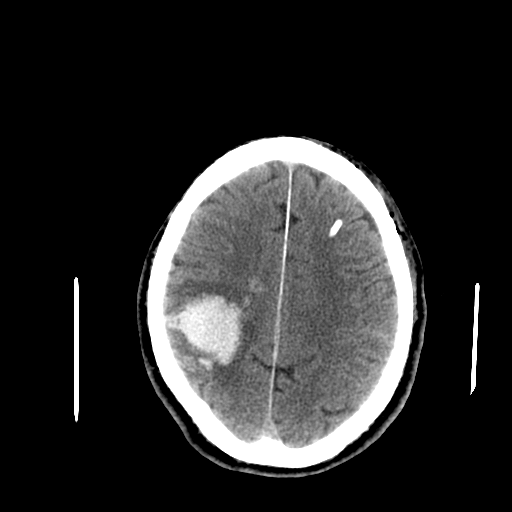

Supplement: S4 Data — (ZIP) [file pone.0295536.s005.zip › S5_Data/FCN_Training set/IM_0017-ID_b97230327.png]

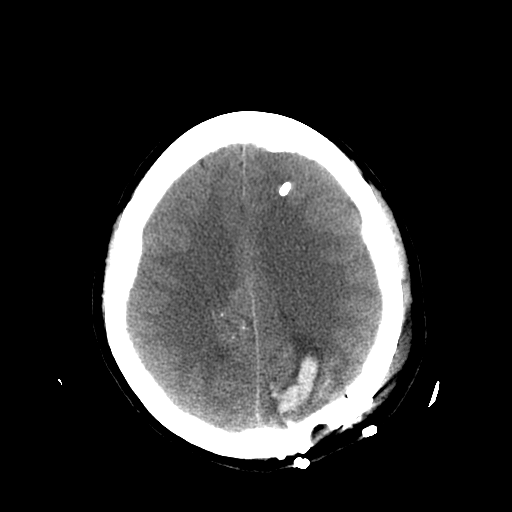

Supplement: S4 Data — (ZIP) [file pone.0295536.s005.zip › S5_Data/FCN_Training set/IM_0017-ID_bfa3ae9d3.png]

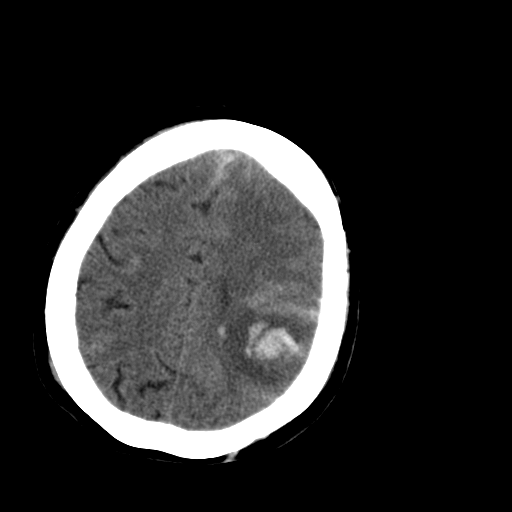

Supplement: S4 Data — (ZIP) [file pone.0295536.s005.zip › S5_Data/FCN_Training set/IM_0017-ID_bff6f5c06.png]

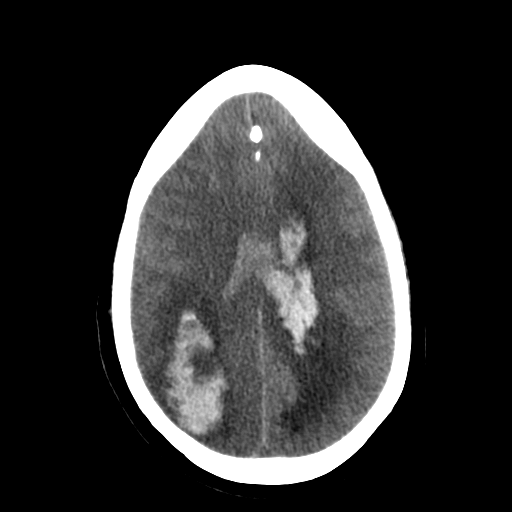

Supplement: S4 Data — (ZIP) [file pone.0295536.s005.zip › S5_Data/FCN_Training set/IM_0017-ID_c0cde8990.png]

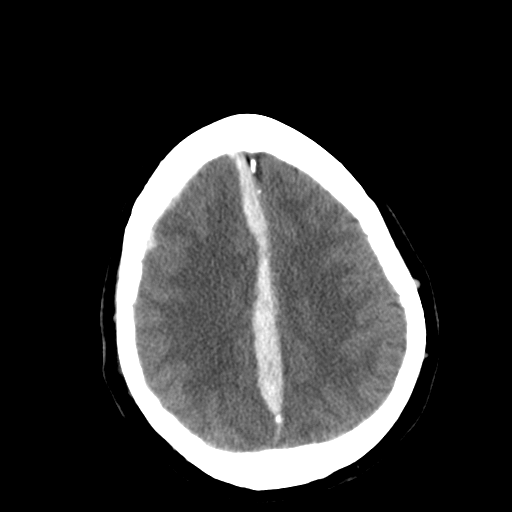

Supplement: S4 Data — (ZIP) [file pone.0295536.s005.zip › S5_Data/FCN_Training set/IM_0017-ID_c1c63442f.png]

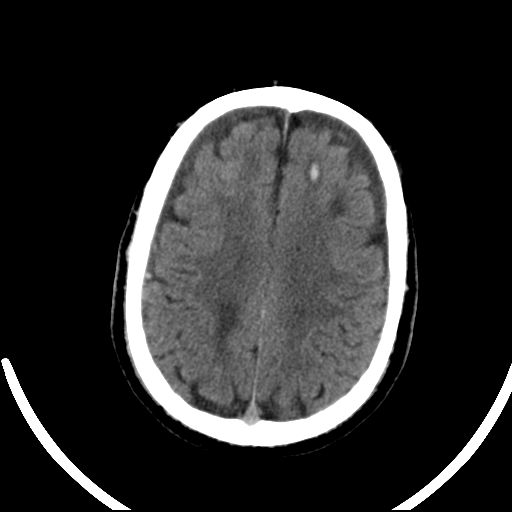

Supplement: S4 Data — (ZIP) [file pone.0295536.s005.zip › S5_Data/FCN_Training set/IM_0017-ID_c2c2d3204.png]

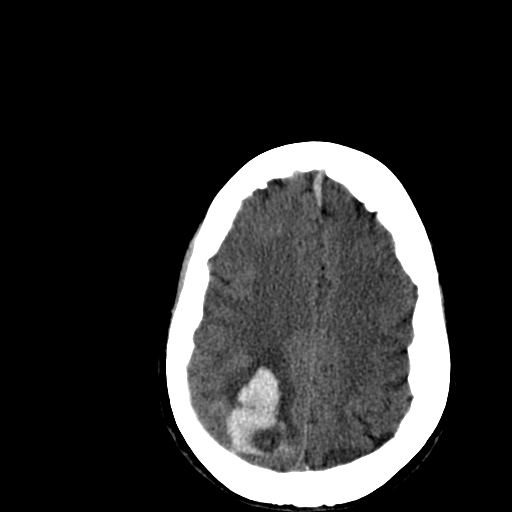

Supplement: S4 Data — (ZIP) [file pone.0295536.s005.zip › S5_Data/FCN_Training set/IM_0017-ID_c49455976.png]

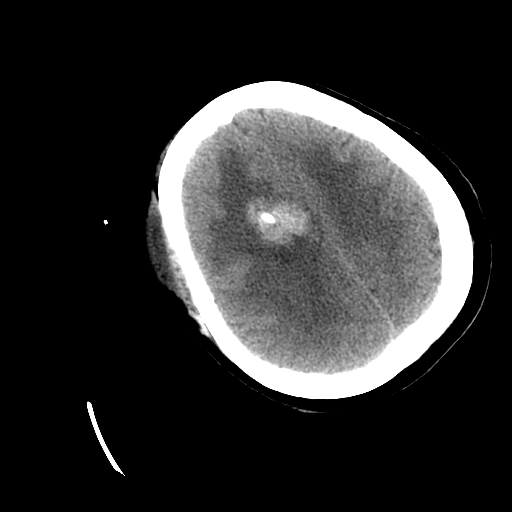

Supplement: S4 Data — (ZIP) [file pone.0295536.s005.zip › S5_Data/FCN_Training set/IM_0017-ID_c556edc07.png]

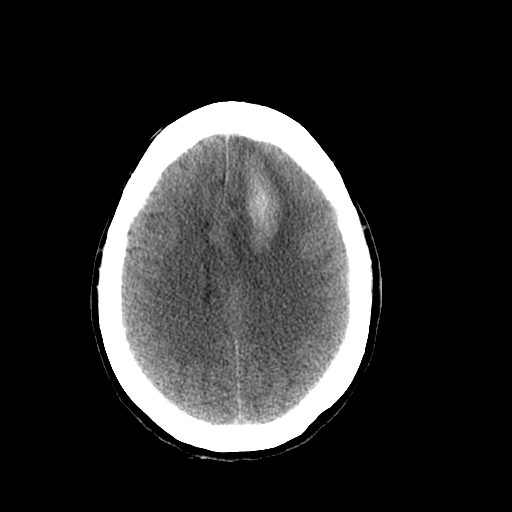

Supplement: S4 Data — (ZIP) [file pone.0295536.s005.zip › S5_Data/FCN_Training set/IM_0017-ID_c5d0f2b49.png]

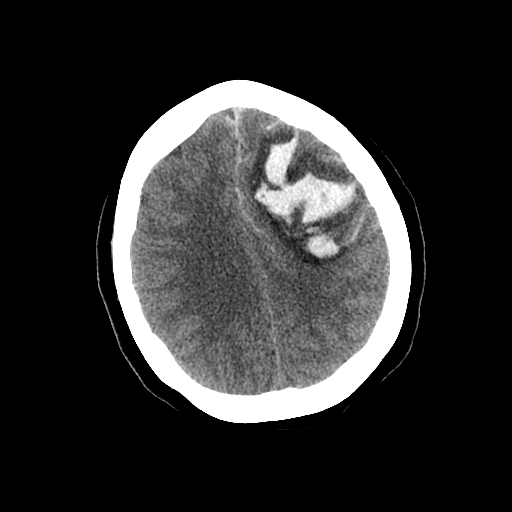

Supplement: S4 Data — (ZIP) [file pone.0295536.s005.zip › S5_Data/FCN_Training set/IM_0017-ID_cd13fd8c1.png]

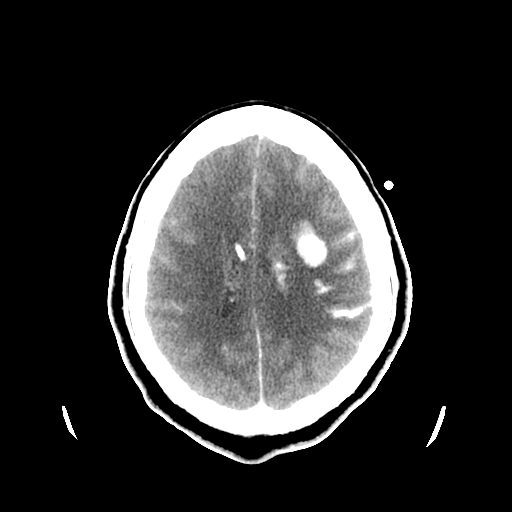

Supplement: S4 Data — (ZIP) [file pone.0295536.s005.zip › S5_Data/FCN_Training set/IM_0017-ID_cdcc48152.png]

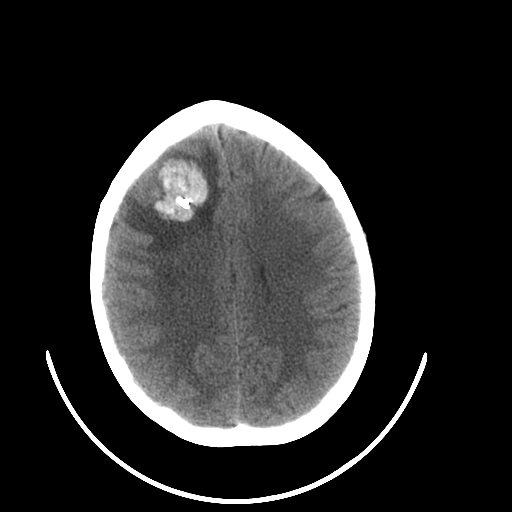

Supplement: S4 Data — (ZIP) [file pone.0295536.s005.zip › S5_Data/FCN_Training set/IM_0017-ID_d16ab5b0b.png]

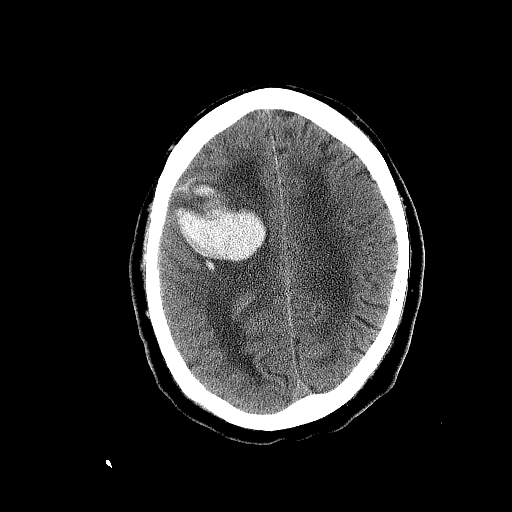

Supplement: S4 Data — (ZIP) [file pone.0295536.s005.zip › S5_Data/FCN_Training set/IM_0017-ID_d278ca49e.png]

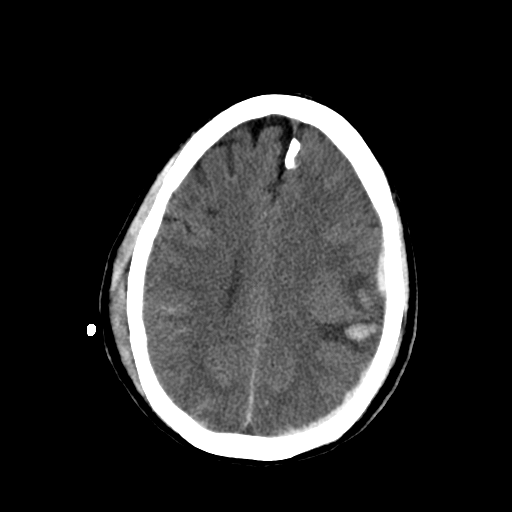

Supplement: S4 Data — (ZIP) [file pone.0295536.s005.zip › S5_Data/FCN_Training set/IM_0017-ID_d2b4f7619.png]

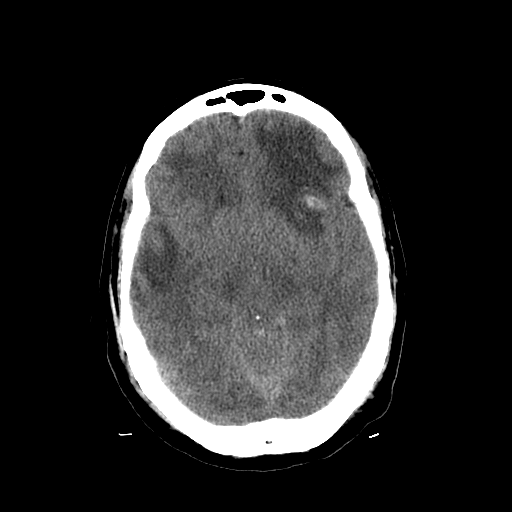

Supplement: S4 Data — (ZIP) [file pone.0295536.s005.zip › S5_Data/FCN_Training set/IM_0017-ID_d372cf611.png]

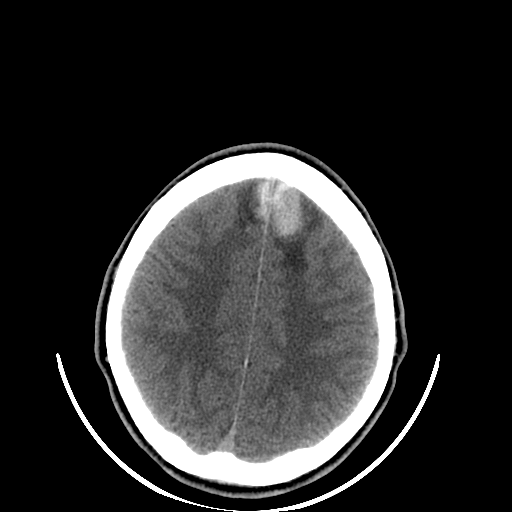

Supplement: S4 Data — (ZIP) [file pone.0295536.s005.zip › S5_Data/FCN_Training set/IM_0017-ID_d99deb703.png]

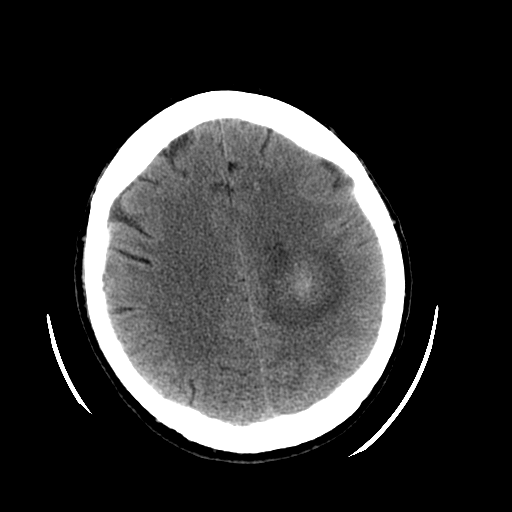

Supplement: S4 Data — (ZIP) [file pone.0295536.s005.zip › S5_Data/FCN_Training set/IM_0017-ID_da45e161c.png]

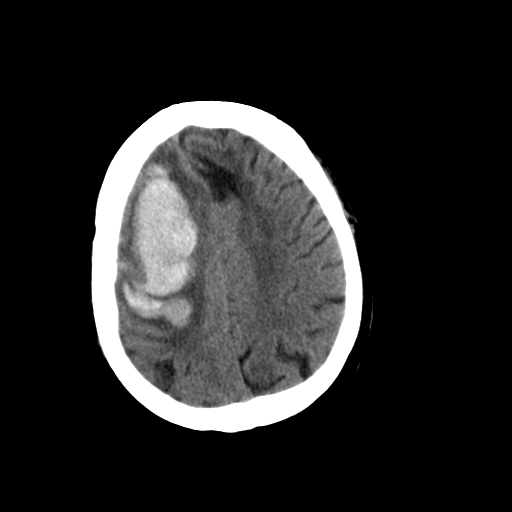

Supplement: S4 Data — (ZIP) [file pone.0295536.s005.zip › S5_Data/FCN_Training set/IM_0017-ID_dabc7b3e7.png]

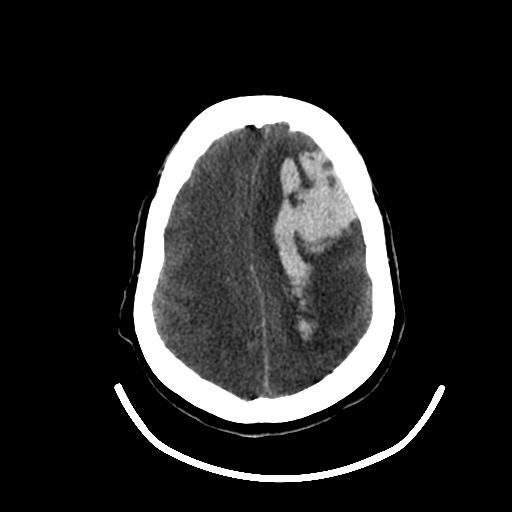

Supplement: S4 Data — (ZIP) [file pone.0295536.s005.zip › S5_Data/FCN_Training set/IM_0017-ID_dd78d3c3f.png]

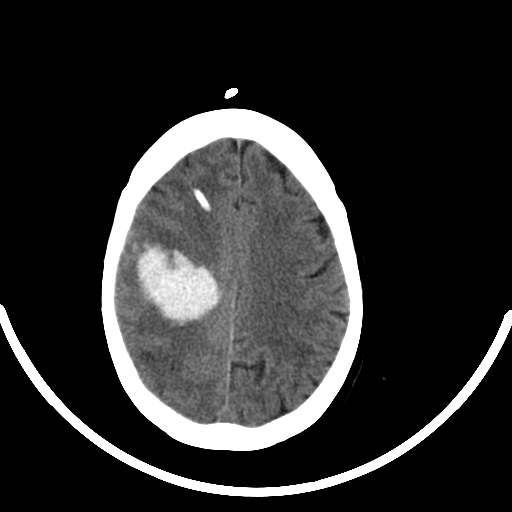

Supplement: S4 Data — (ZIP) [file pone.0295536.s005.zip › S5_Data/FCN_Training set/IM_0017-ID_ddea0d7b5.png]

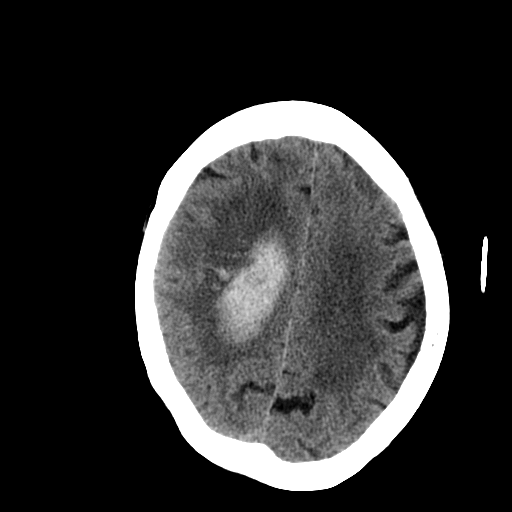

Supplement: S4 Data — (ZIP) [file pone.0295536.s005.zip › S5_Data/FCN_Training set/IM_0017-ID_dfef2dcaf.png]

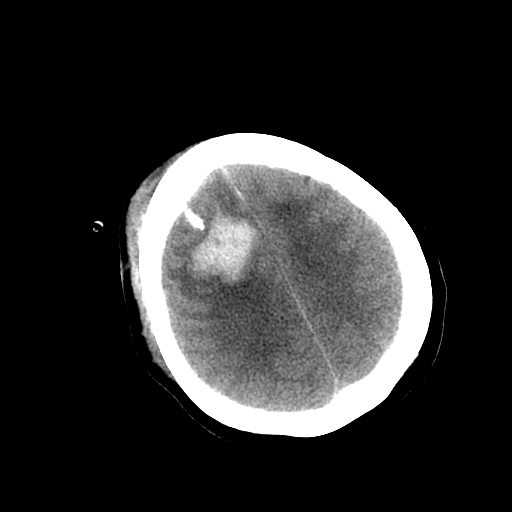

Supplement: S4 Data — (ZIP) [file pone.0295536.s005.zip › S5_Data/FCN_Training set/IM_0017-ID_e5a3cb52b.png]

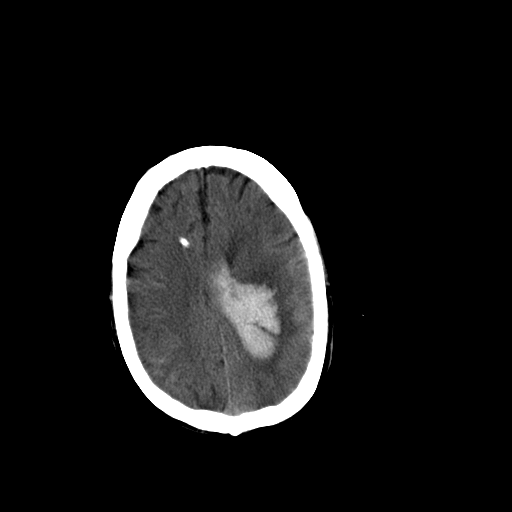

Supplement: S4 Data — (ZIP) [file pone.0295536.s005.zip › S5_Data/FCN_Training set/IM_0017-ID_e772aeb93.png]

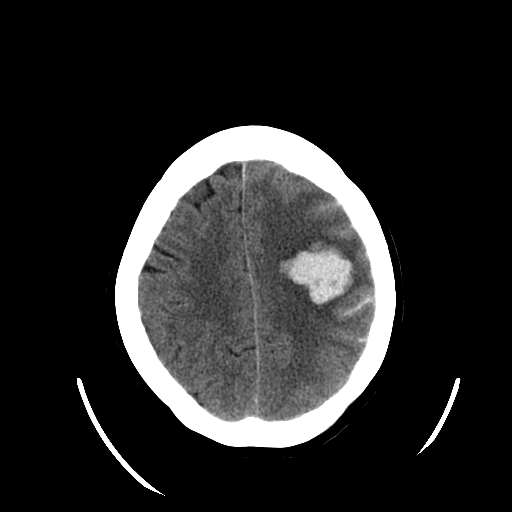

Supplement: S4 Data — (ZIP) [file pone.0295536.s005.zip › S5_Data/FCN_Training set/IM_0017-ID_eadfb607b.png]

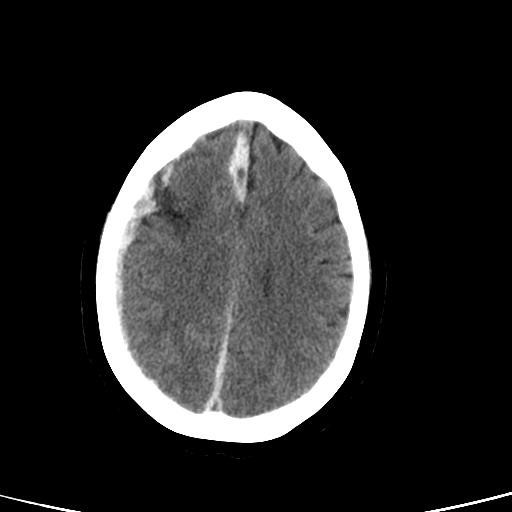

Supplement: S4 Data — (ZIP) [file pone.0295536.s005.zip › S5_Data/FCN_Training set/IM_0017-ID_ef7e27f18.png]

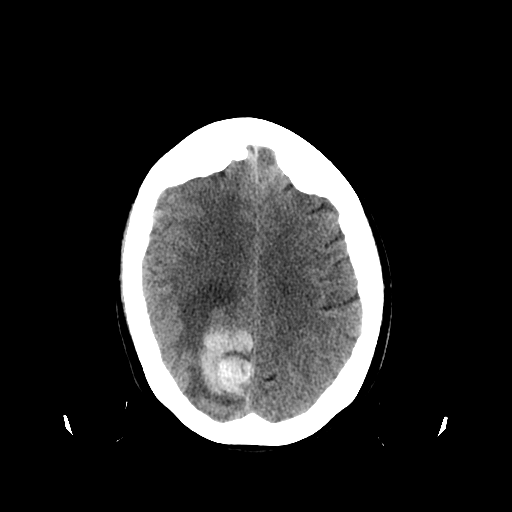

Supplement: S4 Data — (ZIP) [file pone.0295536.s005.zip › S5_Data/FCN_Training set/IM_0017-ID_f2fd5cf47.png]

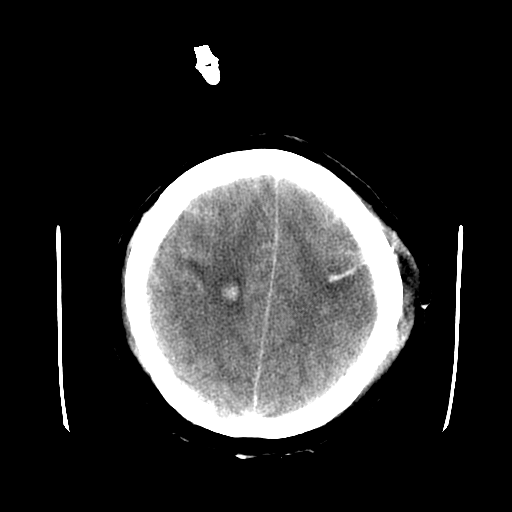

Supplement: S4 Data — (ZIP) [file pone.0295536.s005.zip › S5_Data/FCN_Training set/IM_0017-ID_f50860a4b.png]

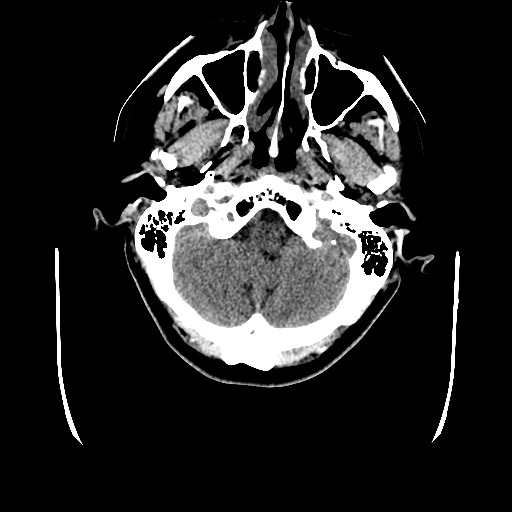

Supplement: S5 Data — (ZIP) [file pone.0295536.s006.zip › S6_Data/Tset set 1/0/82IM_0009-ID_9091863ad.png]
