# Supplementary figures and images for: Adaptive mask-based brain extraction method for head CT images (part 7 of 14)
Source: PLoS One. 2024 Mar 11;19(3):e0295536. doi: 10.1371/journal.pone.0295536 (PMC10927156; doi:10.1371/journal.pone.0295536)

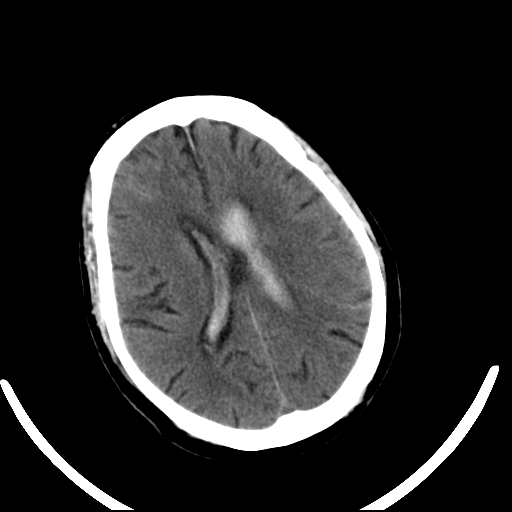

Supplement: S4 Data — (ZIP) [file pone.0295536.s005.zip › S5_Data/FCN_Training set/IM_0013-ID_d3d5c12eb.png]

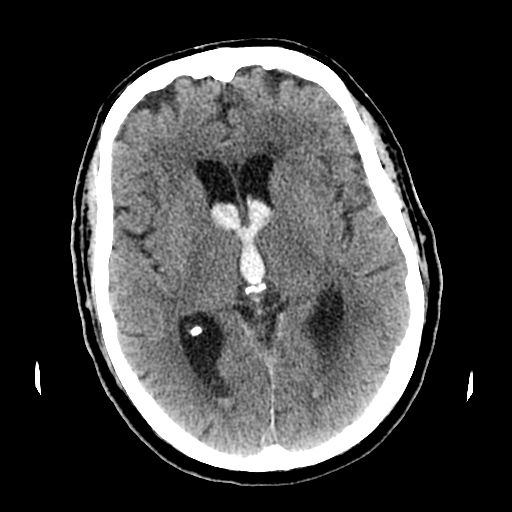

Supplement: S4 Data — (ZIP) [file pone.0295536.s005.zip › S5_Data/FCN_Training set/IM_0013-ID_d71c33904.png]

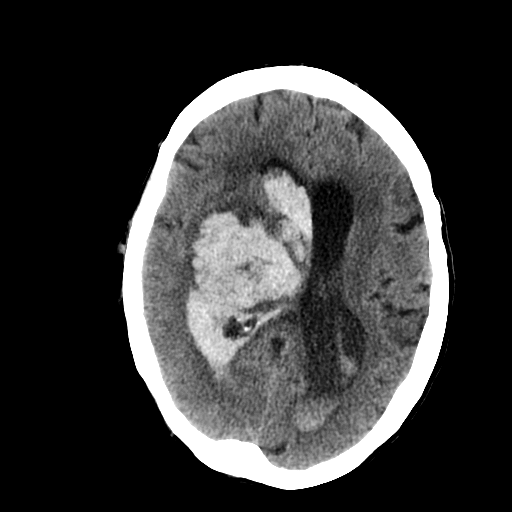

Supplement: S4 Data — (ZIP) [file pone.0295536.s005.zip › S5_Data/FCN_Training set/IM_0013-ID_dbe2dce0c.png]

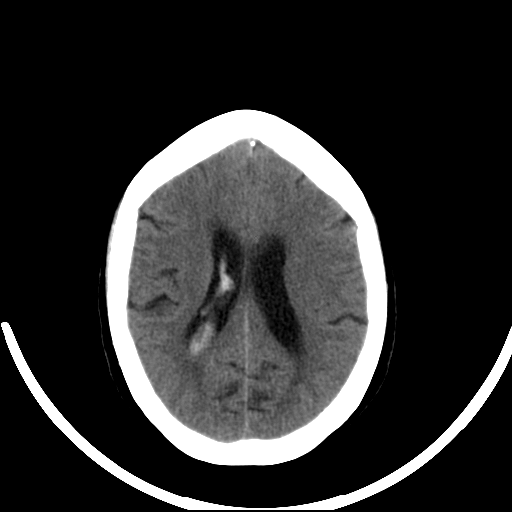

Supplement: S4 Data — (ZIP) [file pone.0295536.s005.zip › S5_Data/FCN_Training set/IM_0013-ID_dbe4e0802.png]

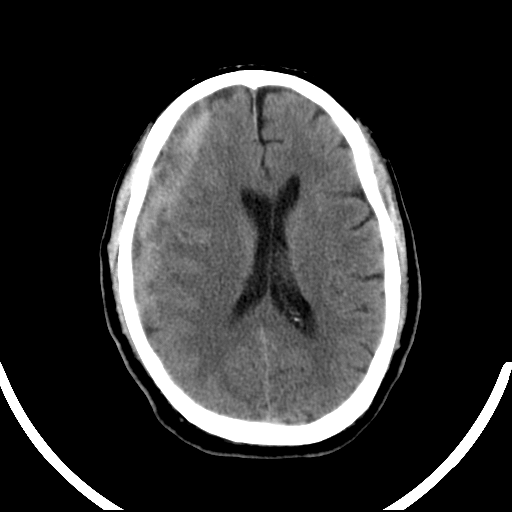

Supplement: S4 Data — (ZIP) [file pone.0295536.s005.zip › S5_Data/FCN_Training set/IM_0013-ID_dcfef51ae.png]

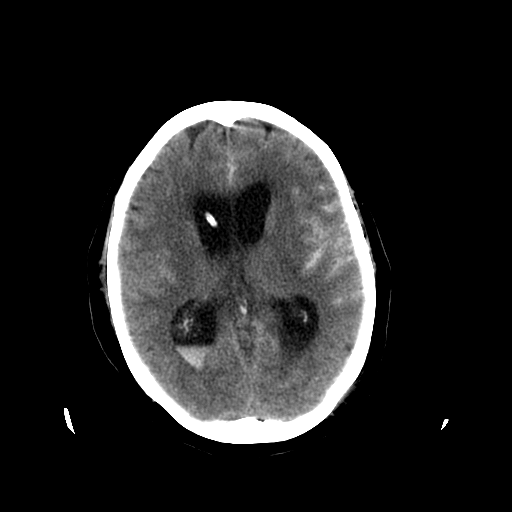

Supplement: S4 Data — (ZIP) [file pone.0295536.s005.zip › S5_Data/FCN_Training set/IM_0013-ID_dda0f785a.png]

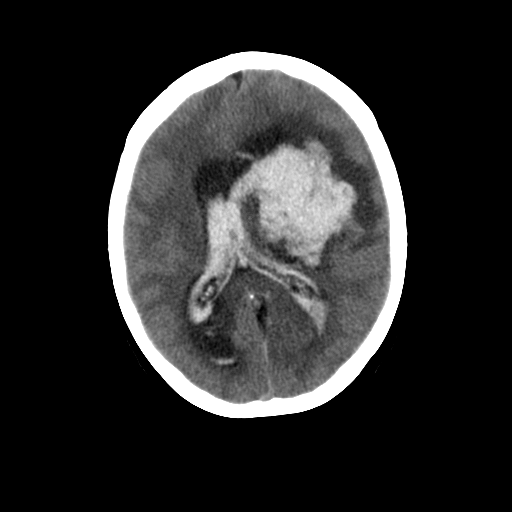

Supplement: S4 Data — (ZIP) [file pone.0295536.s005.zip › S5_Data/FCN_Training set/IM_0013-ID_de071db32.png]

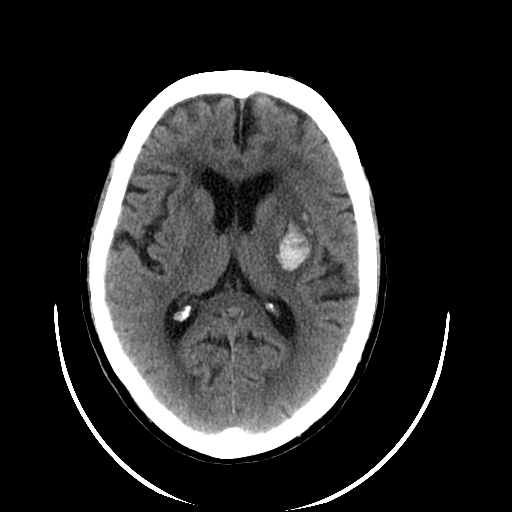

Supplement: S4 Data — (ZIP) [file pone.0295536.s005.zip › S5_Data/FCN_Training set/IM_0013-ID_e0be92c25.png]

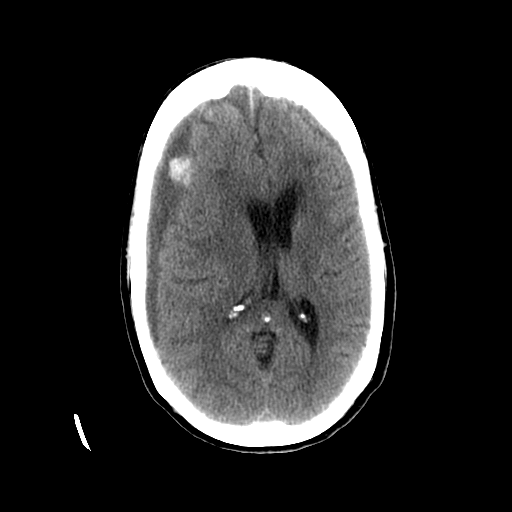

Supplement: S4 Data — (ZIP) [file pone.0295536.s005.zip › S5_Data/FCN_Training set/IM_0013-ID_e163517b3.png]

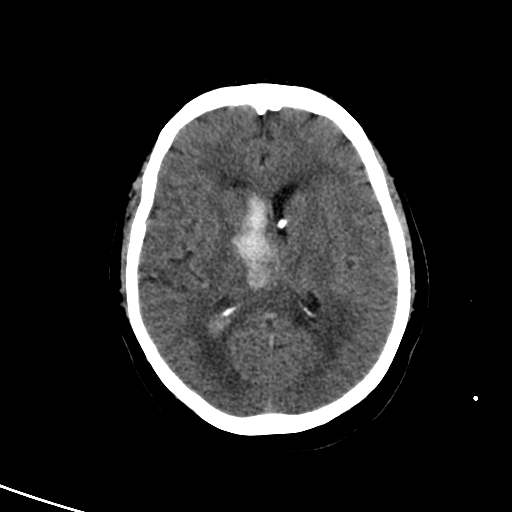

Supplement: S4 Data — (ZIP) [file pone.0295536.s005.zip › S5_Data/FCN_Training set/IM_0013-ID_e223fd64a.png]

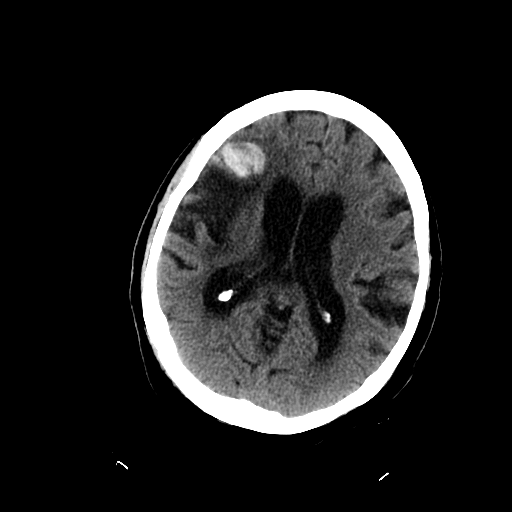

Supplement: S4 Data — (ZIP) [file pone.0295536.s005.zip › S5_Data/FCN_Training set/IM_0013-ID_e36405852.png]

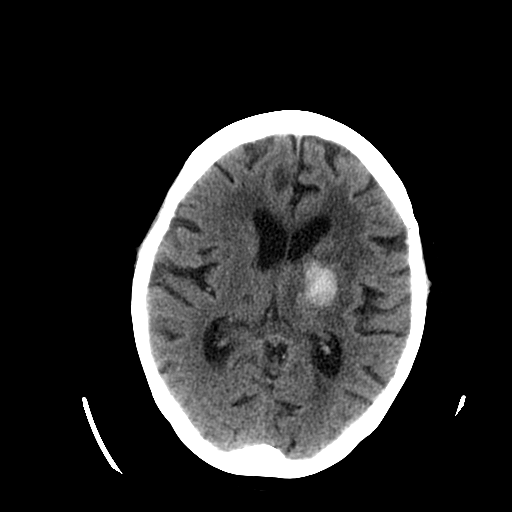

Supplement: S4 Data — (ZIP) [file pone.0295536.s005.zip › S5_Data/FCN_Training set/IM_0013-ID_e6063bf09.png]

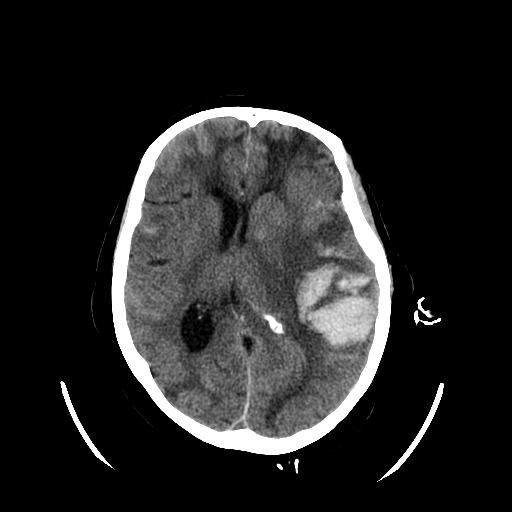

Supplement: S4 Data — (ZIP) [file pone.0295536.s005.zip › S5_Data/FCN_Training set/IM_0013-ID_e86031a23.png]

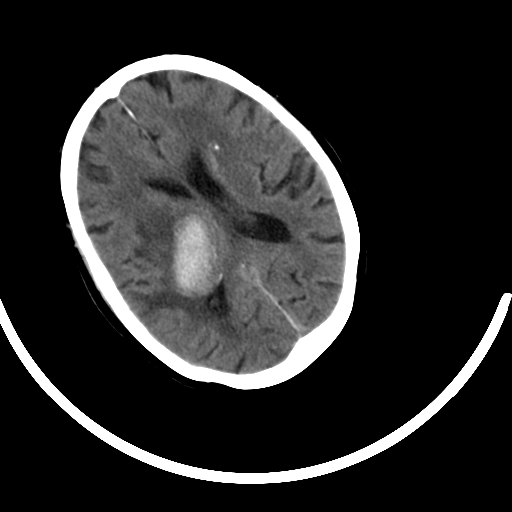

Supplement: S4 Data — (ZIP) [file pone.0295536.s005.zip › S5_Data/FCN_Training set/IM_0013-ID_ec8144f6e.png]

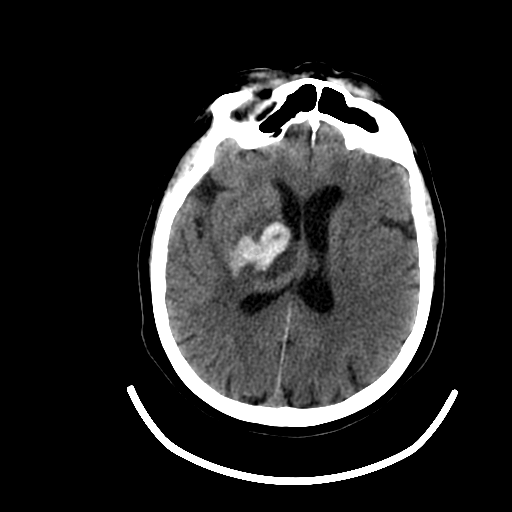

Supplement: S4 Data — (ZIP) [file pone.0295536.s005.zip › S5_Data/FCN_Training set/IM_0013-ID_ee0dc2d34.png]

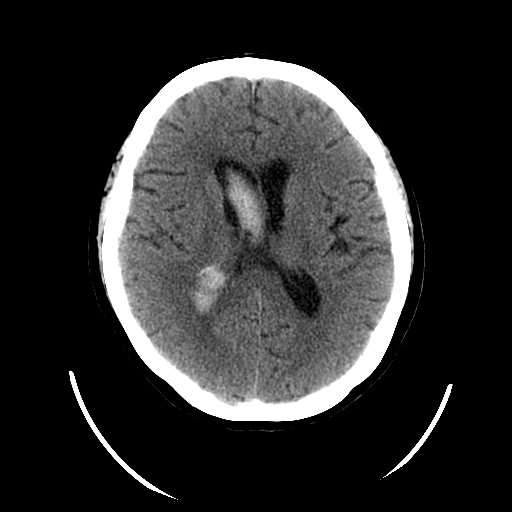

Supplement: S4 Data — (ZIP) [file pone.0295536.s005.zip › S5_Data/FCN_Training set/IM_0013-ID_efd47b155.png]

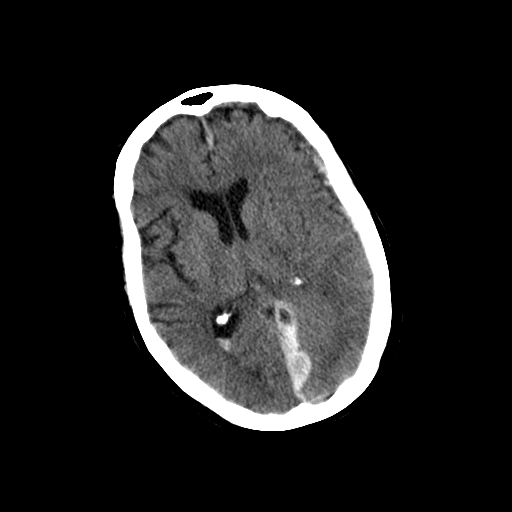

Supplement: S4 Data — (ZIP) [file pone.0295536.s005.zip › S5_Data/FCN_Training set/IM_0013-ID_efe69ee01.png]

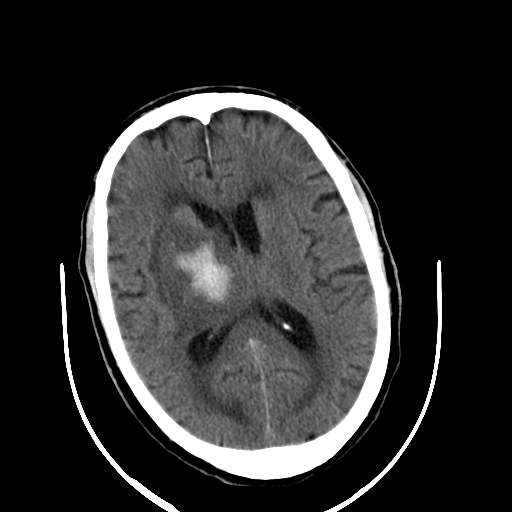

Supplement: S4 Data — (ZIP) [file pone.0295536.s005.zip › S5_Data/FCN_Training set/IM_0013-ID_f0148c63f.png]

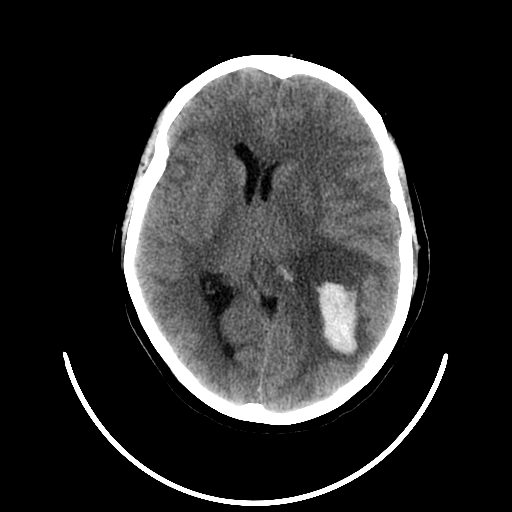

Supplement: S4 Data — (ZIP) [file pone.0295536.s005.zip › S5_Data/FCN_Training set/IM_0013-ID_f03d3cc6b.png]

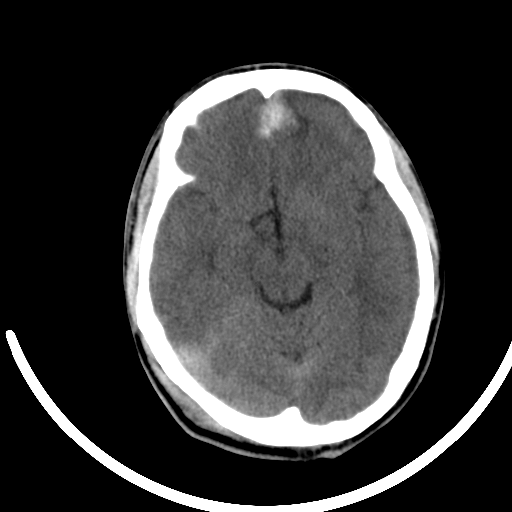

Supplement: S4 Data — (ZIP) [file pone.0295536.s005.zip › S5_Data/FCN_Training set/IM_0013-ID_f2e392735.png]

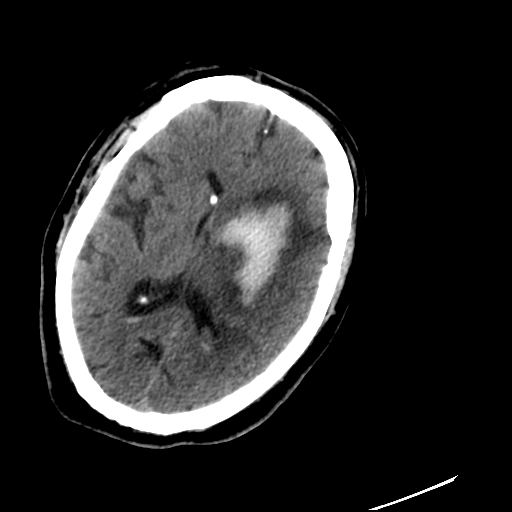

Supplement: S4 Data — (ZIP) [file pone.0295536.s005.zip › S5_Data/FCN_Training set/IM_0013-ID_f2f930122.png]

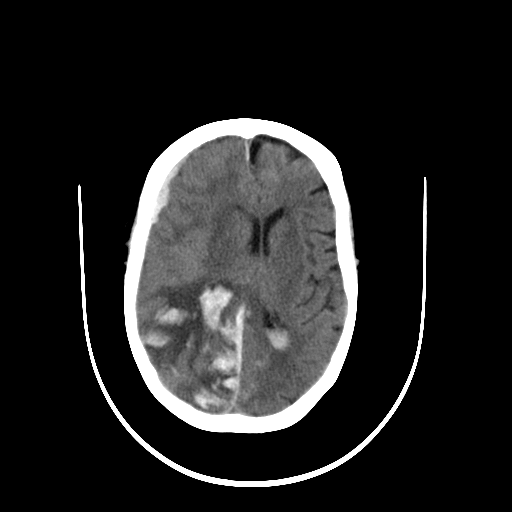

Supplement: S4 Data — (ZIP) [file pone.0295536.s005.zip › S5_Data/FCN_Training set/IM_0013-ID_f44bec5a9.png]

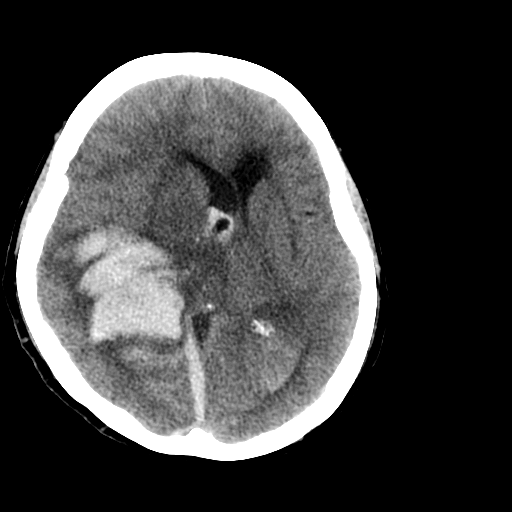

Supplement: S4 Data — (ZIP) [file pone.0295536.s005.zip › S5_Data/FCN_Training set/IM_0013-ID_f47efc993.png]

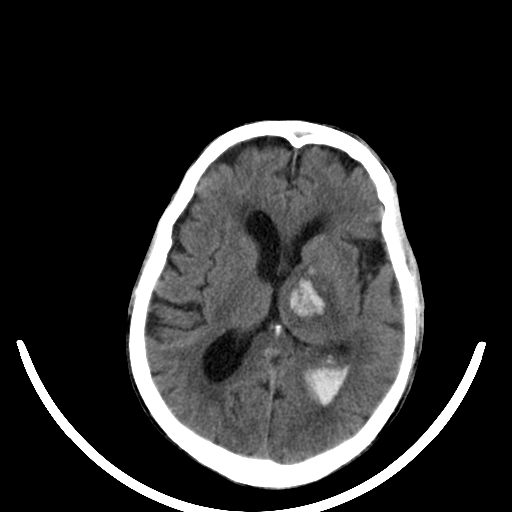

Supplement: S4 Data — (ZIP) [file pone.0295536.s005.zip › S5_Data/FCN_Training set/IM_0013-ID_f50d4823d.png]

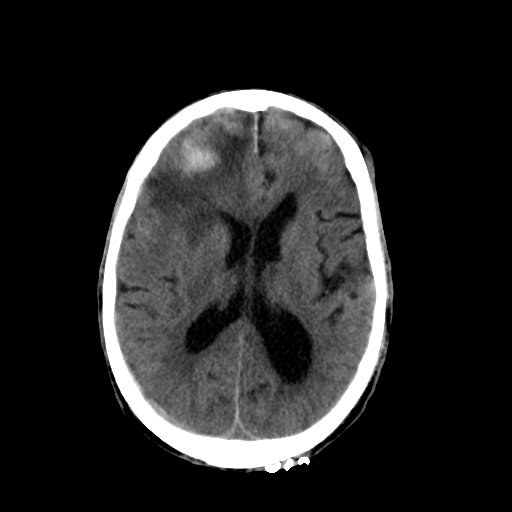

Supplement: S4 Data — (ZIP) [file pone.0295536.s005.zip › S5_Data/FCN_Training set/IM_0013-ID_f7f2e9cf1.png]

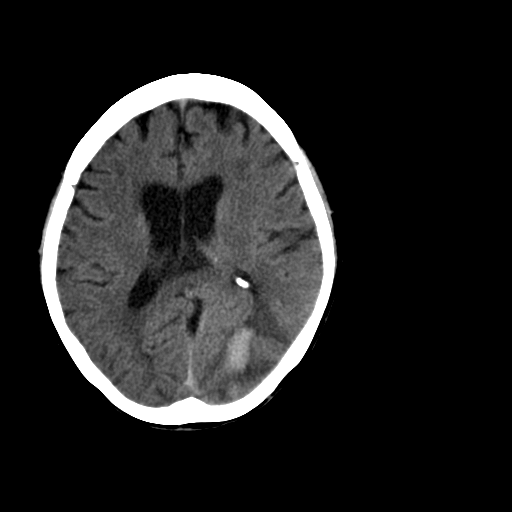

Supplement: S4 Data — (ZIP) [file pone.0295536.s005.zip › S5_Data/FCN_Training set/IM_0013-ID_f7f9e3974.png]

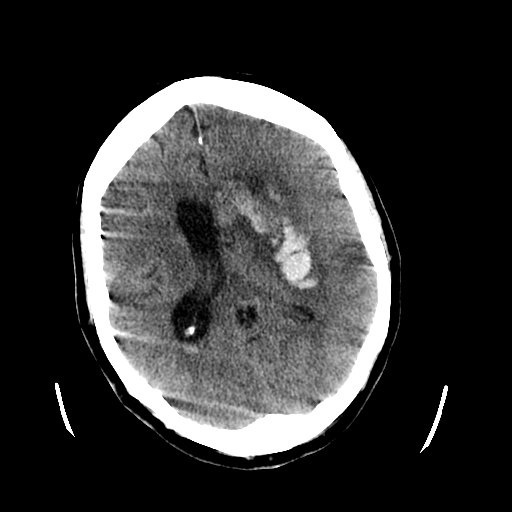

Supplement: S4 Data — (ZIP) [file pone.0295536.s005.zip › S5_Data/FCN_Training set/IM_0013-ID_f9cfbaa36.png]

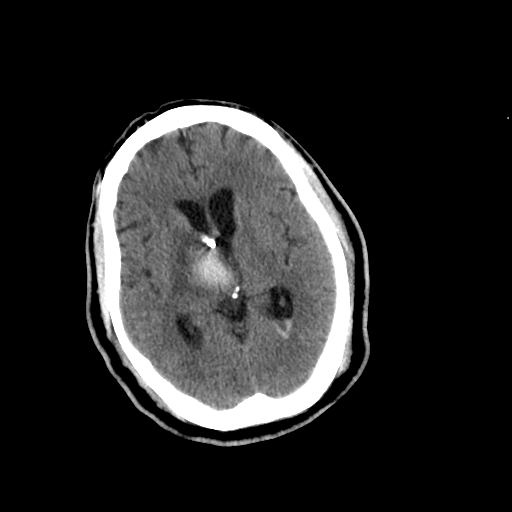

Supplement: S4 Data — (ZIP) [file pone.0295536.s005.zip › S5_Data/FCN_Training set/IM_0013-ID_fa96b7c98.png]

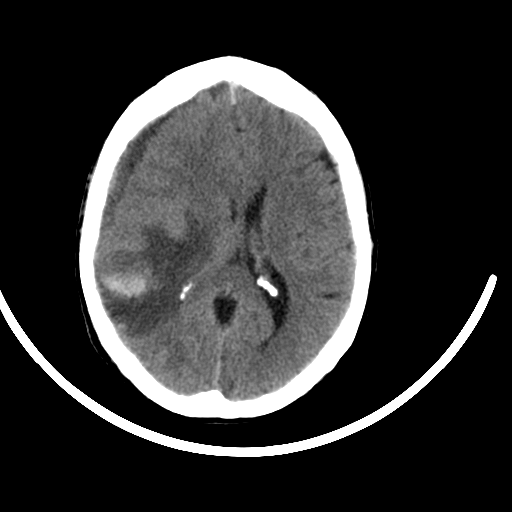

Supplement: S4 Data — (ZIP) [file pone.0295536.s005.zip › S5_Data/FCN_Training set/IM_0013-ID_fac3ddd69.png]

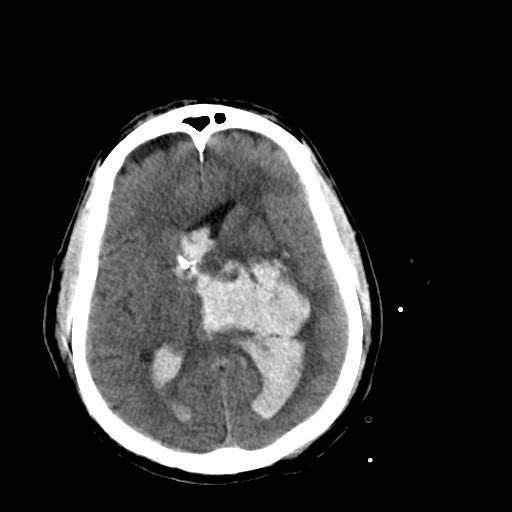

Supplement: S4 Data — (ZIP) [file pone.0295536.s005.zip › S5_Data/FCN_Training set/IM_0013-ID_fb20a4398.png]

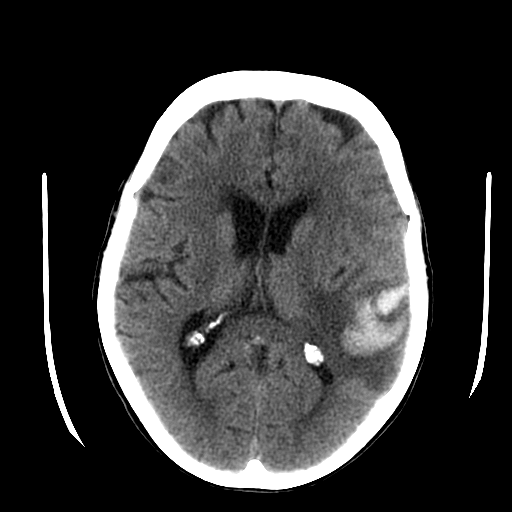

Supplement: S4 Data — (ZIP) [file pone.0295536.s005.zip › S5_Data/FCN_Training set/IM_0013-ID_fbde71d6f.png]

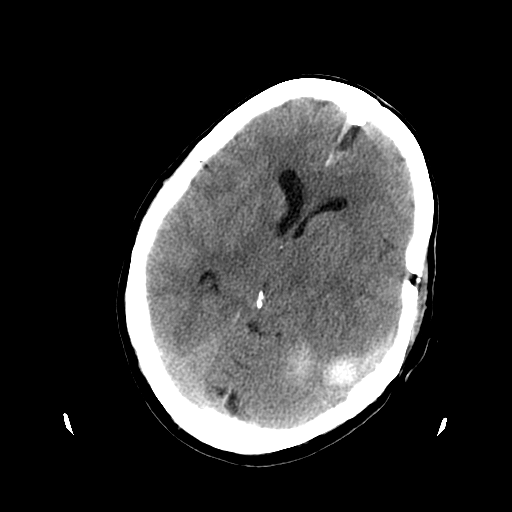

Supplement: S4 Data — (ZIP) [file pone.0295536.s005.zip › S5_Data/FCN_Training set/IM_0013-ID_fc038f904.png]

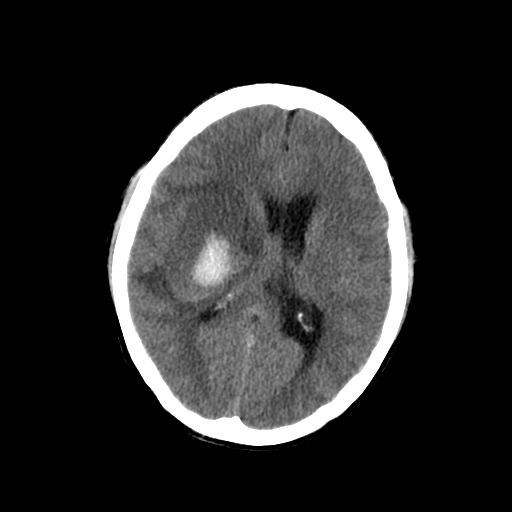

Supplement: S4 Data — (ZIP) [file pone.0295536.s005.zip › S5_Data/FCN_Training set/IM_0013-ID_fce3500c0.png]

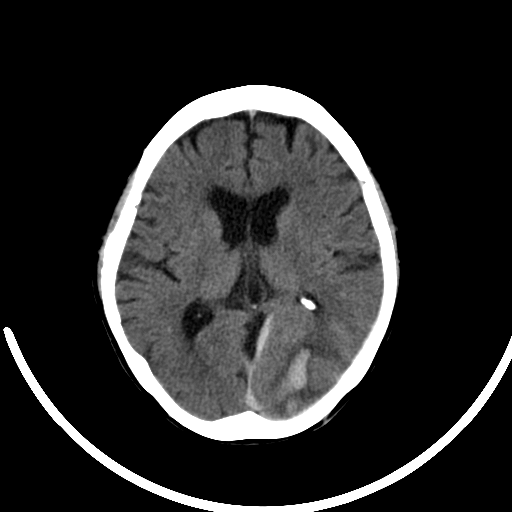

Supplement: S4 Data — (ZIP) [file pone.0295536.s005.zip › S5_Data/FCN_Training set/IM_0013-ID_fd2c8b41b.png]

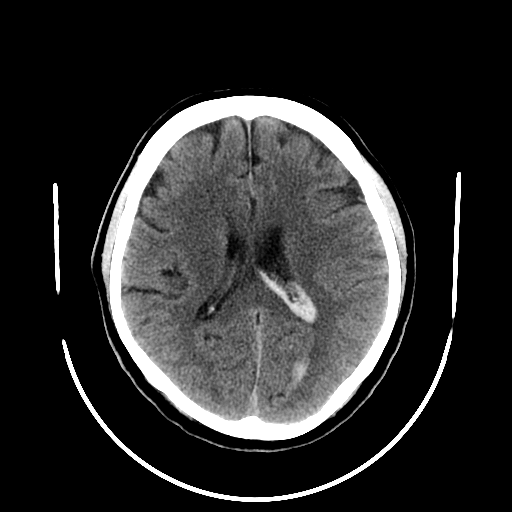

Supplement: S4 Data — (ZIP) [file pone.0295536.s005.zip › S5_Data/FCN_Training set/IM_0013-ID_fd2f069a9.png]

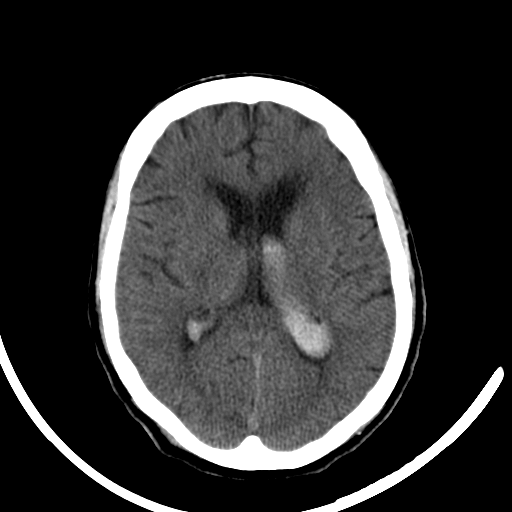

Supplement: S4 Data — (ZIP) [file pone.0295536.s005.zip › S5_Data/FCN_Training set/IM_0013-ID_fe6a07d28.png]

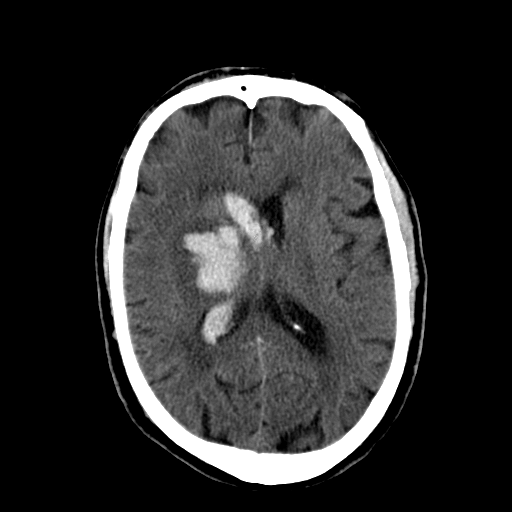

Supplement: S4 Data — (ZIP) [file pone.0295536.s005.zip › S5_Data/FCN_Training set/IM_0013-ID_ffec87273.png]

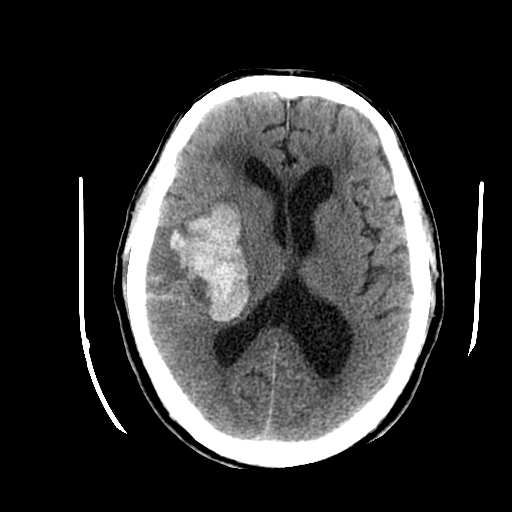

Supplement: S4 Data — (ZIP) [file pone.0295536.s005.zip › S5_Data/FCN_Training set/IM_0014-ID_01de40a17.png]

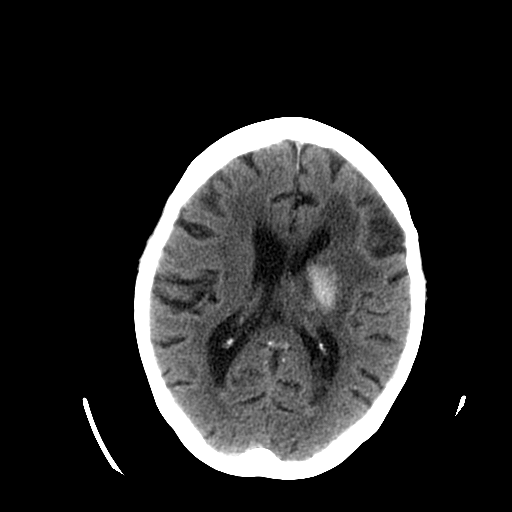

Supplement: S4 Data — (ZIP) [file pone.0295536.s005.zip › S5_Data/FCN_Training set/IM_0014-ID_03d4943a9.png]

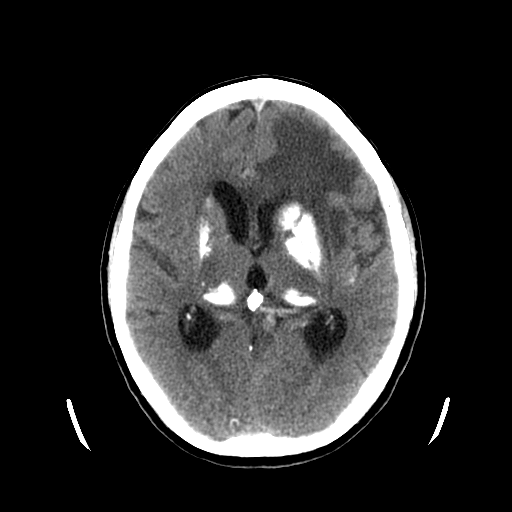

Supplement: S4 Data — (ZIP) [file pone.0295536.s005.zip › S5_Data/FCN_Training set/IM_0014-ID_08a3fc4a2.png]

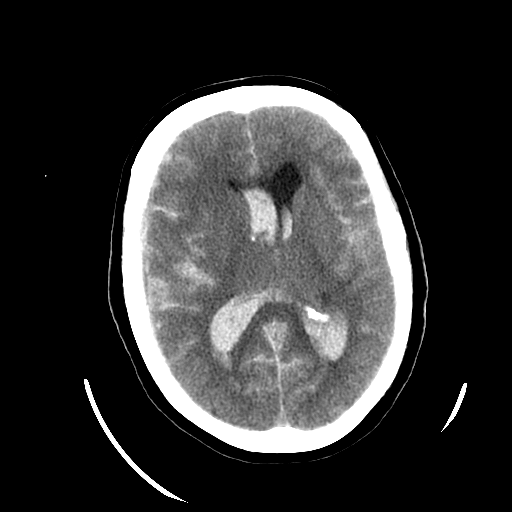

Supplement: S4 Data — (ZIP) [file pone.0295536.s005.zip › S5_Data/FCN_Training set/IM_0014-ID_0a797e4ef.png]

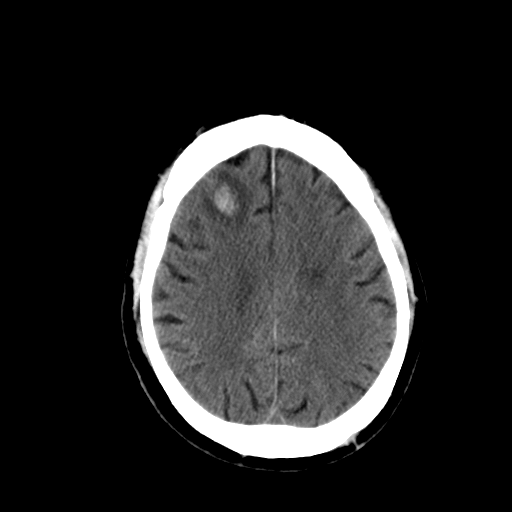

Supplement: S4 Data — (ZIP) [file pone.0295536.s005.zip › S5_Data/FCN_Training set/IM_0014-ID_0ce445973.png]

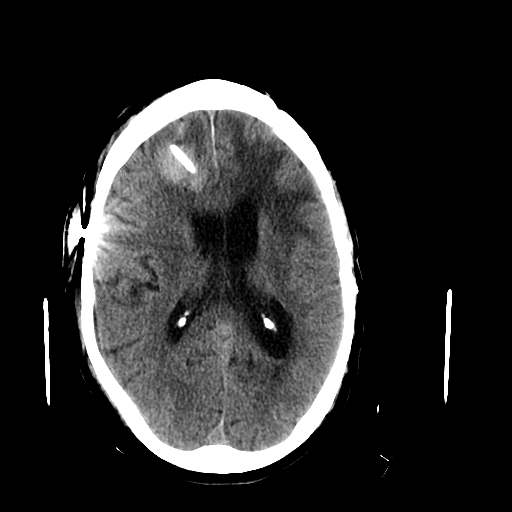

Supplement: S4 Data — (ZIP) [file pone.0295536.s005.zip › S5_Data/FCN_Training set/IM_0014-ID_0d3f5260c.png]

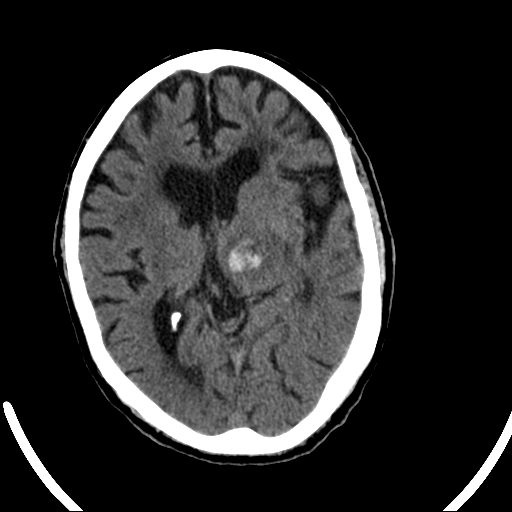

Supplement: S4 Data — (ZIP) [file pone.0295536.s005.zip › S5_Data/FCN_Training set/IM_0014-ID_0f8b44775.png]

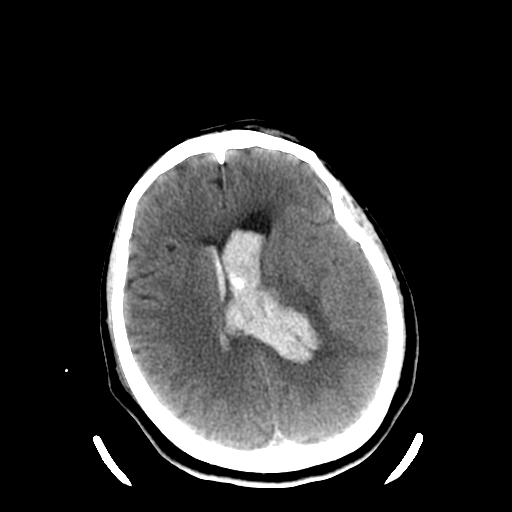

Supplement: S4 Data — (ZIP) [file pone.0295536.s005.zip › S5_Data/FCN_Training set/IM_0014-ID_1b9e0d811.png]

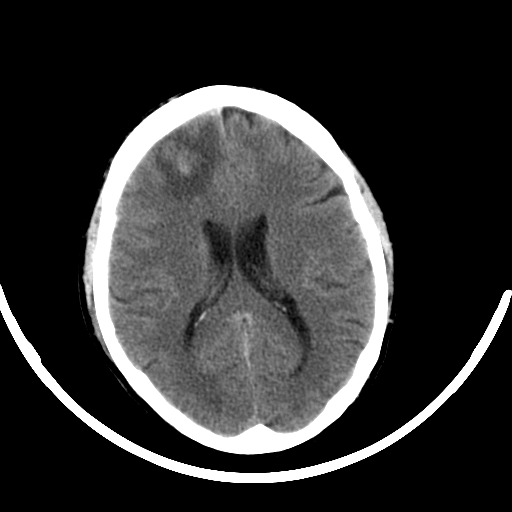

Supplement: S4 Data — (ZIP) [file pone.0295536.s005.zip › S5_Data/FCN_Training set/IM_0014-ID_1c128e0dc.png]

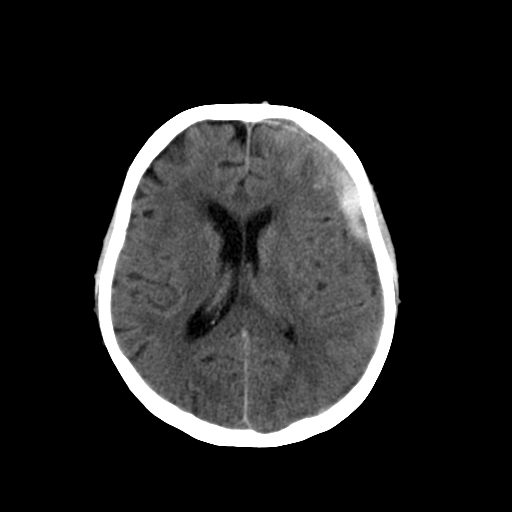

Supplement: S4 Data — (ZIP) [file pone.0295536.s005.zip › S5_Data/FCN_Training set/IM_0014-ID_1c8e240bd.png]

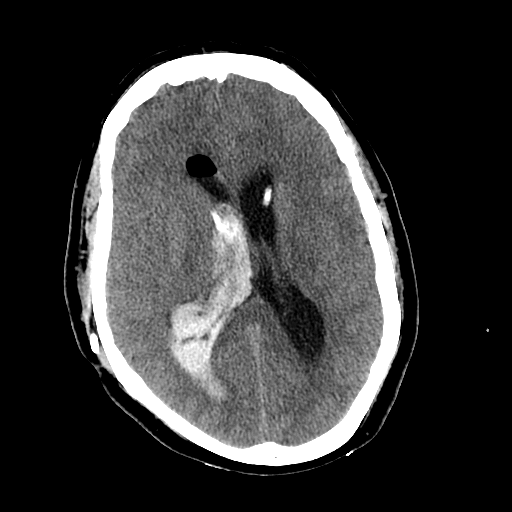

Supplement: S4 Data — (ZIP) [file pone.0295536.s005.zip › S5_Data/FCN_Training set/IM_0014-ID_1ca78597d.png]

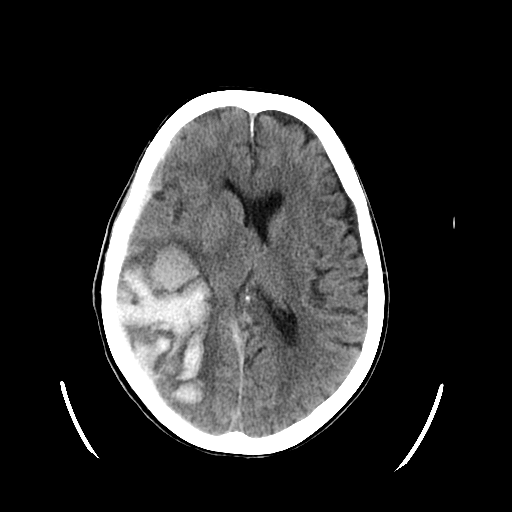

Supplement: S4 Data — (ZIP) [file pone.0295536.s005.zip › S5_Data/FCN_Training set/IM_0014-ID_1fa540939.png]

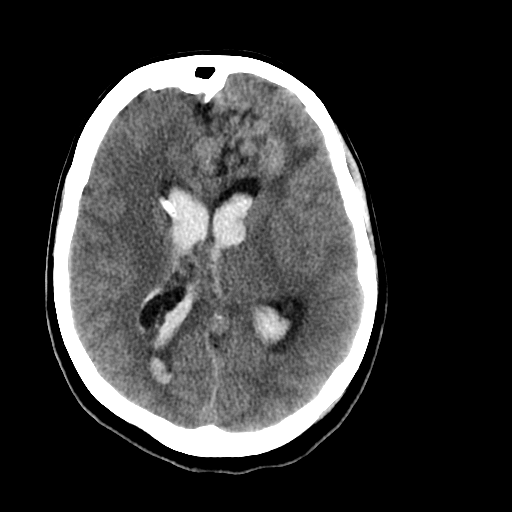

Supplement: S4 Data — (ZIP) [file pone.0295536.s005.zip › S5_Data/FCN_Training set/IM_0014-ID_2a0534532.png]

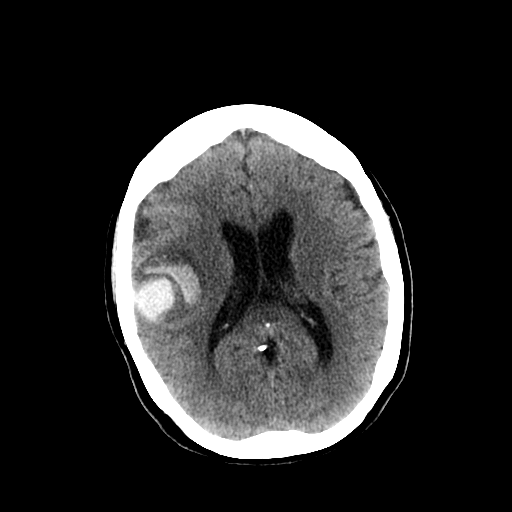

Supplement: S4 Data — (ZIP) [file pone.0295536.s005.zip › S5_Data/FCN_Training set/IM_0014-ID_2a8773709.png]

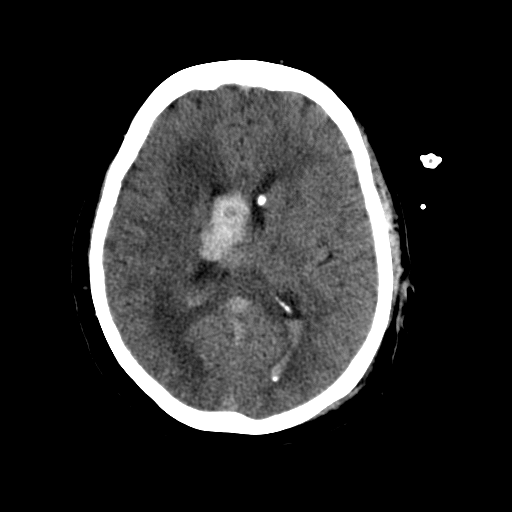

Supplement: S4 Data — (ZIP) [file pone.0295536.s005.zip › S5_Data/FCN_Training set/IM_0014-ID_2a9097225.png]

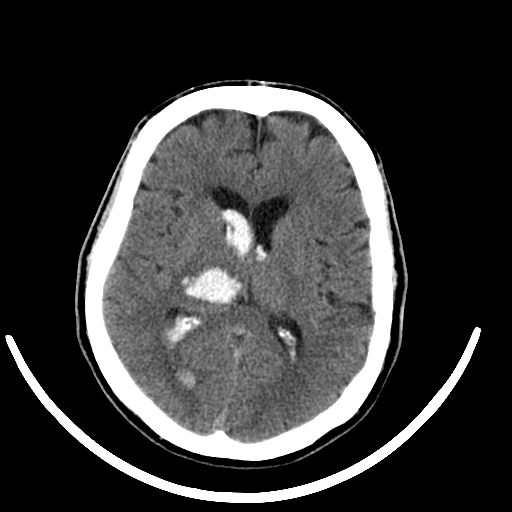

Supplement: S4 Data — (ZIP) [file pone.0295536.s005.zip › S5_Data/FCN_Training set/IM_0014-ID_2ab40a307.png]

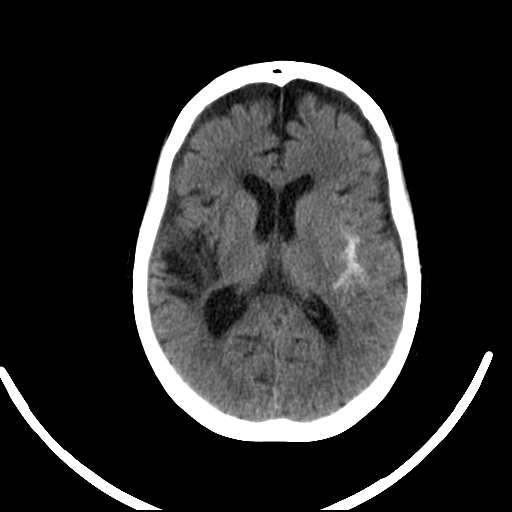

Supplement: S4 Data — (ZIP) [file pone.0295536.s005.zip › S5_Data/FCN_Training set/IM_0014-ID_2bbf72532.png]

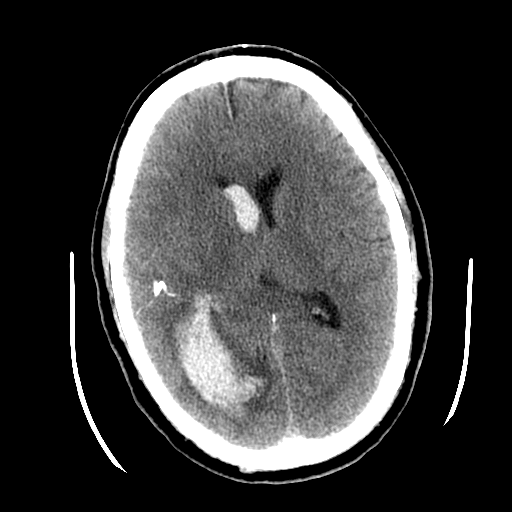

Supplement: S4 Data — (ZIP) [file pone.0295536.s005.zip › S5_Data/FCN_Training set/IM_0014-ID_2dac89820.png]

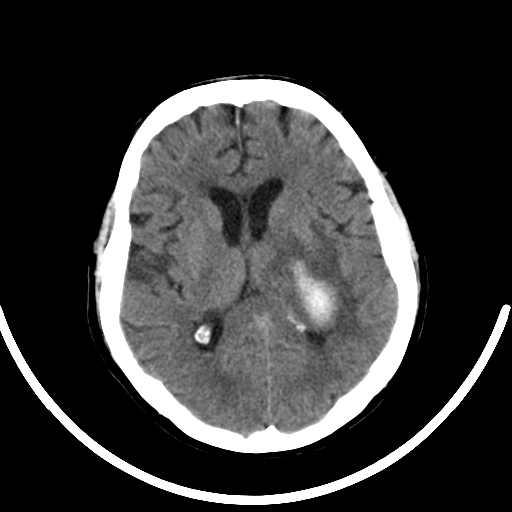

Supplement: S4 Data — (ZIP) [file pone.0295536.s005.zip › S5_Data/FCN_Training set/IM_0014-ID_2f4397a96.png]

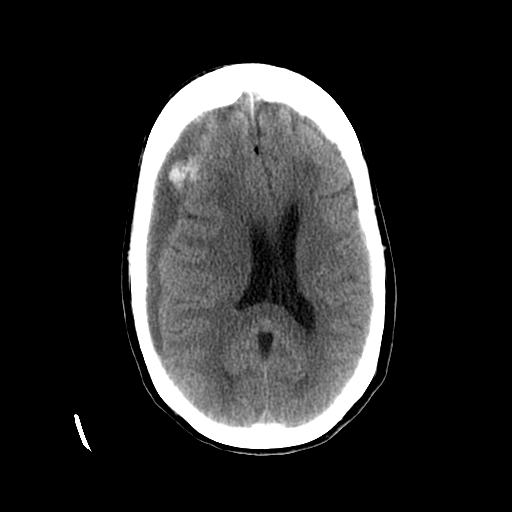

Supplement: S4 Data — (ZIP) [file pone.0295536.s005.zip › S5_Data/FCN_Training set/IM_0014-ID_3a13bacfd.png]

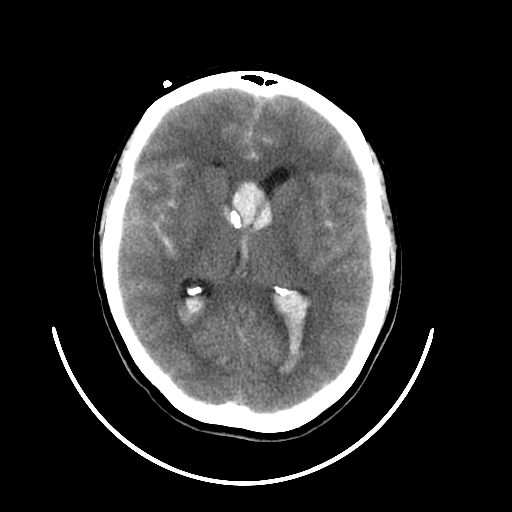

Supplement: S4 Data — (ZIP) [file pone.0295536.s005.zip › S5_Data/FCN_Training set/IM_0014-ID_3a6b8c700.png]

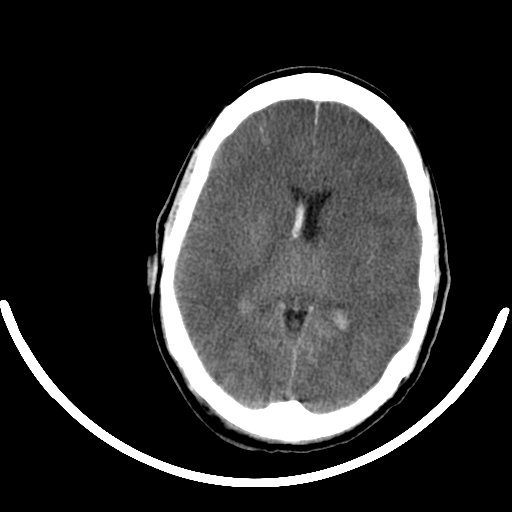

Supplement: S4 Data — (ZIP) [file pone.0295536.s005.zip › S5_Data/FCN_Training set/IM_0014-ID_3a6ca1dd0.png]

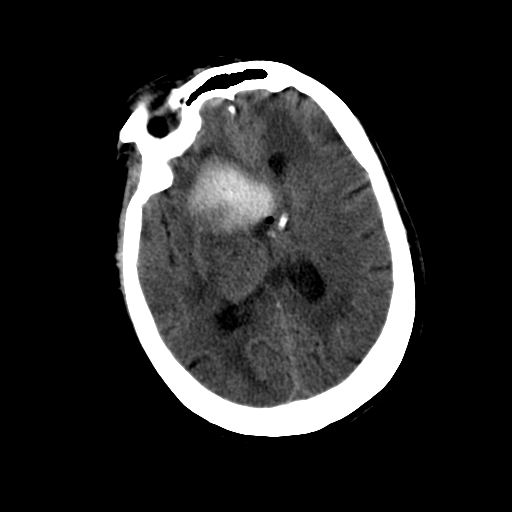

Supplement: S4 Data — (ZIP) [file pone.0295536.s005.zip › S5_Data/FCN_Training set/IM_0014-ID_3c7809a04.png]

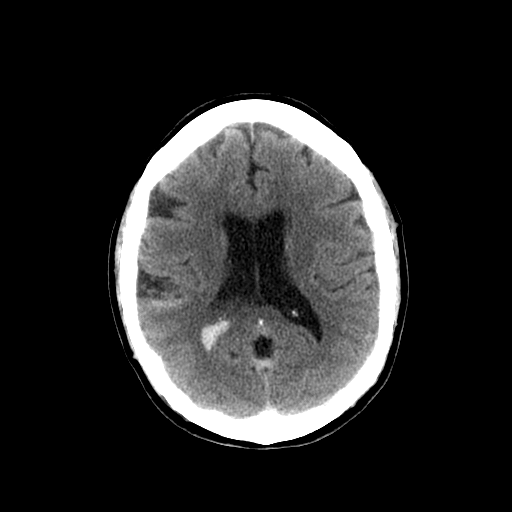

Supplement: S4 Data — (ZIP) [file pone.0295536.s005.zip › S5_Data/FCN_Training set/IM_0014-ID_3c8968f4b.png]

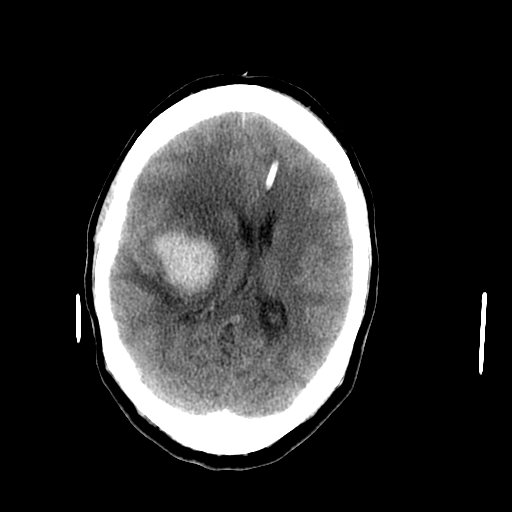

Supplement: S4 Data — (ZIP) [file pone.0295536.s005.zip › S5_Data/FCN_Training set/IM_0014-ID_3d9875df1.png]

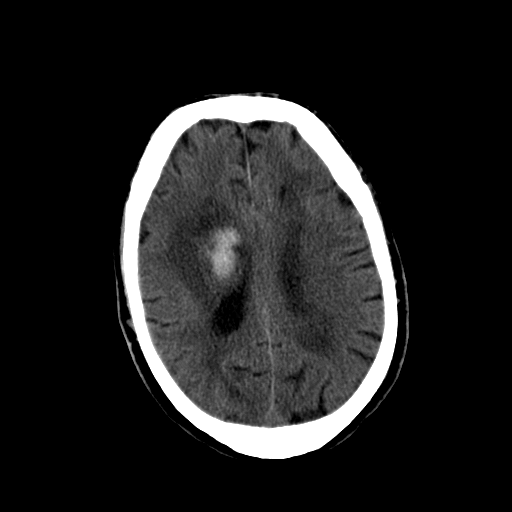

Supplement: S4 Data — (ZIP) [file pone.0295536.s005.zip › S5_Data/FCN_Training set/IM_0014-ID_3e64a669f.png]

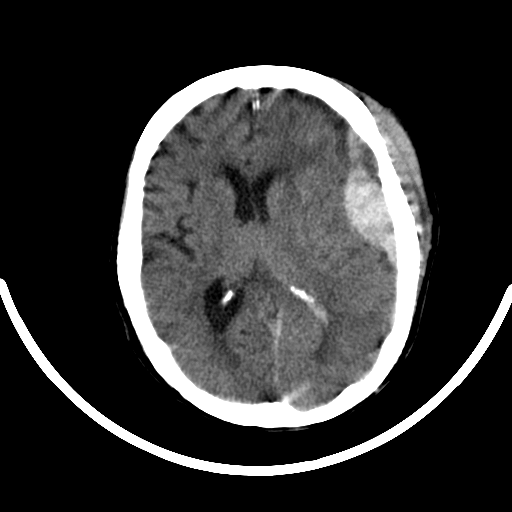

Supplement: S4 Data — (ZIP) [file pone.0295536.s005.zip › S5_Data/FCN_Training set/IM_0014-ID_3edfebdf0.png]

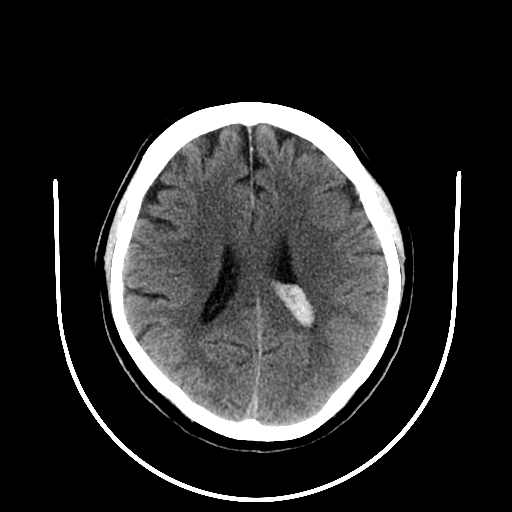

Supplement: S4 Data — (ZIP) [file pone.0295536.s005.zip › S5_Data/FCN_Training set/IM_0014-ID_3f6ff50bd.png]

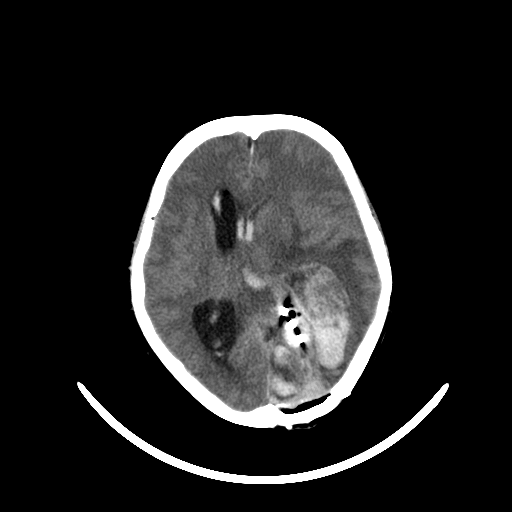

Supplement: S4 Data — (ZIP) [file pone.0295536.s005.zip › S5_Data/FCN_Training set/IM_0014-ID_4a0ce01df.png]

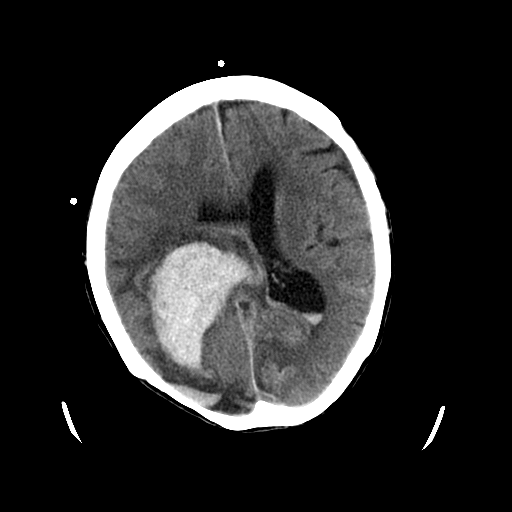

Supplement: S4 Data — (ZIP) [file pone.0295536.s005.zip › S5_Data/FCN_Training set/IM_0014-ID_4ab147506.png]

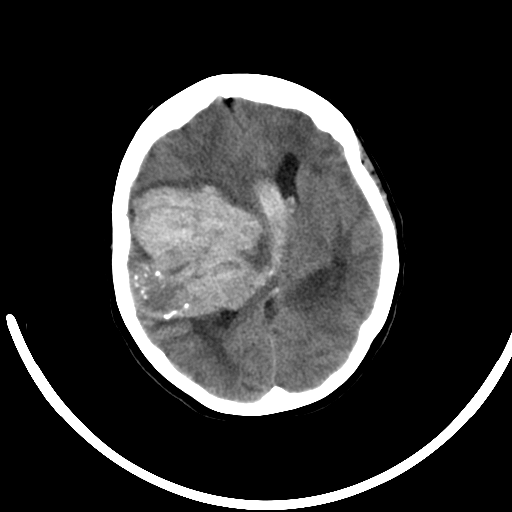

Supplement: S4 Data — (ZIP) [file pone.0295536.s005.zip › S5_Data/FCN_Training set/IM_0014-ID_4c439aa2d.png]

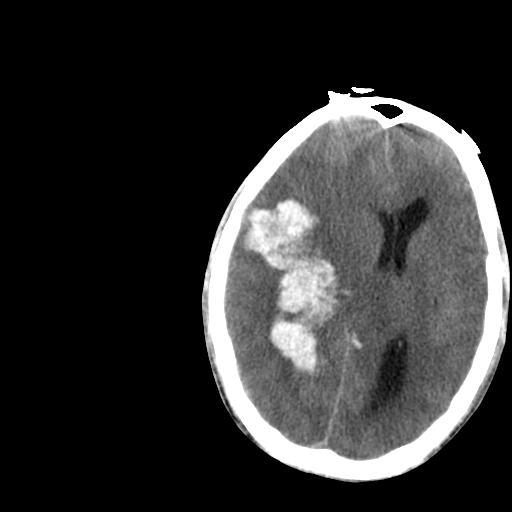

Supplement: S4 Data — (ZIP) [file pone.0295536.s005.zip › S5_Data/FCN_Training set/IM_0014-ID_4cdb33c1e.png]

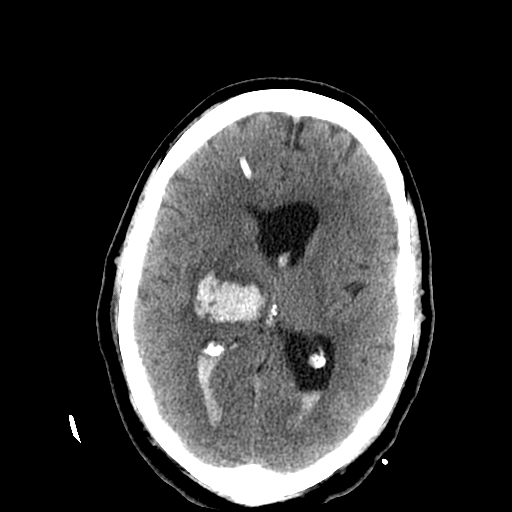

Supplement: S4 Data — (ZIP) [file pone.0295536.s005.zip › S5_Data/FCN_Training set/IM_0014-ID_4e146c17e.png]

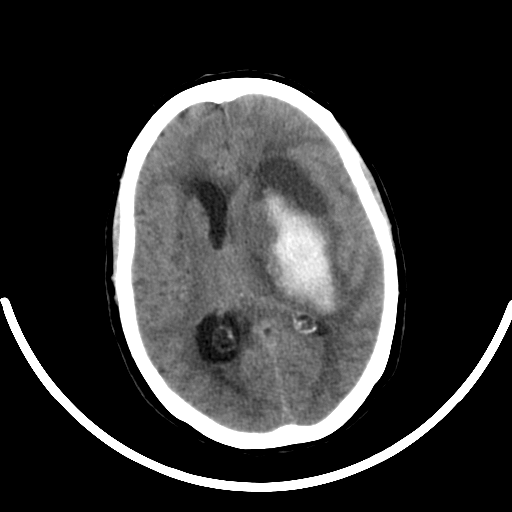

Supplement: S4 Data — (ZIP) [file pone.0295536.s005.zip › S5_Data/FCN_Training set/IM_0014-ID_4e6597289.png]

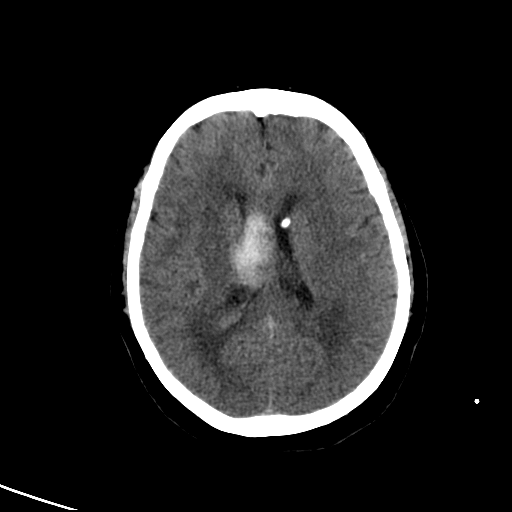

Supplement: S4 Data — (ZIP) [file pone.0295536.s005.zip › S5_Data/FCN_Training set/IM_0014-ID_4faa47a89.png]

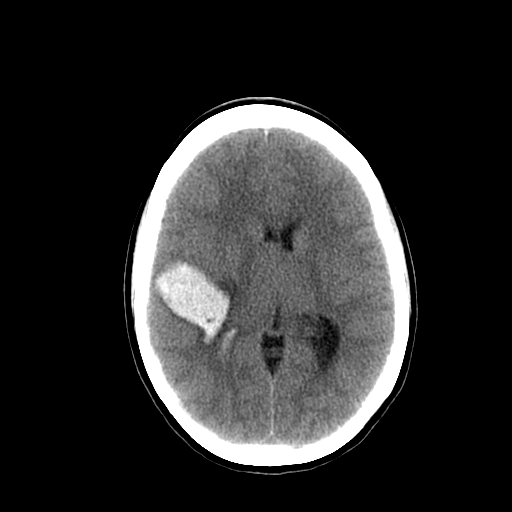

Supplement: S4 Data — (ZIP) [file pone.0295536.s005.zip › S5_Data/FCN_Training set/IM_0014-ID_5c825301e.png]

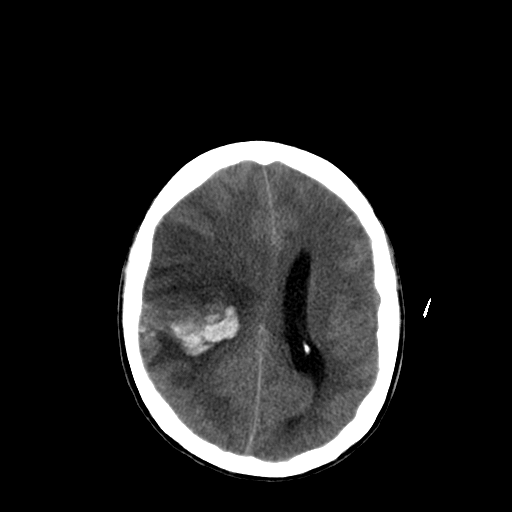

Supplement: S4 Data — (ZIP) [file pone.0295536.s005.zip › S5_Data/FCN_Training set/IM_0014-ID_5c962a6f6.png]

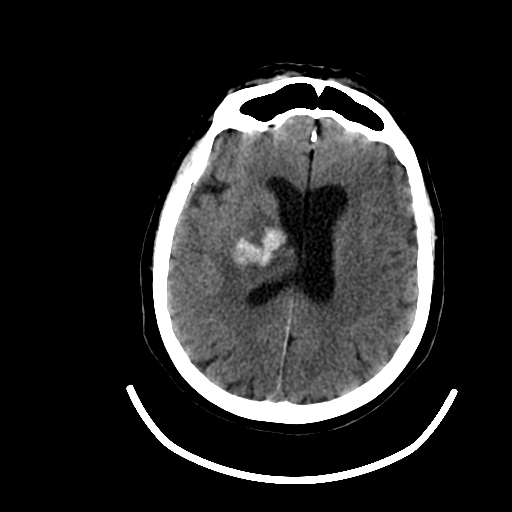

Supplement: S4 Data — (ZIP) [file pone.0295536.s005.zip › S5_Data/FCN_Training set/IM_0014-ID_5d3f5d41c.png]

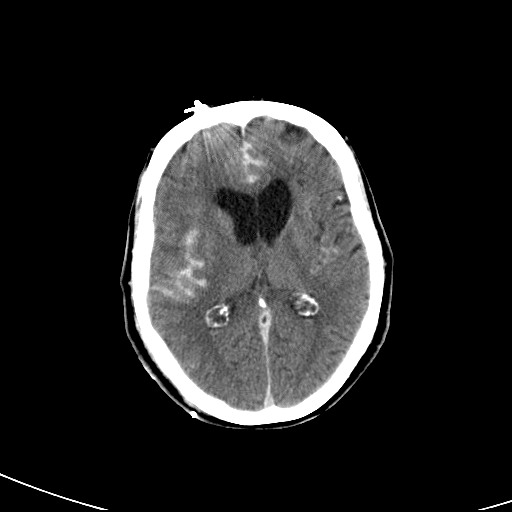

Supplement: S4 Data — (ZIP) [file pone.0295536.s005.zip › S5_Data/FCN_Training set/IM_0014-ID_5d64cb00a.png]

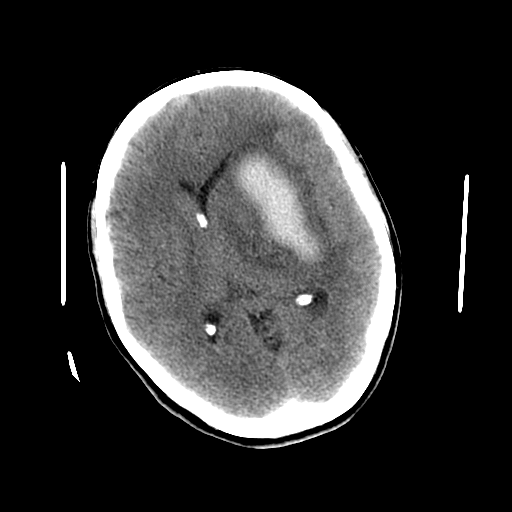

Supplement: S4 Data — (ZIP) [file pone.0295536.s005.zip › S5_Data/FCN_Training set/IM_0014-ID_5eb946f10.png]

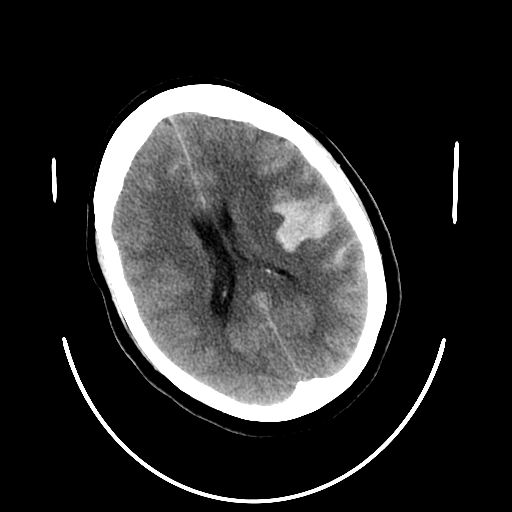

Supplement: S4 Data — (ZIP) [file pone.0295536.s005.zip › S5_Data/FCN_Training set/IM_0014-ID_6c7700e99.png]

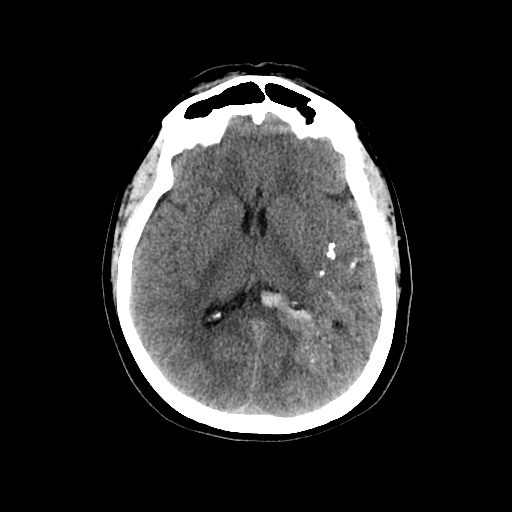

Supplement: S4 Data — (ZIP) [file pone.0295536.s005.zip › S5_Data/FCN_Training set/IM_0014-ID_6cdeaaabf.png]

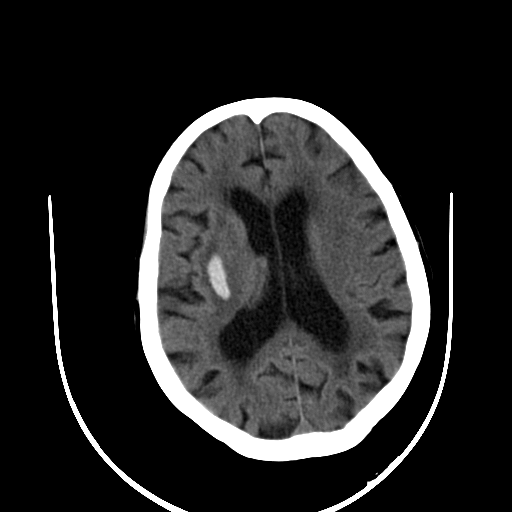

Supplement: S4 Data — (ZIP) [file pone.0295536.s005.zip › S5_Data/FCN_Training set/IM_0014-ID_6d142f416.png]

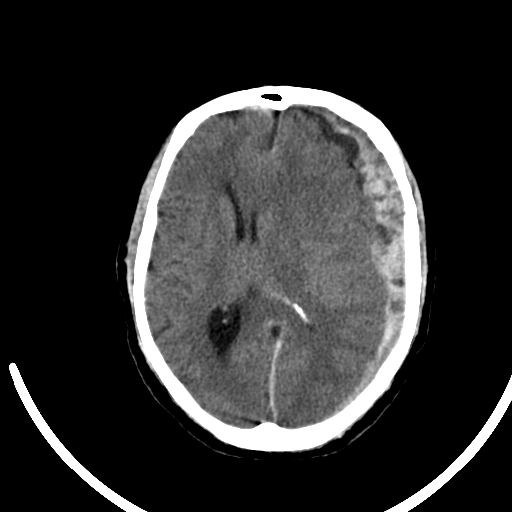

Supplement: S4 Data — (ZIP) [file pone.0295536.s005.zip › S5_Data/FCN_Training set/IM_0014-ID_6d546d96d.png]

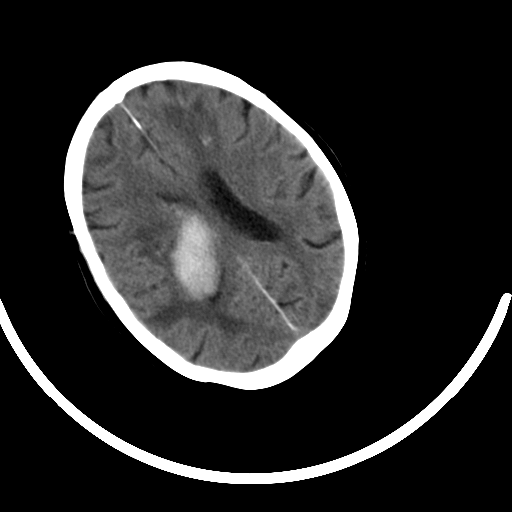

Supplement: S4 Data — (ZIP) [file pone.0295536.s005.zip › S5_Data/FCN_Training set/IM_0014-ID_6d9b12d43.png]

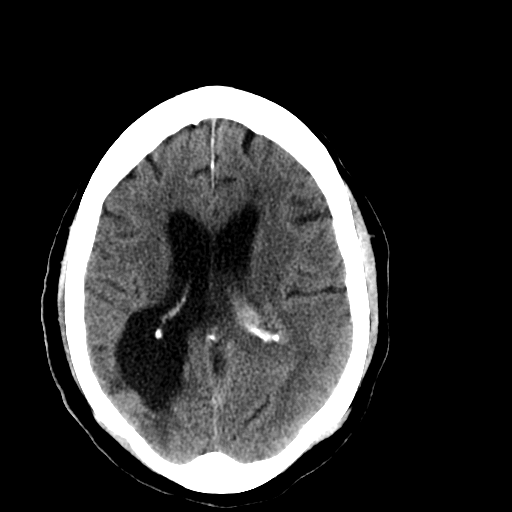

Supplement: S4 Data — (ZIP) [file pone.0295536.s005.zip › S5_Data/FCN_Training set/IM_0014-ID_6df03ab36.png]

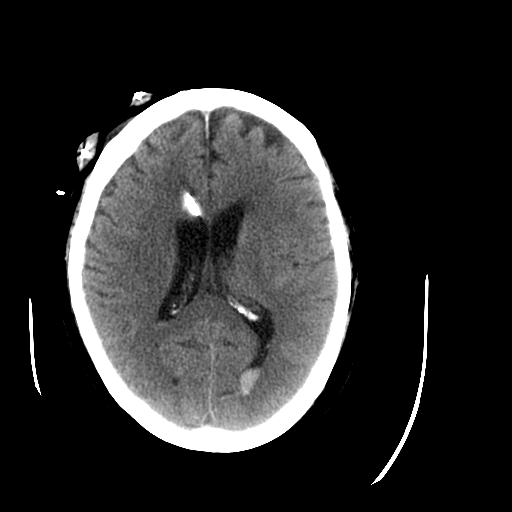

Supplement: S4 Data — (ZIP) [file pone.0295536.s005.zip › S5_Data/FCN_Training set/IM_0014-ID_6df42c874.png]

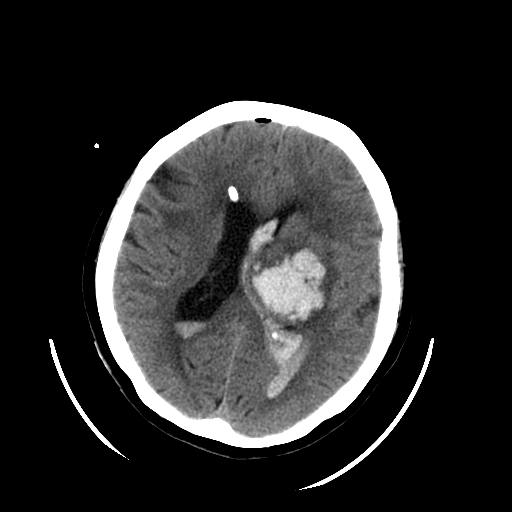

Supplement: S4 Data — (ZIP) [file pone.0295536.s005.zip › S5_Data/FCN_Training set/IM_0014-ID_6e4b61ee0.png]

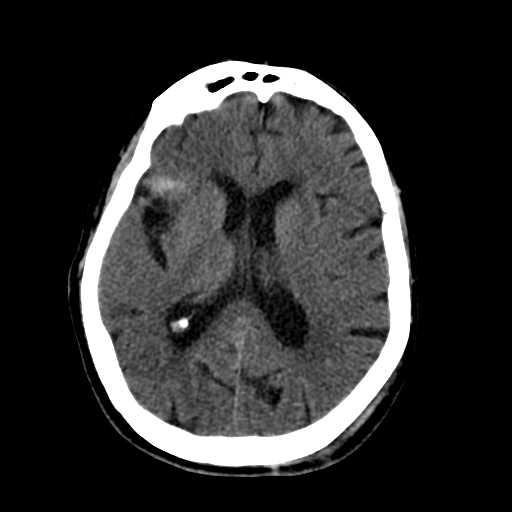

Supplement: S4 Data — (ZIP) [file pone.0295536.s005.zip › S5_Data/FCN_Training set/IM_0014-ID_6e6b61785.png]

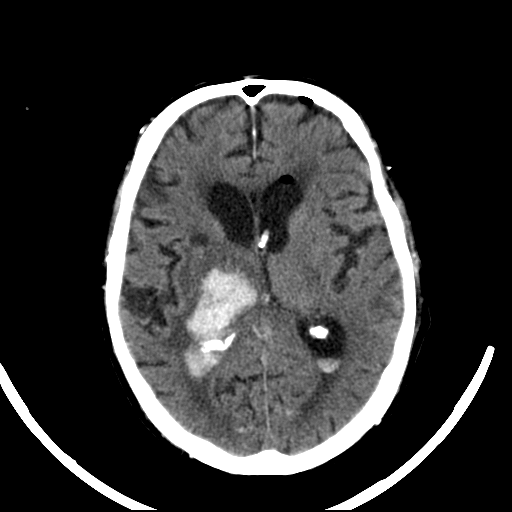

Supplement: S4 Data — (ZIP) [file pone.0295536.s005.zip › S5_Data/FCN_Training set/IM_0014-ID_6eaf6e207.png]

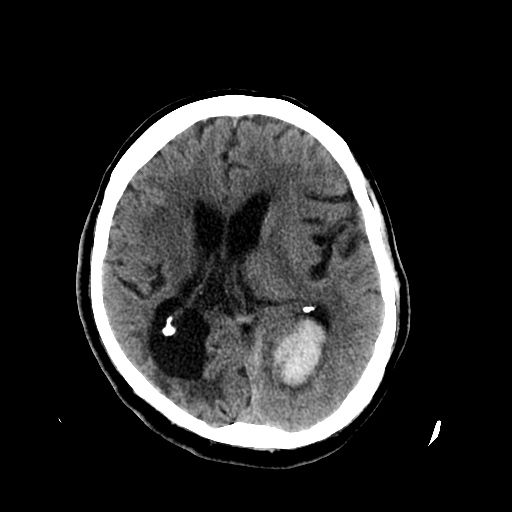

Supplement: S4 Data — (ZIP) [file pone.0295536.s005.zip › S5_Data/FCN_Training set/IM_0014-ID_7a6c2aa44.png]

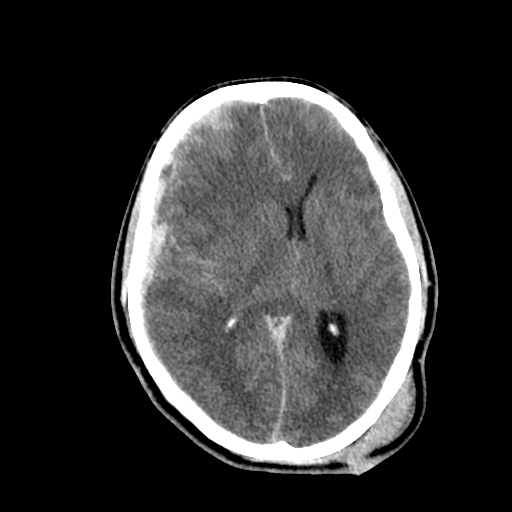

Supplement: S4 Data — (ZIP) [file pone.0295536.s005.zip › S5_Data/FCN_Training set/IM_0014-ID_7ad15a98a.png]

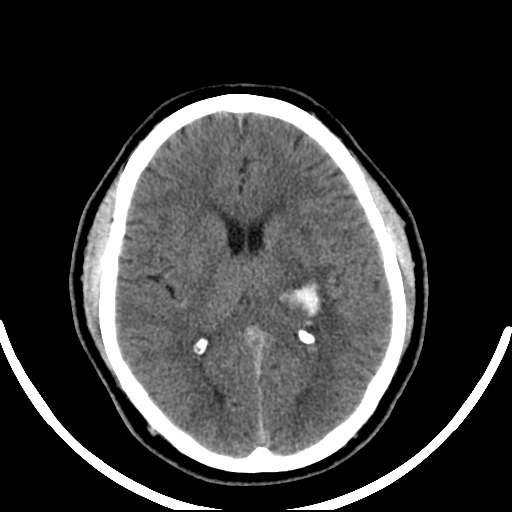

Supplement: S4 Data — (ZIP) [file pone.0295536.s005.zip › S5_Data/FCN_Training set/IM_0014-ID_7cb5f8516.png]

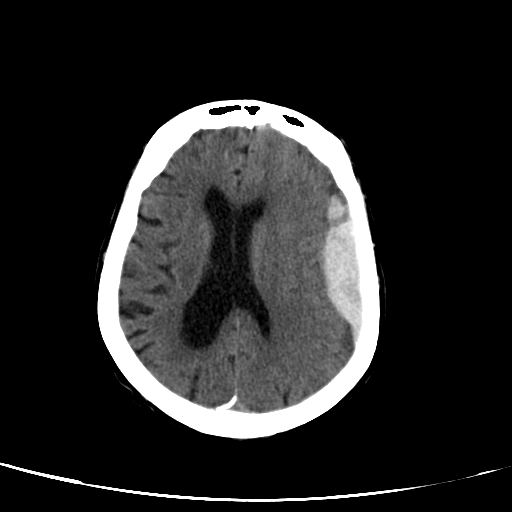

Supplement: S4 Data — (ZIP) [file pone.0295536.s005.zip › S5_Data/FCN_Training set/IM_0014-ID_7df773e3b.png]

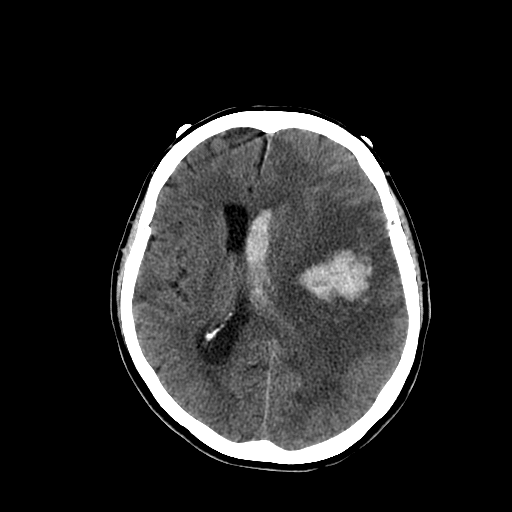

Supplement: S4 Data — (ZIP) [file pone.0295536.s005.zip › S5_Data/FCN_Training set/IM_0014-ID_7fe270e60.png]

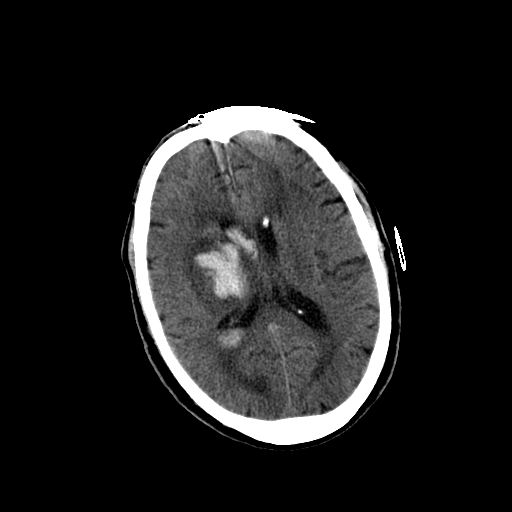

Supplement: S4 Data — (ZIP) [file pone.0295536.s005.zip › S5_Data/FCN_Training set/IM_0014-ID_8a8d1251d.png]

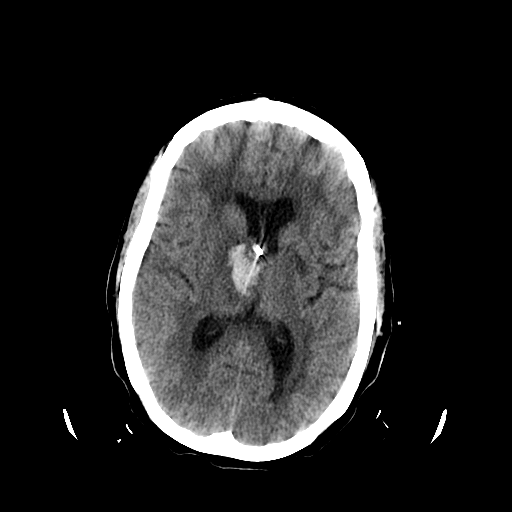

Supplement: S4 Data — (ZIP) [file pone.0295536.s005.zip › S5_Data/FCN_Training set/IM_0014-ID_8b79e2c98.png]

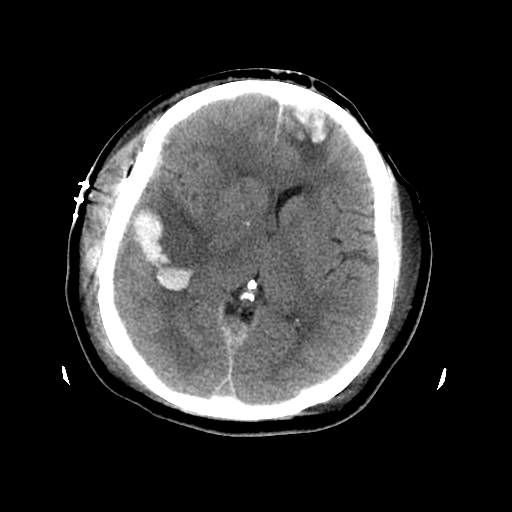

Supplement: S4 Data — (ZIP) [file pone.0295536.s005.zip › S5_Data/FCN_Training set/IM_0014-ID_8bd37d387.png]

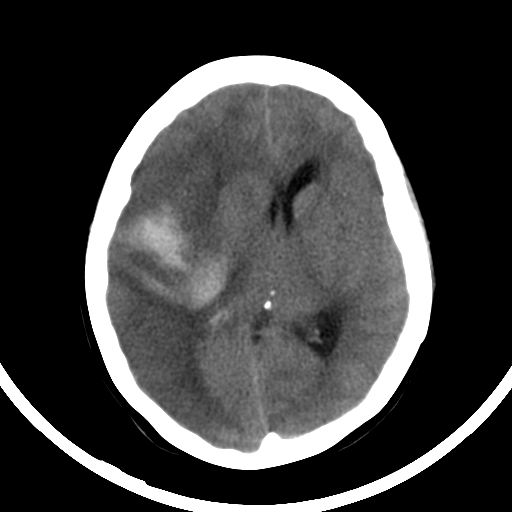

Supplement: S4 Data — (ZIP) [file pone.0295536.s005.zip › S5_Data/FCN_Training set/IM_0014-ID_8bd7251fd.png]

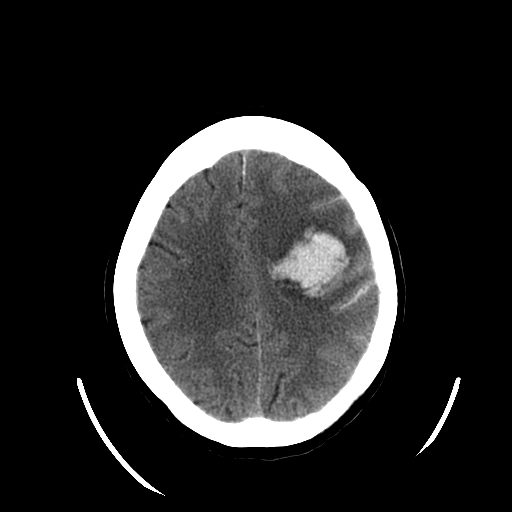

Supplement: S4 Data — (ZIP) [file pone.0295536.s005.zip › S5_Data/FCN_Training set/IM_0016-ID_444735a2f.png]

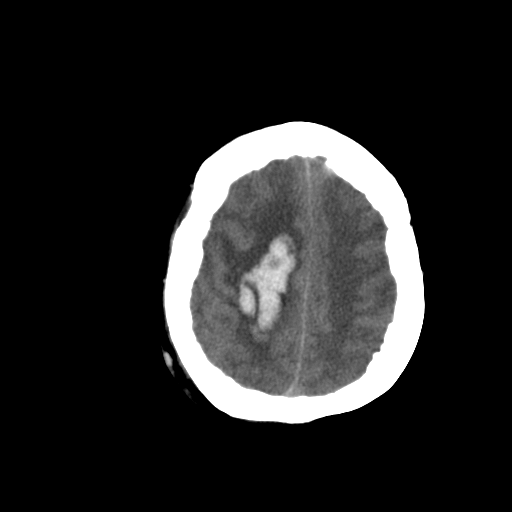

Supplement: S4 Data — (ZIP) [file pone.0295536.s005.zip › S5_Data/FCN_Training set/IM_0016-ID_50834e85a.png]

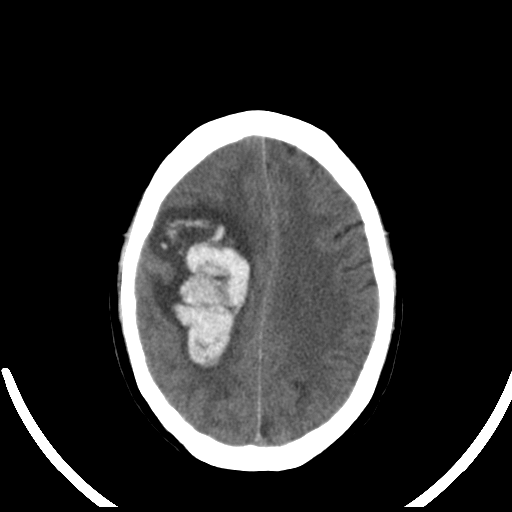

Supplement: S4 Data — (ZIP) [file pone.0295536.s005.zip › S5_Data/FCN_Training set/IM_0016-ID_581464f53.png]

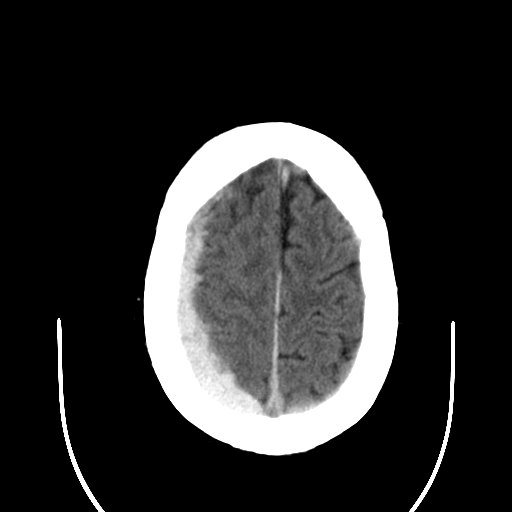

Supplement: S4 Data — (ZIP) [file pone.0295536.s005.zip › S5_Data/FCN_Training set/IM_0016-ID_83222b2c8.png]
