# Supplementary figures and images for: Adaptive mask-based brain extraction method for head CT images (part 10 of 14)
Source: PLoS One. 2024 Mar 11;19(3):e0295536. doi: 10.1371/journal.pone.0295536 (PMC10927156; doi:10.1371/journal.pone.0295536)

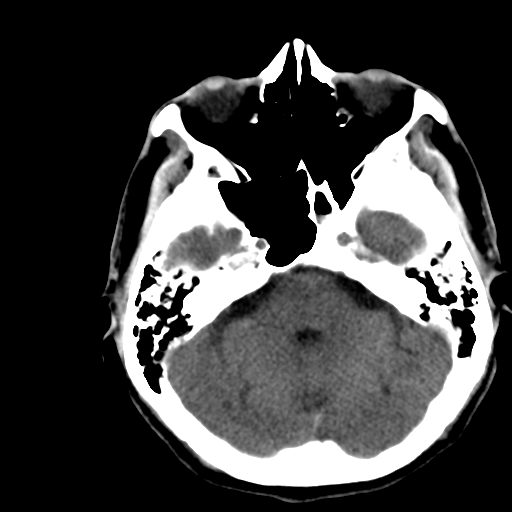

Supplement: S5 Data — (ZIP) [file pone.0295536.s006.zip › S6_Data/Tset set 1/3/125IM_0011-ID_8acea0a21.png]

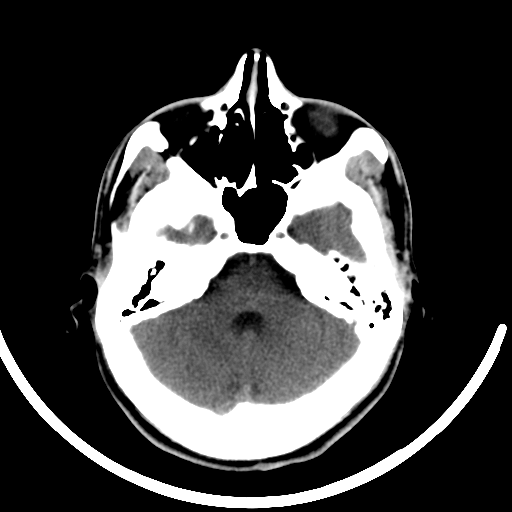

Supplement: S5 Data — (ZIP) [file pone.0295536.s006.zip › S6_Data/Tset set 1/3/126IM_0018-ID_f94b47e72.png]

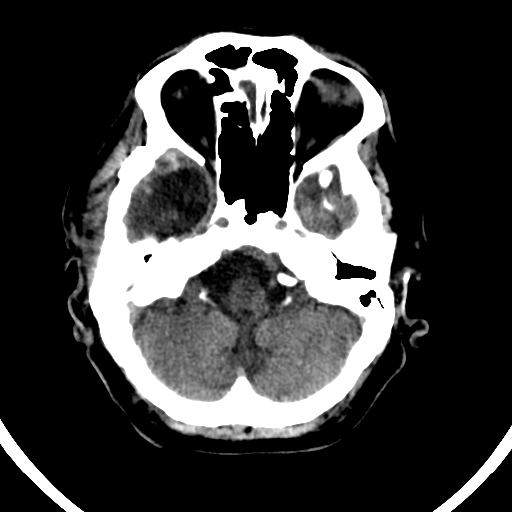

Supplement: S5 Data — (ZIP) [file pone.0295536.s006.zip › S6_Data/Tset set 1/3/13IM_0005-ID_881228d26.png]

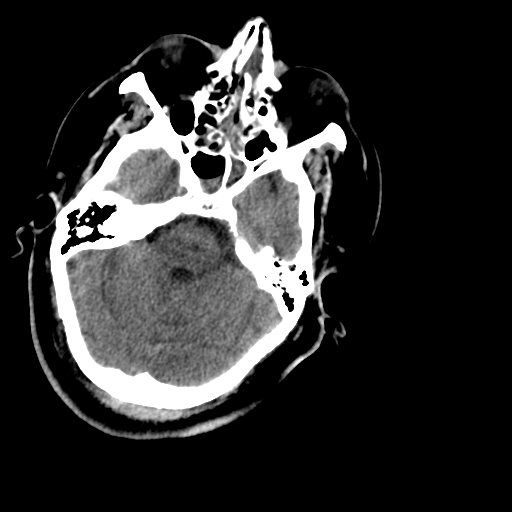

Supplement: S5 Data — (ZIP) [file pone.0295536.s006.zip › S6_Data/Tset set 1/3/14IM_0007-ID_19dab20cd.png]

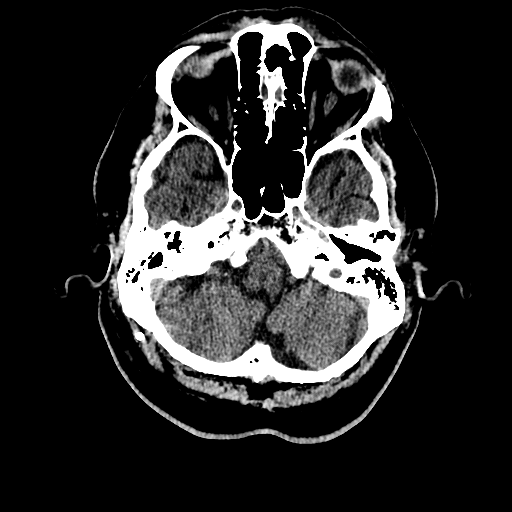

Supplement: S5 Data — (ZIP) [file pone.0295536.s006.zip › S6_Data/Tset set 1/3/15IM_0006-ID_c8aa6abb1.png]

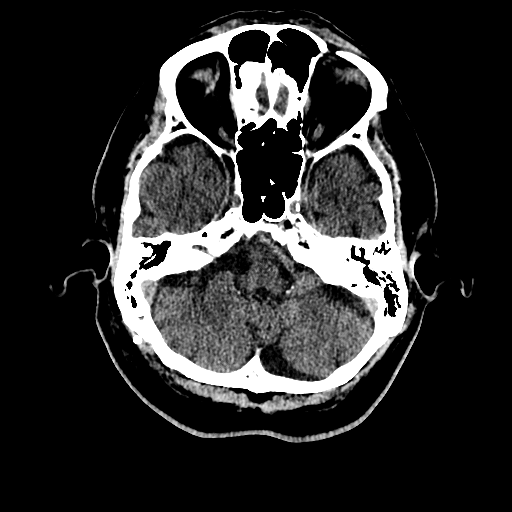

Supplement: S5 Data — (ZIP) [file pone.0295536.s006.zip › S6_Data/Tset set 1/3/15IM_0008-ID_9574f11b7.png]

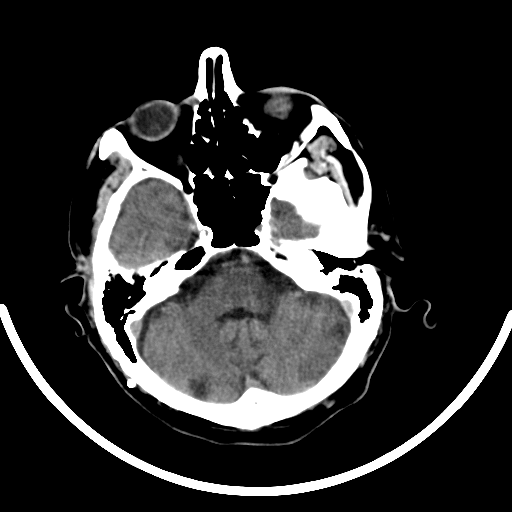

Supplement: S5 Data — (ZIP) [file pone.0295536.s006.zip › S6_Data/Tset set 1/3/16IM_0008-ID_cca9fe4b5.png]

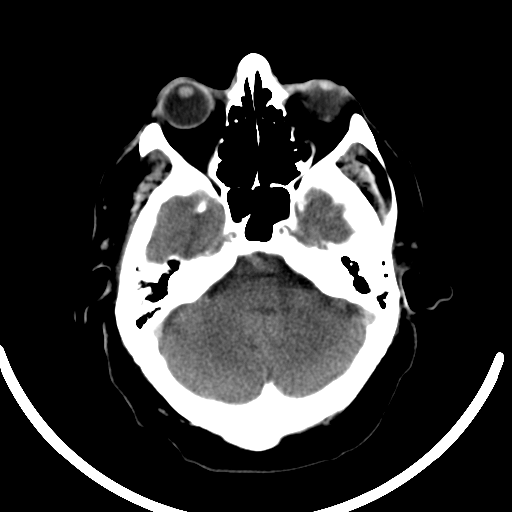

Supplement: S5 Data — (ZIP) [file pone.0295536.s006.zip › S6_Data/Tset set 1/3/5IM_0014-ID_0f069de52.png]

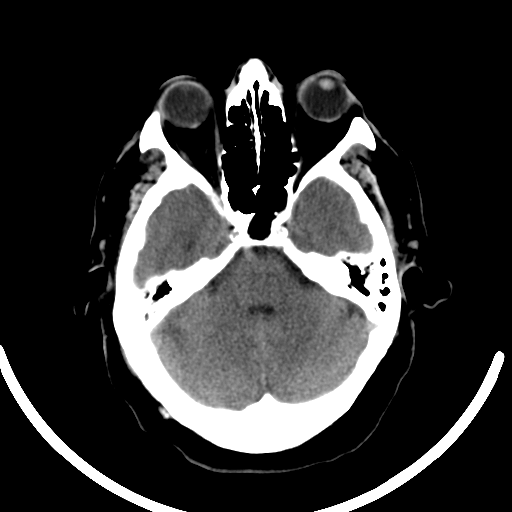

Supplement: S5 Data — (ZIP) [file pone.0295536.s006.zip › S6_Data/Tset set 1/3/5IM_0015-ID_49d97de2d.png]

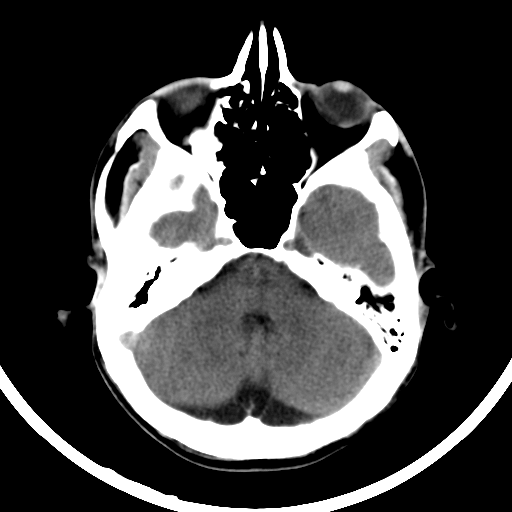

Supplement: S5 Data — (ZIP) [file pone.0295536.s006.zip › S6_Data/Tset set 1/3/7IM_0009-ID_535746734.png]

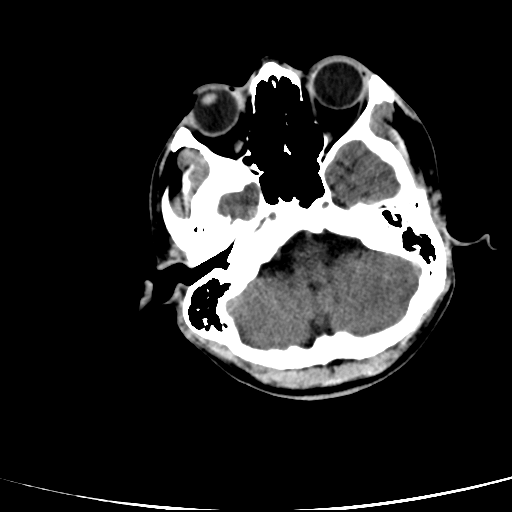

Supplement: S5 Data — (ZIP) [file pone.0295536.s006.zip › S6_Data/Tset set 1/3/8IM_0004-ID_2210c5c20.png]

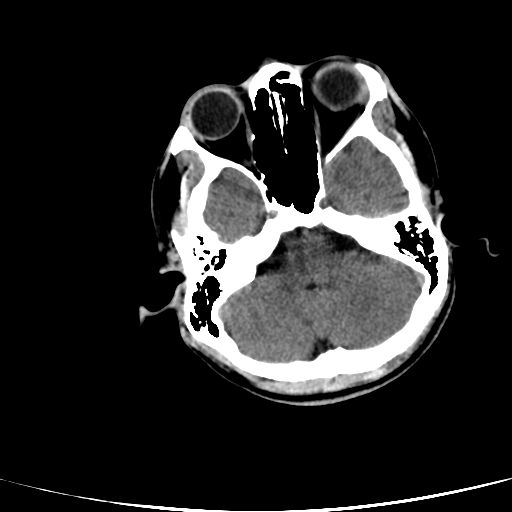

Supplement: S5 Data — (ZIP) [file pone.0295536.s006.zip › S6_Data/Tset set 1/3/8IM_0005-ID_066c3cbb3.png]

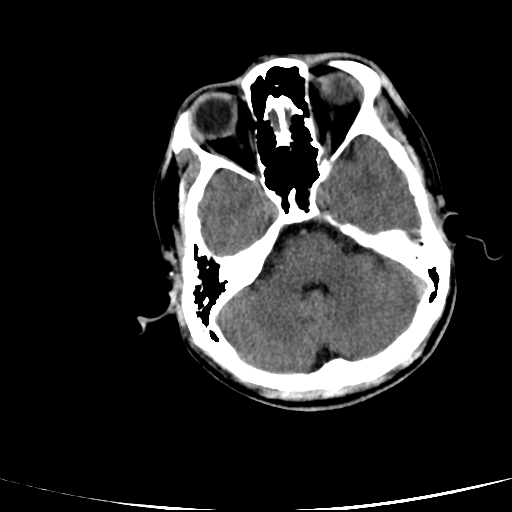

Supplement: S5 Data — (ZIP) [file pone.0295536.s006.zip › S6_Data/Tset set 1/3/8IM_0006-ID_1ba3f1ee3.png]

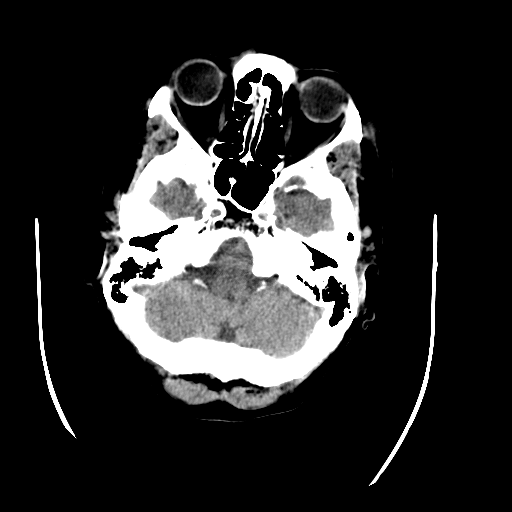

Supplement: S5 Data — (ZIP) [file pone.0295536.s006.zip › S6_Data/Tset set 1/3/IM_0003-ID_c498f995b.png]

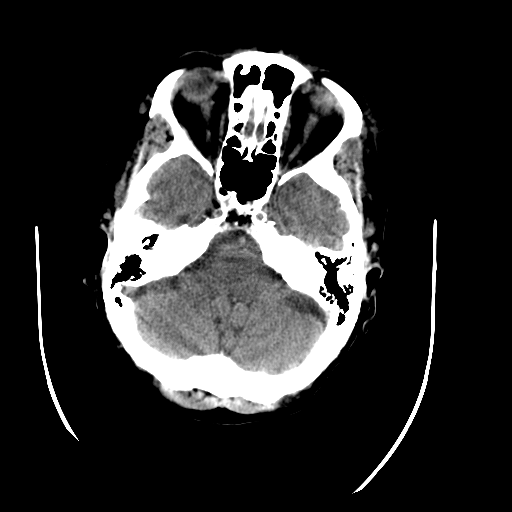

Supplement: S5 Data — (ZIP) [file pone.0295536.s006.zip › S6_Data/Tset set 1/3/IM_0004-ID_9d9d70b8f.png]

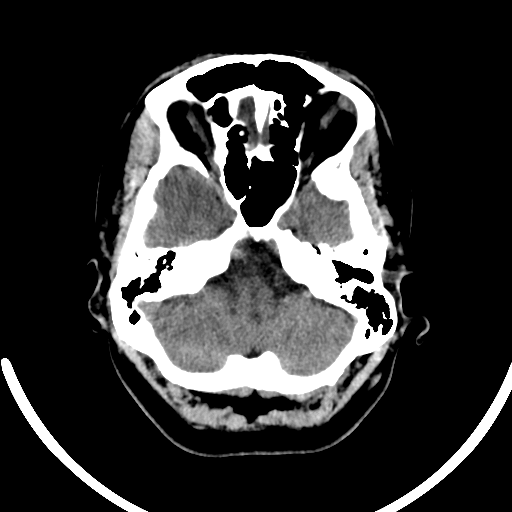

Supplement: S5 Data — (ZIP) [file pone.0295536.s006.zip › S6_Data/Tset set 1/3/IM_0005-ID_9bbaad1fc.png]

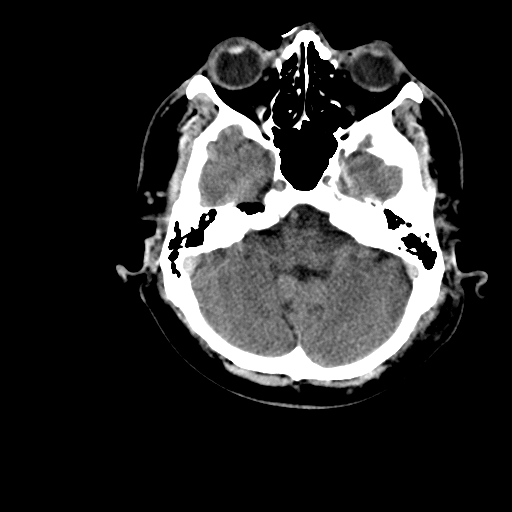

Supplement: S5 Data — (ZIP) [file pone.0295536.s006.zip › S6_Data/Tset set 1/3/IM_0005-ID_b38df351e.png]

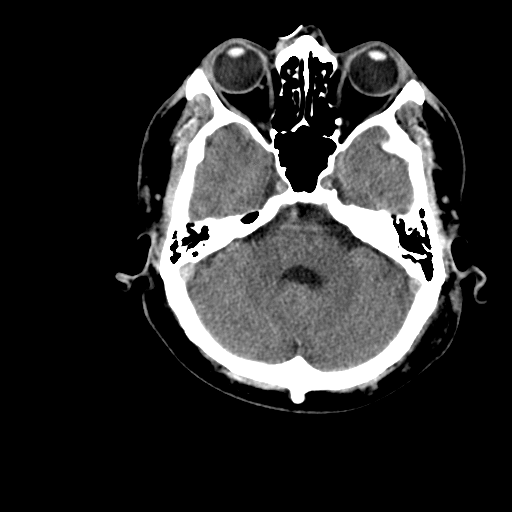

Supplement: S5 Data — (ZIP) [file pone.0295536.s006.zip › S6_Data/Tset set 1/3/IM_0006-ID_246d8ddd5.png]

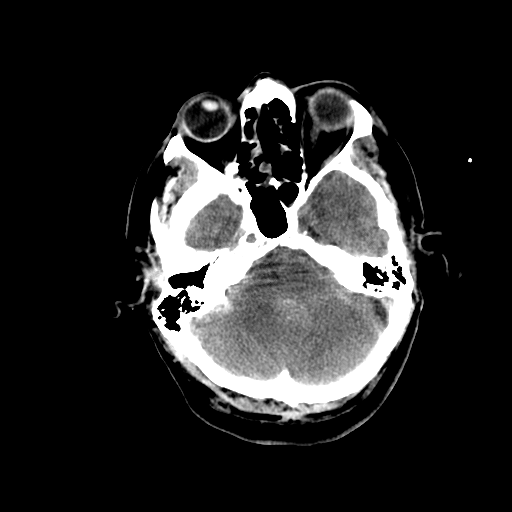

Supplement: S5 Data — (ZIP) [file pone.0295536.s006.zip › S6_Data/Tset set 1/3/IM_0006-ID_bdf973a02.png]

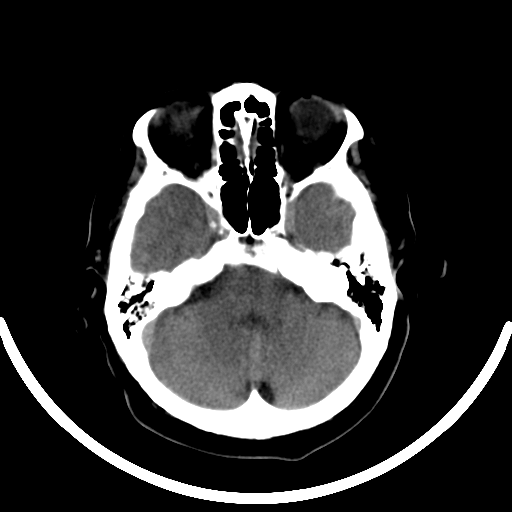

Supplement: S5 Data — (ZIP) [file pone.0295536.s006.zip › S6_Data/Tset set 1/3/IM_0008-ID_03bb456c2.png]

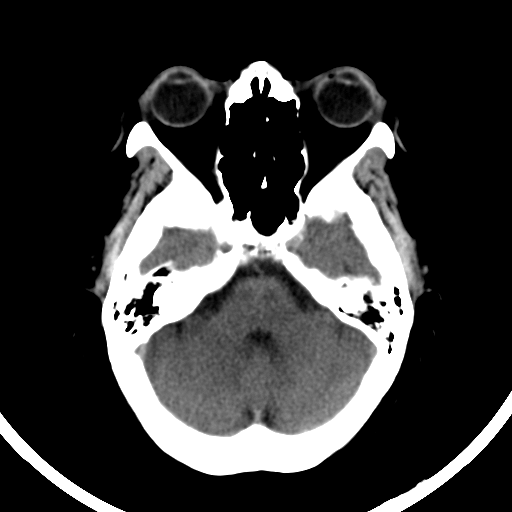

Supplement: S5 Data — (ZIP) [file pone.0295536.s006.zip › S6_Data/Tset set 1/3/IM_0008-ID_64bf53557.png]

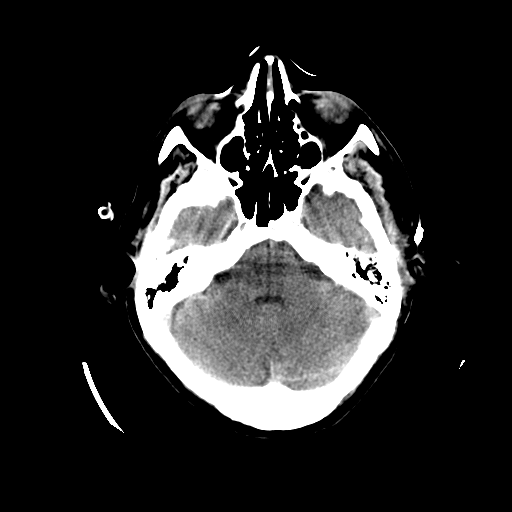

Supplement: S5 Data — (ZIP) [file pone.0295536.s006.zip › S6_Data/Tset set 1/3/IM_0008-ID_cc382f74b.png]

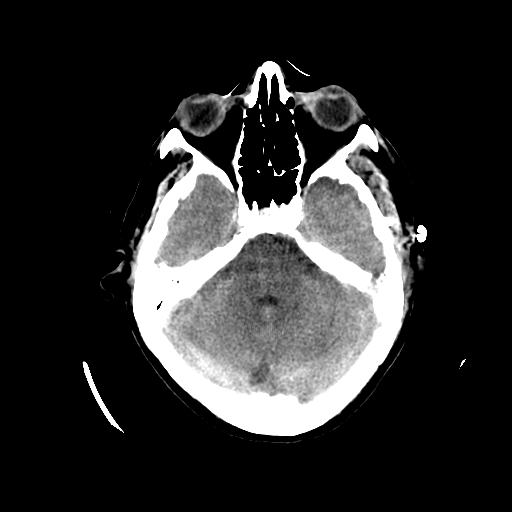

Supplement: S5 Data — (ZIP) [file pone.0295536.s006.zip › S6_Data/Tset set 1/3/IM_0009-ID_498a9785c.png]

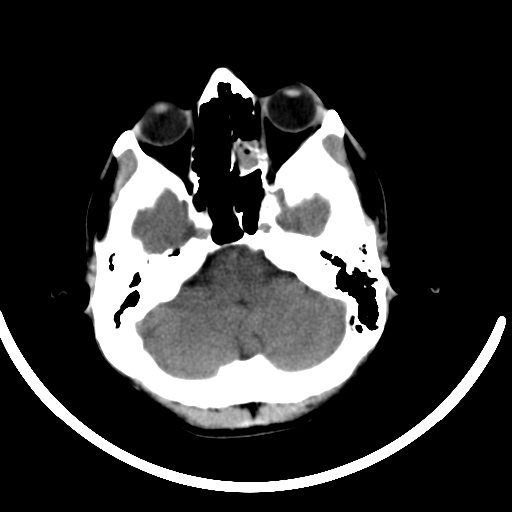

Supplement: S5 Data — (ZIP) [file pone.0295536.s006.zip › S6_Data/Tset set 1/3/IM_0014-ID_2bf278df9.png]

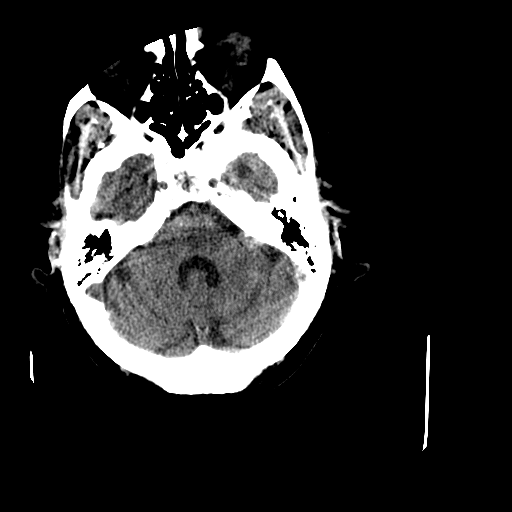

Supplement: S5 Data — (ZIP) [file pone.0295536.s006.zip › S6_Data/Tset set 1/3/IM_0017-ID_5a8c098f2.png]

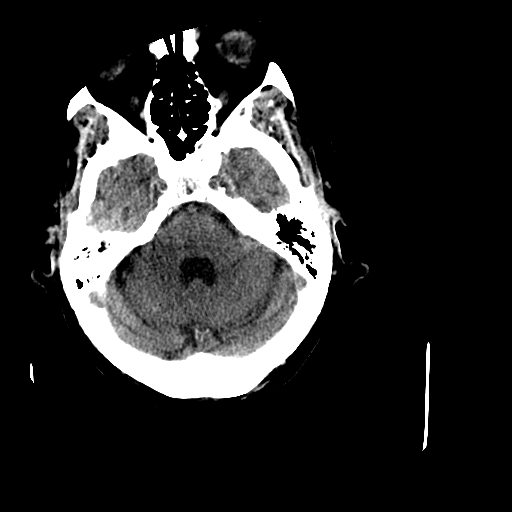

Supplement: S5 Data — (ZIP) [file pone.0295536.s006.zip › S6_Data/Tset set 1/3/IM_0019-ID_56c2edba1.png]

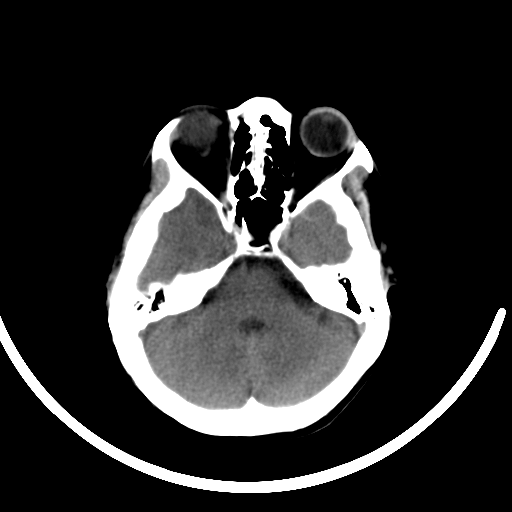

Supplement: S5 Data — (ZIP) [file pone.0295536.s006.zip › S6_Data/Tset set 1/3/IM_0021-ID_6cf0cbcb8.png]

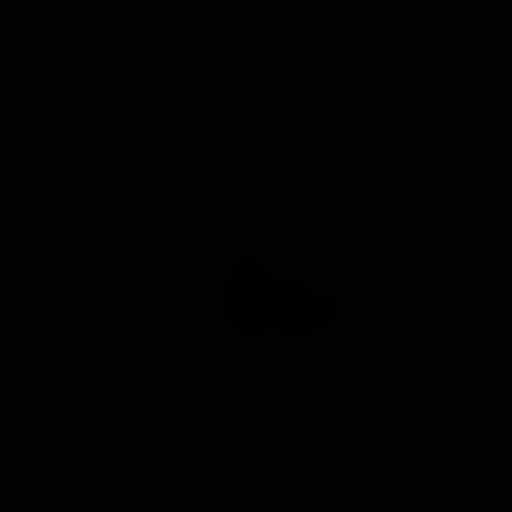

Supplement: S6 Data — (ZIP) [file pone.0295536.s007.zip › S7_Data/Tset set 2/Label/Label_1.png]

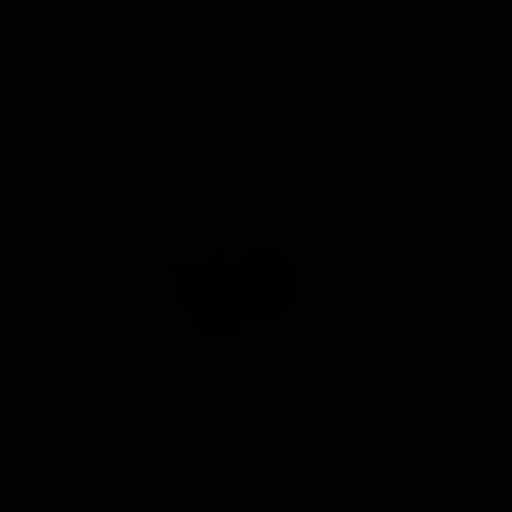

Supplement: S6 Data — (ZIP) [file pone.0295536.s007.zip › S7_Data/Tset set 2/Label/Label_10.png]

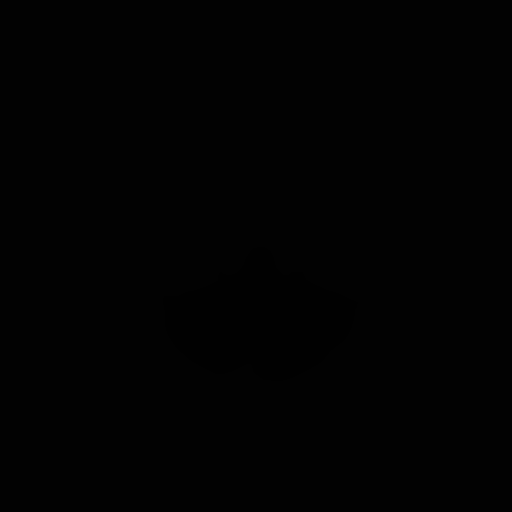

Supplement: S6 Data — (ZIP) [file pone.0295536.s007.zip › S7_Data/Tset set 2/Label/Label_100.png]

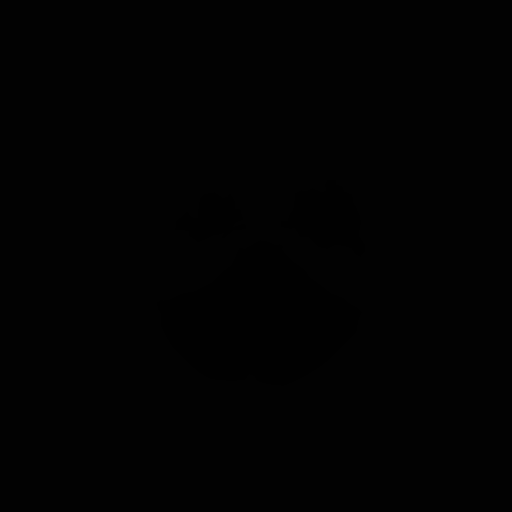

Supplement: S6 Data — (ZIP) [file pone.0295536.s007.zip › S7_Data/Tset set 2/Label/Label_101.png]

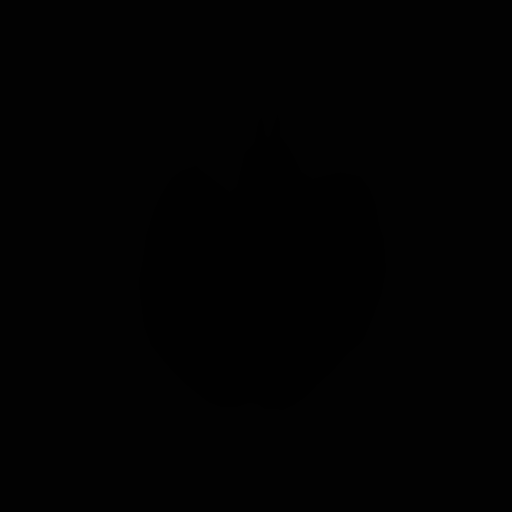

Supplement: S6 Data — (ZIP) [file pone.0295536.s007.zip › S7_Data/Tset set 2/Label/Label_102.png]

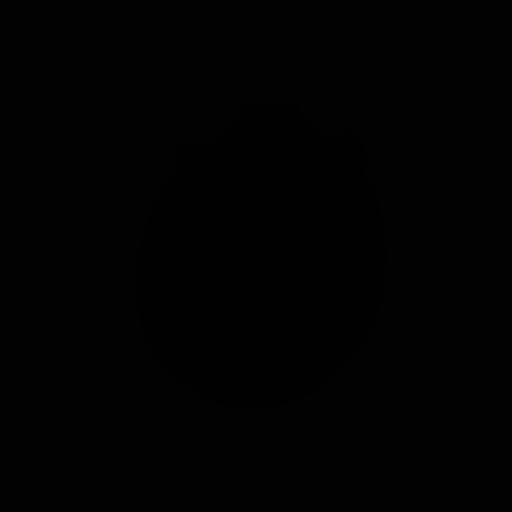

Supplement: S6 Data — (ZIP) [file pone.0295536.s007.zip › S7_Data/Tset set 2/Label/Label_103.png]

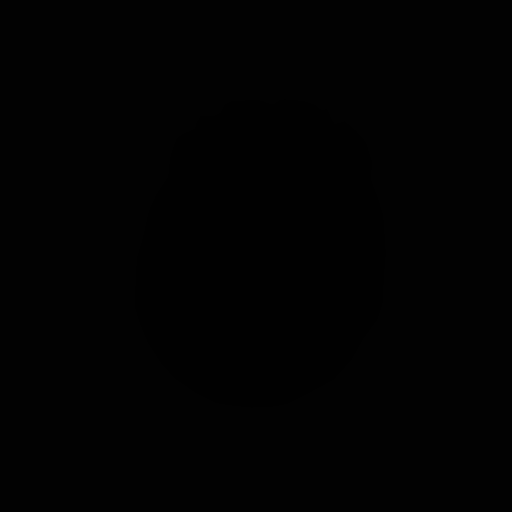

Supplement: S6 Data — (ZIP) [file pone.0295536.s007.zip › S7_Data/Tset set 2/Label/Label_104.png]

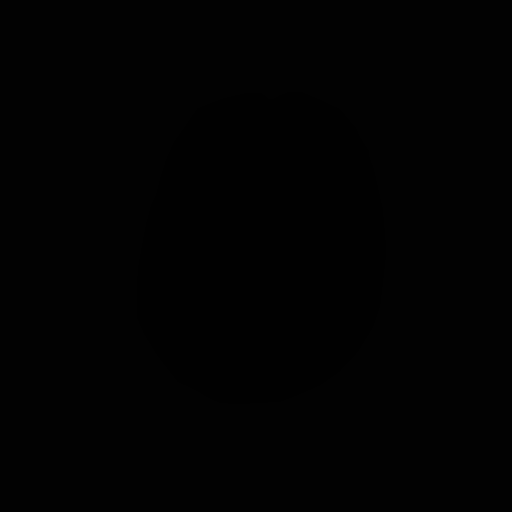

Supplement: S6 Data — (ZIP) [file pone.0295536.s007.zip › S7_Data/Tset set 2/Label/Label_105.png]

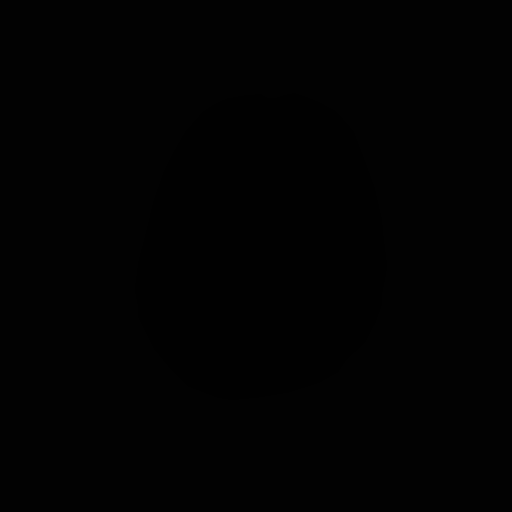

Supplement: S6 Data — (ZIP) [file pone.0295536.s007.zip › S7_Data/Tset set 2/Label/Label_106.png]

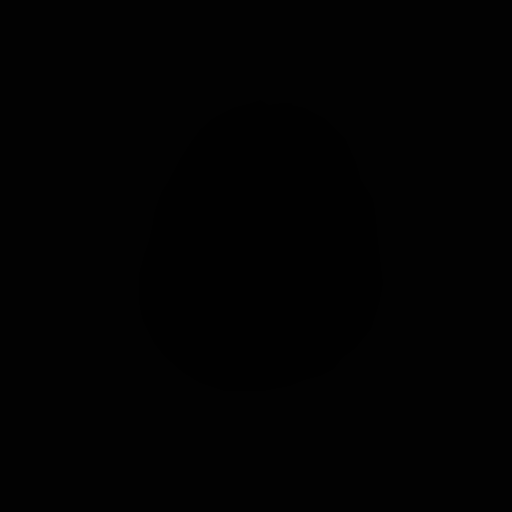

Supplement: S6 Data — (ZIP) [file pone.0295536.s007.zip › S7_Data/Tset set 2/Label/Label_107.png]

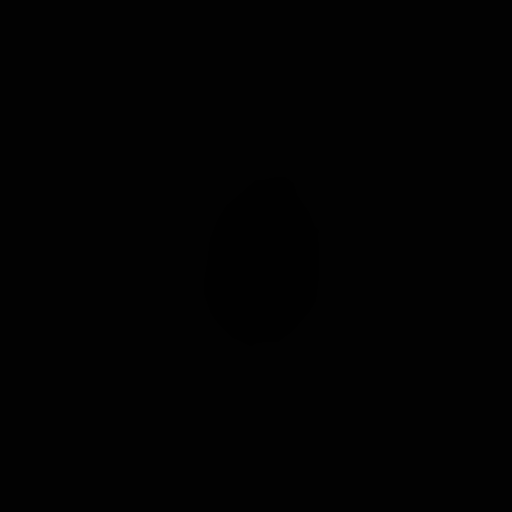

Supplement: S6 Data — (ZIP) [file pone.0295536.s007.zip › S7_Data/Tset set 2/Label/Label_108.png]

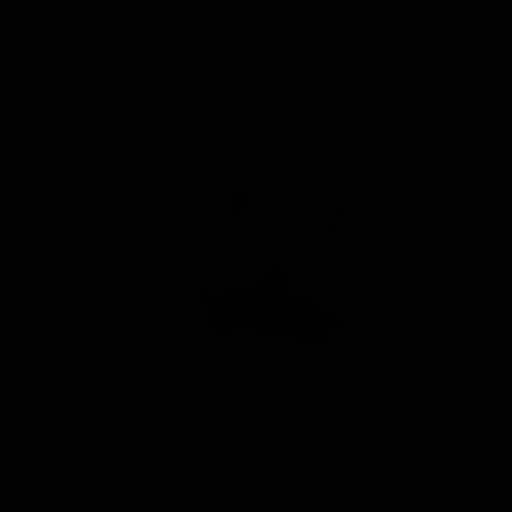

Supplement: S6 Data — (ZIP) [file pone.0295536.s007.zip › S7_Data/Tset set 2/Label/Label_109.png]

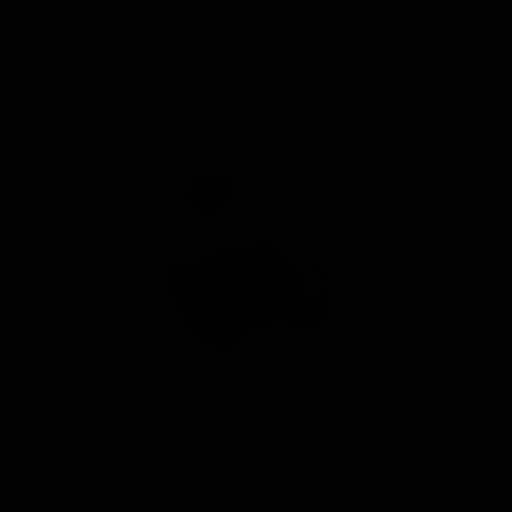

Supplement: S6 Data — (ZIP) [file pone.0295536.s007.zip › S7_Data/Tset set 2/Label/Label_11.png]

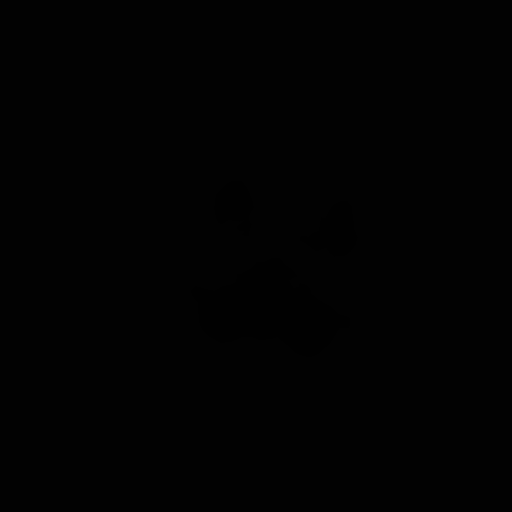

Supplement: S6 Data — (ZIP) [file pone.0295536.s007.zip › S7_Data/Tset set 2/Label/Label_110.png]

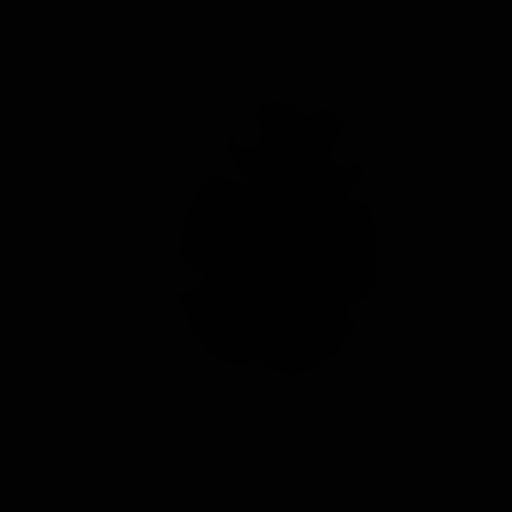

Supplement: S6 Data — (ZIP) [file pone.0295536.s007.zip › S7_Data/Tset set 2/Label/Label_111.png]

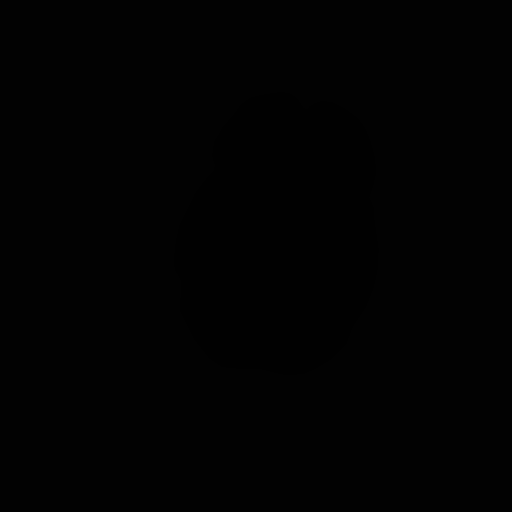

Supplement: S6 Data — (ZIP) [file pone.0295536.s007.zip › S7_Data/Tset set 2/Label/Label_112.png]

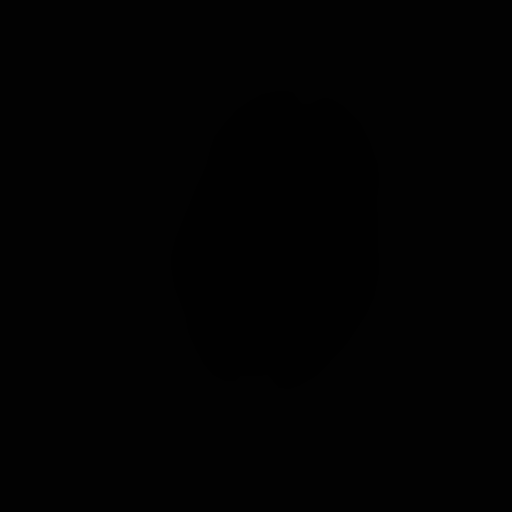

Supplement: S6 Data — (ZIP) [file pone.0295536.s007.zip › S7_Data/Tset set 2/Label/Label_113.png]

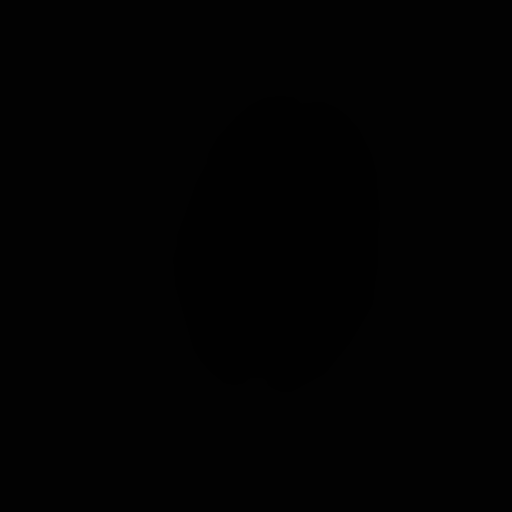

Supplement: S6 Data — (ZIP) [file pone.0295536.s007.zip › S7_Data/Tset set 2/Label/Label_114.png]

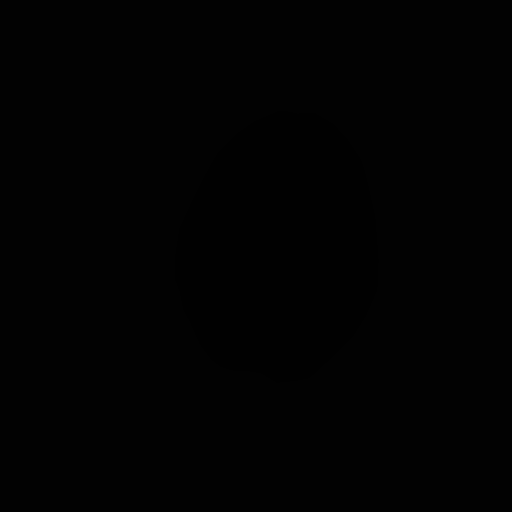

Supplement: S6 Data — (ZIP) [file pone.0295536.s007.zip › S7_Data/Tset set 2/Label/Label_115.png]

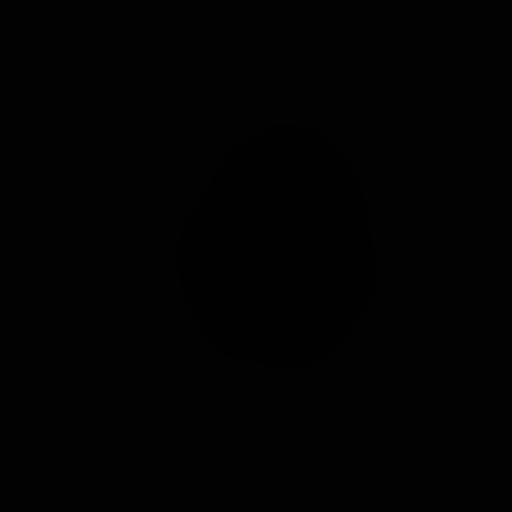

Supplement: S6 Data — (ZIP) [file pone.0295536.s007.zip › S7_Data/Tset set 2/Label/Label_116.png]

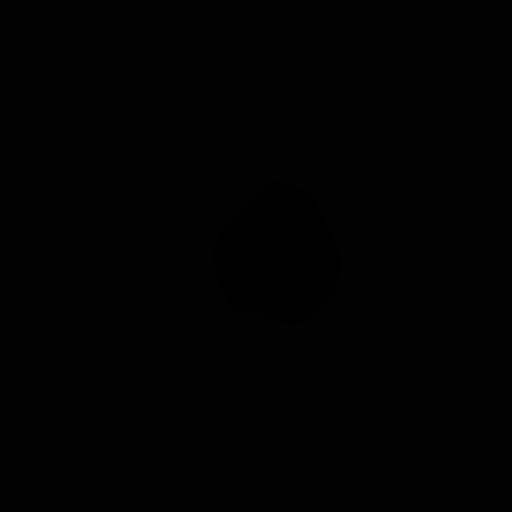

Supplement: S6 Data — (ZIP) [file pone.0295536.s007.zip › S7_Data/Tset set 2/Label/Label_117.png]

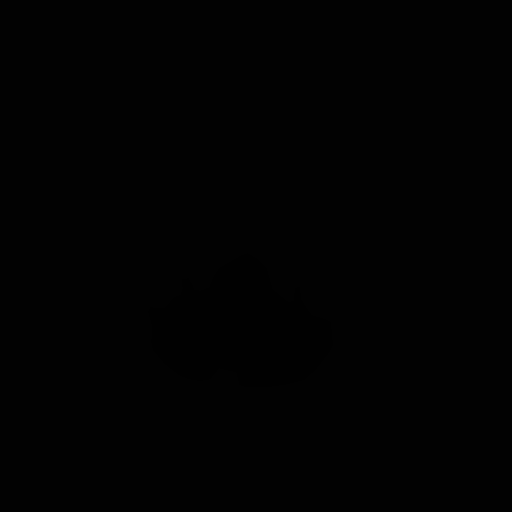

Supplement: S6 Data — (ZIP) [file pone.0295536.s007.zip › S7_Data/Tset set 2/Label/Label_118.png]

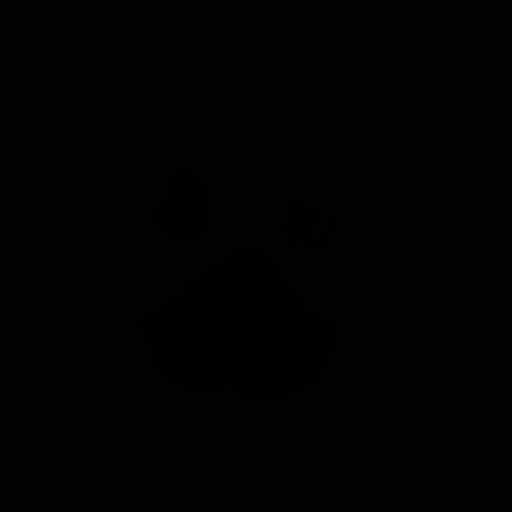

Supplement: S6 Data — (ZIP) [file pone.0295536.s007.zip › S7_Data/Tset set 2/Label/Label_119.png]

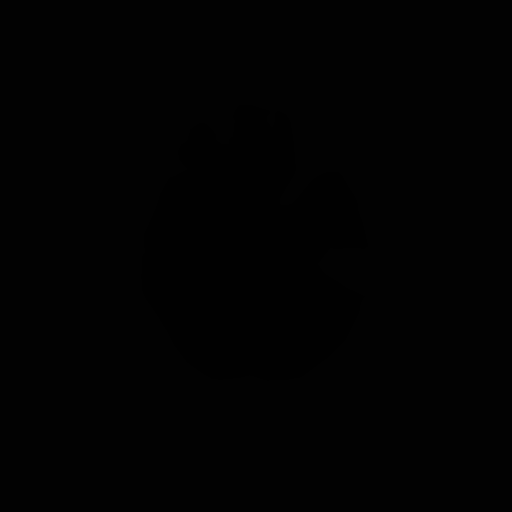

Supplement: S6 Data — (ZIP) [file pone.0295536.s007.zip › S7_Data/Tset set 2/Label/Label_12.png]

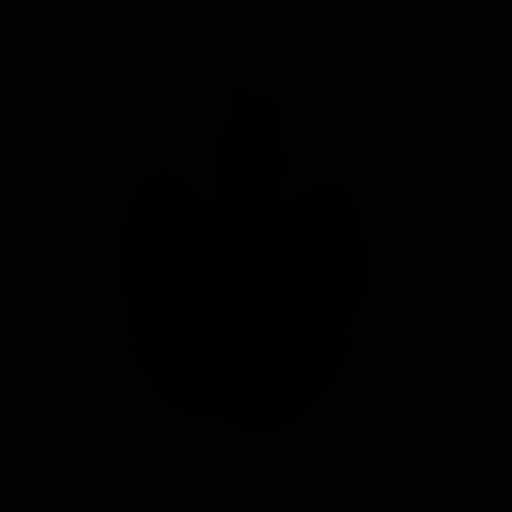

Supplement: S6 Data — (ZIP) [file pone.0295536.s007.zip › S7_Data/Tset set 2/Label/Label_120.png]

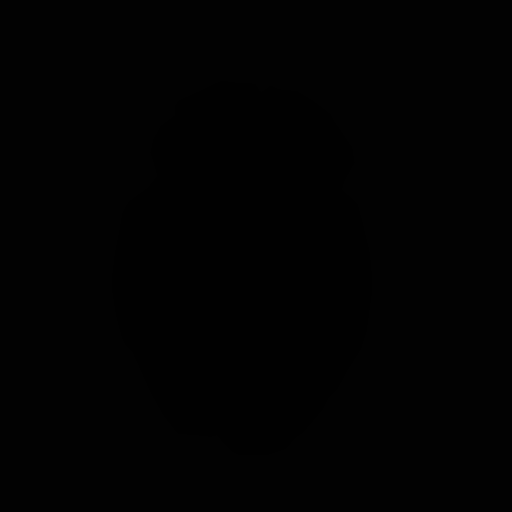

Supplement: S6 Data — (ZIP) [file pone.0295536.s007.zip › S7_Data/Tset set 2/Label/Label_121.png]

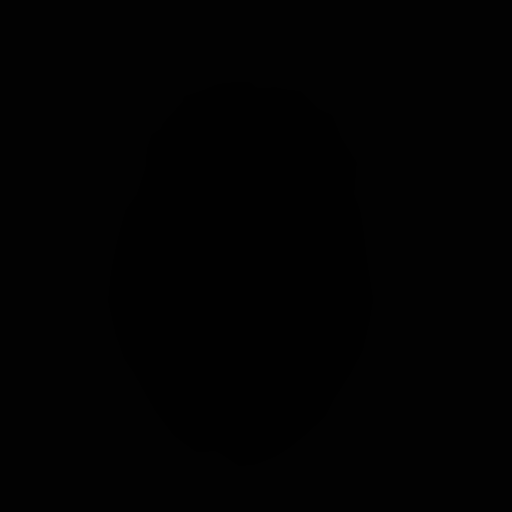

Supplement: S6 Data — (ZIP) [file pone.0295536.s007.zip › S7_Data/Tset set 2/Label/Label_122.png]

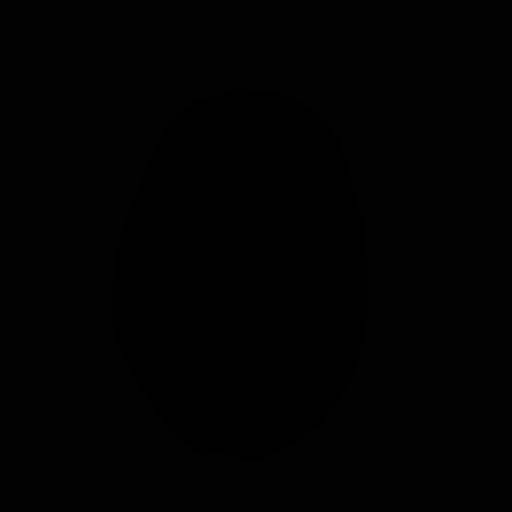

Supplement: S6 Data — (ZIP) [file pone.0295536.s007.zip › S7_Data/Tset set 2/Label/Label_123.png]

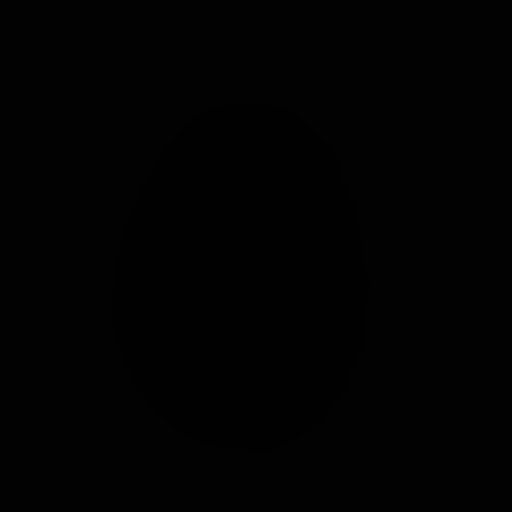

Supplement: S6 Data — (ZIP) [file pone.0295536.s007.zip › S7_Data/Tset set 2/Label/Label_124.png]

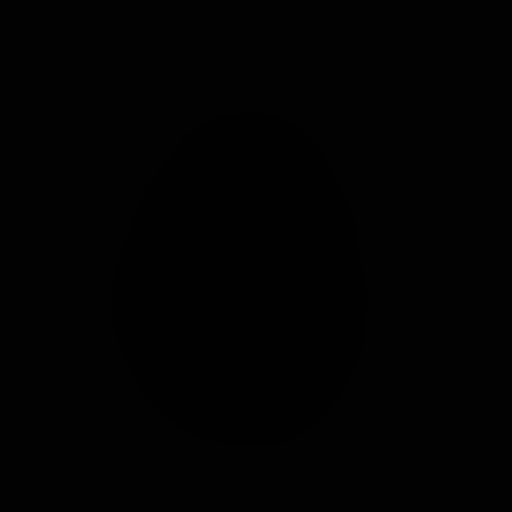

Supplement: S6 Data — (ZIP) [file pone.0295536.s007.zip › S7_Data/Tset set 2/Label/Label_125.png]

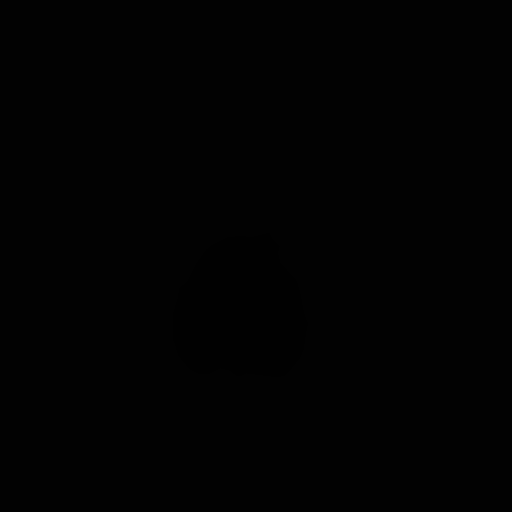

Supplement: S6 Data — (ZIP) [file pone.0295536.s007.zip › S7_Data/Tset set 2/Label/Label_126.png]

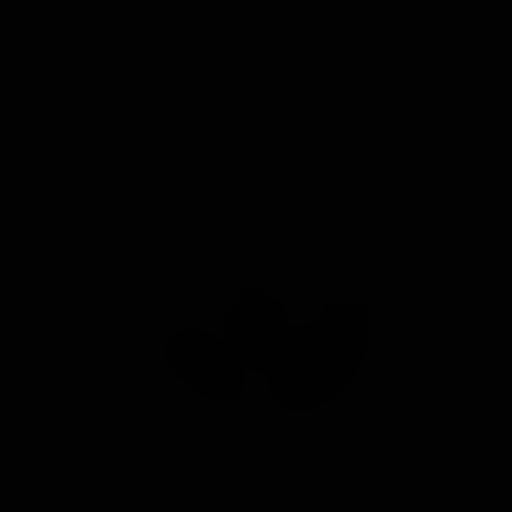

Supplement: S6 Data — (ZIP) [file pone.0295536.s007.zip › S7_Data/Tset set 2/Label/Label_127.png]

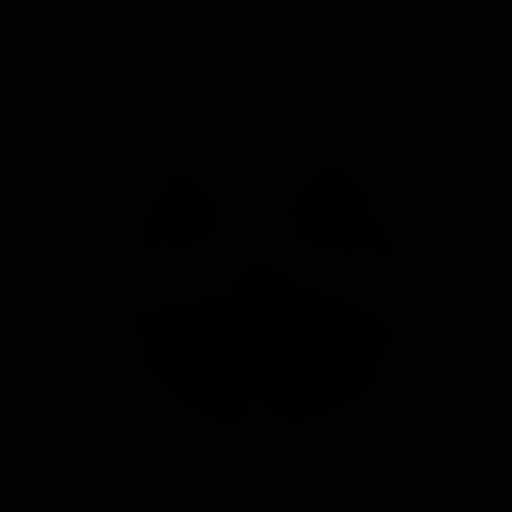

Supplement: S6 Data — (ZIP) [file pone.0295536.s007.zip › S7_Data/Tset set 2/Label/Label_128.png]

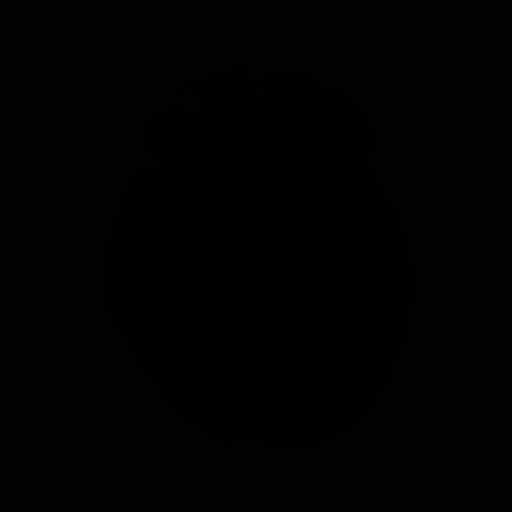

Supplement: S6 Data — (ZIP) [file pone.0295536.s007.zip › S7_Data/Tset set 2/Label/Label_129.png]

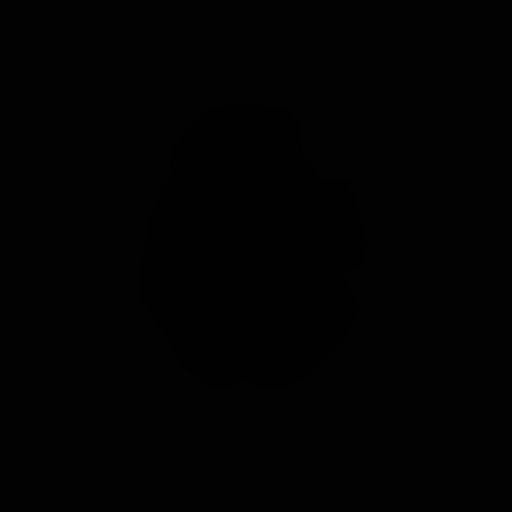

Supplement: S6 Data — (ZIP) [file pone.0295536.s007.zip › S7_Data/Tset set 2/Label/Label_13.png]

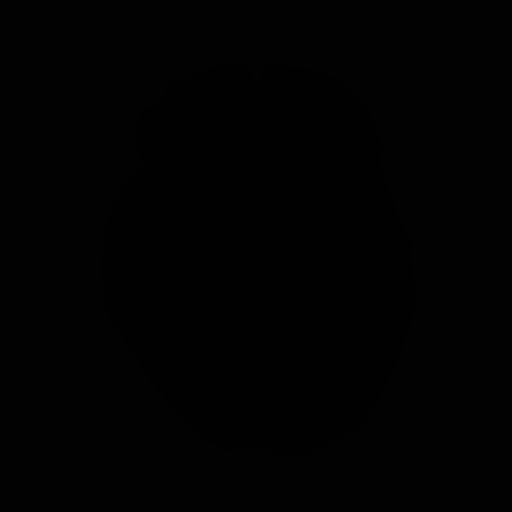

Supplement: S6 Data — (ZIP) [file pone.0295536.s007.zip › S7_Data/Tset set 2/Label/Label_130.png]

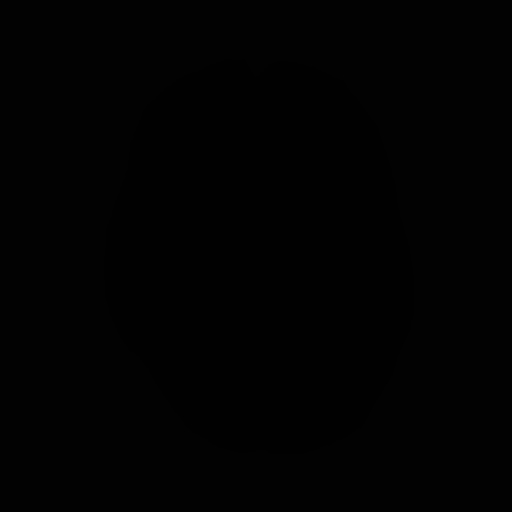

Supplement: S6 Data — (ZIP) [file pone.0295536.s007.zip › S7_Data/Tset set 2/Label/Label_131.png]

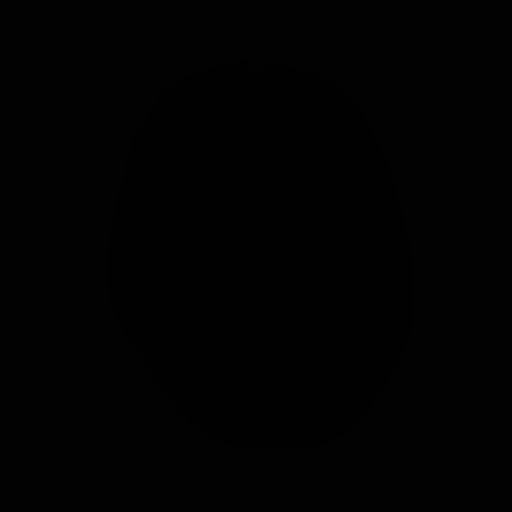

Supplement: S6 Data — (ZIP) [file pone.0295536.s007.zip › S7_Data/Tset set 2/Label/Label_132.png]

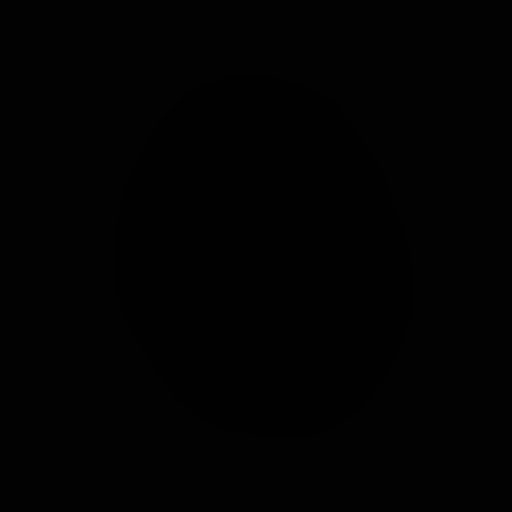

Supplement: S6 Data — (ZIP) [file pone.0295536.s007.zip › S7_Data/Tset set 2/Label/Label_133.png]

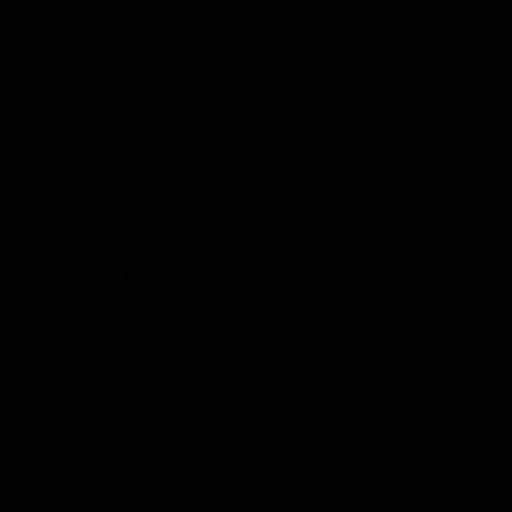

Supplement: S6 Data — (ZIP) [file pone.0295536.s007.zip › S7_Data/Tset set 2/Label/Label_134.png]

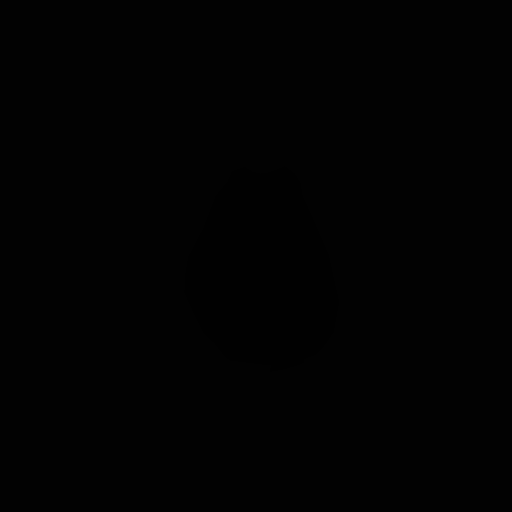

Supplement: S6 Data — (ZIP) [file pone.0295536.s007.zip › S7_Data/Tset set 2/Label/Label_135.png]

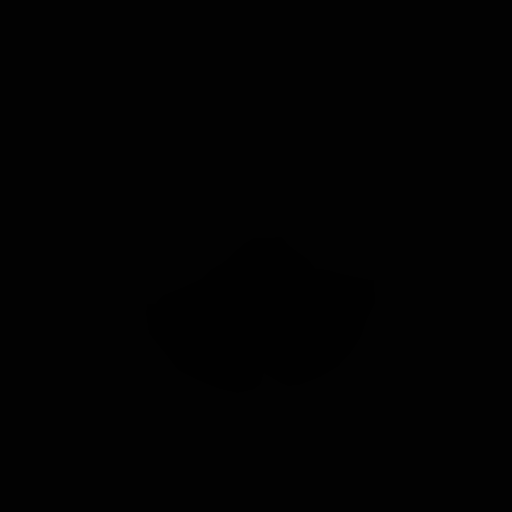

Supplement: S6 Data — (ZIP) [file pone.0295536.s007.zip › S7_Data/Tset set 2/Label/Label_136.png]

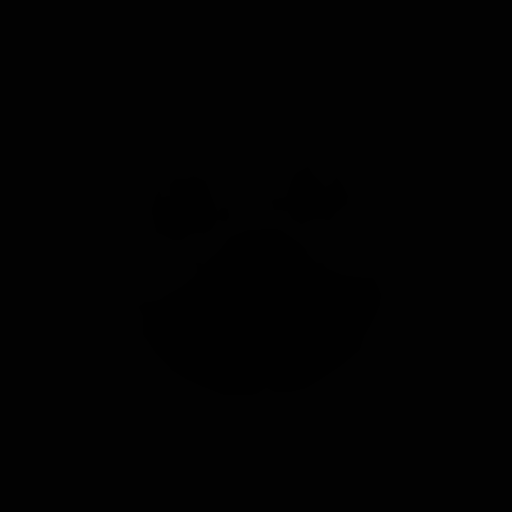

Supplement: S6 Data — (ZIP) [file pone.0295536.s007.zip › S7_Data/Tset set 2/Label/Label_137.png]

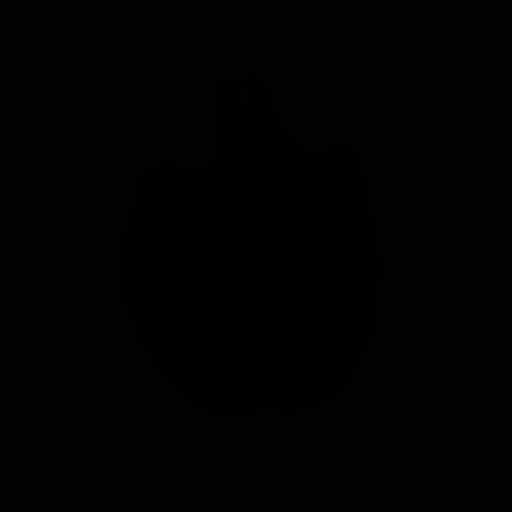

Supplement: S6 Data — (ZIP) [file pone.0295536.s007.zip › S7_Data/Tset set 2/Label/Label_138.png]

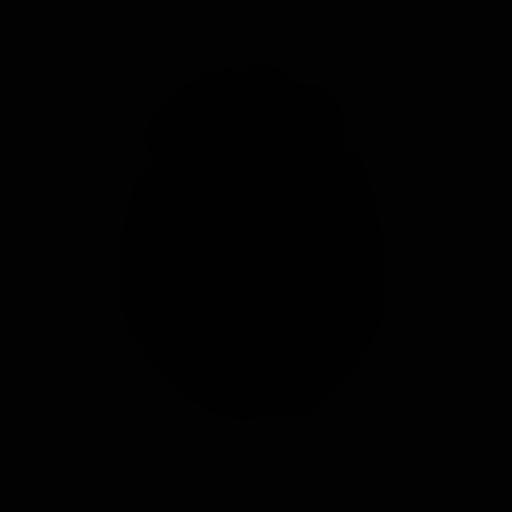

Supplement: S6 Data — (ZIP) [file pone.0295536.s007.zip › S7_Data/Tset set 2/Label/Label_139.png]

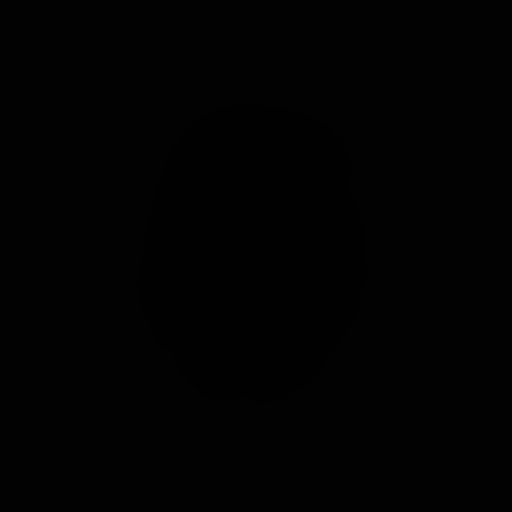

Supplement: S6 Data — (ZIP) [file pone.0295536.s007.zip › S7_Data/Tset set 2/Label/Label_14.png]

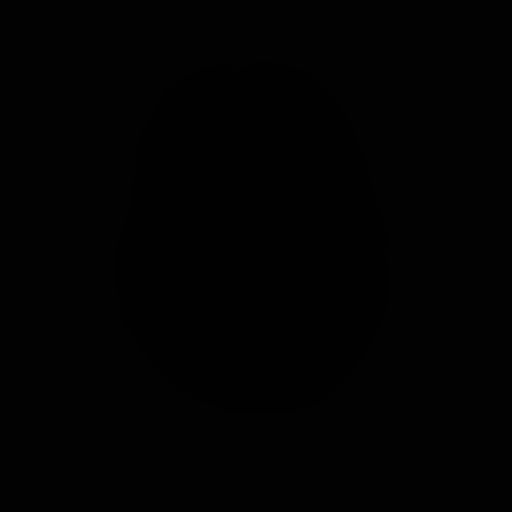

Supplement: S6 Data — (ZIP) [file pone.0295536.s007.zip › S7_Data/Tset set 2/Label/Label_140.png]

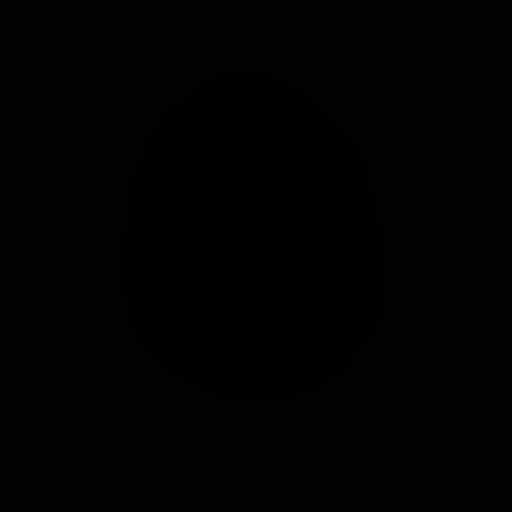

Supplement: S6 Data — (ZIP) [file pone.0295536.s007.zip › S7_Data/Tset set 2/Label/Label_141.png]

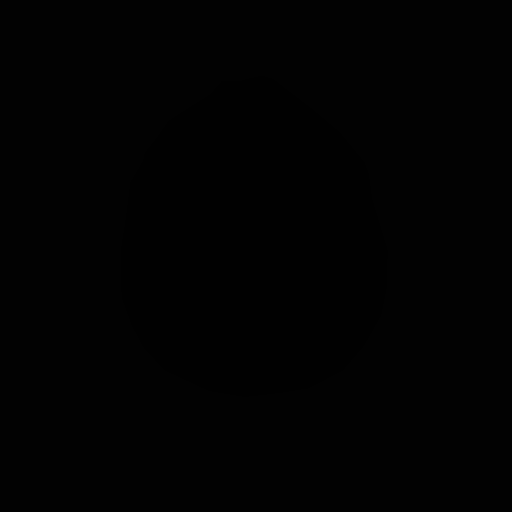

Supplement: S6 Data — (ZIP) [file pone.0295536.s007.zip › S7_Data/Tset set 2/Label/Label_142.png]

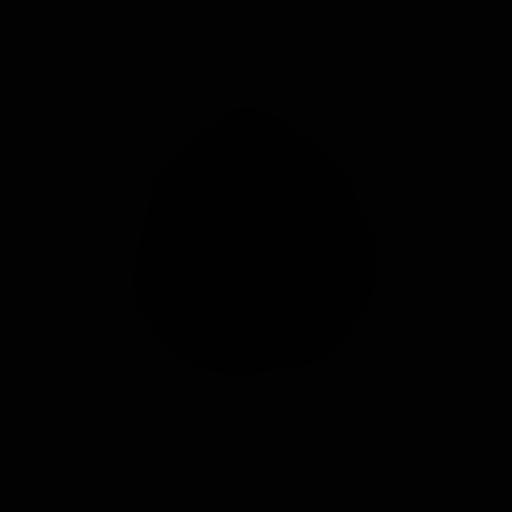

Supplement: S6 Data — (ZIP) [file pone.0295536.s007.zip › S7_Data/Tset set 2/Label/Label_143.png]

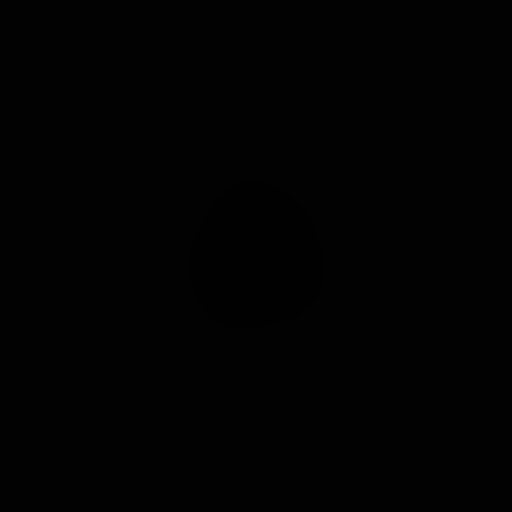

Supplement: S6 Data — (ZIP) [file pone.0295536.s007.zip › S7_Data/Tset set 2/Label/Label_144.png]

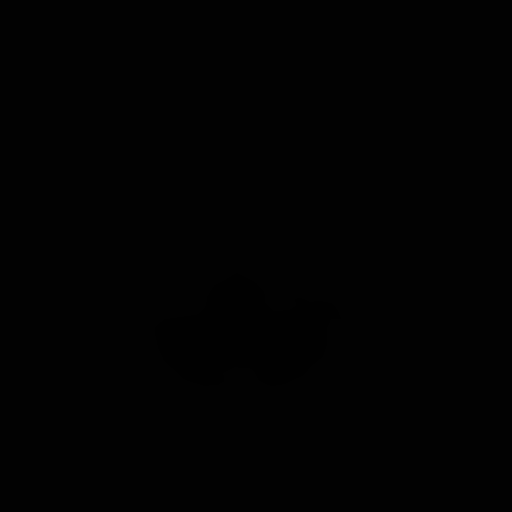

Supplement: S6 Data — (ZIP) [file pone.0295536.s007.zip › S7_Data/Tset set 2/Label/Label_145.png]

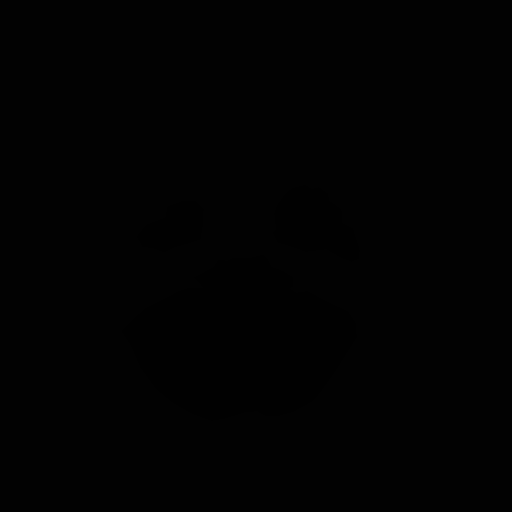

Supplement: S6 Data — (ZIP) [file pone.0295536.s007.zip › S7_Data/Tset set 2/Label/Label_146.png]

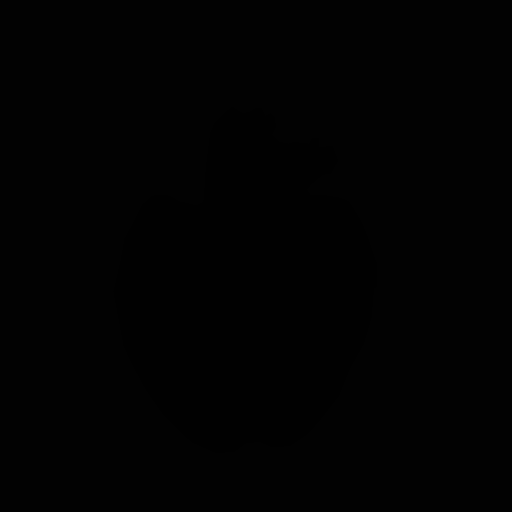

Supplement: S6 Data — (ZIP) [file pone.0295536.s007.zip › S7_Data/Tset set 2/Label/Label_147.png]

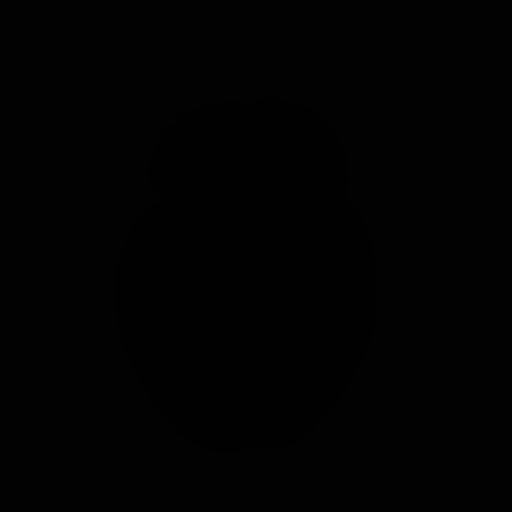

Supplement: S6 Data — (ZIP) [file pone.0295536.s007.zip › S7_Data/Tset set 2/Label/Label_148.png]

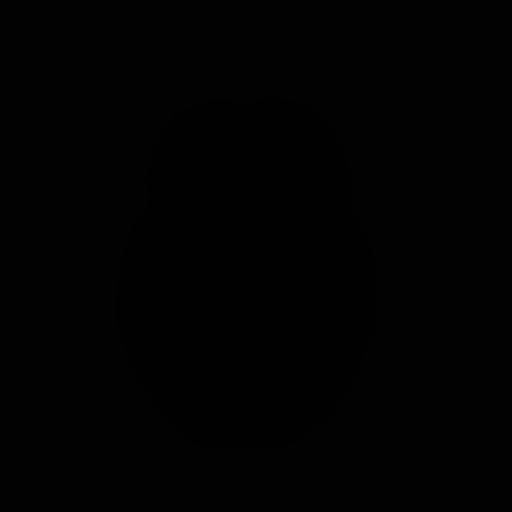

Supplement: S6 Data — (ZIP) [file pone.0295536.s007.zip › S7_Data/Tset set 2/Label/Label_149.png]

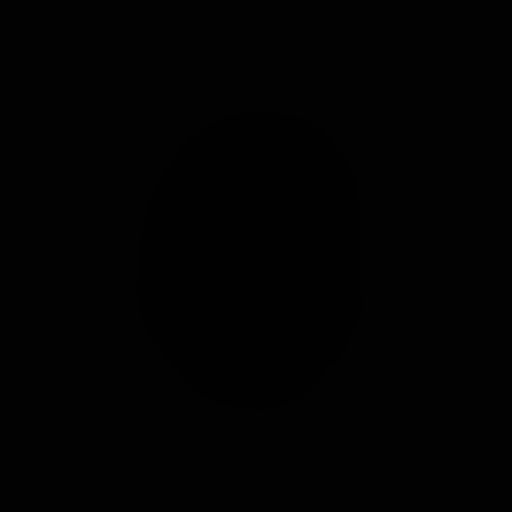

Supplement: S6 Data — (ZIP) [file pone.0295536.s007.zip › S7_Data/Tset set 2/Label/Label_15.png]

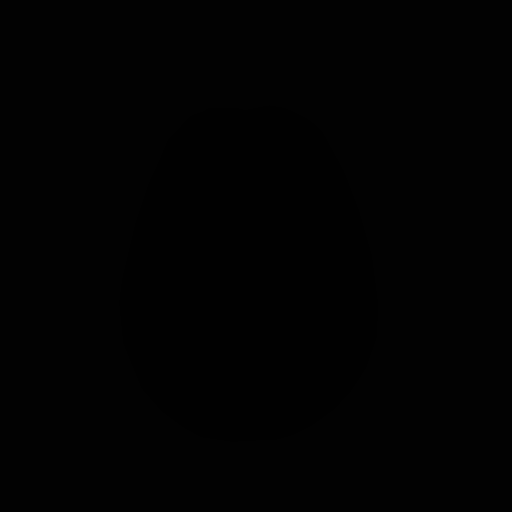

Supplement: S6 Data — (ZIP) [file pone.0295536.s007.zip › S7_Data/Tset set 2/Label/Label_150.png]

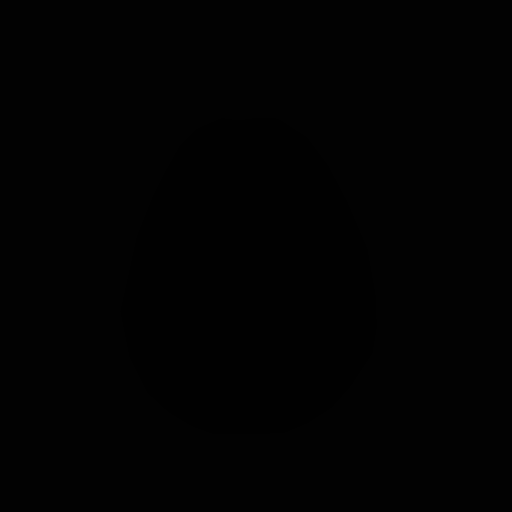

Supplement: S6 Data — (ZIP) [file pone.0295536.s007.zip › S7_Data/Tset set 2/Label/Label_151.png]

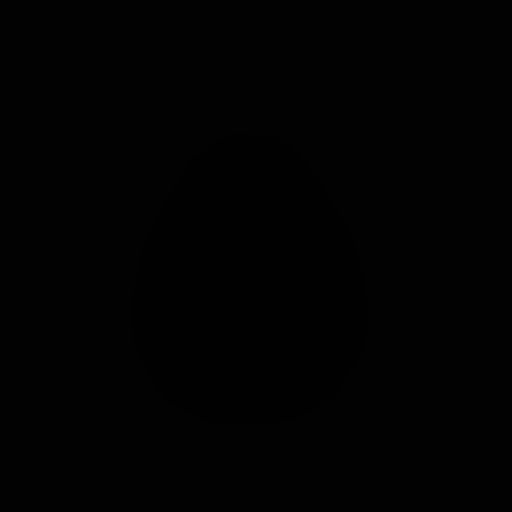

Supplement: S6 Data — (ZIP) [file pone.0295536.s007.zip › S7_Data/Tset set 2/Label/Label_152.png]

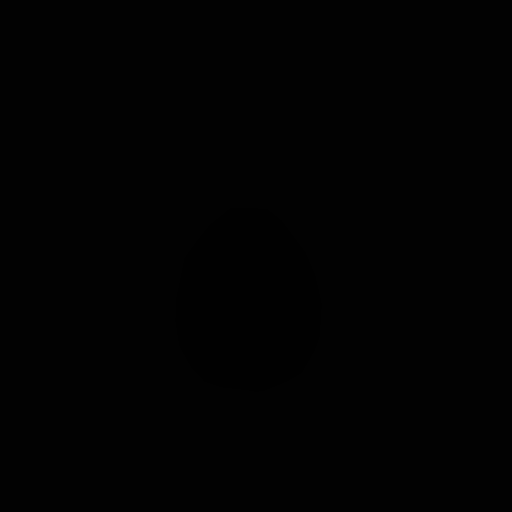

Supplement: S6 Data — (ZIP) [file pone.0295536.s007.zip › S7_Data/Tset set 2/Label/Label_153.png]

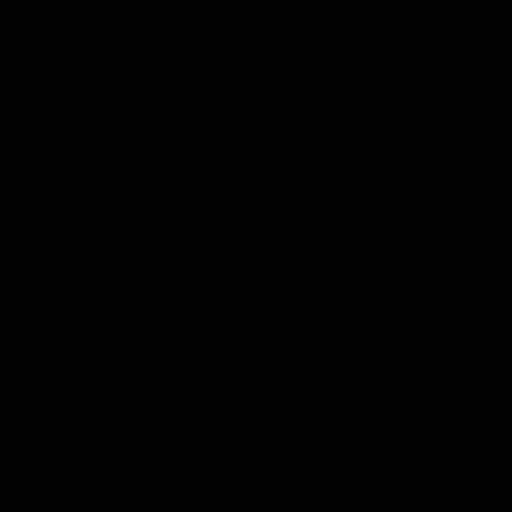

Supplement: S6 Data — (ZIP) [file pone.0295536.s007.zip › S7_Data/Tset set 2/Label/Label_154.png]

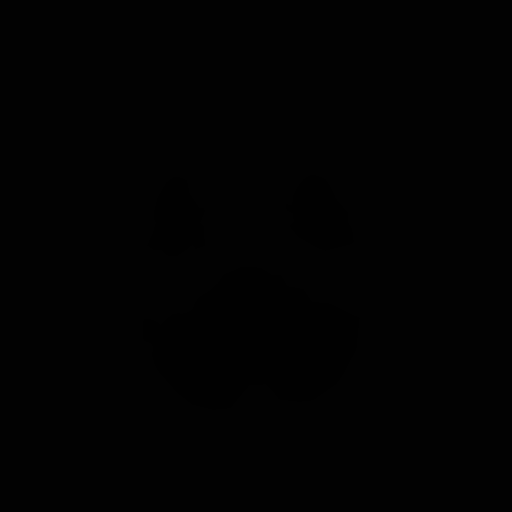

Supplement: S6 Data — (ZIP) [file pone.0295536.s007.zip › S7_Data/Tset set 2/Label/Label_155.png]

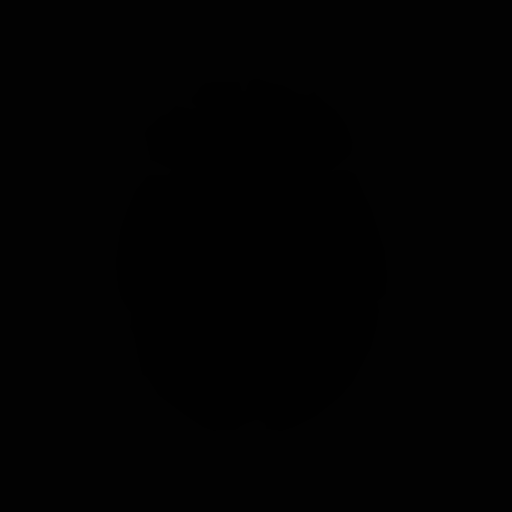

Supplement: S6 Data — (ZIP) [file pone.0295536.s007.zip › S7_Data/Tset set 2/Label/Label_156.png]

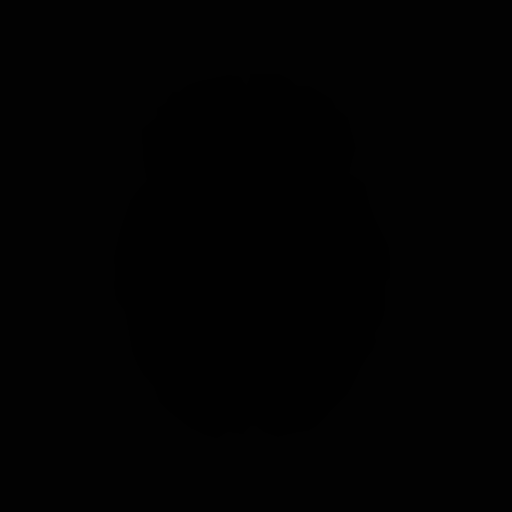

Supplement: S6 Data — (ZIP) [file pone.0295536.s007.zip › S7_Data/Tset set 2/Label/Label_157.png]

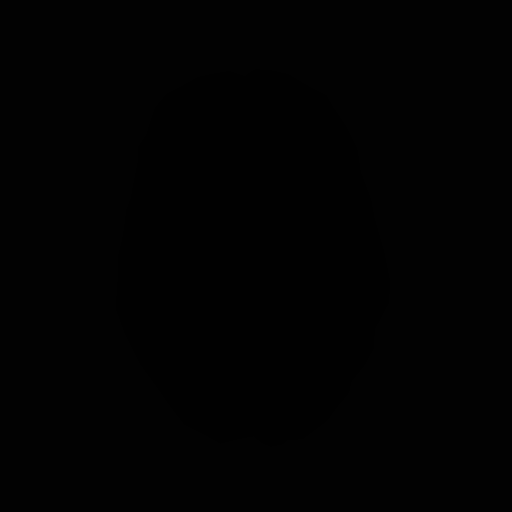

Supplement: S6 Data — (ZIP) [file pone.0295536.s007.zip › S7_Data/Tset set 2/Label/Label_158.png]

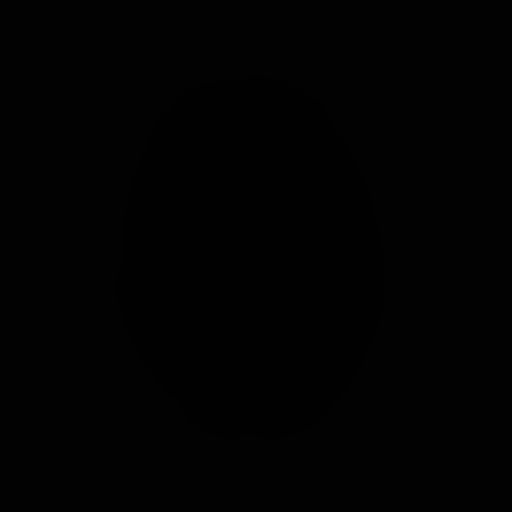

Supplement: S6 Data — (ZIP) [file pone.0295536.s007.zip › S7_Data/Tset set 2/Label/Label_159.png]

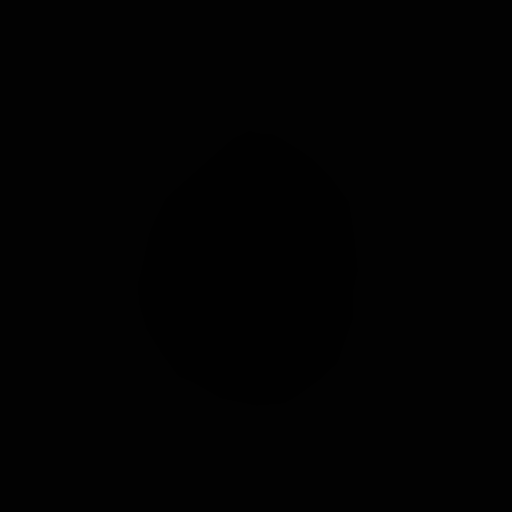

Supplement: S6 Data — (ZIP) [file pone.0295536.s007.zip › S7_Data/Tset set 2/Label/Label_16.png]

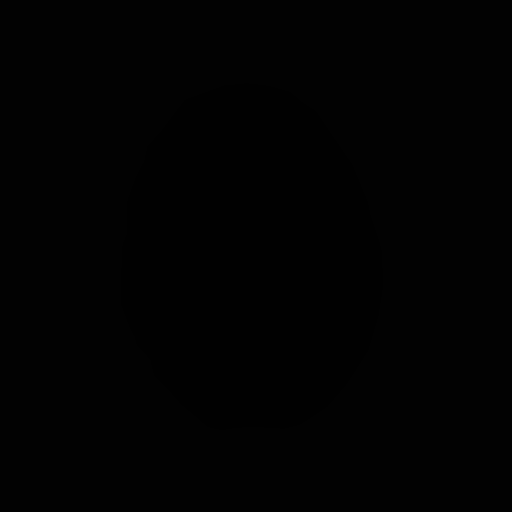

Supplement: S6 Data — (ZIP) [file pone.0295536.s007.zip › S7_Data/Tset set 2/Label/Label_160.png]

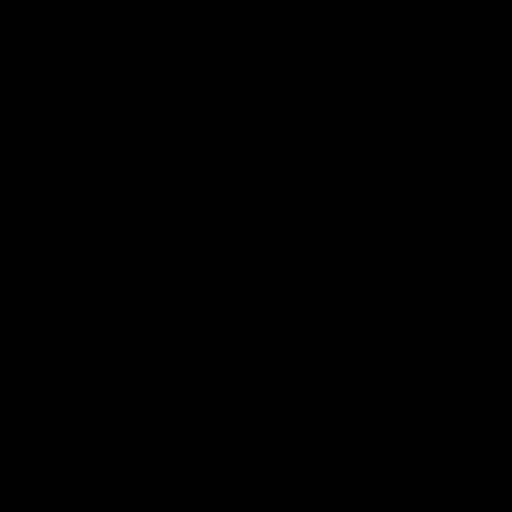

Supplement: S6 Data — (ZIP) [file pone.0295536.s007.zip › S7_Data/Tset set 2/Label/Label_161.png]

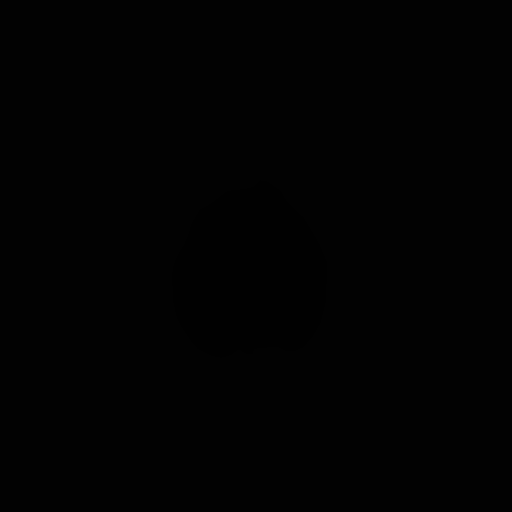

Supplement: S6 Data — (ZIP) [file pone.0295536.s007.zip › S7_Data/Tset set 2/Label/Label_162.png]

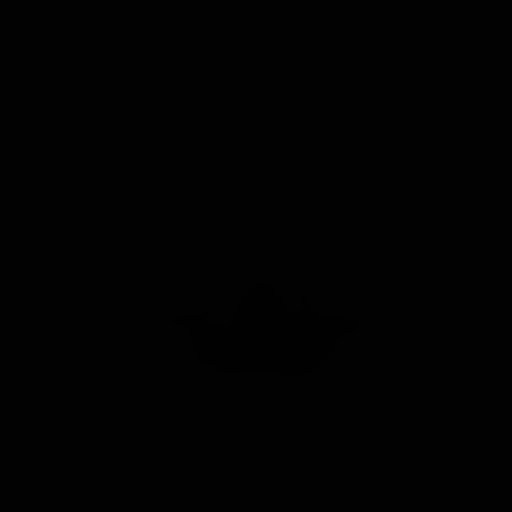

Supplement: S6 Data — (ZIP) [file pone.0295536.s007.zip › S7_Data/Tset set 2/Label/Label_163.png]

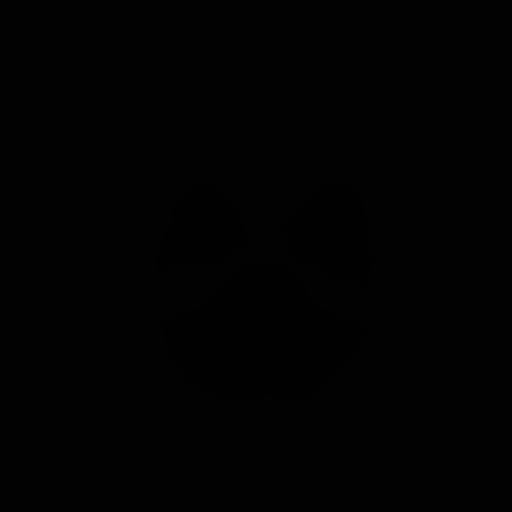

Supplement: S6 Data — (ZIP) [file pone.0295536.s007.zip › S7_Data/Tset set 2/Label/Label_164.png]
