# Supplementary figures and images for: Adaptive mask-based brain extraction method for head CT images (part 9 of 14)
Source: PLoS One. 2024 Mar 11;19(3):e0295536. doi: 10.1371/journal.pone.0295536 (PMC10927156; doi:10.1371/journal.pone.0295536)

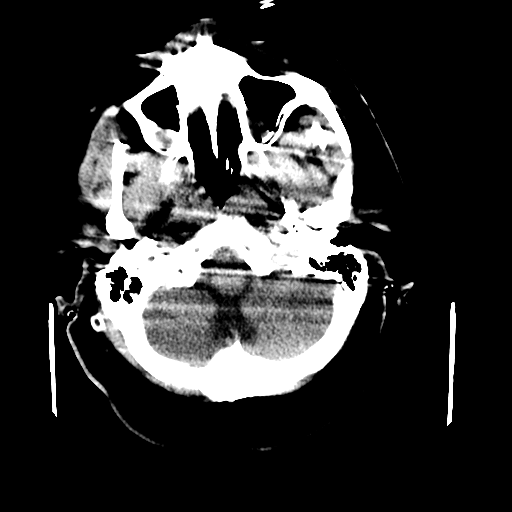

Supplement: S5 Data — (ZIP) [file pone.0295536.s006.zip › S6_Data/Tset set 1/0/85IM_0004-ID_3cf8db104.png]

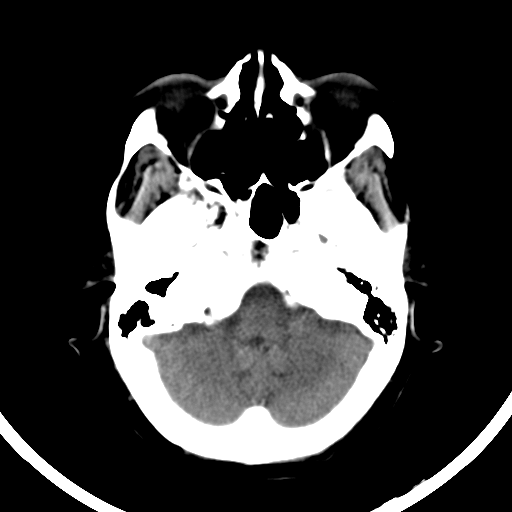

Supplement: S5 Data — (ZIP) [file pone.0295536.s006.zip › S6_Data/Tset set 1/0/IM_0006-ID_096eacffe.png]

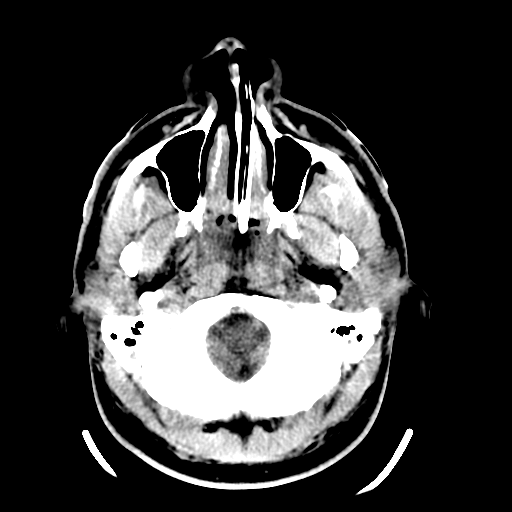

Supplement: S5 Data — (ZIP) [file pone.0295536.s006.zip › S6_Data/Tset set 1/0/IM_0010-ID_548f2ef73.png]

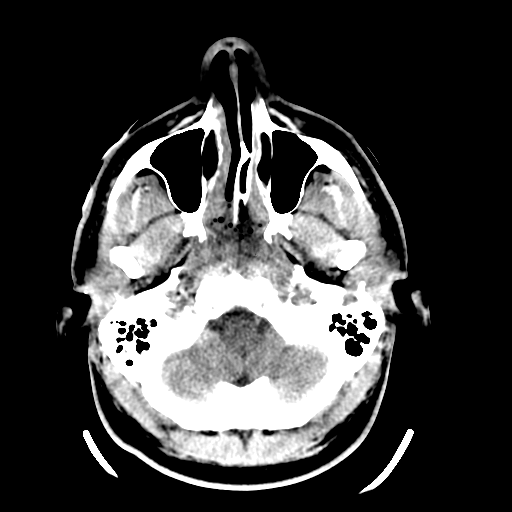

Supplement: S5 Data — (ZIP) [file pone.0295536.s006.zip › S6_Data/Tset set 1/0/IM_0011-ID_437143786.png]

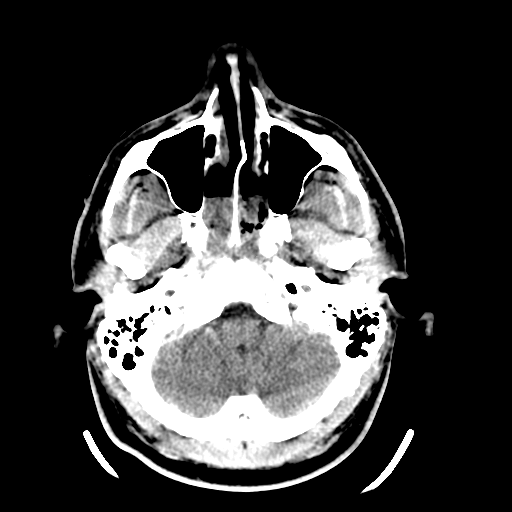

Supplement: S5 Data — (ZIP) [file pone.0295536.s006.zip › S6_Data/Tset set 1/0/IM_0012-ID_dcd51a6ac.png]

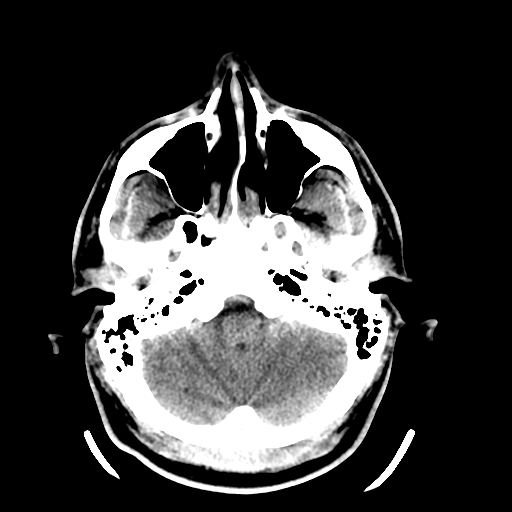

Supplement: S5 Data — (ZIP) [file pone.0295536.s006.zip › S6_Data/Tset set 1/0/IM_0013-ID_e264511b8.png]

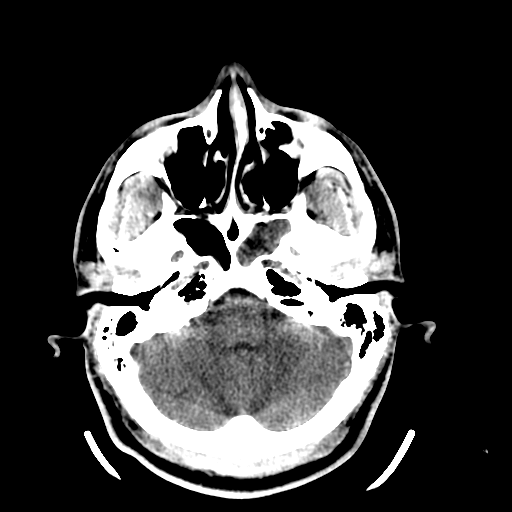

Supplement: S5 Data — (ZIP) [file pone.0295536.s006.zip › S6_Data/Tset set 1/0/IM_0014-ID_8f6067998.png]

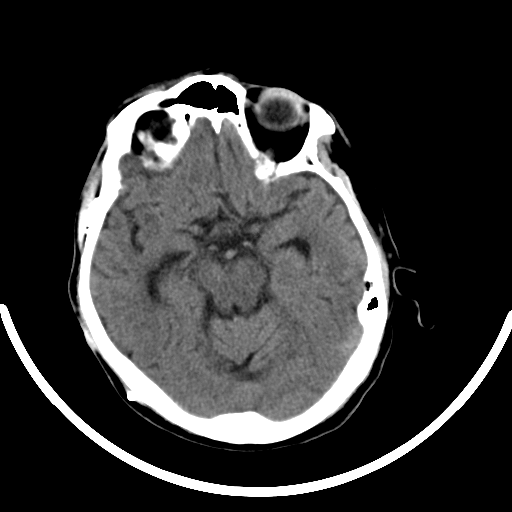

Supplement: S5 Data — (ZIP) [file pone.0295536.s006.zip › S6_Data/Tset set 1/1/16IM_0012-ID_eb78e80f0.png]

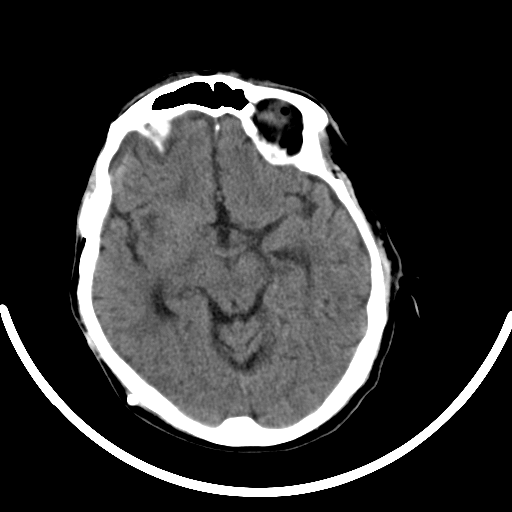

Supplement: S5 Data — (ZIP) [file pone.0295536.s006.zip › S6_Data/Tset set 1/1/16IM_0013-ID_20af19343.png]

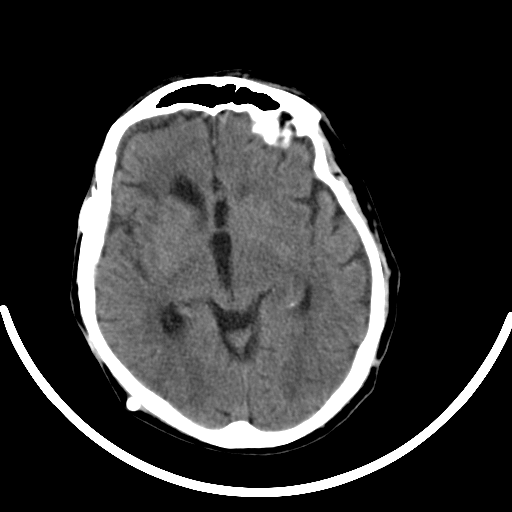

Supplement: S5 Data — (ZIP) [file pone.0295536.s006.zip › S6_Data/Tset set 1/1/16IM_0014-ID_84b58f8f8.png]

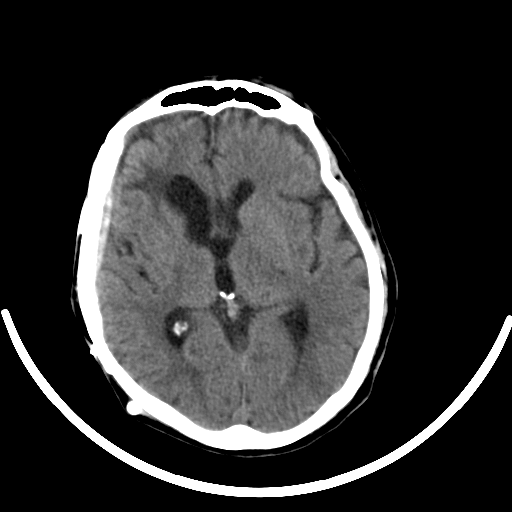

Supplement: S5 Data — (ZIP) [file pone.0295536.s006.zip › S6_Data/Tset set 1/1/16IM_0015-ID_6e50b19ec.png]

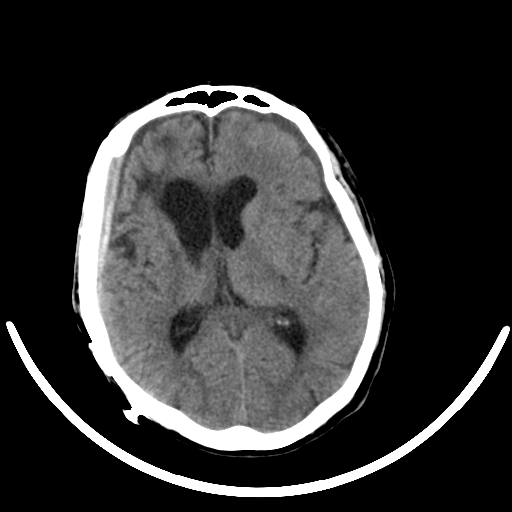

Supplement: S5 Data — (ZIP) [file pone.0295536.s006.zip › S6_Data/Tset set 1/1/16IM_0016-ID_f8d07dbd9.png]

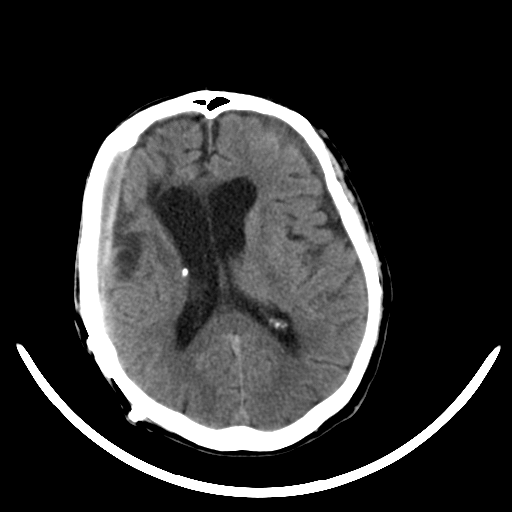

Supplement: S5 Data — (ZIP) [file pone.0295536.s006.zip › S6_Data/Tset set 1/1/16IM_0017-ID_14725ec04.png]

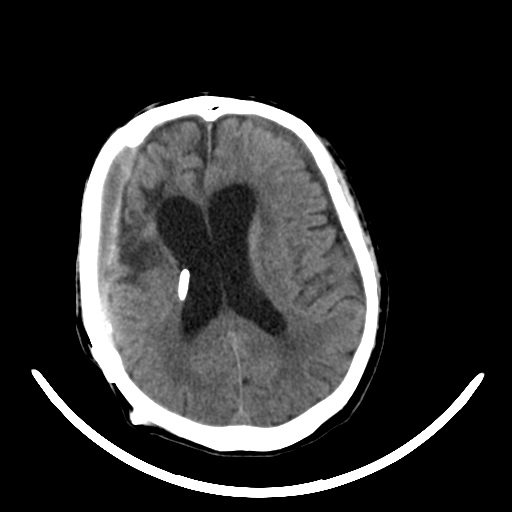

Supplement: S5 Data — (ZIP) [file pone.0295536.s006.zip › S6_Data/Tset set 1/1/16IM_0018-ID_a058e843b.png]

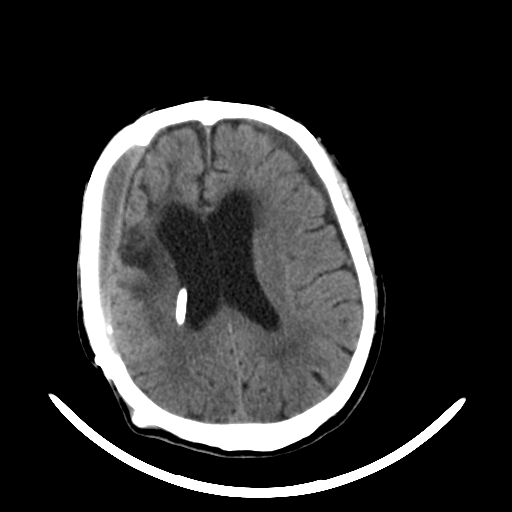

Supplement: S5 Data — (ZIP) [file pone.0295536.s006.zip › S6_Data/Tset set 1/1/16IM_0019-ID_1c5aebafe.png]

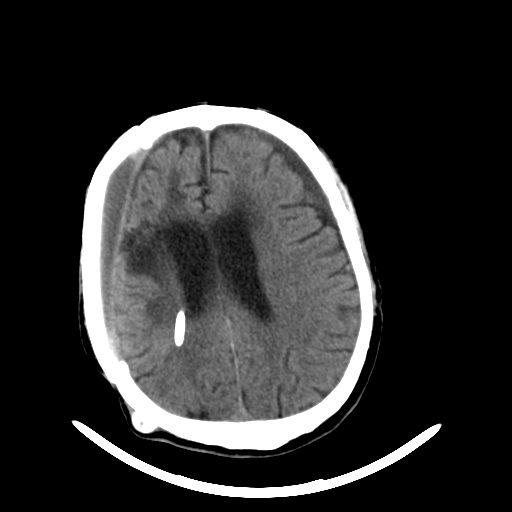

Supplement: S5 Data — (ZIP) [file pone.0295536.s006.zip › S6_Data/Tset set 1/1/16IM_0020-ID_357a827cc.png]

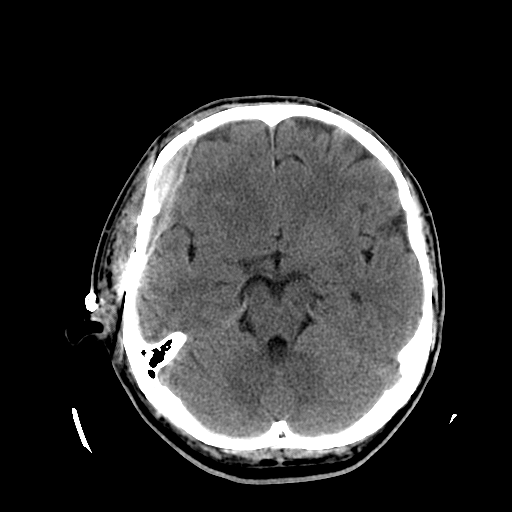

Supplement: S5 Data — (ZIP) [file pone.0295536.s006.zip › S6_Data/Tset set 1/1/19IM_0010-ID_5f97be66c.png]

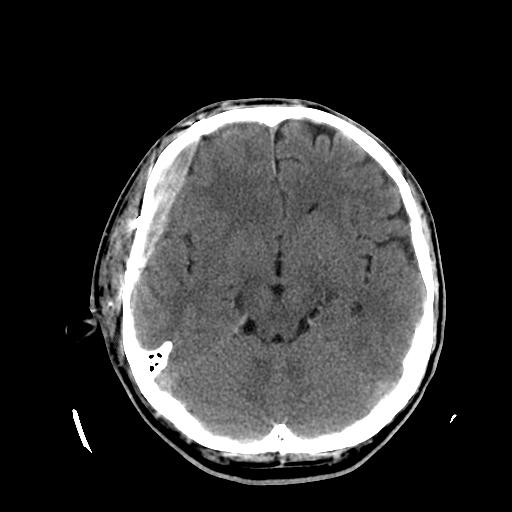

Supplement: S5 Data — (ZIP) [file pone.0295536.s006.zip › S6_Data/Tset set 1/1/19IM_0011-ID_87a32ea00.png]

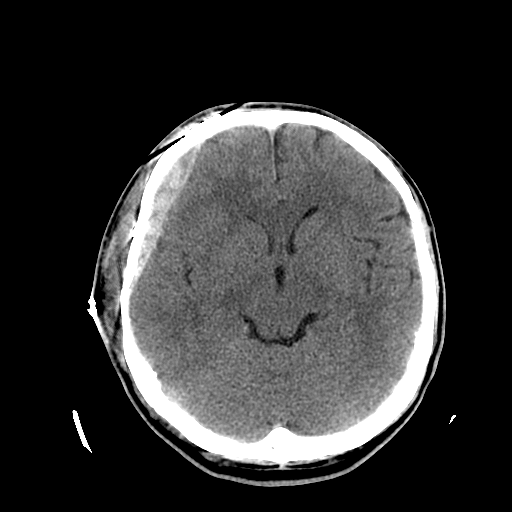

Supplement: S5 Data — (ZIP) [file pone.0295536.s006.zip › S6_Data/Tset set 1/1/19IM_0012-ID_cd4279ffd.png]

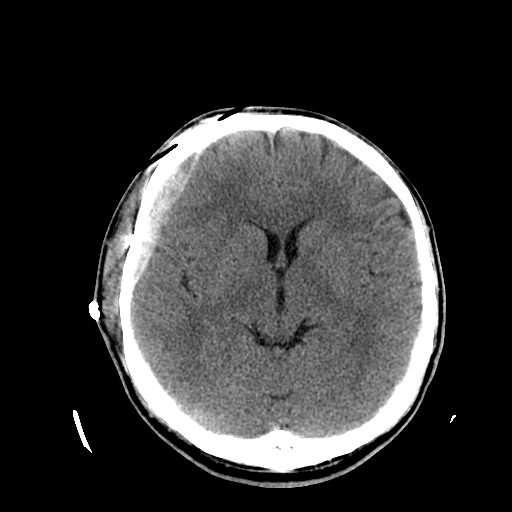

Supplement: S5 Data — (ZIP) [file pone.0295536.s006.zip › S6_Data/Tset set 1/1/19IM_0013-ID_1767dc99e.png]

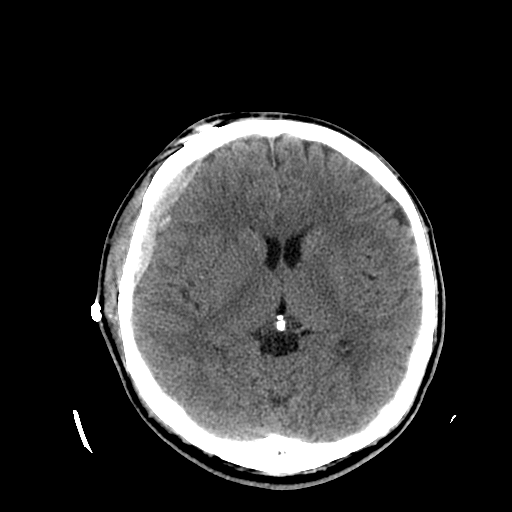

Supplement: S5 Data — (ZIP) [file pone.0295536.s006.zip › S6_Data/Tset set 1/1/19IM_0014-ID_42dd358d6.png]

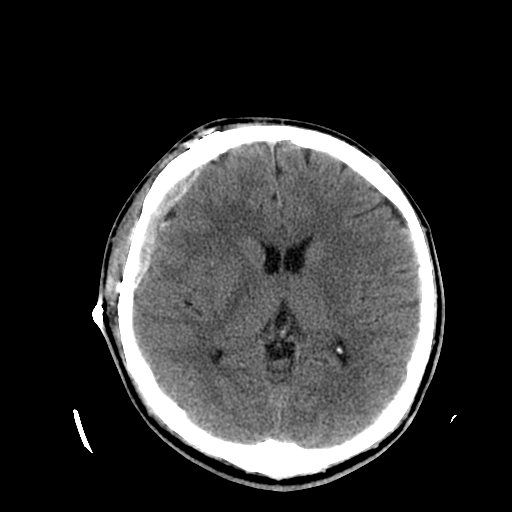

Supplement: S5 Data — (ZIP) [file pone.0295536.s006.zip › S6_Data/Tset set 1/1/19IM_0015-ID_fd9d1beae.png]

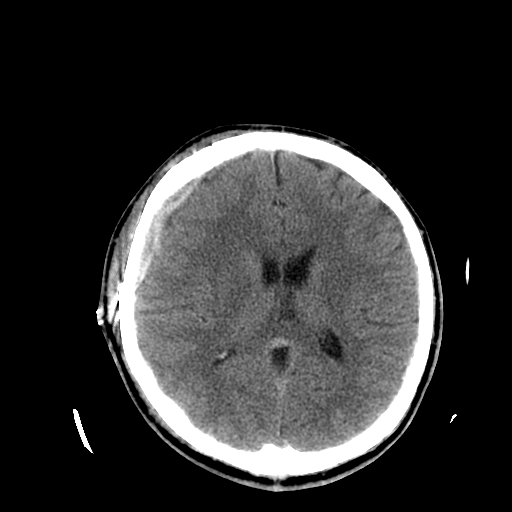

Supplement: S5 Data — (ZIP) [file pone.0295536.s006.zip › S6_Data/Tset set 1/1/19IM_0016-ID_dbc55ddcb.png]

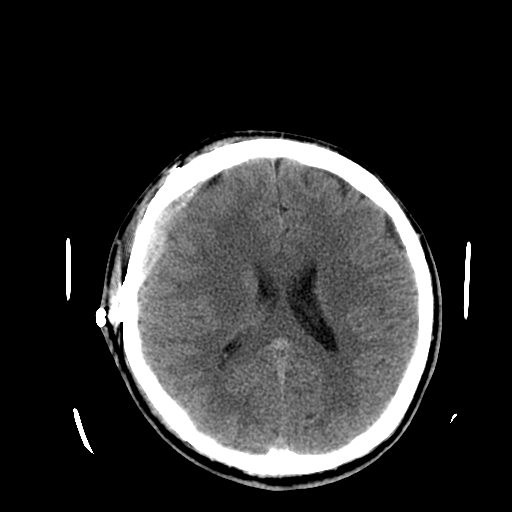

Supplement: S5 Data — (ZIP) [file pone.0295536.s006.zip › S6_Data/Tset set 1/1/19IM_0017-ID_e6bcaf40f.png]

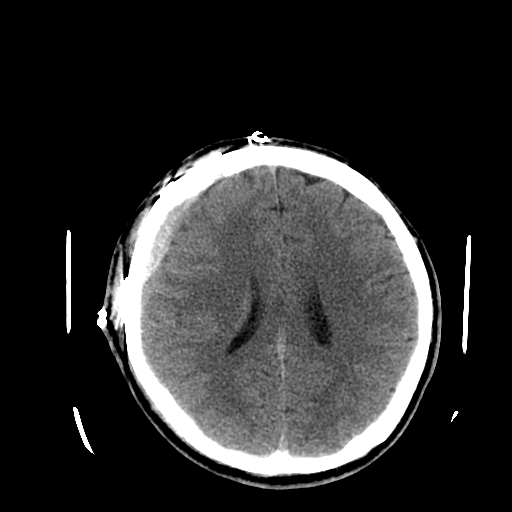

Supplement: S5 Data — (ZIP) [file pone.0295536.s006.zip › S6_Data/Tset set 1/1/19IM_0018-ID_9267048a2.png]

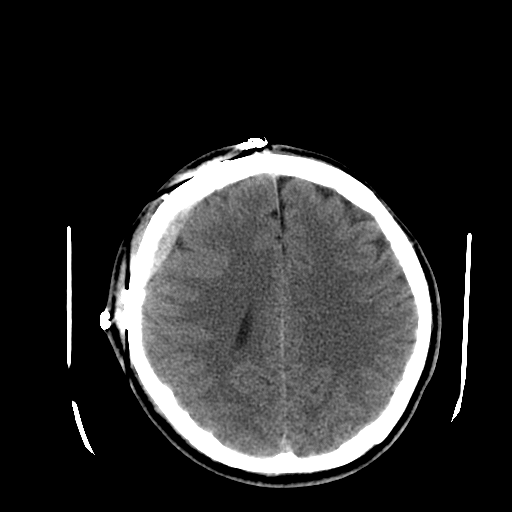

Supplement: S5 Data — (ZIP) [file pone.0295536.s006.zip › S6_Data/Tset set 1/1/19IM_0019-ID_940311b61.png]

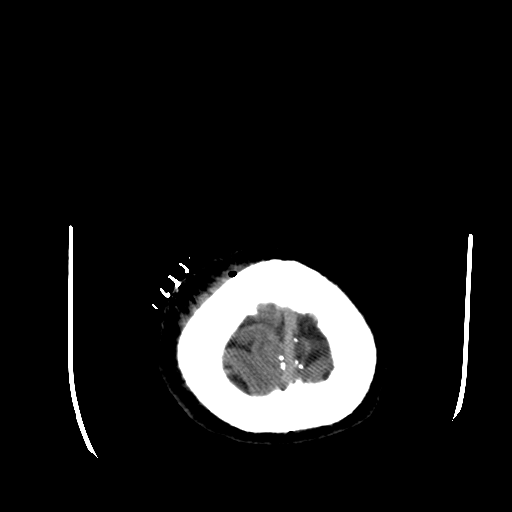

Supplement: S5 Data — (ZIP) [file pone.0295536.s006.zip › S6_Data/Tset set 1/1/19IM_0028-ID_d5afdb7f2.png]

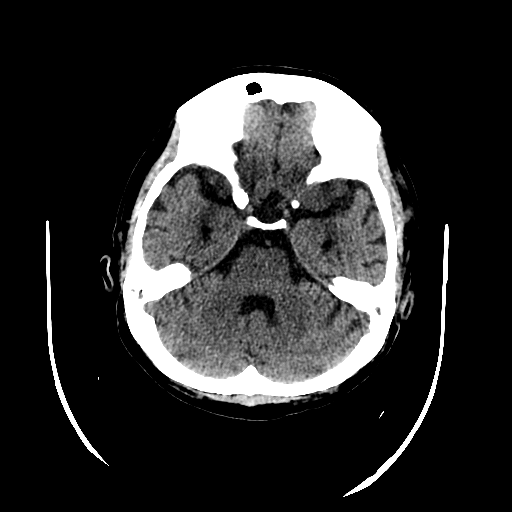

Supplement: S5 Data — (ZIP) [file pone.0295536.s006.zip › S6_Data/Tset set 1/1/20IM_0015-ID_26fbb65bc.png]

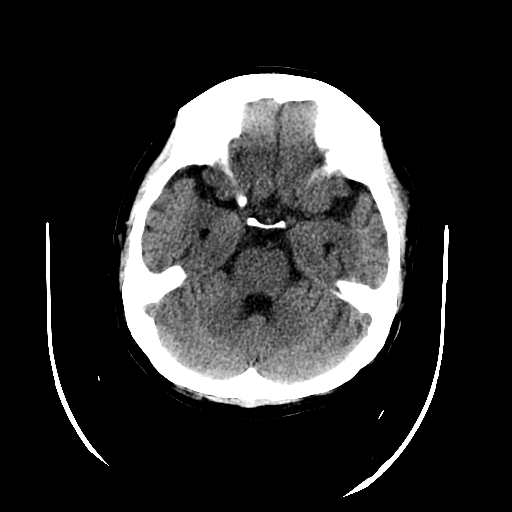

Supplement: S5 Data — (ZIP) [file pone.0295536.s006.zip › S6_Data/Tset set 1/1/20IM_0016-ID_a44e691e2.png]

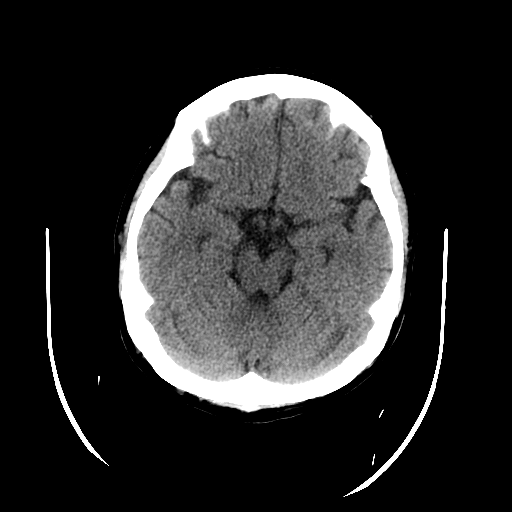

Supplement: S5 Data — (ZIP) [file pone.0295536.s006.zip › S6_Data/Tset set 1/1/20IM_0017-ID_576034982.png]

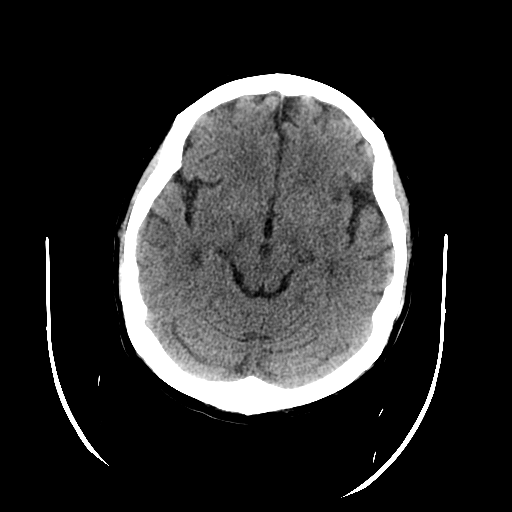

Supplement: S5 Data — (ZIP) [file pone.0295536.s006.zip › S6_Data/Tset set 1/1/20IM_0018-ID_0c93ea540.png]

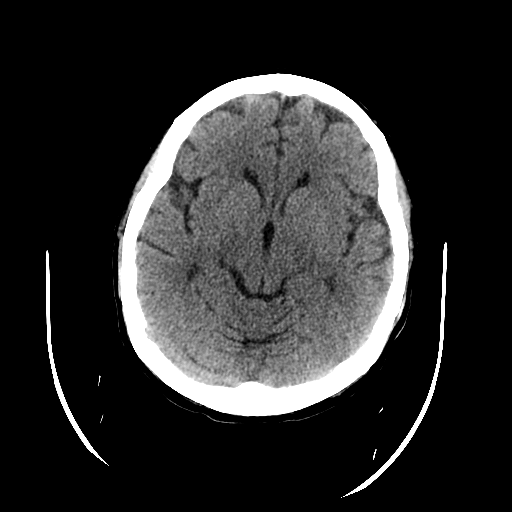

Supplement: S5 Data — (ZIP) [file pone.0295536.s006.zip › S6_Data/Tset set 1/1/20IM_0019-ID_4bd0b4664.png]

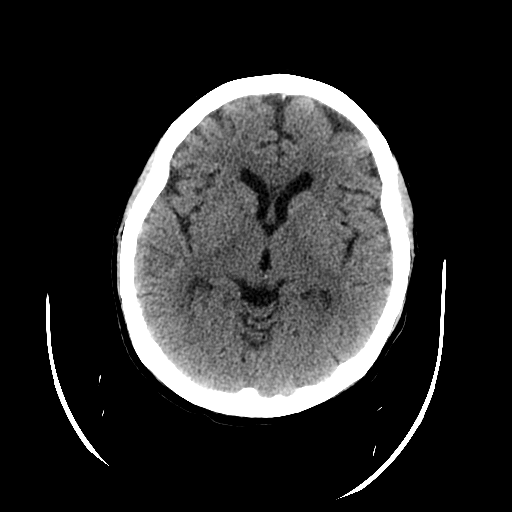

Supplement: S5 Data — (ZIP) [file pone.0295536.s006.zip › S6_Data/Tset set 1/1/20IM_0020-ID_ce2c62a75.png]

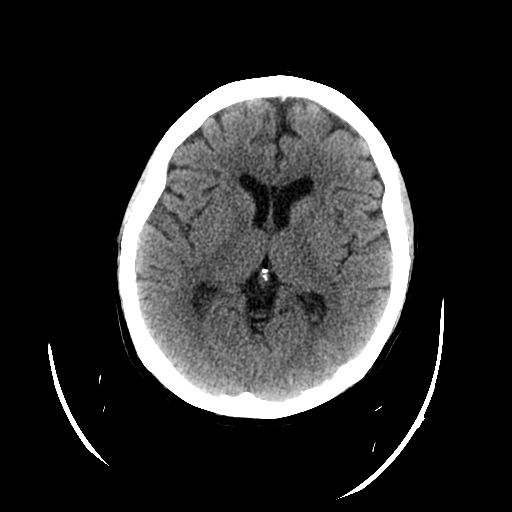

Supplement: S5 Data — (ZIP) [file pone.0295536.s006.zip › S6_Data/Tset set 1/1/20IM_0021-ID_e5267a689.png]

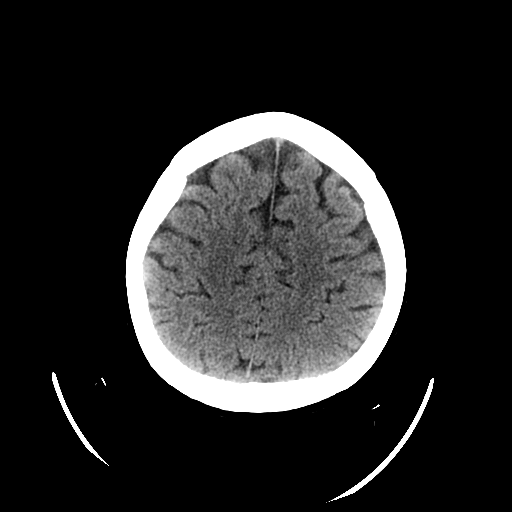

Supplement: S5 Data — (ZIP) [file pone.0295536.s006.zip › S6_Data/Tset set 1/1/20IM_0028-ID_a40897939.png]

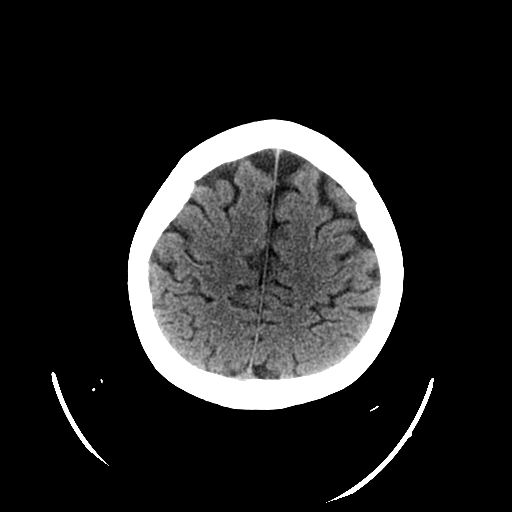

Supplement: S5 Data — (ZIP) [file pone.0295536.s006.zip › S6_Data/Tset set 1/1/20IM_0029-ID_55e7f4b05.png]

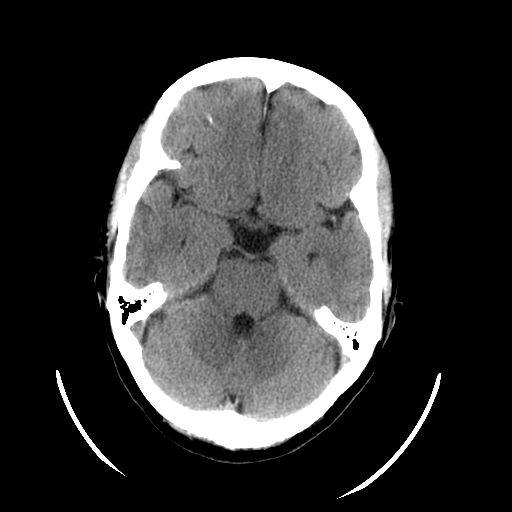

Supplement: S5 Data — (ZIP) [file pone.0295536.s006.zip › S6_Data/Tset set 1/1/21IM_0007-ID_5aabaf612.png]

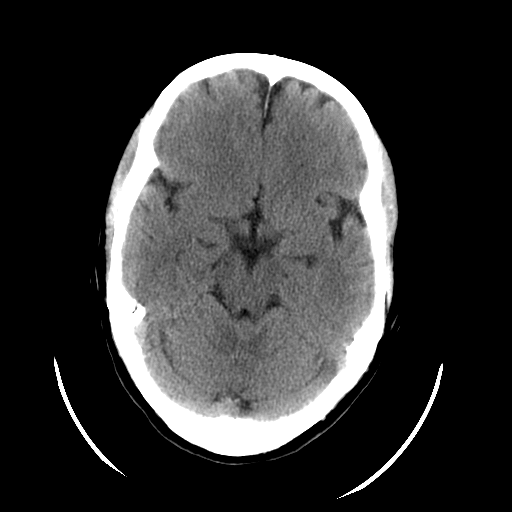

Supplement: S5 Data — (ZIP) [file pone.0295536.s006.zip › S6_Data/Tset set 1/1/21IM_0008-ID_b8df1f0ba.png]

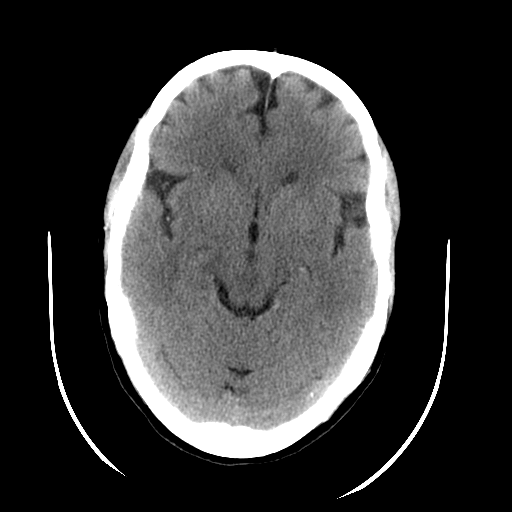

Supplement: S5 Data — (ZIP) [file pone.0295536.s006.zip › S6_Data/Tset set 1/1/21IM_0009-ID_c75e7f9b7.png]

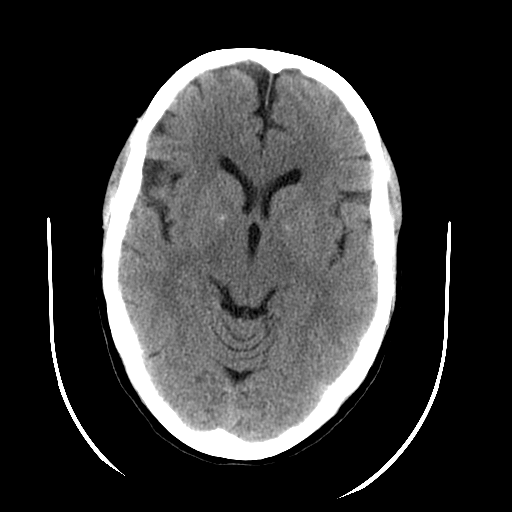

Supplement: S5 Data — (ZIP) [file pone.0295536.s006.zip › S6_Data/Tset set 1/1/21IM_0010-ID_a077f7c80.png]

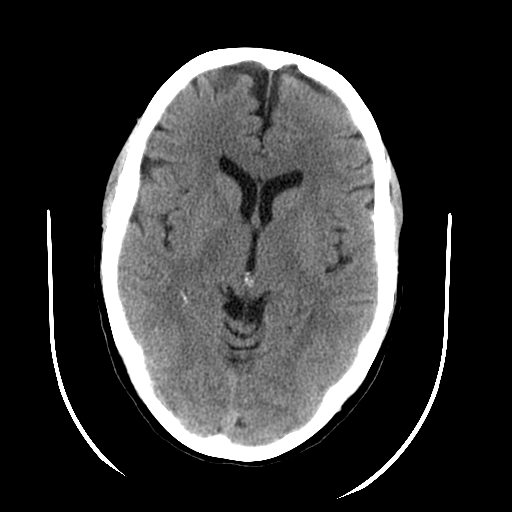

Supplement: S5 Data — (ZIP) [file pone.0295536.s006.zip › S6_Data/Tset set 1/1/21IM_0011-ID_8548bcca9.png]

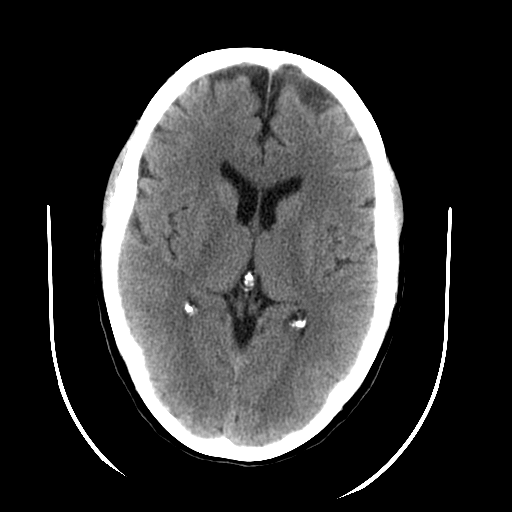

Supplement: S5 Data — (ZIP) [file pone.0295536.s006.zip › S6_Data/Tset set 1/1/21IM_0012-ID_41f026064.png]

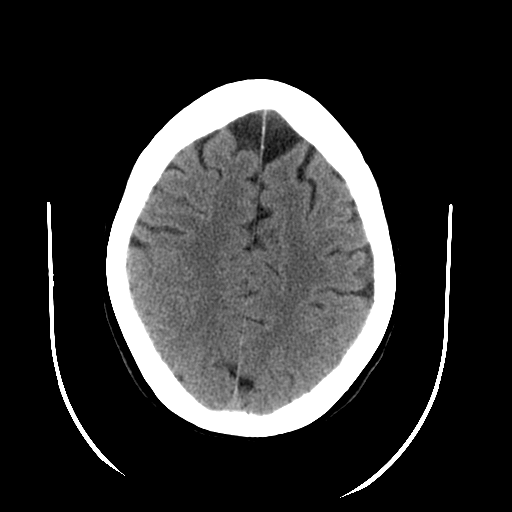

Supplement: S5 Data — (ZIP) [file pone.0295536.s006.zip › S6_Data/Tset set 1/1/21IM_0019-ID_d902fe9e7.png]

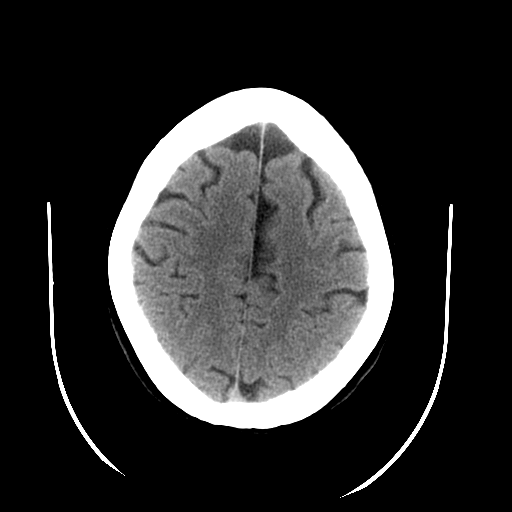

Supplement: S5 Data — (ZIP) [file pone.0295536.s006.zip › S6_Data/Tset set 1/1/21IM_0020-ID_80dfd0fbb.png]

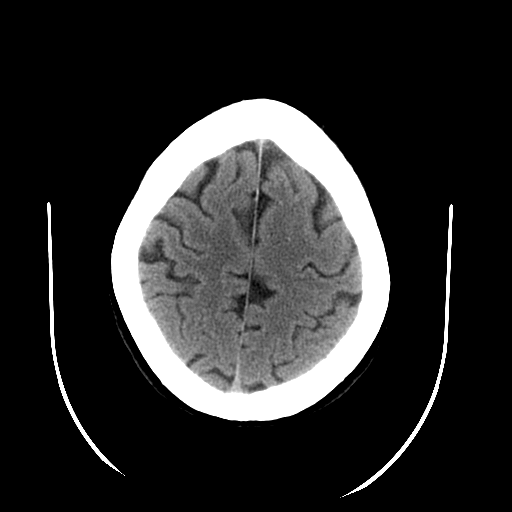

Supplement: S5 Data — (ZIP) [file pone.0295536.s006.zip › S6_Data/Tset set 1/1/21IM_0021-ID_b5d5e8061.png]

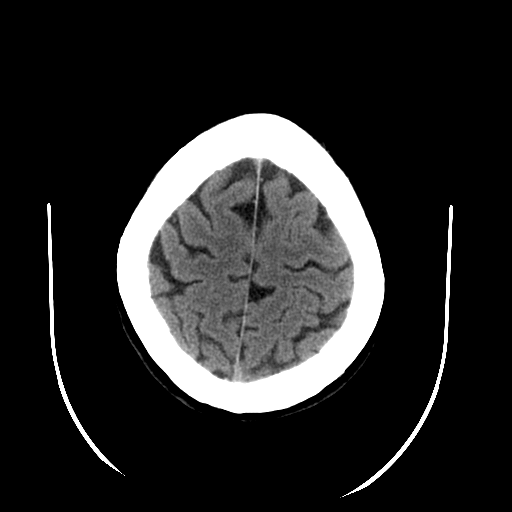

Supplement: S5 Data — (ZIP) [file pone.0295536.s006.zip › S6_Data/Tset set 1/1/21IM_0022-ID_067c20d14.png]

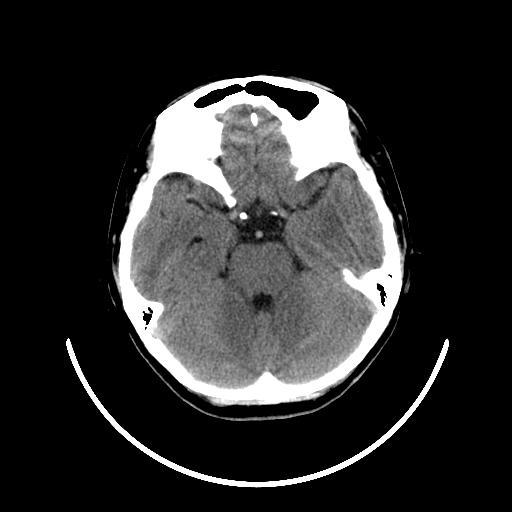

Supplement: S5 Data — (ZIP) [file pone.0295536.s006.zip › S6_Data/Tset set 1/1/22IM_0007-ID_a7cfbff57.png]

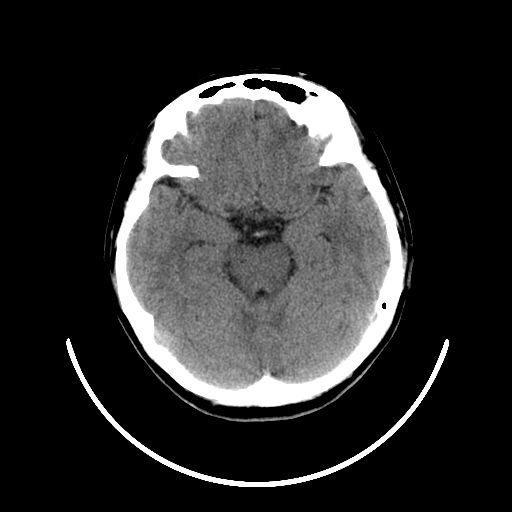

Supplement: S5 Data — (ZIP) [file pone.0295536.s006.zip › S6_Data/Tset set 1/1/22IM_0008-ID_6a68e05b4.png]

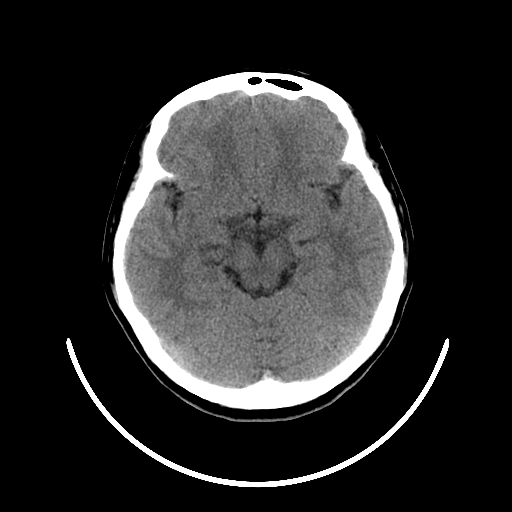

Supplement: S5 Data — (ZIP) [file pone.0295536.s006.zip › S6_Data/Tset set 1/1/22IM_0009-ID_44e5e8b59.png]

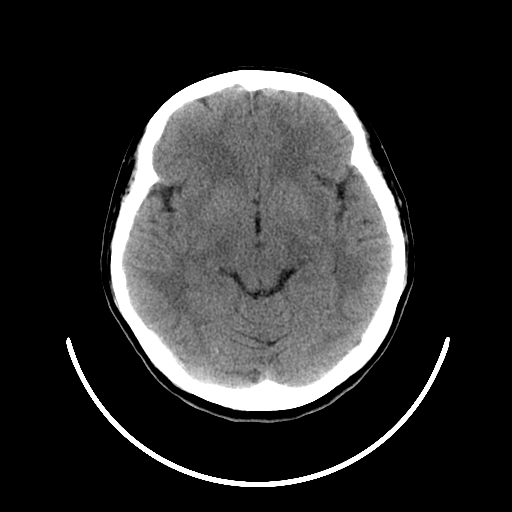

Supplement: S5 Data — (ZIP) [file pone.0295536.s006.zip › S6_Data/Tset set 1/1/22IM_0010-ID_3f0b950d5.png]

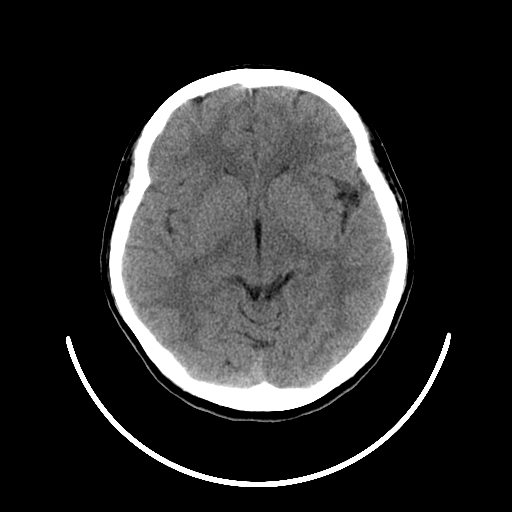

Supplement: S5 Data — (ZIP) [file pone.0295536.s006.zip › S6_Data/Tset set 1/1/22IM_0011-ID_608889648.png]

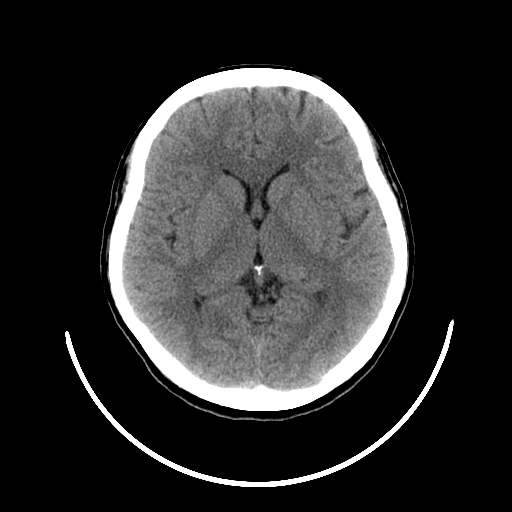

Supplement: S5 Data — (ZIP) [file pone.0295536.s006.zip › S6_Data/Tset set 1/1/22IM_0012-ID_4f2cb07a3.png]

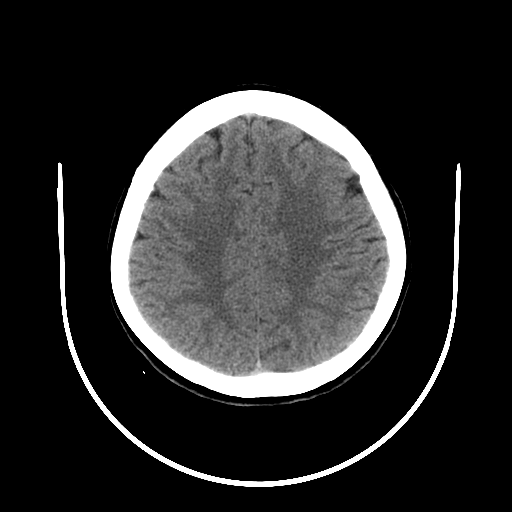

Supplement: S5 Data — (ZIP) [file pone.0295536.s006.zip › S6_Data/Tset set 1/1/22IM_0019-ID_1a3b4c62d.png]

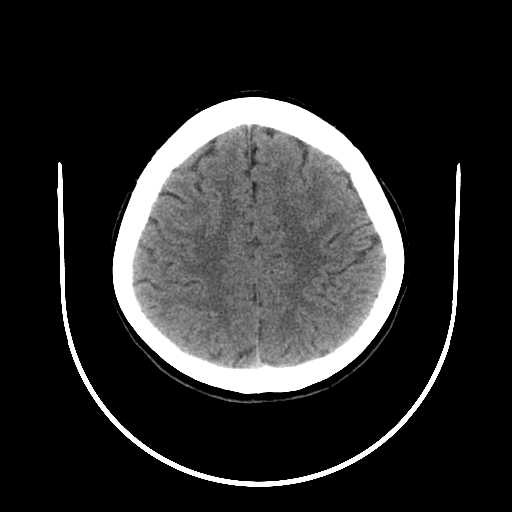

Supplement: S5 Data — (ZIP) [file pone.0295536.s006.zip › S6_Data/Tset set 1/1/22IM_0020-ID_95cf33751.png]

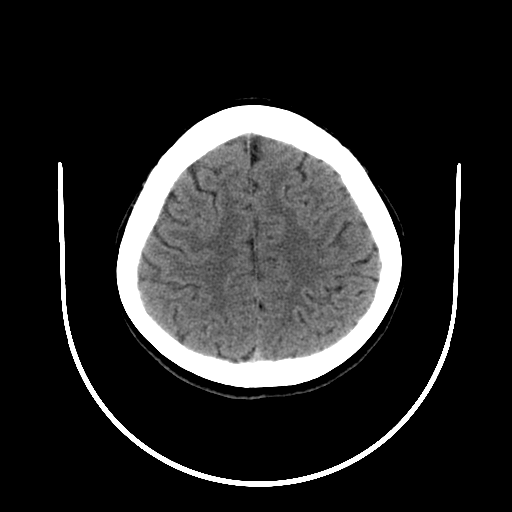

Supplement: S5 Data — (ZIP) [file pone.0295536.s006.zip › S6_Data/Tset set 1/1/22IM_0021-ID_07cd1db52.png]

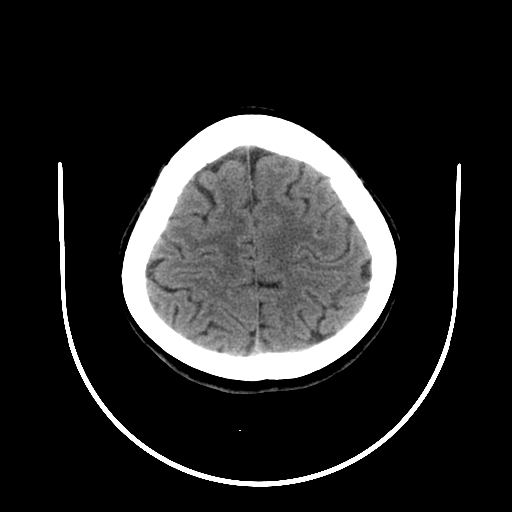

Supplement: S5 Data — (ZIP) [file pone.0295536.s006.zip › S6_Data/Tset set 1/1/22IM_0022-ID_4e24ed877.png]

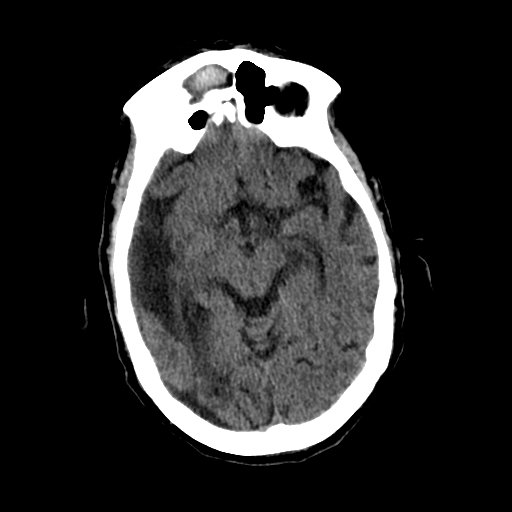

Supplement: S5 Data — (ZIP) [file pone.0295536.s006.zip › S6_Data/Tset set 1/1/23IM_0011-ID_deca969dd.png]

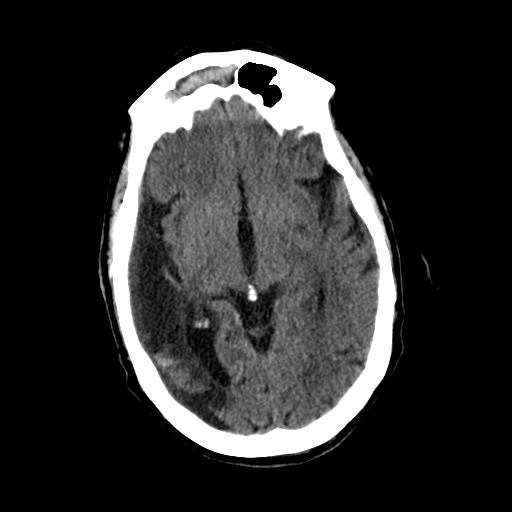

Supplement: S5 Data — (ZIP) [file pone.0295536.s006.zip › S6_Data/Tset set 1/1/23IM_0012-ID_0ee89f783.png]

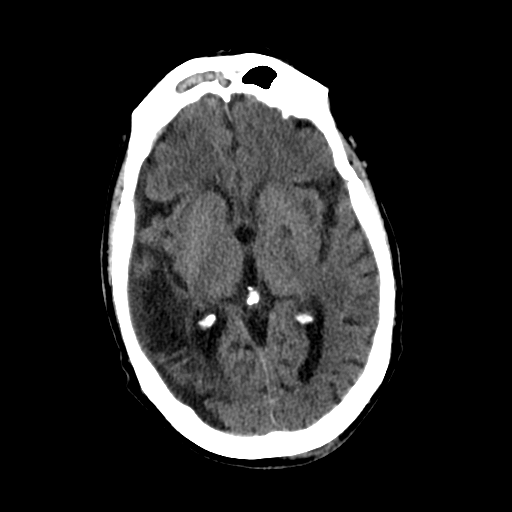

Supplement: S5 Data — (ZIP) [file pone.0295536.s006.zip › S6_Data/Tset set 1/1/23IM_0013-ID_e215d4a05.png]

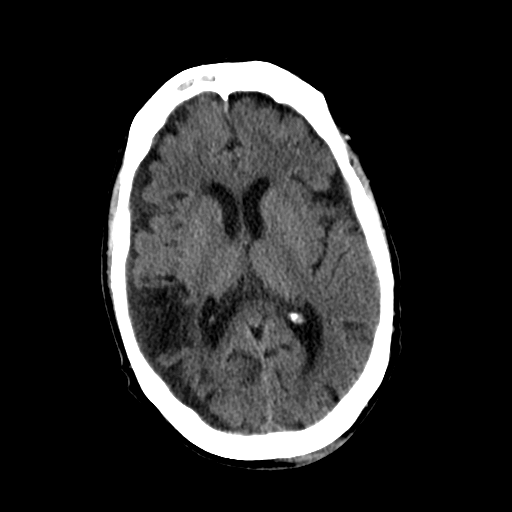

Supplement: S5 Data — (ZIP) [file pone.0295536.s006.zip › S6_Data/Tset set 1/1/23IM_0014-ID_bdcba14eb.png]

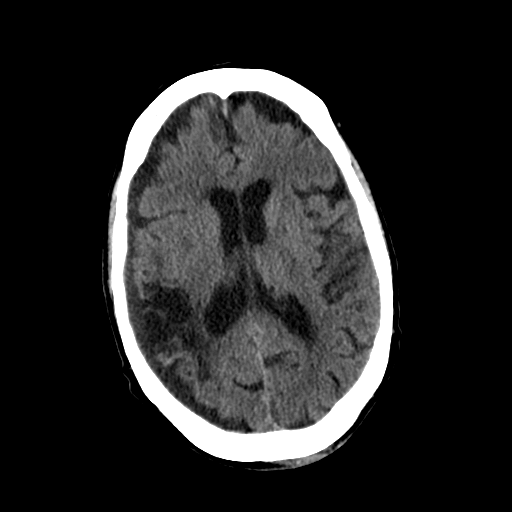

Supplement: S5 Data — (ZIP) [file pone.0295536.s006.zip › S6_Data/Tset set 1/1/23IM_0015-ID_93bde25de.png]

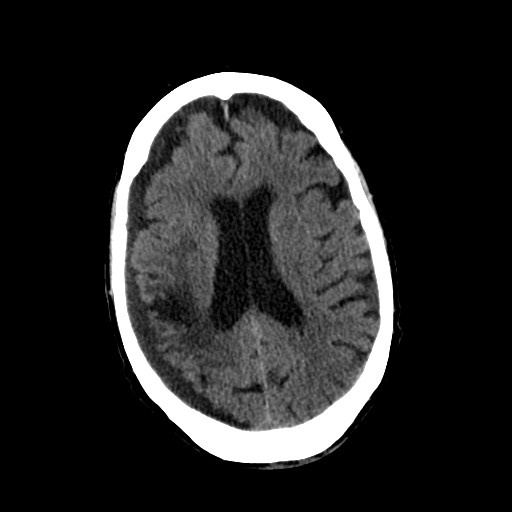

Supplement: S5 Data — (ZIP) [file pone.0295536.s006.zip › S6_Data/Tset set 1/1/23IM_0016-ID_ba5882375.png]

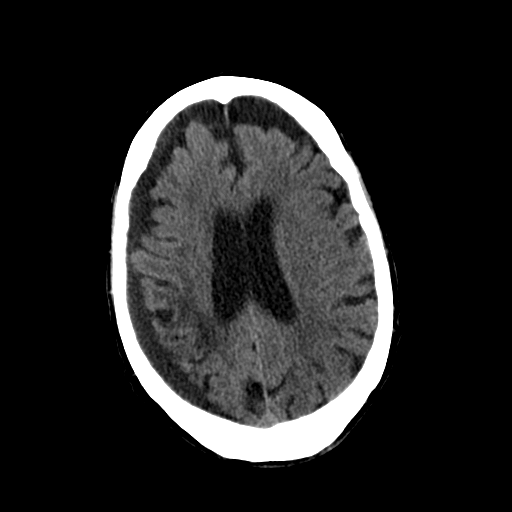

Supplement: S5 Data — (ZIP) [file pone.0295536.s006.zip › S6_Data/Tset set 1/1/23IM_0017-ID_a2bc527b4.png]

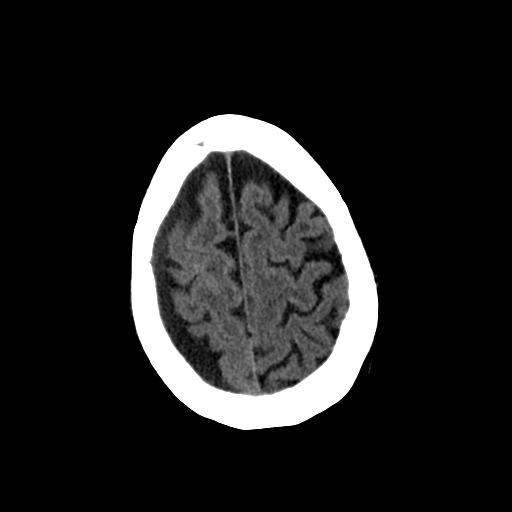

Supplement: S5 Data — (ZIP) [file pone.0295536.s006.zip › S6_Data/Tset set 1/1/23IM_0023-ID_f4771cd14.png]

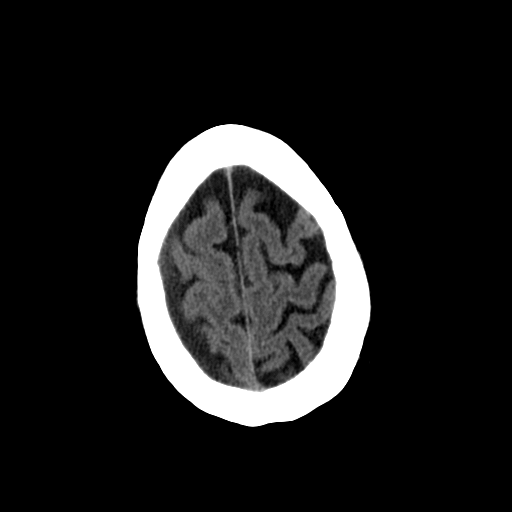

Supplement: S5 Data — (ZIP) [file pone.0295536.s006.zip › S6_Data/Tset set 1/1/23IM_0024-ID_179f1ccfa.png]

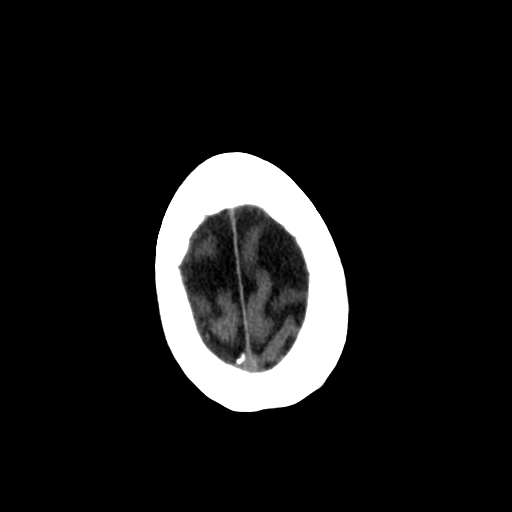

Supplement: S5 Data — (ZIP) [file pone.0295536.s006.zip › S6_Data/Tset set 1/1/23IM_0026-ID_0cd03e06c.png]

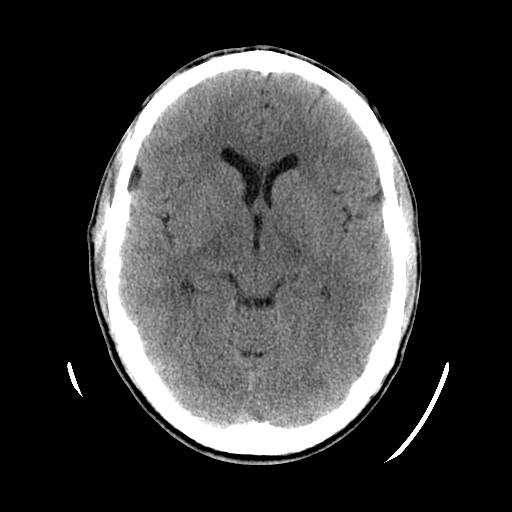

Supplement: S5 Data — (ZIP) [file pone.0295536.s006.zip › S6_Data/Tset set 1/1/3IM_0012-ID_f4ca3026f.png]

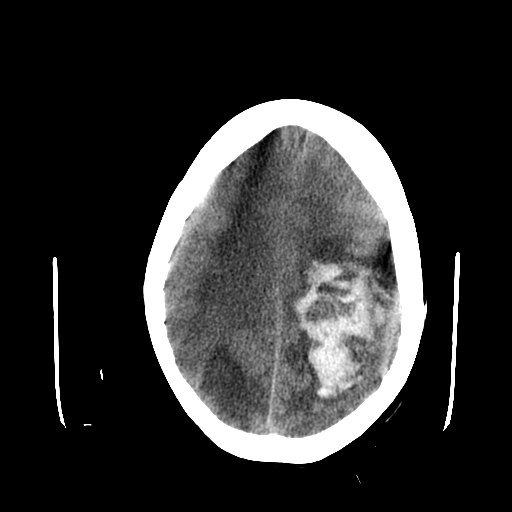

Supplement: S5 Data — (ZIP) [file pone.0295536.s006.zip › S6_Data/Tset set 1/1/IM_0019-ID_0e615c443.png]

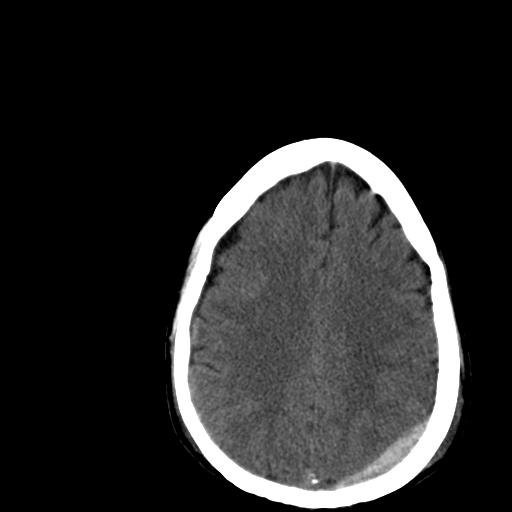

Supplement: S5 Data — (ZIP) [file pone.0295536.s006.zip › S6_Data/Tset set 1/1/IM_0019-ID_217b71668.png]

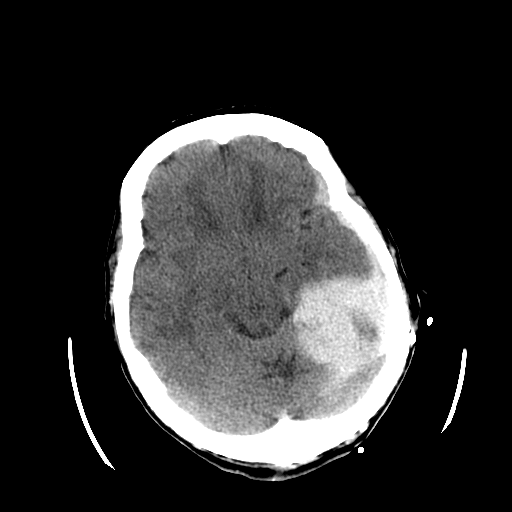

Supplement: S5 Data — (ZIP) [file pone.0295536.s006.zip › S6_Data/Tset set 1/1/IM_0019-ID_3fa02025a.png]

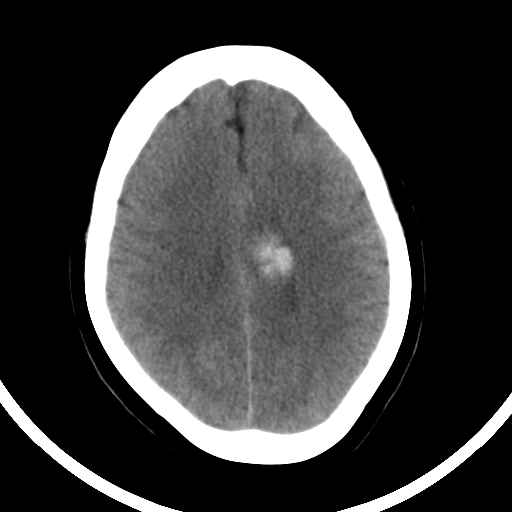

Supplement: S5 Data — (ZIP) [file pone.0295536.s006.zip › S6_Data/Tset set 1/1/IM_0019-ID_48b580bb8.png]

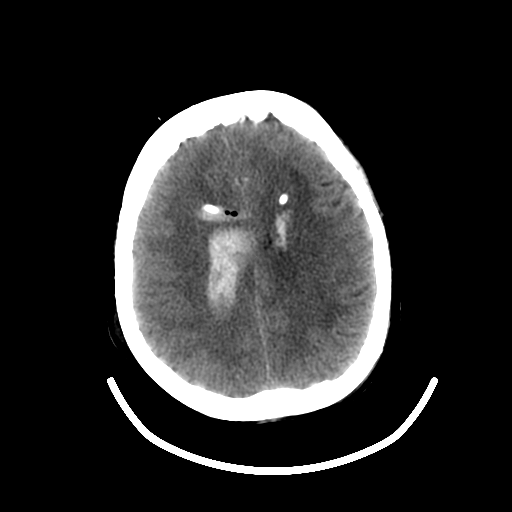

Supplement: S5 Data — (ZIP) [file pone.0295536.s006.zip › S6_Data/Tset set 1/1/IM_0019-ID_8be6ff2c9.png]

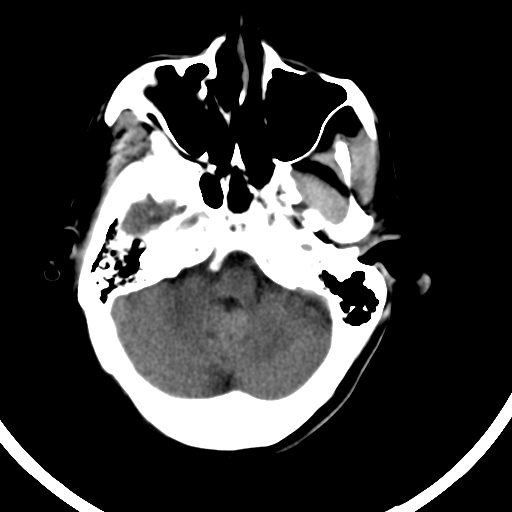

Supplement: S5 Data — (ZIP) [file pone.0295536.s006.zip › S6_Data/Tset set 1/2/18IM_0007-ID_55086bb1e.png]

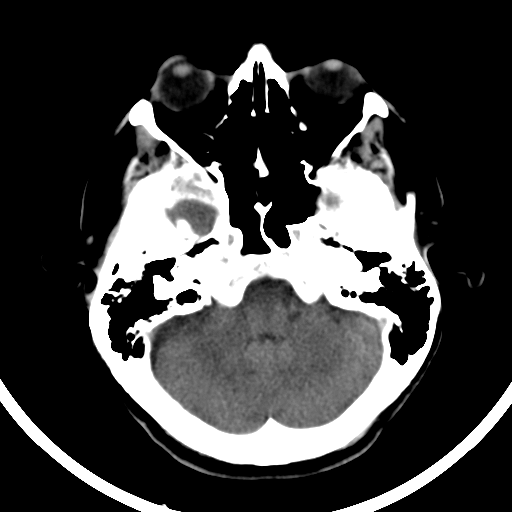

Supplement: S5 Data — (ZIP) [file pone.0295536.s006.zip › S6_Data/Tset set 1/2/29IM_0010-ID_81228b7a1.png]

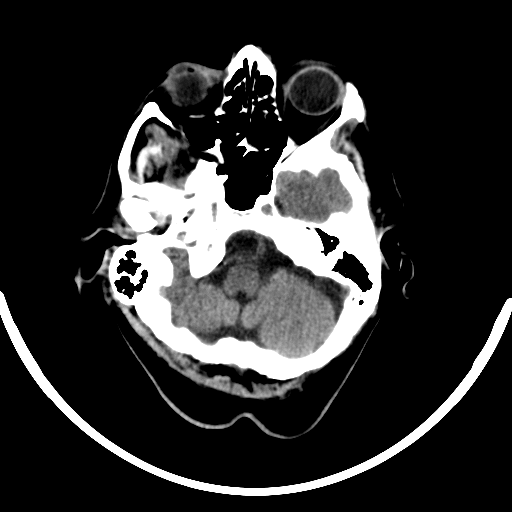

Supplement: S5 Data — (ZIP) [file pone.0295536.s006.zip › S6_Data/Tset set 1/2/44IM_0003-ID_c7d7c263b.png]

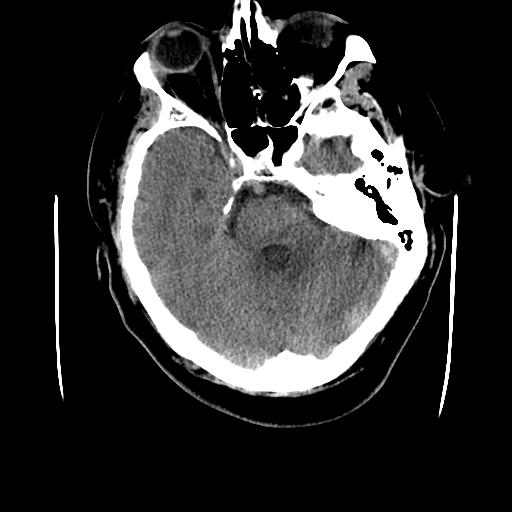

Supplement: S5 Data — (ZIP) [file pone.0295536.s006.zip › S6_Data/Tset set 1/2/61IM_0008-ID_483988457.png]

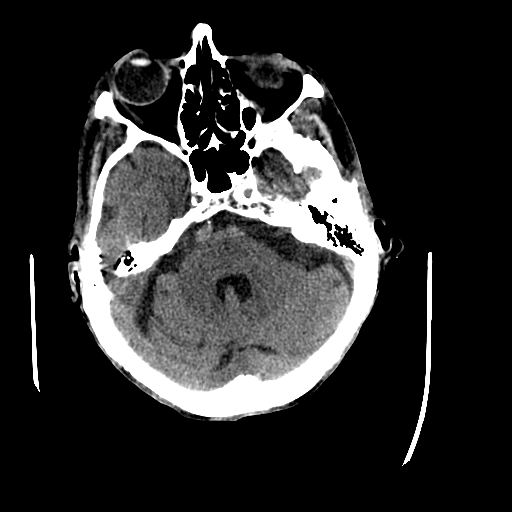

Supplement: S5 Data — (ZIP) [file pone.0295536.s006.zip › S6_Data/Tset set 1/2/68IM_0014-ID_3b8cd18f2.png]

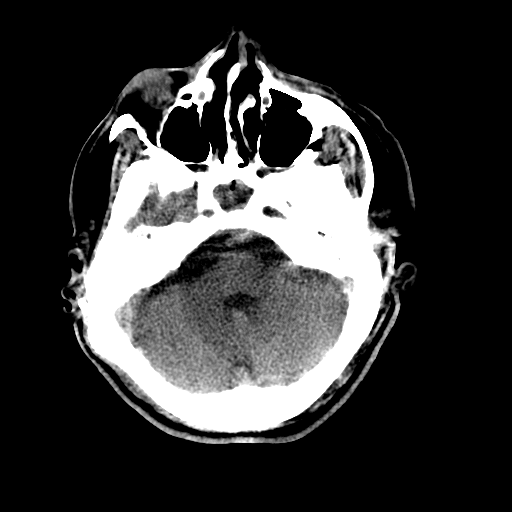

Supplement: S5 Data — (ZIP) [file pone.0295536.s006.zip › S6_Data/Tset set 1/2/6IM_0008-ID_a22adc787.png]

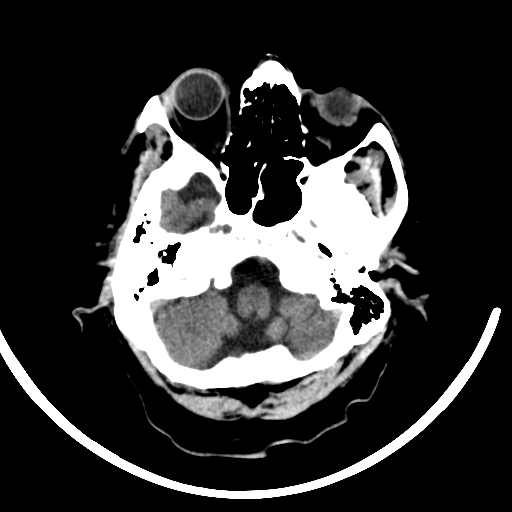

Supplement: S5 Data — (ZIP) [file pone.0295536.s006.zip › S6_Data/Tset set 1/2/IM_0003-ID_b99fbb54c.png]

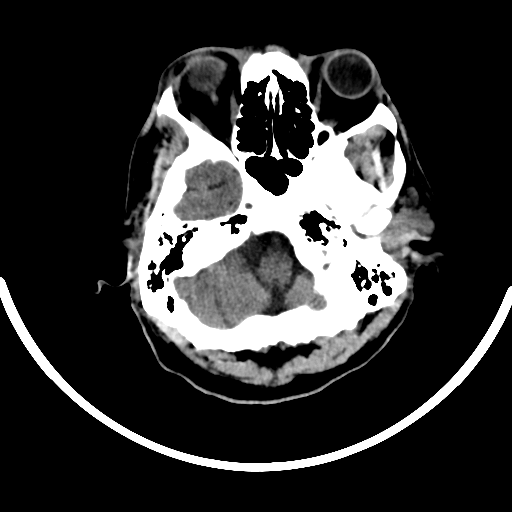

Supplement: S5 Data — (ZIP) [file pone.0295536.s006.zip › S6_Data/Tset set 1/2/IM_0003-ID_e75a2af72.png]

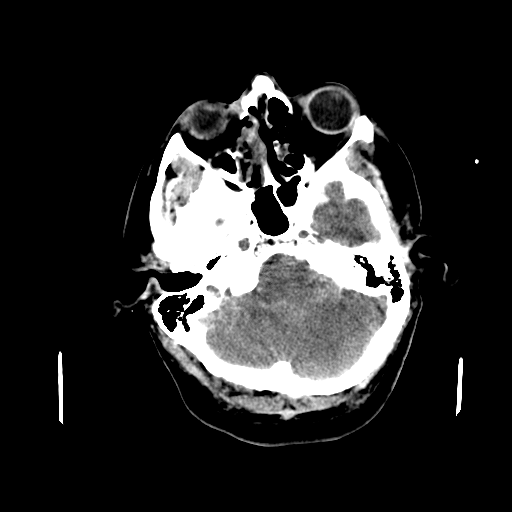

Supplement: S5 Data — (ZIP) [file pone.0295536.s006.zip › S6_Data/Tset set 1/2/IM_0005-ID_b1ed328ce.png]

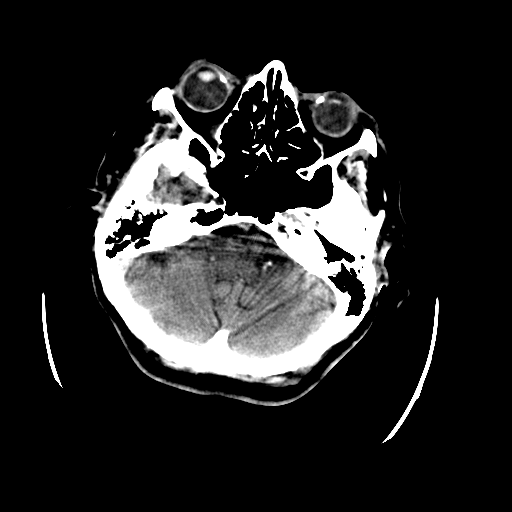

Supplement: S5 Data — (ZIP) [file pone.0295536.s006.zip › S6_Data/Tset set 1/2/IM_0005-ID_f32d24fbf.png]

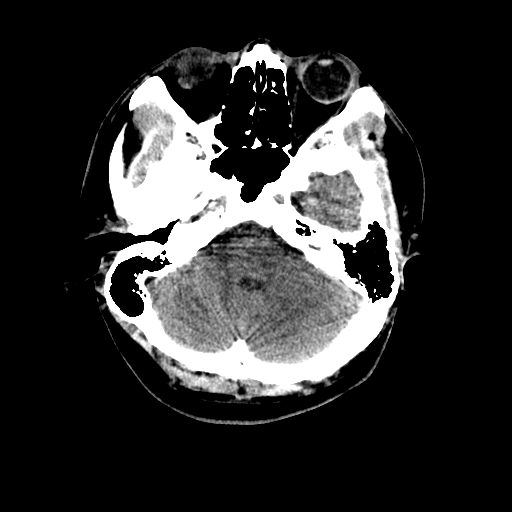

Supplement: S5 Data — (ZIP) [file pone.0295536.s006.zip › S6_Data/Tset set 1/2/IM_0006-ID_9b786f1ed.png]

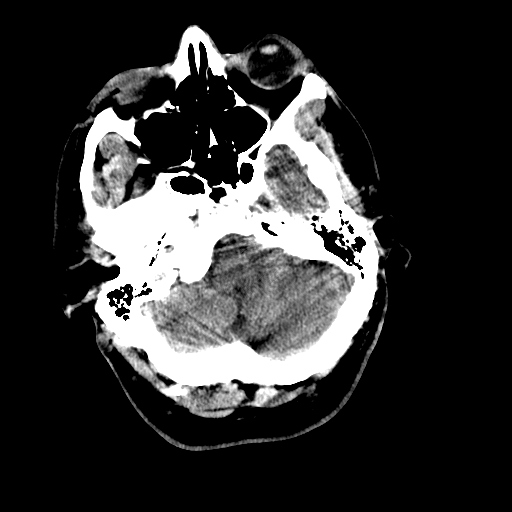

Supplement: S5 Data — (ZIP) [file pone.0295536.s006.zip › S6_Data/Tset set 1/2/IM_0006-ID_bcb51cb4c.png]

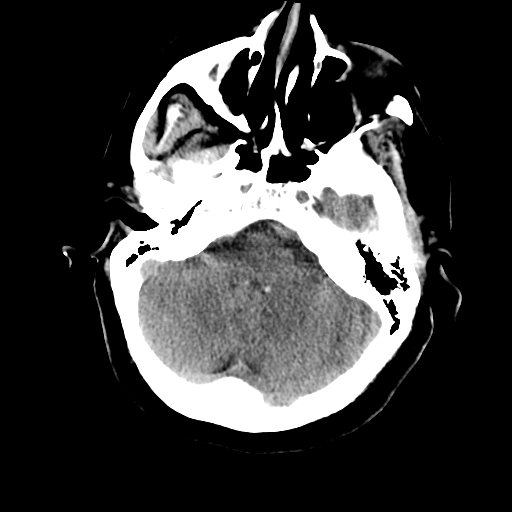

Supplement: S5 Data — (ZIP) [file pone.0295536.s006.zip › S6_Data/Tset set 1/2/IM_0007-ID_432b67d83.png]

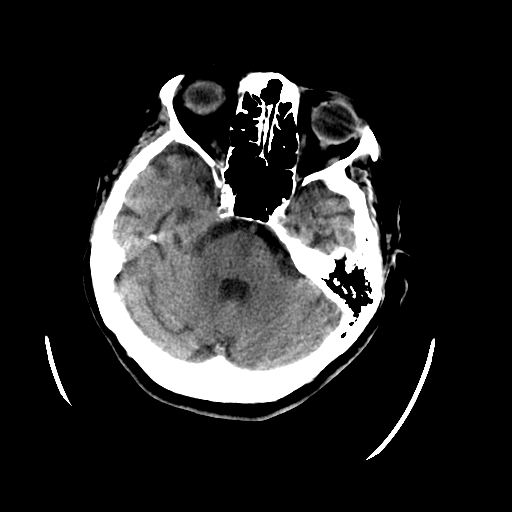

Supplement: S5 Data — (ZIP) [file pone.0295536.s006.zip › S6_Data/Tset set 1/2/IM_0007-ID_52682708d.png]

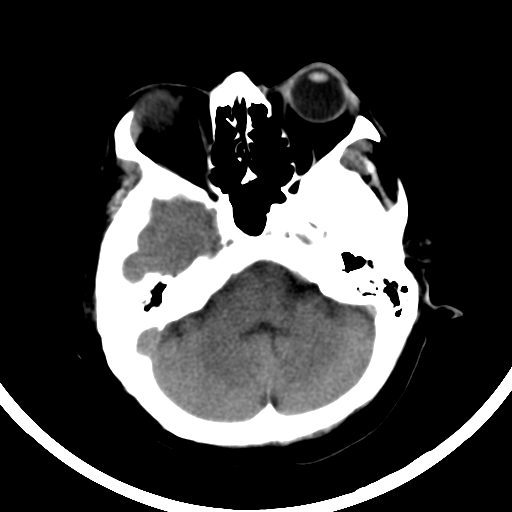

Supplement: S5 Data — (ZIP) [file pone.0295536.s006.zip › S6_Data/Tset set 1/2/IM_0007-ID_5406aa980.png]

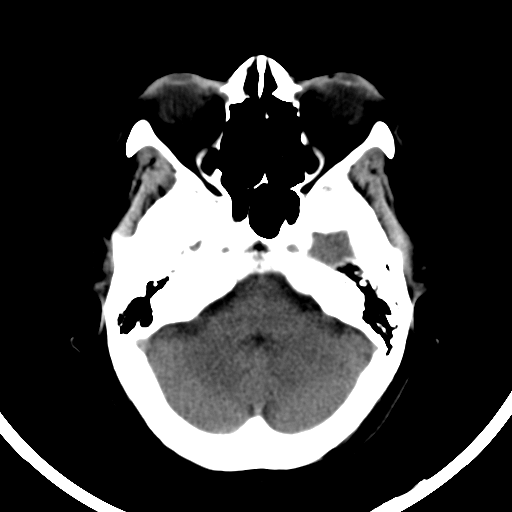

Supplement: S5 Data — (ZIP) [file pone.0295536.s006.zip › S6_Data/Tset set 1/2/IM_0007-ID_d0fc0d5c8.png]

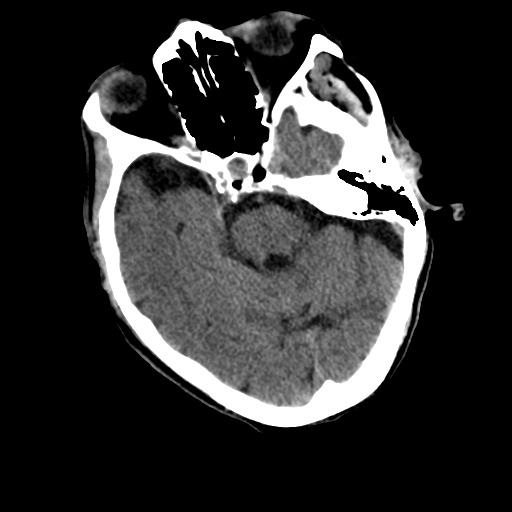

Supplement: S5 Data — (ZIP) [file pone.0295536.s006.zip › S6_Data/Tset set 1/2/IM_0008-ID_6e028f83d.png]

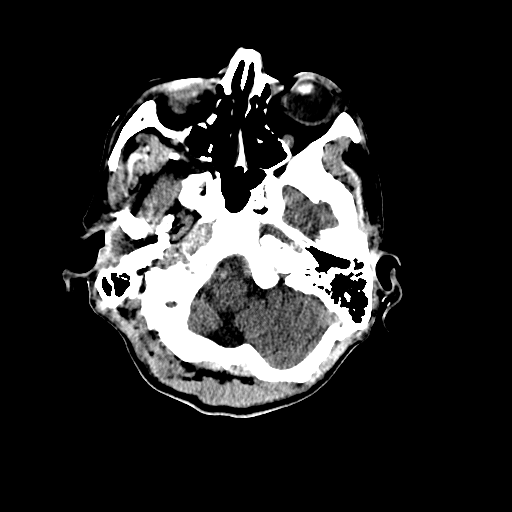

Supplement: S5 Data — (ZIP) [file pone.0295536.s006.zip › S6_Data/Tset set 1/2/IM_0008-ID_72082ff6a.png]

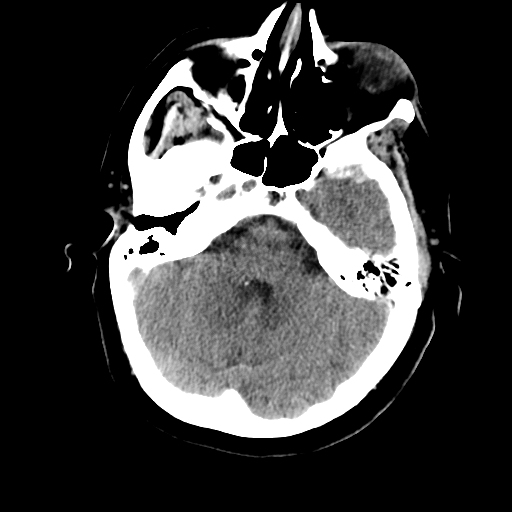

Supplement: S5 Data — (ZIP) [file pone.0295536.s006.zip › S6_Data/Tset set 1/2/IM_0008-ID_eb52411e6.png]

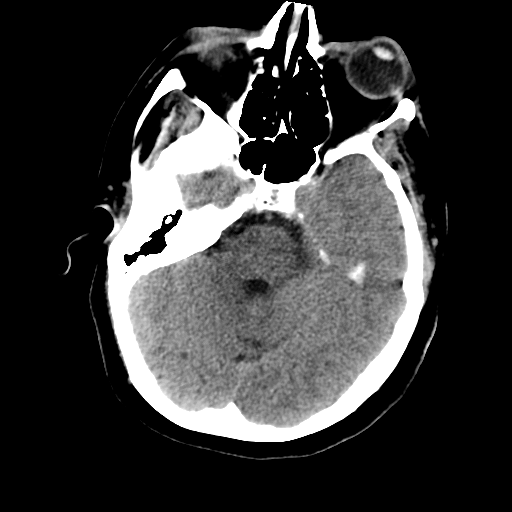

Supplement: S5 Data — (ZIP) [file pone.0295536.s006.zip › S6_Data/Tset set 1/2/IM_0009-ID_113d92efc.png]

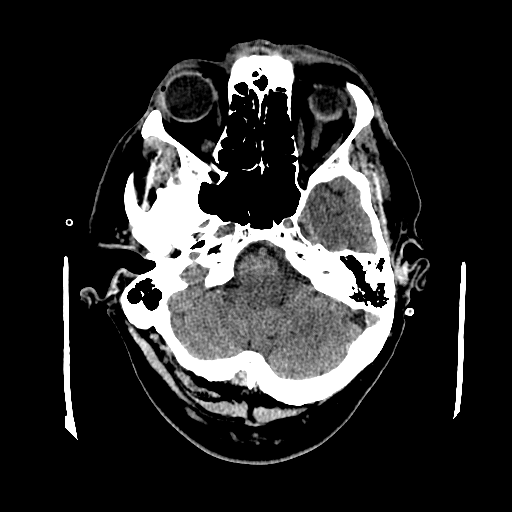

Supplement: S5 Data — (ZIP) [file pone.0295536.s006.zip › S6_Data/Tset set 1/2/IM_0010-ID_37b7ec0c7.png]

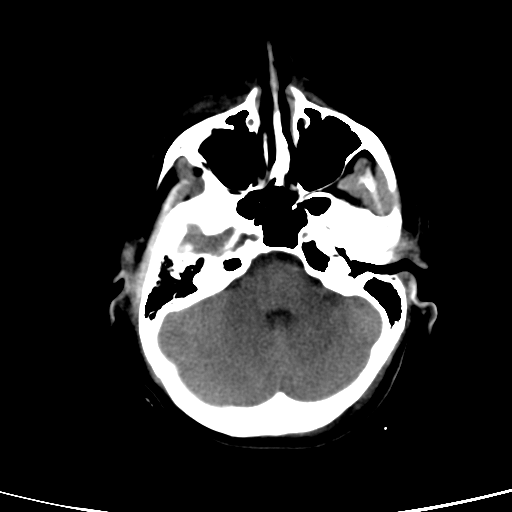

Supplement: S5 Data — (ZIP) [file pone.0295536.s006.zip › S6_Data/Tset set 1/2/IM_0010-ID_8e616f79c.png]

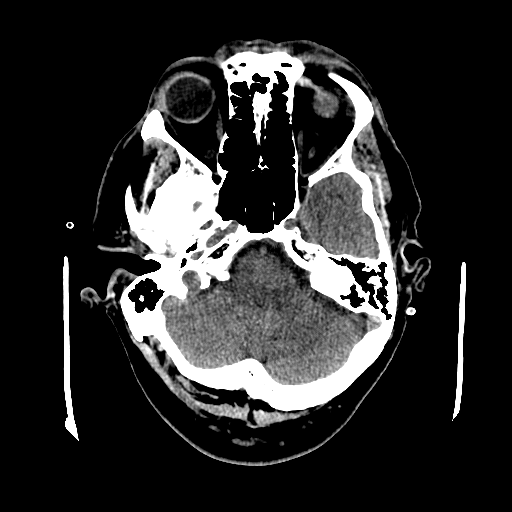

Supplement: S5 Data — (ZIP) [file pone.0295536.s006.zip › S6_Data/Tset set 1/2/IM_0011-ID_4c5e1229a.png]

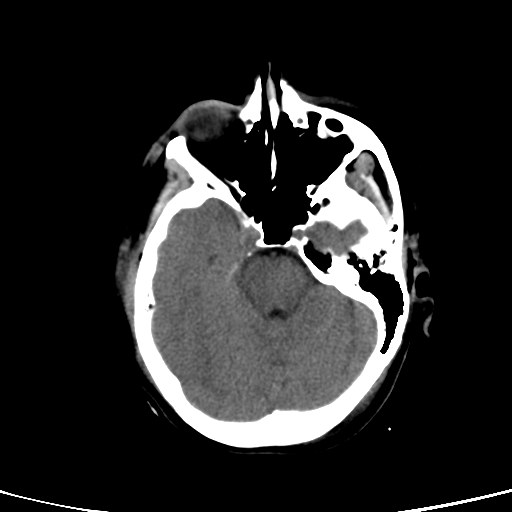

Supplement: S5 Data — (ZIP) [file pone.0295536.s006.zip › S6_Data/Tset set 1/2/IM_0012-ID_fa0e3e666.png]

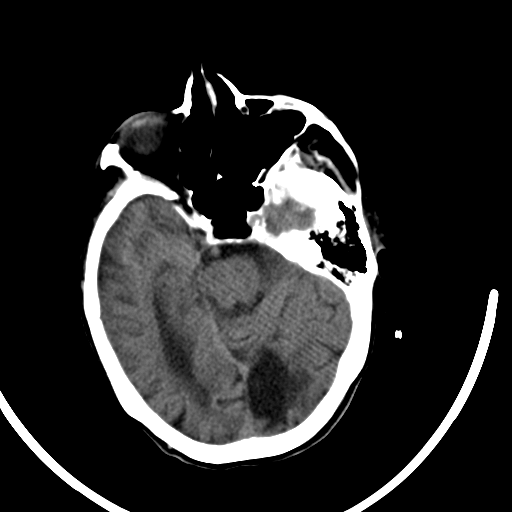

Supplement: S5 Data — (ZIP) [file pone.0295536.s006.zip › S6_Data/Tset set 1/2/IM_0017-ID_1f63529d7.png]

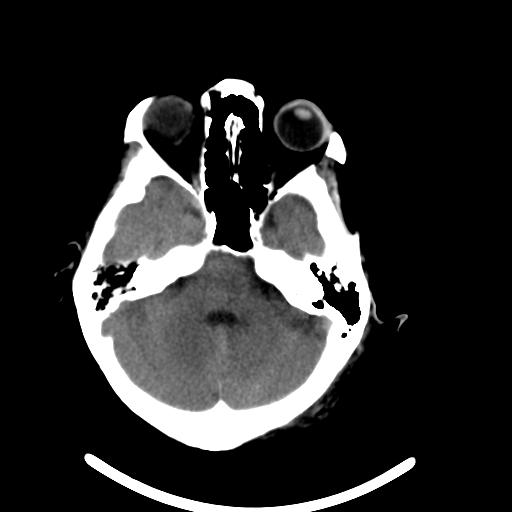

Supplement: S5 Data — (ZIP) [file pone.0295536.s006.zip › S6_Data/Tset set 1/3/10IM_0011-ID_159b9b980.png]

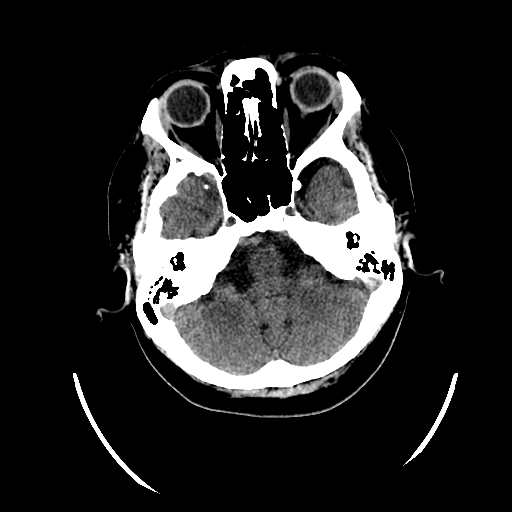

Supplement: S5 Data — (ZIP) [file pone.0295536.s006.zip › S6_Data/Tset set 1/3/124IM_0009-ID_ef2926f11.png]

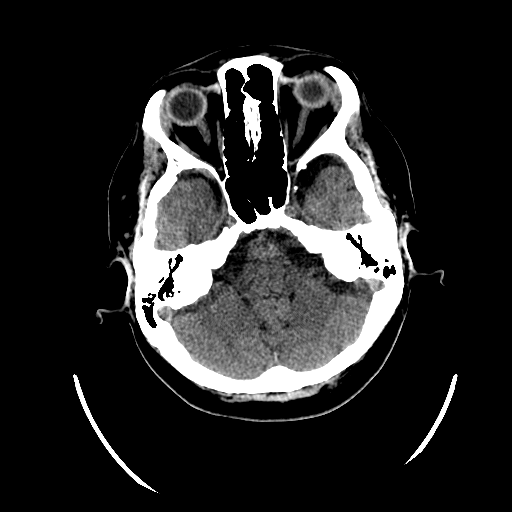

Supplement: S5 Data — (ZIP) [file pone.0295536.s006.zip › S6_Data/Tset set 1/3/124IM_0010-ID_3a348c321.png]
