# Supplementary figures and images for: Adaptive mask-based brain extraction method for head CT images (part 12 of 14)
Source: PLoS One. 2024 Mar 11;19(3):e0295536. doi: 10.1371/journal.pone.0295536 (PMC10927156; doi:10.1371/journal.pone.0295536)

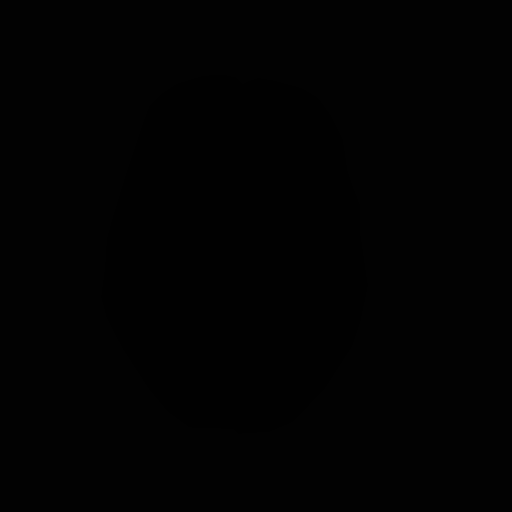

Supplement: S6 Data — (ZIP) [file pone.0295536.s007.zip › S7_Data/Tset set 2/Label/Label_77.png]

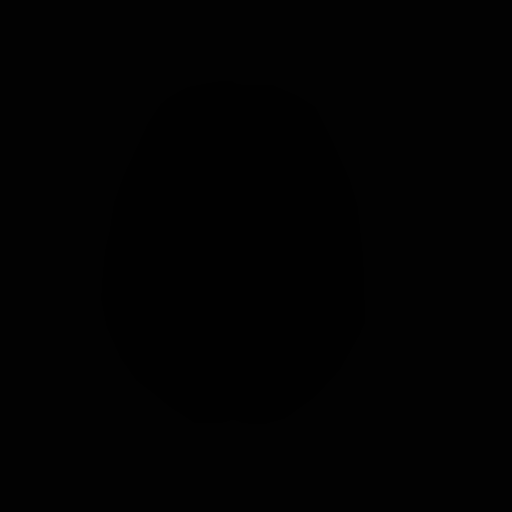

Supplement: S6 Data — (ZIP) [file pone.0295536.s007.zip › S7_Data/Tset set 2/Label/Label_78.png]

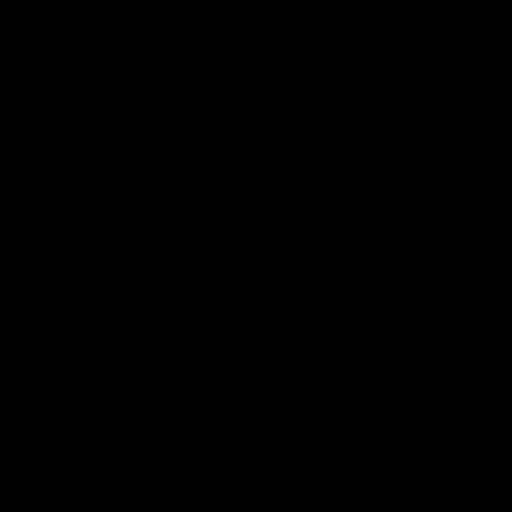

Supplement: S6 Data — (ZIP) [file pone.0295536.s007.zip › S7_Data/Tset set 2/Label/Label_79.png]

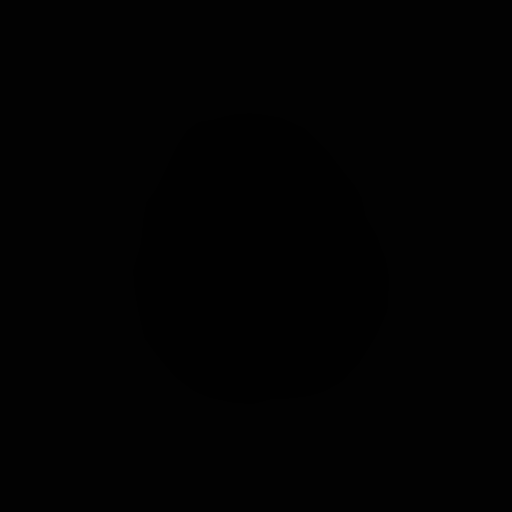

Supplement: S6 Data — (ZIP) [file pone.0295536.s007.zip › S7_Data/Tset set 2/Label/Label_8.png]

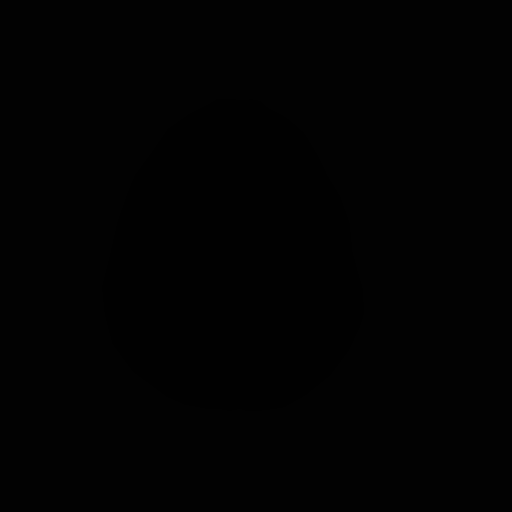

Supplement: S6 Data — (ZIP) [file pone.0295536.s007.zip › S7_Data/Tset set 2/Label/Label_80.png]

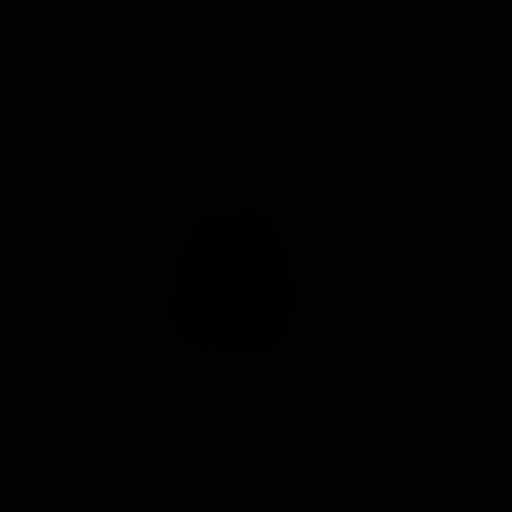

Supplement: S6 Data — (ZIP) [file pone.0295536.s007.zip › S7_Data/Tset set 2/Label/Label_81.png]

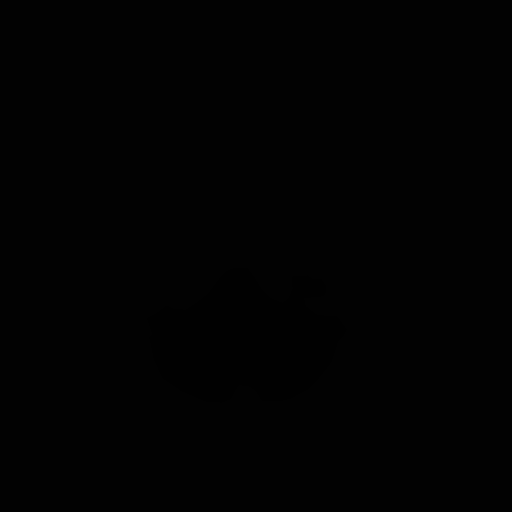

Supplement: S6 Data — (ZIP) [file pone.0295536.s007.zip › S7_Data/Tset set 2/Label/Label_82.png]

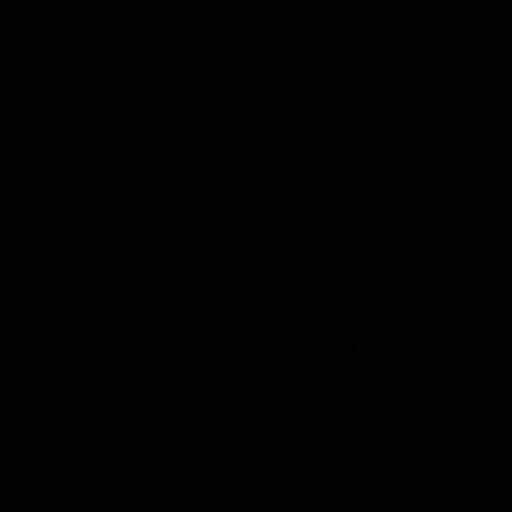

Supplement: S6 Data — (ZIP) [file pone.0295536.s007.zip › S7_Data/Tset set 2/Label/Label_83.png]

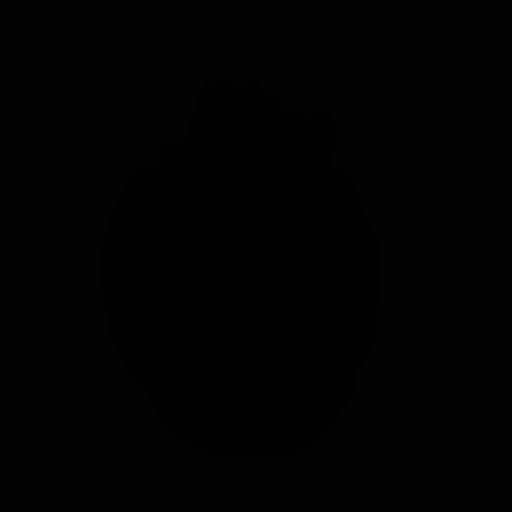

Supplement: S6 Data — (ZIP) [file pone.0295536.s007.zip › S7_Data/Tset set 2/Label/Label_84.png]

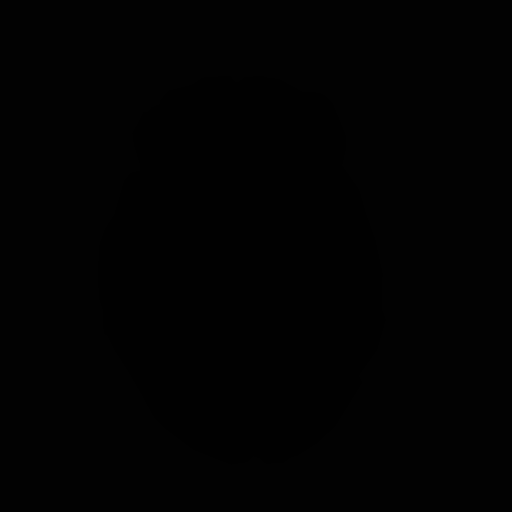

Supplement: S6 Data — (ZIP) [file pone.0295536.s007.zip › S7_Data/Tset set 2/Label/Label_85.png]

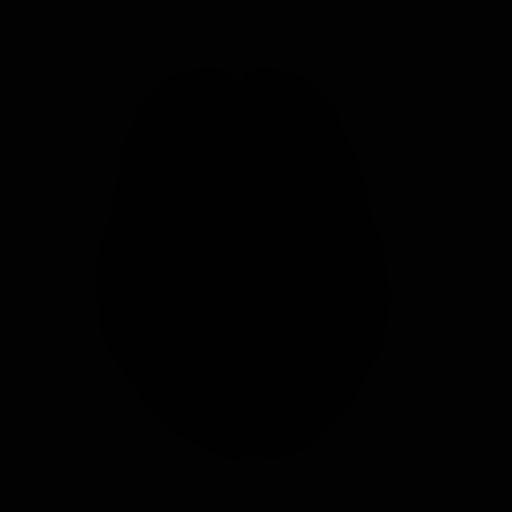

Supplement: S6 Data — (ZIP) [file pone.0295536.s007.zip › S7_Data/Tset set 2/Label/Label_86.png]

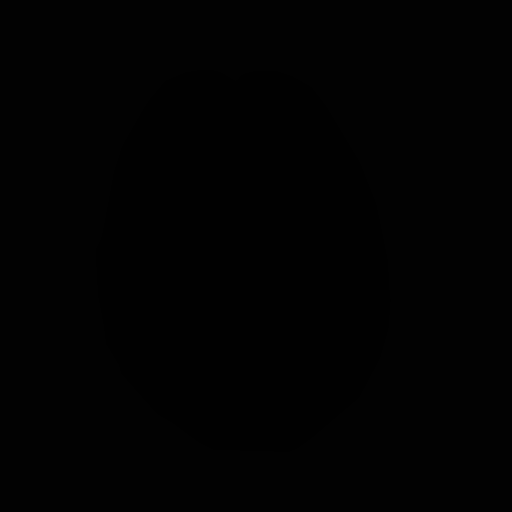

Supplement: S6 Data — (ZIP) [file pone.0295536.s007.zip › S7_Data/Tset set 2/Label/Label_87.png]

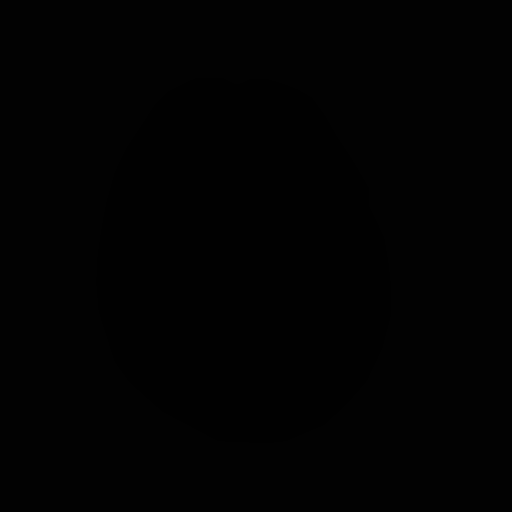

Supplement: S6 Data — (ZIP) [file pone.0295536.s007.zip › S7_Data/Tset set 2/Label/Label_88.png]

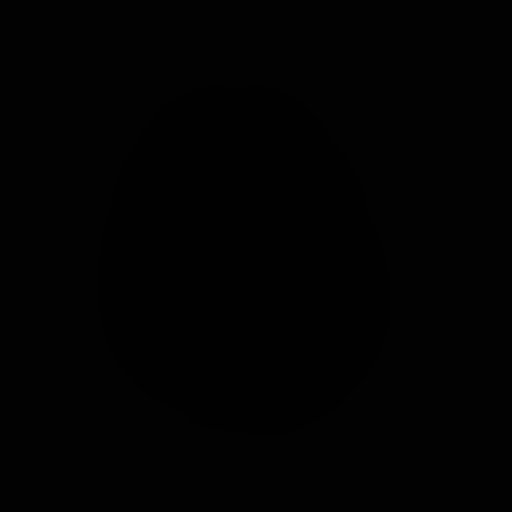

Supplement: S6 Data — (ZIP) [file pone.0295536.s007.zip › S7_Data/Tset set 2/Label/Label_89.png]

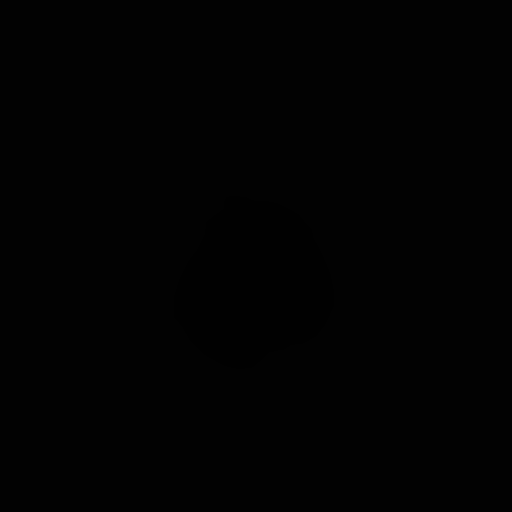

Supplement: S6 Data — (ZIP) [file pone.0295536.s007.zip › S7_Data/Tset set 2/Label/Label_9.png]

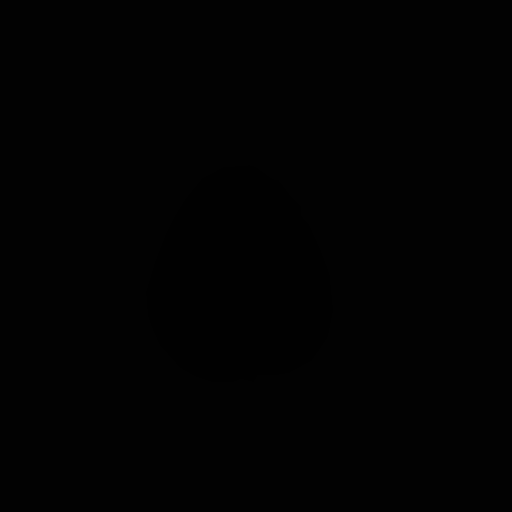

Supplement: S6 Data — (ZIP) [file pone.0295536.s007.zip › S7_Data/Tset set 2/Label/Label_90.png]

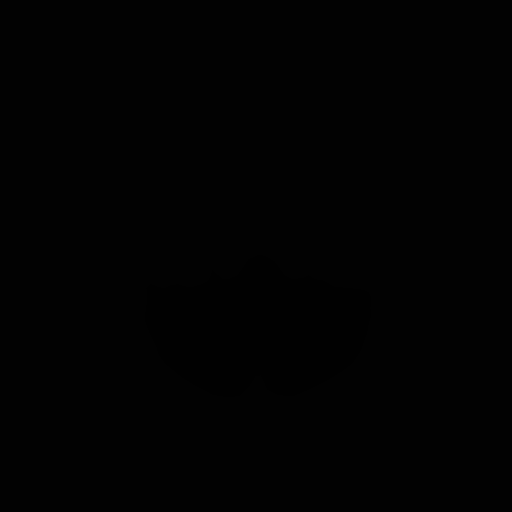

Supplement: S6 Data — (ZIP) [file pone.0295536.s007.zip › S7_Data/Tset set 2/Label/Label_91.png]

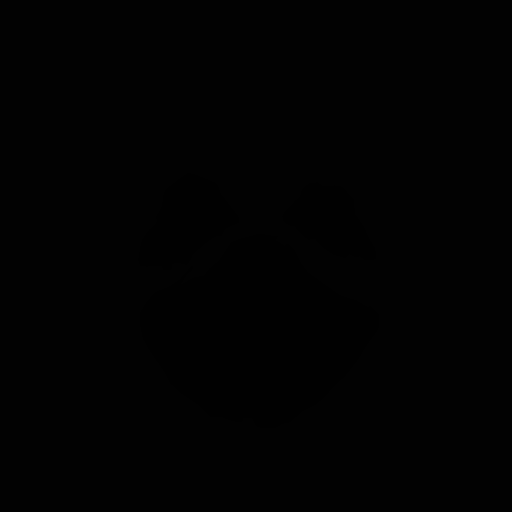

Supplement: S6 Data — (ZIP) [file pone.0295536.s007.zip › S7_Data/Tset set 2/Label/Label_92.png]

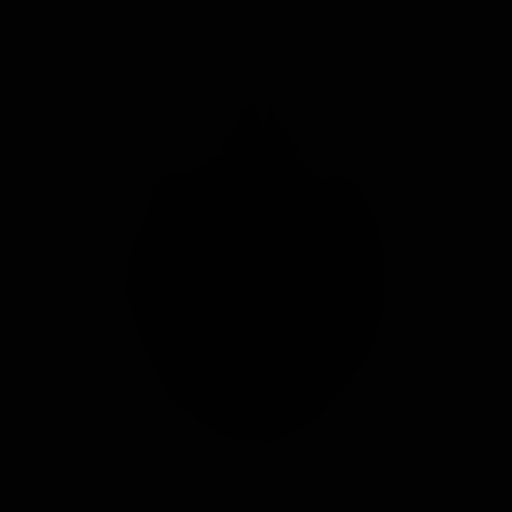

Supplement: S6 Data — (ZIP) [file pone.0295536.s007.zip › S7_Data/Tset set 2/Label/Label_93.png]

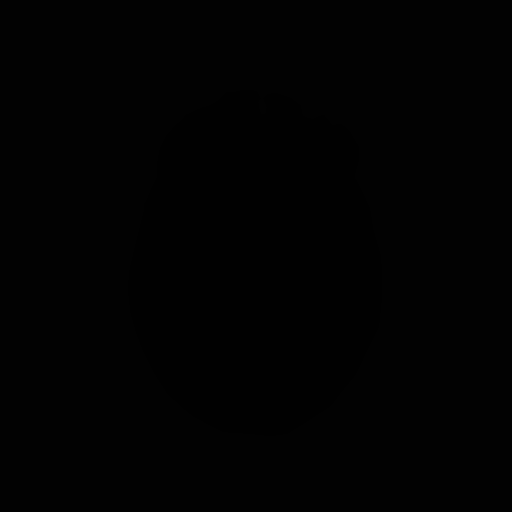

Supplement: S6 Data — (ZIP) [file pone.0295536.s007.zip › S7_Data/Tset set 2/Label/Label_94.png]

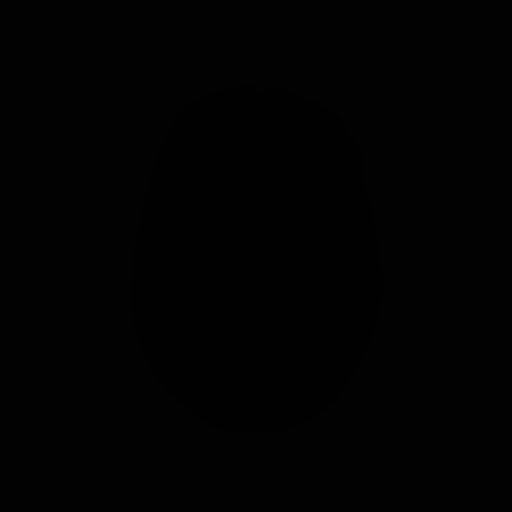

Supplement: S6 Data — (ZIP) [file pone.0295536.s007.zip › S7_Data/Tset set 2/Label/Label_95.png]

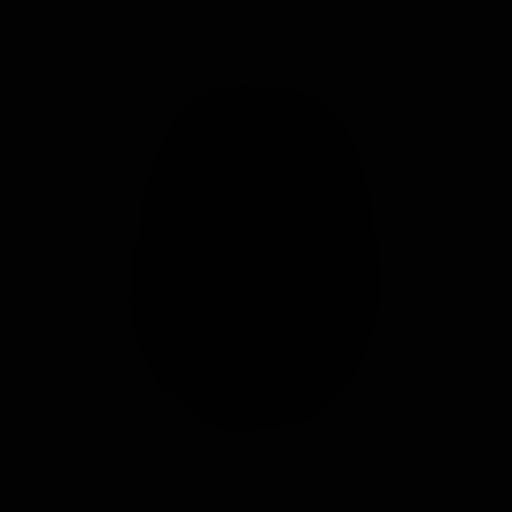

Supplement: S6 Data — (ZIP) [file pone.0295536.s007.zip › S7_Data/Tset set 2/Label/Label_96.png]

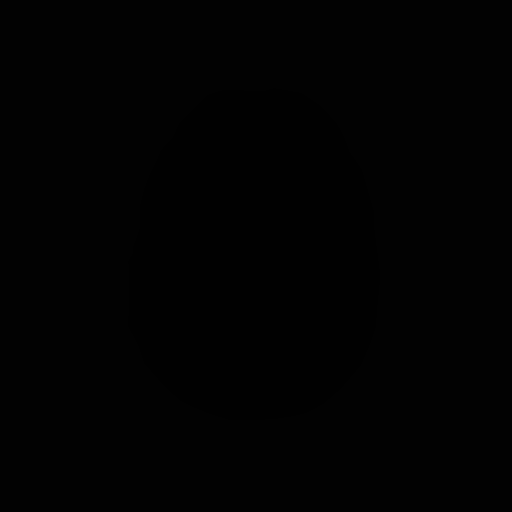

Supplement: S6 Data — (ZIP) [file pone.0295536.s007.zip › S7_Data/Tset set 2/Label/Label_97.png]

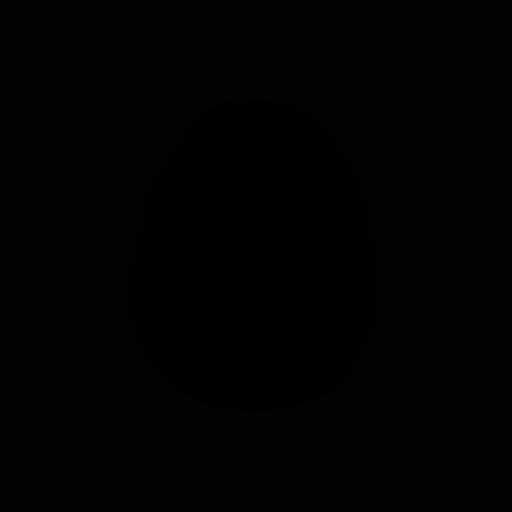

Supplement: S6 Data — (ZIP) [file pone.0295536.s007.zip › S7_Data/Tset set 2/Label/Label_98.png]

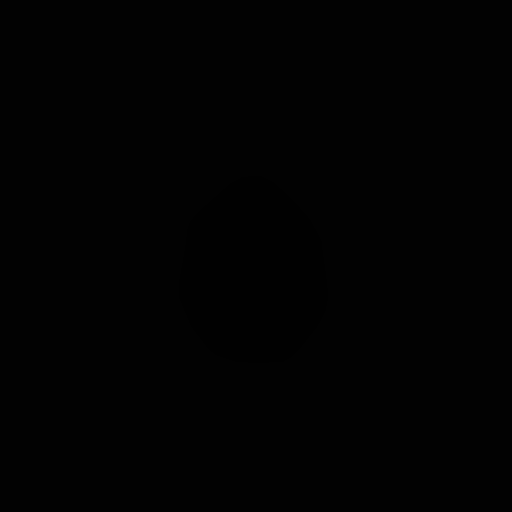

Supplement: S6 Data — (ZIP) [file pone.0295536.s007.zip › S7_Data/Tset set 2/Label/Label_99.png]

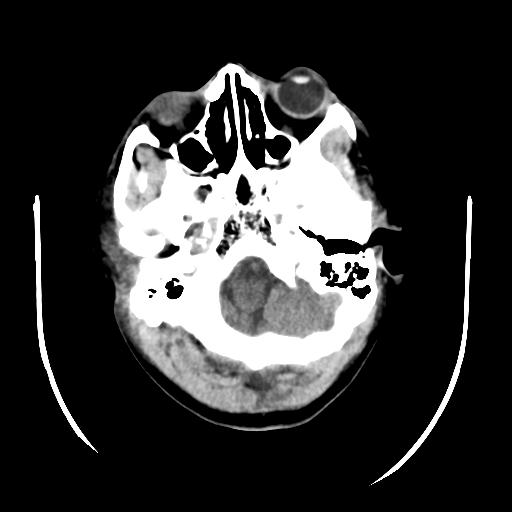

Supplement: S6 Data — (ZIP) [file pone.0295536.s007.zip › S7_Data/Tset set 2/Set/Label_1.png]

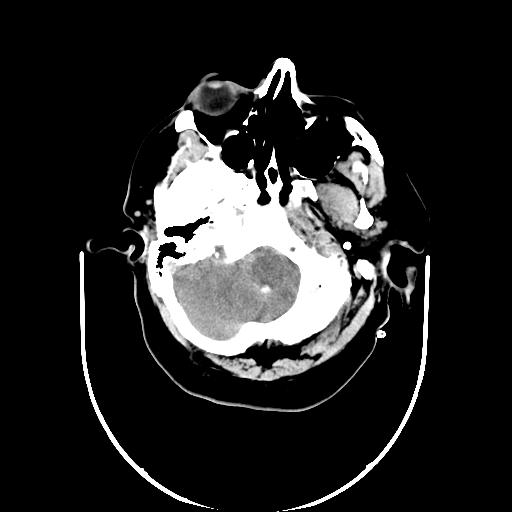

Supplement: S6 Data — (ZIP) [file pone.0295536.s007.zip › S7_Data/Tset set 2/Set/Label_10.png]

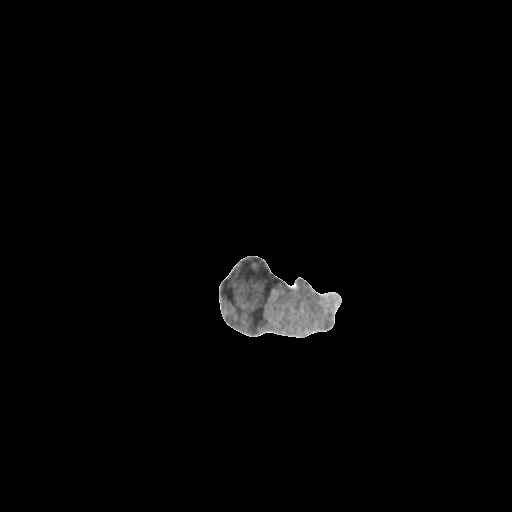

Supplement: S1 Fig — (ZIP) [file pone.0295536.s008.zip › S8_Fig/Segmentation result of AMBBEM with three FCNs in test set 2/AMBBEM/Label_1.png]

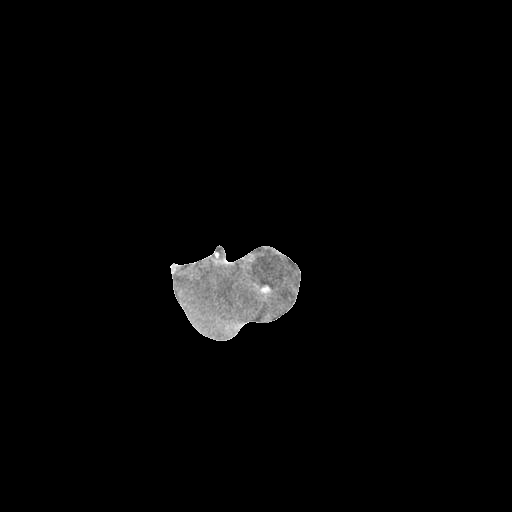

Supplement: S1 Fig — (ZIP) [file pone.0295536.s008.zip › S8_Fig/Segmentation result of AMBBEM with three FCNs in test set 2/AMBBEM/Label_10.png]

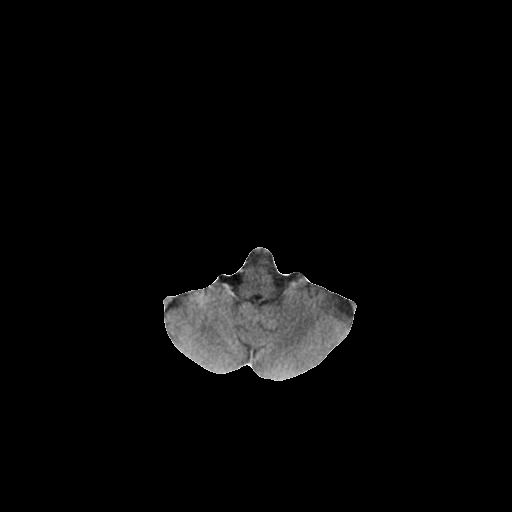

Supplement: S1 Fig — (ZIP) [file pone.0295536.s008.zip › S8_Fig/Segmentation result of AMBBEM with three FCNs in test set 2/AMBBEM/Label_100.png]

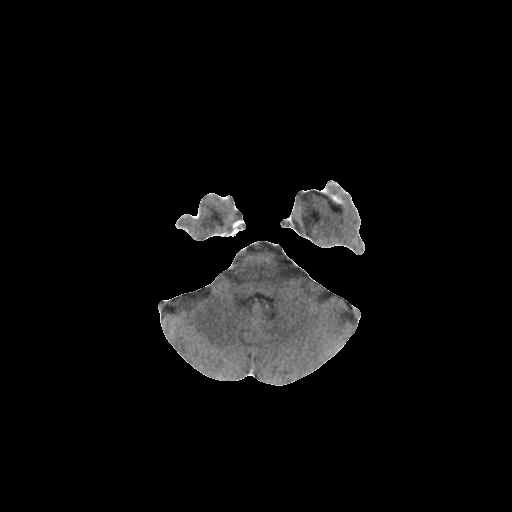

Supplement: S1 Fig — (ZIP) [file pone.0295536.s008.zip › S8_Fig/Segmentation result of AMBBEM with three FCNs in test set 2/AMBBEM/Label_101.png]

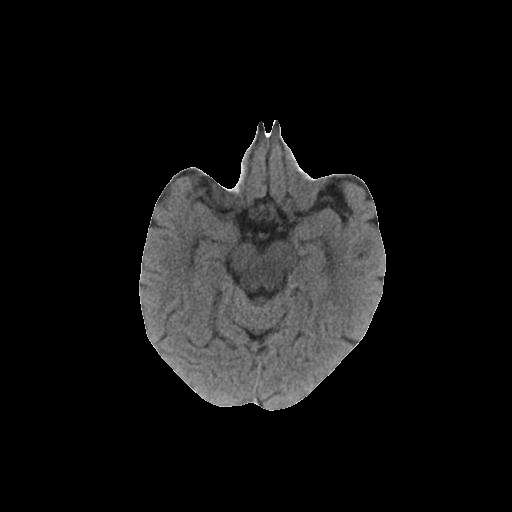

Supplement: S1 Fig — (ZIP) [file pone.0295536.s008.zip › S8_Fig/Segmentation result of AMBBEM with three FCNs in test set 2/AMBBEM/Label_102.png]

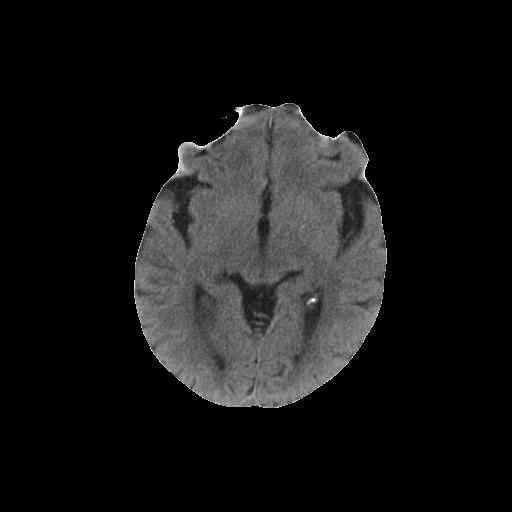

Supplement: S1 Fig — (ZIP) [file pone.0295536.s008.zip › S8_Fig/Segmentation result of AMBBEM with three FCNs in test set 2/AMBBEM/Label_103.png]

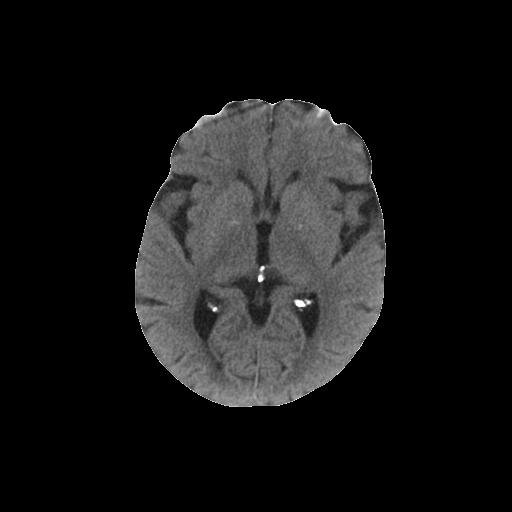

Supplement: S1 Fig — (ZIP) [file pone.0295536.s008.zip › S8_Fig/Segmentation result of AMBBEM with three FCNs in test set 2/AMBBEM/Label_104.png]

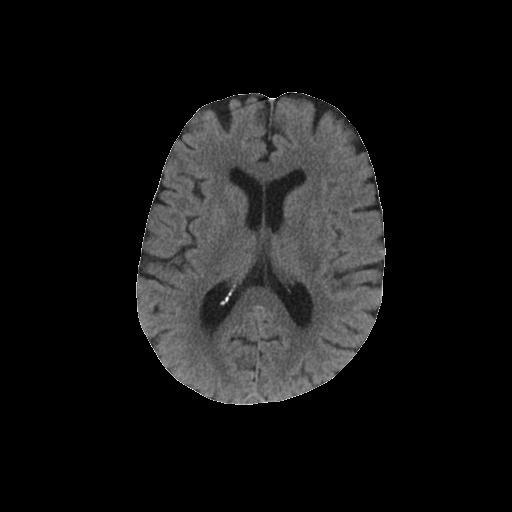

Supplement: S1 Fig — (ZIP) [file pone.0295536.s008.zip › S8_Fig/Segmentation result of AMBBEM with three FCNs in test set 2/AMBBEM/Label_105.png]

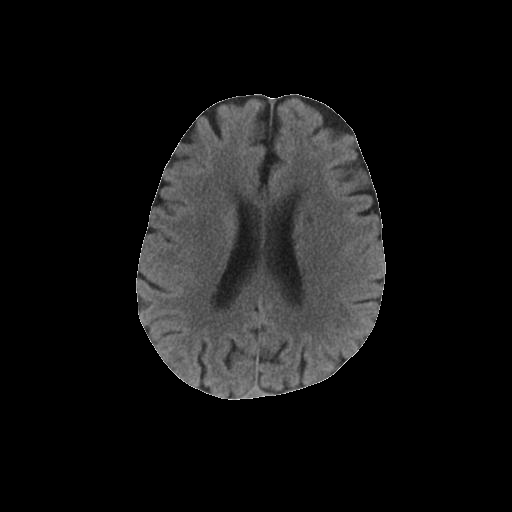

Supplement: S1 Fig — (ZIP) [file pone.0295536.s008.zip › S8_Fig/Segmentation result of AMBBEM with three FCNs in test set 2/AMBBEM/Label_106.png]

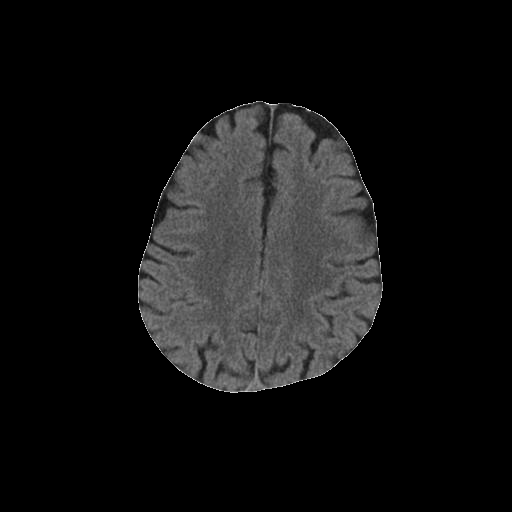

Supplement: S1 Fig — (ZIP) [file pone.0295536.s008.zip › S8_Fig/Segmentation result of AMBBEM with three FCNs in test set 2/AMBBEM/Label_107.png]

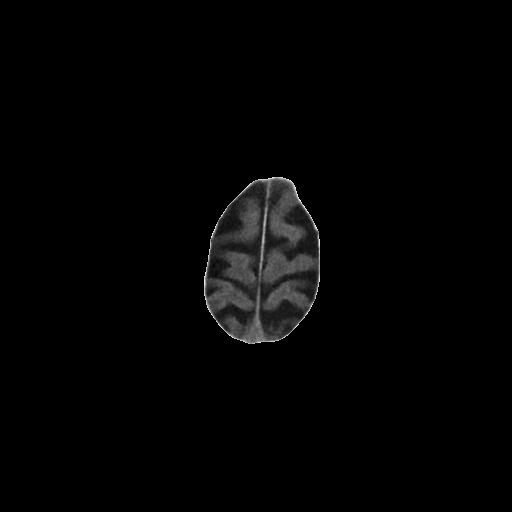

Supplement: S1 Fig — (ZIP) [file pone.0295536.s008.zip › S8_Fig/Segmentation result of AMBBEM with three FCNs in test set 2/AMBBEM/Label_108.png]

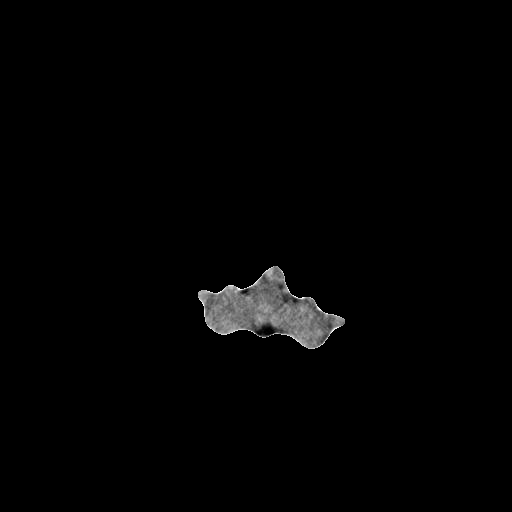

Supplement: S1 Fig — (ZIP) [file pone.0295536.s008.zip › S8_Fig/Segmentation result of AMBBEM with three FCNs in test set 2/AMBBEM/Label_109.png]

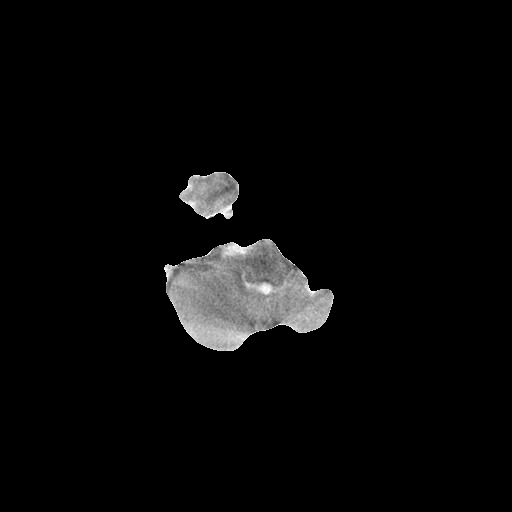

Supplement: S1 Fig — (ZIP) [file pone.0295536.s008.zip › S8_Fig/Segmentation result of AMBBEM with three FCNs in test set 2/AMBBEM/Label_11.png]

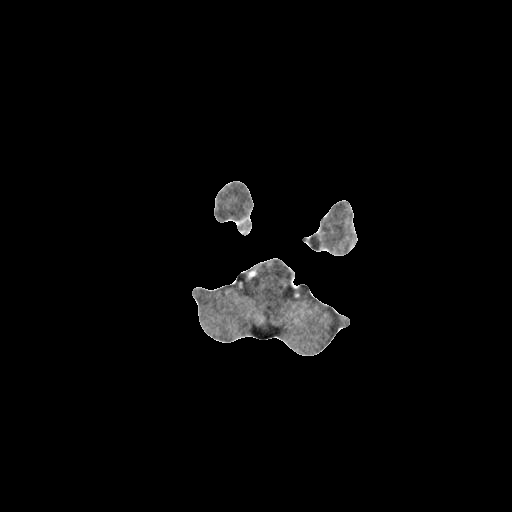

Supplement: S1 Fig — (ZIP) [file pone.0295536.s008.zip › S8_Fig/Segmentation result of AMBBEM with three FCNs in test set 2/AMBBEM/Label_110.png]

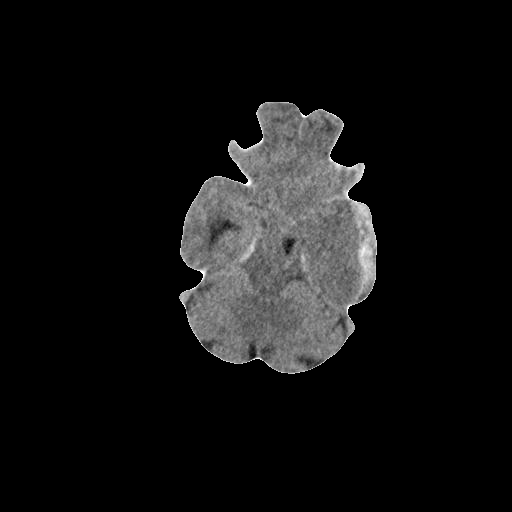

Supplement: S1 Fig — (ZIP) [file pone.0295536.s008.zip › S8_Fig/Segmentation result of AMBBEM with three FCNs in test set 2/AMBBEM/Label_111.png]

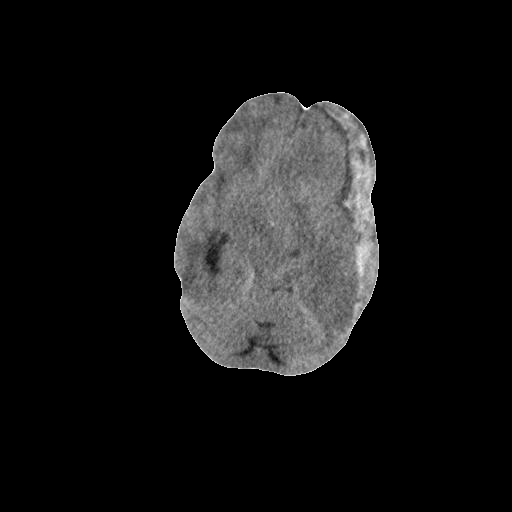

Supplement: S1 Fig — (ZIP) [file pone.0295536.s008.zip › S8_Fig/Segmentation result of AMBBEM with three FCNs in test set 2/AMBBEM/Label_112.png]

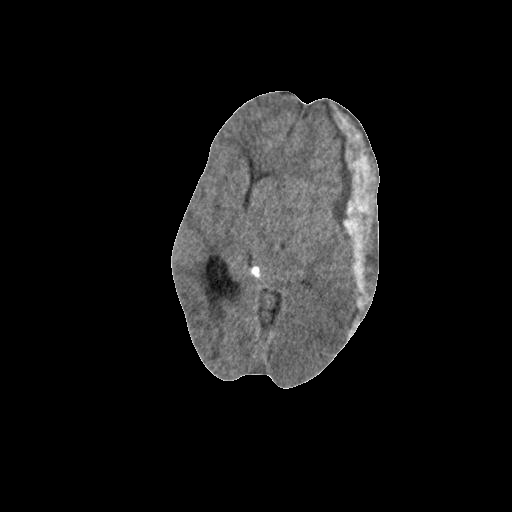

Supplement: S1 Fig — (ZIP) [file pone.0295536.s008.zip › S8_Fig/Segmentation result of AMBBEM with three FCNs in test set 2/AMBBEM/Label_113.png]

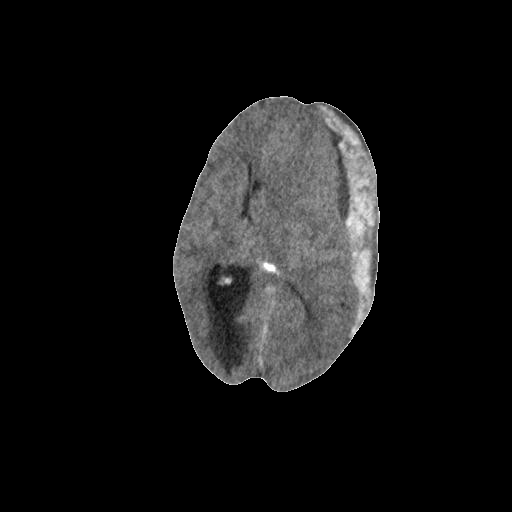

Supplement: S1 Fig — (ZIP) [file pone.0295536.s008.zip › S8_Fig/Segmentation result of AMBBEM with three FCNs in test set 2/AMBBEM/Label_114.png]

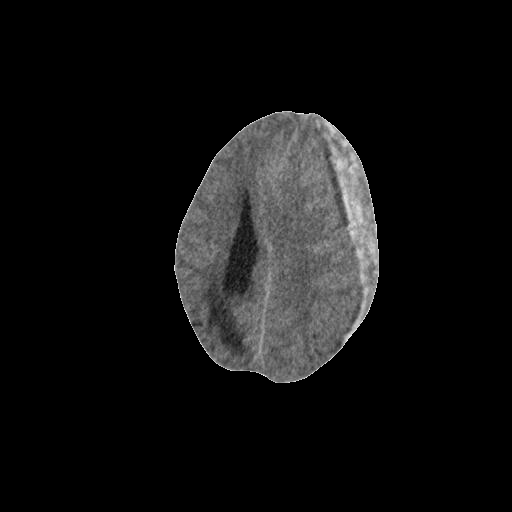

Supplement: S1 Fig — (ZIP) [file pone.0295536.s008.zip › S8_Fig/Segmentation result of AMBBEM with three FCNs in test set 2/AMBBEM/Label_115.png]

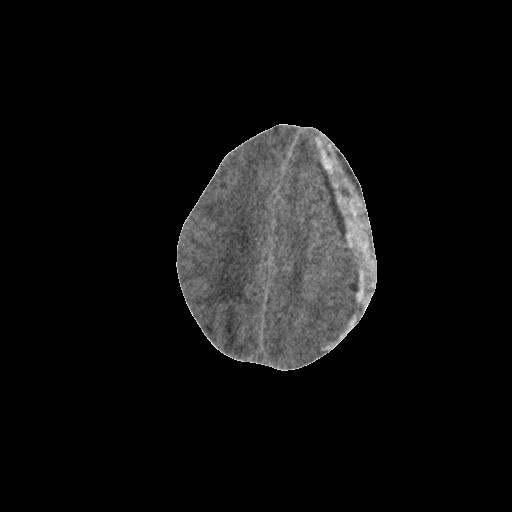

Supplement: S1 Fig — (ZIP) [file pone.0295536.s008.zip › S8_Fig/Segmentation result of AMBBEM with three FCNs in test set 2/AMBBEM/Label_116.png]

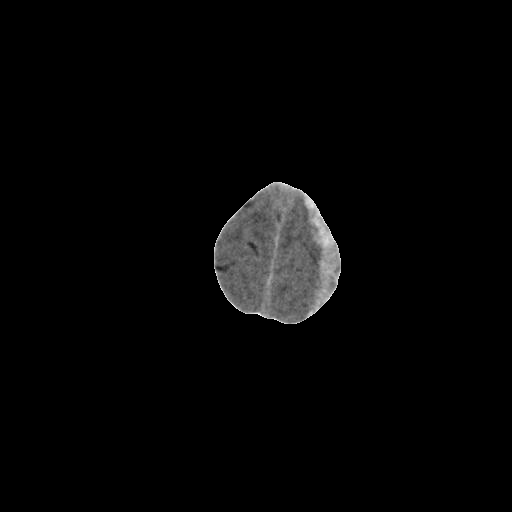

Supplement: S1 Fig — (ZIP) [file pone.0295536.s008.zip › S8_Fig/Segmentation result of AMBBEM with three FCNs in test set 2/AMBBEM/Label_117.png]

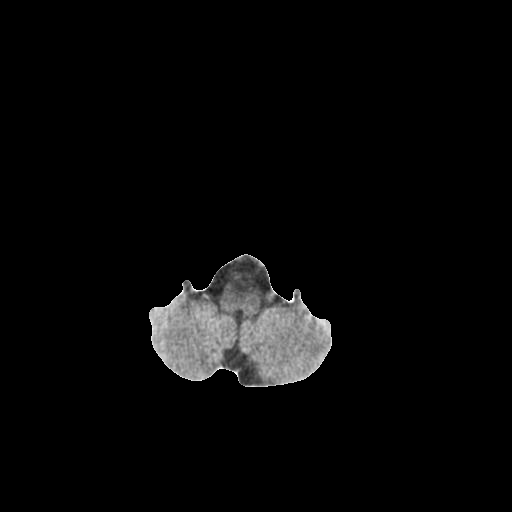

Supplement: S1 Fig — (ZIP) [file pone.0295536.s008.zip › S8_Fig/Segmentation result of AMBBEM with three FCNs in test set 2/AMBBEM/Label_118.png]

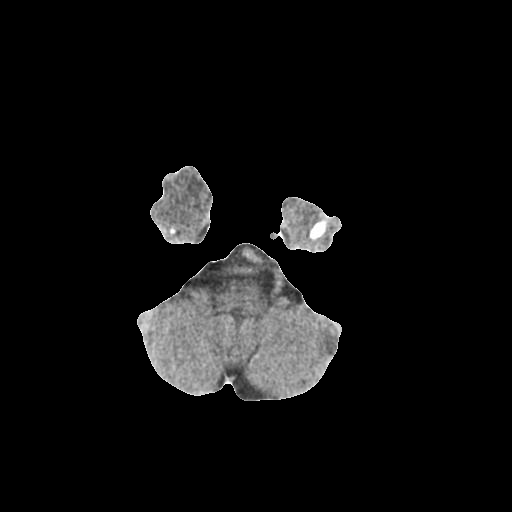

Supplement: S1 Fig — (ZIP) [file pone.0295536.s008.zip › S8_Fig/Segmentation result of AMBBEM with three FCNs in test set 2/AMBBEM/Label_119.png]

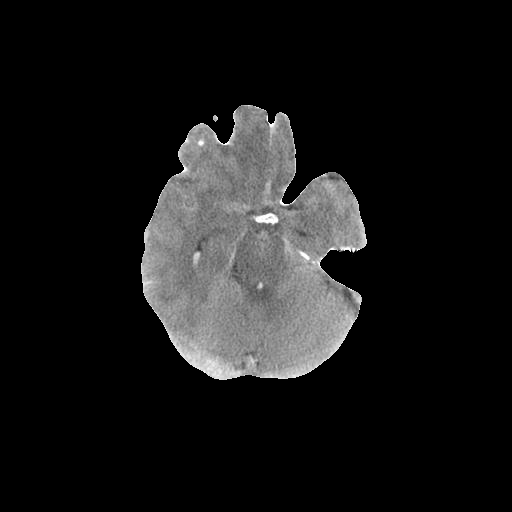

Supplement: S1 Fig — (ZIP) [file pone.0295536.s008.zip › S8_Fig/Segmentation result of AMBBEM with three FCNs in test set 2/AMBBEM/Label_12.png]

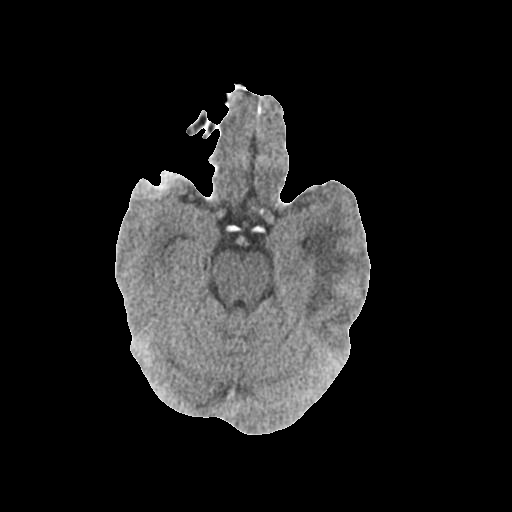

Supplement: S1 Fig — (ZIP) [file pone.0295536.s008.zip › S8_Fig/Segmentation result of AMBBEM with three FCNs in test set 2/AMBBEM/Label_120.png]

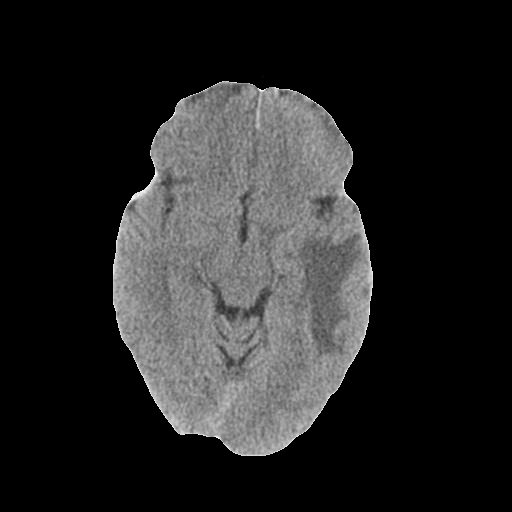

Supplement: S1 Fig — (ZIP) [file pone.0295536.s008.zip › S8_Fig/Segmentation result of AMBBEM with three FCNs in test set 2/AMBBEM/Label_121.png]

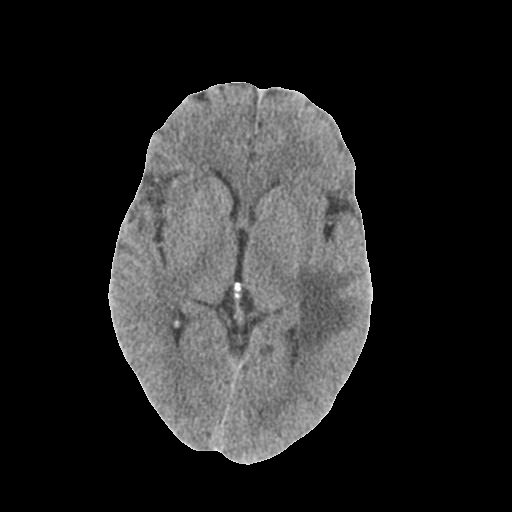

Supplement: S1 Fig — (ZIP) [file pone.0295536.s008.zip › S8_Fig/Segmentation result of AMBBEM with three FCNs in test set 2/AMBBEM/Label_122.png]

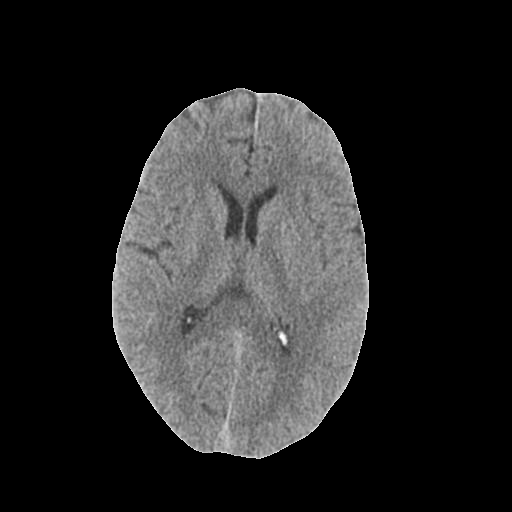

Supplement: S1 Fig — (ZIP) [file pone.0295536.s008.zip › S8_Fig/Segmentation result of AMBBEM with three FCNs in test set 2/AMBBEM/Label_123.png]

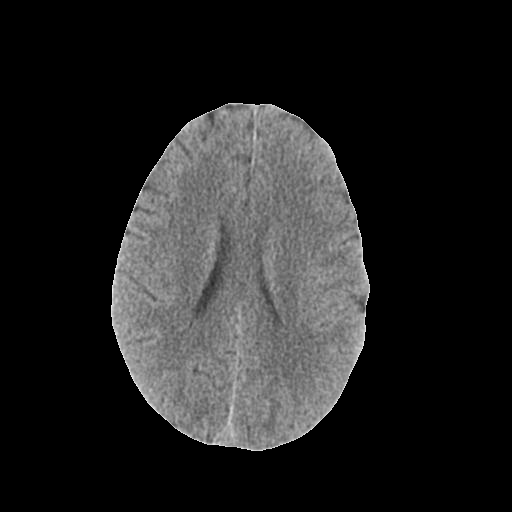

Supplement: S1 Fig — (ZIP) [file pone.0295536.s008.zip › S8_Fig/Segmentation result of AMBBEM with three FCNs in test set 2/AMBBEM/Label_124.png]

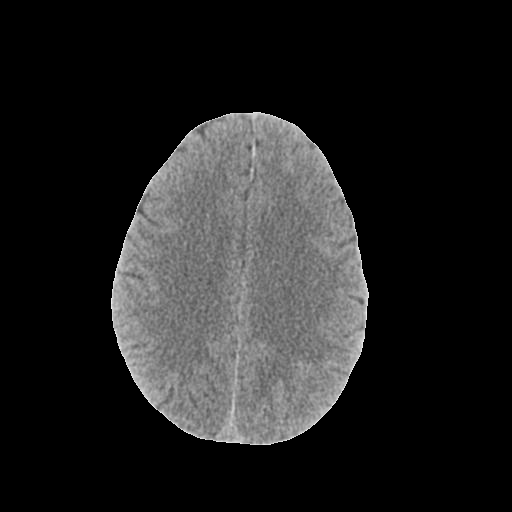

Supplement: S1 Fig — (ZIP) [file pone.0295536.s008.zip › S8_Fig/Segmentation result of AMBBEM with three FCNs in test set 2/AMBBEM/Label_125.png]

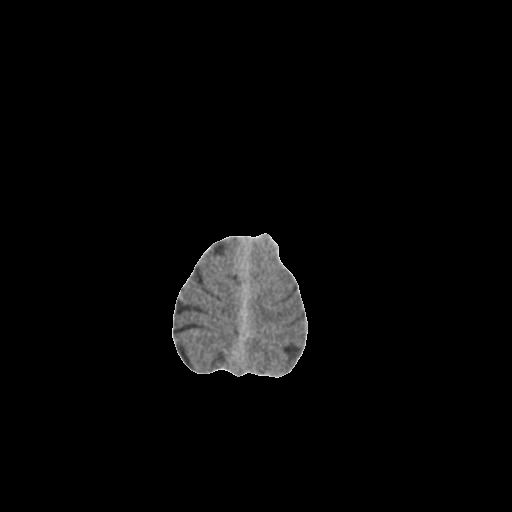

Supplement: S1 Fig — (ZIP) [file pone.0295536.s008.zip › S8_Fig/Segmentation result of AMBBEM with three FCNs in test set 2/AMBBEM/Label_126.png]

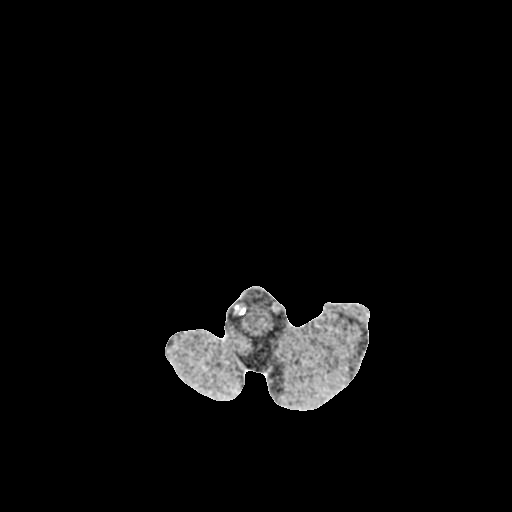

Supplement: S1 Fig — (ZIP) [file pone.0295536.s008.zip › S8_Fig/Segmentation result of AMBBEM with three FCNs in test set 2/AMBBEM/Label_127.png]

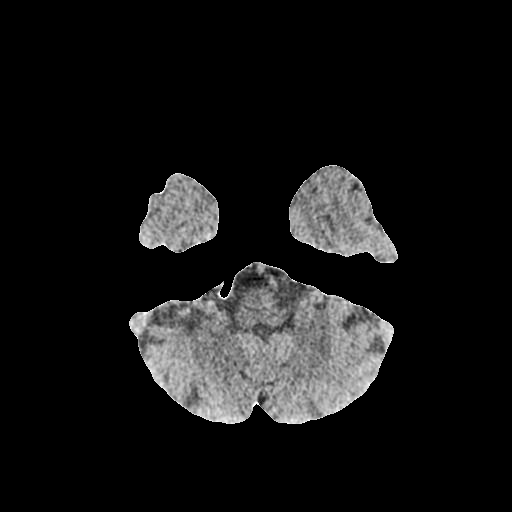

Supplement: S1 Fig — (ZIP) [file pone.0295536.s008.zip › S8_Fig/Segmentation result of AMBBEM with three FCNs in test set 2/AMBBEM/Label_128.png]

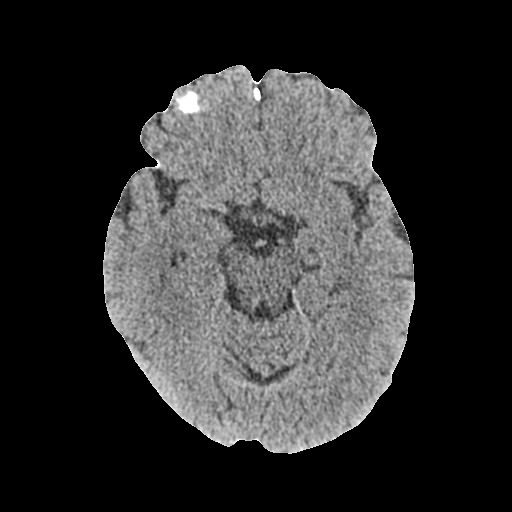

Supplement: S1 Fig — (ZIP) [file pone.0295536.s008.zip › S8_Fig/Segmentation result of AMBBEM with three FCNs in test set 2/AMBBEM/Label_129.png]

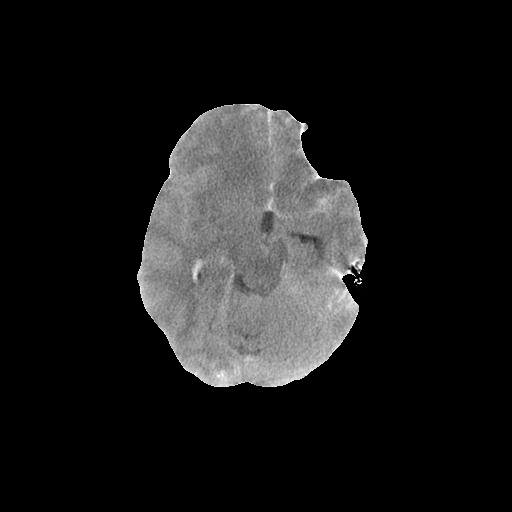

Supplement: S1 Fig — (ZIP) [file pone.0295536.s008.zip › S8_Fig/Segmentation result of AMBBEM with three FCNs in test set 2/AMBBEM/Label_13.png]

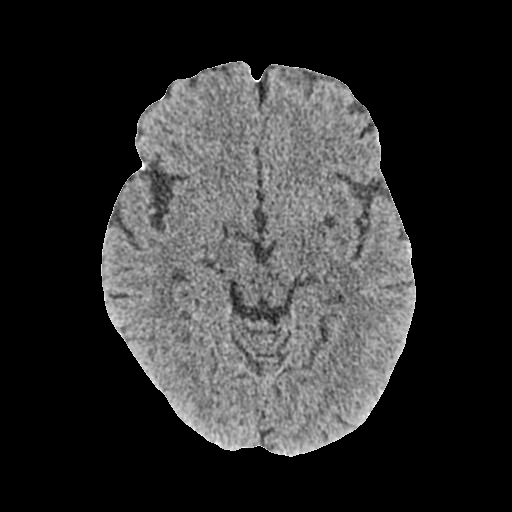

Supplement: S1 Fig — (ZIP) [file pone.0295536.s008.zip › S8_Fig/Segmentation result of AMBBEM with three FCNs in test set 2/AMBBEM/Label_130.png]

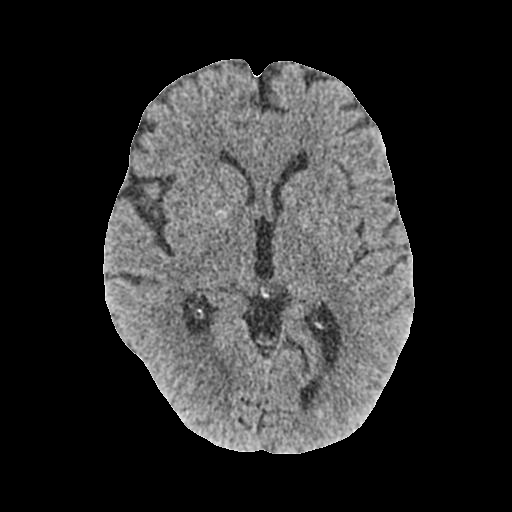

Supplement: S1 Fig — (ZIP) [file pone.0295536.s008.zip › S8_Fig/Segmentation result of AMBBEM with three FCNs in test set 2/AMBBEM/Label_131.png]

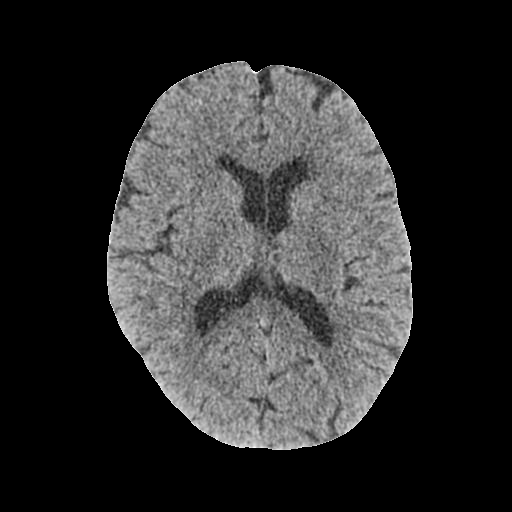

Supplement: S1 Fig — (ZIP) [file pone.0295536.s008.zip › S8_Fig/Segmentation result of AMBBEM with three FCNs in test set 2/AMBBEM/Label_132.png]

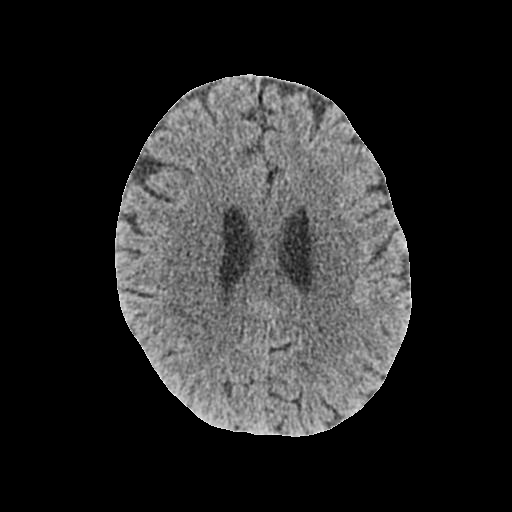

Supplement: S1 Fig — (ZIP) [file pone.0295536.s008.zip › S8_Fig/Segmentation result of AMBBEM with three FCNs in test set 2/AMBBEM/Label_133.png]

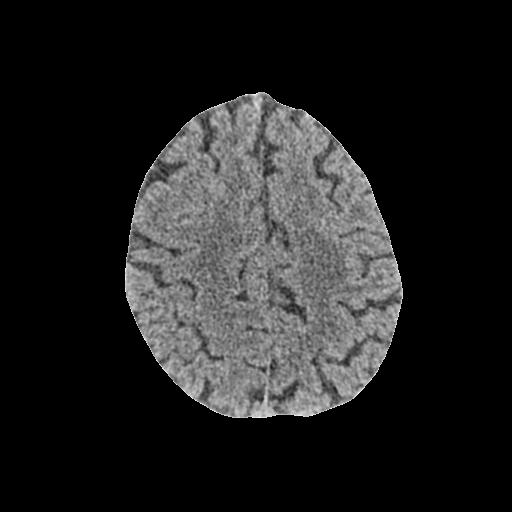

Supplement: S1 Fig — (ZIP) [file pone.0295536.s008.zip › S8_Fig/Segmentation result of AMBBEM with three FCNs in test set 2/AMBBEM/Label_134.png]

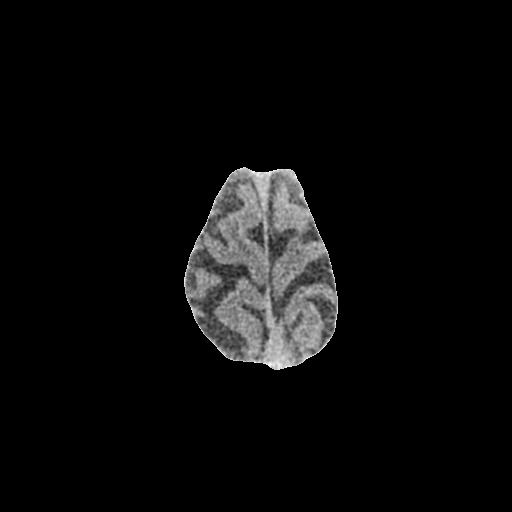

Supplement: S1 Fig — (ZIP) [file pone.0295536.s008.zip › S8_Fig/Segmentation result of AMBBEM with three FCNs in test set 2/AMBBEM/Label_135.png]

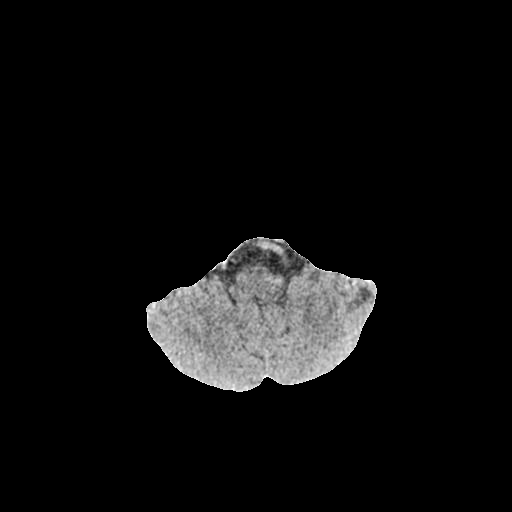

Supplement: S1 Fig — (ZIP) [file pone.0295536.s008.zip › S8_Fig/Segmentation result of AMBBEM with three FCNs in test set 2/AMBBEM/Label_136.png]

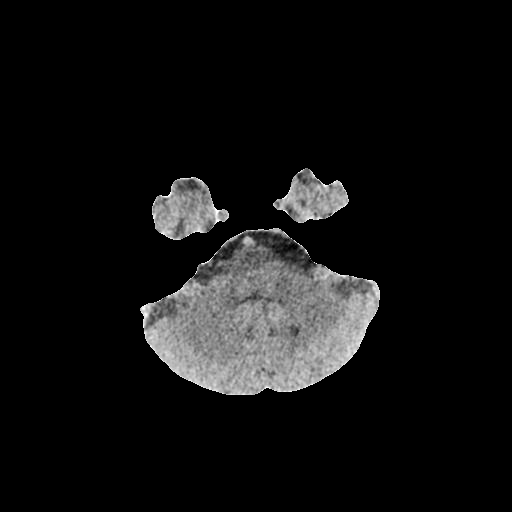

Supplement: S1 Fig — (ZIP) [file pone.0295536.s008.zip › S8_Fig/Segmentation result of AMBBEM with three FCNs in test set 2/AMBBEM/Label_137.png]

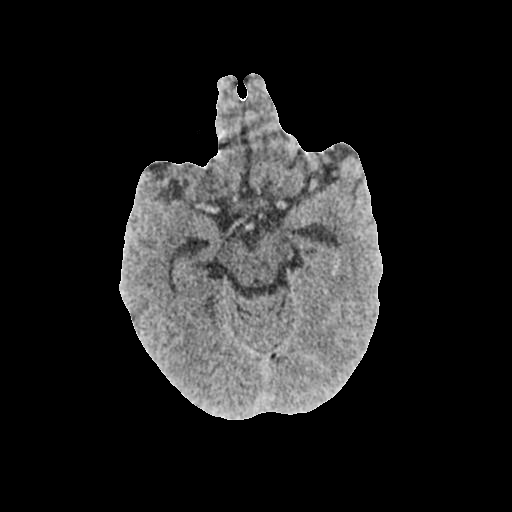

Supplement: S1 Fig — (ZIP) [file pone.0295536.s008.zip › S8_Fig/Segmentation result of AMBBEM with three FCNs in test set 2/AMBBEM/Label_138.png]

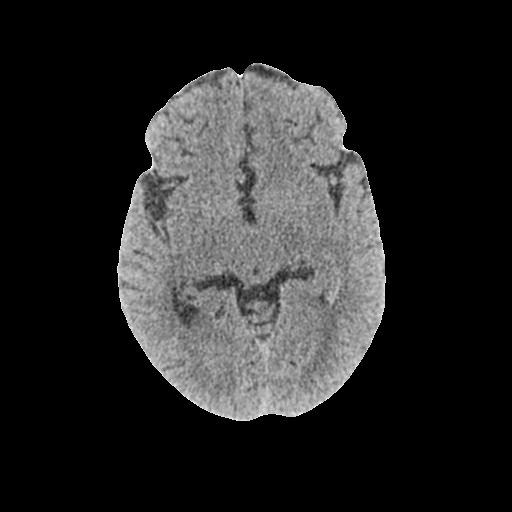

Supplement: S1 Fig — (ZIP) [file pone.0295536.s008.zip › S8_Fig/Segmentation result of AMBBEM with three FCNs in test set 2/AMBBEM/Label_139.png]

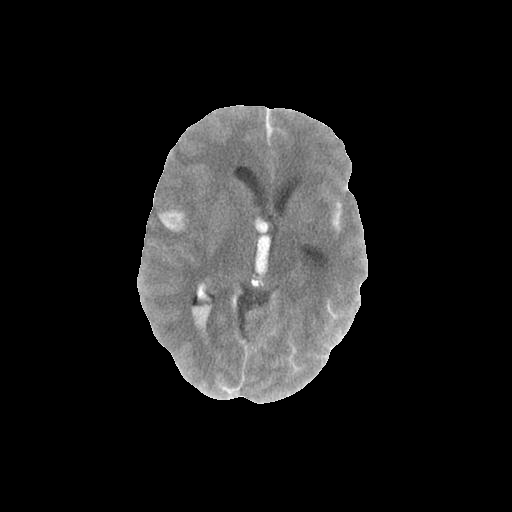

Supplement: S1 Fig — (ZIP) [file pone.0295536.s008.zip › S8_Fig/Segmentation result of AMBBEM with three FCNs in test set 2/AMBBEM/Label_14.png]

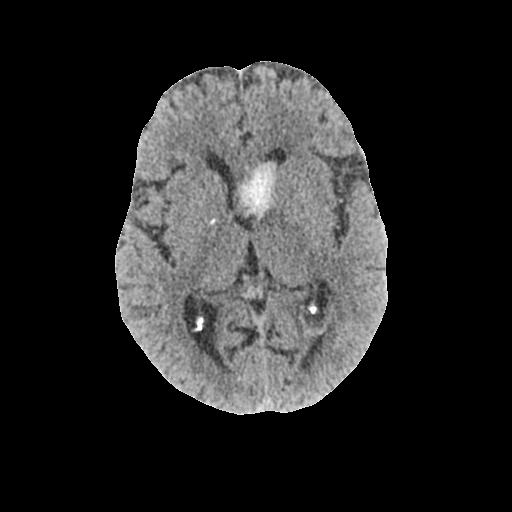

Supplement: S1 Fig — (ZIP) [file pone.0295536.s008.zip › S8_Fig/Segmentation result of AMBBEM with three FCNs in test set 2/AMBBEM/Label_140.png]

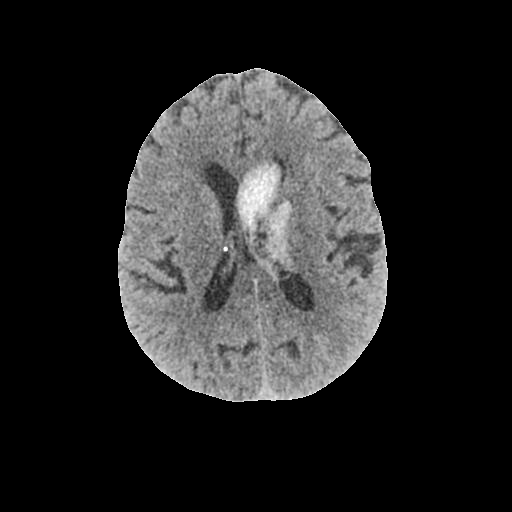

Supplement: S1 Fig — (ZIP) [file pone.0295536.s008.zip › S8_Fig/Segmentation result of AMBBEM with three FCNs in test set 2/AMBBEM/Label_141.png]

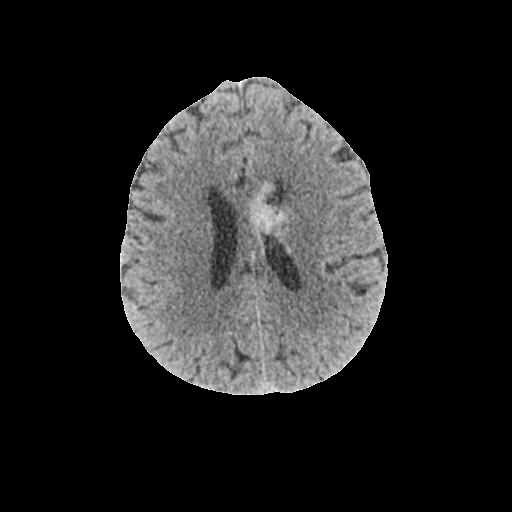

Supplement: S1 Fig — (ZIP) [file pone.0295536.s008.zip › S8_Fig/Segmentation result of AMBBEM with three FCNs in test set 2/AMBBEM/Label_142.png]

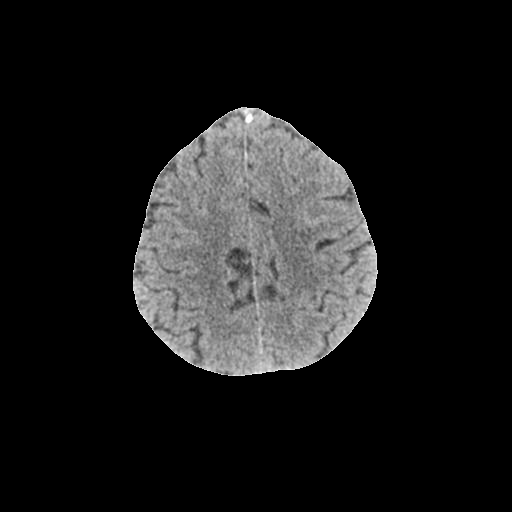

Supplement: S1 Fig — (ZIP) [file pone.0295536.s008.zip › S8_Fig/Segmentation result of AMBBEM with three FCNs in test set 2/AMBBEM/Label_143.png]

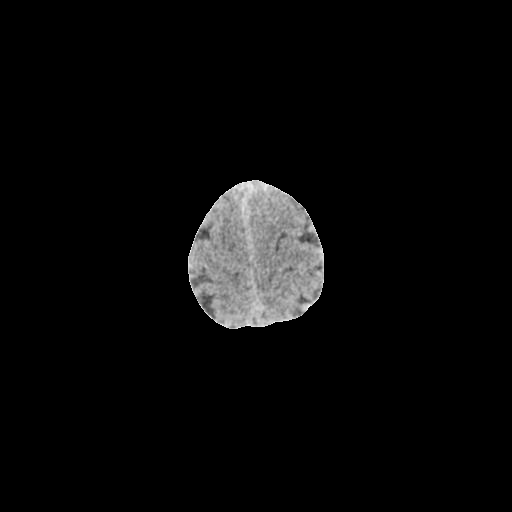

Supplement: S1 Fig — (ZIP) [file pone.0295536.s008.zip › S8_Fig/Segmentation result of AMBBEM with three FCNs in test set 2/AMBBEM/Label_144.png]

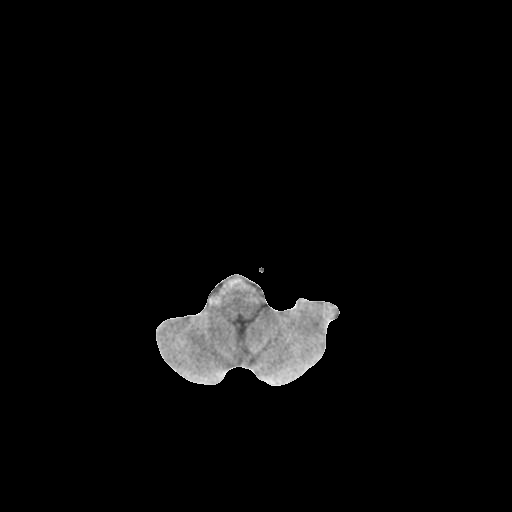

Supplement: S1 Fig — (ZIP) [file pone.0295536.s008.zip › S8_Fig/Segmentation result of AMBBEM with three FCNs in test set 2/AMBBEM/Label_145.png]

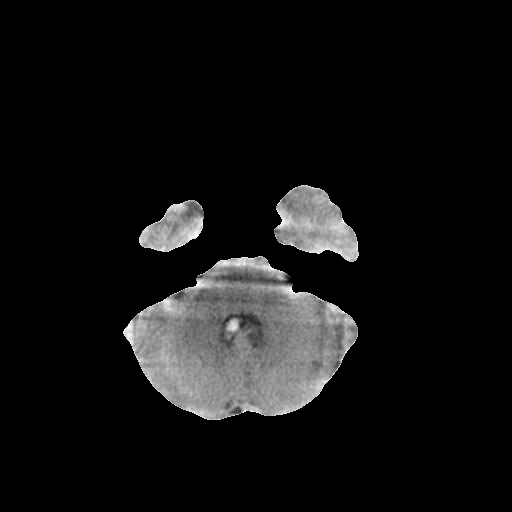

Supplement: S1 Fig — (ZIP) [file pone.0295536.s008.zip › S8_Fig/Segmentation result of AMBBEM with three FCNs in test set 2/AMBBEM/Label_146.png]

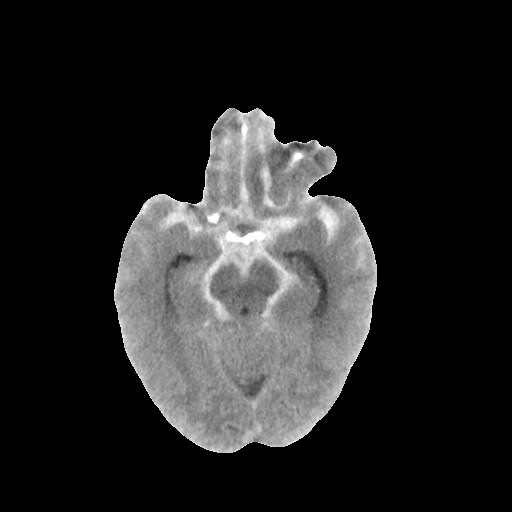

Supplement: S1 Fig — (ZIP) [file pone.0295536.s008.zip › S8_Fig/Segmentation result of AMBBEM with three FCNs in test set 2/AMBBEM/Label_147.png]

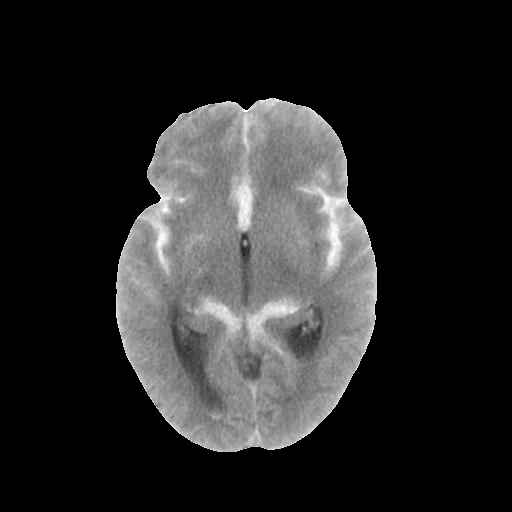

Supplement: S1 Fig — (ZIP) [file pone.0295536.s008.zip › S8_Fig/Segmentation result of AMBBEM with three FCNs in test set 2/AMBBEM/Label_148.png]

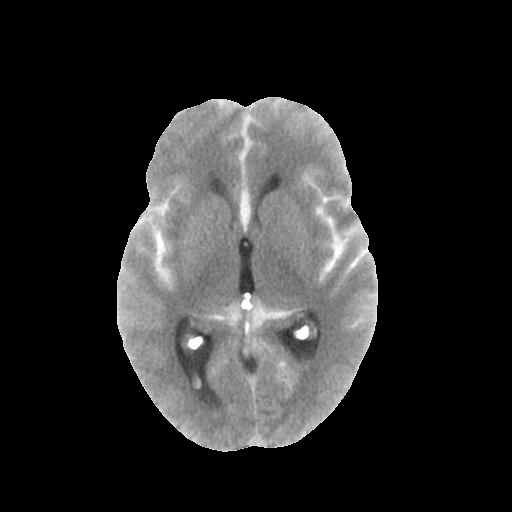

Supplement: S1 Fig — (ZIP) [file pone.0295536.s008.zip › S8_Fig/Segmentation result of AMBBEM with three FCNs in test set 2/AMBBEM/Label_149.png]

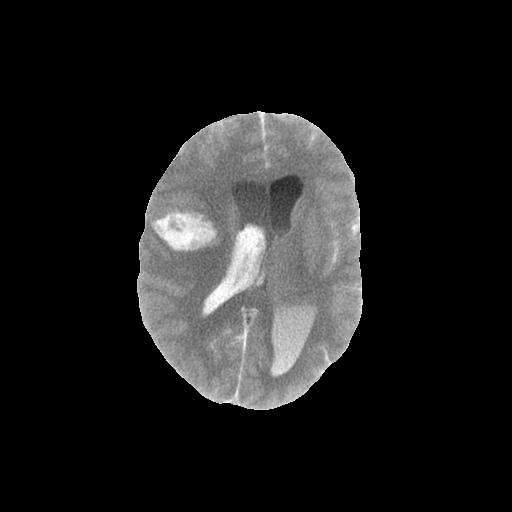

Supplement: S1 Fig — (ZIP) [file pone.0295536.s008.zip › S8_Fig/Segmentation result of AMBBEM with three FCNs in test set 2/AMBBEM/Label_15.png]

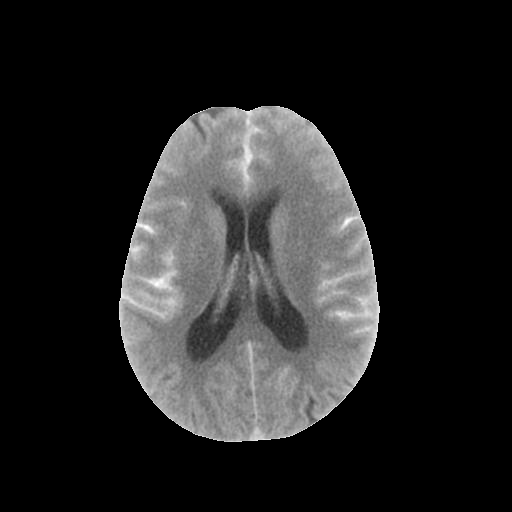

Supplement: S1 Fig — (ZIP) [file pone.0295536.s008.zip › S8_Fig/Segmentation result of AMBBEM with three FCNs in test set 2/AMBBEM/Label_150.png]

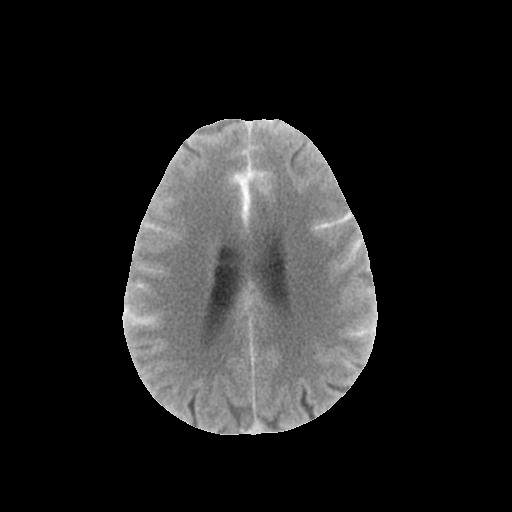

Supplement: S1 Fig — (ZIP) [file pone.0295536.s008.zip › S8_Fig/Segmentation result of AMBBEM with three FCNs in test set 2/AMBBEM/Label_151.png]

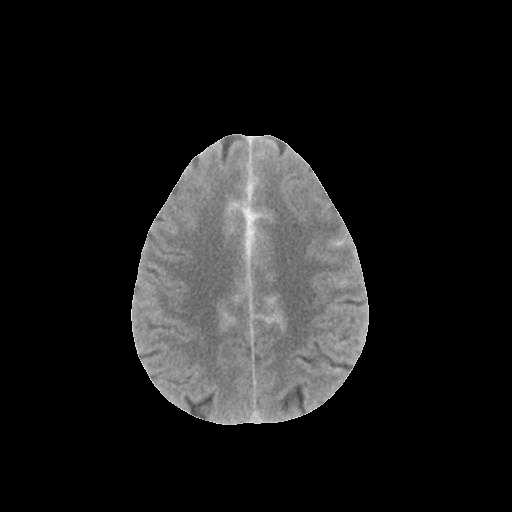

Supplement: S1 Fig — (ZIP) [file pone.0295536.s008.zip › S8_Fig/Segmentation result of AMBBEM with three FCNs in test set 2/AMBBEM/Label_152.png]

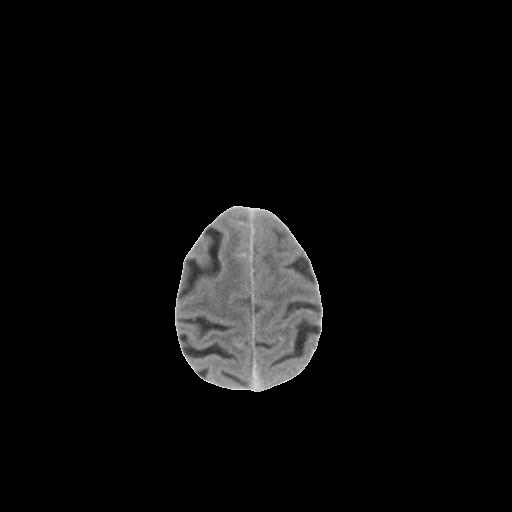

Supplement: S1 Fig — (ZIP) [file pone.0295536.s008.zip › S8_Fig/Segmentation result of AMBBEM with three FCNs in test set 2/AMBBEM/Label_153.png]

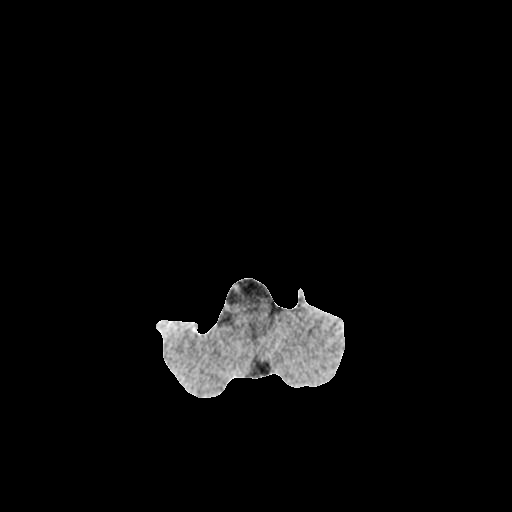

Supplement: S1 Fig — (ZIP) [file pone.0295536.s008.zip › S8_Fig/Segmentation result of AMBBEM with three FCNs in test set 2/AMBBEM/Label_154.png]

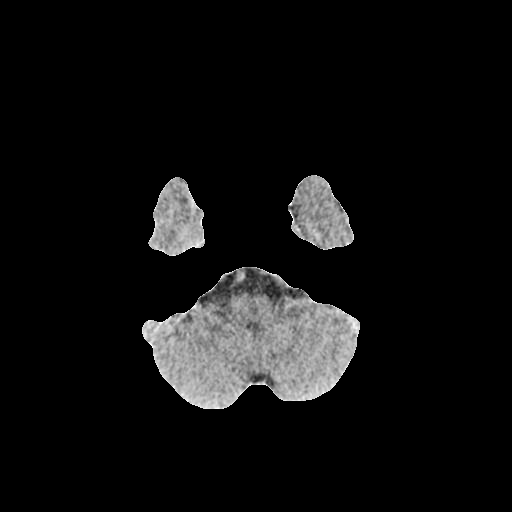

Supplement: S1 Fig — (ZIP) [file pone.0295536.s008.zip › S8_Fig/Segmentation result of AMBBEM with three FCNs in test set 2/AMBBEM/Label_155.png]

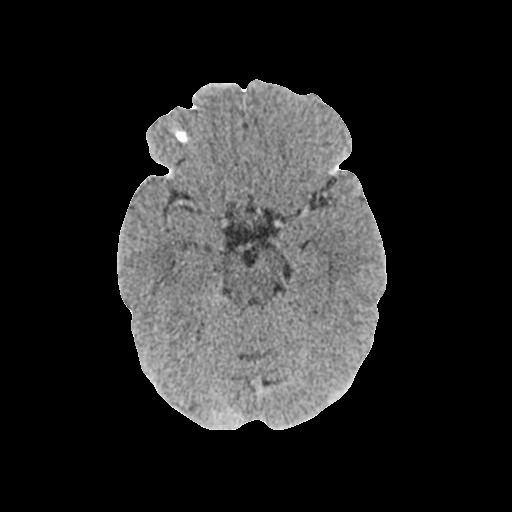

Supplement: S1 Fig — (ZIP) [file pone.0295536.s008.zip › S8_Fig/Segmentation result of AMBBEM with three FCNs in test set 2/AMBBEM/Label_156.png]

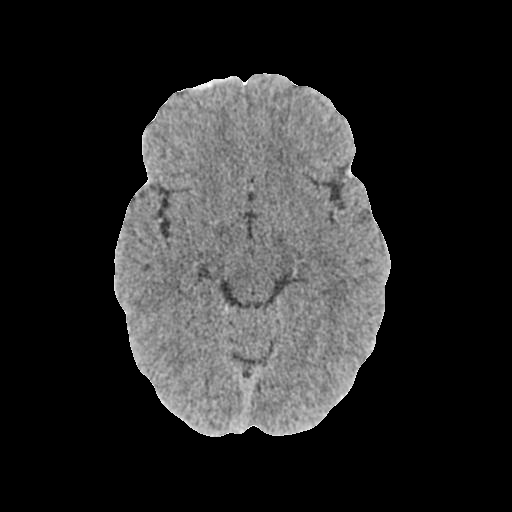

Supplement: S1 Fig — (ZIP) [file pone.0295536.s008.zip › S8_Fig/Segmentation result of AMBBEM with three FCNs in test set 2/AMBBEM/Label_157.png]

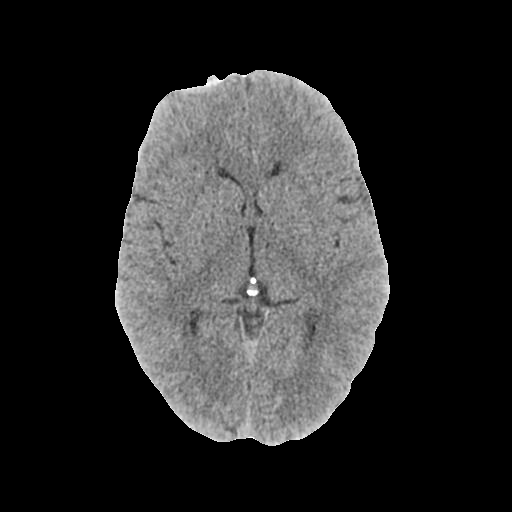

Supplement: S1 Fig — (ZIP) [file pone.0295536.s008.zip › S8_Fig/Segmentation result of AMBBEM with three FCNs in test set 2/AMBBEM/Label_158.png]

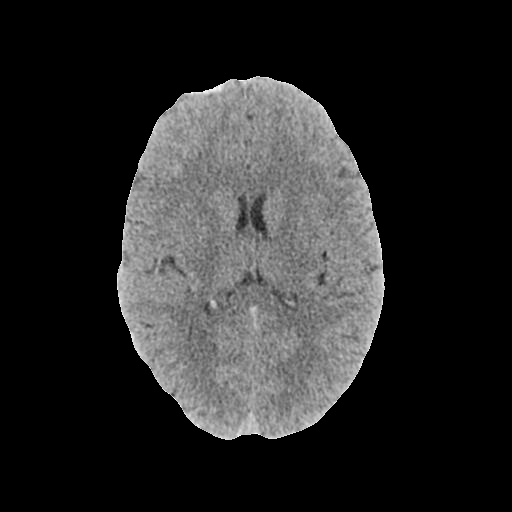

Supplement: S1 Fig — (ZIP) [file pone.0295536.s008.zip › S8_Fig/Segmentation result of AMBBEM with three FCNs in test set 2/AMBBEM/Label_159.png]

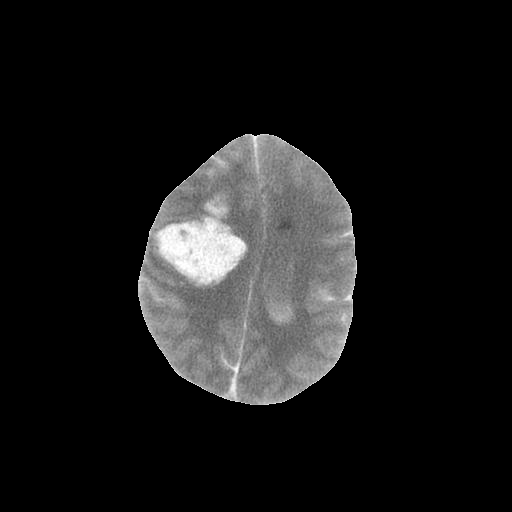

Supplement: S1 Fig — (ZIP) [file pone.0295536.s008.zip › S8_Fig/Segmentation result of AMBBEM with three FCNs in test set 2/AMBBEM/Label_16.png]

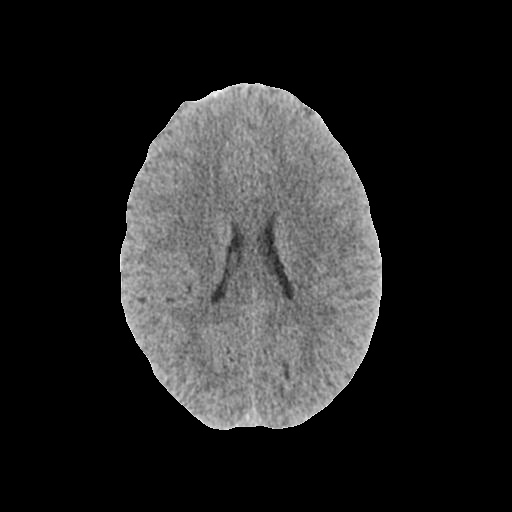

Supplement: S1 Fig — (ZIP) [file pone.0295536.s008.zip › S8_Fig/Segmentation result of AMBBEM with three FCNs in test set 2/AMBBEM/Label_160.png]

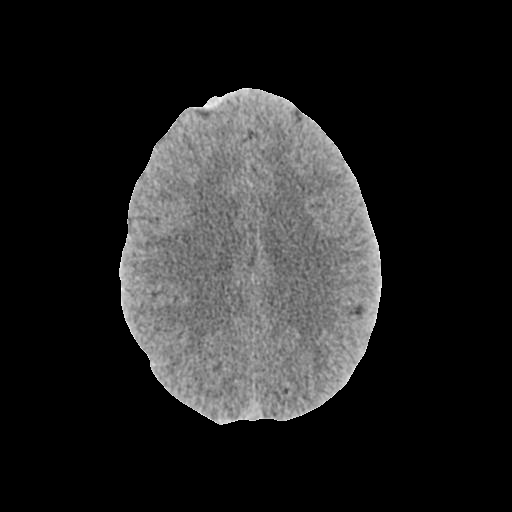

Supplement: S1 Fig — (ZIP) [file pone.0295536.s008.zip › S8_Fig/Segmentation result of AMBBEM with three FCNs in test set 2/AMBBEM/Label_161.png]

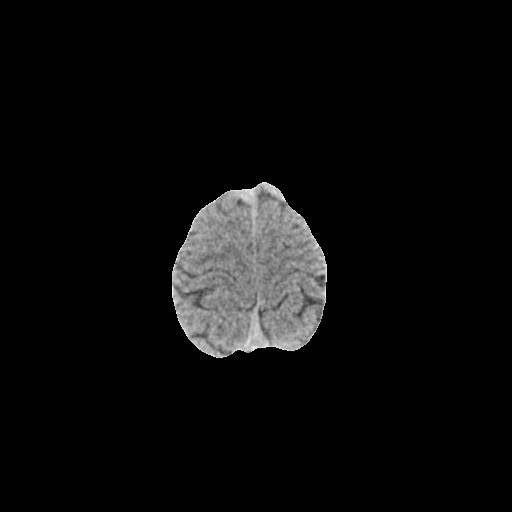

Supplement: S1 Fig — (ZIP) [file pone.0295536.s008.zip › S8_Fig/Segmentation result of AMBBEM with three FCNs in test set 2/AMBBEM/Label_162.png]

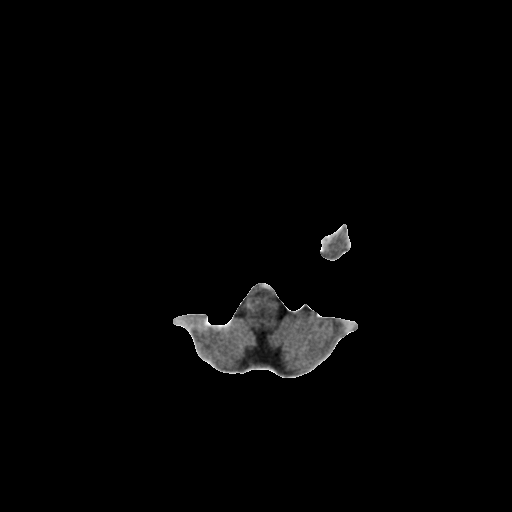

Supplement: S1 Fig — (ZIP) [file pone.0295536.s008.zip › S8_Fig/Segmentation result of AMBBEM with three FCNs in test set 2/AMBBEM/Label_163.png]

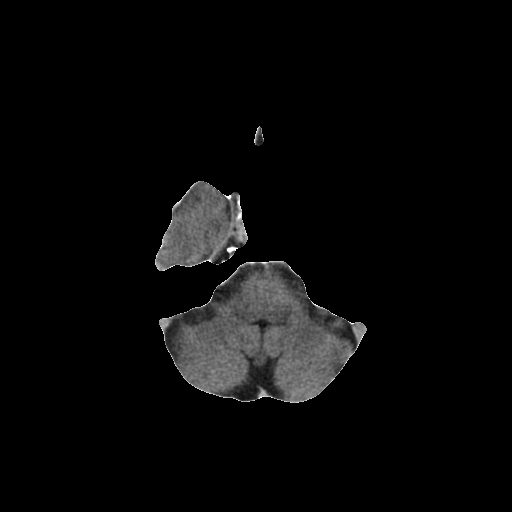

Supplement: S1 Fig — (ZIP) [file pone.0295536.s008.zip › S8_Fig/Segmentation result of AMBBEM with three FCNs in test set 2/AMBBEM/Label_164.png]
