# Supplementary figures and images for: Adaptive mask-based brain extraction method for head CT images (part 14 of 14)
Source: PLoS One. 2024 Mar 11;19(3):e0295536. doi: 10.1371/journal.pone.0295536 (PMC10927156; doi:10.1371/journal.pone.0295536)

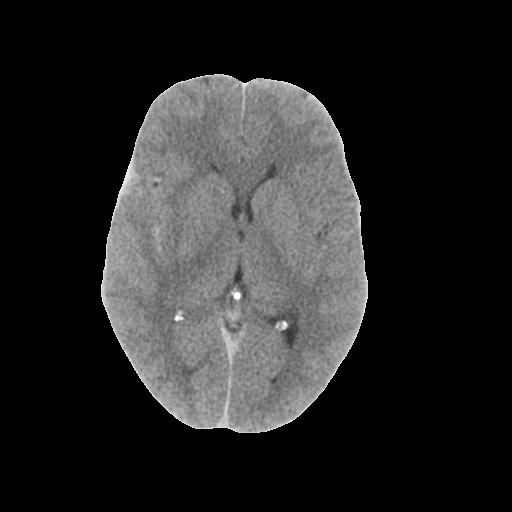

Supplement: S1 Fig — (ZIP) [file pone.0295536.s008.zip › S8_Fig/Segmentation result of AMBBEM with three FCNs in test set 2/AMBBEM/Label_77.png]

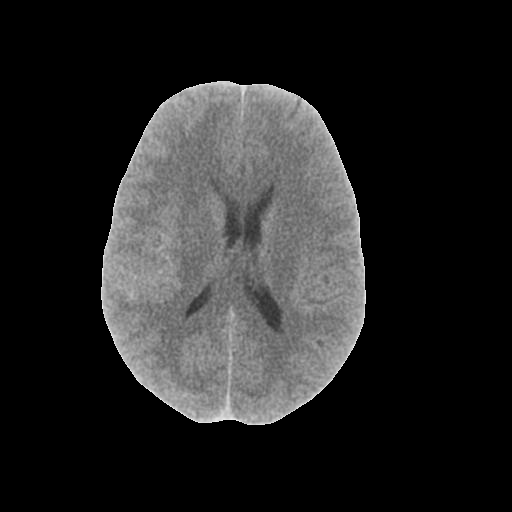

Supplement: S1 Fig — (ZIP) [file pone.0295536.s008.zip › S8_Fig/Segmentation result of AMBBEM with three FCNs in test set 2/AMBBEM/Label_78.png]

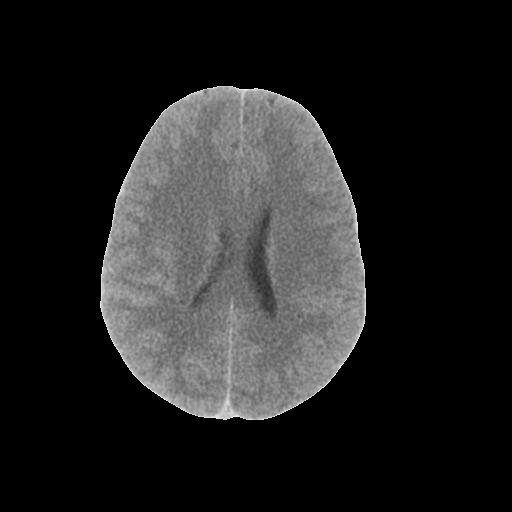

Supplement: S1 Fig — (ZIP) [file pone.0295536.s008.zip › S8_Fig/Segmentation result of AMBBEM with three FCNs in test set 2/AMBBEM/Label_79.png]

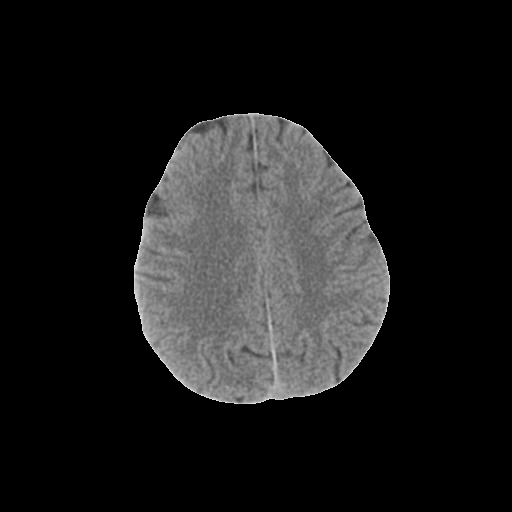

Supplement: S1 Fig — (ZIP) [file pone.0295536.s008.zip › S8_Fig/Segmentation result of AMBBEM with three FCNs in test set 2/AMBBEM/Label_8.png]

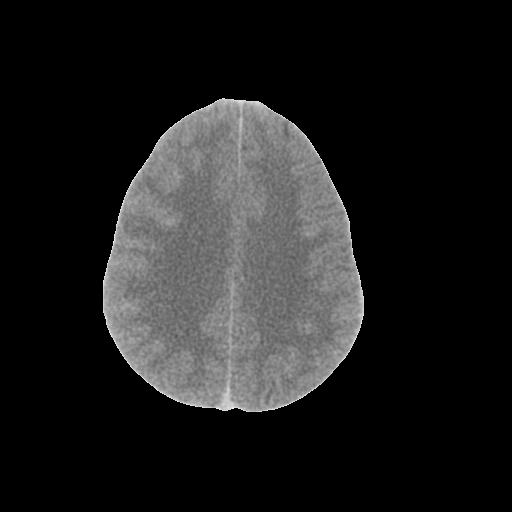

Supplement: S1 Fig — (ZIP) [file pone.0295536.s008.zip › S8_Fig/Segmentation result of AMBBEM with three FCNs in test set 2/AMBBEM/Label_80.png]

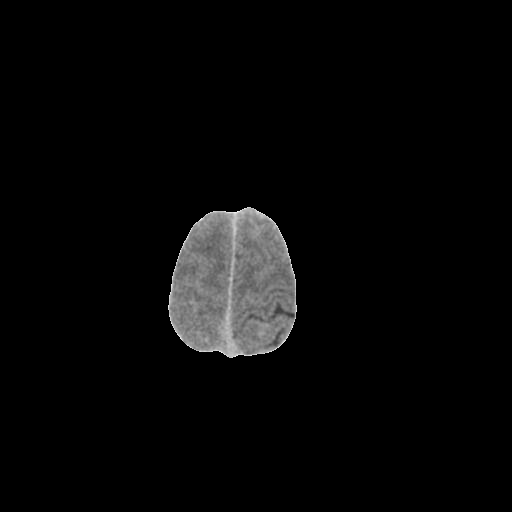

Supplement: S1 Fig — (ZIP) [file pone.0295536.s008.zip › S8_Fig/Segmentation result of AMBBEM with three FCNs in test set 2/AMBBEM/Label_81.png]

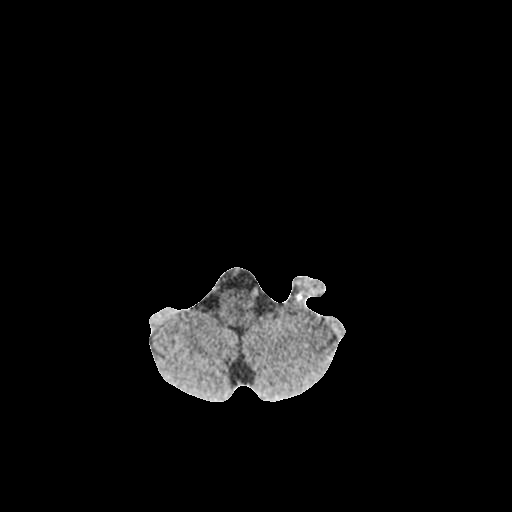

Supplement: S1 Fig — (ZIP) [file pone.0295536.s008.zip › S8_Fig/Segmentation result of AMBBEM with three FCNs in test set 2/AMBBEM/Label_82.png]

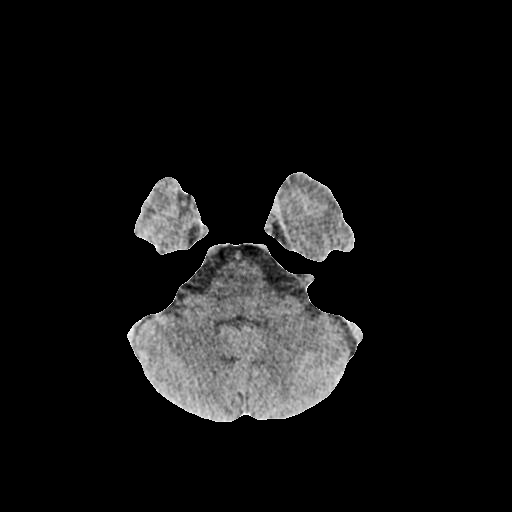

Supplement: S1 Fig — (ZIP) [file pone.0295536.s008.zip › S8_Fig/Segmentation result of AMBBEM with three FCNs in test set 2/AMBBEM/Label_83.png]

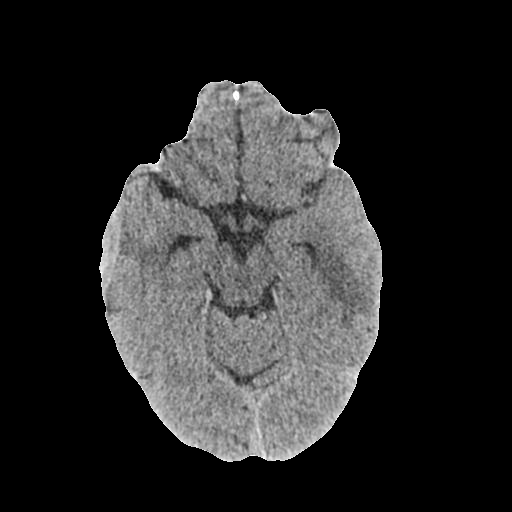

Supplement: S1 Fig — (ZIP) [file pone.0295536.s008.zip › S8_Fig/Segmentation result of AMBBEM with three FCNs in test set 2/AMBBEM/Label_84.png]

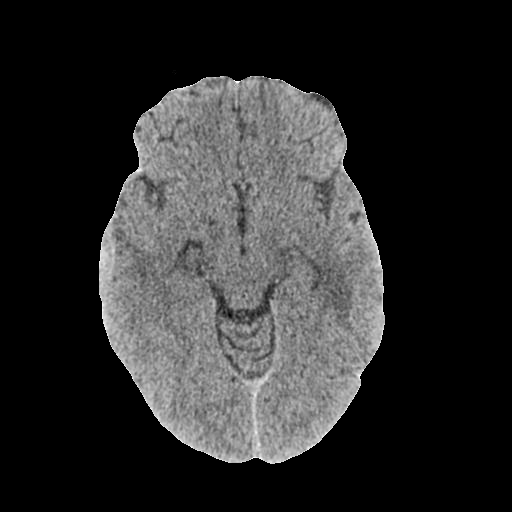

Supplement: S1 Fig — (ZIP) [file pone.0295536.s008.zip › S8_Fig/Segmentation result of AMBBEM with three FCNs in test set 2/AMBBEM/Label_85.png]

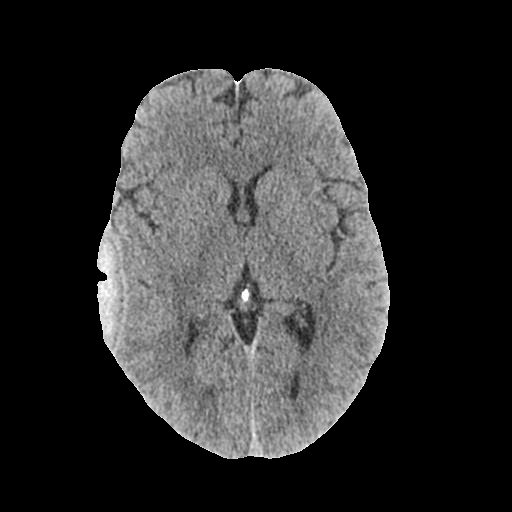

Supplement: S1 Fig — (ZIP) [file pone.0295536.s008.zip › S8_Fig/Segmentation result of AMBBEM with three FCNs in test set 2/AMBBEM/Label_86.png]

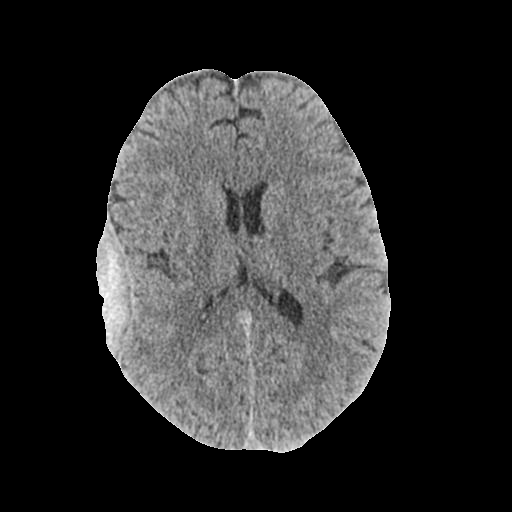

Supplement: S1 Fig — (ZIP) [file pone.0295536.s008.zip › S8_Fig/Segmentation result of AMBBEM with three FCNs in test set 2/AMBBEM/Label_87.png]

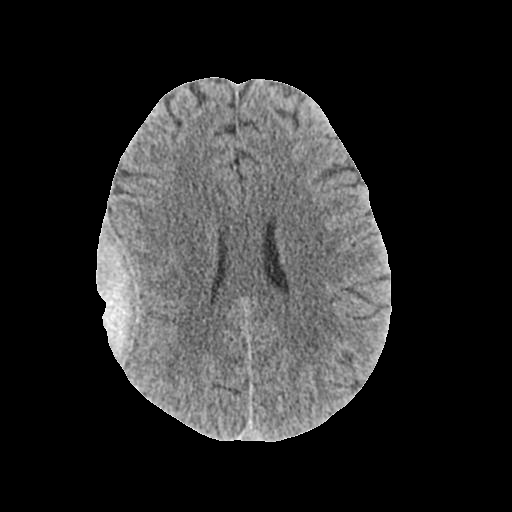

Supplement: S1 Fig — (ZIP) [file pone.0295536.s008.zip › S8_Fig/Segmentation result of AMBBEM with three FCNs in test set 2/AMBBEM/Label_88.png]

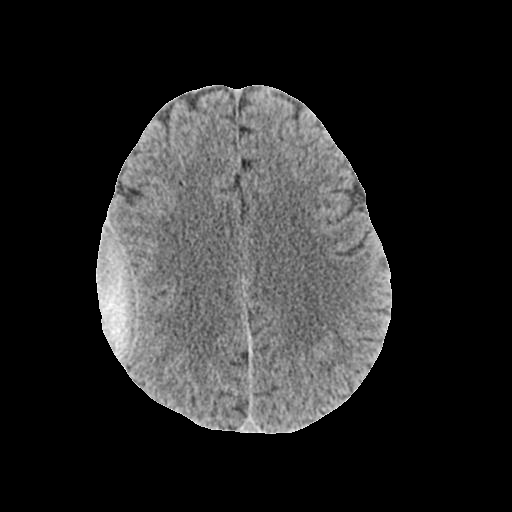

Supplement: S1 Fig — (ZIP) [file pone.0295536.s008.zip › S8_Fig/Segmentation result of AMBBEM with three FCNs in test set 2/AMBBEM/Label_89.png]

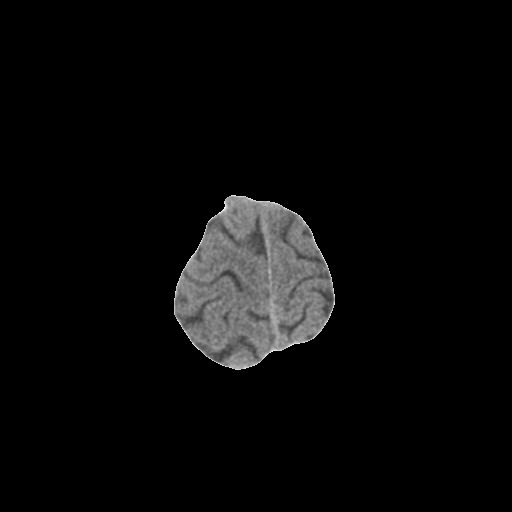

Supplement: S1 Fig — (ZIP) [file pone.0295536.s008.zip › S8_Fig/Segmentation result of AMBBEM with three FCNs in test set 2/AMBBEM/Label_9.png]

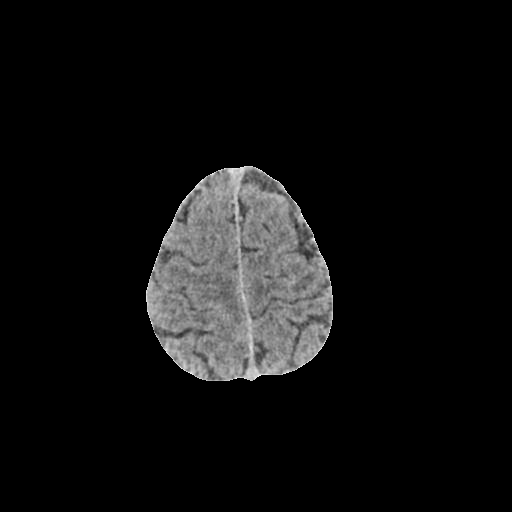

Supplement: S1 Fig — (ZIP) [file pone.0295536.s008.zip › S8_Fig/Segmentation result of AMBBEM with three FCNs in test set 2/AMBBEM/Label_90.png]

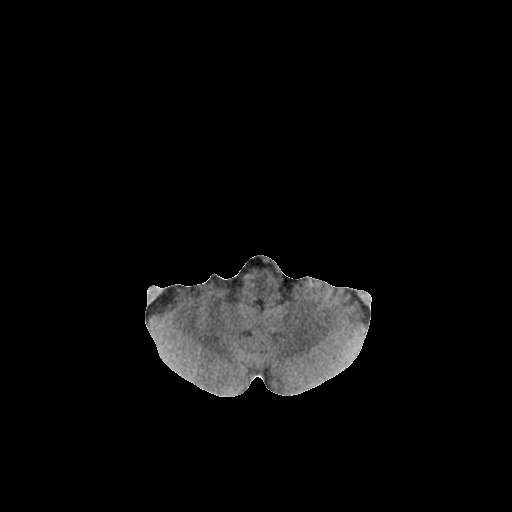

Supplement: S1 Fig — (ZIP) [file pone.0295536.s008.zip › S8_Fig/Segmentation result of AMBBEM with three FCNs in test set 2/AMBBEM/Label_91.png]

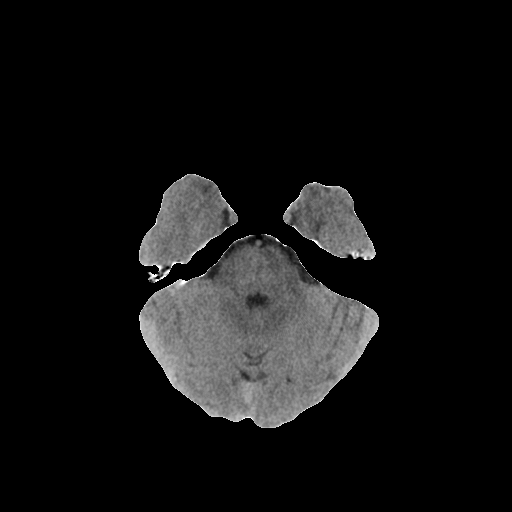

Supplement: S1 Fig — (ZIP) [file pone.0295536.s008.zip › S8_Fig/Segmentation result of AMBBEM with three FCNs in test set 2/AMBBEM/Label_92.png]

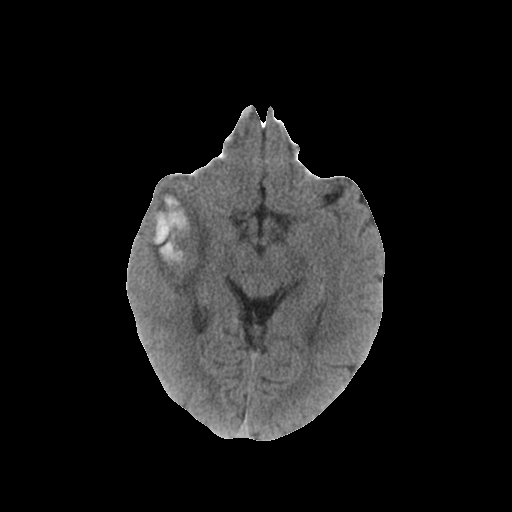

Supplement: S1 Fig — (ZIP) [file pone.0295536.s008.zip › S8_Fig/Segmentation result of AMBBEM with three FCNs in test set 2/AMBBEM/Label_93.png]

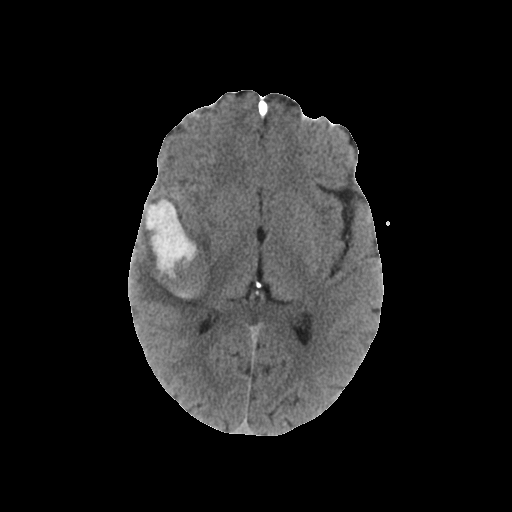

Supplement: S1 Fig — (ZIP) [file pone.0295536.s008.zip › S8_Fig/Segmentation result of AMBBEM with three FCNs in test set 2/AMBBEM/Label_94.png]

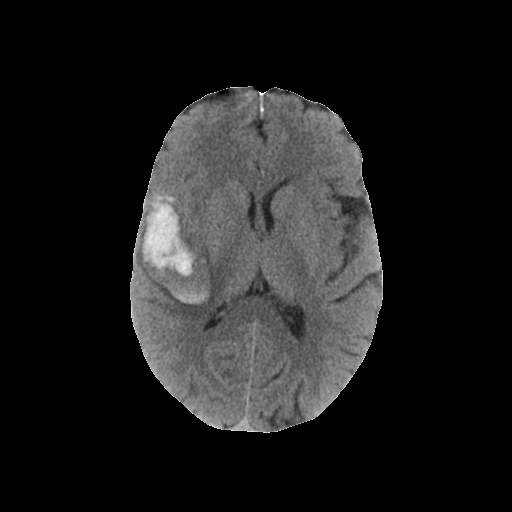

Supplement: S1 Fig — (ZIP) [file pone.0295536.s008.zip › S8_Fig/Segmentation result of AMBBEM with three FCNs in test set 2/AMBBEM/Label_95.png]

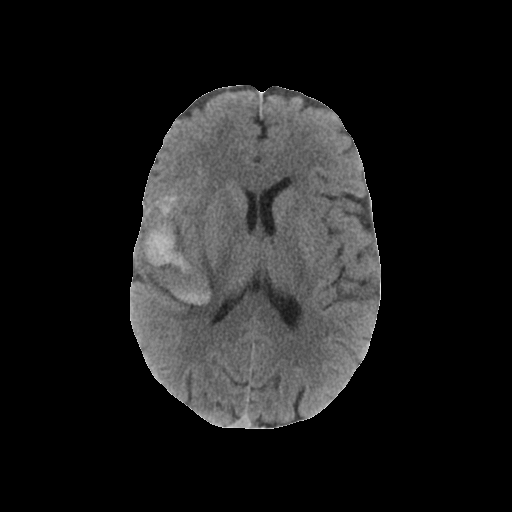

Supplement: S1 Fig — (ZIP) [file pone.0295536.s008.zip › S8_Fig/Segmentation result of AMBBEM with three FCNs in test set 2/AMBBEM/Label_96.png]

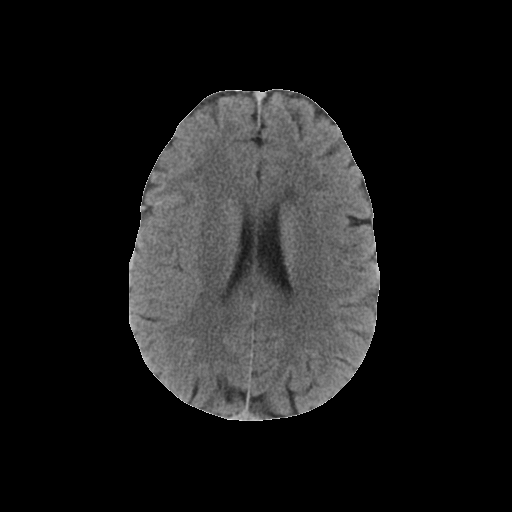

Supplement: S1 Fig — (ZIP) [file pone.0295536.s008.zip › S8_Fig/Segmentation result of AMBBEM with three FCNs in test set 2/AMBBEM/Label_97.png]

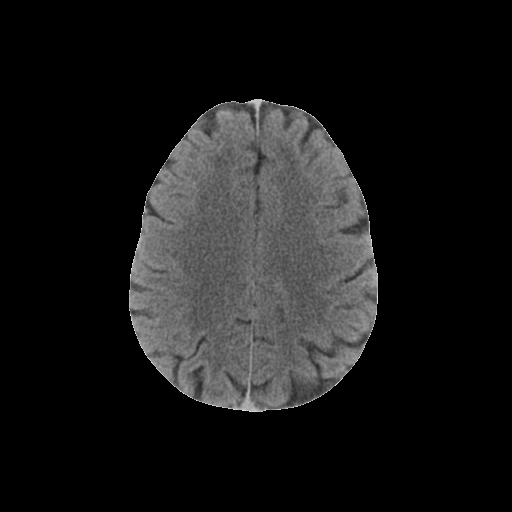

Supplement: S1 Fig — (ZIP) [file pone.0295536.s008.zip › S8_Fig/Segmentation result of AMBBEM with three FCNs in test set 2/AMBBEM/Label_98.png]

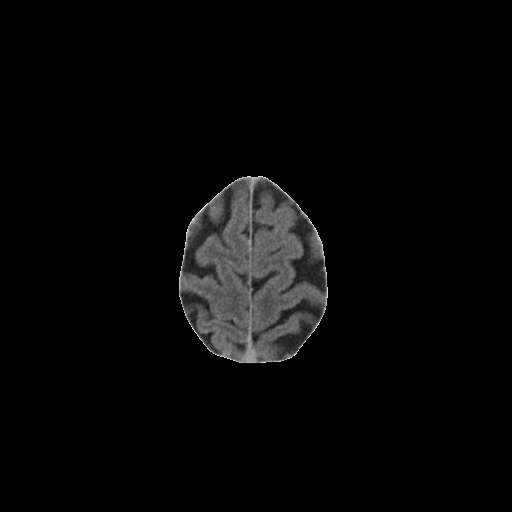

Supplement: S1 Fig — (ZIP) [file pone.0295536.s008.zip › S8_Fig/Segmentation result of AMBBEM with three FCNs in test set 2/AMBBEM/Label_99.png]

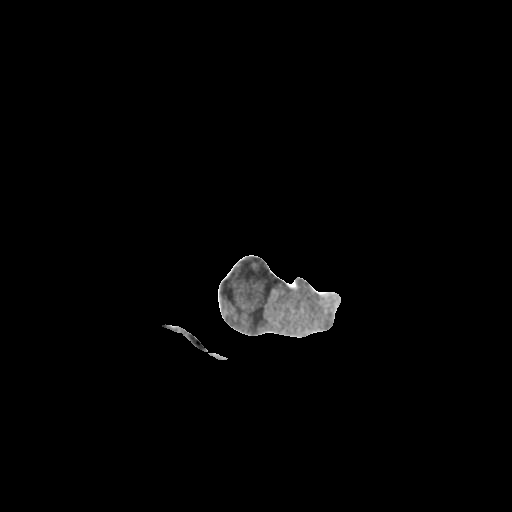

Supplement: S1 Fig — (ZIP) [file pone.0295536.s008.zip › S8_Fig/Segmentation result of AMBBEM with three FCNs in test set 2/Deeplabv3+/Label_1.png]

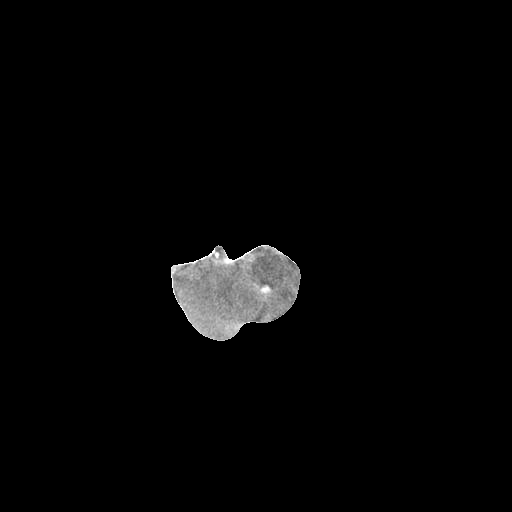

Supplement: S1 Fig — (ZIP) [file pone.0295536.s008.zip › S8_Fig/Segmentation result of AMBBEM with three FCNs in test set 2/Deeplabv3+/Label_10.png]
