# Supplementary figures and images for: Adaptive mask-based brain extraction method for head CT images (part 13 of 14)
Source: PLoS One. 2024 Mar 11;19(3):e0295536. doi: 10.1371/journal.pone.0295536 (PMC10927156; doi:10.1371/journal.pone.0295536)

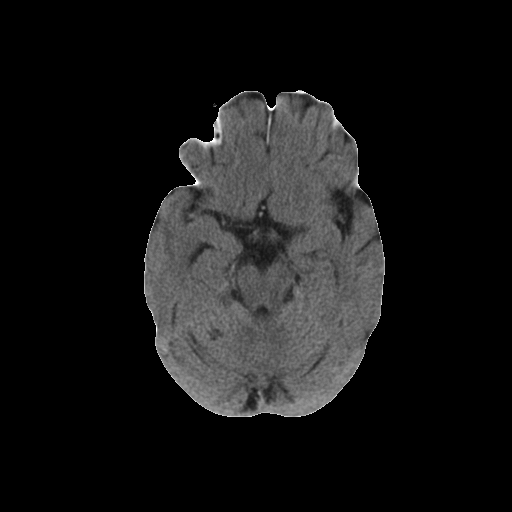

Supplement: S1 Fig — (ZIP) [file pone.0295536.s008.zip › S8_Fig/Segmentation result of AMBBEM with three FCNs in test set 2/AMBBEM/Label_165.png]

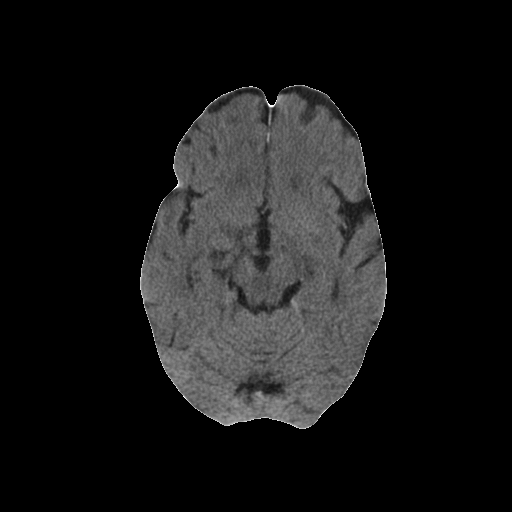

Supplement: S1 Fig — (ZIP) [file pone.0295536.s008.zip › S8_Fig/Segmentation result of AMBBEM with three FCNs in test set 2/AMBBEM/Label_166.png]

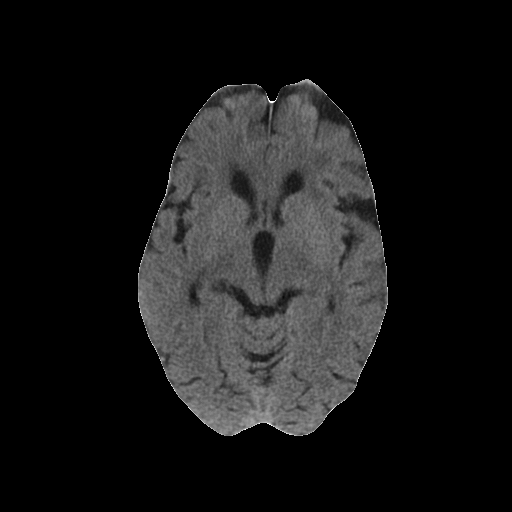

Supplement: S1 Fig — (ZIP) [file pone.0295536.s008.zip › S8_Fig/Segmentation result of AMBBEM with three FCNs in test set 2/AMBBEM/Label_167.png]

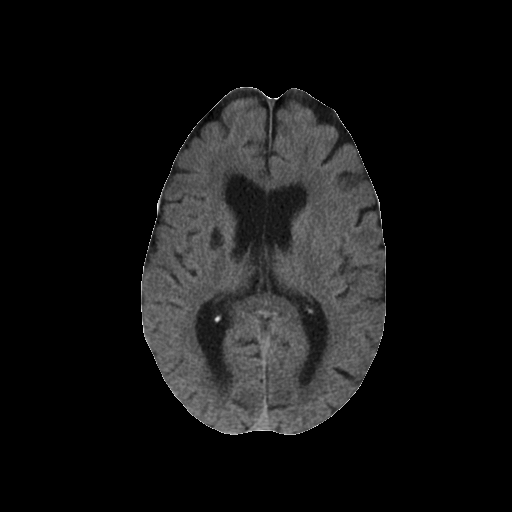

Supplement: S1 Fig — (ZIP) [file pone.0295536.s008.zip › S8_Fig/Segmentation result of AMBBEM with three FCNs in test set 2/AMBBEM/Label_168.png]

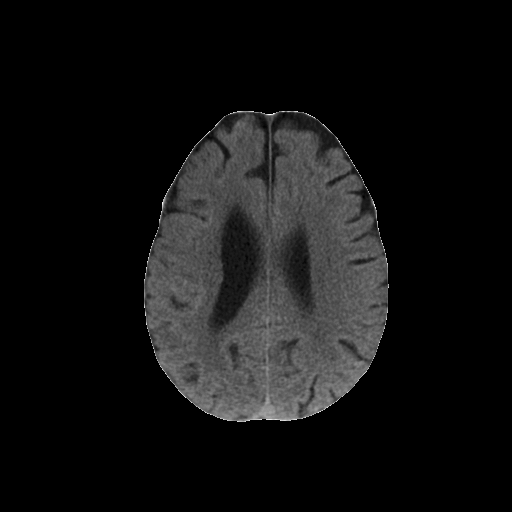

Supplement: S1 Fig — (ZIP) [file pone.0295536.s008.zip › S8_Fig/Segmentation result of AMBBEM with three FCNs in test set 2/AMBBEM/Label_169.png]

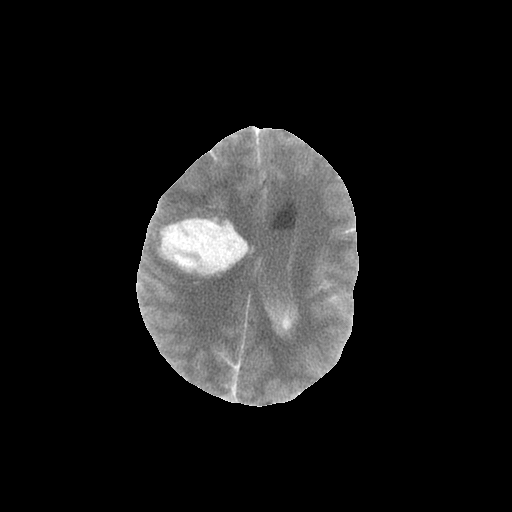

Supplement: S1 Fig — (ZIP) [file pone.0295536.s008.zip › S8_Fig/Segmentation result of AMBBEM with three FCNs in test set 2/AMBBEM/Label_17.png]

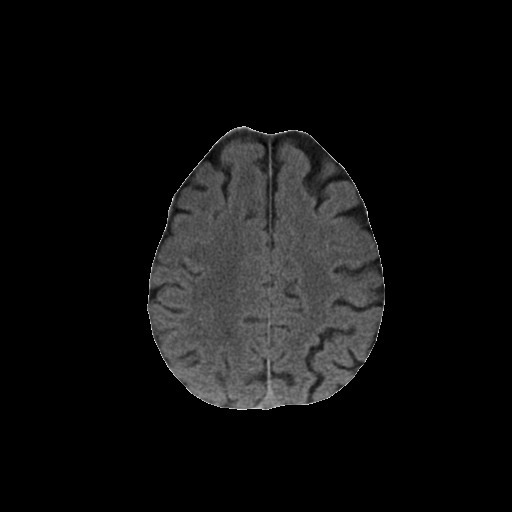

Supplement: S1 Fig — (ZIP) [file pone.0295536.s008.zip › S8_Fig/Segmentation result of AMBBEM with three FCNs in test set 2/AMBBEM/Label_170.png]

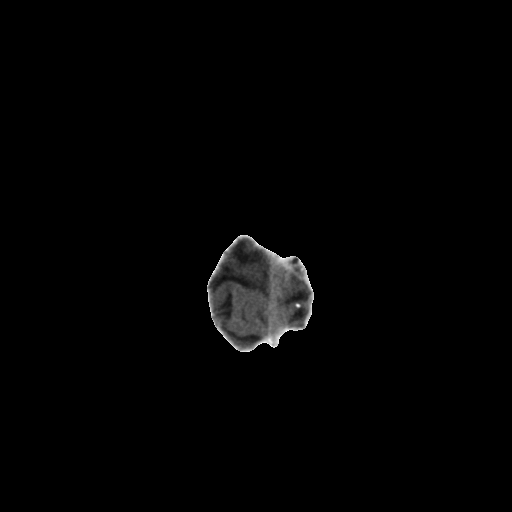

Supplement: S1 Fig — (ZIP) [file pone.0295536.s008.zip › S8_Fig/Segmentation result of AMBBEM with three FCNs in test set 2/AMBBEM/Label_171.png]

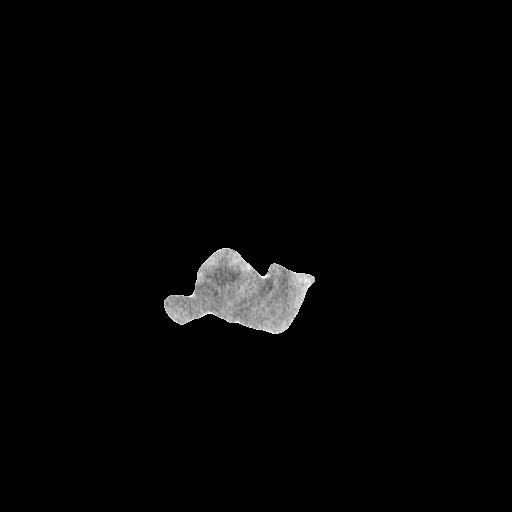

Supplement: S1 Fig — (ZIP) [file pone.0295536.s008.zip › S8_Fig/Segmentation result of AMBBEM with three FCNs in test set 2/AMBBEM/Label_172.png]

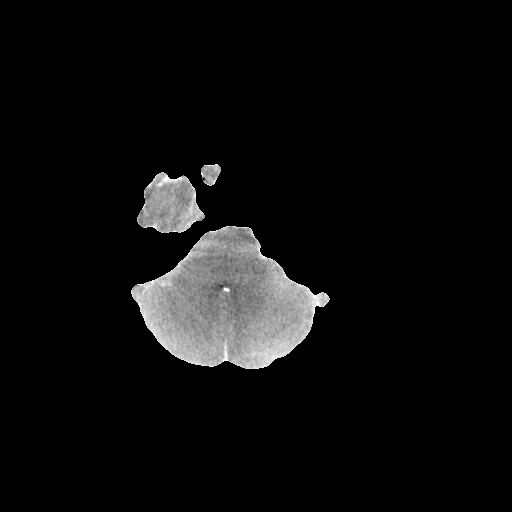

Supplement: S1 Fig — (ZIP) [file pone.0295536.s008.zip › S8_Fig/Segmentation result of AMBBEM with three FCNs in test set 2/AMBBEM/Label_173.png]

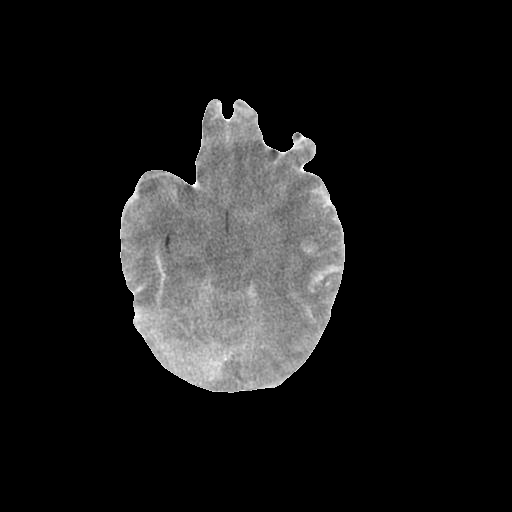

Supplement: S1 Fig — (ZIP) [file pone.0295536.s008.zip › S8_Fig/Segmentation result of AMBBEM with three FCNs in test set 2/AMBBEM/Label_174.png]

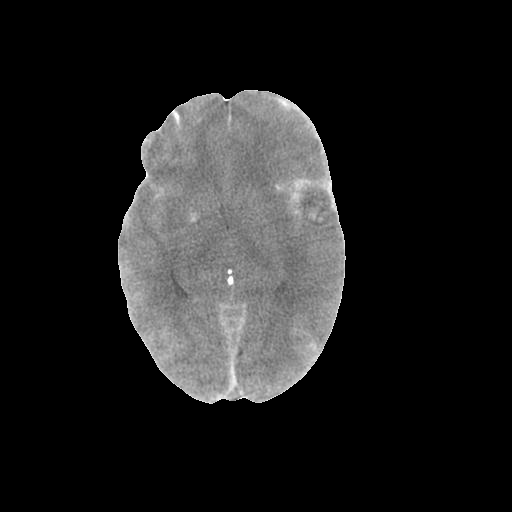

Supplement: S1 Fig — (ZIP) [file pone.0295536.s008.zip › S8_Fig/Segmentation result of AMBBEM with three FCNs in test set 2/AMBBEM/Label_175.png]

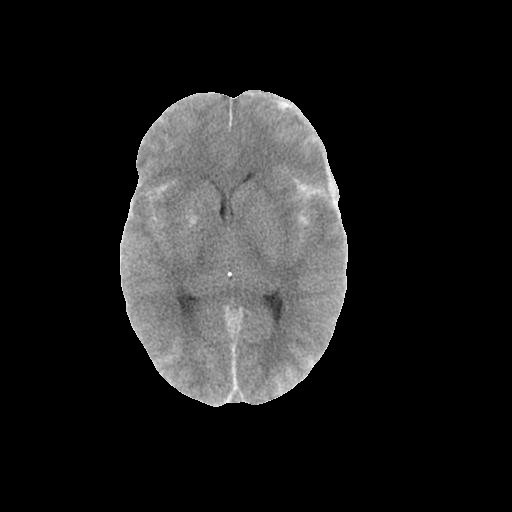

Supplement: S1 Fig — (ZIP) [file pone.0295536.s008.zip › S8_Fig/Segmentation result of AMBBEM with three FCNs in test set 2/AMBBEM/Label_176.png]

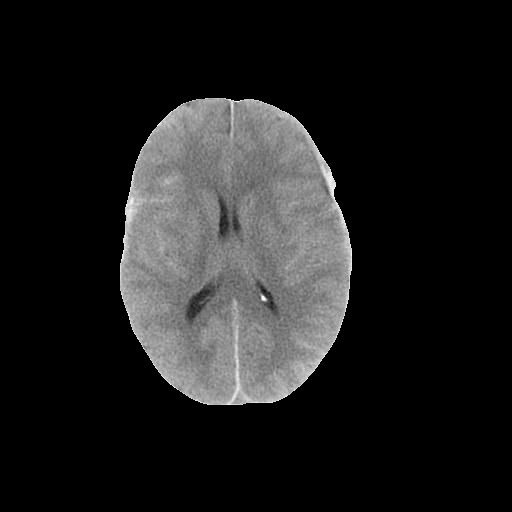

Supplement: S1 Fig — (ZIP) [file pone.0295536.s008.zip › S8_Fig/Segmentation result of AMBBEM with three FCNs in test set 2/AMBBEM/Label_177.png]

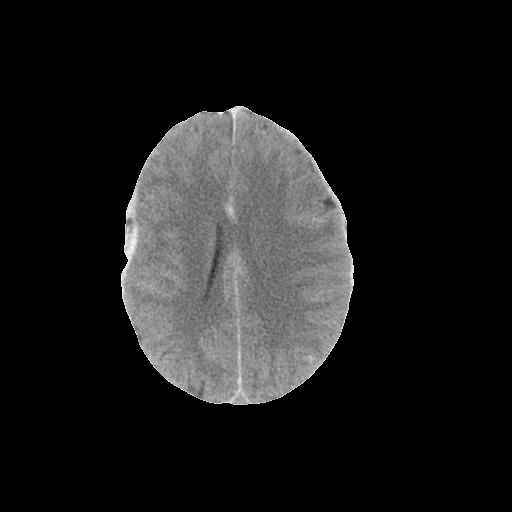

Supplement: S1 Fig — (ZIP) [file pone.0295536.s008.zip › S8_Fig/Segmentation result of AMBBEM with three FCNs in test set 2/AMBBEM/Label_178.png]

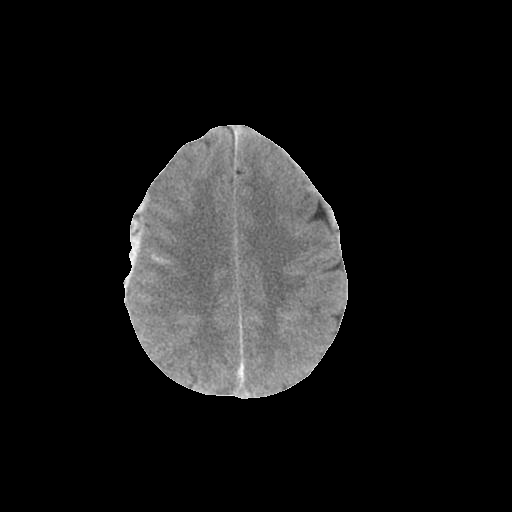

Supplement: S1 Fig — (ZIP) [file pone.0295536.s008.zip › S8_Fig/Segmentation result of AMBBEM with three FCNs in test set 2/AMBBEM/Label_179.png]

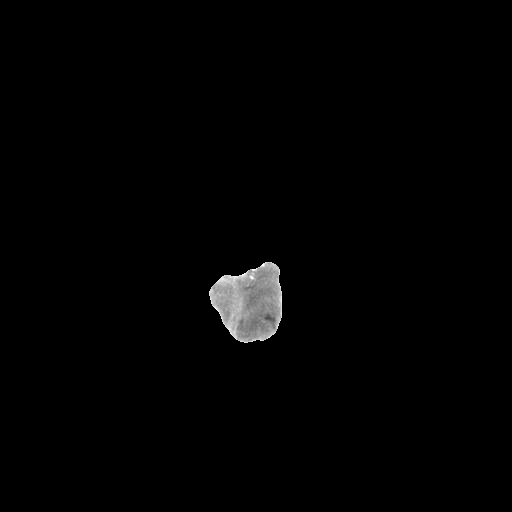

Supplement: S1 Fig — (ZIP) [file pone.0295536.s008.zip › S8_Fig/Segmentation result of AMBBEM with three FCNs in test set 2/AMBBEM/Label_18.png]

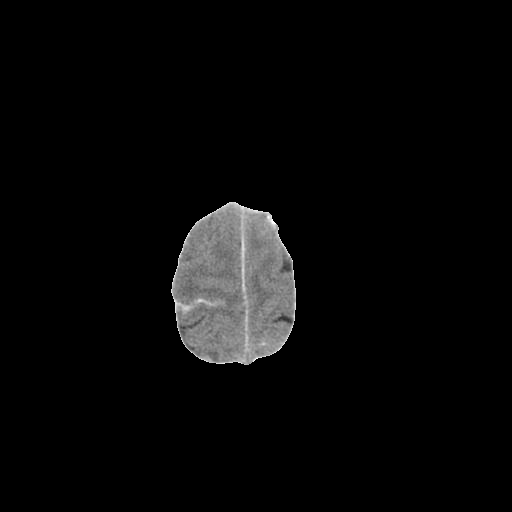

Supplement: S1 Fig — (ZIP) [file pone.0295536.s008.zip › S8_Fig/Segmentation result of AMBBEM with three FCNs in test set 2/AMBBEM/Label_180.png]

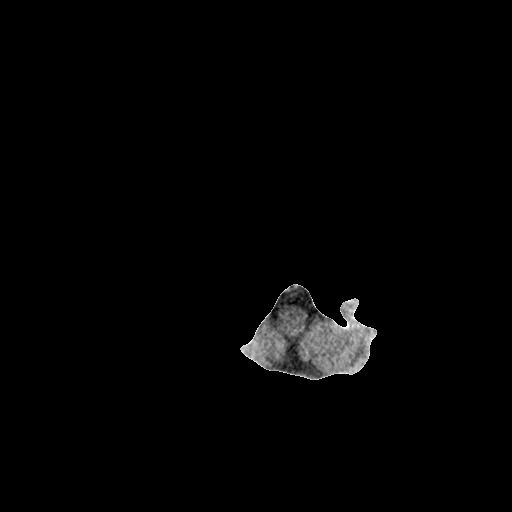

Supplement: S1 Fig — (ZIP) [file pone.0295536.s008.zip › S8_Fig/Segmentation result of AMBBEM with three FCNs in test set 2/AMBBEM/Label_181.png]

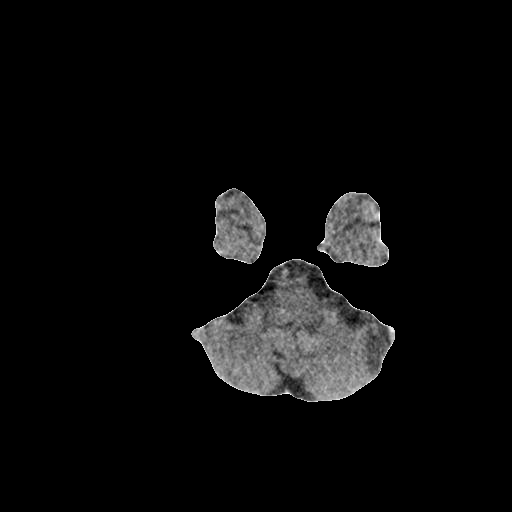

Supplement: S1 Fig — (ZIP) [file pone.0295536.s008.zip › S8_Fig/Segmentation result of AMBBEM with three FCNs in test set 2/AMBBEM/Label_182.png]

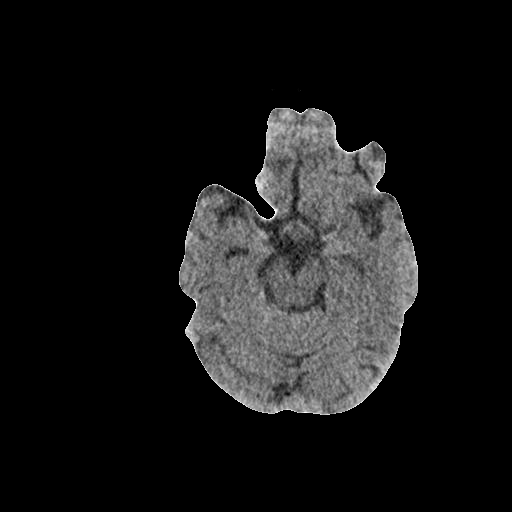

Supplement: S1 Fig — (ZIP) [file pone.0295536.s008.zip › S8_Fig/Segmentation result of AMBBEM with three FCNs in test set 2/AMBBEM/Label_183.png]

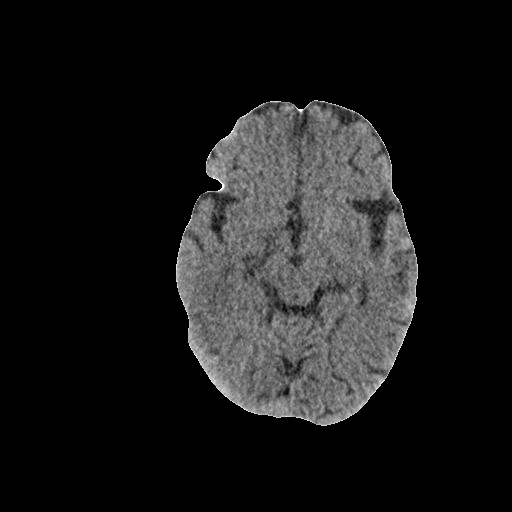

Supplement: S1 Fig — (ZIP) [file pone.0295536.s008.zip › S8_Fig/Segmentation result of AMBBEM with three FCNs in test set 2/AMBBEM/Label_184.png]

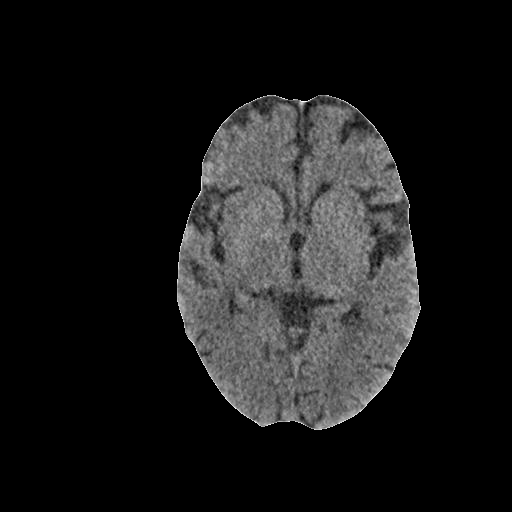

Supplement: S1 Fig — (ZIP) [file pone.0295536.s008.zip › S8_Fig/Segmentation result of AMBBEM with three FCNs in test set 2/AMBBEM/Label_185.png]

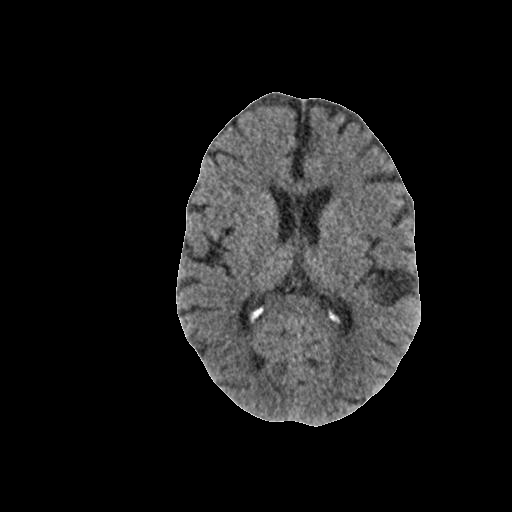

Supplement: S1 Fig — (ZIP) [file pone.0295536.s008.zip › S8_Fig/Segmentation result of AMBBEM with three FCNs in test set 2/AMBBEM/Label_186.png]

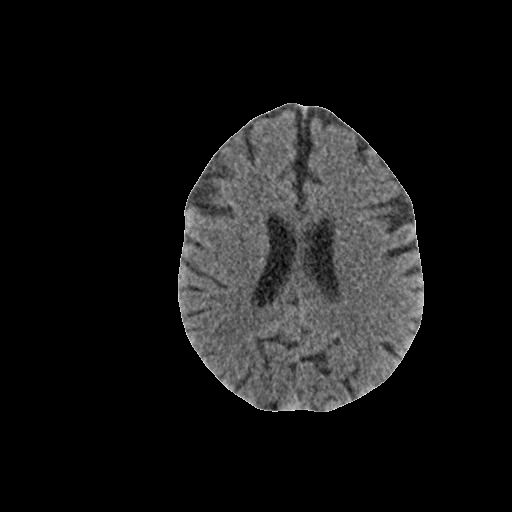

Supplement: S1 Fig — (ZIP) [file pone.0295536.s008.zip › S8_Fig/Segmentation result of AMBBEM with three FCNs in test set 2/AMBBEM/Label_187.png]

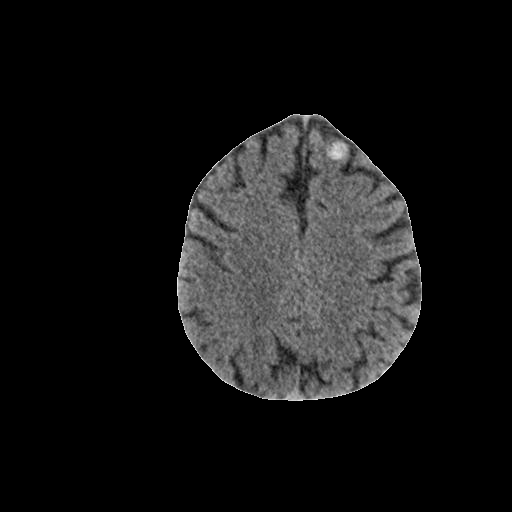

Supplement: S1 Fig — (ZIP) [file pone.0295536.s008.zip › S8_Fig/Segmentation result of AMBBEM with three FCNs in test set 2/AMBBEM/Label_188.png]

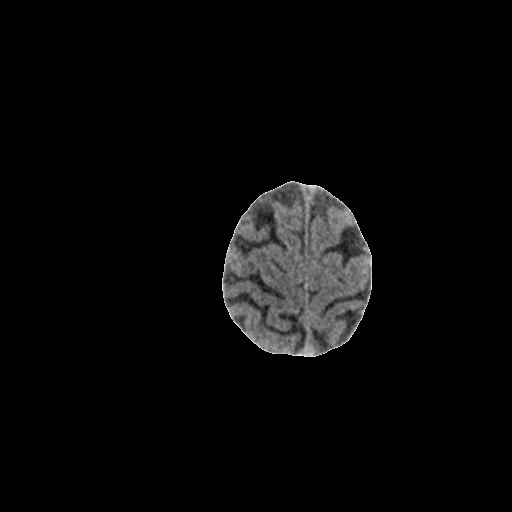

Supplement: S1 Fig — (ZIP) [file pone.0295536.s008.zip › S8_Fig/Segmentation result of AMBBEM with three FCNs in test set 2/AMBBEM/Label_189.png]

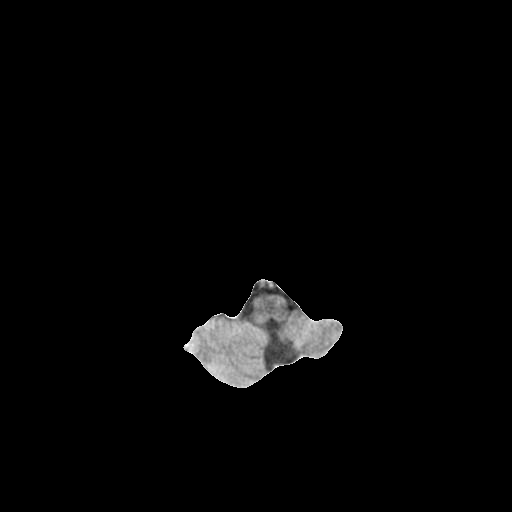

Supplement: S1 Fig — (ZIP) [file pone.0295536.s008.zip › S8_Fig/Segmentation result of AMBBEM with three FCNs in test set 2/AMBBEM/Label_19.png]

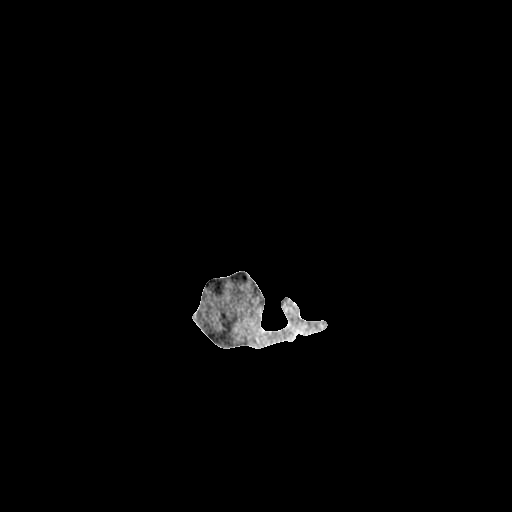

Supplement: S1 Fig — (ZIP) [file pone.0295536.s008.zip › S8_Fig/Segmentation result of AMBBEM with three FCNs in test set 2/AMBBEM/Label_190.png]

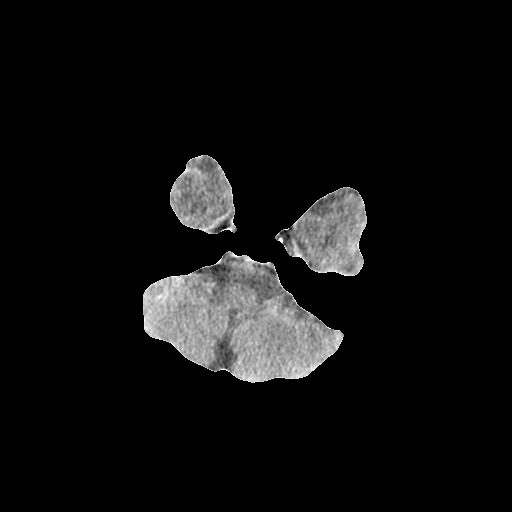

Supplement: S1 Fig — (ZIP) [file pone.0295536.s008.zip › S8_Fig/Segmentation result of AMBBEM with three FCNs in test set 2/AMBBEM/Label_191.png]

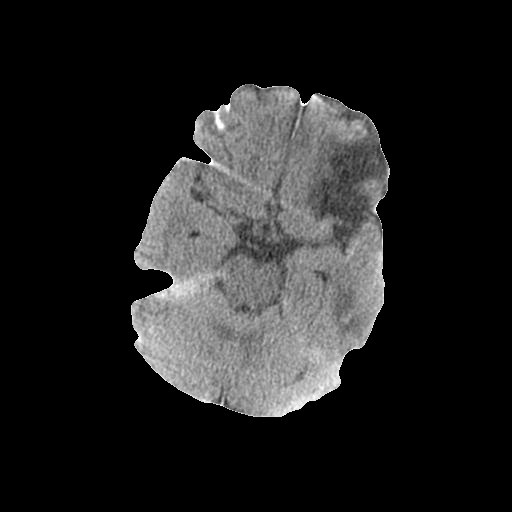

Supplement: S1 Fig — (ZIP) [file pone.0295536.s008.zip › S8_Fig/Segmentation result of AMBBEM with three FCNs in test set 2/AMBBEM/Label_192.png]

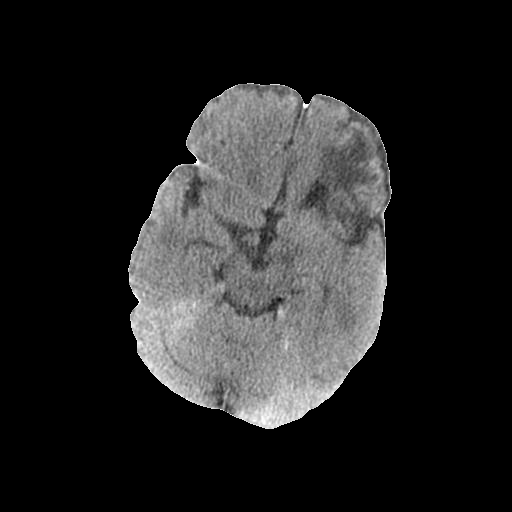

Supplement: S1 Fig — (ZIP) [file pone.0295536.s008.zip › S8_Fig/Segmentation result of AMBBEM with three FCNs in test set 2/AMBBEM/Label_193.png]

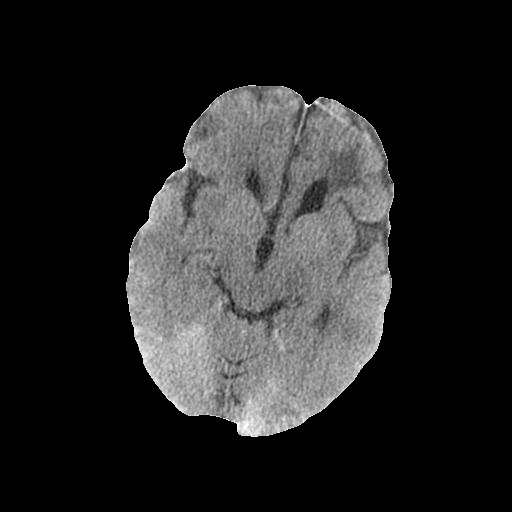

Supplement: S1 Fig — (ZIP) [file pone.0295536.s008.zip › S8_Fig/Segmentation result of AMBBEM with three FCNs in test set 2/AMBBEM/Label_194.png]

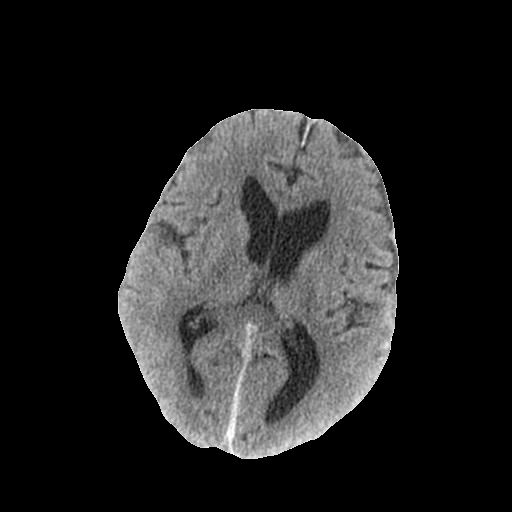

Supplement: S1 Fig — (ZIP) [file pone.0295536.s008.zip › S8_Fig/Segmentation result of AMBBEM with three FCNs in test set 2/AMBBEM/Label_195.png]

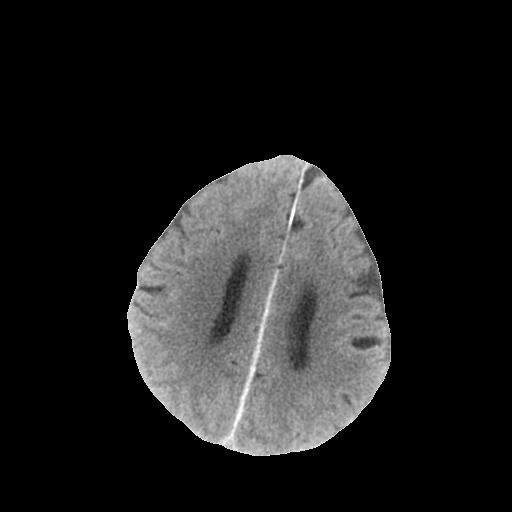

Supplement: S1 Fig — (ZIP) [file pone.0295536.s008.zip › S8_Fig/Segmentation result of AMBBEM with three FCNs in test set 2/AMBBEM/Label_196.png]

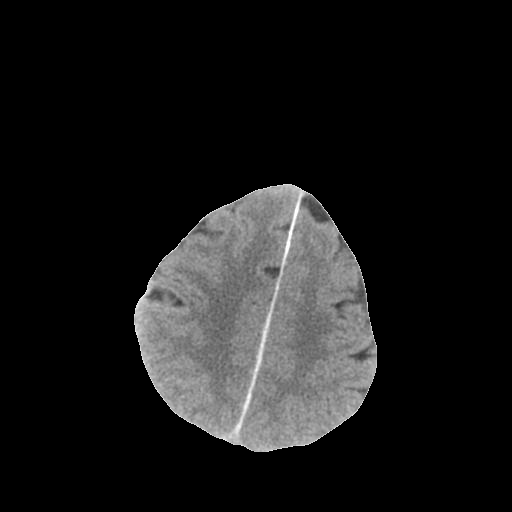

Supplement: S1 Fig — (ZIP) [file pone.0295536.s008.zip › S8_Fig/Segmentation result of AMBBEM with three FCNs in test set 2/AMBBEM/Label_197.png]

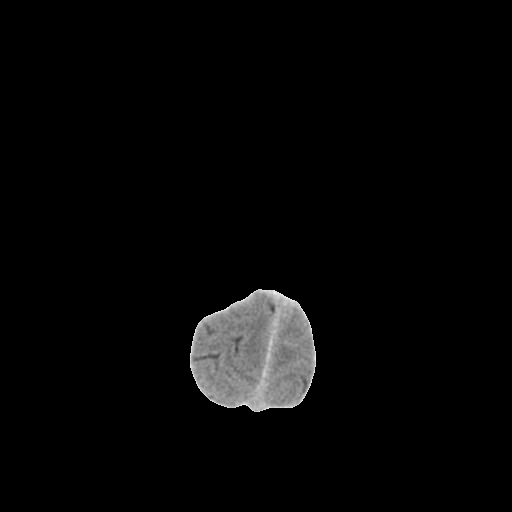

Supplement: S1 Fig — (ZIP) [file pone.0295536.s008.zip › S8_Fig/Segmentation result of AMBBEM with three FCNs in test set 2/AMBBEM/Label_198.png]

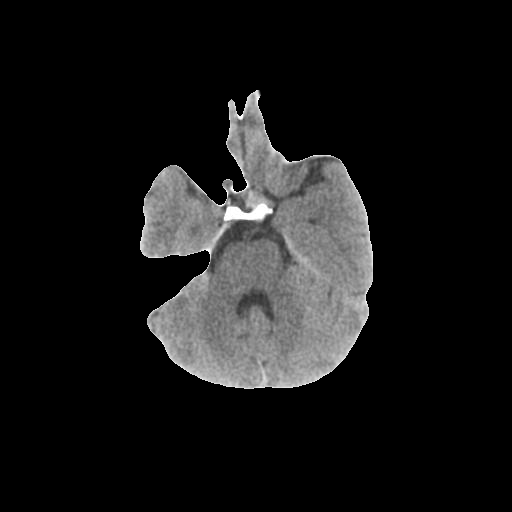

Supplement: S1 Fig — (ZIP) [file pone.0295536.s008.zip › S8_Fig/Segmentation result of AMBBEM with three FCNs in test set 2/AMBBEM/Label_2.png]

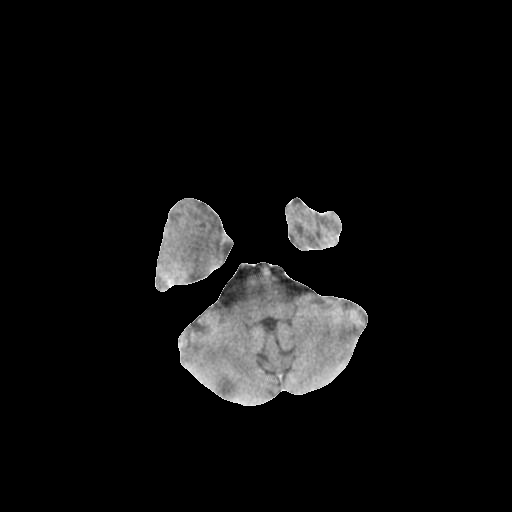

Supplement: S1 Fig — (ZIP) [file pone.0295536.s008.zip › S8_Fig/Segmentation result of AMBBEM with three FCNs in test set 2/AMBBEM/Label_20.png]

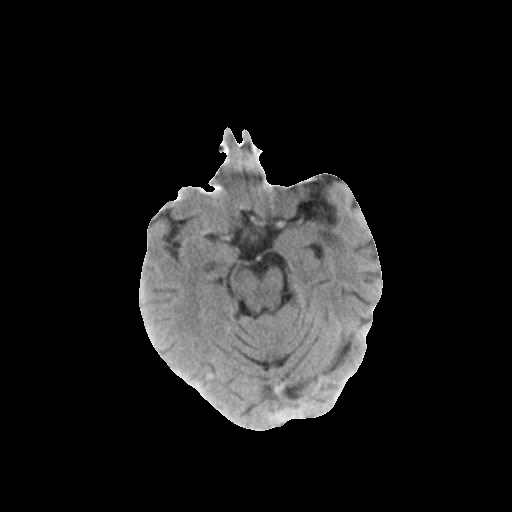

Supplement: S1 Fig — (ZIP) [file pone.0295536.s008.zip › S8_Fig/Segmentation result of AMBBEM with three FCNs in test set 2/AMBBEM/Label_21.png]

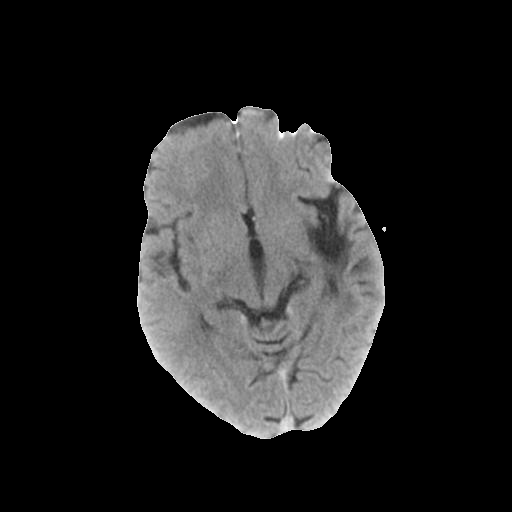

Supplement: S1 Fig — (ZIP) [file pone.0295536.s008.zip › S8_Fig/Segmentation result of AMBBEM with three FCNs in test set 2/AMBBEM/Label_22.png]

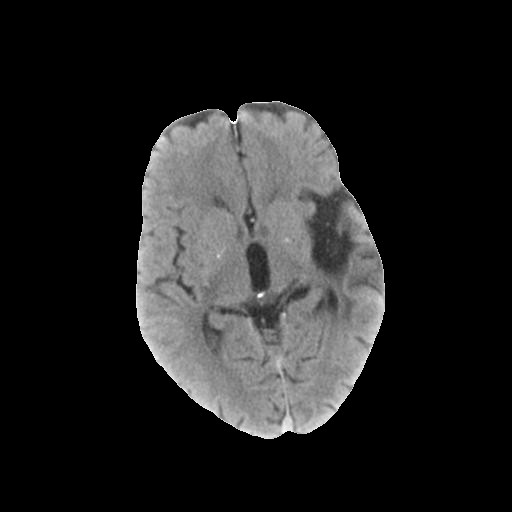

Supplement: S1 Fig — (ZIP) [file pone.0295536.s008.zip › S8_Fig/Segmentation result of AMBBEM with three FCNs in test set 2/AMBBEM/Label_23.png]

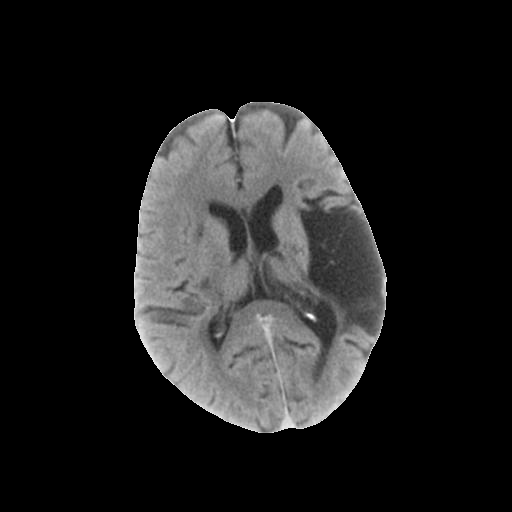

Supplement: S1 Fig — (ZIP) [file pone.0295536.s008.zip › S8_Fig/Segmentation result of AMBBEM with three FCNs in test set 2/AMBBEM/Label_24.png]

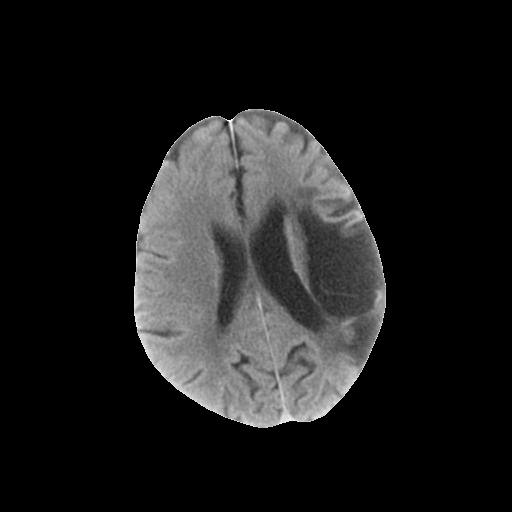

Supplement: S1 Fig — (ZIP) [file pone.0295536.s008.zip › S8_Fig/Segmentation result of AMBBEM with three FCNs in test set 2/AMBBEM/Label_25.png]

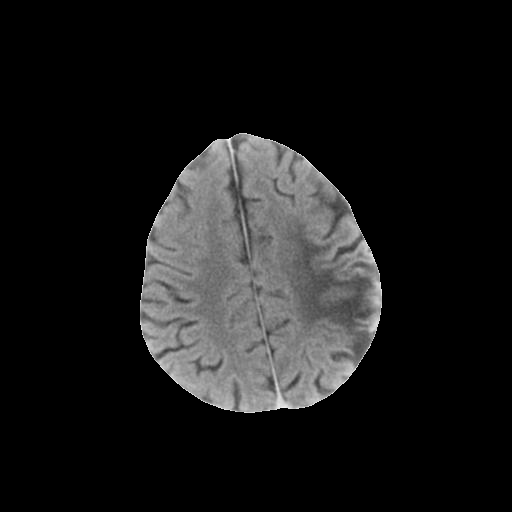

Supplement: S1 Fig — (ZIP) [file pone.0295536.s008.zip › S8_Fig/Segmentation result of AMBBEM with three FCNs in test set 2/AMBBEM/Label_26.png]

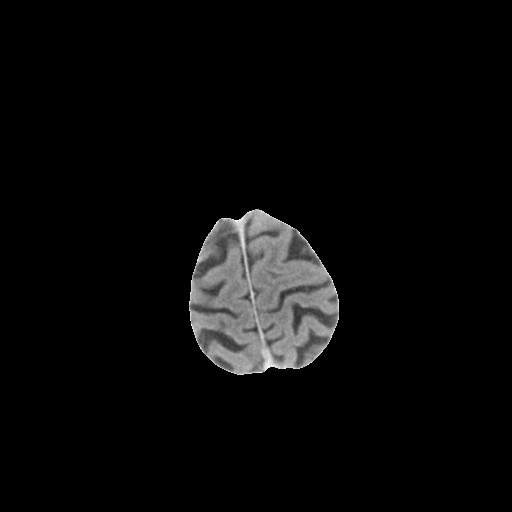

Supplement: S1 Fig — (ZIP) [file pone.0295536.s008.zip › S8_Fig/Segmentation result of AMBBEM with three FCNs in test set 2/AMBBEM/Label_27.png]

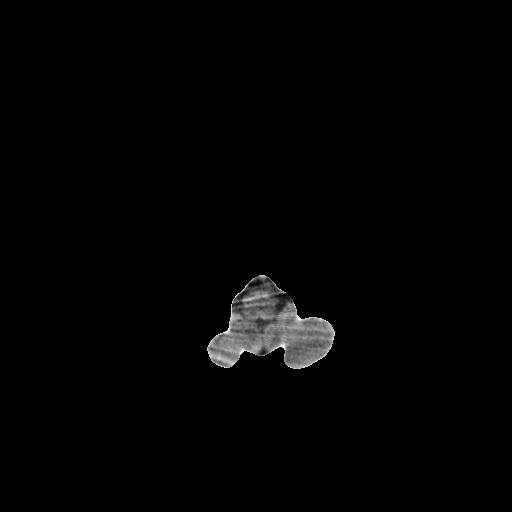

Supplement: S1 Fig — (ZIP) [file pone.0295536.s008.zip › S8_Fig/Segmentation result of AMBBEM with three FCNs in test set 2/AMBBEM/Label_28.png]

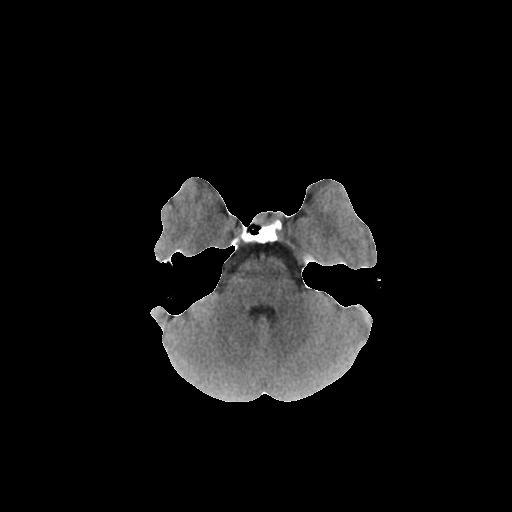

Supplement: S1 Fig — (ZIP) [file pone.0295536.s008.zip › S8_Fig/Segmentation result of AMBBEM with three FCNs in test set 2/AMBBEM/Label_29.png]

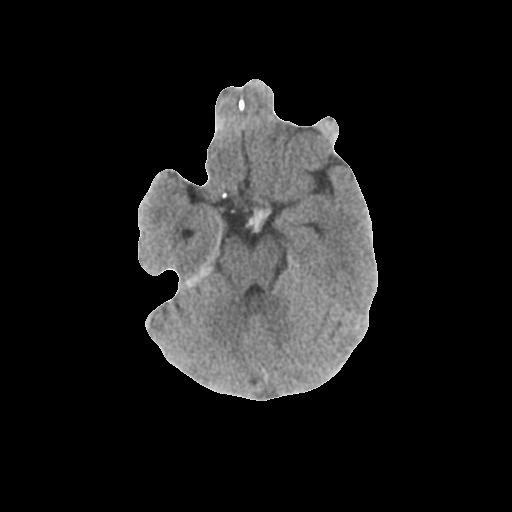

Supplement: S1 Fig — (ZIP) [file pone.0295536.s008.zip › S8_Fig/Segmentation result of AMBBEM with three FCNs in test set 2/AMBBEM/Label_3.png]

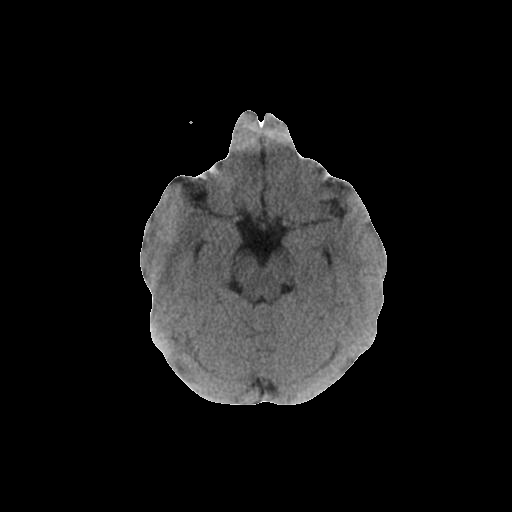

Supplement: S1 Fig — (ZIP) [file pone.0295536.s008.zip › S8_Fig/Segmentation result of AMBBEM with three FCNs in test set 2/AMBBEM/Label_30.png]

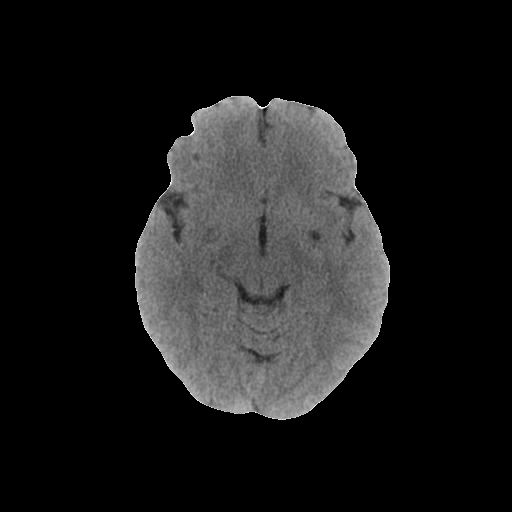

Supplement: S1 Fig — (ZIP) [file pone.0295536.s008.zip › S8_Fig/Segmentation result of AMBBEM with three FCNs in test set 2/AMBBEM/Label_31.png]

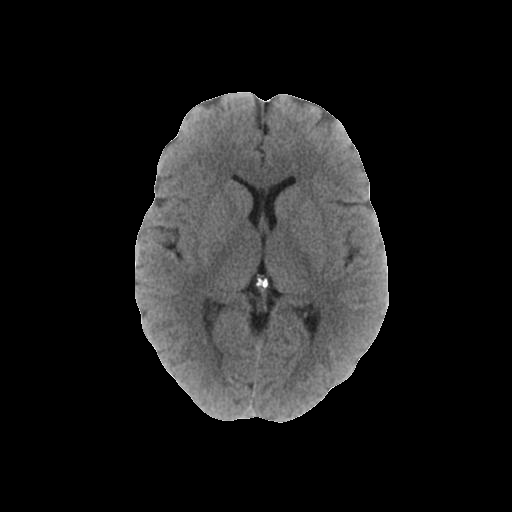

Supplement: S1 Fig — (ZIP) [file pone.0295536.s008.zip › S8_Fig/Segmentation result of AMBBEM with three FCNs in test set 2/AMBBEM/Label_32.png]

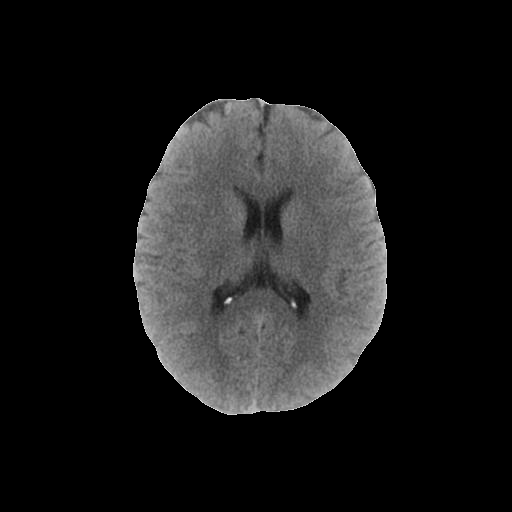

Supplement: S1 Fig — (ZIP) [file pone.0295536.s008.zip › S8_Fig/Segmentation result of AMBBEM with three FCNs in test set 2/AMBBEM/Label_33.png]

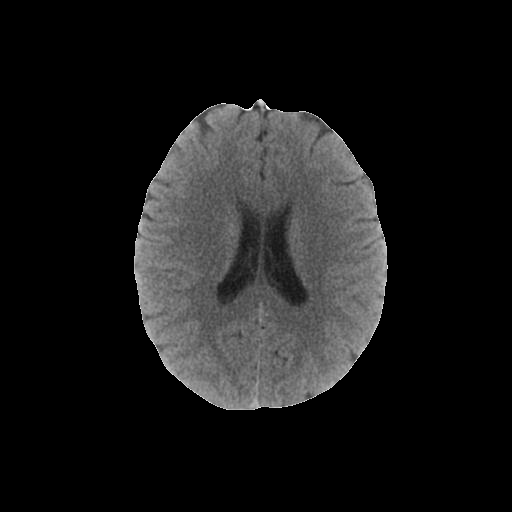

Supplement: S1 Fig — (ZIP) [file pone.0295536.s008.zip › S8_Fig/Segmentation result of AMBBEM with three FCNs in test set 2/AMBBEM/Label_34.png]

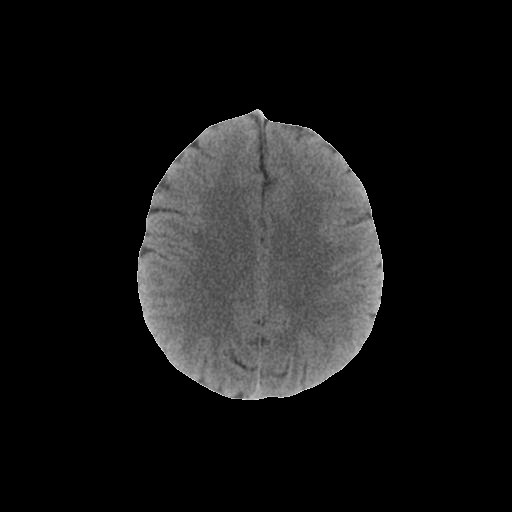

Supplement: S1 Fig — (ZIP) [file pone.0295536.s008.zip › S8_Fig/Segmentation result of AMBBEM with three FCNs in test set 2/AMBBEM/Label_35.png]

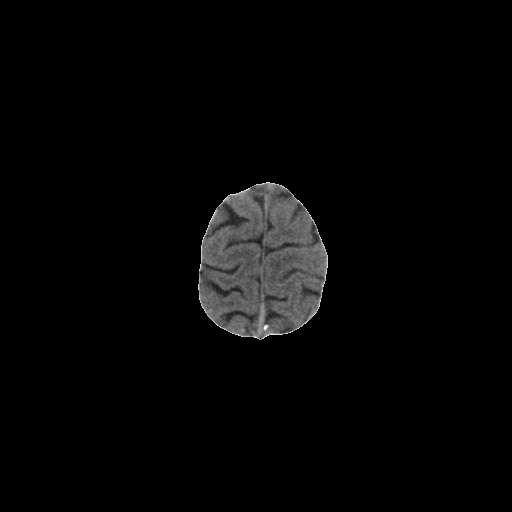

Supplement: S1 Fig — (ZIP) [file pone.0295536.s008.zip › S8_Fig/Segmentation result of AMBBEM with three FCNs in test set 2/AMBBEM/Label_36.png]

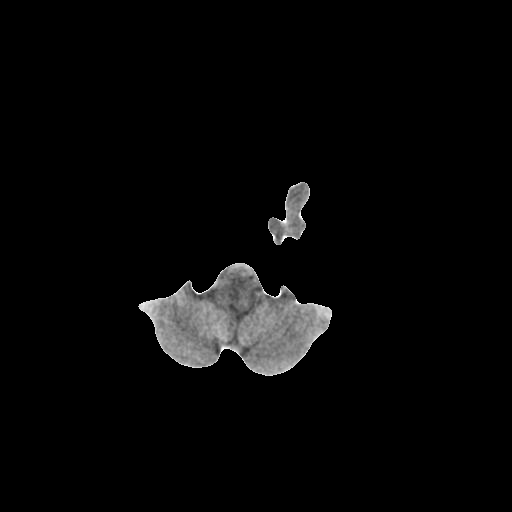

Supplement: S1 Fig — (ZIP) [file pone.0295536.s008.zip › S8_Fig/Segmentation result of AMBBEM with three FCNs in test set 2/AMBBEM/Label_37.png]

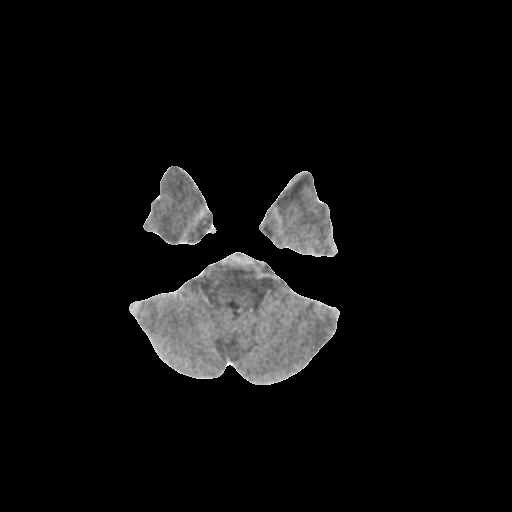

Supplement: S1 Fig — (ZIP) [file pone.0295536.s008.zip › S8_Fig/Segmentation result of AMBBEM with three FCNs in test set 2/AMBBEM/Label_38.png]

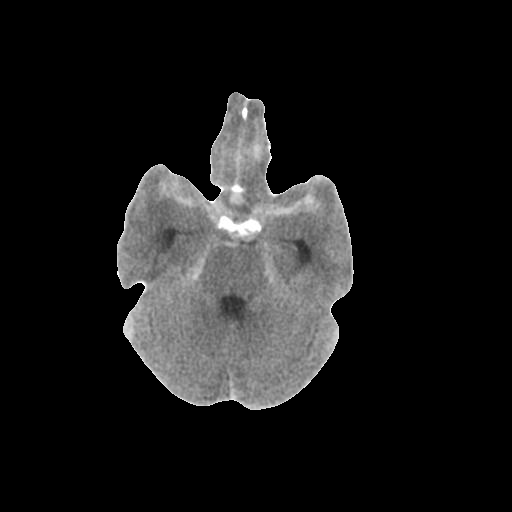

Supplement: S1 Fig — (ZIP) [file pone.0295536.s008.zip › S8_Fig/Segmentation result of AMBBEM with three FCNs in test set 2/AMBBEM/Label_39.png]

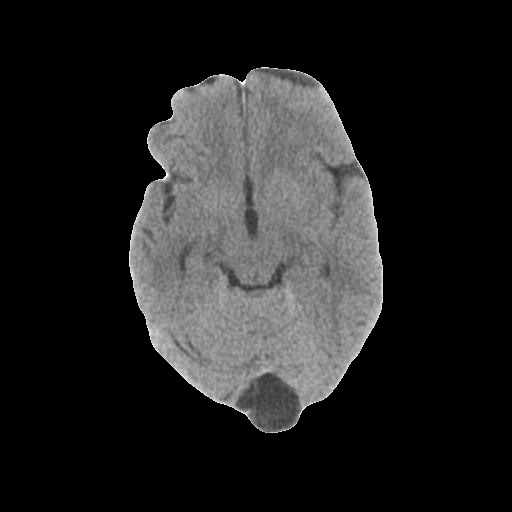

Supplement: S1 Fig — (ZIP) [file pone.0295536.s008.zip › S8_Fig/Segmentation result of AMBBEM with three FCNs in test set 2/AMBBEM/Label_4.png]

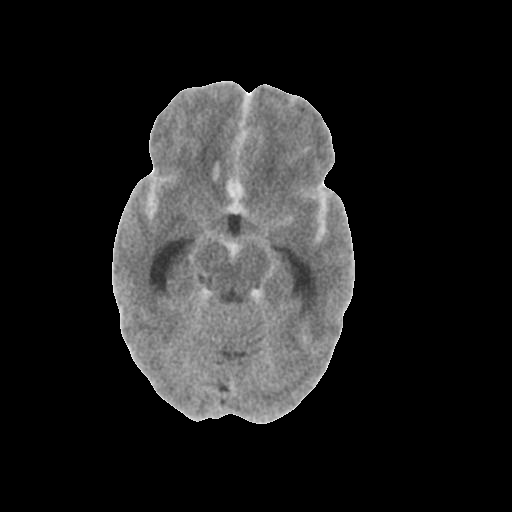

Supplement: S1 Fig — (ZIP) [file pone.0295536.s008.zip › S8_Fig/Segmentation result of AMBBEM with three FCNs in test set 2/AMBBEM/Label_40.png]

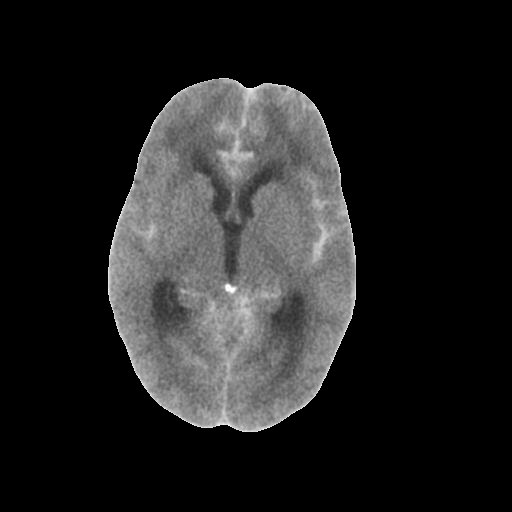

Supplement: S1 Fig — (ZIP) [file pone.0295536.s008.zip › S8_Fig/Segmentation result of AMBBEM with three FCNs in test set 2/AMBBEM/Label_41.png]

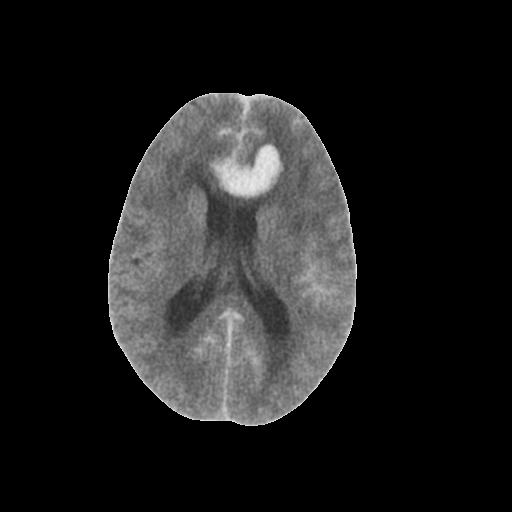

Supplement: S1 Fig — (ZIP) [file pone.0295536.s008.zip › S8_Fig/Segmentation result of AMBBEM with three FCNs in test set 2/AMBBEM/Label_42.png]

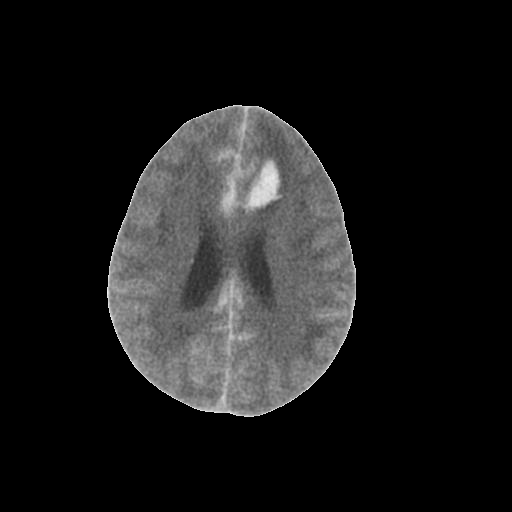

Supplement: S1 Fig — (ZIP) [file pone.0295536.s008.zip › S8_Fig/Segmentation result of AMBBEM with three FCNs in test set 2/AMBBEM/Label_43.png]

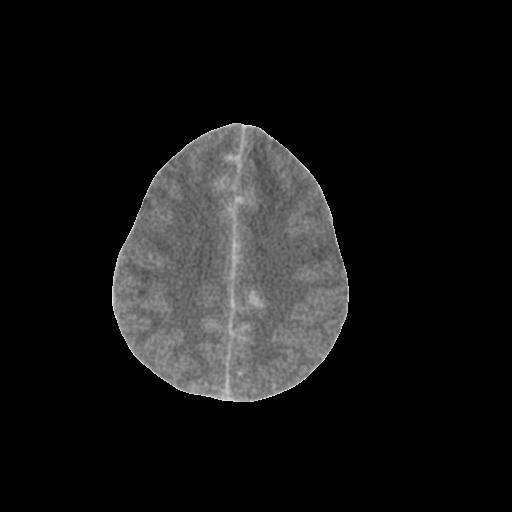

Supplement: S1 Fig — (ZIP) [file pone.0295536.s008.zip › S8_Fig/Segmentation result of AMBBEM with three FCNs in test set 2/AMBBEM/Label_44.png]

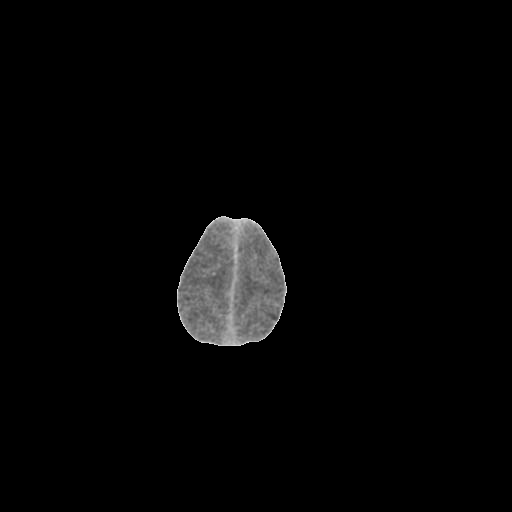

Supplement: S1 Fig — (ZIP) [file pone.0295536.s008.zip › S8_Fig/Segmentation result of AMBBEM with three FCNs in test set 2/AMBBEM/Label_45.png]

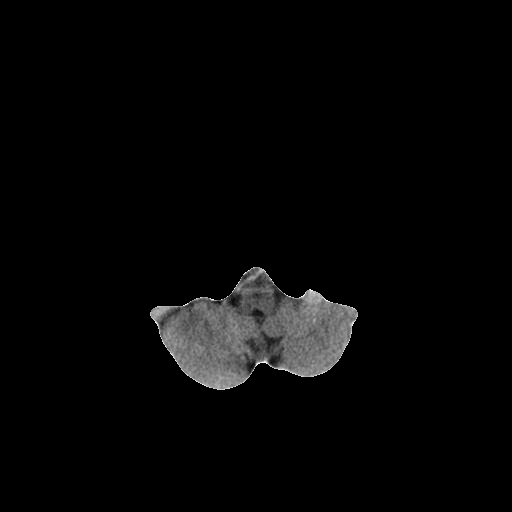

Supplement: S1 Fig — (ZIP) [file pone.0295536.s008.zip › S8_Fig/Segmentation result of AMBBEM with three FCNs in test set 2/AMBBEM/Label_46.png]

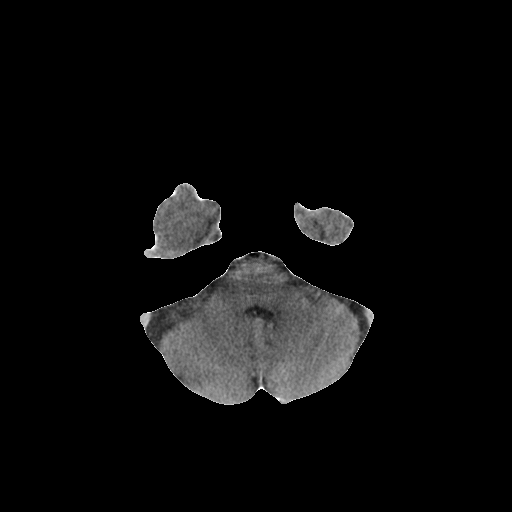

Supplement: S1 Fig — (ZIP) [file pone.0295536.s008.zip › S8_Fig/Segmentation result of AMBBEM with three FCNs in test set 2/AMBBEM/Label_47.png]

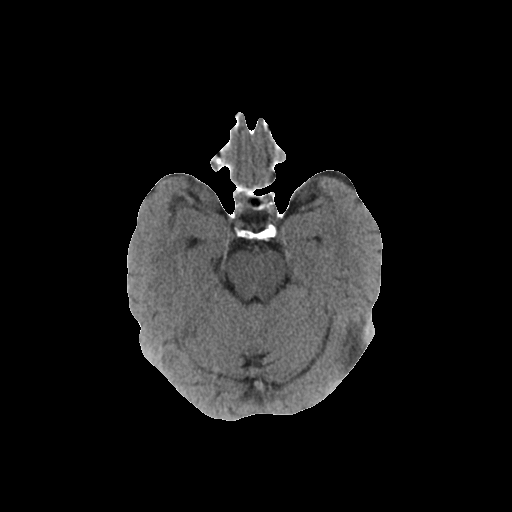

Supplement: S1 Fig — (ZIP) [file pone.0295536.s008.zip › S8_Fig/Segmentation result of AMBBEM with three FCNs in test set 2/AMBBEM/Label_48.png]

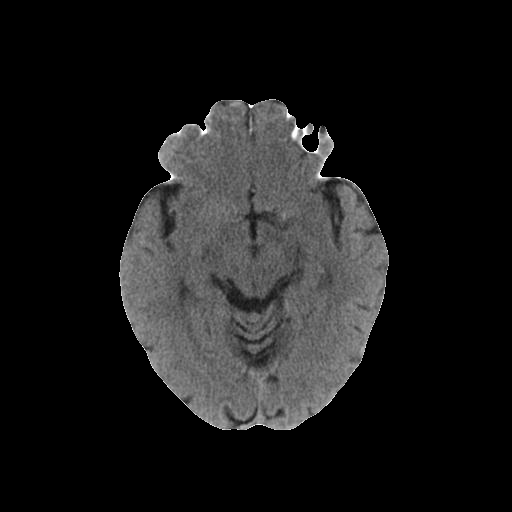

Supplement: S1 Fig — (ZIP) [file pone.0295536.s008.zip › S8_Fig/Segmentation result of AMBBEM with three FCNs in test set 2/AMBBEM/Label_49.png]

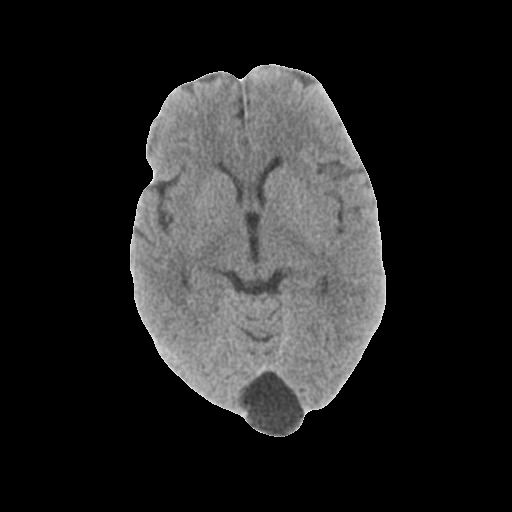

Supplement: S1 Fig — (ZIP) [file pone.0295536.s008.zip › S8_Fig/Segmentation result of AMBBEM with three FCNs in test set 2/AMBBEM/Label_5.png]

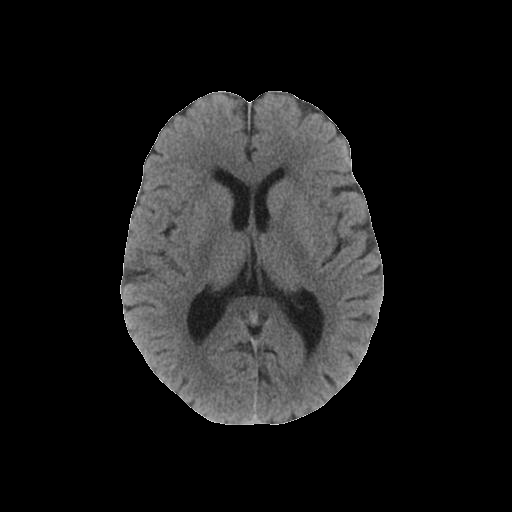

Supplement: S1 Fig — (ZIP) [file pone.0295536.s008.zip › S8_Fig/Segmentation result of AMBBEM with three FCNs in test set 2/AMBBEM/Label_50.png]

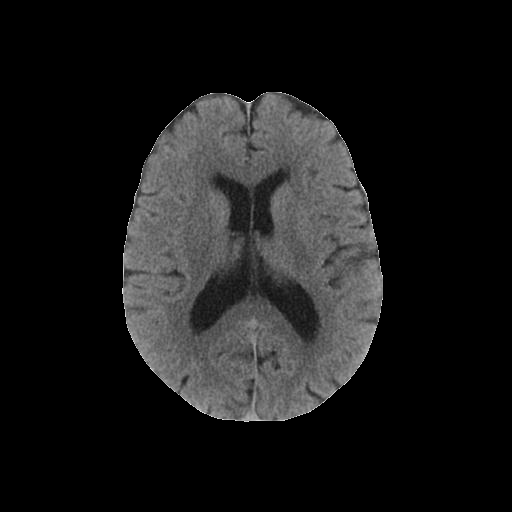

Supplement: S1 Fig — (ZIP) [file pone.0295536.s008.zip › S8_Fig/Segmentation result of AMBBEM with three FCNs in test set 2/AMBBEM/Label_51.png]

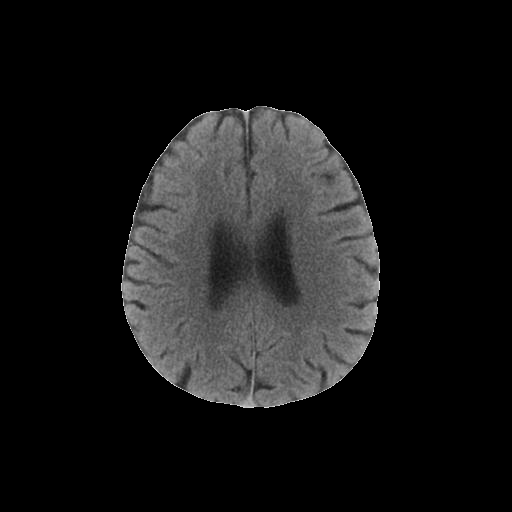

Supplement: S1 Fig — (ZIP) [file pone.0295536.s008.zip › S8_Fig/Segmentation result of AMBBEM with three FCNs in test set 2/AMBBEM/Label_52.png]

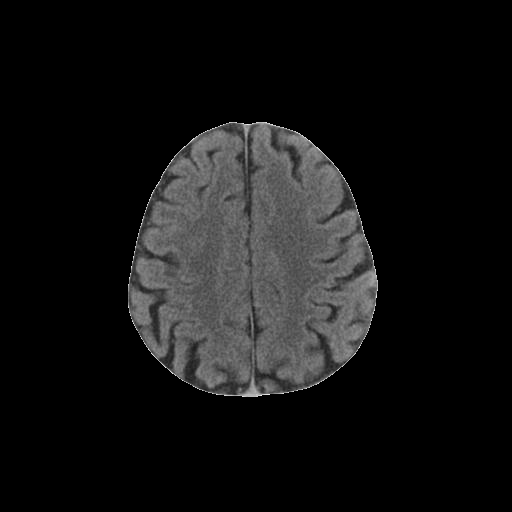

Supplement: S1 Fig — (ZIP) [file pone.0295536.s008.zip › S8_Fig/Segmentation result of AMBBEM with three FCNs in test set 2/AMBBEM/Label_53.png]

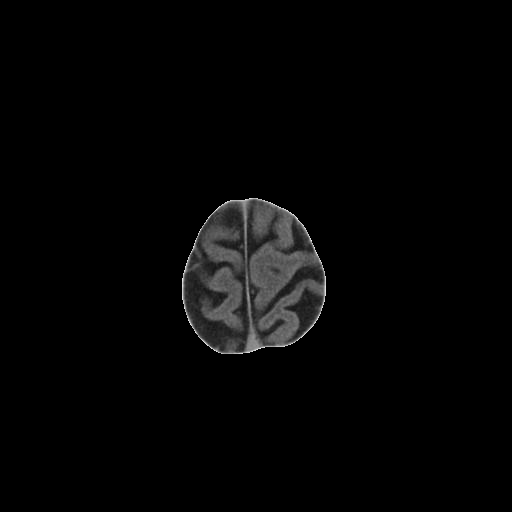

Supplement: S1 Fig — (ZIP) [file pone.0295536.s008.zip › S8_Fig/Segmentation result of AMBBEM with three FCNs in test set 2/AMBBEM/Label_54.png]

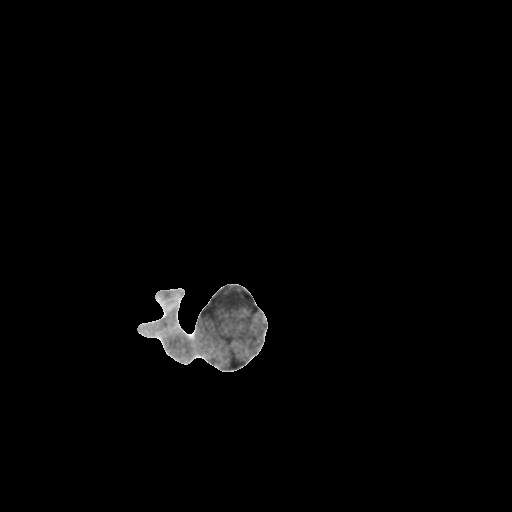

Supplement: S1 Fig — (ZIP) [file pone.0295536.s008.zip › S8_Fig/Segmentation result of AMBBEM with three FCNs in test set 2/AMBBEM/Label_55.png]

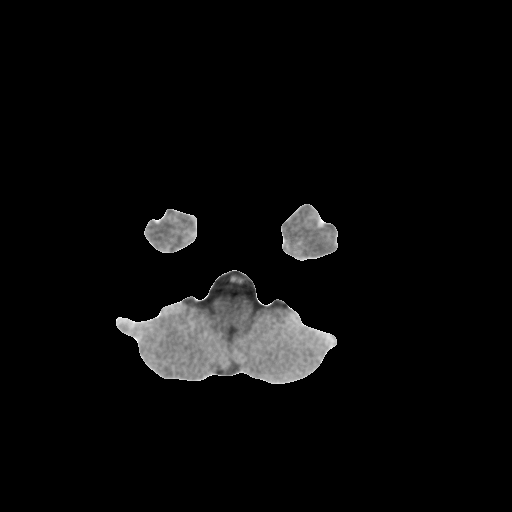

Supplement: S1 Fig — (ZIP) [file pone.0295536.s008.zip › S8_Fig/Segmentation result of AMBBEM with three FCNs in test set 2/AMBBEM/Label_56.png]

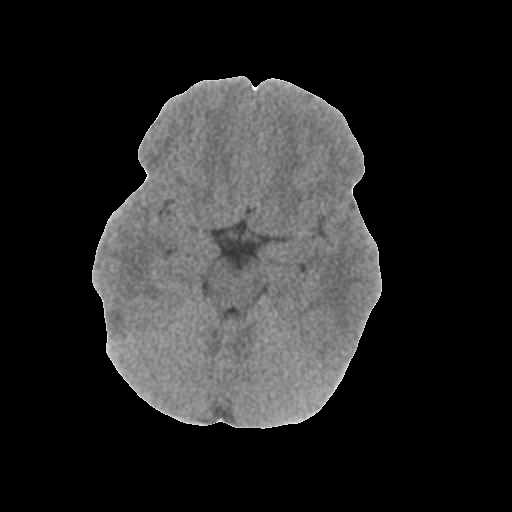

Supplement: S1 Fig — (ZIP) [file pone.0295536.s008.zip › S8_Fig/Segmentation result of AMBBEM with three FCNs in test set 2/AMBBEM/Label_57.png]

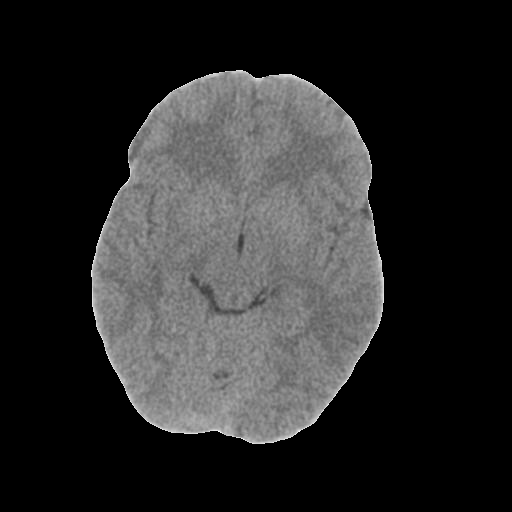

Supplement: S1 Fig — (ZIP) [file pone.0295536.s008.zip › S8_Fig/Segmentation result of AMBBEM with three FCNs in test set 2/AMBBEM/Label_58.png]

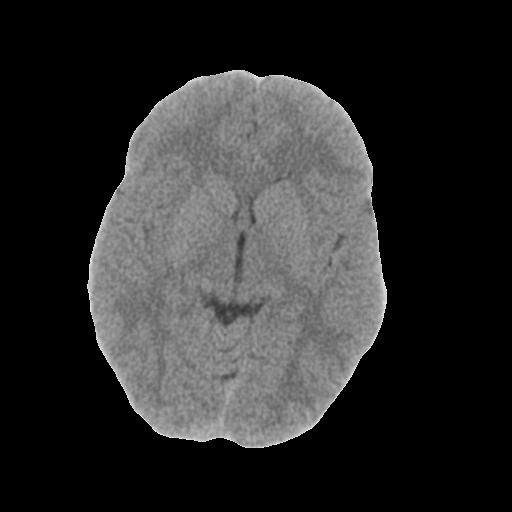

Supplement: S1 Fig — (ZIP) [file pone.0295536.s008.zip › S8_Fig/Segmentation result of AMBBEM with three FCNs in test set 2/AMBBEM/Label_59.png]

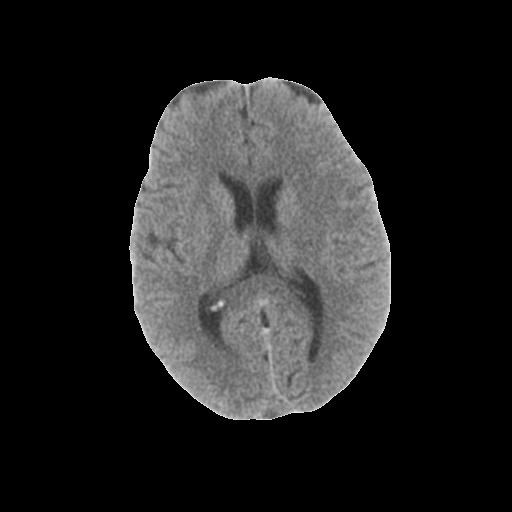

Supplement: S1 Fig — (ZIP) [file pone.0295536.s008.zip › S8_Fig/Segmentation result of AMBBEM with three FCNs in test set 2/AMBBEM/Label_6.png]

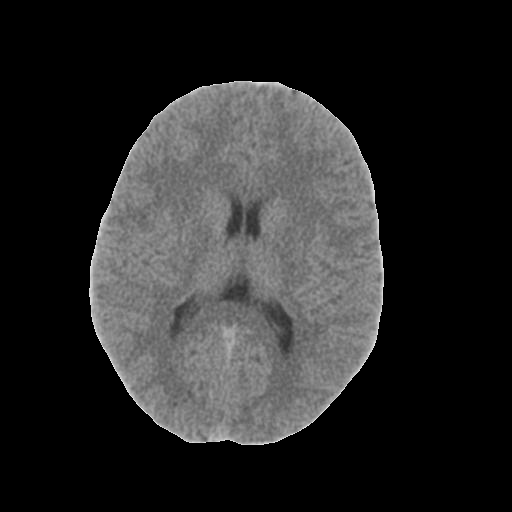

Supplement: S1 Fig — (ZIP) [file pone.0295536.s008.zip › S8_Fig/Segmentation result of AMBBEM with three FCNs in test set 2/AMBBEM/Label_60.png]

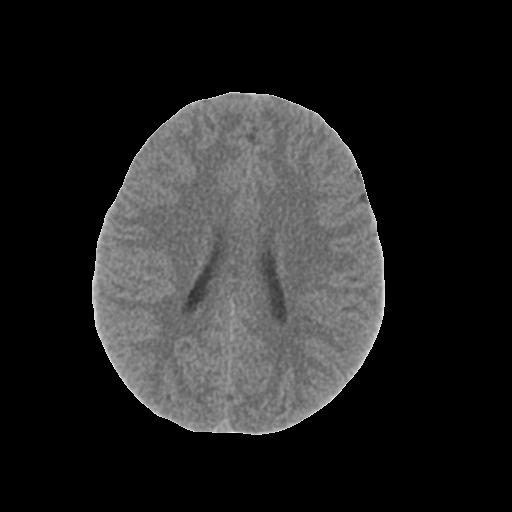

Supplement: S1 Fig — (ZIP) [file pone.0295536.s008.zip › S8_Fig/Segmentation result of AMBBEM with three FCNs in test set 2/AMBBEM/Label_61.png]

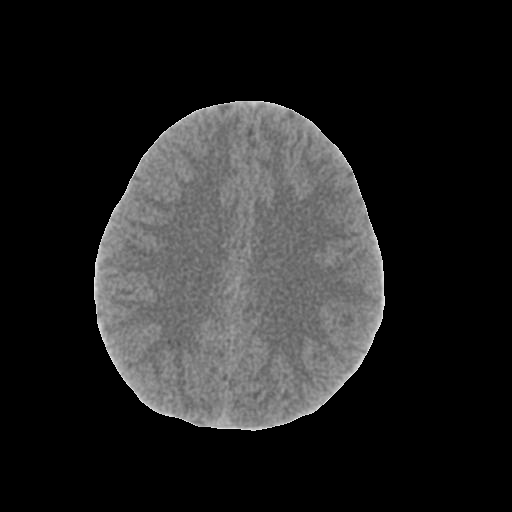

Supplement: S1 Fig — (ZIP) [file pone.0295536.s008.zip › S8_Fig/Segmentation result of AMBBEM with three FCNs in test set 2/AMBBEM/Label_62.png]

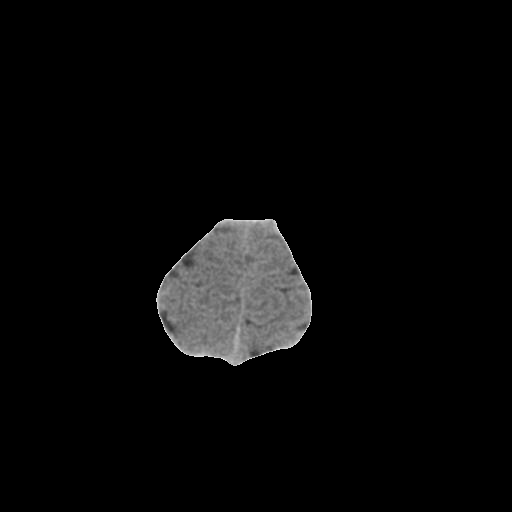

Supplement: S1 Fig — (ZIP) [file pone.0295536.s008.zip › S8_Fig/Segmentation result of AMBBEM with three FCNs in test set 2/AMBBEM/Label_63.png]

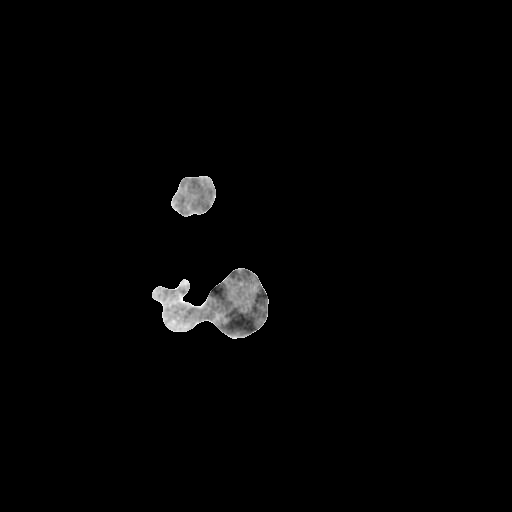

Supplement: S1 Fig — (ZIP) [file pone.0295536.s008.zip › S8_Fig/Segmentation result of AMBBEM with three FCNs in test set 2/AMBBEM/Label_64.png]

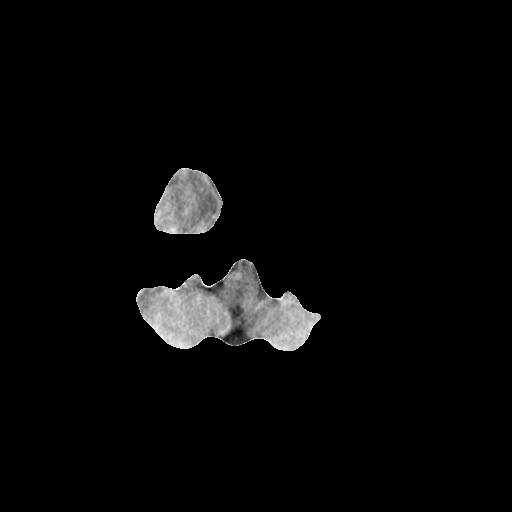

Supplement: S1 Fig — (ZIP) [file pone.0295536.s008.zip › S8_Fig/Segmentation result of AMBBEM with three FCNs in test set 2/AMBBEM/Label_65.png]

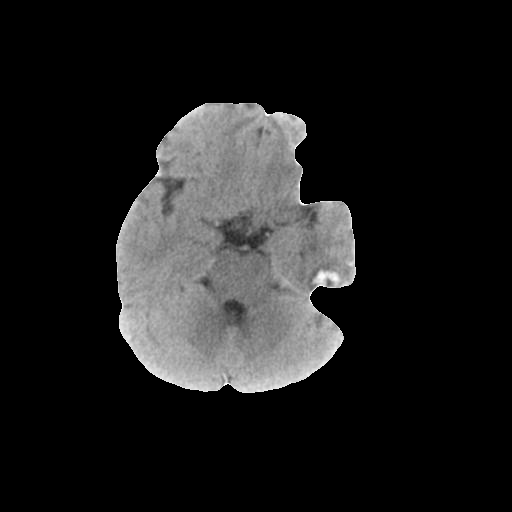

Supplement: S1 Fig — (ZIP) [file pone.0295536.s008.zip › S8_Fig/Segmentation result of AMBBEM with three FCNs in test set 2/AMBBEM/Label_66.png]

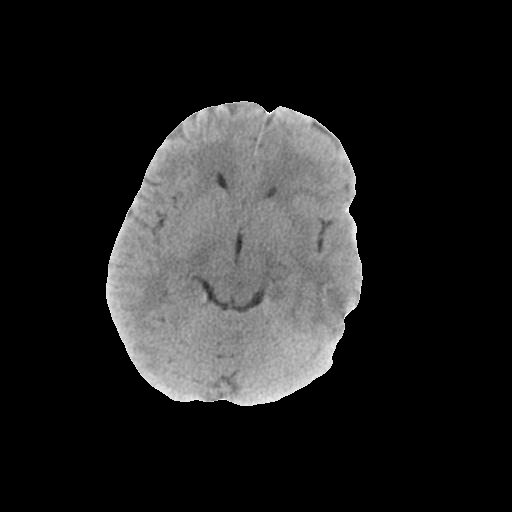

Supplement: S1 Fig — (ZIP) [file pone.0295536.s008.zip › S8_Fig/Segmentation result of AMBBEM with three FCNs in test set 2/AMBBEM/Label_67.png]

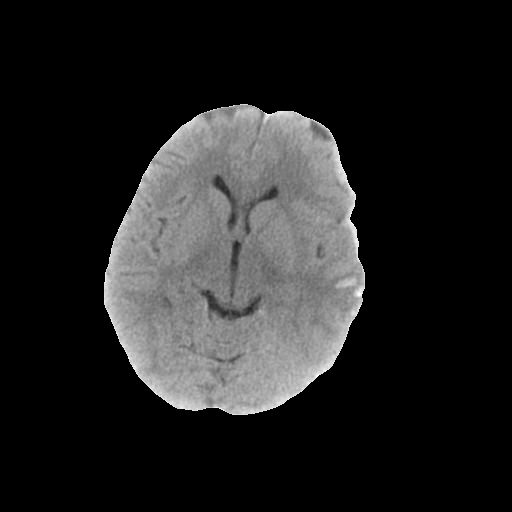

Supplement: S1 Fig — (ZIP) [file pone.0295536.s008.zip › S8_Fig/Segmentation result of AMBBEM with three FCNs in test set 2/AMBBEM/Label_68.png]

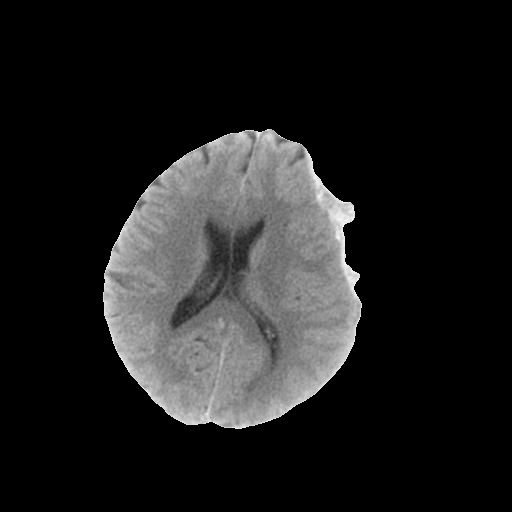

Supplement: S1 Fig — (ZIP) [file pone.0295536.s008.zip › S8_Fig/Segmentation result of AMBBEM with three FCNs in test set 2/AMBBEM/Label_69.png]

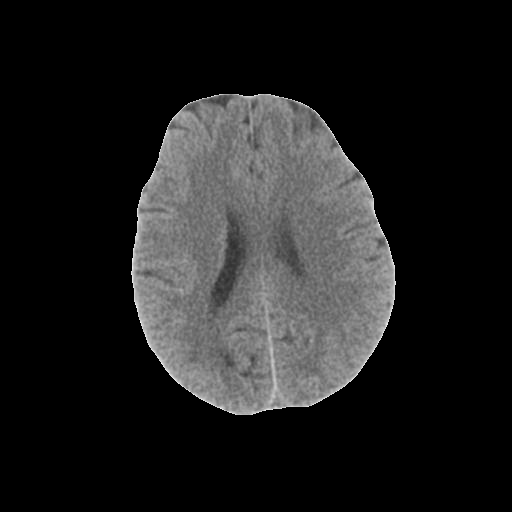

Supplement: S1 Fig — (ZIP) [file pone.0295536.s008.zip › S8_Fig/Segmentation result of AMBBEM with three FCNs in test set 2/AMBBEM/Label_7.png]

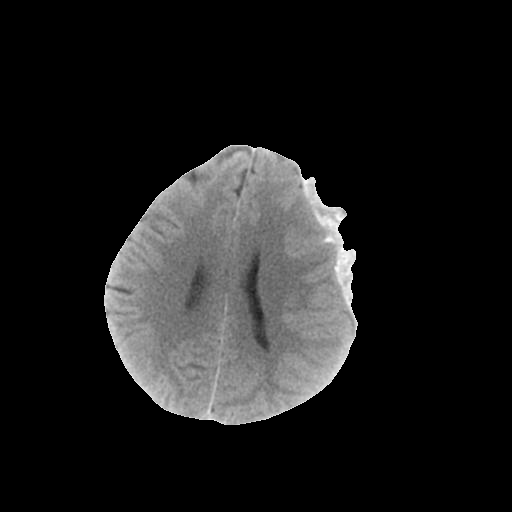

Supplement: S1 Fig — (ZIP) [file pone.0295536.s008.zip › S8_Fig/Segmentation result of AMBBEM with three FCNs in test set 2/AMBBEM/Label_70.png]

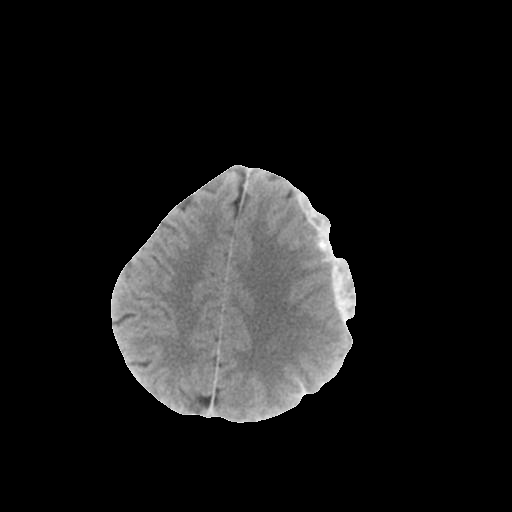

Supplement: S1 Fig — (ZIP) [file pone.0295536.s008.zip › S8_Fig/Segmentation result of AMBBEM with three FCNs in test set 2/AMBBEM/Label_71.png]

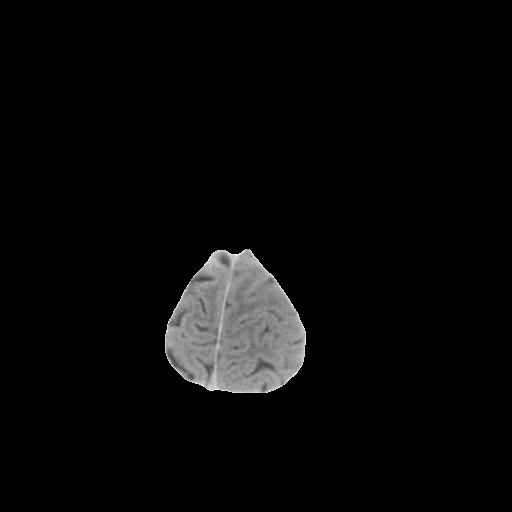

Supplement: S1 Fig — (ZIP) [file pone.0295536.s008.zip › S8_Fig/Segmentation result of AMBBEM with three FCNs in test set 2/AMBBEM/Label_72.png]

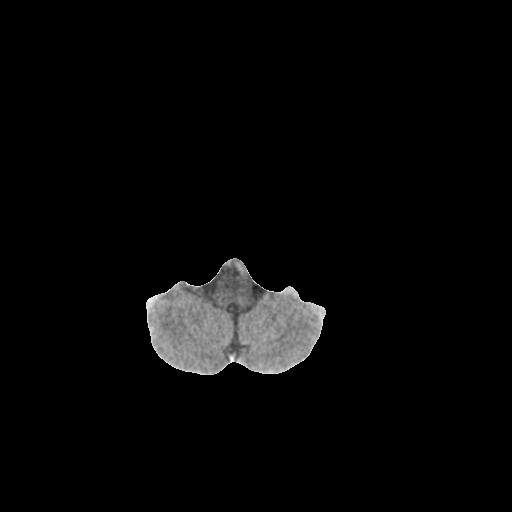

Supplement: S1 Fig — (ZIP) [file pone.0295536.s008.zip › S8_Fig/Segmentation result of AMBBEM with three FCNs in test set 2/AMBBEM/Label_73.png]

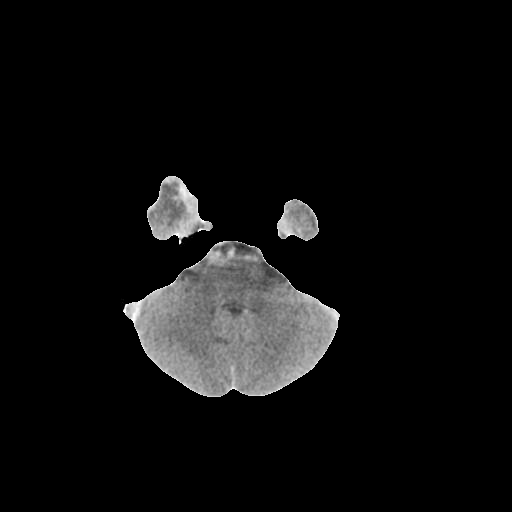

Supplement: S1 Fig — (ZIP) [file pone.0295536.s008.zip › S8_Fig/Segmentation result of AMBBEM with three FCNs in test set 2/AMBBEM/Label_74.png]

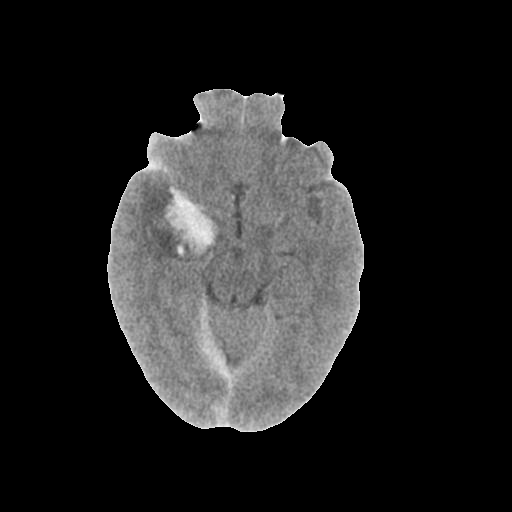

Supplement: S1 Fig — (ZIP) [file pone.0295536.s008.zip › S8_Fig/Segmentation result of AMBBEM with three FCNs in test set 2/AMBBEM/Label_75.png]

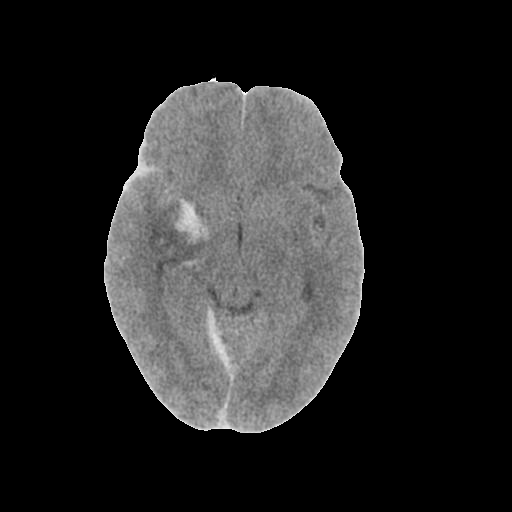

Supplement: S1 Fig — (ZIP) [file pone.0295536.s008.zip › S8_Fig/Segmentation result of AMBBEM with three FCNs in test set 2/AMBBEM/Label_76.png]
