# Supplementary material for: Adaptive mask-based brain extraction method for head CT images
Source: PLoS One. 2024 Mar 11;19(3):e0295536. doi: 10.1371/journal.pone.0295536 (PMC10927156; doi:10.1371/journal.pone.0295536)
Supplement: Attachment — Submitted filename: Response to Reviewers.docx [file pone.0295536.s009.docx]

**Dear Editors,**

Many thanks to the editor and reviewers for their valuable comments. We have adjusted our work accordingly based on your comments and those of the two reviewers, and the adjustments are shown in ' Revised Manuscript with Track Changes,' 'Manuscript,' and 'Cover Letter, ' respectively, according to your request. Below we detail the changes we have made to each of the recommendations.

First, for the five editorial requirements, we made the following adjustments:

**1.** Please ensure that your manuscript meets PLOS ONE's style requirements, including those for file naming.

**Our response：** We apologize for the formatting issues with the manuscript. We have revised the paper according to the PLOS ONE style template. We refer to the latest published articles in PLOS ONE for some unsure details of the modification. If formatting issues still need to be revised, please give us your specific suggestions, and we will make modifications as soon as possible according to your requirements.

**2.** Please note that PLOS ONE has specific guidelines on code sharing for submissions in which author-generated code underpins the findings in the manuscript. In these cases, all author-generated code must be made available without restrictions upon publication of the work.

**Our response：** We understand and support the journal’s requirements and are willing to actively cooperate with the related work. The source code, dataset, and mathematical model are put into the supporting information. If specific operations are required in the future, we will actively cooperate.

**3.** Please state what role the funders took in the study.

**Our response:** Based on the actual situation, we provide a more detailed description of the funders’ situation in the cover letter.

**4.** We note that you have provided funding information that is not currently declared in your Funding Statement. However, funding information should not appear in the Acknowledgments section or other areas of your manuscript. We will only publish funding information present in the Funding Statement section of the online submission form.

**Our response:** We removed the funding information from the manuscript and added a statement to the cover letter upon request.

**5.** Please upload a copy of Supporting Information [**S1** **File**](http://www.plosone.org/article/fetchSingleRepresentation.action?uri=info:doi/10.1371/journal.pone.0261315.s001) which you refer to in your text on page 10.

**Our response:** We apologize for failing to upload support information in time! We have reorganized the support information and uploaded them one by one. If there is still a need for additional information, we will be sure to upload it upon request.

In response to reviewer #1's four comments, we made the following changes:

**1.** The introduction section should be re-write based on the introduction of the field as well motivation and problem statement as well as contribution of the work. The authors can get ideas from the following papers. “ Enhancement of Medical Images through an Iterative McCann Retinex Algorithm: A Case of Detecting Brain Tumor and Retinal Vessel Segmentation” YE Almalki, NA Jandan, TA Soomro, A Ali, P Kumar… - Applied Sciences, 2022.
“Image segmentation for MR brain tumor detection using machine learning: A Review,” TA Soomro, L Zheng, AJ Afifi, A Ali, S Soomro, M Yin… - IEEE Reviews in Biomedical Engineering, 2022.
“Neural network based denoised methods for retinal fundus images and MRI brain images,” TA Soomro, J Gao - 2016 International Joint Conference on Neural …, 2016.

**Our response:** First of all, we acknowledge and appreciate the reviewer's suggestion and realize the shortcomings of the introduction section and that drawing on this suggestion does make the introduction section more organized. Therefore, we read the recommended articles in detail according to the reviewers’ suggestions and used some of them for reference. Finally, we have rewritten the introduction according to the proposal to provide an introduction to the current state and problems of the field and to describe specifically the motivation for our work and the corresponding contributions.

**2.** A related work section should be introduced.

**Our response** According to this recommendation, we provide an overview of the relevant literature and studies in the introduction, outlining in detail the implications, gaps, and problems of the existing work. And in the discussion section of the article, we position our study.

**3.** The proposed method is the combination of multiple existing methods; author should explain the purpose of use these methods in this research along with their contribution.

**Our response:** After reading this comment, we realized the shortcomings of the manuscript and the necessity to revise it.AMBBEM, as an integration algorithm, an introduction to the relevant methods used is necessary. Therefore, we present the purpose and contribution of the methods used separately in the last part of Section 2.

**4.** The results section should be improved and compared with other recently proposed techniques and why only CT images, why do they not use MRI images also?

**Our response** We sincerely thank you for this suggestion and acknowledge and understand it. We have revised the experimental results according to your suggestion. We have again investigated the current state of research in this field, and we can confirm that U-net, SegNet, and Deeplabv3+ are widely used in medical image segmentation with good segmentation results. Further, according to the paper "Robust brain extraction tool for CT head images" (Neurocomputing. 2020;392: 189-195. doi:10.1016/j.neucom.2018. 12.085) and other papers, for brain extraction of head CT images, the U-net and SegNet models show good robustness, accuracy, and segmentation speed, which are superior to other existing methods. In our work, considering many model parameters such as U-net and SegNet, the segmentation speed is relatively slow, so we aim to improve the segmentation speed while ensuring good robustness and accuracy. Therefore, combined with the above situation and modification suggestions, we additionally added the SegNet model as a comparison object in the experimental results section. And in the discussion section, AMABBEM is compared theoretically with existing traditional image segmentation methods and the secondary development of medical image post-processing software to analyze various methods objectively. Why not use MRI images? It is undeniable that researchers at home and abroad have done more research on brain extraction of MRI images and proposed more methods. For the testing of MRI images and the comparison with the latest techniques, we are working on the next one. Finally, if this revision still needs to be improved, we are willing to make further changes based on the reviewers' comments.

In response to reviewer #2's five major comments and six minor comments, we made the following changes:

First of all, for the five major comments proposed:

**1.** The AlexNet is a classic and sound network, but not perfect today. Have you ever tried some relatively SOTA classification networks?

**Our response:** We are very grateful to the reviewers for this suggestion, which has broadened our thinking and improved our algorithm to some extent. Based on this suggestion, we trained and tested AlexNet, VGG19, RestNet50, and squeezeNet networks, compared and analyzed them in the experimental and discussion sections, and then selected the optimal network model for the classification task of CT images based on the comparison results.

**2.** The Alexnet is improved by batch normalization. How about the U-net using batch normalization?

**Our response:** This suggestion is complementary to the first suggestion and helps a lot to improve the manuscript. According to this suggestion, we introduce the method to improve AleNet in the algorithm design section, compare the modified version of AleNet with AleNet in the experimental section, and analyze the experimental results in the discussion section.

**3.** It is unfair that the AlexNet network used 5000 images for training while U-net and Deeplabv3 used 1400 images.

**Our response:** We thank the reviewers for this suggestion, which makes the article more rigorous and convincing. Based on this suggestion, we have redone the relevant experiments, using the same training set for six CNN models and three FCN models.

**4.** In experiments, the training settings, such as optimizer, learning rate, BatchSize, epochs et al., are identical for Unet and Deeplabv3+. Do they both converge and get the best results?

**Our response:** We sincerely thank the reviewers for this suggestion, according to which we tested different parameter settings in the training of CNN and FCN models and selected the optimal ones. In the experimental section of the manuscript, we present the training settings in detail.

**5.** Although the U-net network is widely used in medical image segmentation, it is not designed for segmenting CT brain images. Have you ever compared the proposed method with other methods?

**Our response：** I sincerely thank the reviewer for this suggestion, and I also acknowledge and understand this suggestion. We have revised the experimental results according to your suggestion. We have again investigated the current state of research in this field, and we can confirm that U-net, SegNet, and Deeplabv3+ are widely used in medical image segmentation with good segmentation results. Further, according to the paper "Robust brain extraction tool for CT head images" (Neurocomputing. 2020;392: 189-195. doi:10.1016/j.neucom.2018. 12.085) and other papers, for brain extraction of head CT images, the U-net and SegNet models show good robustness, accuracy, and segmentation speed, which are superior to other existing methods. In our work, considering a large number of model parameters such as U-net and SegNet, the segmentation speed is relatively slow, so we aim to: further improve the segmentation speed while ensuring good robustness and accuracy. Therefore, combined with the above situation and modification suggestions, we additionally added the SegNet model as a comparison object in the experimental results section. And in the discussion section, AMABBEM is compared theoretically with existing traditional image segmentation methods and the secondary development of medical image post-processing software to analyze various methods objectively. The latest technology is mostly applied to MRI images. For the expansion of the application of AMABBEM (application to MRI images) and the comparison with the latest technology, we are working on it in the next work. Finally, if this revision still does not meet the relevant requirements, we are willing to make further revisions based on the reviewers' comments.

Finally, for the 6 Minor comments:

**1.** Figure 6 is upside down, and "The algorithm designs a second loop as shown in Figure 7" actually means in Figure 6

**Our response:** We are very sorry for this error. We have replaced most of the diagrams in the manuscript, and the replacement is clearer and more concise.

**2.** All Figure numbers are mismatched in the text from Figure 6.

**Our response：** We are very sorry for such an error. We have re-edited the relevant content and checked it several times to ensure the figures correspond to the chart.

**3.** Lack of explanation for some settings, such as why cycle 1 is five times instead of 3 or 10 times, and Q is 0.22, and so on. Moreover, the results of Alexnet with batch normalization or without are not compared.

**Our response：** Thank you very much for this suggestion! In our algorithm design, q and the number of cycles are introduced in detail. The experimental part compares the modified version of Ale Net with Ale Net, and the experimental results are analyzed in the discussion section.

**4.** Some figures and formulas are difficult to read. Highlight the best results of each evaluation metric in tables.

**Our response:** We apologize that the numbers and formulas were difficult to read. We have re-edited the relevant content to make it more specific and concise. For the experimental results, we select the best results for each algorithm upon request.

**5.** Some sentences are hard to follow, so language and grammar editing is highly recommended.

**Our response:** We apologize for the sentence description issue. We have re-edited the entire article and inspected it several times to ensure grammatical correctness and clarity of language presentation.

**6.** PLOS authors have the option to publish the peer review history of their article ([what does this mean?](https://journals.plos.org/plosone/s/editorial-and-peer-review-process#loc-peer-review-history)). If published, this will include your full peer review and any attached files.

**Our response:** For this suggestion, we thank the reviewers for their kind words! However, we still need to understand peer review well after reading the relevant guidelines, but we are willing to actively cooperate with the reviewers and editors if peer review helps the reviewers and editors.

Finally, we would like to express again our deep appreciation to the editors and reviewers for their valuable suggestions. We have significantly benefited from your suggestions. If the manuscript still needs to be modified, we are willing to revise it according to your requests. Finally, we wish you all the best in your review!

Yours sincerely,

Ding-Yuan Hu
