# Supplementary material for: Adaptive mask-based brain extraction method for head CT images
Source: PLoS One. 2024 Mar 11;19(3):e0295536. doi: 10.1371/journal.pone.0295536 (PMC10927156; doi:10.1371/journal.pone.0295536)
Supplement: Attachment — Submitted filename: Response to Reviewers.docx [file pone.0295536.s010.docx]

Dear Editor：

I am very grateful to the editor for replying to my busy schedule, and I would like to thank the reviewers again for their valuable comments. In publishing a paper, receiving comments and guidance from the reviewers is crucial for better solving practical problems. We are lucky because the reviewers' comments are professional and rigorous, which opened our eyes and standardized the manuscript. We answered each reviewer's comments and adjusted the manuscript and algorithms. Our specific answers and revisions are listed below:

First of all, we carefully reviewed the references for the journal's requirements for a reference list, combined with the actual situation of manuscript changes. We made the following adjustments:

1. To further highlight the characteristics of CT imaging and to facilitate the reader's understanding of the latter. We replaced the original reference ' Parameter Calibration and Image Reconstruction of CT System ' with ' Improving Sensitivity on Identification and Delineation of Intracranial Hemorrhage Lesion Using Cascaded Deep Learning Models '.
2. Because the manuscript excludes the use of AlexNet, we also exclude the reference to the literature "ImageNet Classification with Deep Convolutional Neural Networks."
3. The manuscript also tested the EfficientNet network and used the SE and CBAM modules. I have added three articles: ' EfficientNet: Rethinking Model Scaling for Convolutional Neural Networks, '' CBAM: Convolutional Block Attention Module ' and ' Squeeze-and-Excitation Networks '.
4. To make it easier for readers to understand the characteristics of the region growing algorithm, we also cite the literature "A review on brain tumor segmentation of MRI images."

Second, we responded to reviewer #1's comment as follows:

1. no comments. All the comments are address. The comments have been addressed, but I was unable to locate the highlighted manuscript for tracking. I recommended this paper after reviewing the editable PDF format, as the highlighted PDF is more suitable for the reviewer

Our response: We are very grateful to the reviewer for this comment and for recognizing the last revision. Further, we apologize for the reviewer's inability to locate the highlighted manuscript and thus follow it up; this time, we have made the revision marks on the original manuscript much clearer.

Third, we have made the following responses and revisions to the six comments made by reviewer 2:

1. AlexNet, VGG19, ResNet50, and squeezeNet mentioned in response are all classic classification networks proposed before 2016. How about the results for methods in recent years, such as EfficientNet, ResNeXt?

Our response: Thank you very much to the reviewer for this comment! This comment once again broadens our horizons, and introducing more excellent networks further improves the superiority of our algorithm. As the reviewer said, AlexNet, VGG19, ResNet50, and squeezeNet are indeed relatively old, and the subsequent more excellent EfficientNet and ResNeXt are more worth trying. Therefore, we also tested the EfficientNet network and again delved into the ResNet family of networks. We found that ResNeXt was obtained by improving ResNet while also noting that SE_ResNet50... and other networks. Finally, we try to improve on ResNet50 by adding a CBAM module to the Stem Block session of ResNet50 and then adding an SE module to each of the four Bottleneck sessions. Then, we fine-tune the training set and leave the test set 1 unchanged. Finally, VGG19, ResNet50, squeezeNet, EfficientNet, and the improved ResNet50 are trained on the training set; during the training process, multiple sets of optimizers and hyperparameters are tested for each network model, and the best optimizer and hyperparameters are selected. The experimental results show that the improved ResNet50 gets a significant improvement in both Accuracy and AP values.

In addition, we again performed further inspection and correction of test set 2 with the assistance of a radiologist, but of course, the adjustments were minimal. Finally, each FCN and AMBBEM were tested again. The results showed that the segmentation speed of AMBBEM was further improved after the replacement of the CNN, which was attributed to the improved resnet50 with higher accuracy and a lower number of parameters than the previous CNN, which resulted in a faster classification speed!

Of course, the article has been adjusted accordingly in the Abstract, Algorithm Design, Experimental Results, and Talking About sections because of the adjustments to the CNN network.

2. The caption of subfigure "d" in Fig 3 is missing.

Our response: We apologize for this comment! We have corrected this error.

3. In Figure 6, is the "Extract objects from an image using area" module parallel to the main path? And what is "single-region distribution"?

Our response: We thank the reviewer for this comment! This evaluation made us aware of errors and deficiencies in Figure 6 and its presentation. We have revised Figure 6. In addition, the term "single-region distribution" indicates that the brain tissue is a single block in a head CT image, as opposed to the brain being distributed in multiple blocks. Finally, we have made adjustments in the presentation of the manuscript to address these two issues to facilitate better understanding by the readers.
4. Describe the input sizes of the first four models among the six CNN models and briefly analyze the results. Additionally, if the regular AlexNet and VGG19 also use the image with the size of 512×512×3, how would the results be?

Our response: Much respect to the reviewers for their dedication and seriousness! We should describe the CNN networks' input sizes in further detail. In addition, we also tested the normal AlexNet and VGG19 networks with the input size of 512×512×3, respectively. The results show that, due to the setting of the three fully connected layers in the VGG19, the input layer size is fixed (224×224×3), and the input size can not be changed. For the normal AlexNet, the adjustment of the input size does not have any significant effect on it. To show the experimental conclusions more concisely. We only offer the test results of VGG19, ResNet50, squeezeNet, EfficientNet, and the improved ResNet50 and exclude the results of ordinary AlexNet and improved AlexNet from the manuscript content.
5. The format of Mask 1 should be consistent. Some are "mask1", and others are "Mask 1". So as the "Mask2".

Our response: I thank the reviewer for this comment and apologize for this oversight; we have standardized the formatting involved.
6. There is no caption for many figures with sub-figures.

Our response: We have been discussing this evaluation carefully and have always found the reviewer's suggestion more reasonable. We have added a description of the images with subplots based on the format of the latest published paper in the journal.

7. In the section "Re-segmentation of the mask," what is the "region growing algorithm" for the re-segmentation of mask 2?

Our response: Thanks to the reviewers for this comment! We apologize for the lack of clarity here. Region growing algorithm is an image segmentation technique. The basic idea will be to combine pixels with similar criteria to form regions based on certain discriminative criteria. The main step is to find a seed pixel for each region to be segmented as a starting point for growth, and then according to a certain discriminative criterion, the similar pixels around the seed pixel are discriminated, and the pixels with higher similarity are merged so that they germinate and grow like a seed. We have adapted the presentation part of the region growing algorithm in the article, in addition to adding references to facilitate the reader's understanding.

In addition, we have negotiated and hope to make the following adjustments to the paper:

1. The paper was published with equally significant contributions from Shiya Qu, so we would like to list Shiya Qu as the 1st set of equal contributors. In addition, we would like to add Qingyan Zhang (radiologist) as the sixth author, considering that he has provided a lot of help in the process of publication and revision of the paper. Of course, this change must be made with the editor's permission. We are willing to follow the editor's arrangement if the editor feels something inappropriate, and the editor's arrangement will prevail.

Finally, we checked and proofread the article several times and adjusted some words and grammar. For the supporting material, we were keen to follow the request to share experimental materials. However, some of the datasets were too large, and multiple uploads failed. For this reason, we can only split the training set (S1) into four parts for uploading. The file "S1_CNN_Training set" contains the dataset required for CNN training, which has already been categorized and can be used directly. File "S1_FCN_Training set_Label" contains the labels required for FCN model training, "S1_FCN_Training set_Set_1" and "S1_FCN_Training set_Set_2" files together contain 1400 images required for FCN model training, which need to be combined into one piece after downloading. Files S2 through S5 are as described in the manuscript. Where S5 contains instructions for use and an improved ResNet50 network model in addition to the code. Also, we mention in the manuscript that readers can also direct questions about the experimental material to the corresponding author.
